# Novel Low Abundance and Transient RNAs in Yeast Revealed by Tiling Microarrays and Ultra High–Throughput Sequencing Are Not Conserved Across Closely Related Yeast Species

**DOI:** 10.1371/journal.pgen.1000299

**Published:** 2008-12-19

**Authors:** Albert Lee, Kasper Daniel Hansen, James Bullard, Sandrine Dudoit, Gavin Sherlock

**Affiliations:** 1 Department of Genetics, Stanford University, Stanford, California, United of States of America; 2 Division of Biostatistics, School of Public Health, University of California Berkeley, Berkeley, California, United States of America; Yale University, United States of America

## Abstract

A complete description of the transcriptome of an organism is crucial for a comprehensive understanding of how it functions and how its transcriptional networks are controlled, and may provide insights into the organism's evolution. Despite the status of *Saccharomyces cerevisiae* as arguably the most well-studied model eukaryote, we still do not have a full catalog or understanding of all its genes. In order to interrogate the transcriptome of *S. cerevisiae* for low abundance or rapidly turned over transcripts, we deleted elements of the RNA degradation machinery with the goal of preferentially increasing the relative abundance of such transcripts. We then used high-resolution tiling microarrays and ultra high–throughput sequencing (UHTS) to identify, map, and validate unannotated transcripts that are more abundant in the RNA degradation mutants relative to wild-type cells. We identified 365 currently unannotated transcripts, the majority presumably representing low abundance or short-lived RNAs, of which 185 are previously unknown and unique to this study. It is likely that many of these are cryptic unstable transcripts (CUTs), which are rapidly degraded and whose function(s) within the cell are still unclear, while others may be novel functional transcripts. Of the 185 transcripts we identified as novel to our study, greater than 80 percent come from regions of the genome that have lower conservation scores amongst closely related yeast species than 85 percent of the verified ORFs in *S. cerevisiae*. Such regions of the genome have typically been less well-studied, and by definition transcripts from these regions will distinguish *S. cerevisiae* from these closely related species.

## Author Summary

The budding yeast *Saccharomyces cerevisiae*, because of the relative ease of its genetic manipulation and its ease of handling in the laboratory, has long served as a model on which studies in higher organisms have been based. To more fully understand how eukaryotic cells express their genomes, we sought to identify RNA species that are transcribed at very low levels or that are rapidly degraded. We created mutants deficient in the ability to degrade RNA, with the expectation that this would increase the relative abundance of such RNAs, and then used high-resolution microarrays and sequencing technologies to locate and identify from where these RNAs are transcribed. Using this approach, we have identified 365 transcripts that do not appear in the most current list of annotated *S. cerevisiae* RNA transcripts; of these, 185 are unique to our study. Many of these novel transcripts derive from regions of the genome that are poorly conserved between *S. cerevisiae* and other closely related yeast species, suggesting that these RNAs may play an important role in the divergent microevolution of *S. cerevisiae*.

## Introduction

Twelve years ago, in a landmark study resulting from the collaborative work of hundreds of scientists around the world, the budding yeast *Saccharomyces cerevisiae* became the first eukaryote to have its genome fully sequenced [1]. The initial analysis of the genome utilized the following (necessarily) arbitrary rules for defining whether an Open Reading Frame (ORF) was a protein-coding gene (a “genic ORF”) or not: 1) a genic ORF had to start with ATG and have at least 100 sense codons, and 2) if two ORFs of more than 100 sense codons overlapped one another by more than 50% of their lengths, then the longer was picked as being a genic ORF, while the shorter was discarded. In this way, it was determined that the sequence of 12,068 kilobases contained 5,885 potential protein-coding genes. In addition, non-protein-coding genes consisting of approximately 140 ribosomal RNA genes, 40 small nuclear RNA genes, and 275 transfer RNA genes were identified using various criteria, resulting in a total of approximately 6,340 genes.

Early analyses of the predicted protein-coding genes showed that about 35% had no known function or homolog [2], leading to questions about the validity of the rules used to identify genic ORFs. Various algorithmic methods have predicted fewer genes in the yeast genome than the originally predicted number of 6,340, based on a variety of criteria [3]–[7], while other methods have found and verified new ones, especially non-coding genes [8],[9]. Comparative genomics [10]–[12], and various experimental methods [13]–[17] have also resulted in significant changes to the primary annotation of the yeast genome, introducing hundreds of newly predicted genic ORFs, while marking many others as ‘dubious’. However, new genes added by one study are frequently marked as ‘dubious’ by another, as recorded within the *Saccharomyces* Genome Database (SGD) [18], indicating the speculative nature of many of these annotations. Additionally, a recent study [19] has shown that the use of comparative genomics alone to determine whether or not a genomic region is likely to harbor a genic ORF can result in false negatives, since many transcribed elements may not be conserved across even closely related species. It has been suggested that such ORFs may be important for the micro-evolutionary divergence between species. Clearly, even in a genome as simple as, and containing as few introns as that of *S. cerevisiae*, it is still not straightforward to identify all of the genes simply based on the DNA sequence.

Hybridization of RNA to tiling microarrays (microarrays containing overlapping, offset probes that tile across the entire genome) has been used to generate genome-wide transcript profiles and to detect previously unannotated transcripts. While this technique has its own caveats, it overcomes the limitations of many previous attempts to find undiscovered transcripts, by providing direct experimental support with high-resolution data. Tiling array studies have revealed more than 5,000 novel transcripts in *Arabidopsis* [20] and rice [21], and more than 10,000 previously unknown transcripts in human cells [22]–[24]. In yeast, tiling array experiments performed by David et al. [25], using RNA isolated from a single experimental condition, identified almost 800 novel (i.e., not annotated in SGD [18]) transcripts.

Recently, Miura et al. [26], also working with *S. cerevisiae*, performed large-scale sequencing of vector-capped cDNA clones [27],[28] from two cDNA libraries to accurately map over 11,000 transcriptional start sites (TSSs). Of these predicted transcripts, 667 were novel (many of which were also identified by David et al.), and contained ORFs corresponding to 100 amino acids or less and thus would have been missed in the original annotation. Furthermore, they discovered 45 new introns, 367 novel antisense transcripts, and showed that most yeast genes have two or more TSSs, demonstrating that the transcriptional potential of the yeast genome is more complex than previously thought. In total, their analysis detected only 3,599 of the more than 6,000 currently annotated genic ORFs, suggesting either that many genes were missing from their cDNA library, or that many of the annotated genic ORFs are not correct.

Recent advances in sequencing technology [29]–[32] have allowed an unprecedented look at the transcriptome, using a method known as RNA-Seq [33]. This method can yield millions of sequence reads from cDNA libraries, and has been used to discover and validate transcribed regions of the genome in various organisms [34]–[36]. Most recently, RNA-Seq has been used to identify additional transcripts expressed in *S. cerevisiae* growing in rich medium [37], and transcripts expressed in *S. pombe* growing under several different conditions, including a meiotic time course [38]. From tens of millions of sequence reads, 204 novel transcripts were identified in *S. cerevisiae*, and 453 novel transcripts in *S. pombe*; additionally, many transcript boundaries were refined, and novel introns identified. The functions of these novel transcripts remain unknown, with few expected to be protein-coding [38].

**Figure 1:**
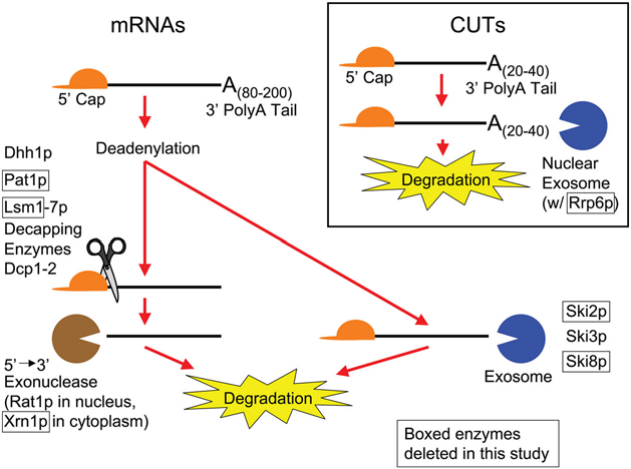
A summary of the degradation pathways for mRNAs and cryptic unstable transcripts (CUTs). Enzymes in boxes were deleted in this study in order to stabilize RNA transcripts.

There exist various mechanisms by which RNA is processed, surveyed, and turned over. In *S. cerevisiae*, there are two major pathways that play a role in the decay of mRNAs in the cytoplasm, both of which involve deadenylation (Figure 1). In the first pathway, deadenylation is followed by the removal of the 5′ m7G cap by Dcp1p and Dcp2p, which is then followed by degradation in the 5′ to 3′ direction by Xrn1p [39]–[44]. In addition to Dcp1p and Dcp2p, there exists a group of proteins that function as activators for decapping, including Pat1p, the Lsm1-7p complex, and Dhh1p [45]–[49]. In the second pathway, deadenylated mRNAs are degraded in the 3′ to 5′ direction by the exosome and the Ski complex (consisting of Ski2p, Ski3p, and Ski8p) [50],[51]. In the nucleus, mRNAs that are unspliced, improperly processed, and/or otherwise unable to leave the nucleus are degraded in pathways using the same machinery [52]–[55]. Rrp6p, a nuclear-only component of the exosome which has 3′ to 5′ exonuclease activity [56],[57], plays a major role in the nuclear degradation of mRNAs as well as CUTs ([58] and reviewed in [59],[60]).

As described above, genome-wide screens for novel transcripts have revealed the existence of many non-coding, intergenic, and/or antisense RNAs. Such RNAs are poorly understood, sometimes being referred to as ‘transcriptional noise’, whose expression may be initiated from inadvertent binding of RNA polymerase complexes to DNA sequences that bear resemblance to ‘real’ transcriptional promoters. In *S. cerevisiae*, some of these transcripts are rapidly degraded and have been labeled as cryptic unstable transcripts or CUTs (Figure 1; [58] and reviewed in [59],[60]). While the roles of these CUTs are unclear, the mechanism by which these RNAs are degraded has been elucidated and it has been shown that they are specifically targeted for degradation via polyadenylation by the non-canonical polyadenylation protein Trf4p, a component of the TRAMP complex [58],[61],[62]. Why these RNAs are transcribed at all, and why a specific degradation pathway exists for them in the budding yeast remains speculative.

To identify additional novel transcripts in the yeast *S. cerevisiae*, we have employed both tiling microarrays and RNA-Seq, with the explicit goal of identifying those transcripts that are either short-lived and/or occur in low abundance. Such transcripts may include previously unrecognized protein-coding transcripts and non-coding transcripts, as well as cryptic unstable transcripts and ‘transcriptional noise’. To allow better detection of these types of transcripts, we have analyzed RNA isolated from three strains containing various combinations of deletions of six genes that play a role in RNA processing (*RRP6*, *XRN1*, *PAT1*, *LSM1*, *SKI2* and *SKI8*), with the hypothesis that the most unstable and/or least abundant transcripts would show the greatest relative change in abundance in such mutants. The mutant-derived RNA was compared to RNA from wild-type cells, using Affymetrix strand-specific tiling microarrays. Novel strand-specific transcripts were identified by segmentation of the relative expression measures from the tiling arrays and subsequently validated using Illumina's Solexa sequencing platform. Using a combined tiling array and RNA-Seq approach, we have identified a total of 365 transcripts that are currently unannotated in SGD. Comparison of our data to various recently published transcriptome studies [25],[26],[37],[63] reveals that of these unannotated transcripts, 185 are novel and unique to our study.

## Results

### Rationale

Our primary goal was the discovery of novel transcripts based on comparing RNA from mutants deficient in RNA degradation pathways to RNA from a wild-type strain. We wanted to provide, in a high-throughput fashion, distinct and complementary lines of evidence for the existence of each putative transcript. We thus selected two technologies as being appropriate for this aim: tiling arrays and high-throughput sequencing. We used the tiling arrays to discover novel transcribed segments, with their strand of origin information. This approach has been used successfully in previous studies [25] and there are well-established computational and statistical methods for analyzing tiling array data. Tiling arrays, as opposed to high-throughput sequencing, provide an even spacing of measurements across the entire genome, making them more amenable to off-the-shelf segmentation algorithms. In addition, an entire population of molecules is hybridized to the microarray, whereas a sequencing based approach is inherently a sampling strategy, limited by the depth to which one can afford to sequence, and by the complexity of the sample being sequenced. However, high-throughput sequencing provides an independent experimental platform well-suited for transcript validation as each read provides distinct evidence for the presence of a transcribed segment.

### Discovery of Novel Transcripts Using Tiling Microarrays

Tiling microarray analysis of mRNA from yeast grown under a diverse set of several different conditions suggested that the greatest fraction of known transcripts are detectable in the presence of high salt (0.8 M NaCl) (our unpublished results); we thus chose high salt as the growth condition used in the experiments described herein. All our deletion strains (the ‘mutant’ strains) and the wild-type strain (see Table 1 for strain details) were shocked with high salt for 30 minutes; total RNA was isolated from each strain, from which a poly A+ RNA sample was also purified, resulting in two different RNA preparations per strain. These RNAs were then labeled and hybridized to both forward and reverse strand Affymetrix yeast genome tiling microarrays (see Materials and Methods).

**Table 1:** Genes deleted and strains used.

Gene	Function
*LSM1*	mRNA decapping factor
*PAT1*	mRNA decapping factor
*RRP6*	exonuclease component of the nuclear exosome
*SKI2*	involved in 3′->5′ exosome mediated mRNA degradation
*SKI8*	involved in 3′->5′ exosome mediated mRNA degradation
*XRN1*	5′->3′ cytoplasmic exonuclease

Strain ID	Deletion	Genotype	*MAT*	Geneticin
GSY1231	WT	*leu2-Δ1*, *ura3-52*, *his3-Δ200*, *trp1-Δ63*	a	Sensitive
GSY1283	*Δrrp6*, *Δlsm1*, *Δpat1*	*leu2-Δ1*, *ura3-52*, *his3-Δ200*, *trp1-Δ63*	a	Resistant
GSY1284	*Δski2*, *Δski8*, *Δrrp6*	*leu2-Δ1*, *ura3-52*, *his3-Δ200*, *trp1-Δ63*	a	Resistant
GSY1289	*Δxrn1*, *Δrrp6*, *Δlsm1*, *Δpat1*	*leu2-Δ1*, *ura3-52*, *his3-Δ200*, *trp1-Δ63*	a	Resistant

Only perfect match (PM) probes mapping uniquely to the genome were used in the analysis; mismatch probes were discarded. In order to correct for probe-specific effects and to detect only those transcripts that were differentially expressed between a mutant and the wild-type, we used as expression measures the log ratio of mutant PM intensities to wild-type PM intensities. We segmented the log ratios using a piecewise constant change point model as implemented in the ‘segment’ function in the R package ‘tilingArray’ [64] from Bioconductor [65]. Following Huber et al., we utilized the Bayesian information criterion (BIC) penalized likelihood to select the number of transcribed segments. Poly A+ RNA and total RNA microarray data were segmented separately. Based on a visual assessment of the resulting segmentation it appeared that BIC overestimated the number of segments (also noted by Huber et al.).

Oversegmentation makes downstream validation of the segments more challenging, as putative segments are judged in pieces as opposed to their entirety. Thus, we post-processed the segmented data to: (1) join adjacent segments with similar expression measures, (2) drop segments that are not differentially expressed, using a threshold of <0.5 on the log_2_ scale, (3) remove segments overlapping known annotation on the same strand, (4) remove segments containing fewer than 5 probes, and (5) remove segments opposite known annotation if they had a log_2_ fold change less than 2, or there was detectable transcription on the opposite strand (see Materials and Methods for a detailed discussion). For the sake of consistency, we will now refer to our post-processed segments as clusters, as they may refer to one or more original segments. After segmentation and post-processing of the tiling microarray data, we identified 892 candidate clusters in the poly A+ RNA data (826 of which were intergenic) and 338 from the total RNA data (324 of which were intergenic). Our criteria in analyzing the microarray data were somewhat liberal, with the aim of being as inclusive as possible; however, we coupled this with more stringent criteria for subsequent validation by sequencing, with the expectation that many of these clusters identified from the tiling microarrays would not be subsequently validated. All subsequent analyses were done at the cluster level.

### Validation of Novel Transcripts Using Ultra−High Throughput Sequencing

Following identification of these clusters from the tiling array data, we sought to validate them using sequencing. The same RNAs harvested for the tiling microarray experiments were used to generate cDNA libraries for ultra high-throughput sequencing on a Solexa 1G Genome Analyzer. Libraries were generated from double polyA purified RNAs (see Materials and Methods) from both the wild-type and the mutant strains, and were run on four lanes each of a Solexa flow cell. Reads that passed Solexa's software filters were aligned to the genome using ELAND, allowing up to two mismatches per read. For subsequent analyses, we retained only reads mapping to a unique location, and in total, we generated more than 50 million uniquely mappable reads across all four strains. The wild-type library generated a total of 14,103,067 uniquely mapped reads from four lanes, the Δ*rrp6*Δ*lsm1*Δ*pat1* mutant library generated 14,745,813 reads, the Δ*ski2*Δ*ski8*Δ*rrp6* mutant library 14,973,577 reads, and the Δ*xrn1*Δ*rrp6*Δ*lsm1*Δ*pat1* mutant library 10,714,094 reads. Following an assessment of the inter-lane variation we combined data across lanes for each strain (see Materials and Methods and Figure S2).

**Figure 2:**
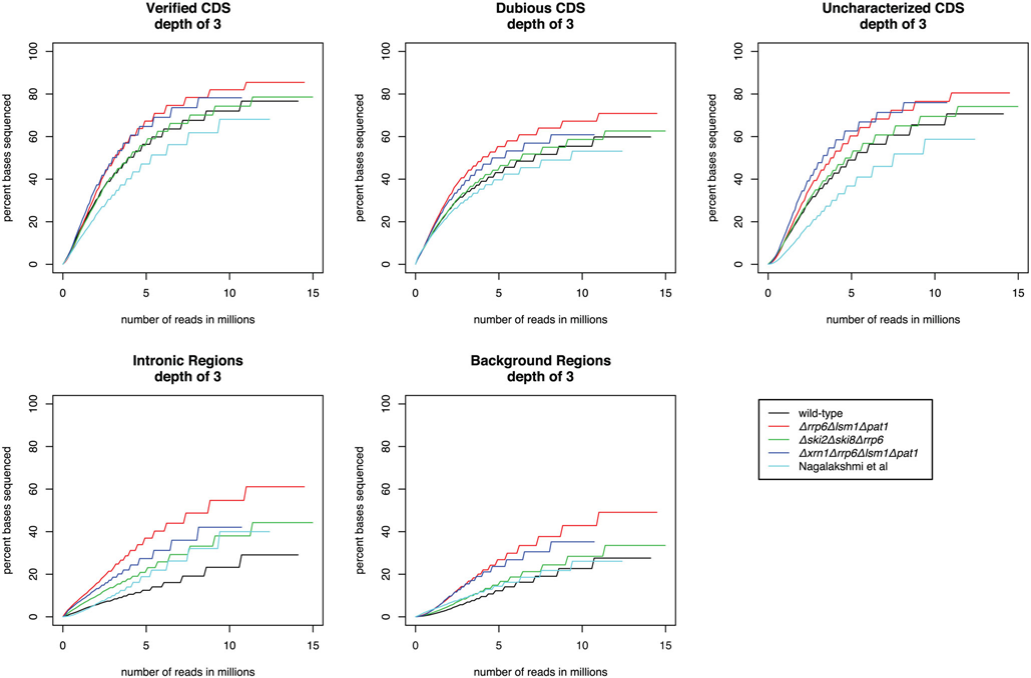
Coverage as determined by Solexa Sequencing. Each line corresponds to a class of genomic region, and for each class, we show the percentage of genomic bases annotated in the class that have been sequenced at a depth of 3 or more, as a function of sequencing depth. Each plot depicts this relationship for one of the 4 datasets considered in this study, as well as the data from Nagalakshmi et al. Verified ORFs, Uncharacterized ORFs, Dubious ORFs, Introns are as defined in SGD, and Background regions are defined in Materials and Methods.

In order to determine whether the sequence reads generated from the cDNA libraries contained sufficient coverage and depth of the transcriptome, we determined the coverage at each base within the following classes: Verified ORFs, Uncharacterized ORFs, Dubious ORFs, Introns, and Background regions. Background regions were defined as regions that were intergenic on both strands, with the following additional regions removed: novel regions identified in David et al., Davis and Ares, Miura et al., and Nagalakshmi et al. [25],[26],[37],[63], as well as putative novel regions identified in this study using the tiling array. For each of these categories we determined the percentage of total bases sequenced to a depth of 3 or greater (see Figure 2 and Figures S3 and S4). For comparison, we have included the publicly available data from Nagalakshmi et al [37].

Figure 2 demonstrates that with an increase in sequencing effort there would be a diminishing return in terms of percentage of bases sequenced to a certain depth. Figure 2 also illustrates that an increase in sequencing effort results in an increase in the percentage of bases sequenced from both background and intronic regions (see discussion). This is the case in our data as well as those of Nagalakshmi et al. This implies that any method for declaring a gene as “detected” must evaluate the data in the context of the reads observed in these regions.

**Figure 3:**
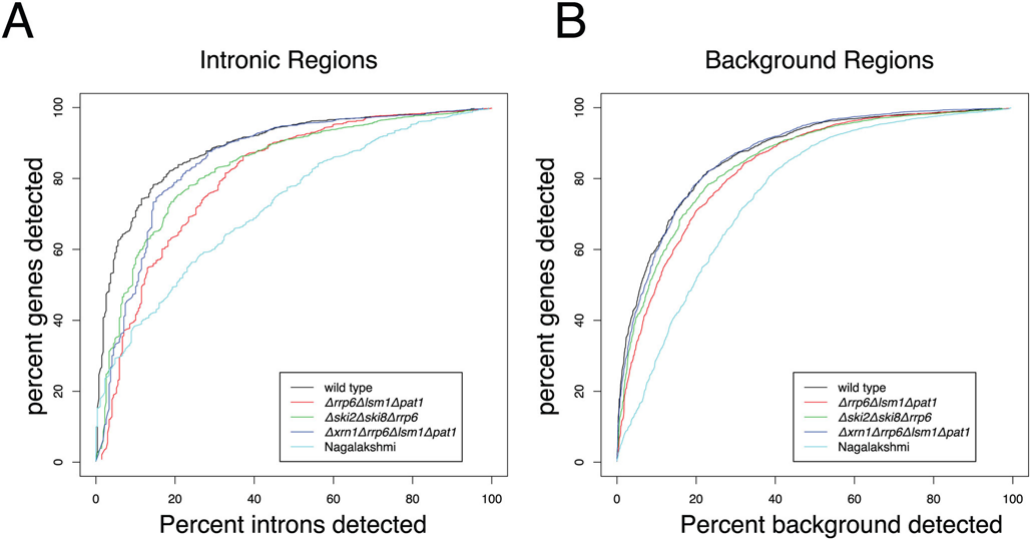
ROC-like curves depicting the relationship between the percentage of detected Verified ORFs and percentage of detected Intronic/Background regions, as the detection threshold varies.

Figure 3 shows ROC-like curves depicting the tradeoff between detecting ORFs and detecting background regions, as we vary the detection cutoff. These plots demonstrate that the choice of a detection cutoff imposes a sample specific tradeoff between detecting annotated ORFs and background regions. For subsequent analyses, we chose a cutoff corresponding to calling 20% of background regions detected. Using this cutoff, we detected on average 75% of the Verified ORFs across all four experiments.

A GO analysis [66] of the Verified ORFs that were not detected above background indicated a significant enrichment for ORFs whose gene products are involved in the cell cycle and sporulation. The lack of sporulation gene expression is not surprising, as the cells would not be expected to be undergoing sporulation under these conditions; as for cell cycle gene expression, presumably the salt shock shuts off the cell cycle, and those transcripts are no longer detected at these thresholds by the time we collected the cells (30 minutes after exposure to salt).

In addition, we also analyzed our sequence reads to look at the dynamic range of detected transcripts. By considering Verified ORFs (>50 unique bp) that were detectable above background in the sequence data, the most abundantly expressed transcript in every mutant, and the wild-type, in terms of number of mapped reads per unique base was that of *HSP12* (*YFL014W*), which is known to be induced under conditions of osmotic stress. Its average number of reads per unique nucleotide was ∼400 in every case. The least abundant transcript was different in each mutant, but with an average number of reads per base of less than 1. Thus, transcript abundances of the Verified ORFs (as measured by sequencing) span at least 3 orders of magnitude (see Table S3 for read counts and RPKMs [33] for all annotated ORFs).

As another measurement of the validity of the sequenced libraries, we determined how many known introns we were able to detect by looking for reads that spanned exon-exon junctions. To detect these intron spanning reads, we identified those reads that mapped to the set of spliced genic ORFs but did not map to the unspliced genome. The wild-type and mutant libraries each generated sequence reads that map to exon-exon junctions, which, when combined, confirm splice junctions in 244 (86%) of the 284 known spliced ORFs reported in the current SGD annotation. In the most extreme case (*RPL28*) we saw 1399 reads that mapped to the exon-exon junction in the data from the Δ*rrp6*Δ*lsm1*Δ*pat1* mutant. Of those forty genes whose exon-exon junctions we failed to detect, two were in mitochondrial genes, and 16 were in Dubious or Uncharacterized ORFs. Of the remaining 22, six of the genes are expressed in meiosis, and fourteen have an initial exon of only a few residues. These were less likely to have been detected by our strategy, as we looked for reads that matched the ORF sequence and not the genome, which would have had to start at a few specific residues to be detected. Subsequent analysis, by inclusion of 5′ UTR sequence to capture such exon boundary spanning reads, was able to identify these remaining introns. Thus, only two Verified ORFs, *YER014C-A/BUD25* and *YPL075W/GCR1*, which were not meiosis specific, failed to have reads detected that spanned their exon junctions. *BUD25* is opposite two other Verified ORFs in the genome, while Nagalakshmi et al [37] also noted that they were unable to identify exon-exon boundary spanning reads for *GCR1*. Indeed, we were able to identify reads that spanned the 5′ exon-intron junction, and the 3′ intron-exon junction, suggesting that the intron is misannotated.

We then examined an integrated dataset consisting of our tiling array and sequencing data as well as data from other published high-resolution studies. Various statistics of the potentially novel transcripts were computed to determine our proposed changes to the set of transcripts produced from the yeast genome. Firstly, we required that a cluster had to contain at least 50% uniquely mappable bases. For every potential novel transcript identified by our microarray data in a particular mutant, we employed the following criteria to Solexa data originating from the same mutant to validate the transcript: (A) the transcript detectable above background level, (B) the transcript differentially expressed between the mutant and the wild-type, and (C) the transcript differentially expressed when compared to its surrounding regions (see Materials and Methods for detailed explanation of precise criteria and cutoffs used for determination of validity).

In addition, we analyzed our data for the presence of reads containing a putative poly A+ tail, which would allow us to infer both the strand of origin as well as a precise 3′ boundary, however, very few such reads were present in our dataset most likely due to our use of random priming as opposed to oligo dT priming.

**Figure 4:**
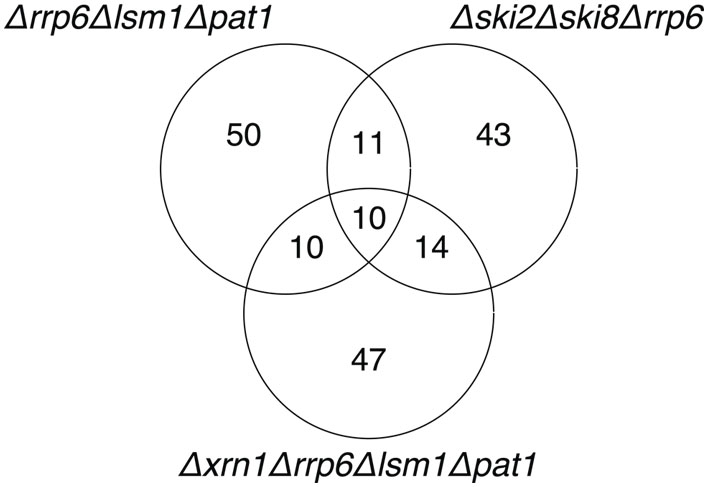
Venn diagram showing the distribution of novel segments between the different mutants within which they were discovered.

Following validation of individual clusters, we determined which clusters were common across the different mutants and as well as our poly A+ and total RNA hybridizations. 240 of our validated clusters were found in data from only one microarray, 79 were found in 2, 26 in 3, and 20 were found in 4 or more of the six microarrays, resulting in 365 validated transcripts (see Table S1), identified by virtue of differential transcript abundance between one or more mutants and the wild-type strain. Of these, 204 were found exclusively in the poly A+ RNA, 86 were found exclusively in the total RNA fraction, and 75 were detected in both. Several of these overlap with novel transcripts identified in recent studies: 67 with David et al. [25], 116 with Miura et al. [26], 46 with Nagalakshmi et al. [37], and 43 with Davis et al [63]. Beyond these, our 365 validated transcripts includes 185 additional previously undescribed transcripts, which we were able to discover by down-regulating RNA degradation. The majority of these novel transcripts (140 of 185) were found and validated in a single mutant only, with only 45 of them being identified and validated on two or more mutants (Figure 4).

### Characterization of Validated Transcripts

**Figure 5:**
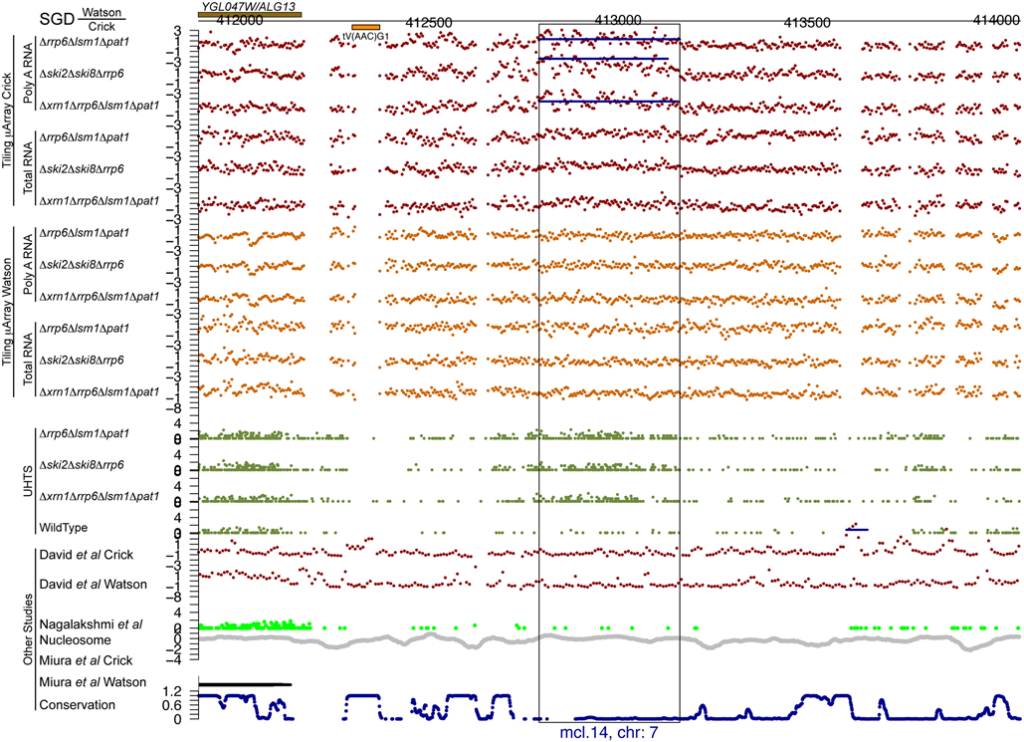
An unannotated transcript found in this study. There are the following information tracks from top to bottom: SGD annotation on the Watson and Crick strands, our tiling microarray data from the Crick and Watson strands (poly A+ RNA above total RNA), our UHTS data for the mutant and wild-type strains, tiling microarray data from David et al. for the Crick and Watson strands, UHTS data from Nagalakshmi et al., nucleosome position, data from Miura et al., and degree of conservation. The name and chromosome of origin of each transcript are indicated below. For the UHTS data, each point plotted corresponds to the 5′ end of sequence reads, and the position of the plotted point above the axis indicates (on a log scale) how many reads mapped to that position. Horizontal lines in a track indicate novel segments found in the corresponding study (black for forward strand and blue for reverse strand).

For each of the potential novel transcripts, their immediate surrounding regions were plotted (e.g. see Figures 5 through [Fig pgen-1000299-g006] [Fig pgen-1000299-g007] [Fig pgen-1000299-g008] [Fig pgen-1000299-g009] 10 and Figures S5 and S6) along with a track of the current annotation from SGD [18], and data from David et al. [25], Miura et al. [26], and Nagalakshmi et al. [37]. Additional tracks representing nucleosome positioning [67] and the degree of conservation between *Saccharomyces cerevisiae* and other closely related yeast species [68] were also plotted. In addition, the transcript's chromosome and its strand of origin are shown at the bottom of each plot. Six examples of transcripts unannotated in SGD and identified in this study can be seen in Figures 5 through [Fig pgen-1000299-g006] [Fig pgen-1000299-g007] [Fig pgen-1000299-g008] [Fig pgen-1000299-g009] 10, all of which are located in regions currently described as intergenic. Plots for all 365 currently unannotated transcripts identified in this study can be found in Figures S5 and S6.

**Figure 6 pgen-1000299-g006:**
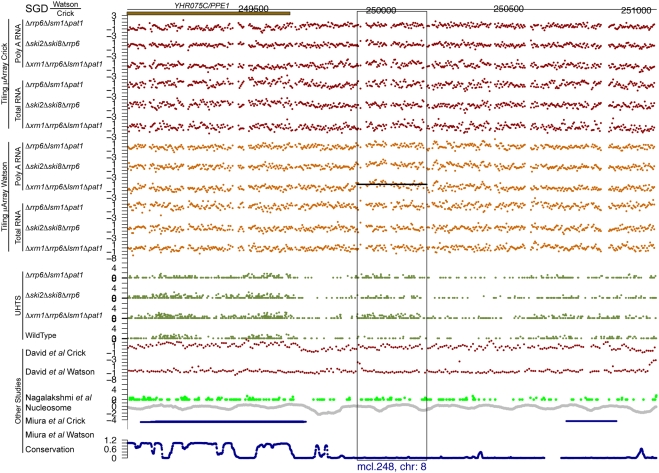
An unannotated transcript found in this study. See the legend for Figure 5 for details.

**Figure 7 pgen-1000299-g007:**
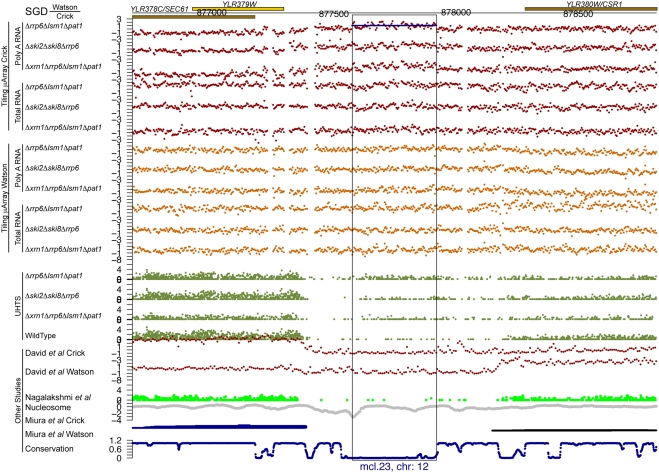
An unannotated transcript found in this study. See the legend for Figure 5 for details.

**Figure 8 pgen-1000299-g008:**
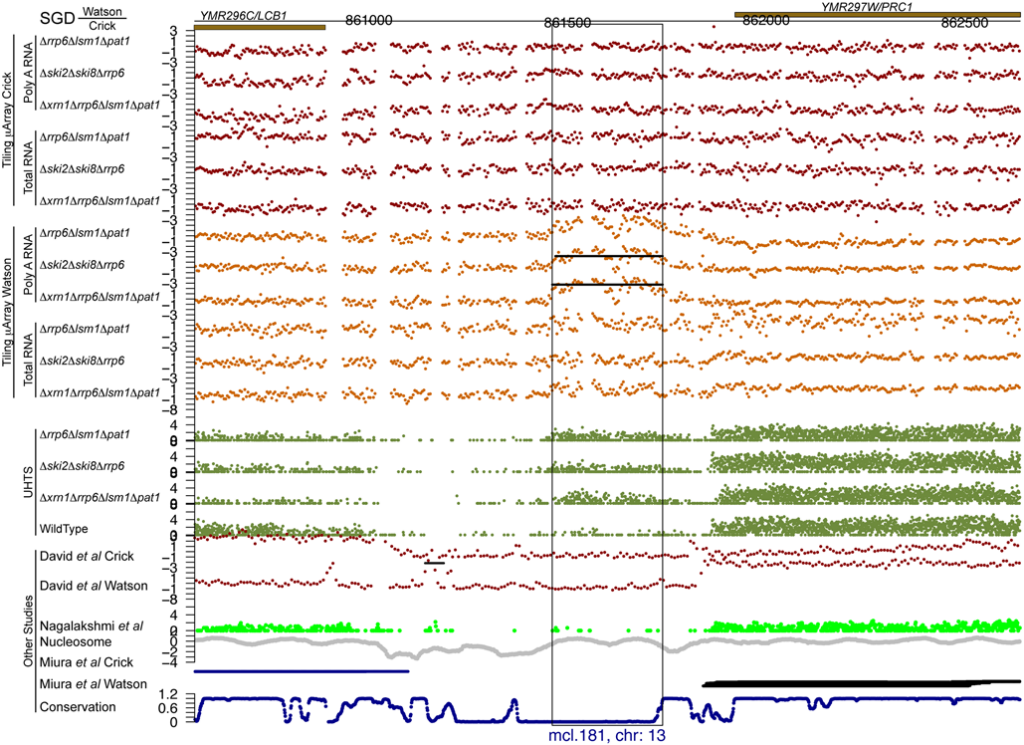
An unannotated transcript found in this study. See the legend for Figure 5 for details.

**Figure 9 pgen-1000299-g009:**
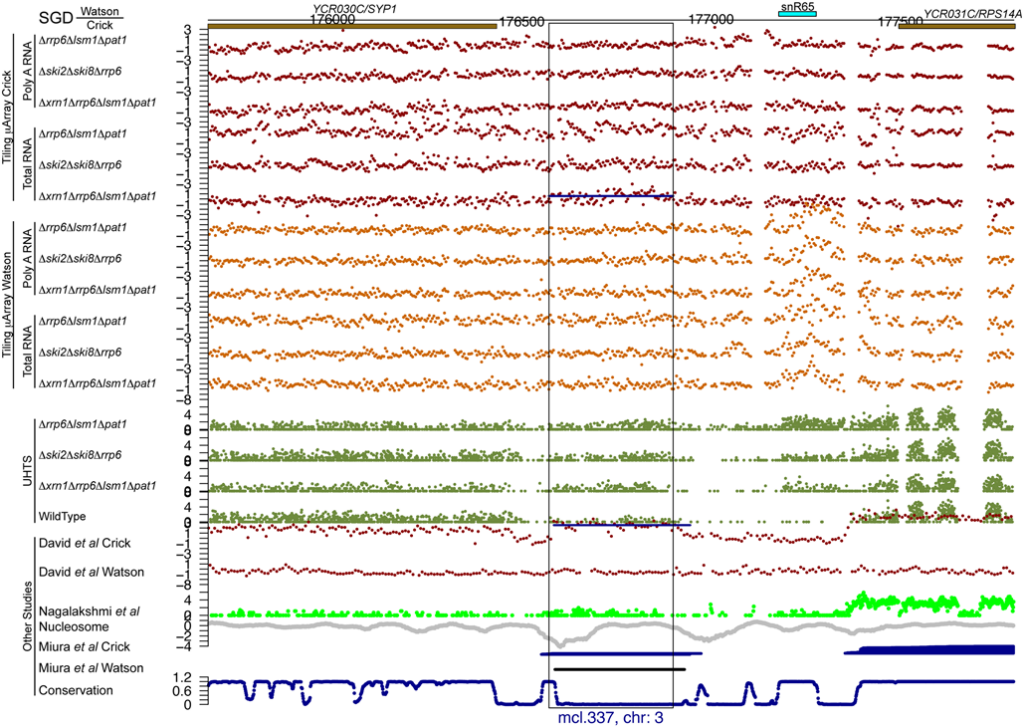
An unannotated transcript found in this study, also found in other studies. See the legend for Figure 5 for details.

**Figure 10:**
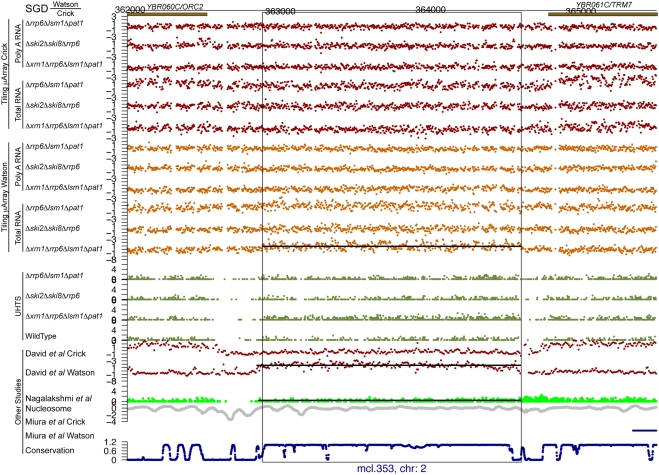
An unannotated transcript found in this study, also found in other studies. This one is in a region of high conservation. See the legend for Figure 5 for details.

**Figure 11:**
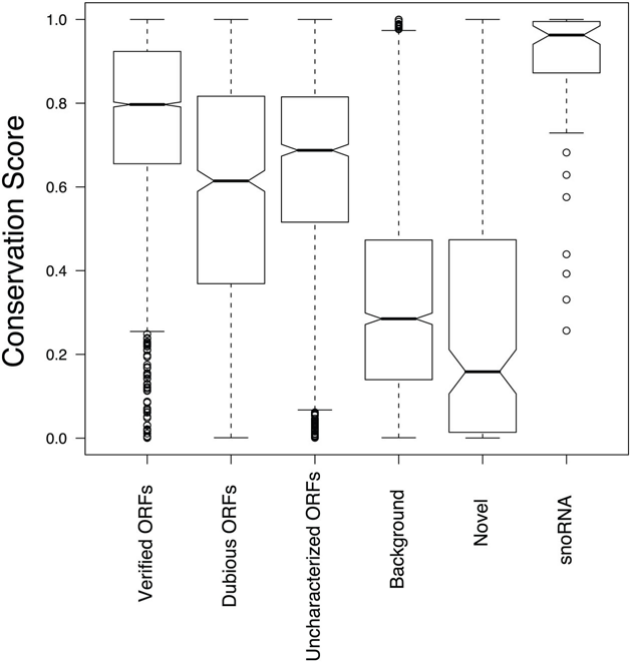
Box plots illustrating the conservation scores [68] of the various types of transcripts across closely related yeast species. The boxplot depicts the distribution of the conservation scores, with the box surrounding the 25% and 75% quantiles. The center of the notch corresponds to the median. If two notches do not overlap, it is evidence for the medians being different. Novel refers to novel transcripts found in this study, background regions are defined in materials and methods, while other classes are the same as defined in the Figure 2.

Of the 185 transcripts novel to this study, more than 80% have an average conservation score lower than 85% of the Verified ORFs (see Figure 11, as well as Figures 5 through [Fig pgen-1000299-g006] [Fig pgen-1000299-g007] [Fig pgen-1000299-g008] 9 for five such examples; see also Figure S7). This implies that the vast majority of these transcripts could not have been found using comparative genomics.

Figures 5 through [Fig pgen-1000299-g006] [Fig pgen-1000299-g007] 8 show four novel transcripts unique to this study that are all located in regions of the genome that show poor conservation across different *Saccharomyces* species, as indicated by the conservation track at the bottom of each plot. Both our tiling microarray data and our UHTS data clearly show that the transcripts in Figures 5 through [Fig pgen-1000299-g006] [Fig pgen-1000299-g007] 8 are only seen in the one or more of the mutant strains and not in the wild-type, which was the criterion that enabled us to identify them. Prior transcript discovery studies, however, were only able to identify transcripts that are present in the wild-type, and in Figures 5 through [Fig pgen-1000299-g006] [Fig pgen-1000299-g007] 8, there are no data from David et al., Miura et al., or Nagalakshmi et al. to suggest that they could detect these novel transcripts. In some cases the nucleosome track is suggestive of transcriptional potential, due to there being low occupancy immediately upstream of the potential transcript. In Figures 5 through [Fig pgen-1000299-g006] [Fig pgen-1000299-g007] 8 there is a nucleosome dip immediately upstream of the identified segment, which is frequently observed in connection with transcribed regions [67].

Figures 9 and 10 illustrate two examples of intergenic transcripts found in this study that have been found in at least one other study (we considered a transcript to be one found by another study if there was a 25% overlap between the transcripts on the same strand); one of these falls in a conserved region (Figure 10), while the other does not (Figure 9). Additionally, in both examples, it is clear in our UHTS data that these transcripts were present in the wild-type strain, though at lower levels than within our mutants, indicating that they could readily be detected in the other studies, as they indeed have been. Figure 9 shows a transcript on the Crick strand that is upstream of a verified ORF and is seen in all three of the other studies (though Nagalakshmi et al. do not call it). There is a large region of low nucleosome occupancy just upstream of it, suggesting that the region is indeed transcribed, and the transcript itself overlaps with the nucleosome dip of the downstream ORF, suggesting that this new transcript may play a role in the transcriptional regulation of the ORF downstream of it. Figure 10 shows a relatively long transcript (1,721 bp) on the Watson strand that is also seen in David et al. and Nagalakshmi et al. It is highly conserved and the presence of a nucleosome dip upstream suggests that this region is transcribed.

We analyzed all of our novel transcripts for potential open reading frames, to determine if any were likely to be protein-coding. In each case, the longest open reading frame was translated and blasted against the non-redundant protein dataset (nr) from GenBank. The shortest novel transcript identified was 47 nucleotides long (intergenic), while the longest was 1,869 nucleotides in length (also intergenic), though the longest ORF that it contains only has the potential to encode a peptide 80 amino acids in length. The longest ORF that we discovered within all of our novel transcripts was within an ∼438 bp transcript on the Watson strand of chromosome 7 (coordinates 23,339–23,777), with the potential to encode an 87 amino acid polypeptide. However, this potential peptide showed no significant similarity when BLASTed against the GenBank non-redundant protein dataset. The remaining longest ORFs within each novel transcript were all shorter, with no significant similarities to any known proteins. It is not clear whether this means they do not encode proteins, or whether they encode novel, short proteins, which are currently uncharacterized due to their low conservation. We also analyzed each of our novel transcripts for any matches to known RNA structures present in the RFAM database [69],[70], but none of the sequences showed matches to any RFAM entries.

### Validation of Transcripts Identified in Other Studies

Using our detected above background statistic, we sought to determine the percentage of recently published novel transcripts present in our sequencing data. It should be noted that non-detection based on our data does not imply non-existence of these transcripts due to the differing experimental conditions as well as the distinct assays. Using our wild-type data, we detected 18.1% of the 487 Nagalakshmi et al. transcripts, 43.7% of the 784 David et al. transcripts, and 16.3% of the 667 Miura et al. transcripts. Using our Δ*rrp6*Δ*lsm1*Δ*pat1* data, we detected 65.3% of the 176 Davis and Ares transcripts (see Table S2 and Table S2 for a discussion of which transcripts were used from each study).

## Discussion

In this study, we have clearly demonstrated that there is still much we do not know about the transcriptome of *S. cerevisiae*, despite its deserved reputation as the most well-characterized eukaryote. Unbiased genome-wide studies of the budding yeast transcriptome [25],[26],[37] have yielded a remarkable amount of information, regarding new transcripts, new introns, the presence and location of antisense transcripts, and corrections to the current annotation. As described here, we have utilized tiling microarrays in conjunction with “next-generation” technologies to sequence cDNA libraries, with which we generated more than 50 million uniquely mappable reads from a wild-type and four mutant strains. Using these data, we have identified and validated 365 transcripts, the majority of which are more abundant in one or more of the RNA turnover mutants than in the wild-type strain (with a minority being less abundant), all of which are currently unannotated in SGD. The functions of these new RNAs remain unknown, though it is possible that many of the newly discovered transcripts correspond to CUTs, which normally would have been targeted for degradation by the TRAMP complex, but have been stabilized in the mutant background. Others may correspond to novel functional transcripts. These novel transcripts do not contain long ORFs capable of encoding proteins with recognizable similarity to known proteins; it is not clear whether this means they do not encode proteins or whether they code for hitherto unknown proteins with no known homologs. They also do not contain any recognizable RNA structures found in the RFAM database.

While our work described here has much in common with the work described in David et al. and Nagalakshmi et al., our use of RNA turnover mutants resulted in the finding of an additional 185 novel transcripts that may have otherwise remained undiscovered. Miura et al.'s use of vector-capped cDNA clone libraries is powerful in that it has a single nucleotide resolution, as opposed to our tiling microarray resolution of 4 nucleotides, allowing these authors to map transcriptional start sites to the exact nucleotide, in a high throughput manner. The use of overlapping, but non-identical, techniques among all these studies (including this one) has resulted in an ever more detailed knowledge of the yeast transcriptome.

In our approach, we utilized a high-throughput discovery and validation pipeline. Clearly, much work needs to be done to characterize and understand the transcripts discovered here as well as those discovered in previous studies, however a first step in characterizing the transcripts is localization and then validation. In our computational analysis we employed a strategy of being lenient in identification of putative novel transcripts (differentially expressed at 0.5 on the log2 scale). This was followed by a strict validation step (at our thresholds, on average 75% of annotated Verified ORFs were detected in our 3 mutant experiments as described by the ROC-like curves in Figure 3). Many (∼55%) of the clusters found in the microarray analysis were not validated by these stringent thresholds. These tended to be shorter, be less differentially expressed and included many clusters that were less abundant in the mutants as compared to wild-type. By using distinct assays with rigorous criteria for transcript validation, we have elucidated more of the regions of the yeast genome that are transcribed.

In our attempt to find low abundance and transient transcripts by restricting our search to transcripts that were present in differential relative abundance in our RNA processing mutants, we may have missed transcripts that are present in the mutant and the wild-type at the same abundance. This was a caveat we had to consider in the pursuit of transcripts that we believed would otherwise be difficult to detect, and the discovery of 185 novel transcripts despite the work of other comprehensive genome-wide transcriptome studies shows that our strategy was a fruitful one. By utilizing the strand-specific tiling array were able to localize transcripts to their strand of origin, something that was not possible (without introducing a 3′ bias to the data by priming the labeling reaction with oligo-dT) with the current protocols for RNA-Seq using the Solexa 1G Genome Analyzer. It is likely that modified protocols will soon address this shortcoming, and indeed such protocols for the ABI SOLiD sequencing system have been recently published [71].

We can now ask the important and obvious question: has the yeast transcriptome been completely described, and what does completion mean? It is possible that if we sequence deeply enough, we may observe that every nucleotide within the genome is transcribed at some level (see Figure 2), though clearly this is not a strict enough criterion to allow us to identify a transcribed segment. The genome-wide studies that have set out to discover new transcripts in yeast in an unbiased fashion have so far used a limited set of experimental conditions. Thus, it seems likely that deep sequencing of RNA from dozens of possible conditions (which must be carefully chosen to span as much of the “expression space” as possible) will yield yet more new transcripts, or show new variations in existing ones. It will be of particular interest to profile all of these novel transcripts under a variety of conditions to see how they are regulated and co-regulated, as well as to determine whether they encode proteins or functional RNAs, and whether their absence results in a detectable phenotype.

Since many of the recently discovered transcripts (including those in this study) have been found in regions of the genome where there is little or no sequence conservation (though the conservation scores from Siepel et al. [68] do not indicate whether these regions are evolving neutrally, or under positive selection), it will be informative to profile different and diverse strains of *S. cerevisiae* to determine if these transcripts are ubiquitous within the species, and to determine whether the syntenic (but non-conserved) regions within closely related species within the *Saccharomyces sensu stricto* are also transcribed. With such data, we can hope to discover and hopefully appreciate not only how each of these species are related to one another, but also how their transcriptional potential and networks have diverged.

Since the landmark publication of the *S. cerevisiae* genome sequence 12 years ago, more than 25,000 research publications on yeast have appeared, yet we are still adding to our knowledge of the transcriptome of *S. cerevisiae*. While arguably the most well-understood eukaryote, we still do not have a complete understanding of such a fundamental concept as “what and where are all of its genes.” New technologies such as high resolution tiling microarrays and ultra high-throughput sequencing are opening up new avenues of research, and it is clear that the quantity of data that these technologies allow us to generate will only increase. This study (and others like it) underscores how much work remains to be done in understanding and cataloging the transcriptomes of even the most well-studied model organisms.

## Materials and Methods

### Strains

All deletions were created in a diploid *Saccharomyces cerevisiae* strain which was created by crossing strains FY23 and FY86 [72], which are isogenic to the sequenced strain S288C and carry the auxotrophic markers: his3-Δ200, leu2-Δ1, trp1-Δ63, and ura3-52. All deletions were created using the Geneticin antibiotic resistance marker, utilizing the system described in [73]. Specifically, primers specific to regions to be deleted by homologous recombination were designed to utilize the plasmid pFA6-kanMX6 as a PCR template in order to replace the regions of interest with the gene encoding for resistance against the antibiotic Geneticin.

PCR was performed (see Table 2 for primers), generating approximately 1.5 kb DNA fragments in agreement with the size of the Geneticin resistance gene, which were then transformed using standard lithium acetate transformation techniques into the diploid cells grown in YPD at 30°C at mid-log phase. Cells were selected on YPD agar plates with 300 µg/ml working concentration of Geneticin. Deletions were confirmed by PCR (see Table 2 for primers) and the diploids were sporulated and their tetrads dissected to generate haploid segregants carrying the deletions of interest.

**Table 2:** Primers used for creation and confirmation of deletion mutations.

**Primers used to create deletions:**	
*RRP6* Forward	5′GAGGGCATCGGAAAATTTTTCAGTAATGAATATTAATGTTCATCTGAAGACGGATCCCCGGGTTAATTAA3′
*RRP6* Reverse	5′ATAACTCCATGACACAGATATTCGATTAGATGAATTTAGAGGTCTTAAATGAATTCGAGCTCGTTTAAAC3′
*XRN1* Forward	5′CAATAAGCAATTGACTAATCCTAGGACGATTCGTGTACTATAAGGAGAAACGGATCCCCGGGTTAATTAA3′
*XRN1* Reverse	5′TTCTTAACAAGATCAACGATTAAATACAAATACCCCTCTTTATATAGGTCGAATTCGAGCTCGTTTAAAC3′
*SKI2* Forward	5′AATTTAAAAGTCAACGCAGAAACTATAATACATTGCCACATAGTTCTTTCCGGATCCCCGGGTTAATTAA3′
*SKI2* Reverse	5′TAAAAACTATGTATACGTGTGTGTGTGTGTGTGCAATAAGAGTTCGAAAAGAATTCGAGCTCGTTTAAAC3′
*SKI8* Forward	5′ATAAAGTAAAGAAGGAAAAATTAGGCGATATTAAAACAAATCTAAAATAACGGATCCCCGGGTTAATTAA3′
*SKI8* Reverse	5′TATTAAATATTACTGAAATTTTATGAACCAAAAAGAATAATGGATGATGTGAATTCGAGCTCGTTTAAAC3′
*PAT1* Forward	5′GAAAGAAACAAGGTGAATGAAAAGAAACATGTACACCTTGAAGGAAGCAACGGATCCCCGGGTTAATTAA3′
*PAT1* Reverse	5′CATATACAATAAATGATCTACAAAGGGTAGGAAATAAAAAATAAGGGAGAGAATTCGAGCTCGTTTAAAC3′
*LSM1* Forward	5′AACAGGATTGCCAACGCTGCAGTAGAGTTATACCAACATTTGCTCCGCTTCGGATCCCCGGGTTAATTAA3′
*LSM1* Reverse	5′TTGATTAAGTGTACGGATAGGTAAAACTGAATGTGGAAATTTTTGAGAGTGAATTCGAGCTCGTTTAAAC3′
**Primers used to confirm deletions:**	
*RRP6* Forward	5′ATGCAAAATAAGTTCACGTG3′
*RRP6* Reverse	5′GGAGATGAAGGGAAACACAG3′
*XRN1* Forward	5′AAGGATACTGTCTTCTTCCG3′
*XRN1* Reverse	5′GCTTTGTGTAAAAAATACCC3′
*SKI2* Forward	5′TCAGAACGCCCATCGGATGG3′
*SKI2* Reverse	5′TACAATAGTCCGCCCGTTGC3′
*SKI8* Forward	5′AATTGATACAAATCTTTAGG3′
*SKI8* Reverse	5′AGTGAAATTCATACATTGGC3′
*PAT1* Forward	5′TACTATTGTTATCACTTCCC3′
*PAT1* Reverse	5′TATGGTGGTATTATTGATGC3′
*LSM1* Forward	5′TCAGCACCTGTATTTCAATC3′
*LSM1* Reverse	5′CTGCGCAAATACGTTACTTC3′

Different deletions strains were mated to generate diploids, which were then sporulated and tetrads were dissected. Because only the Geneticin marker was used to generate these deletions, PCR analysis was used to confirm all newly generated double mutant strains. The process was repeated to generate the triple and quadruple mutants (see Table 1 for resulting strains used in this study). Some deletion combinations could not be generated, suggesting they are synthetically lethal, and thus were not used in this study. For instance, Δ*xrn1* and Δ*ski8* are synthetically lethal, as any attempt to combine strains with these deletions was unsuccessful. Haploid strains exhibiting phenotypes suggesting the accumulation of suppressor mutations were not used for further study. Originally the decapping factor *DHH1* and the Ski complex component *SKI3* were selected to be included, but strains carrying either Δ*dhh1* or Δ*ski3* showed a propensity to accumulate suppressor mutations when combined with other deletions from this study and thus were dropped from the analysis. The Affymetrix tiling array data as well as the sequencing data confirmed that there was no expression signal corresponding to the genetic loci of the deleted genes.

### NaCl Exposure

Our unpublished studies suggested that among two dozen or so different conditions that we have assayed, exposure to high salt (0.8 M NaCl) results in the expression of the greatest fraction of known and novel transcripts, and thus was chosen as the experimental condition to use to find previously unannotated and low abundance transcripts. Cells were grown at 30°C in YPD to approximately 1×10^7^ cells/ml as determined by a Beckman Coulter Z2 Particle Count and Size Analyzer. 1.6 M NaCl (in YPD) was added in an equal volume of YPD prewarmed to 30°C (final concentration 0.8 M). Cells were harvested after 30 minutes by filtration, frozen in liquid nitrogen, and kept at −80°C until RNA extraction and purification.

### RNA Extraction and Purification

RNA was extracted from the cells using a slightly modified version of the traditional hot phenol protocol [74] followed by ethanol precipitation and washing. Briefly, 5 ml of lysis buffer (10 mM EDTA pH 8.0, 0.5% SDS, 10 mM Tris-HCl pH 7.5) and 5 ml of acid phenol were added to frozen cells and incubated at 60°C for 1 hour with occasional vortexing, then placed on ice. The aqueous phase was extracted after centrifuging and additional phenol extraction steps were performed as needed, followed by a chloroform extraction. Total RNA was precipitated from the final aqueous solution with 10% volume 3 M sodium acetate pH 5.2, and ethanol, and resuspended in nuclease-free water.

### RNA Preparation for Use on Affymetrix Tiling Microarrays

All microarray analyses were carried out using Affymetrix GeneChip *S. cerevisiae* Tiling 1.0R Array (Reverse) (part number: 900645) for Watson strand expression or GeneChip *S. cerevisiae* Tiling 1.0F Array (Forward) (part number: 520286) for Crick strand expression.

The arrays each contain more than 2.5 million perfect match probes, which are offset from one another by 4 bases across the genome (21 bp overlap). Thus, each residue in the genome is interrogated on average by 6 oligonucleotide probes.

Total RNA samples were prepared following the protocol exactly as described in David et al. [25].

PolyA RNA samples were prepared as follows. 500 µg of total RNA were PolyA purified using Qiagen Oligotex suspension to produce approximately 10 µg of PolyA RNA as determined by OD_260/280_. 2 µg of the PolyA purified RNA were then used in the generation of cDNA as per Affymetrix First Strand and Second Strand Synthesis protocols utilizing a T7-Oligo(dT) as the primer for the First Strand, followed by *in vitro* transcription to generate biotin labeled cRNA, as outlined by Affymetrix protocols. The cRNA was fragmented as described by Affymetrix, and then sent for hybridization and scanning by the PAN facility at Stanford (http://cmgm.stanford.edu/pan/) according to standard Affymetrix protocols.

### Discovery of Novel Transcripts Using Tiling Microarrays

Our goal was to identify short-lived transcripts based on measured intensities of probes tiling the genome. It is well known that probe affinities significantly bias the relationship between measured intensity and actual transcript abundance. In David et al. this was addressed by effectively forming log ratios between wild-type and genomic DNA hybridization. In order to highlight the changes between mutants and wild-type transcription and to reduce the effect of probe affinities we formed log ratios between mutant and wild-type intensities. This approach has the same effect on probe affinities as the approach used by David et al., see Figures S1 and S2.

### Mapping and Pre-Processing

The probes on the tiling array were mapped to the yeast genome, as downloaded from the *Saccharomyces* Genome Database on May 19^th^ 2008, using MUMmer [75]. Only perfect match (PM) probes mapping to a unique region were retained for further analysis. For each mutant RNA hybridization, log ratios of mutant PM intensities to wild-type PM intensities were calculated.

### Segmentation

The resulting data were segmented using the ‘segment’ function in the R package ‘tilingArray’ [64] from Bioconductor release 2.1, which performs a simple change-point analysis. The log ratios of mutant compared to wild-type for total RNA and poly A+ purified mRNA extractions for each mutant and chromosome strand were segmented separately. An open question in any segmentation analysis is the selection of the number of segments. We followed Huber et al. (2006) in using the Bayesian information criterion (BIC) penalized log-likelihood, noting that this tends to overestimate the number of segments (see below).

### Post-Processing of Segments

Following the segmentation we were left with a set of segments for each of the three mutants and two RNA sample types (total RNA or poly A purified RNA). Our analyses indicated that transcripts are often split into a number of segments due to various artifacts of the array data (outliers, incomplete probe-affinity correction, cross-hybridization). At this stage, we wished to both join appropriate segments into adjacent co-expressed segments (clusters) as well as filter out *a priori* uninteresting clusters. The pipeline for constructing clusters from segments and producing a set of putative clusters to be validated using the sequencing data worked as follows:

1. Label each segment as upregulated (med(seg) − med(microarray) ≥.5), downregulated (med(seg) − med(microarray) ≤−.5), or baseline ( −.5<med(seg) − med(microarray) <.5). Here med(.) is the median of log_2_(mutant/wild-type) for either the segment or the entire microarray.
2. Drop any baseline segment containing 5 probes or less. This step attempts to avoid the creation of separate segments due to non-responsive probes.
3. Join adjacent segments if they have the same regulation label (i.e., up, down, or baseline), unless the following criteria hold: the absolute difference in medians between the two segments exceeds 1 and the lengths of the two segments are greater than 30 probes and span more than 150 bp. Uneven spacing in the tiling array probes occurs due to repeat regions often leaving an area of the chromosome tiled at a lower density. In order to keep the cutoffs consistent through these areas we employ the strategy of enforcing a minimum length at the base and probe level. These joined segments were then referred to as clusters.
4. Drop all baseline clusters as well as any cluster with fewer than 5 probes or a length less than 40 bp.
5. Remove any cluster that overlaps any known transcribed annotation on the same strand. We extend each annotated element by 100 bp on both the 5′ and 3′ end to account for UTRs.
6. For any cluster that overlaps annotation on the opposite strand we further required a log_2_ fold change of at least 2 as well as a log_2_ fold change of less than 1 on the strand opposite the cluster.

This process resulted in a set of putative clusters that were subsequently considered for validation by Solexa sequencing.

### Kits and Reagents Used in the Ultra High–Throughput Sequencing (UHTS) RNAseq Library Construction for the Solexa Platform

In order to generate libraries for the Solexa platform, various reagents and kits were required. At the time that these experiments were performed, Illumina did not have an RNA-Seq specific kit, and thus parts of various kits were utilized. Note that not all of the reagents from the kits provided by Illumina were used, as these kits were adapted for use in the protocol below and not necessarily used as described in the instructions that came with the kit. They are as follows:

For protocols desiring PolyA purified RNAs:

Illumina Digital Gene Expression-Tag Profiling for *Nla*III Sample Prep Kit (part number 1002390)
Illumina Genomic DNA Sample Prep Kit (part number 1000181)
Invitrogen SuperScript III Reverse Transcriptase (part number 18080-044)
Invitrogen Random (N6) Primers (part number 48190-011)
Qiagen MinElute Reaction Cleanup Kit (part number 28204)
Qiagen QIAquick PCR Purification Kit (part number 28104)
Zymo Research Zymoclean Gel DNA Recovery Kit (part number D4001)
Amersham Biosciences MicroSpin G-50 Columns (part number 27-5330-01)
Millipore Microcon Ultracel YM-30 Centrifugal Filter Devices (part number 42410)

Also required was a magnetic stand that can accommodate 1.5 ml microcentrifuge tubes. The protocol as described below was done using DNase/RNase certified free siliconized 1.5 ml microcentrifuge tubes.

### UHTS PolyA RNA Preparation

Strains used for our UHTS experiments are GSY147 and GSY1289 (see Table 1). GSY147 was derived from DBY10146 (a gift from David Botstein) (which itself was derived from an FY background [72]) which was backcrossed by Katja Schwartz to FY2 and FY3 [72] to generate a wild-type S288C strain that had no auxotrophies or mutations.

### Double PolyA mRNA Preparation

Two consecutive purifications using oligo dT conjugated magnetic beads were performed as follows. 100 µg of Total RNA were diluted in a final volume of 100 µl water and heated at 65°C for two minutes and then placed on ice. 200 µl of beads were equilibrated by two consecutive 100 µl washes in binding buffer (mixed gently by hand), using a magnetic stand to separate the beads from the buffer. The beads were then resuspended in 100 µl of binding buffer. The RNA was added to the beads, and the tube was mixed gently by hand for 5 minutes at room temperature and then placed on the magnetic stand to separate the beads from the supernatant. The supernatant was discarded, and the beads underwent two consecutive washes with 200 µl washing buffer. The beads were resuspended in 10 mM Tris-HCl pH 7.5, and the tube was heated at 80°C for two minutes and then immediately placed on the magnetic stand where the supernatant was transferred to a new tube. The beads were saved and prepared for the second round of PolyA purification by washing them once with 200 µl washing buffer. The entire process was then repeated once for a second round of purification, beginning with the dilution of the RNA and the denaturing of the RNA secondary structure.

### UHTS RNA Fragmentation

PolyA purified treated RNA samples were then fragmented to ensure an unbiased binding of the random hexamers during cDNA synthesis. 5× Fragmentation Buffer (200 mM Tris Acetate pH 8.2, 500 mM Potassium Acetate, 150 mM Magnesium Acetate) was made, of which 5 µl was added to the RNA sample, and the total reaction was brought up to 25 µl. The sample was heated at 94°C for 2.5 minutes and immediately placed on ice. The sample was then run through a G-50 spin column that has been equilibrated with 3×400 µl of nuclease free water to remove ions from the fragmentation. The sample was concentrated to 10.5 µl with a Micron filter.

### UHTS cDNA Synthesis

First Strand Synthesis:

10.5 µl of fragmented RNAs were transferred to a PCR tube and 1 µl of random hexamer (3 µg/µl) was added. The tube was heated to 65°C for 5 minutes and then placed on ice. The following reagents from the Illumina kit were then added: 4 µl 5×1^st^ strand buffer, 2 µl 100 mM DTT, 1 µl 10 mM dNTP, and 0.5 µl RNaseOUT (40 U/µl). The tube was mixed and left at room temperature for 2 minutes. 1 µl SuperScript III (200 U/µl) was added, and the sample was placed in a thermocycler with the following program: 25°C for 10 minutes, 42°C for 50 minutes, 70°C for 15 minutes, 4°C hold.

Second Strand Synthesis:

The first strand synthesis reaction was transferred to a 1.5 ml siliconized microcentrifuge tube and placed on ice. 61 µl nuclease free water was added to the sample, along with the following reagents from the Illumina kit: 10 µl 2^nd^ strand buffer, 3 µl 10 mM dNTPs, 1 µl RNase H (2 U/µl), and 5 µl DNA Pol I (10 U/µl). The sample was vortexed and placed in an Eppendorf Thermomixer R (set at 16°C and programmed to spin at 1400 rpm for 15 seconds and stand for 2 minutes) overnight (minimum 2.5 hours).

The newly synthesized cDNA was purified with a QIAquick PCR spin column as per Qiagen protocols and eluted in 30 µl EB solution.

### UHTS cDNA Repair

The following reagents from the Illumina kit were added to the 30 µl sample as follows: 45 µl nuclease free water, 10 µl T4 DNA ligase buffer with 10 mM ATP, 4 µl 10 mM dNTPs, 5 µl T4 DNA polymerase (3 U/µl), 1 µl Klenow DNA polymerase (5 U/µl), 5 µl T4 PNK (10 U/µl). The sample was vortexed and incubated at 20°C for 30 minutes. Afterwards, the sample was purified with a QIAquick PCR spin column as per Qiagen protocols and eluted in 32 µl EB solution.

### UHTS cDNA Preparation for Adaptor Ligation by the Addition of an A Base

The following reagents from the Illumina kit were added to the 32 µl sample as follows: 5 µl Klenow buffer, 10 µl 1 mM dATP, and 1 µl Klenow 3′ to 5′ exonuclease (5 U/µl). The sample was vortexed and incubated at 37°C for 30 minutes. Afterwards, the sample was purified with a MinElute spin column as per Qiagen protocols and eluted in 10 µl EB solution.

### UHTS Adaptor Ligation

The following reagents from the Illumina kit were added to the 10 µl sample as follows: 25 µl DNA ligase buffer, 2 µl adaptor oligo mix, and 5 µl DNA ligase (1 U/µl). The sample was vortexed and incubated at 25°C for 15 minutes. Afterwards, the sample was purified with a MinElute spin column as per Qiagen protocols and eluted in 10 µl EB solution.

### UHTS cDNA Size Selection and Gel Purification

The 10 µl sample was loaded onto a 1% TAE agarose gel at least one lane away from a 100 bp ladder. The sample was run sufficiently far enough and a gel slice corresponding to approximately 200 bp+/−50 bp was excised out of the gel with a scalpel (note that no cDNA may be visible on the gel). The cDNA was purified using a Zymo Research Zymoclean Gel DNA Recovery Kit and eluted in 10 µl nuclease free water.

### UHTS cDNA Amplification and Sequencing

The 10 µl sample was transferred to a PCR tube. The following reagents from the Illumina kit were added to the 10 µl sample as follows: 27 µl nuclease free water, 10 µl 5× cloned Phu buffer, 1 µl oligo 1.1, 1 µl oligo 2.1, 0.5 µl 25 mM dNTPs, 0.5 µl Phu polymerase. The sample was then run on a thermocycler using the following program: 98°C hold for 30 seconds, 98°C for 10 seconds, 65°C for 30 seconds, 72°C for 30 seconds, 72°C hold for 5 minutes, 4°C hold, for 50 cycles. The sample was purified with a QIAquick PCR spin column as per Qiagen protocols and eluted in 30 µl EB solution. The sample was then run through a G-50 spin column that had been equilibrated with 3×400 µl of nuclease free water to remove any remaining unincorporated nucleotides that would interfere with the concentration determination of the library. The DNA was concentrated through the use of a Speed Vac until the final volume of the library was 10 µl. The cDNA was quantified using a Nanodrop. A concentration range between 10–100 ng/ml final concentration of an RNAseq library is required for good quality sequencing. The sample was then sent for sequencing in the Genetics Department Solexa machine at Stanford.

### Mapping of Solexa Reads to the Yeast Genome

Sequence reads that passed Solexa's quality filters were aligned to both the yeast genome and the spliced yeast ORF set (allowing up to 2 mismatches), downloaded from the *Saccharomyces* Genome Database (SGD) [76] on May 19^th^, 2008, using ELAND, which is part of the Solexa analysis pipeline [77] (we used version 0.3.0). Only reads mapping uniquely to the genome were retained.

### Comparison and Combining of Sequence Data across Flow Cell Lanes

We examined the goodness of fit for a simple Poisson model described below, using the chi-square goodness of fit statistic (see [78]). QQ-plots of the observed statistic for each known gene against the theoretical distribution are shown in Figure S2 and show a remarkably good fit. Based on this model, we aggregated data for each strain across the multiple lanes on the Solexa flow cell.

### Validation of Putative Novel Transcripts Using Solexa Sequencing

In order to validate each putative transcript identified by tiling array data analysis, we investigated the following three criteria:

- the transcript is expressed above a suitably defined background level;
- the transcript is differentially expressed in the mutant as compared to the wild-type;
- the transcript is differentially expressed as compared to the surrounding region;

An important consideration in all subsequent analyses was that certain areas of the genome are unmappable due to repeated sequences. We defined a base as non-unique if the 25mer starting at that position occurs elsewhere in the genome. We excluded all such bases from consideration in subsequent analyses.

#### A. Above background expression

First, we determined whether the transcript was above background level. Background regions were defined in the following fashion:

1. All regions that are intergenic on both strands were obtained.
2. Any region which overlaps the segments reported by Nagalakshmi et al., David et al., Miura et al., or Davis and Ares was removed (See Table S2 for tables of genes used from other studies). The clusters discovered in our tiling array experiment were also removed.
3. All regions were sheared by 100 bp on both sides to remove any possible UTRs from surrounding annotation.
4. Any region less than 50 bp in length was discarded.

This resulted in 1,525 background regions comprising 708,315 unique bases. For each background region, we computed the average number of reads per base. We then compared each putative transcript to this distribution to determine to what degree a transcript exceeded what was observed in background regions. In order to declare a transcript as above background we computed the .8 quantile from the background region distribution and declared a transcript as present if the average number of reads per base exceeded this .8 quantile. The .8 threshold corresponds to detecting on average 75% of Verified ORFS. This was done separately for each mutant to provide a sample-specific background distribution and therefore a sample-specific threshold for detection.

In order to construct statistics for differential expression, we considered the following model. Let *X_i_*_,*j*_ denote the number of reads with left end in a region of interest (ROI) indexed by *j* = 1,…,*J*, and lane indexed by *i* = 1,…,*I*. Let *K_j_* denote the length of ROI *j* and let *a*(*i*) denote the type of sample assayed in lane *i*, i.e., *a*(*i*)∈{*wt*, *mt*}, where the short-hand notation *wt* and *mt* refers to the wild-type and mutant yeast strains, respectively. As a first-pass modeling attempt, suppose the counts *X_i_*_,*j*_ have a Poisson distribution with mean λ*_a_*_(*i*),*j*_β*_i_*, where λ*_a_*_(*i*),*j*_ is the parameter of interest representing the expression level of ROI *j* in samples of type *a*(*i*)∈{*wt*, *mt*} and β*_i_* is a lane effect. The maximum likelihood estimator (MLE) of the parameter λ*_a,j_*_,_, subject to the identifiability constraints Σ*_j_*λ*_a_*_,*j*_ = 1 for each *a*, is
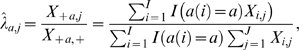
where I(.) is the indicator function, equal to one if the condition in parentheses is true and zero otherwise. Thus, intuitively, the MLE of the parameter λ*_a_*_,*j*_ is the proportion of the total read counts for type *a* samples that fall in ROI *j*.

#### B. Differential expression between mutant and wild-type strains

For a given ROI *j*, a natural measure of differential expression between mutant and wild-type strains is the log-ratio log(λ*_mt_*_,*j*_/λ*_wt_*_,*j*_). Using the delta method, it can be argued that the estimator 
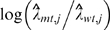
 has an approximate Gaussian distribution with mean log(λ*_mt_*_,*j*_/λ*_wt_*_,*j*_) and estimated variance
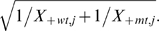



Thus, one can identify differentially expressed ROI between the mutant and wild-type strains based on the following test statistics:
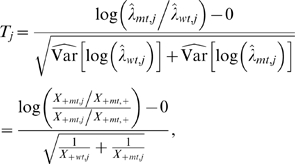
with approximate standard Gaussian distribution under the null hypothesis of no differential expression, i.e., λ*_mt_*_,*j*_ = λ*_wt_*_,*j*_.

#### C. Differential expression between ROI

Another question of interest is the comparison of expression levels between two ROI *j* and *j*′ for a given strain *a*∈{*wt*, *mt*}. In this case, a natural measure of differential expression is the log-ratio log((λ*_a_*_,*j*_/*K_j_*)/(λ*_a_*_,*j*′_/*K_j_*_′_)), which adjusts for differences in ROI length. Another application of the delta method suggests the following test statistic for determining whether ROI *j* and *j*′ are differentially expressed within strain *a*,
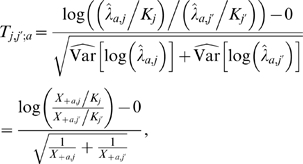
with approximate standard Gaussian distribution under the null hypothesis of no differential expression, i.e., λ*_a_*_,*j*_/*K_j_* = λ*_a_*_, *j*′_/*K_j_*_′_.

### Validation of Putative Unannotated from Other Studies' Transcripts Using UHTS

We applied the detected above background statistic described above with a cutoff of .8. Results are available in Table S2.

### Data Availability

All raw data have been deposited in the GEO database with accession number GSE11802.

## Supporting Information

Figure S1 Microarray data for A. Pre-Normalization and B. Post-Normalization stretches of Chromosome 4. The plots indicate that by forming the log ratio between the mutant and wild-type samples, we highlight differences between the two samples. At approximately base 248,000, we can see an unannotated upregulation in the mutant versus the wild-type. This region stands out much more prominently in the Post-Normalization plots, which was the intention of using the wild-type data.
(0.50 MB PDF)

Figure S2 Here we plot a goodness of fit statistic computed under the model described in the text. We compute an expected number of counts for each gene and compare this to the observed number of counts. This gives us a chi-squared statistic for each gene. If the gene counts are distributed as Y_j,i ∼ Poisson(\lambda_j\beta_i), then the test statistic will have a null distribution of Chisquare with lanes-1 degree of freedom. The plots demonstrate a very strong correspondence between our model and the observations.
(1.48 MB PDF)

Figure S3 Coverage plots as described in Figure 2 in the main text. These coverage plots were produced at a depth of 5.
(0.05 MB PDF)

Figure S4 Coverage plots as described in Figure 2 in the main text. hese coverage plots were produced at a depth of 10.
(0.05 MB PDF)

Figure S5 Unannotated non-intergenic transcripts found in this study. Each page shows one transcript, with the following information tracks from top to bottom: SGD annotation on the Watson and Crick strands), our tiling microarray data from the Crick and Watson strands (poly A+ RNA above total RNA), our UHTS data for the mutant and wild-type strains, tiling microarray data from David et al. for the Crick and Watson strands, UHTS data from Nagalakshmi et al., nucleosome position, data from Miura et al., and degree of conservation. The name and chromosome of origin of each transcript are indicated below each panel. For the UHTS data, each point plotted corresponds to the 5′ end of sequence reads, and the y position of the plotted point above the axis indicates (on a log scale) how many reads mapped to that position. Horizontal lines in a track indicate novel segments found in the corresponding study (black for forward strand and blue for reverse strand).
(6.83 MB PDF)

Figure S6 As in Figure S5, but for intergenic transcripts.
(13.33 MB PDF)

Figure S7 Density plot of conservation scores for different categories of segment.
(0.04 MB PDF)

Table S1 Table of validated transcripts. Here there are 566 rows, each corresponding to an individual cluster. The column metaName groups the clusters together into transcripts, so that there are 365 unique different metaNames.
(0.13 MB TXT)

Table S2 This table shows which previously reported unannotated transcripts were above background level in this study (use the column background20, with TRUE meaning that the transcript was detected).
(3.90 MB TXT)

Table S3 Table of RPKMs for our different datasets for SGD annotated features.
(0.66 MB TXT)

Text S1 Supplementary File Descriptions.
(0.09 MB DOC)
