# Supplementary figures and images for: Novel Low Abundance and Transient RNAs in Yeast Revealed by Tiling Microarrays and Ultra High–Throughput Sequencing Are Not Conserved Across Closely Related Yeast Species
Source: PLoS Genet. 2008 Dec 19;4(12):e1000299. doi: 10.1371/journal.pgen.1000299 (PMC2601015; doi:10.1371/journal.pgen.1000299)

A.

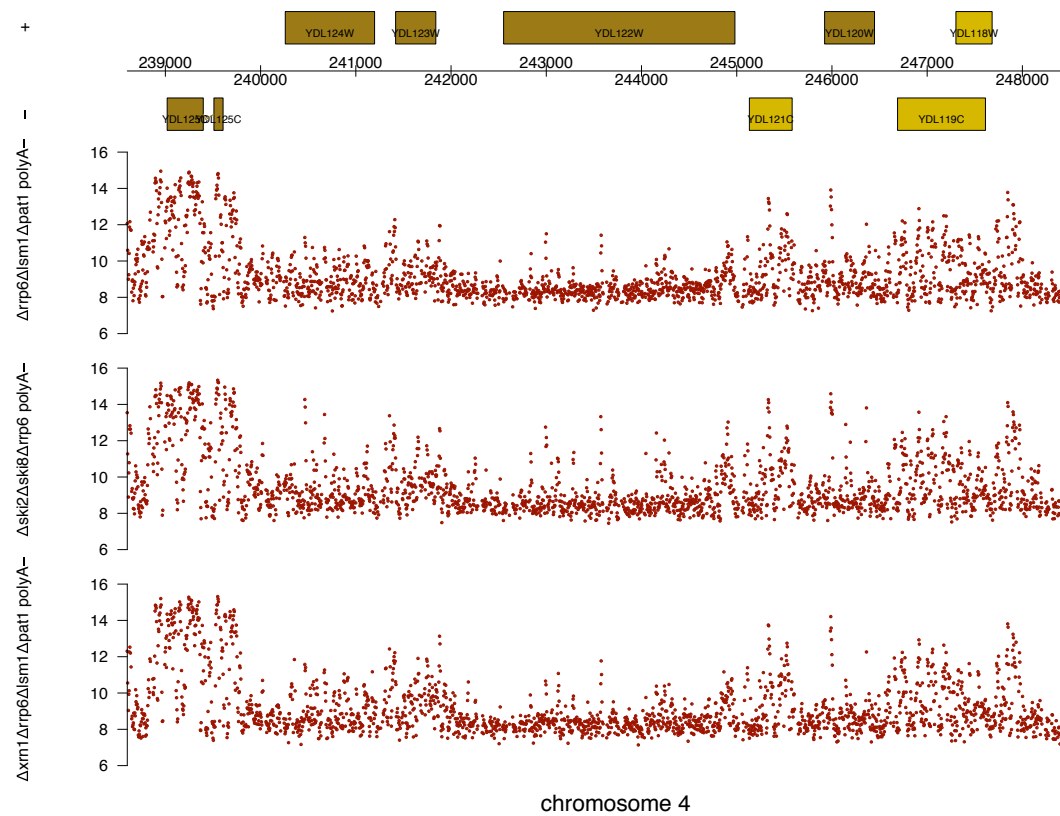

B.

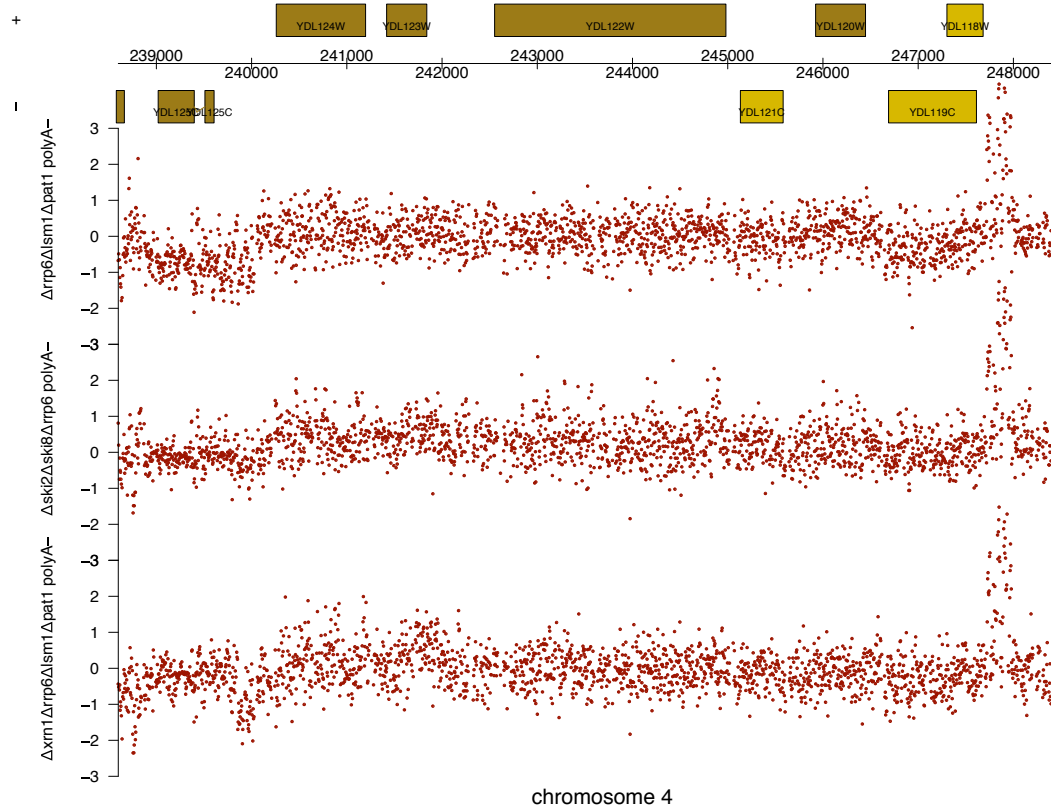

Figure S1

Supplement: Figure S1 — Microarray data for A. Pre-Normalization and B. Post-Normalization stretches of Chromosome 4. The plots indicate that by forming the log ratio between the mutant and wild-type samples, we highlight differences between the two samples. At approximately base 248,000, we can see an unannotated upregulation in the mutant versus the wild-type. This region stands out much more prominently in the Post-Normalization plots, which was the intention of using the wild-type data. (0.50 MB PDF) [file pgen.1000299.s001.pdf]

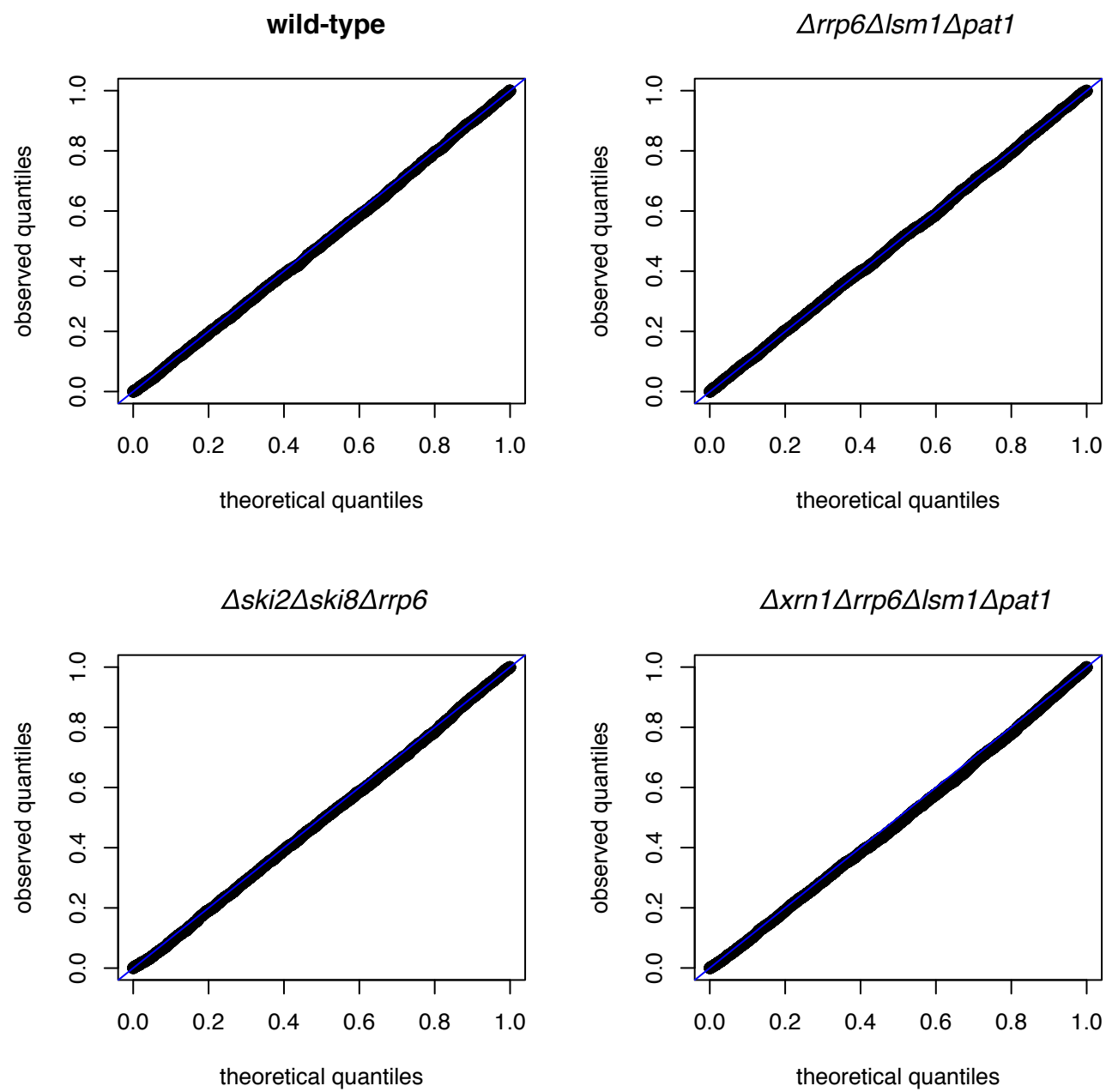

Figure S2

Supplement: Figure S2 — Here we plot a goodness of fit statistic computed under the model described in the text. We compute an expected number of counts for each gene and compare this to the observed number of counts. This gives us a chi-squared statistic for each gene. If the gene counts are distributed as Y_j,i ∼ Poisson(\lambda_j\beta_i), then the test statistic will have a null distribution of Chisquare with lanes-1 degree of freedom. The plots demonstrate a very strong correspondence between our model and the observations. (1.48 MB PDF) [file pgen.1000299.s002.pdf]

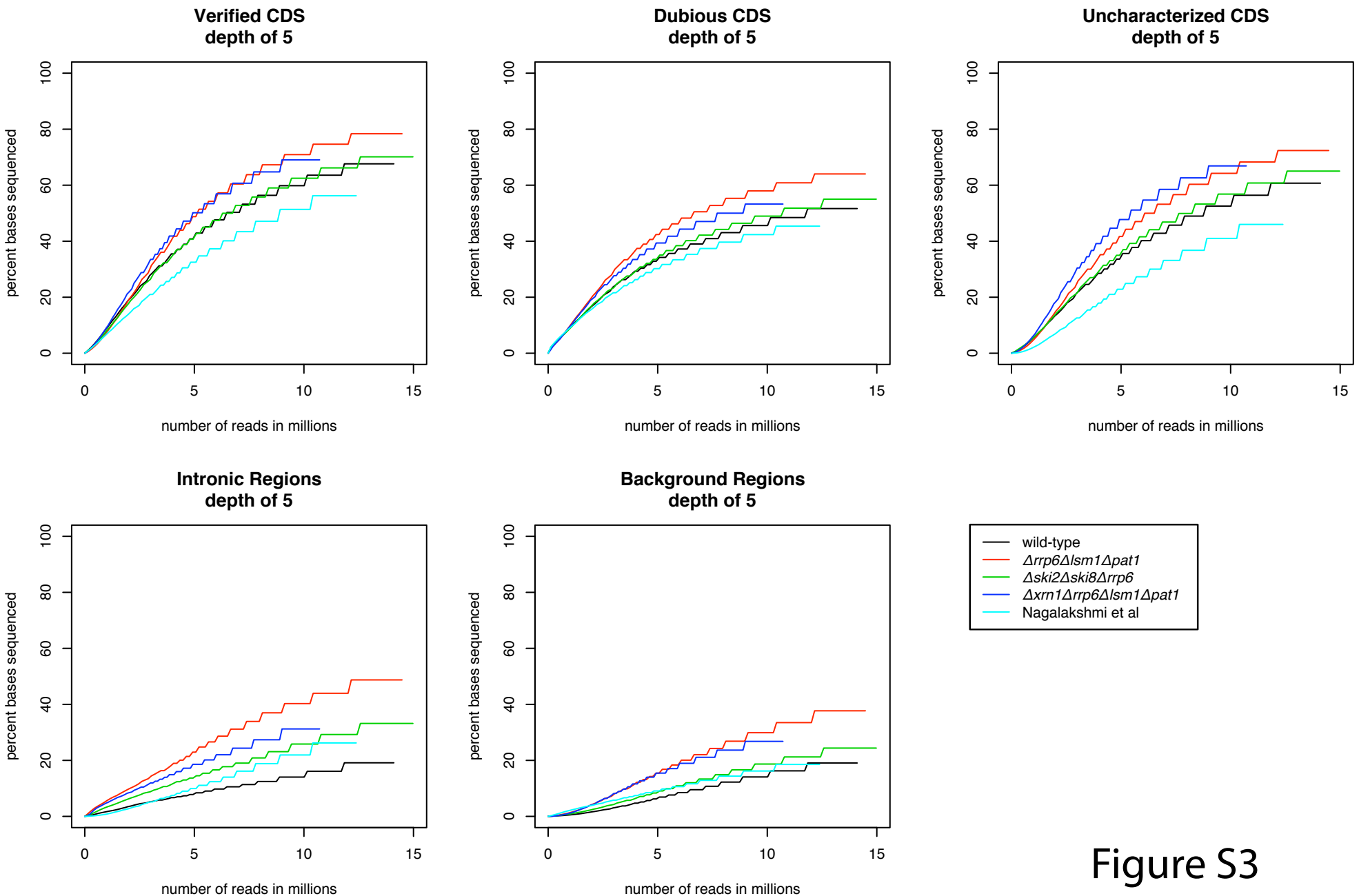

Figure S3

Supplement: Figure S3 — Coverage plots as described in Figure 2 in the main text. These coverage plots were produced at a depth of 5. (0.05 MB PDF) [file pgen.1000299.s003.pdf]

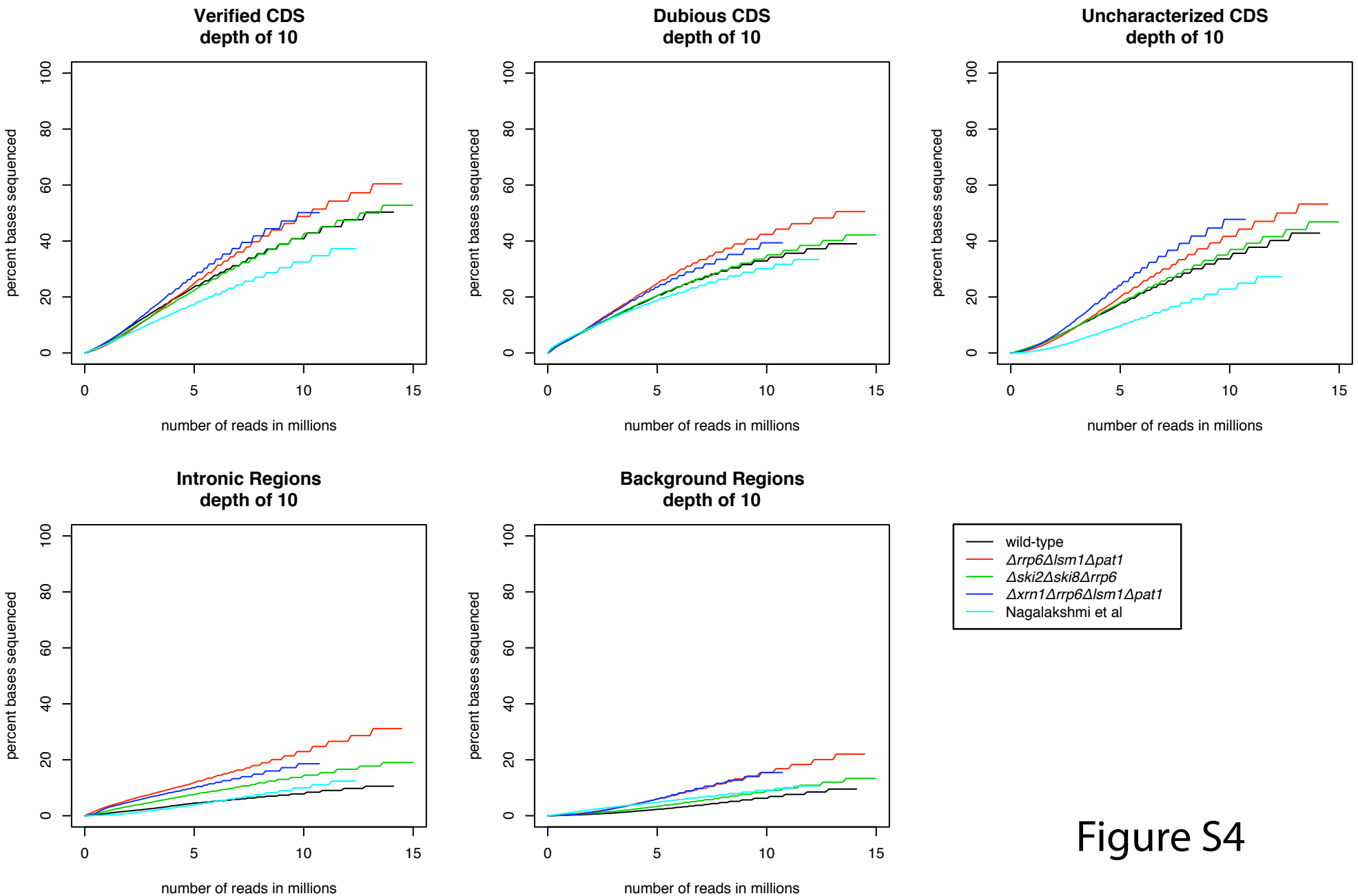

Figure S4

Supplement: Figure S4 — Coverage plots as described in Figure 2 in the main text. hese coverage plots were produced at a depth of 10. (0.05 MB PDF) [file pgen.1000299.s004.pdf]

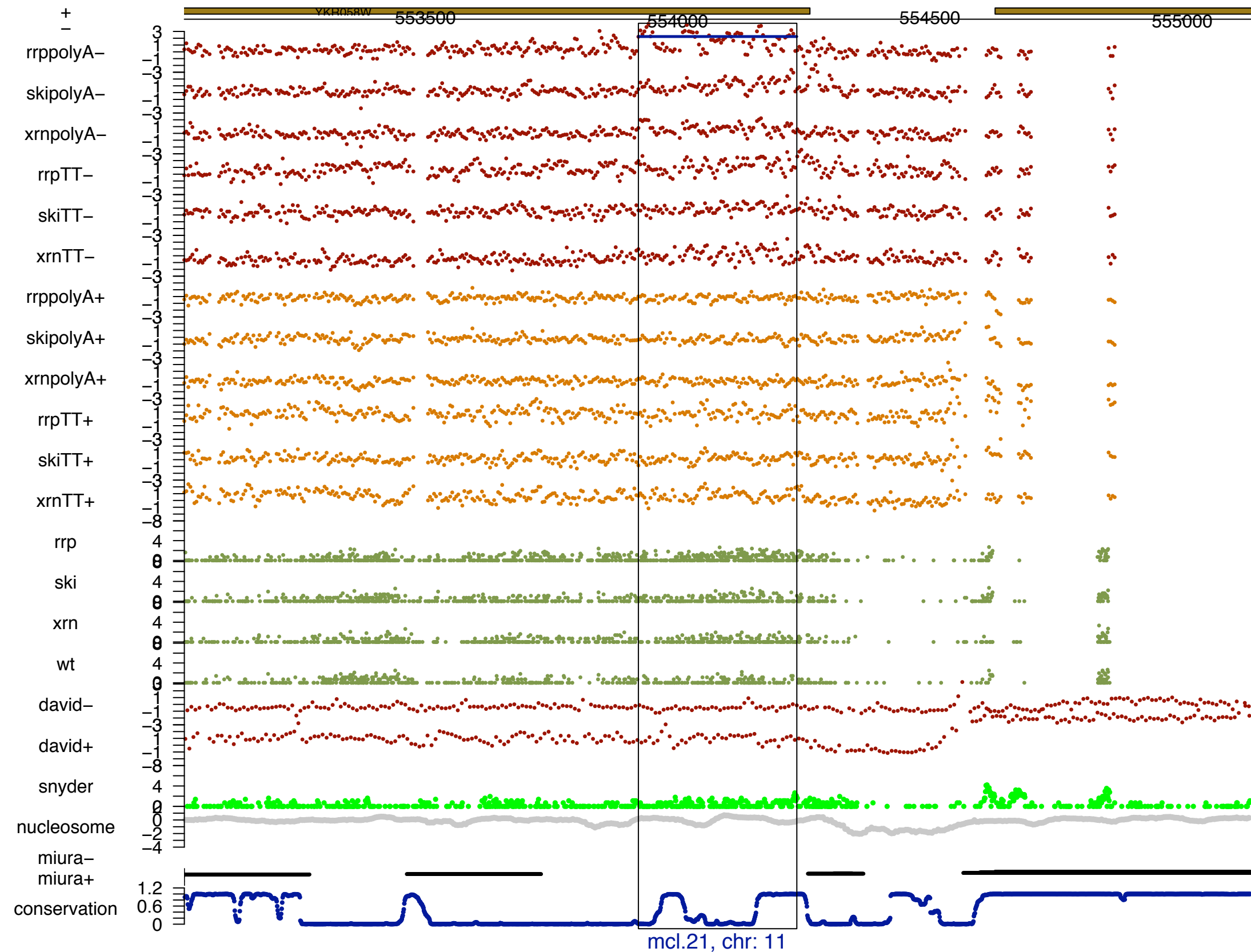

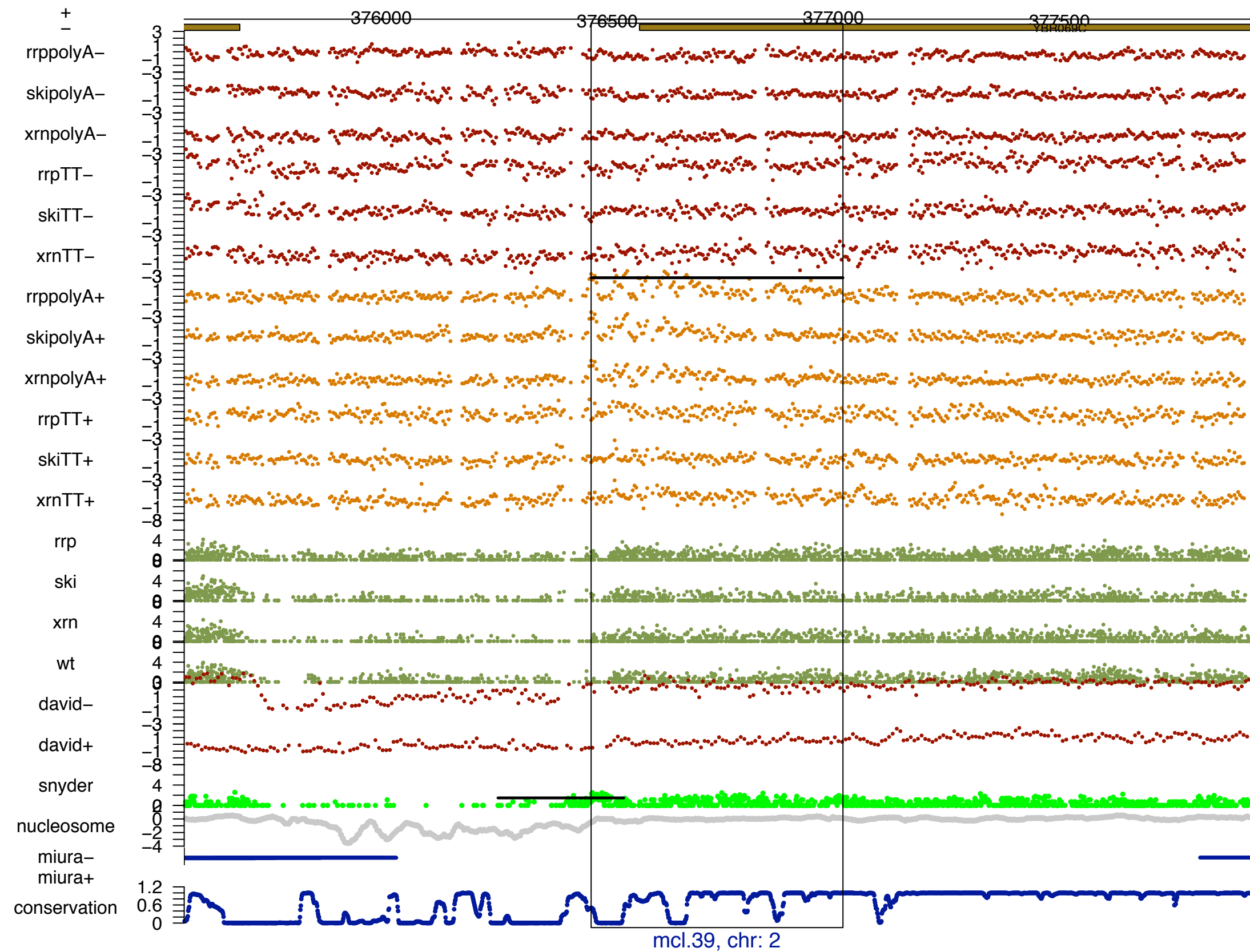

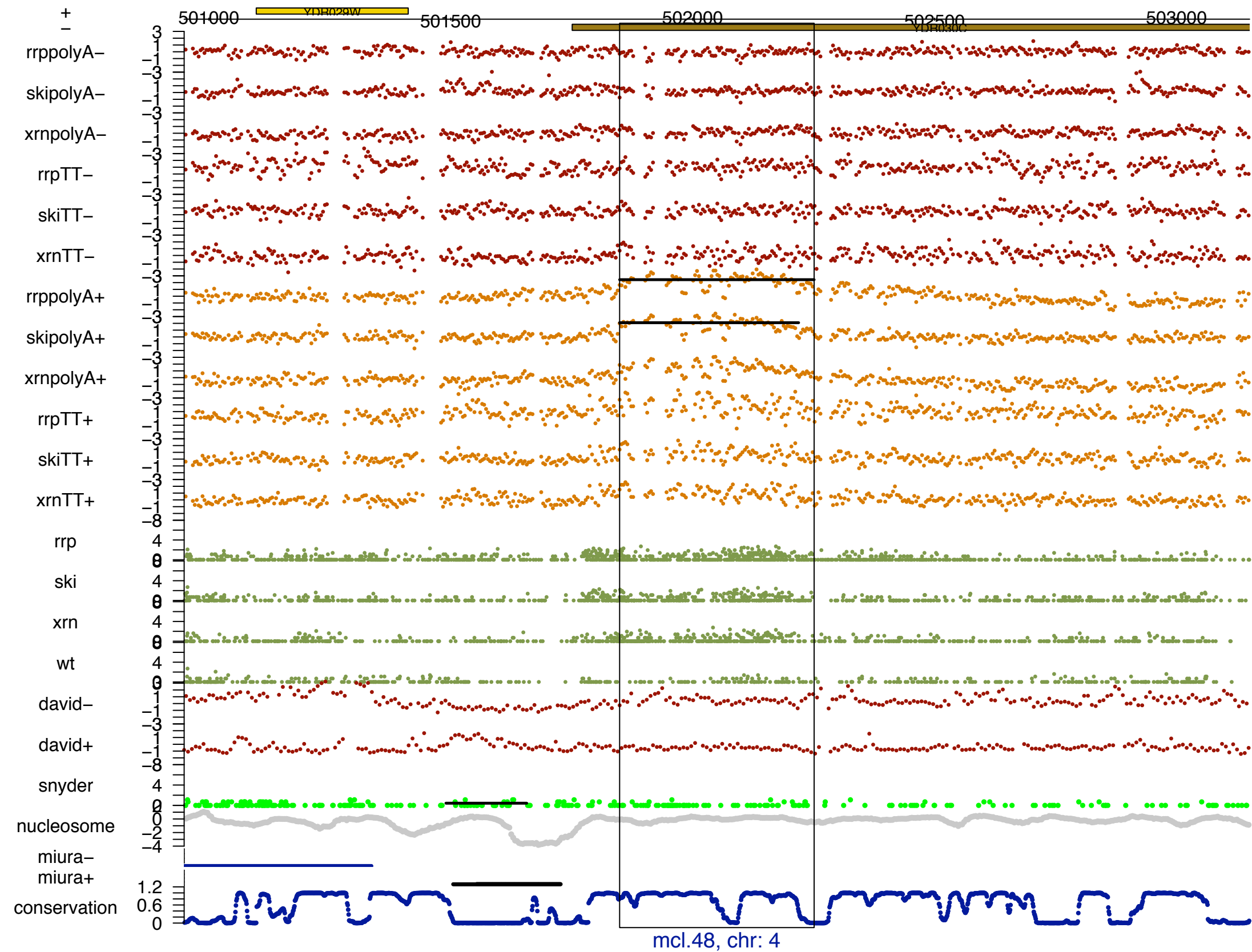

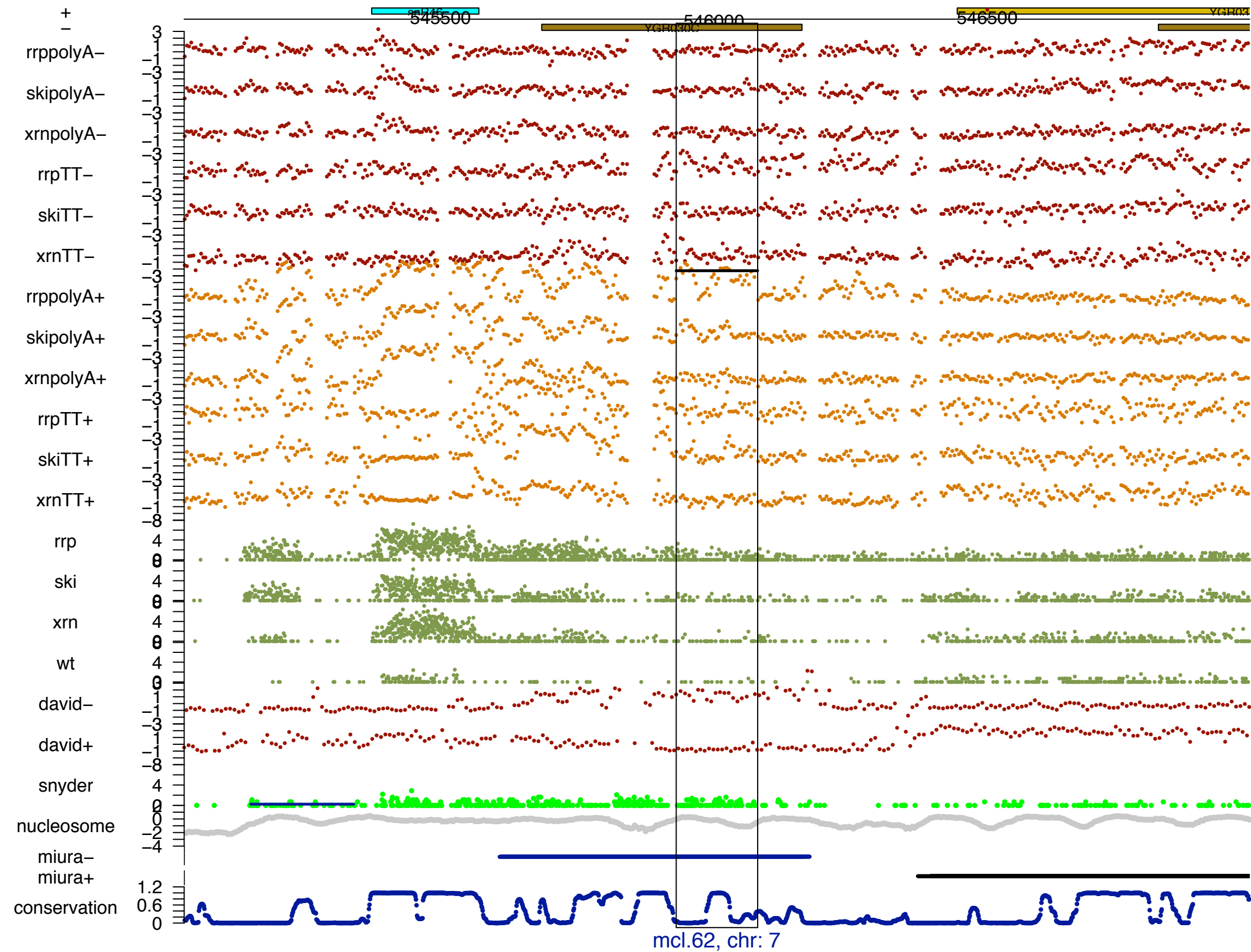

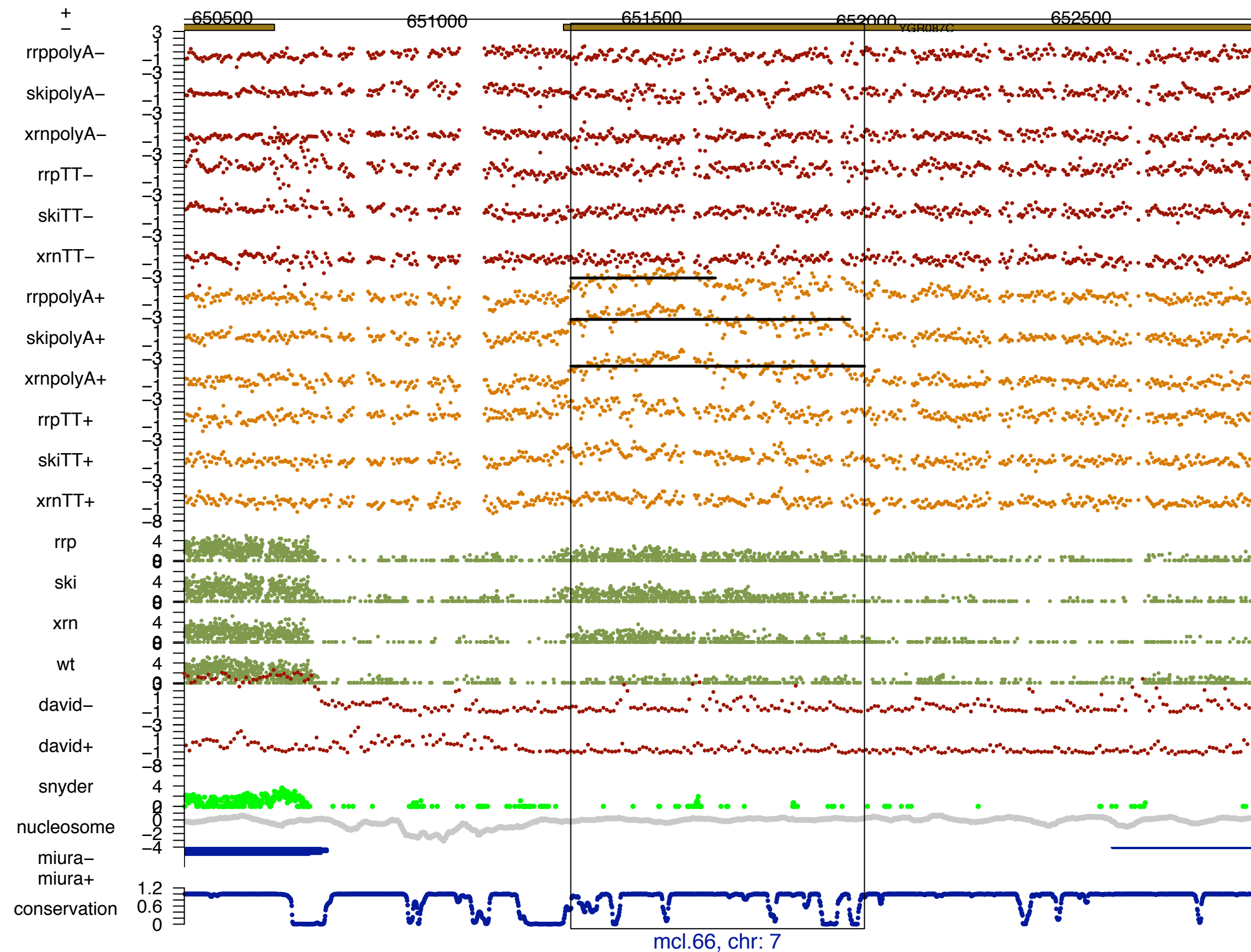

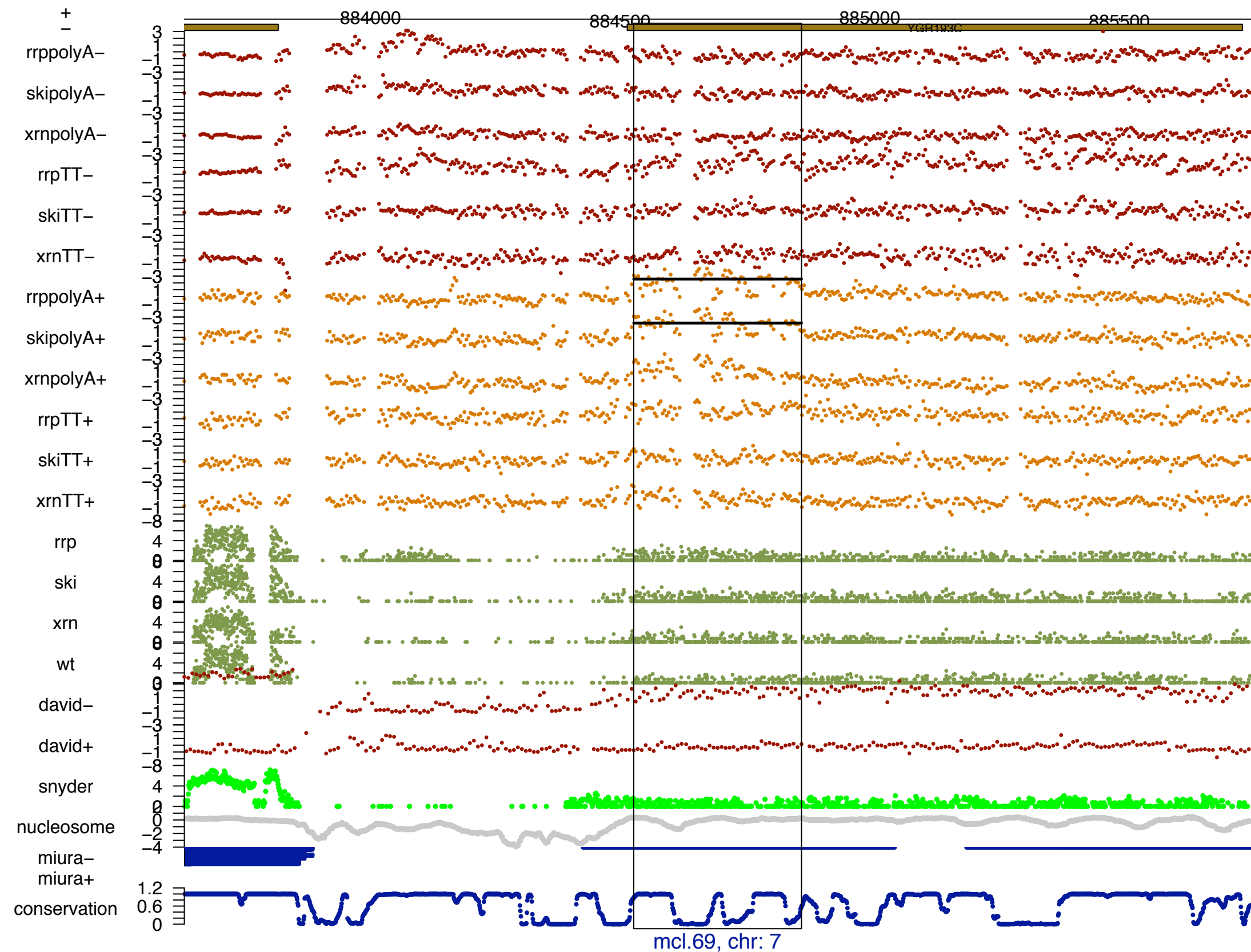

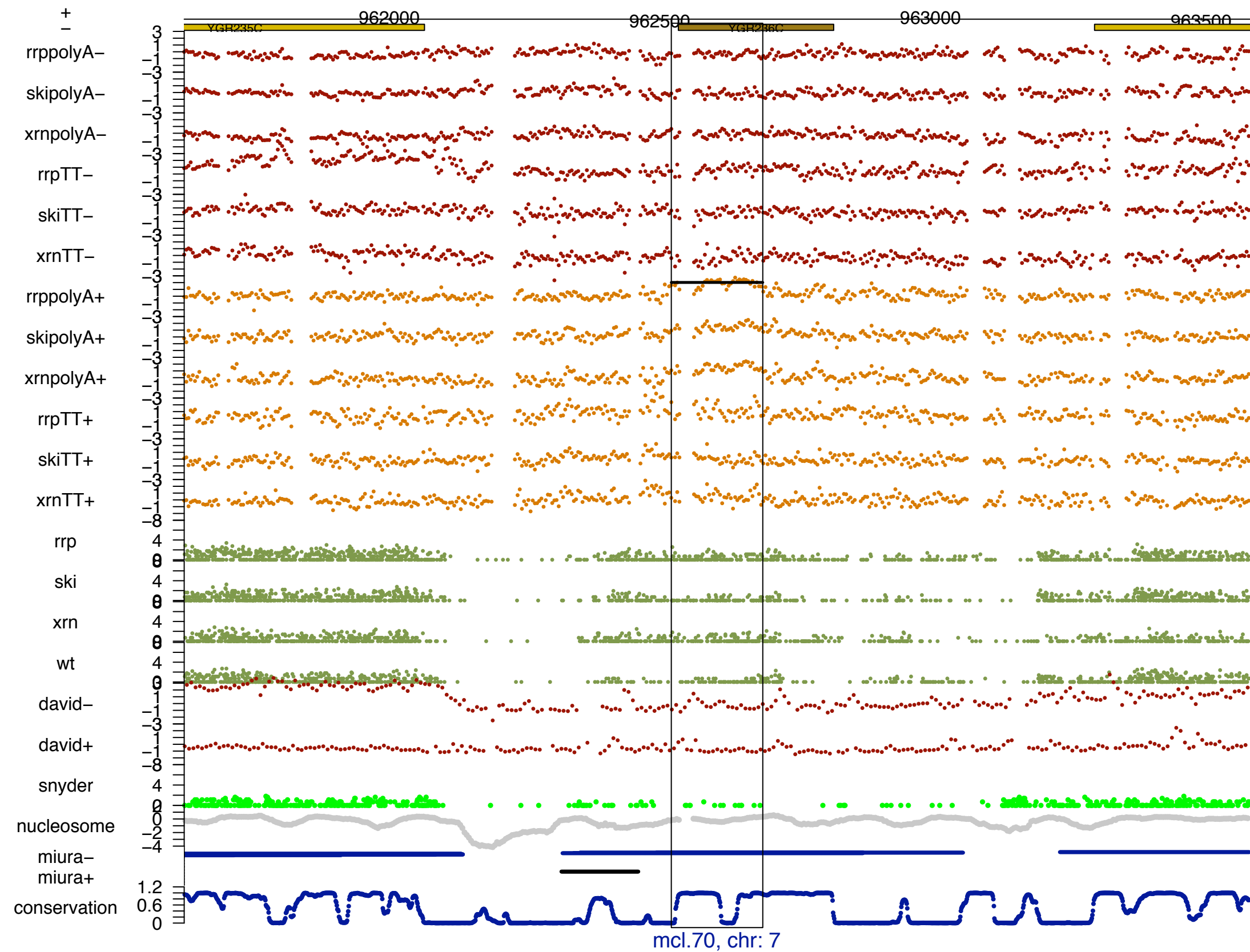

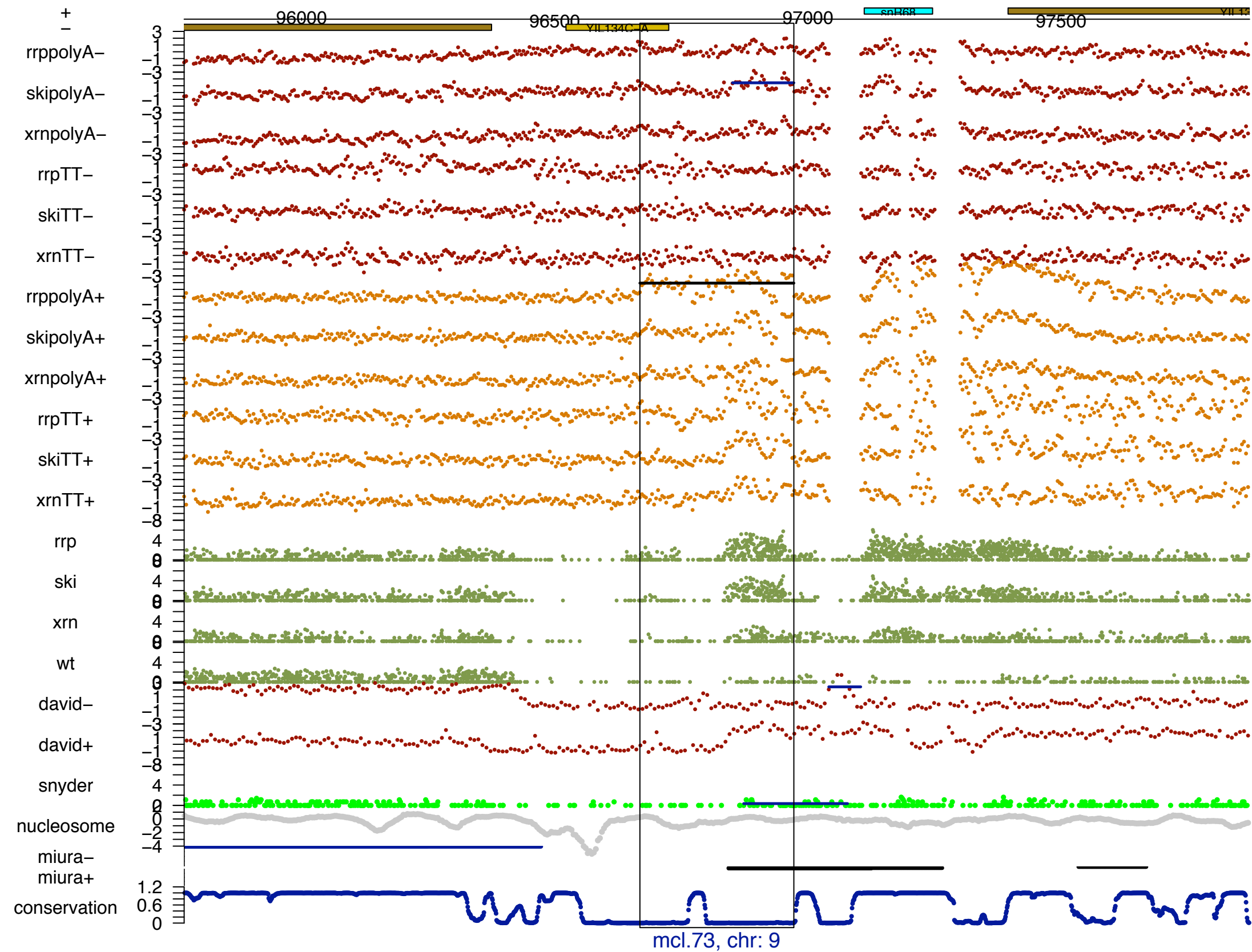

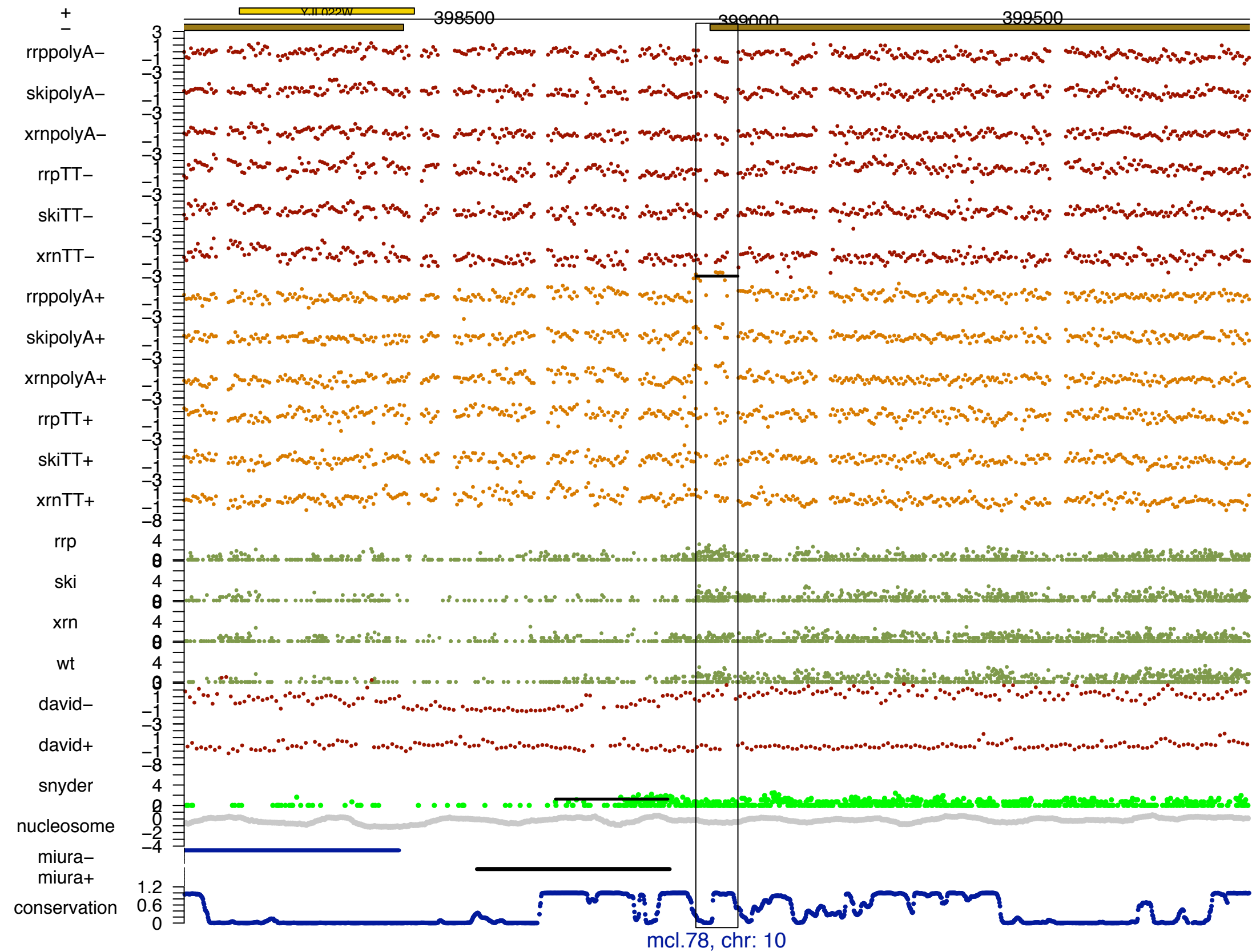

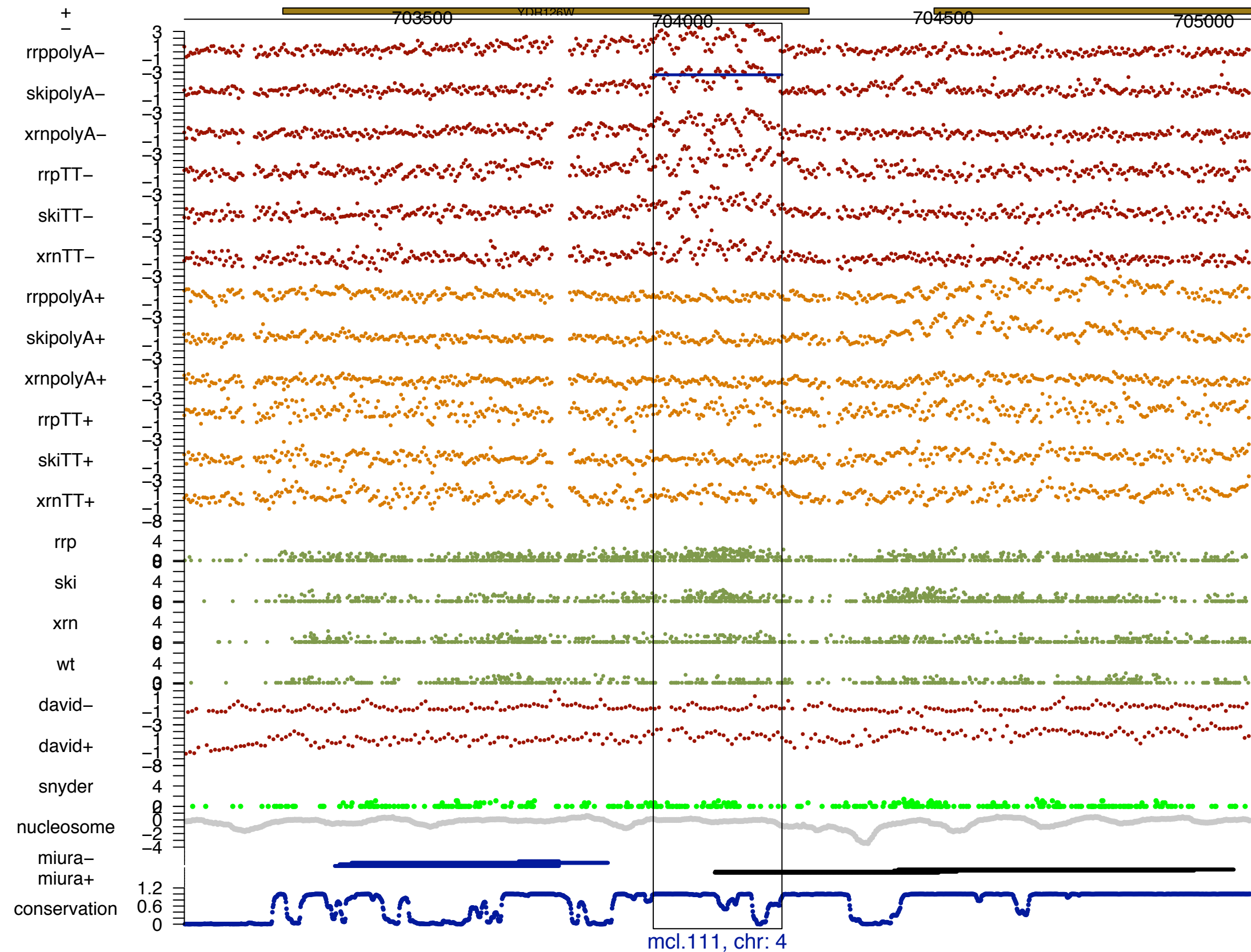

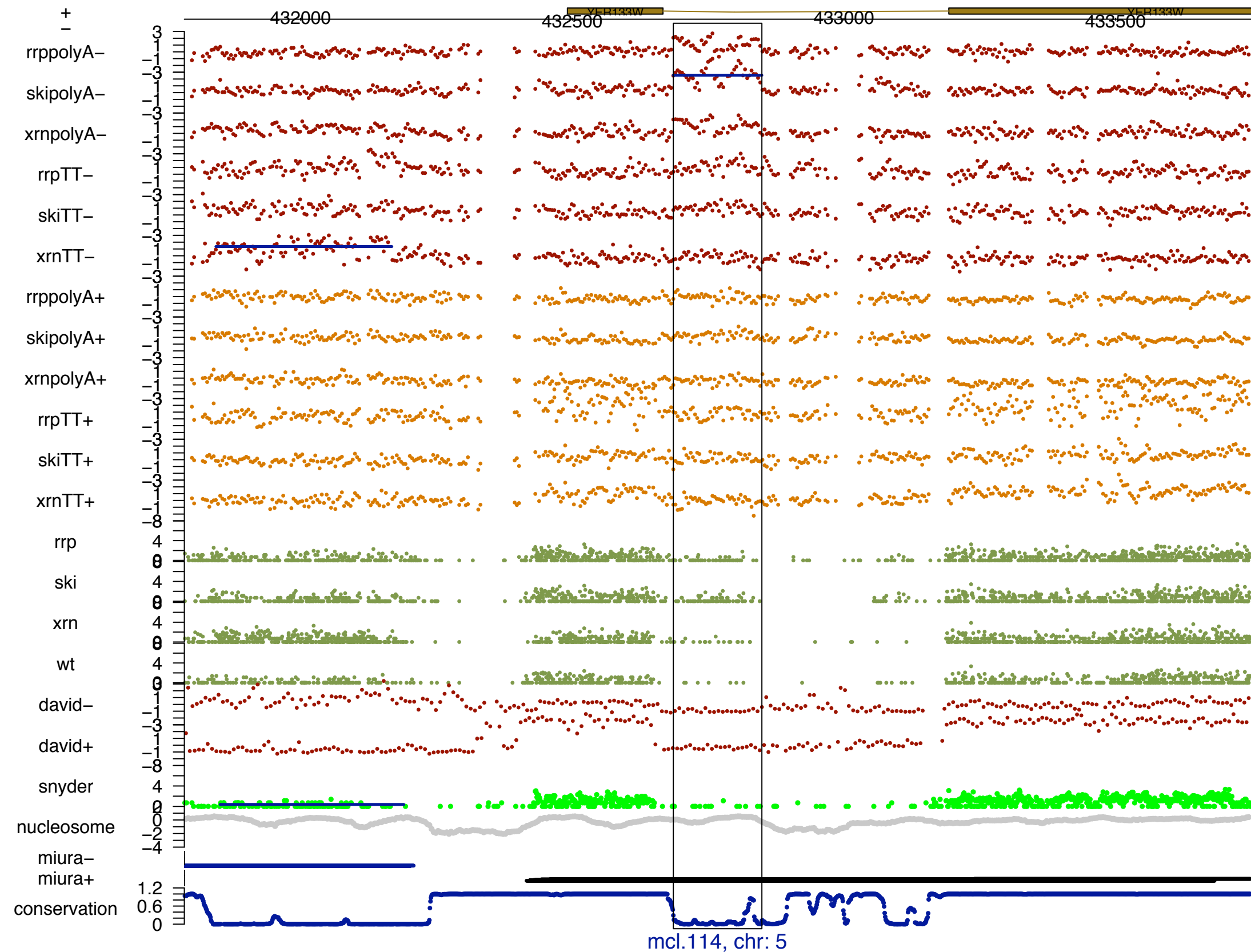

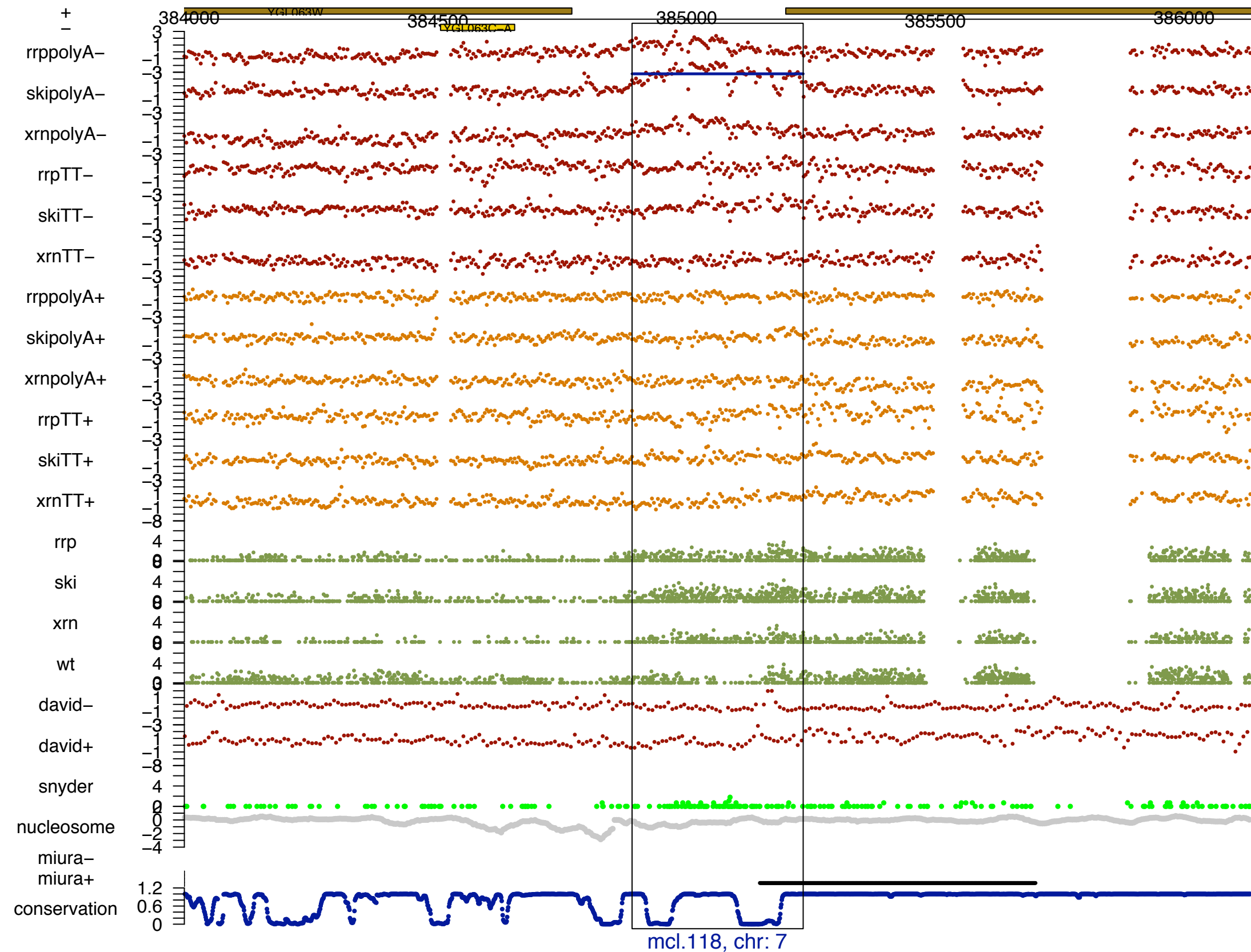

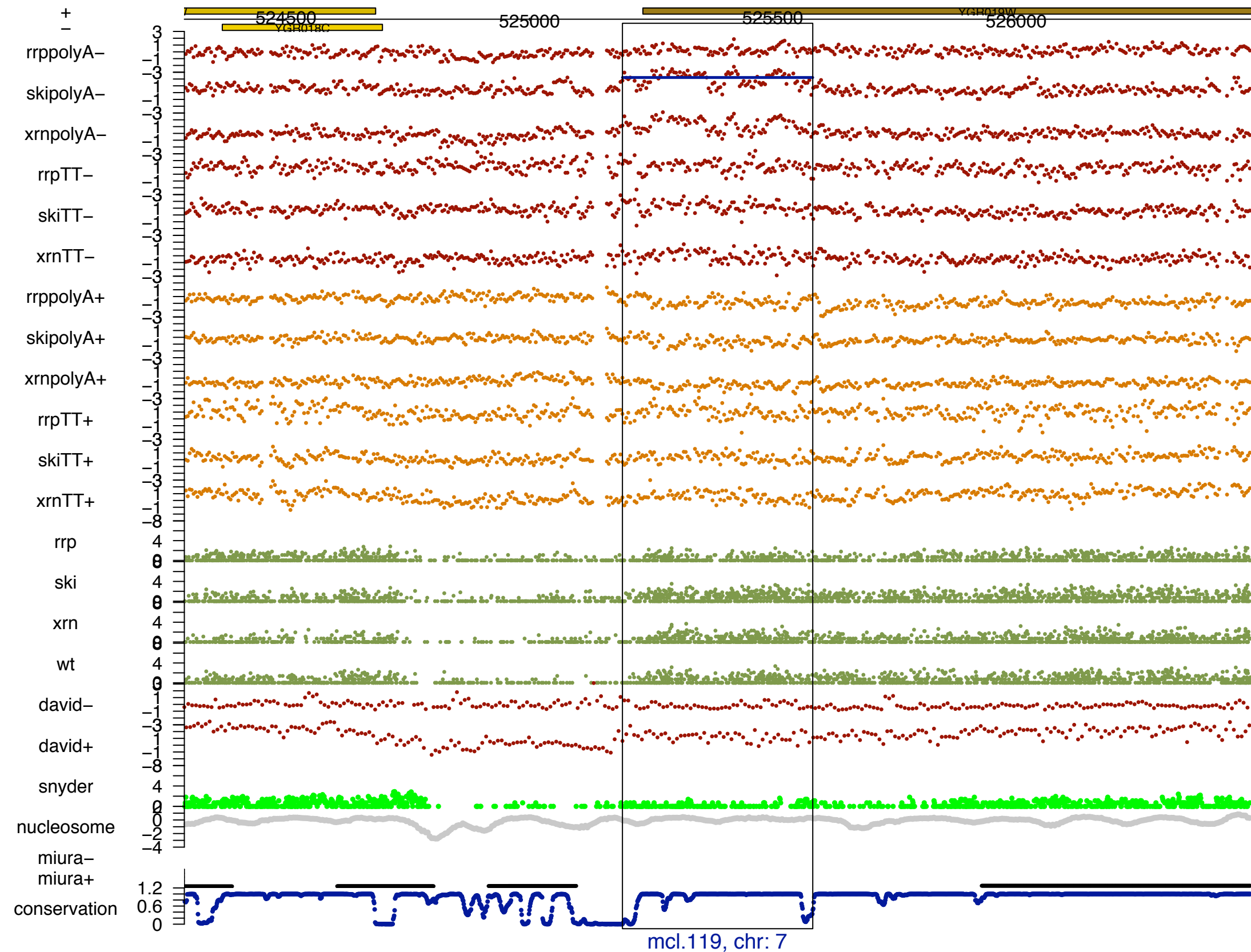

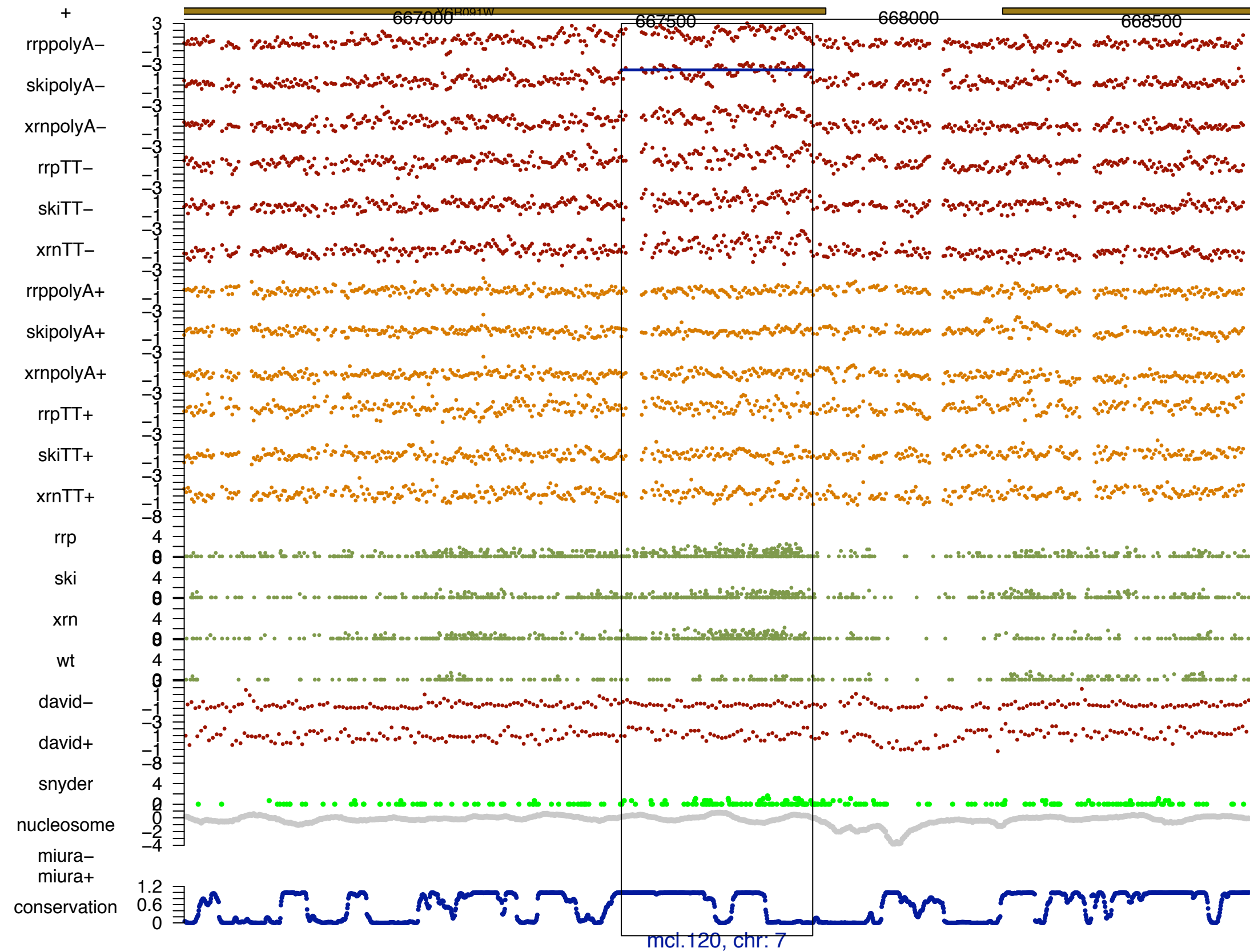

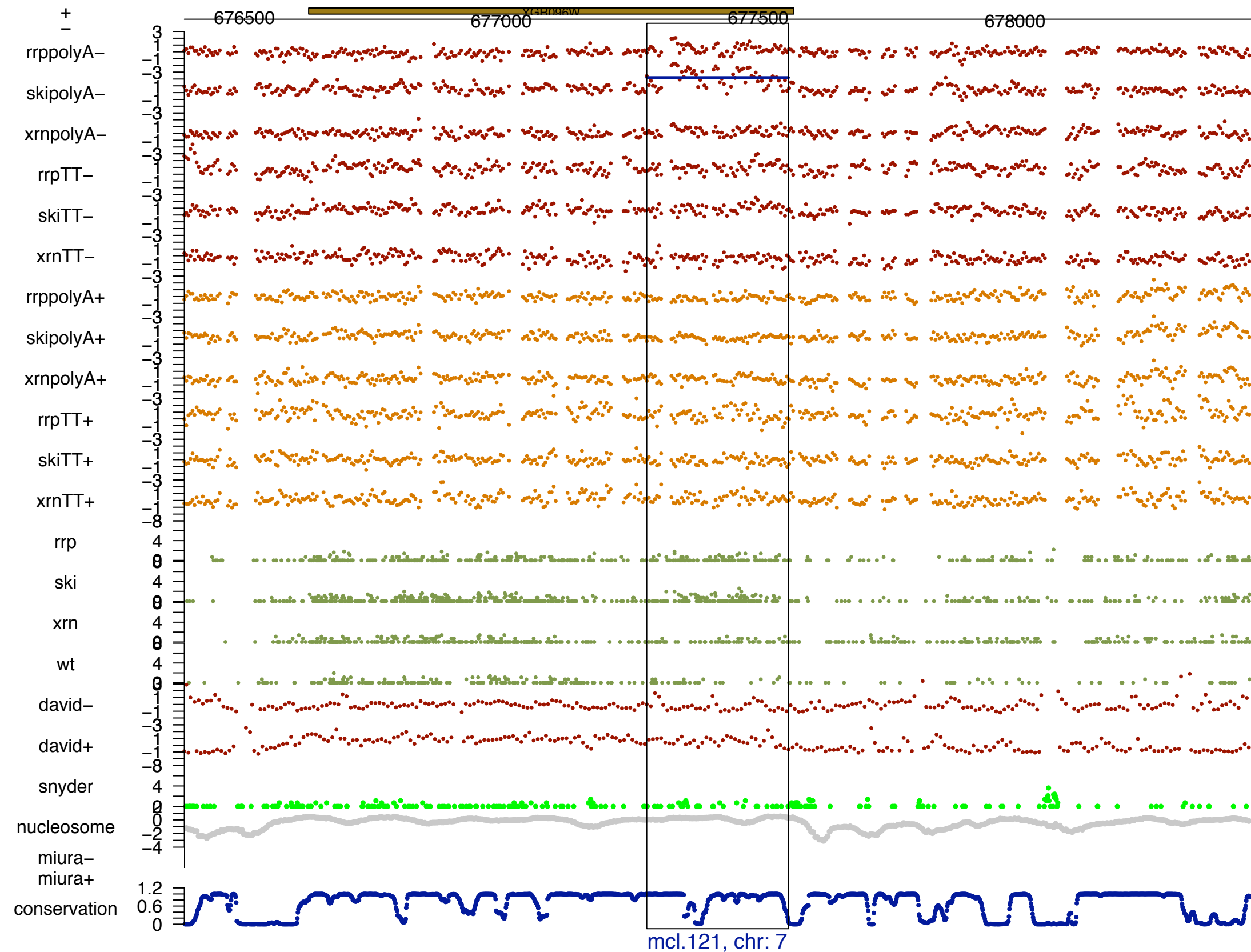

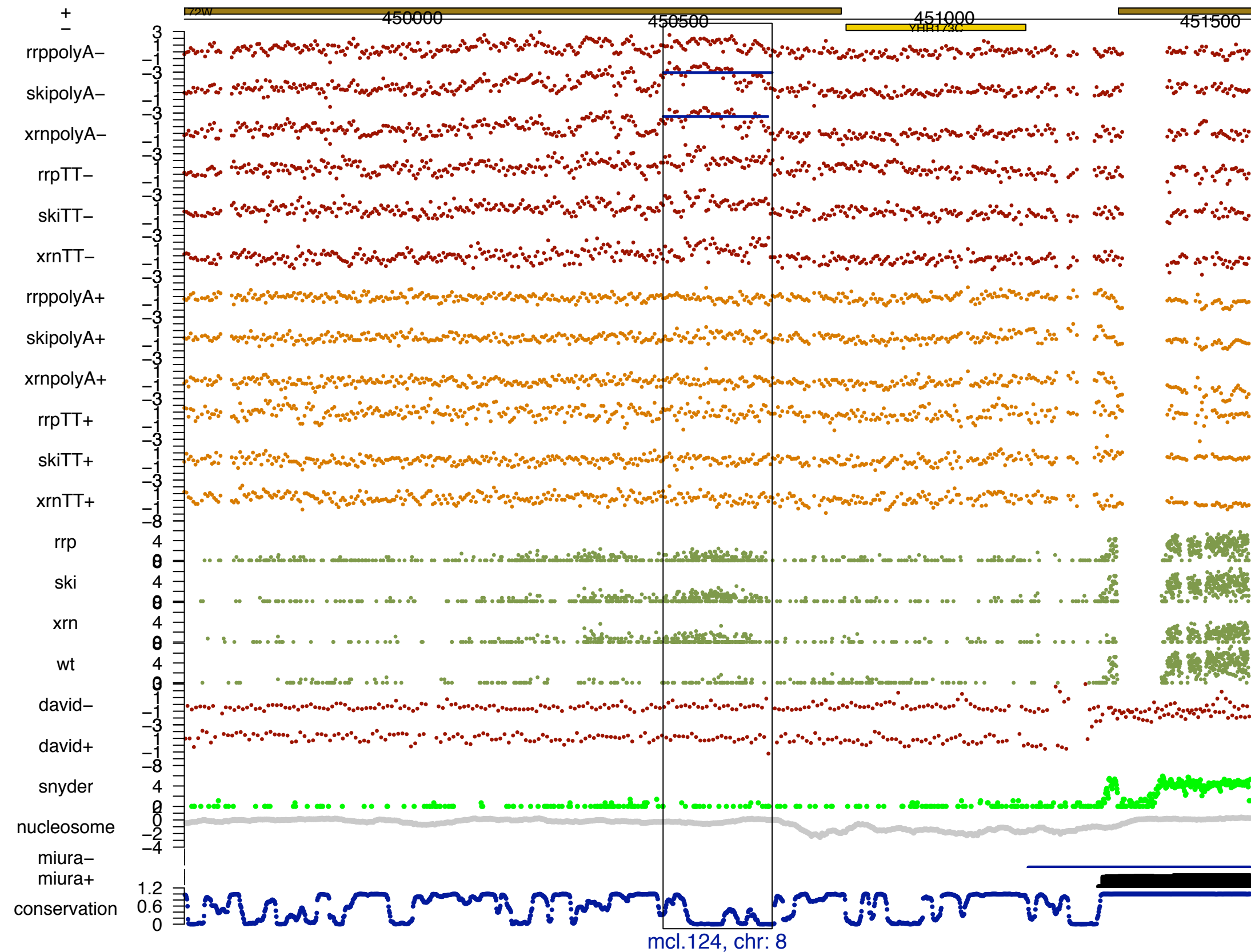

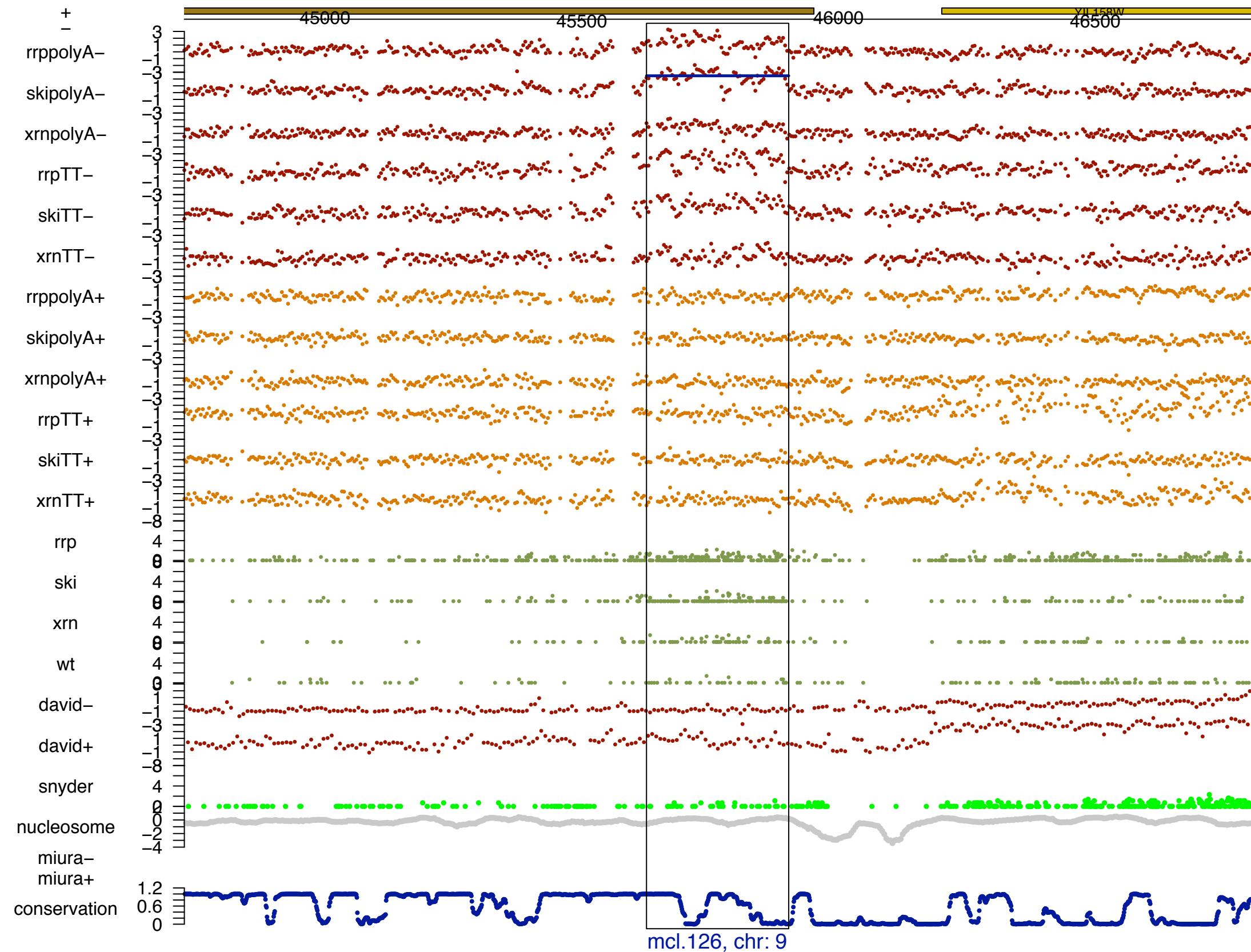

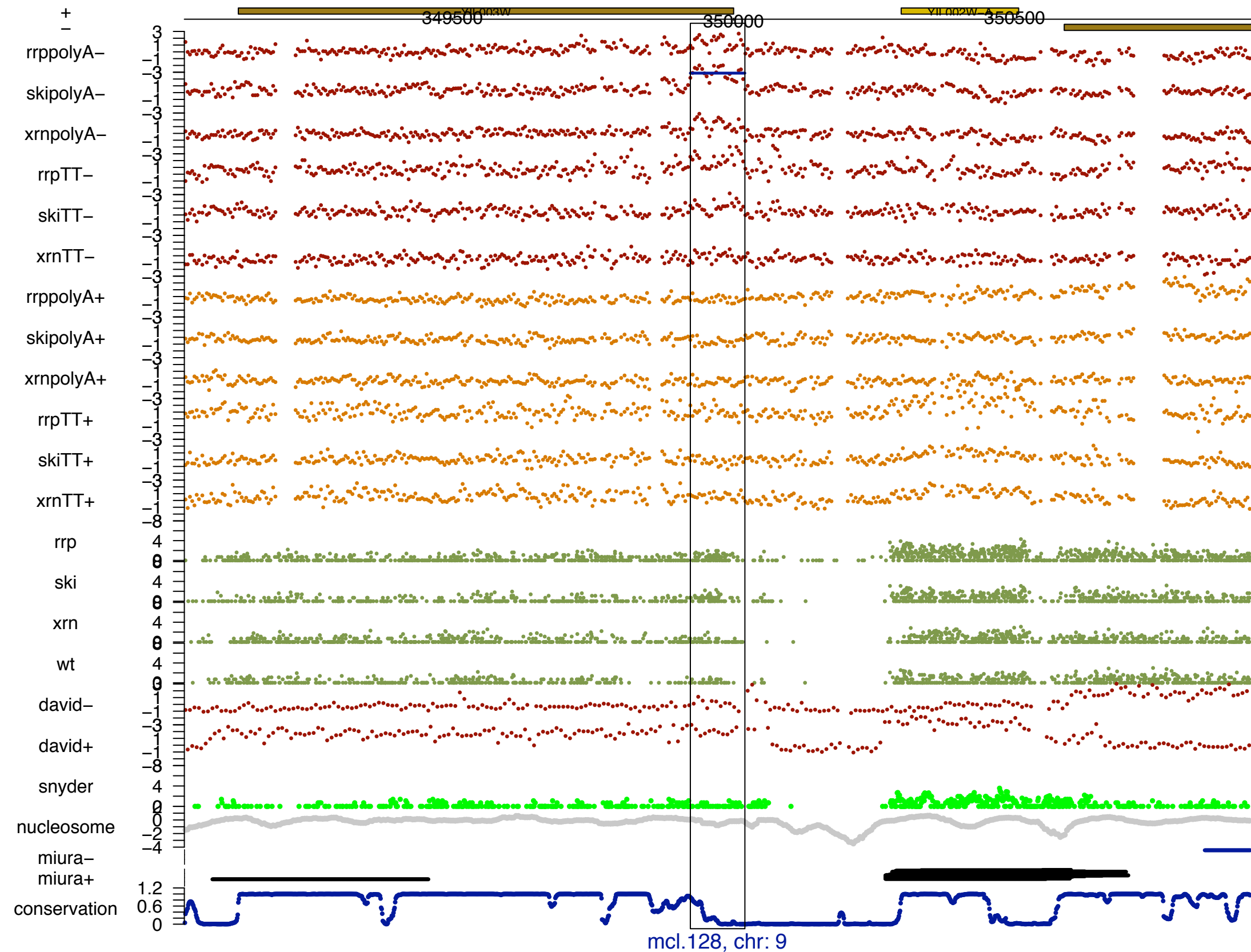

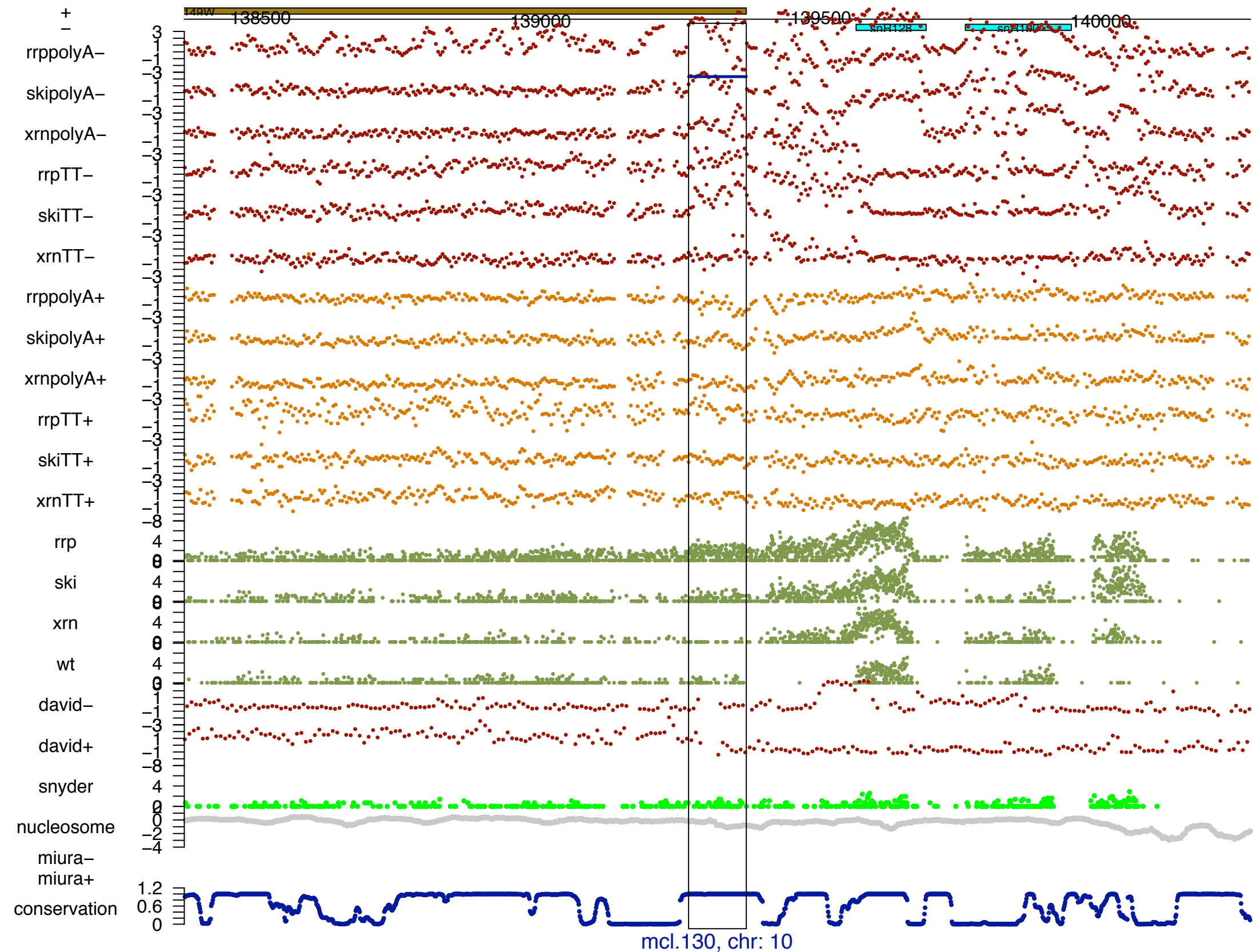

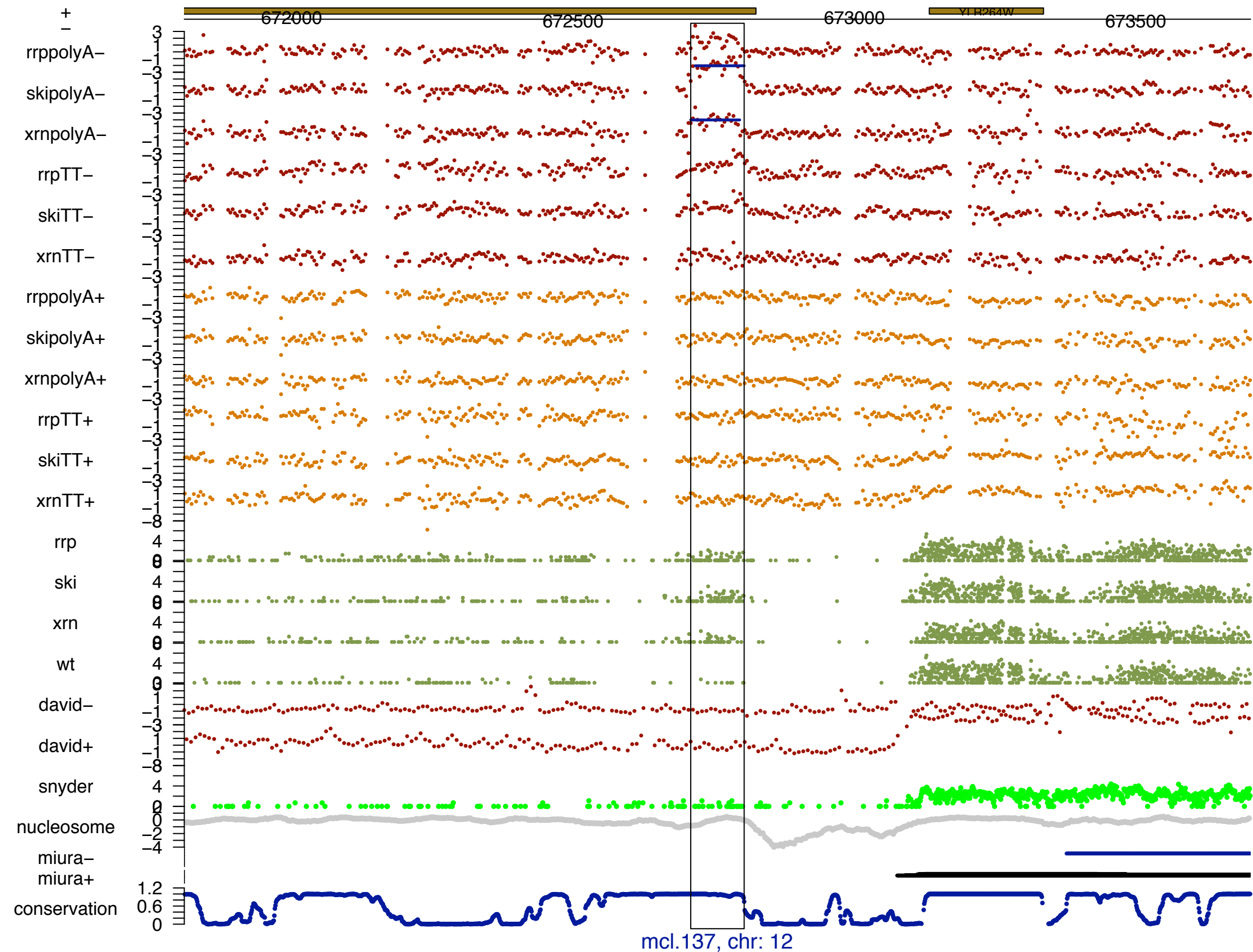

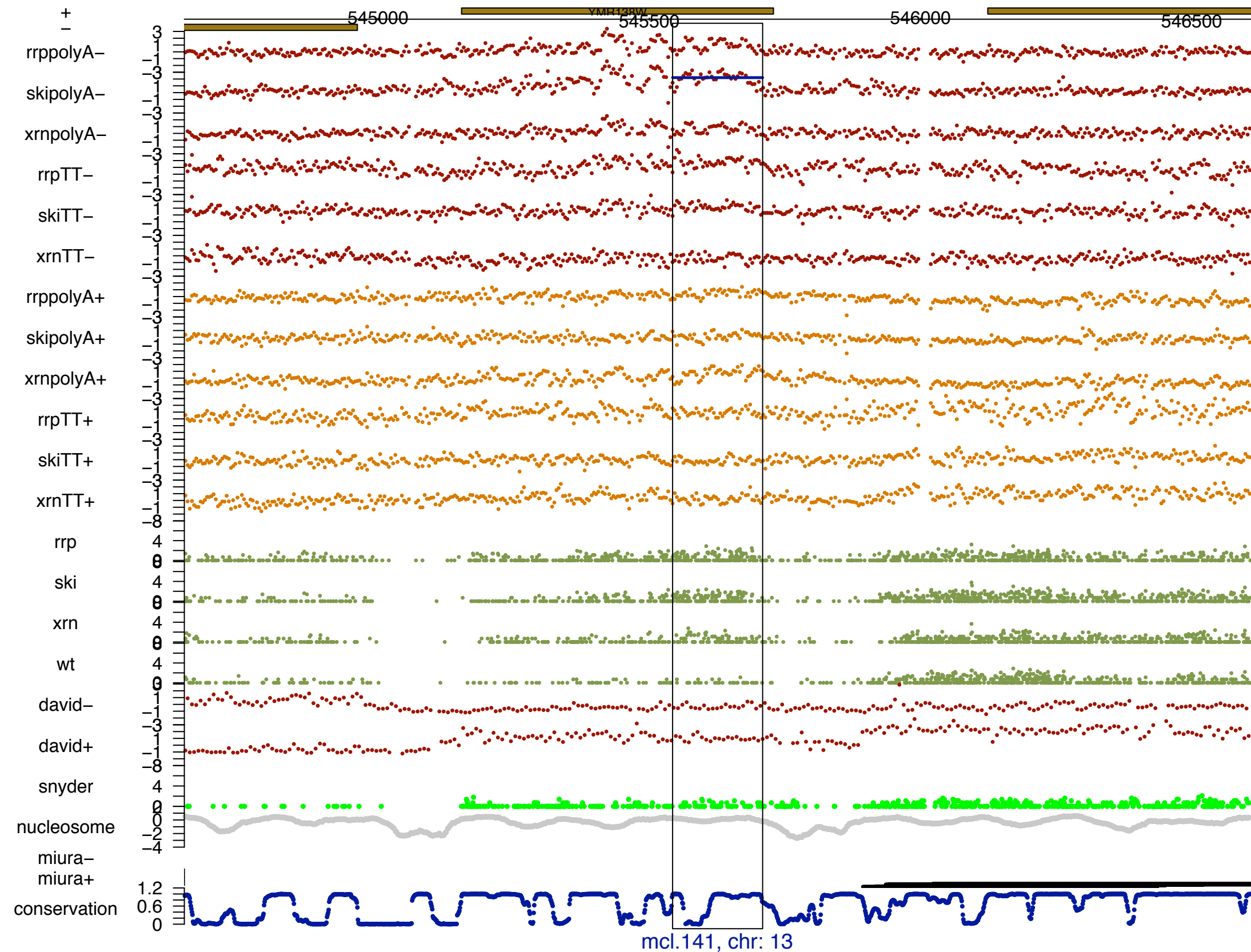

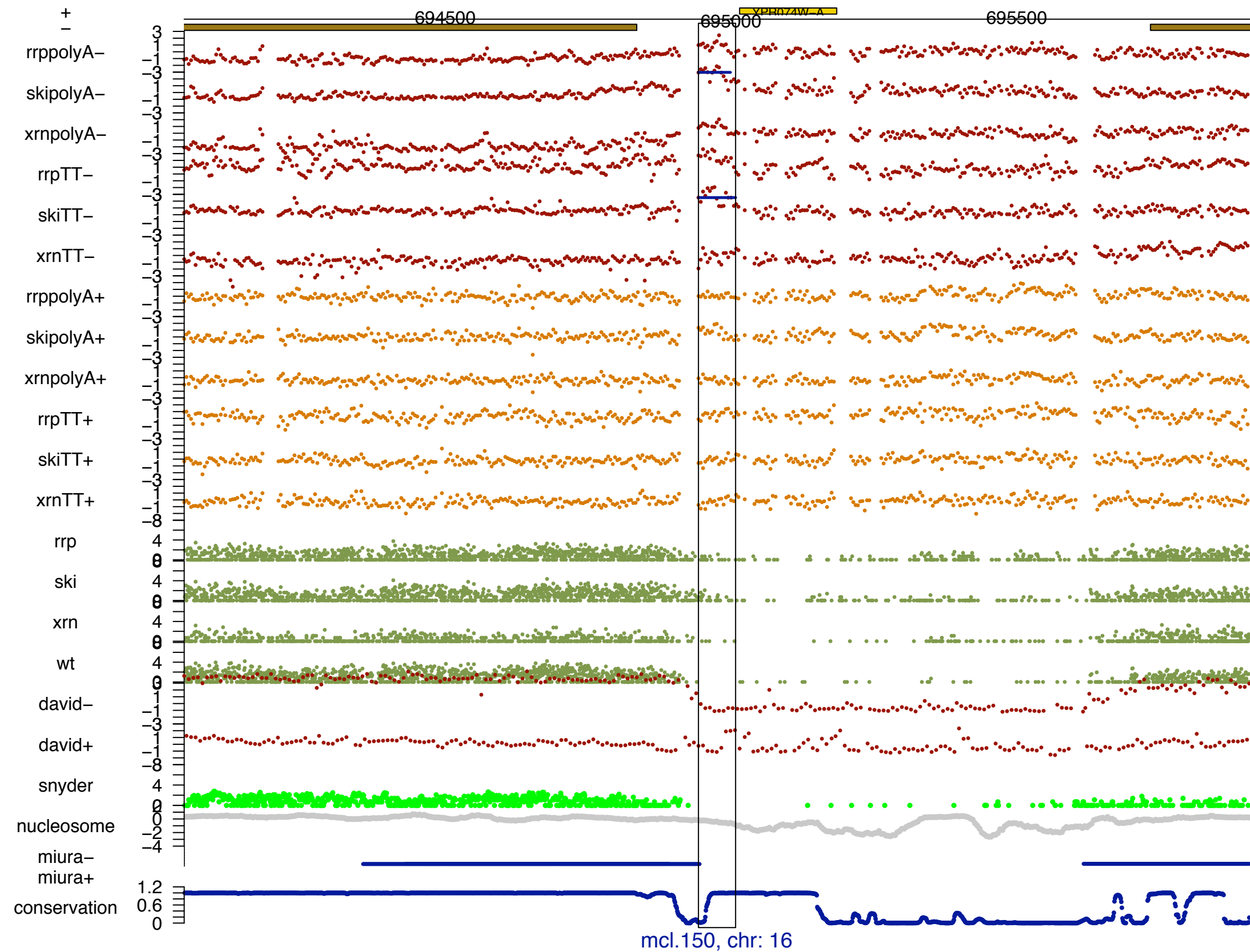

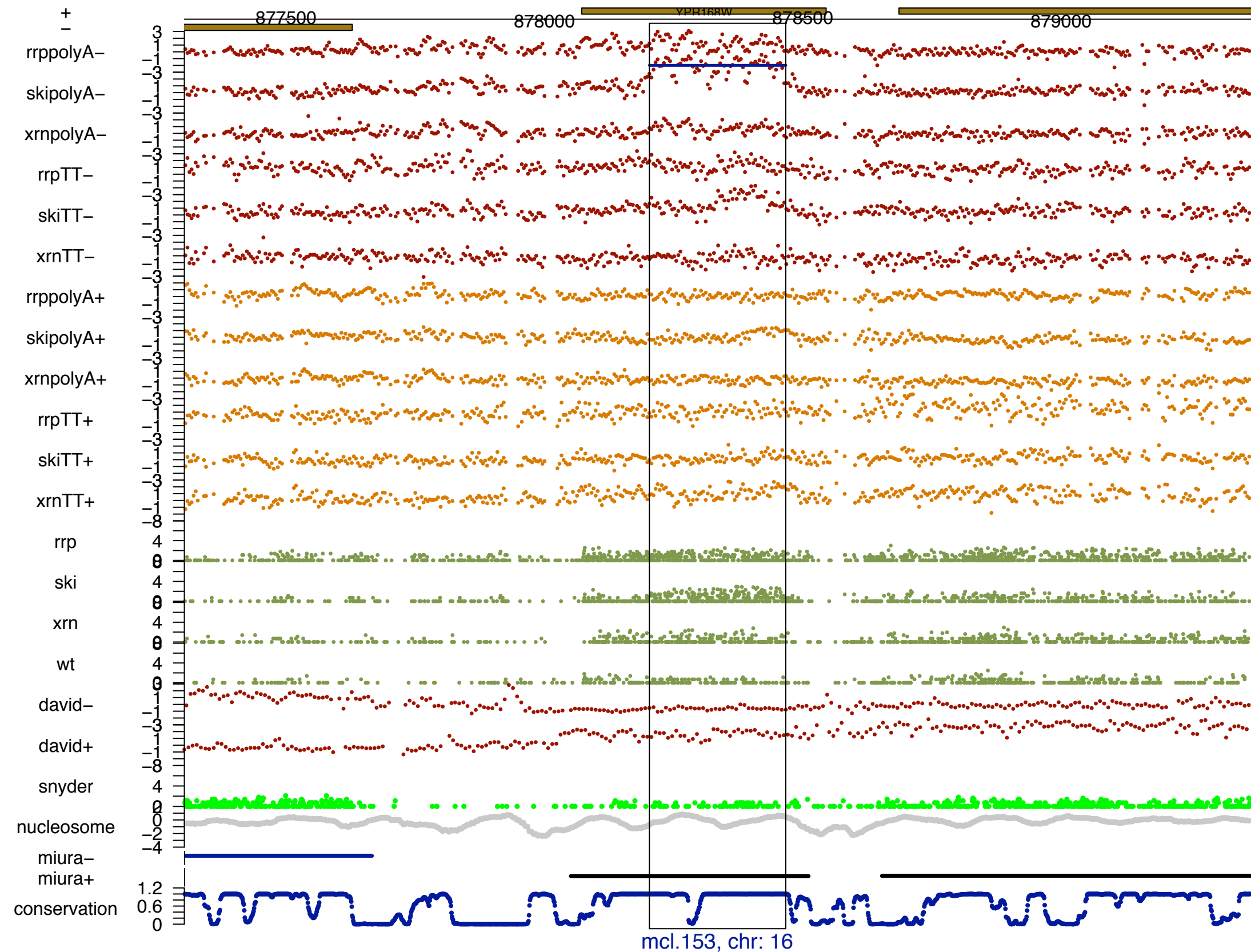

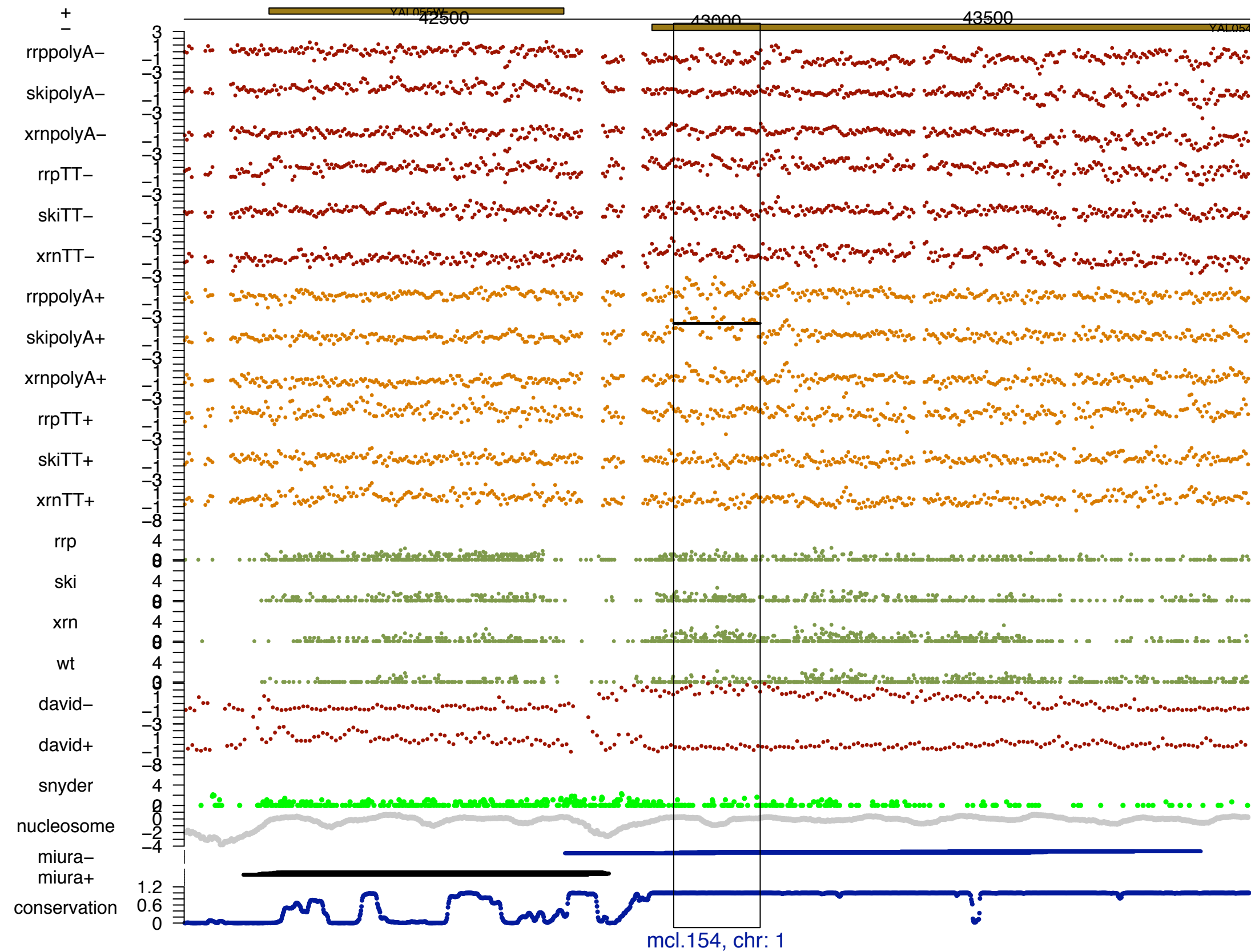

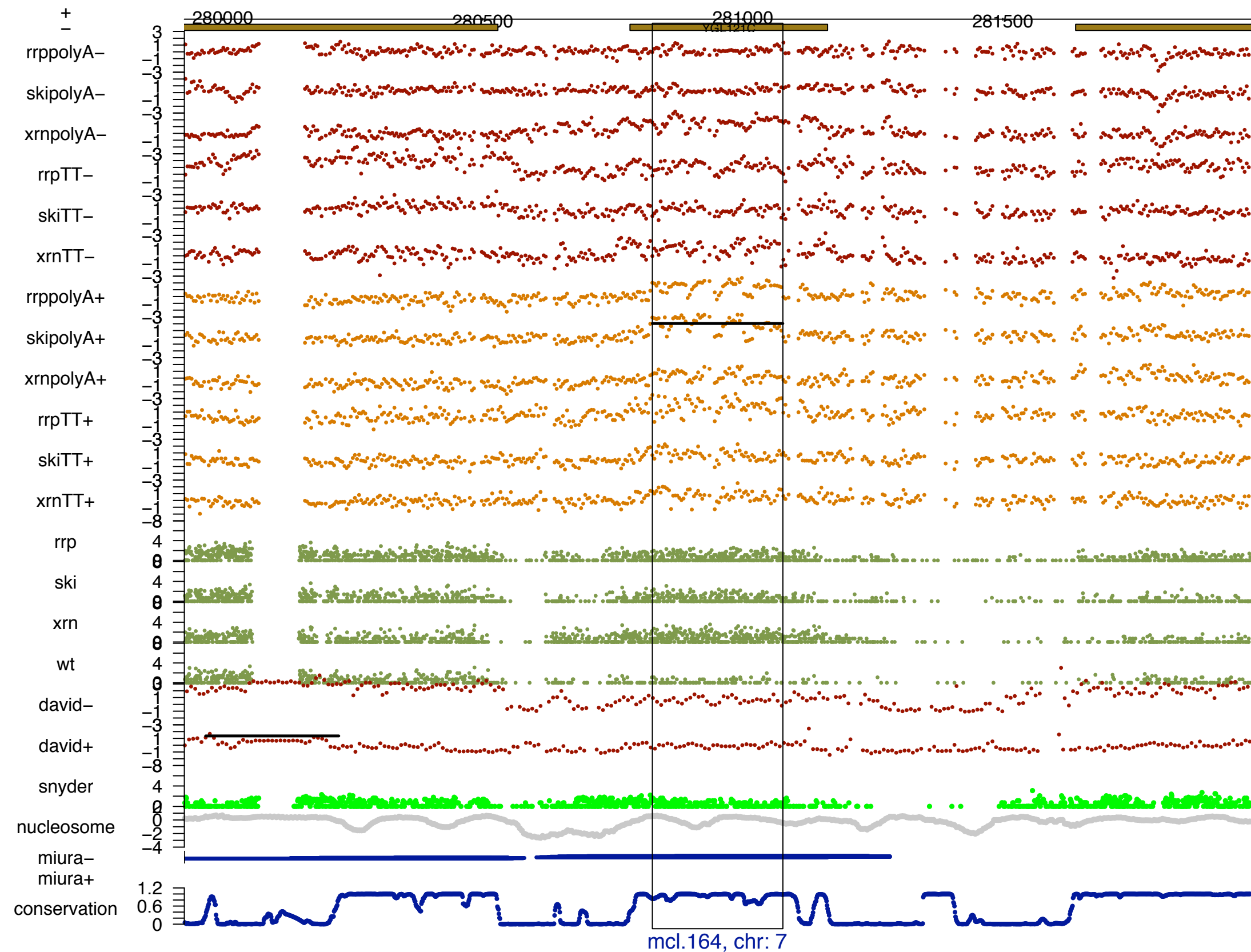

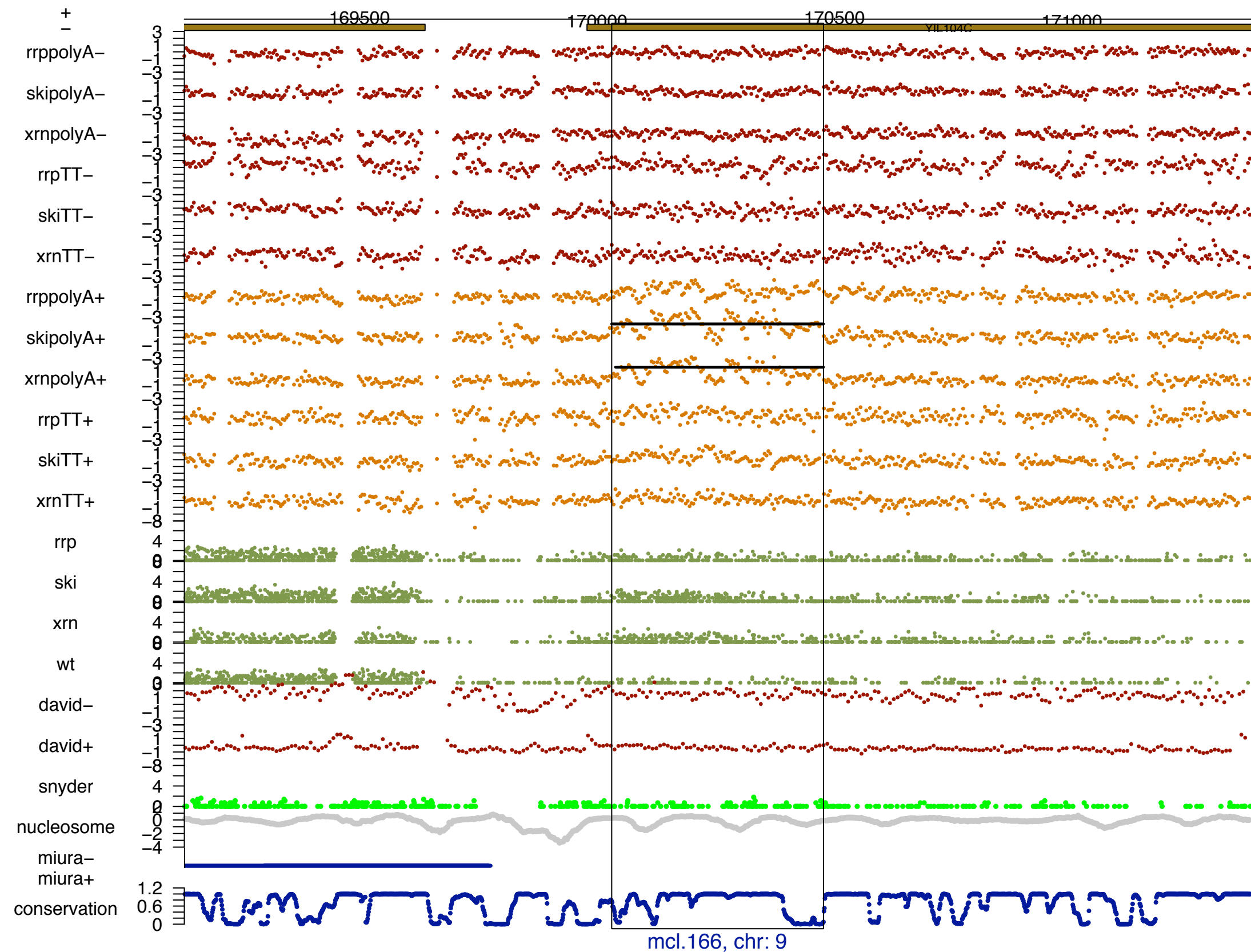

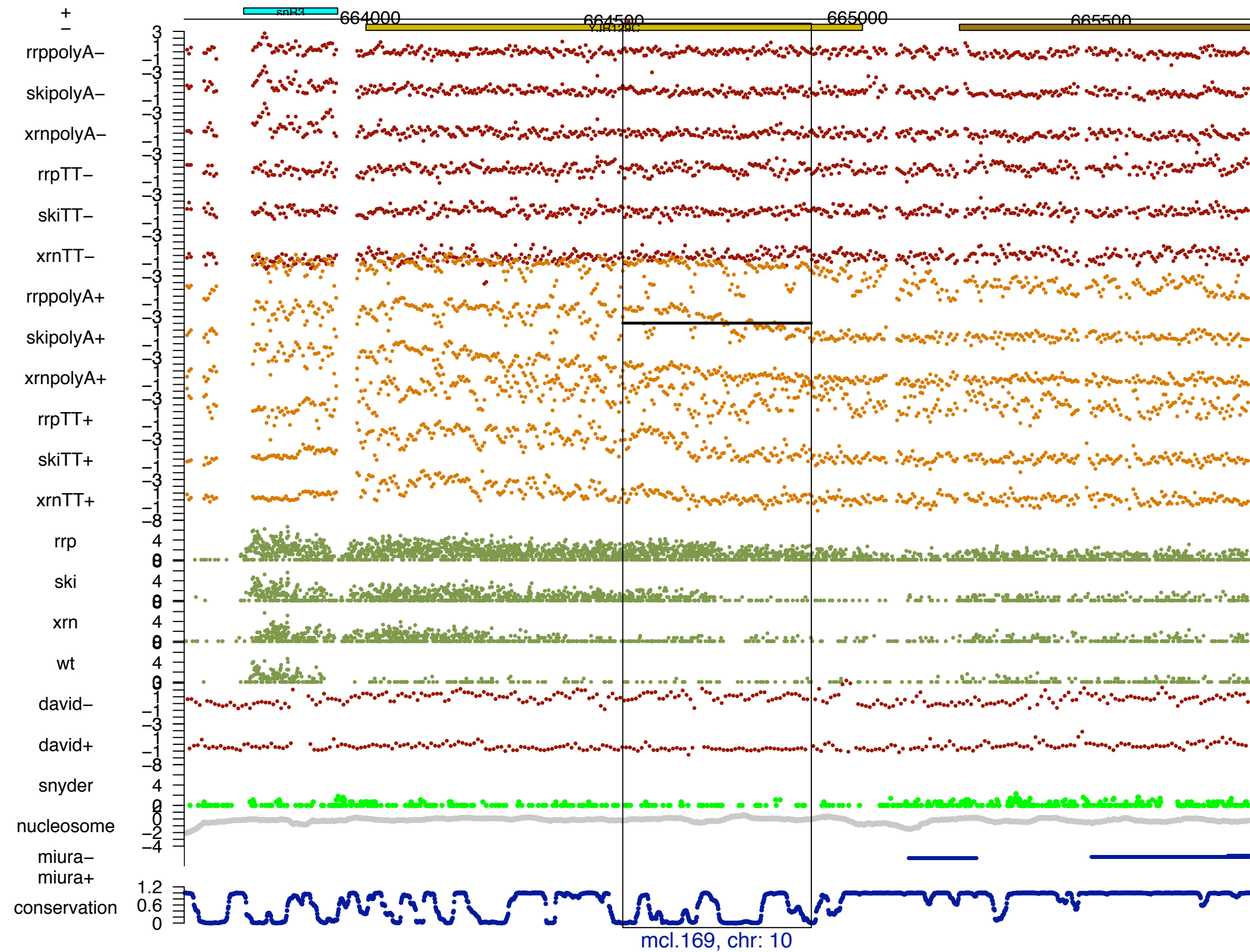

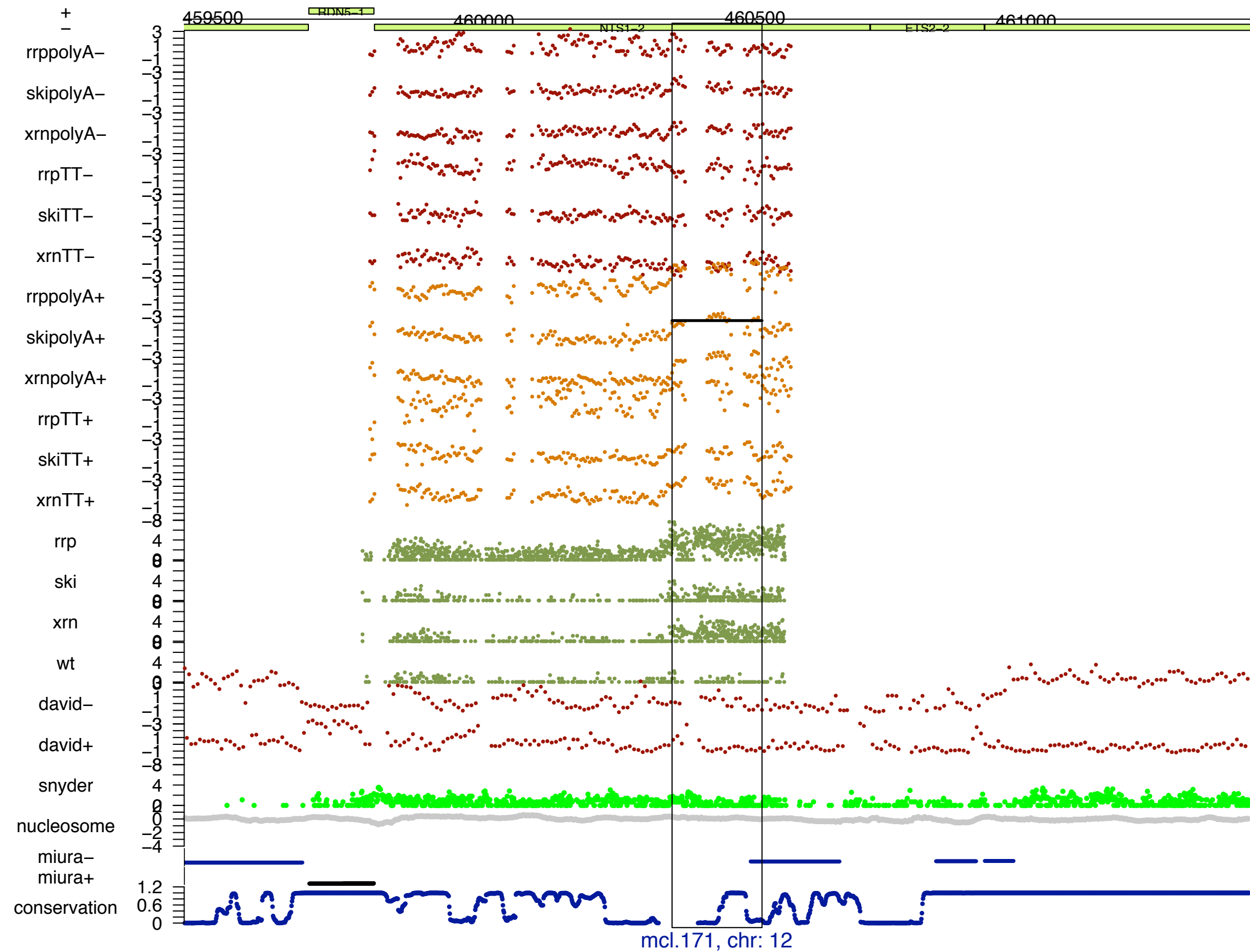

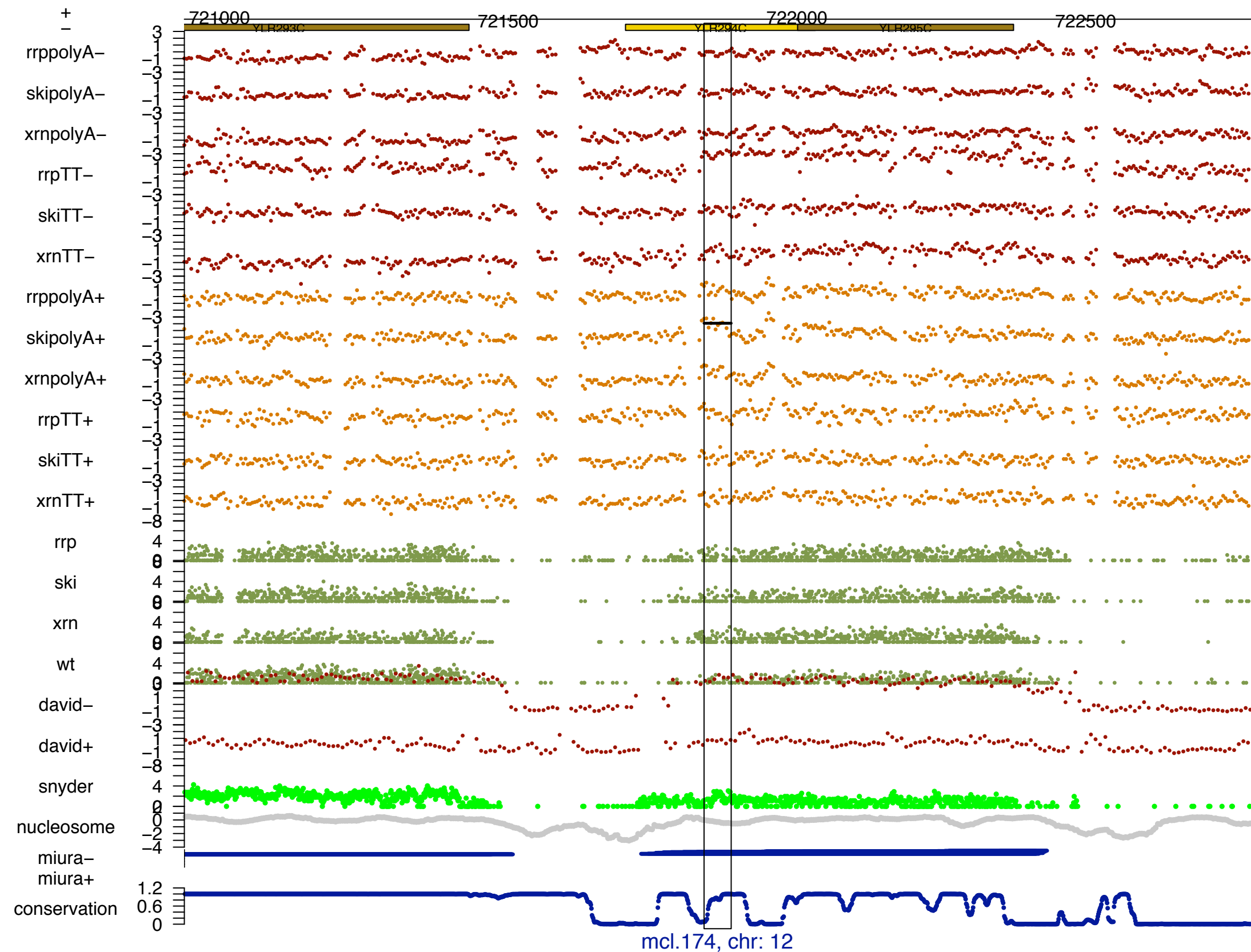

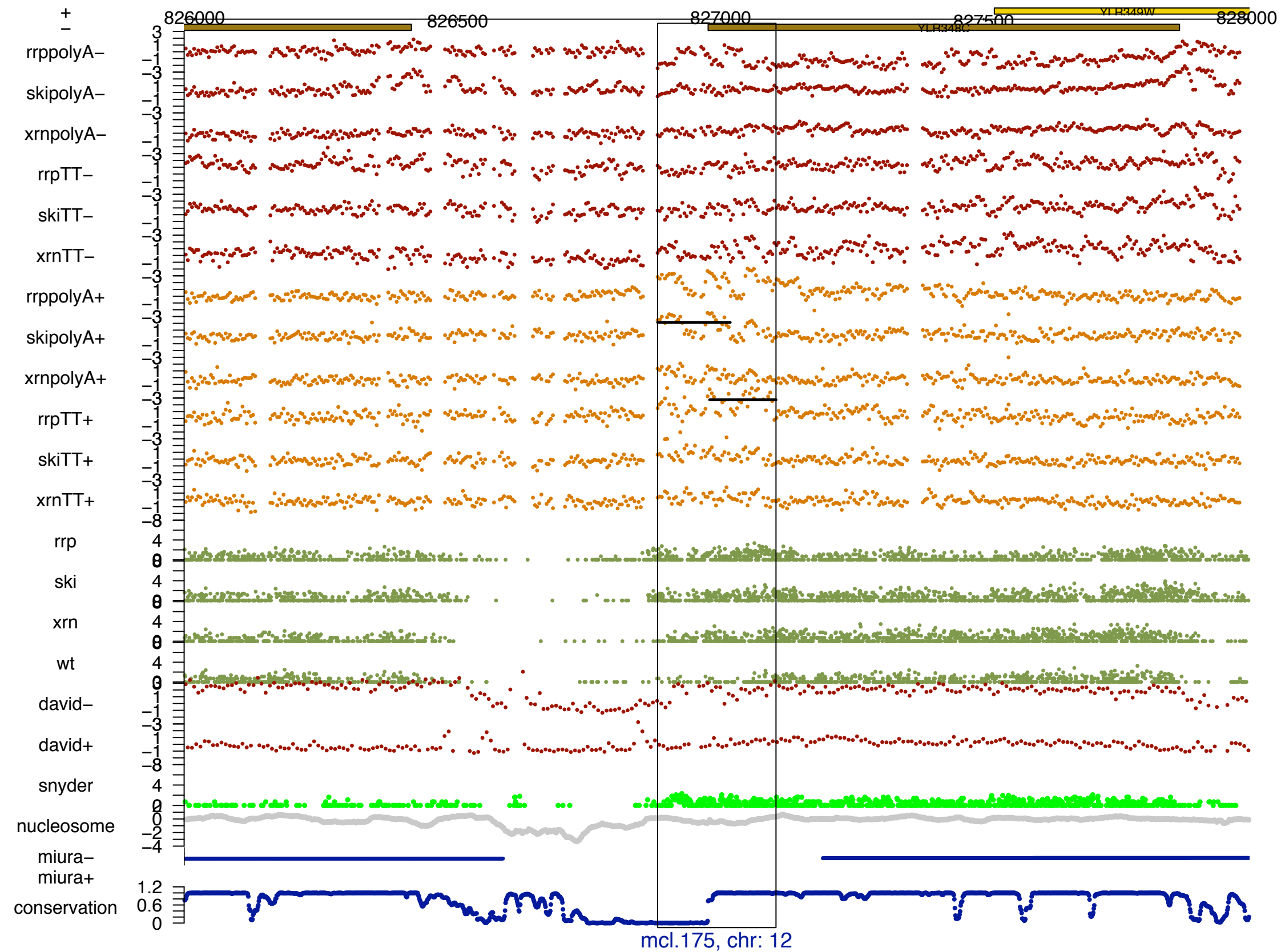

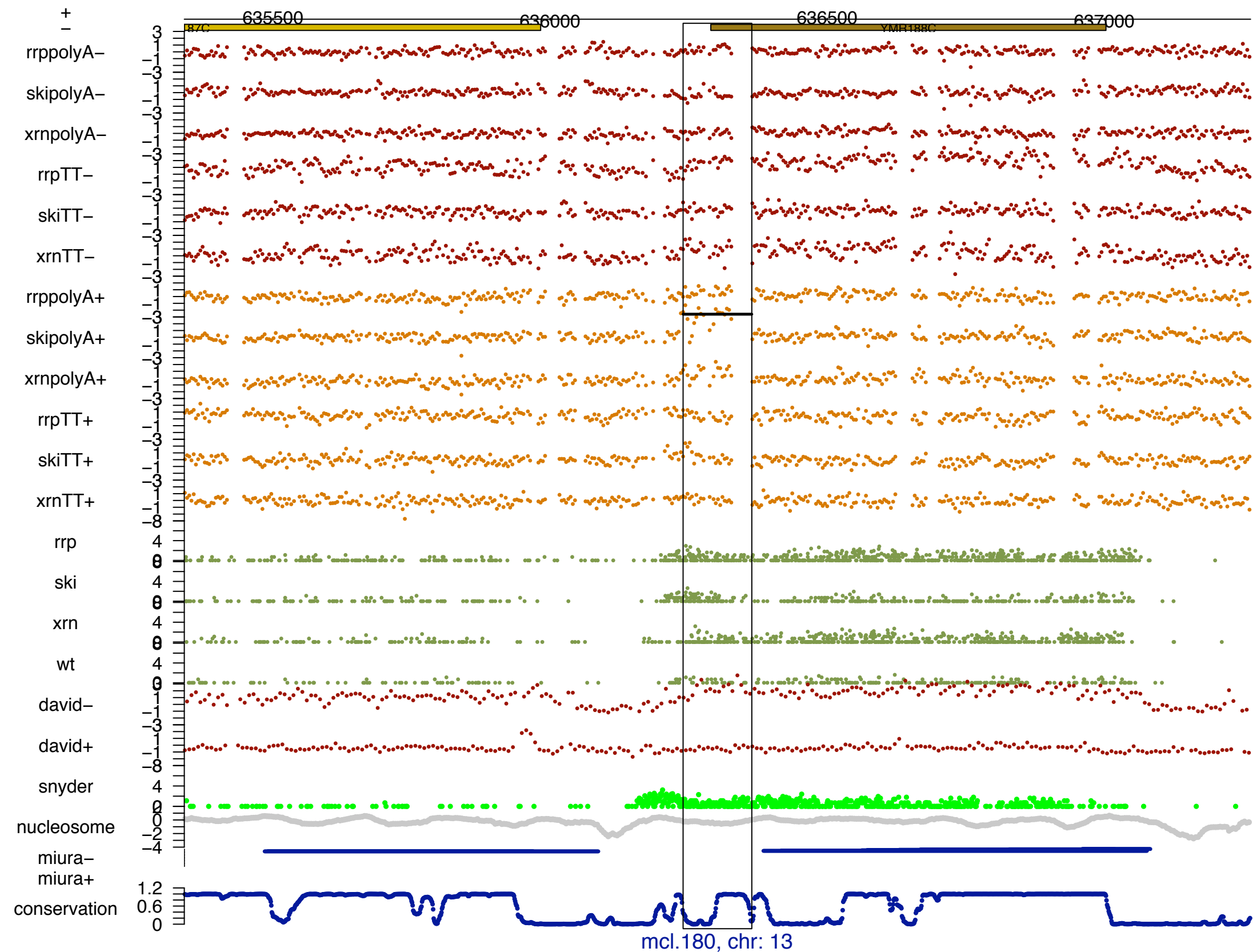

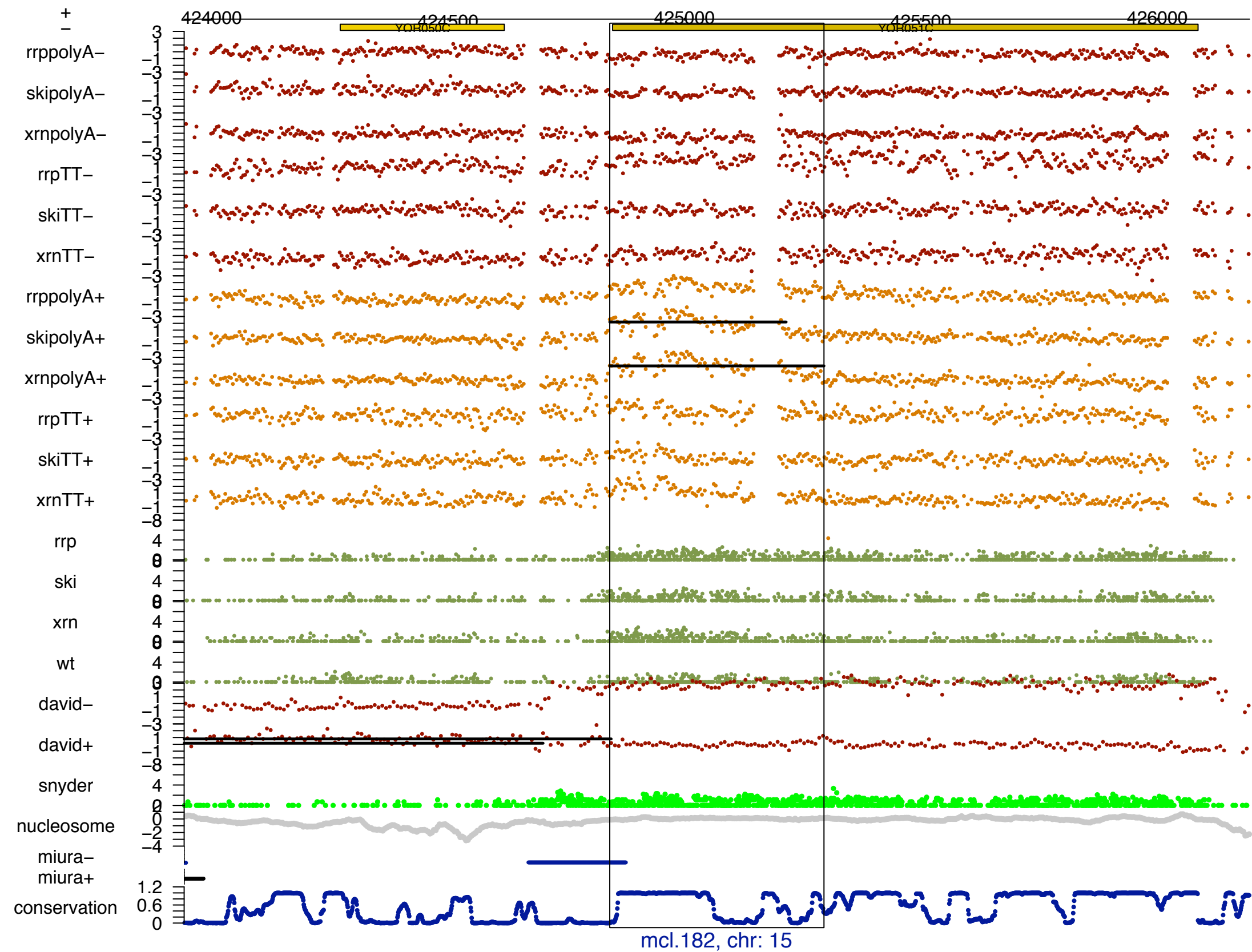

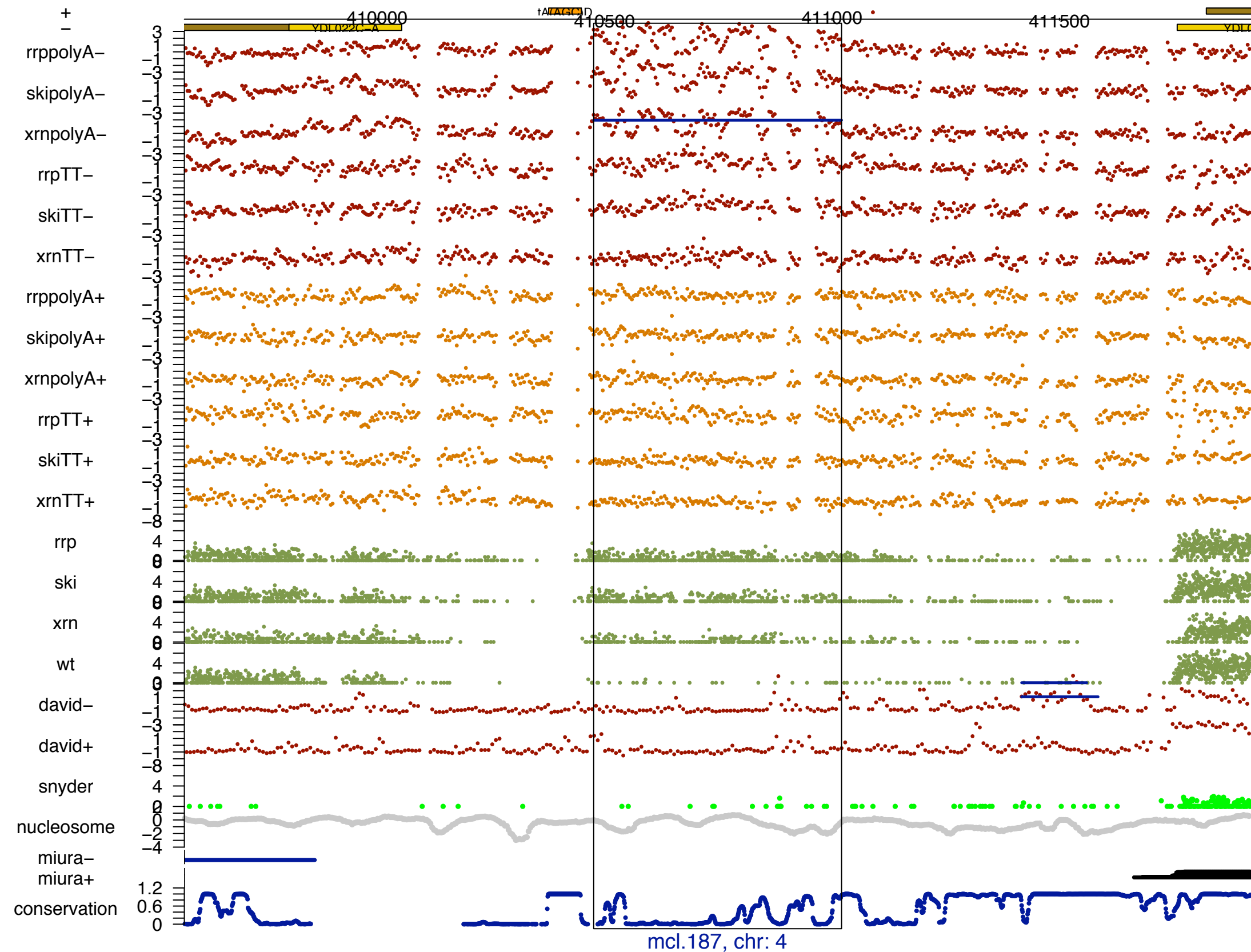

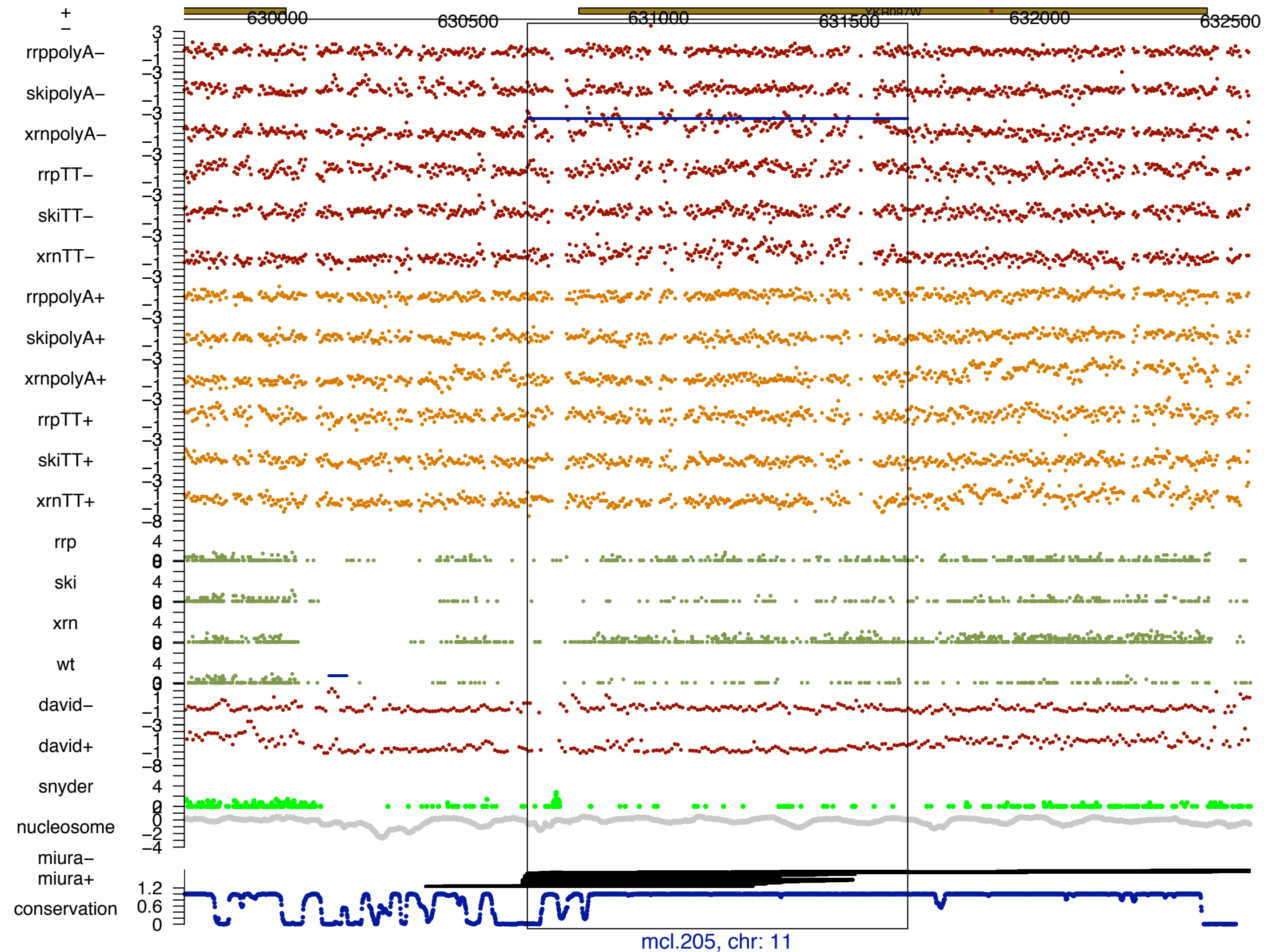

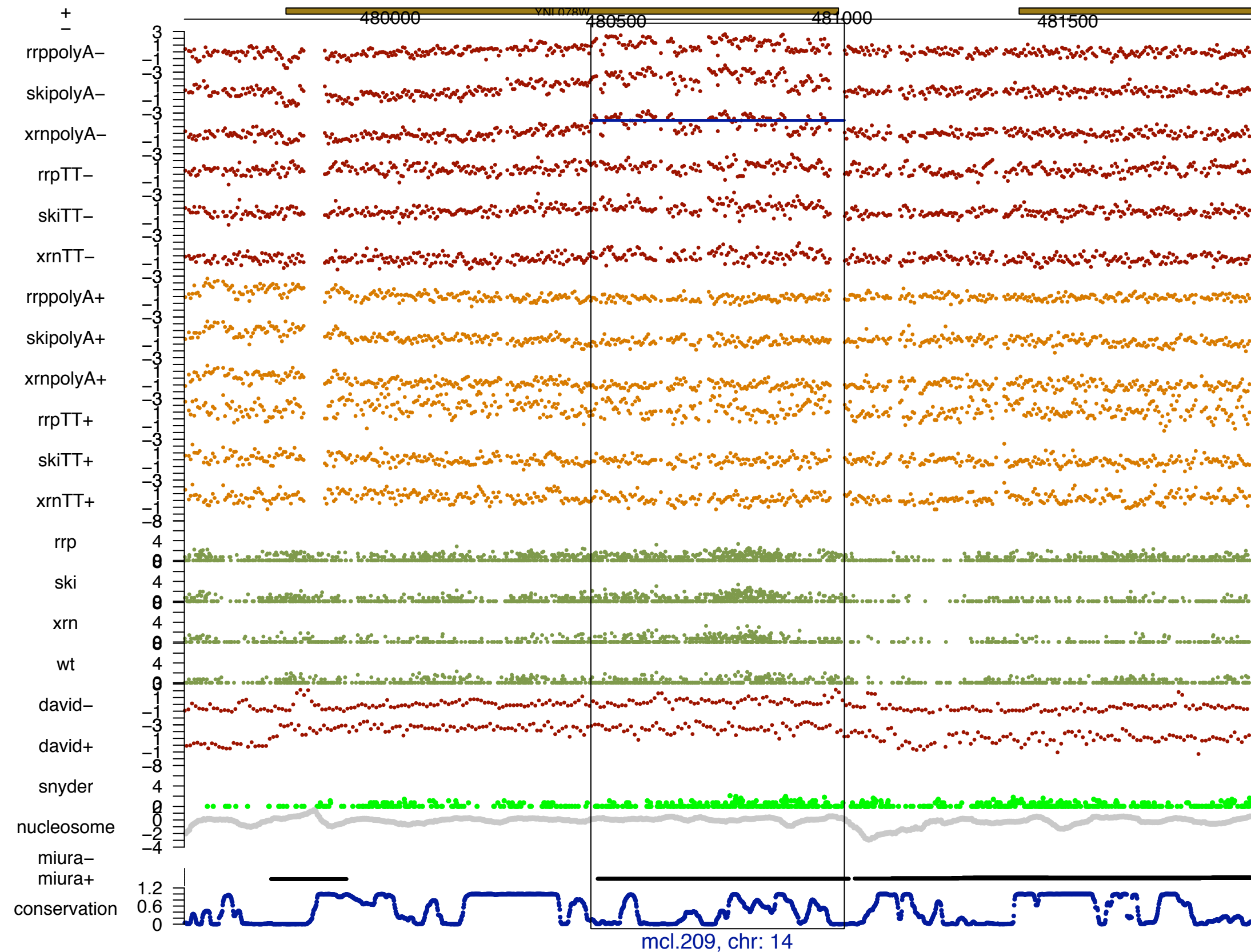

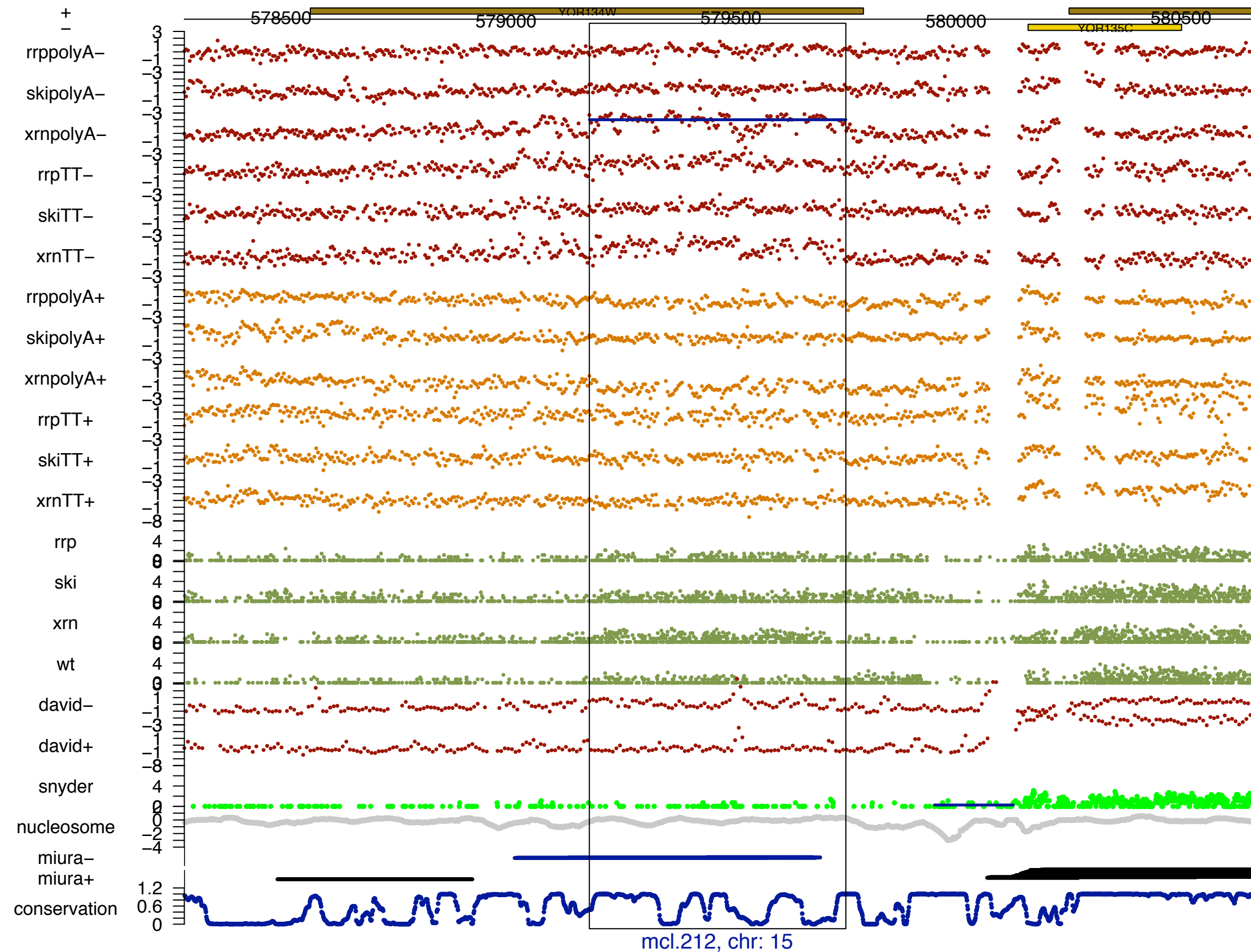

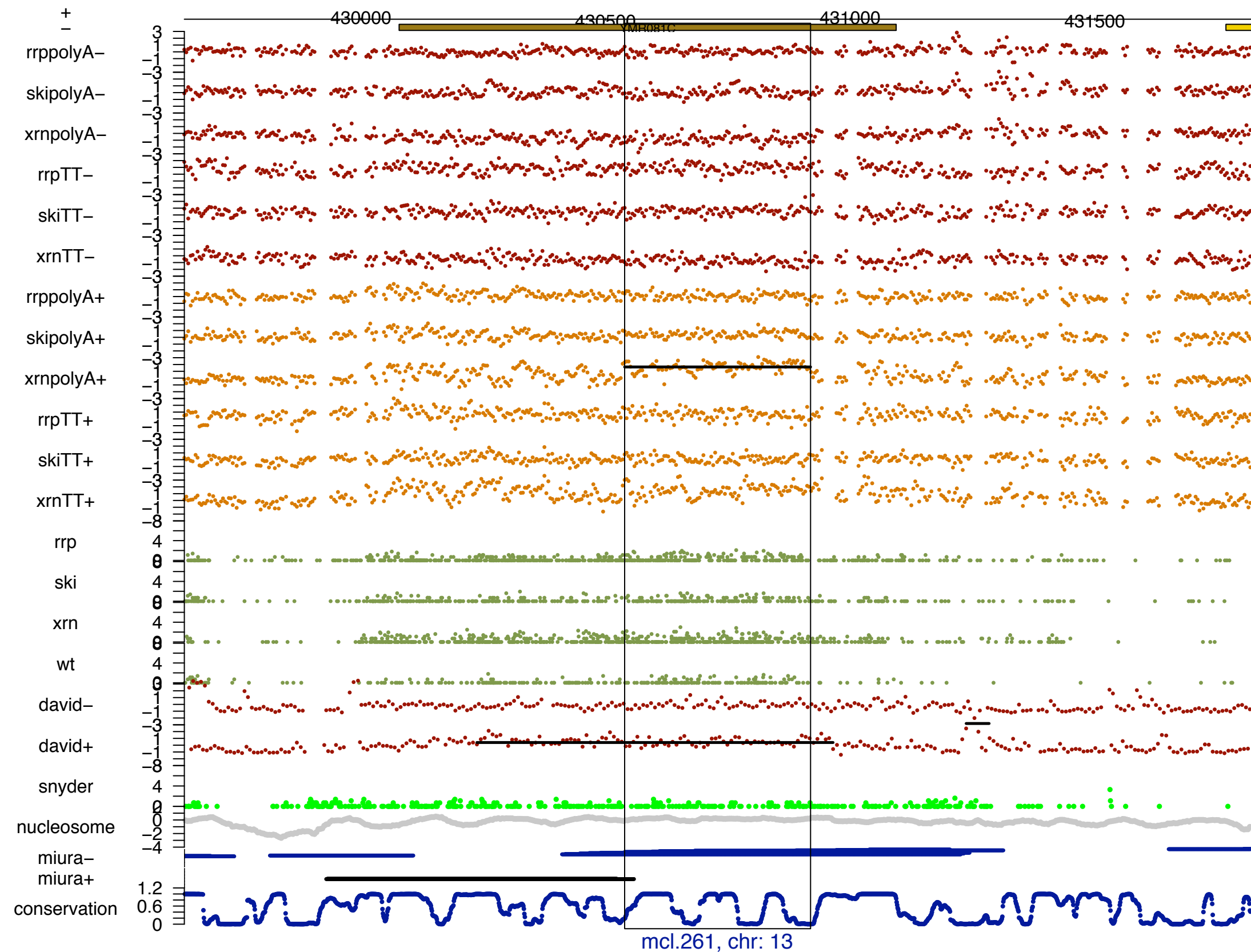

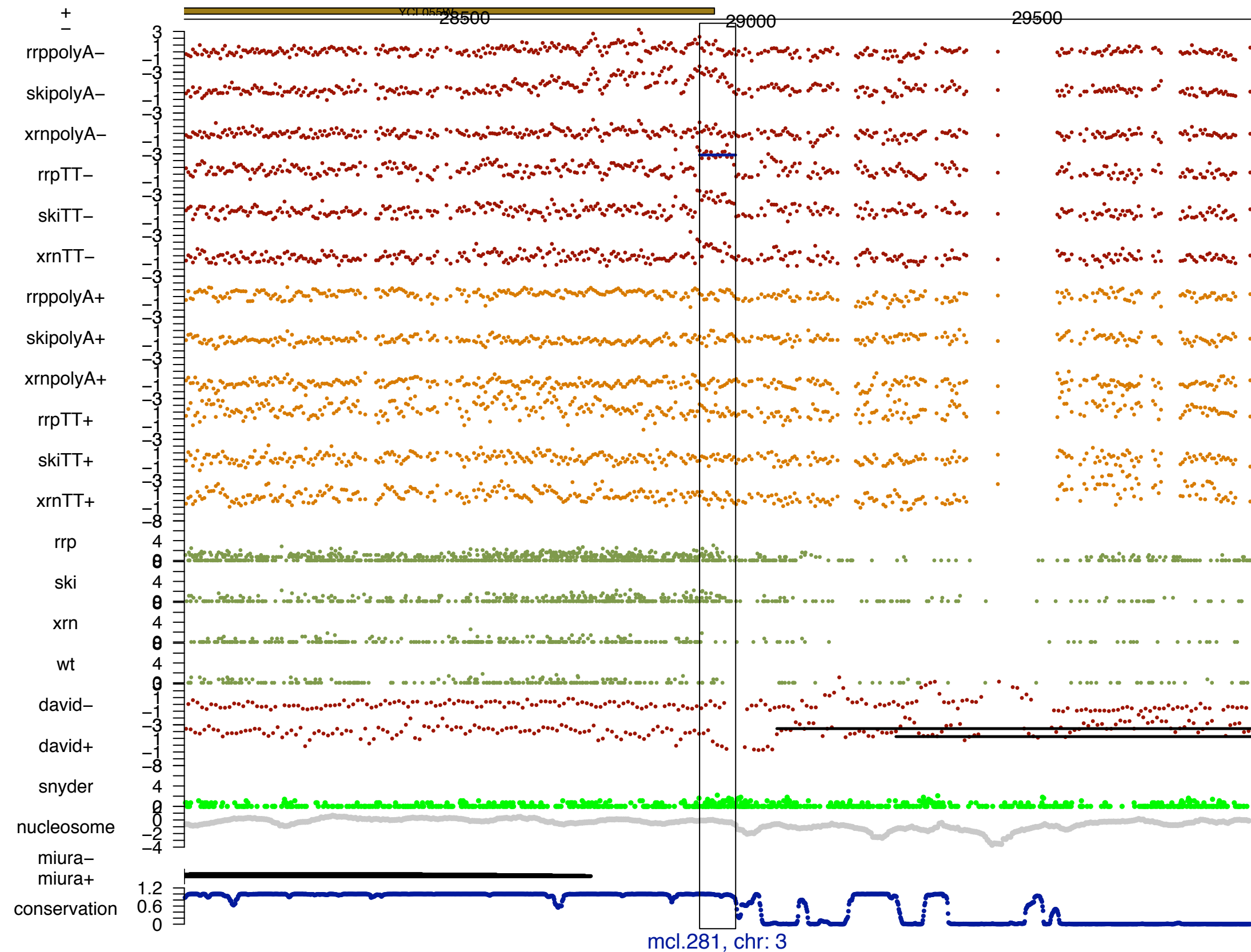

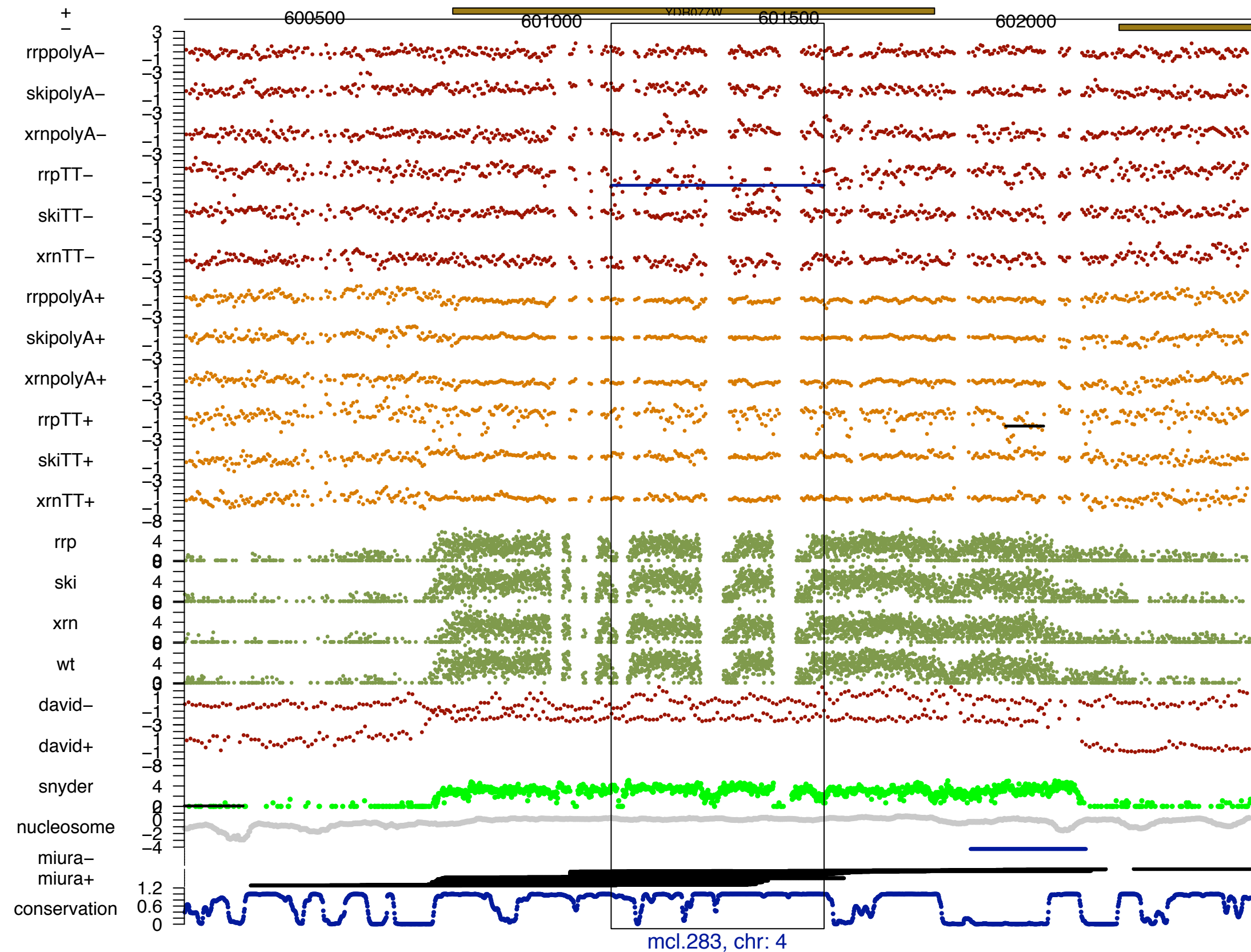

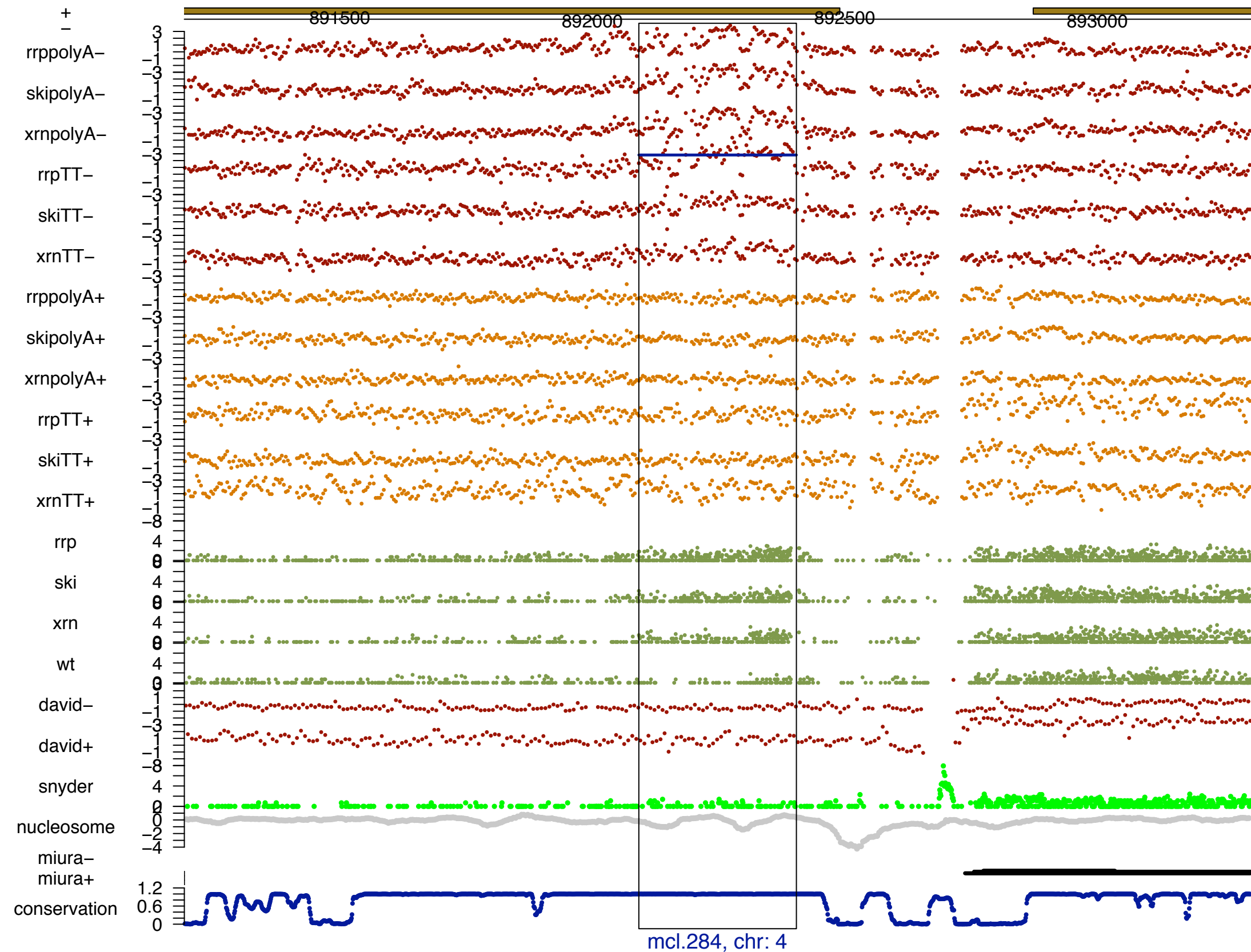

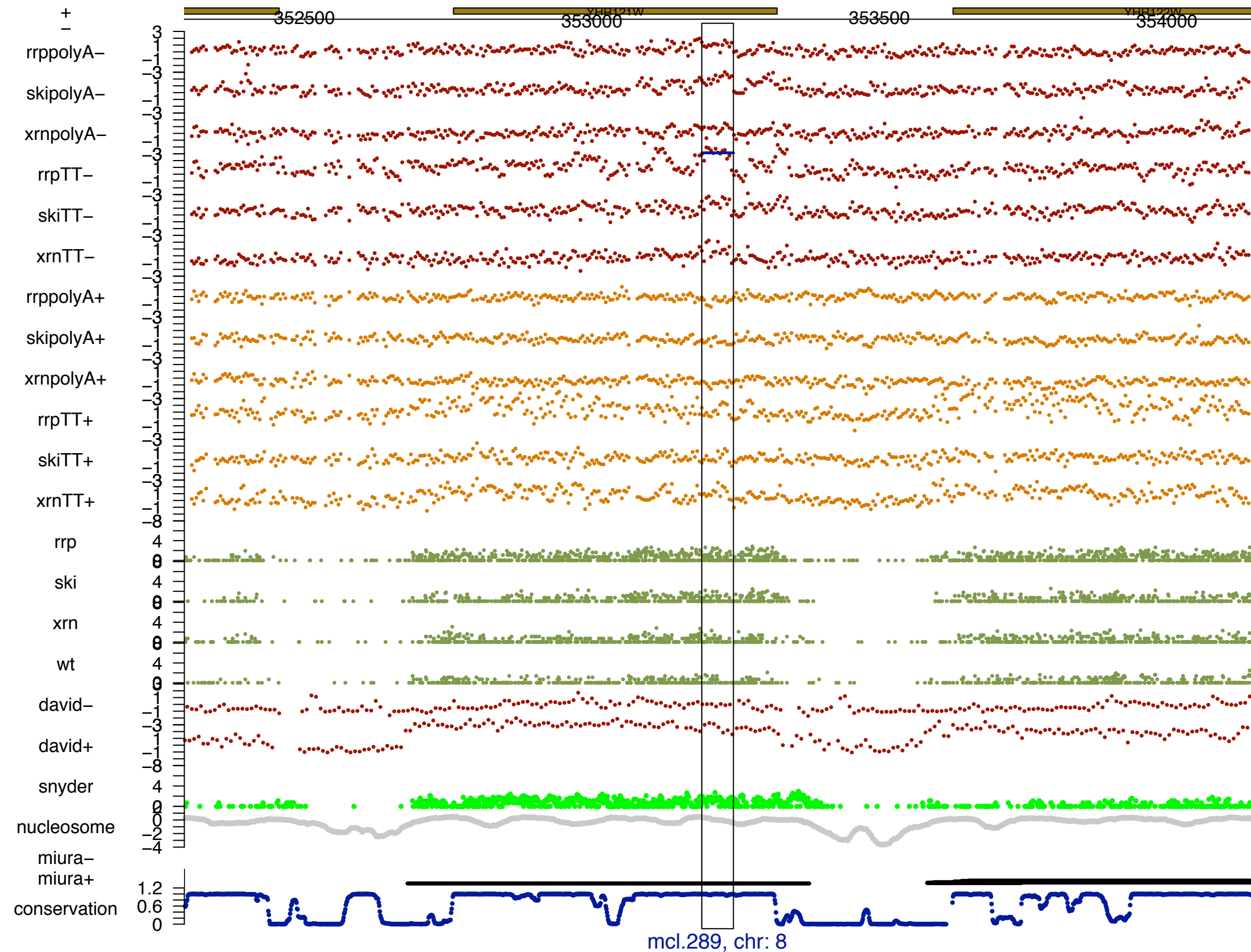

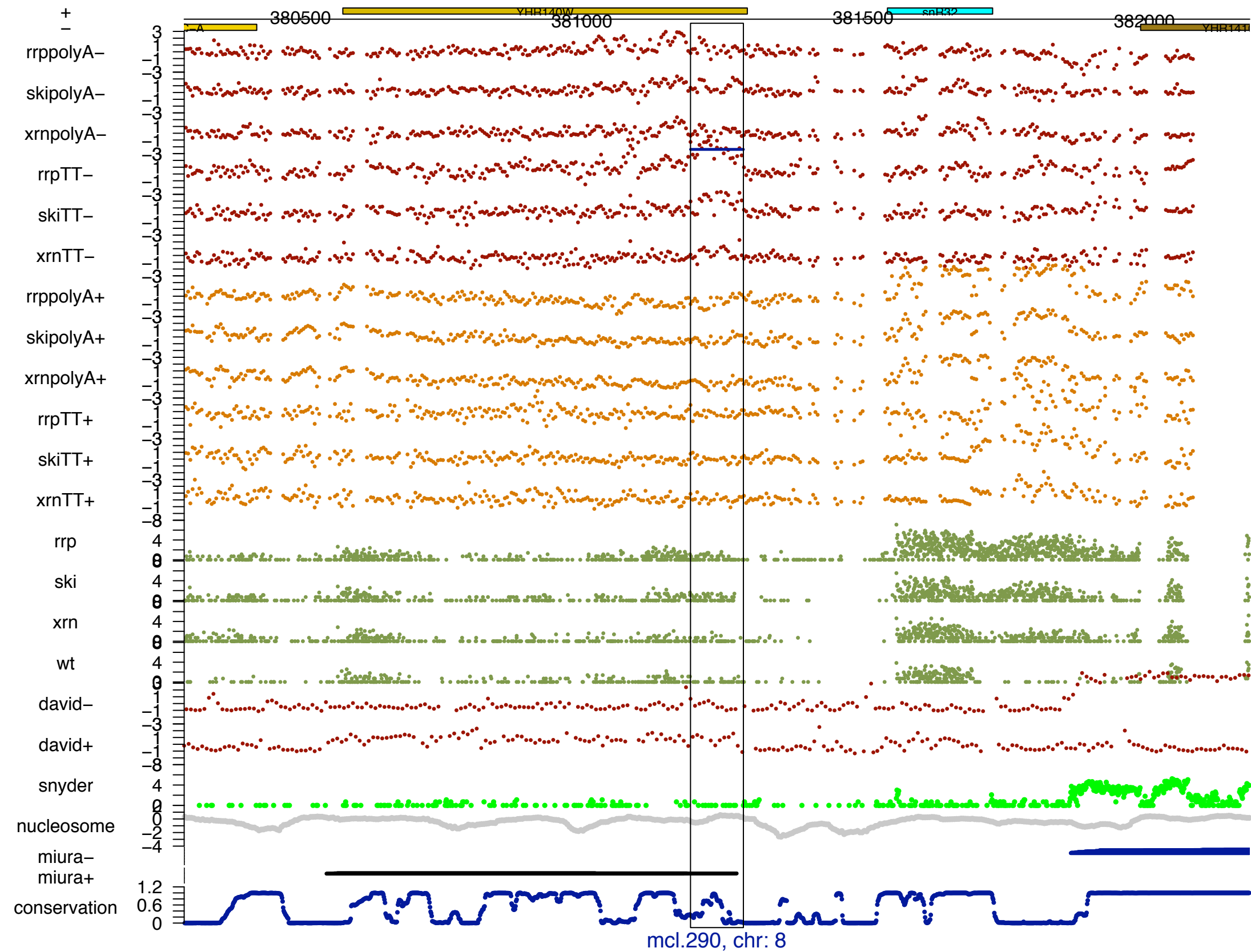

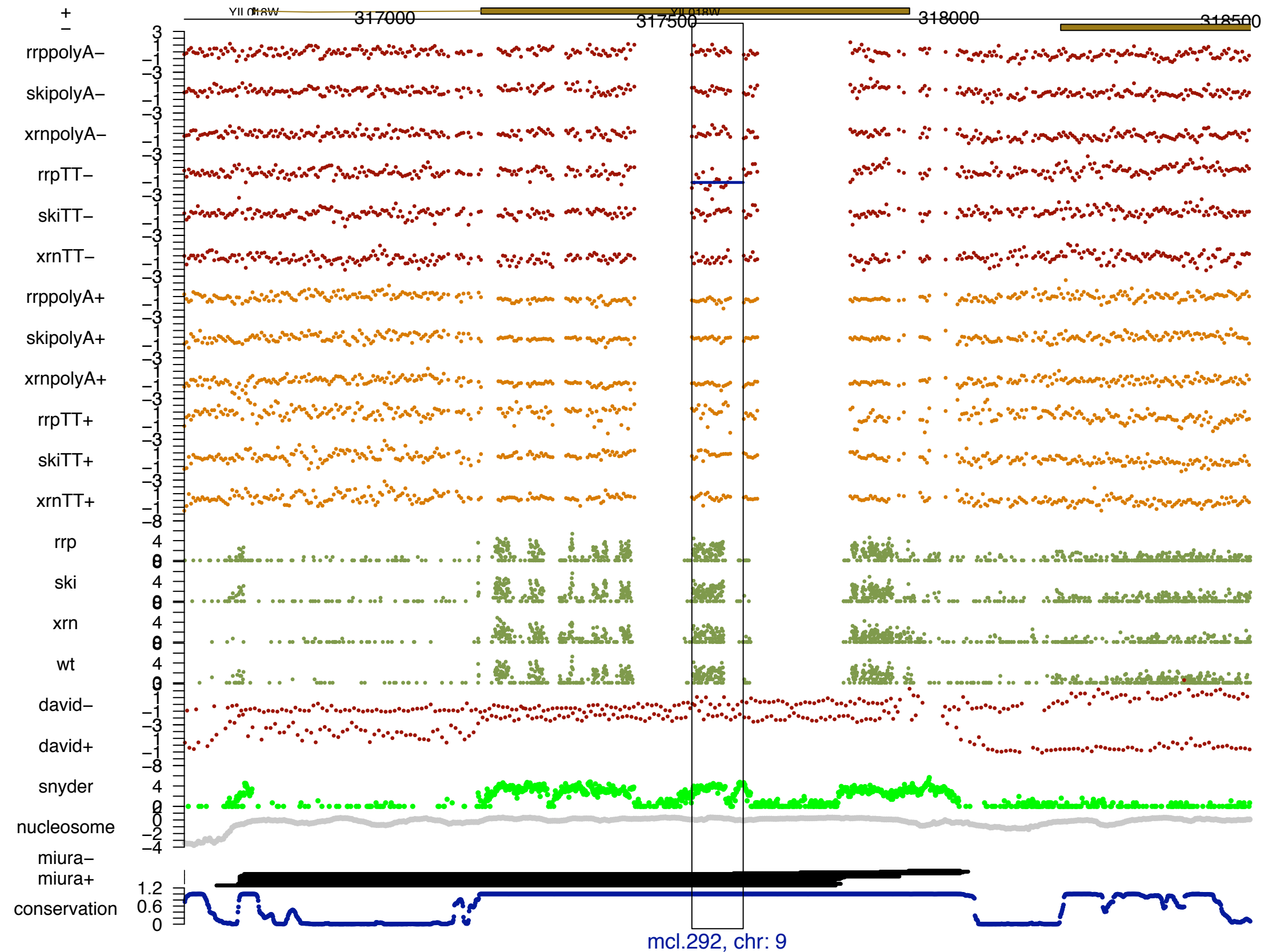

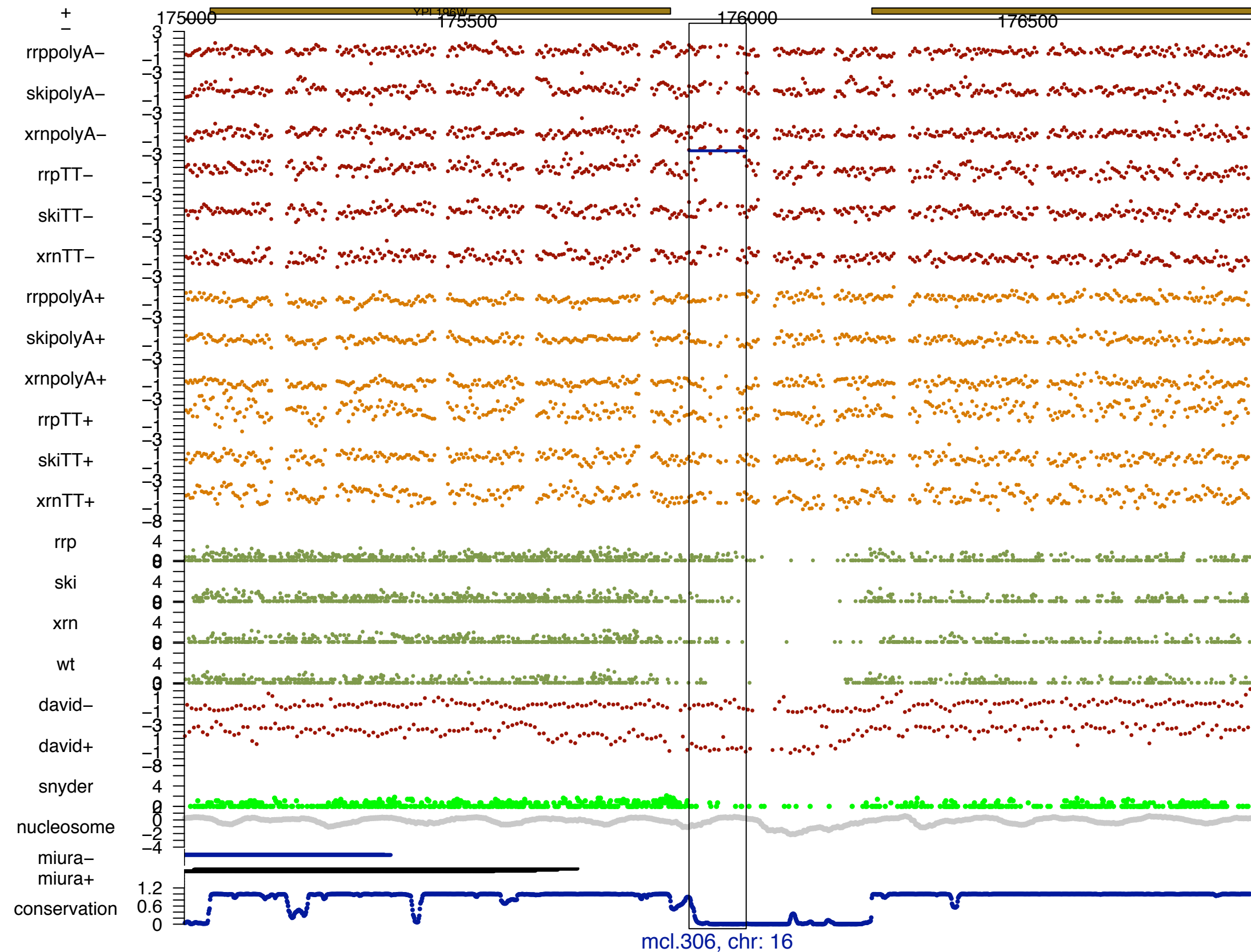

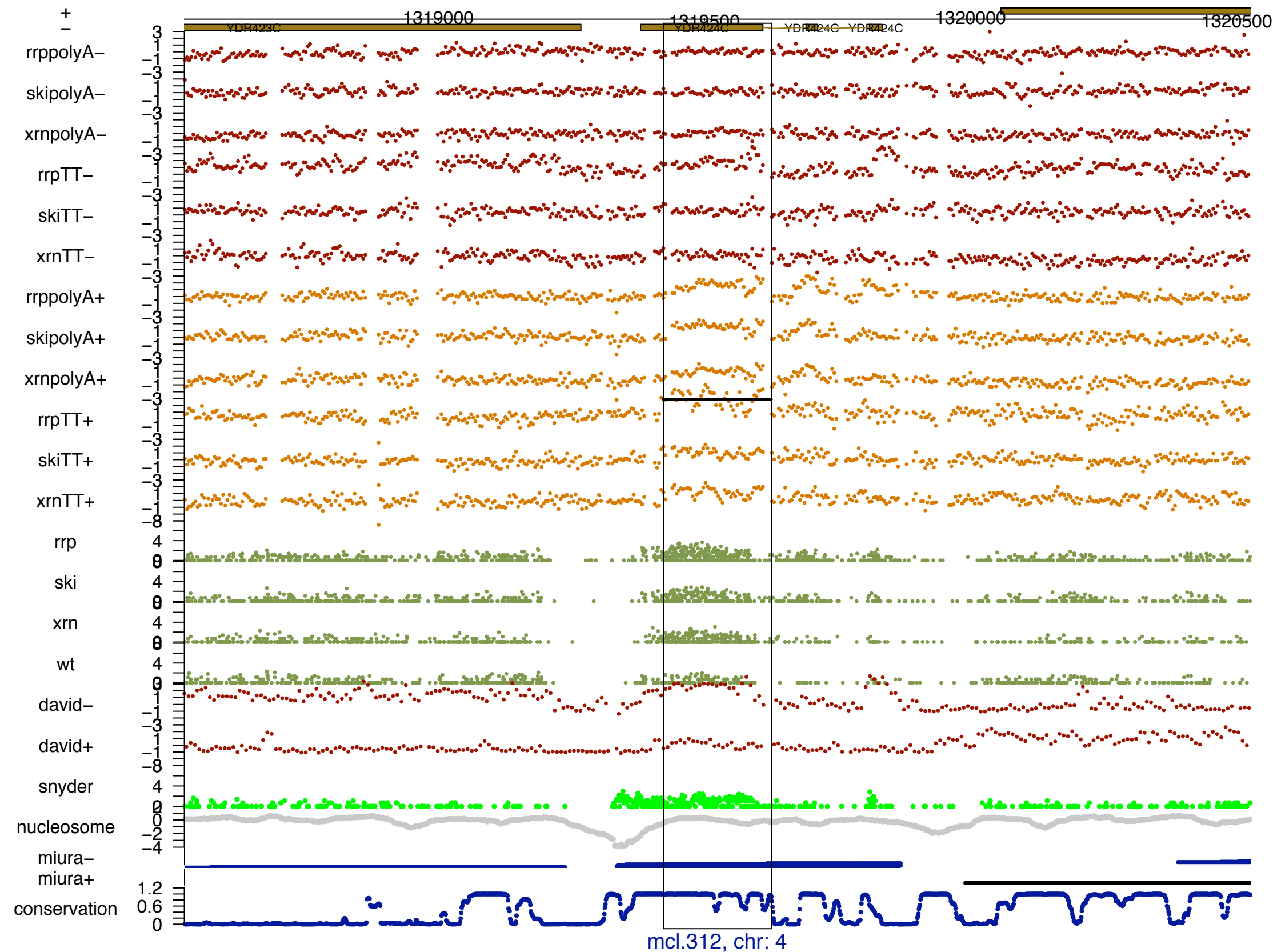

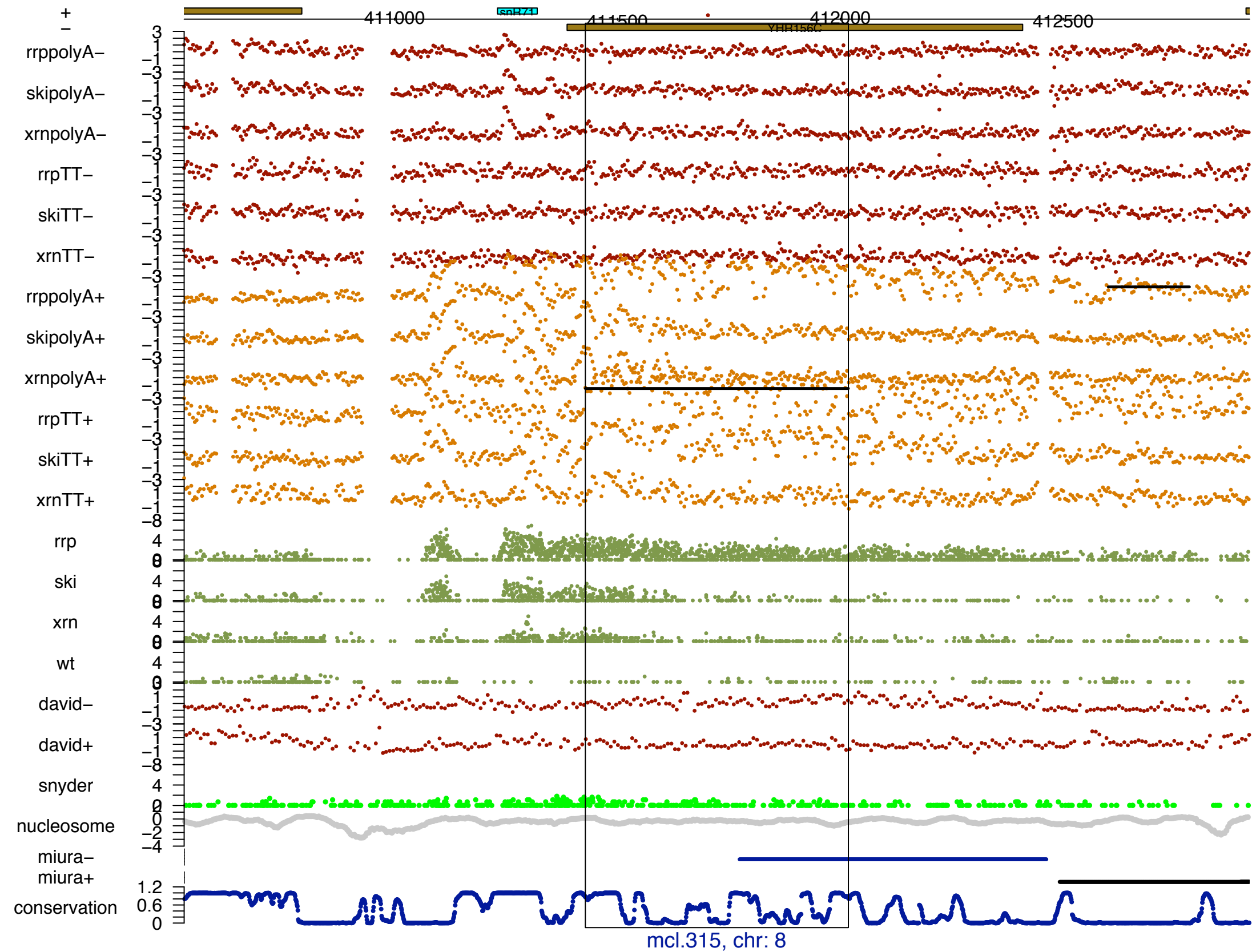

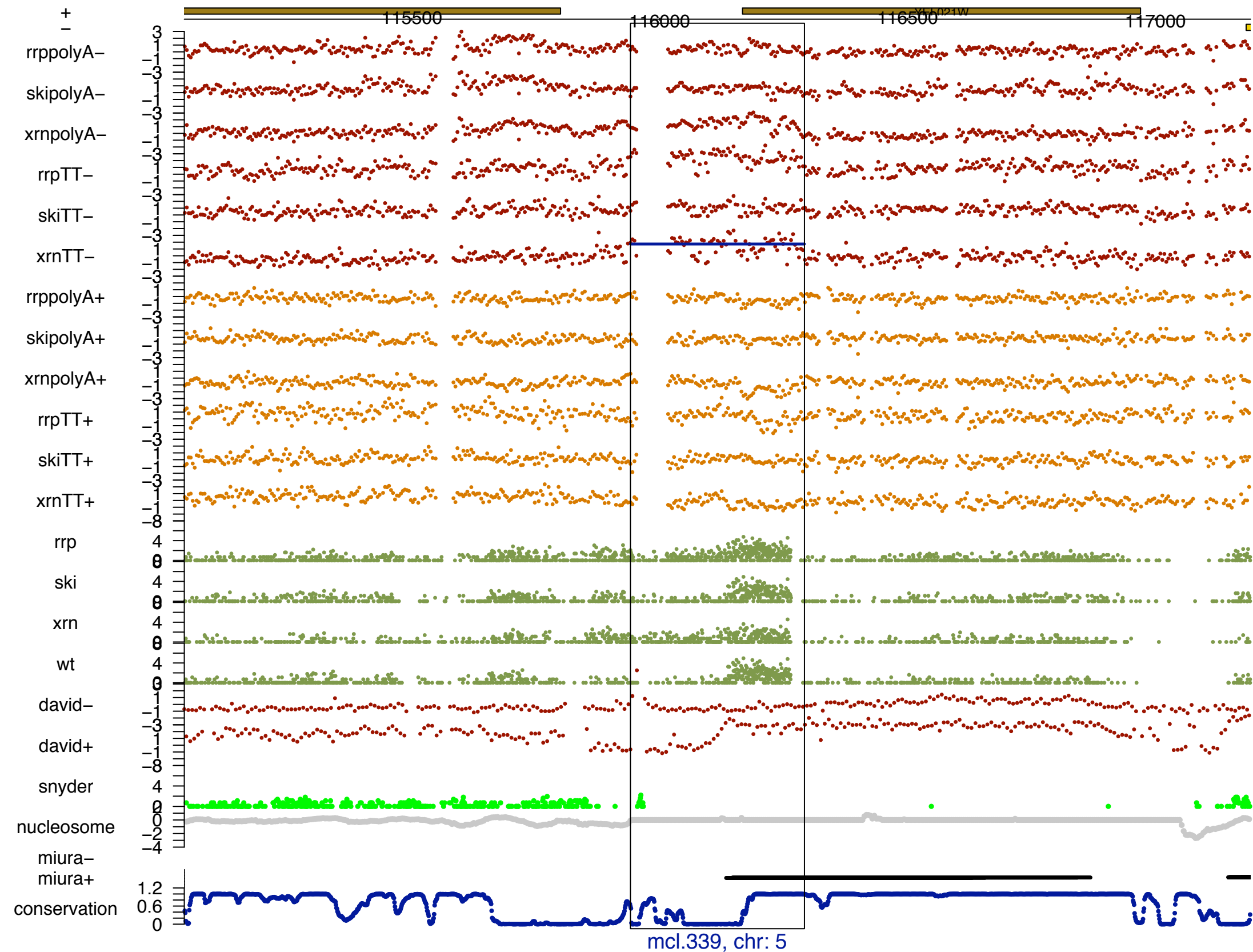

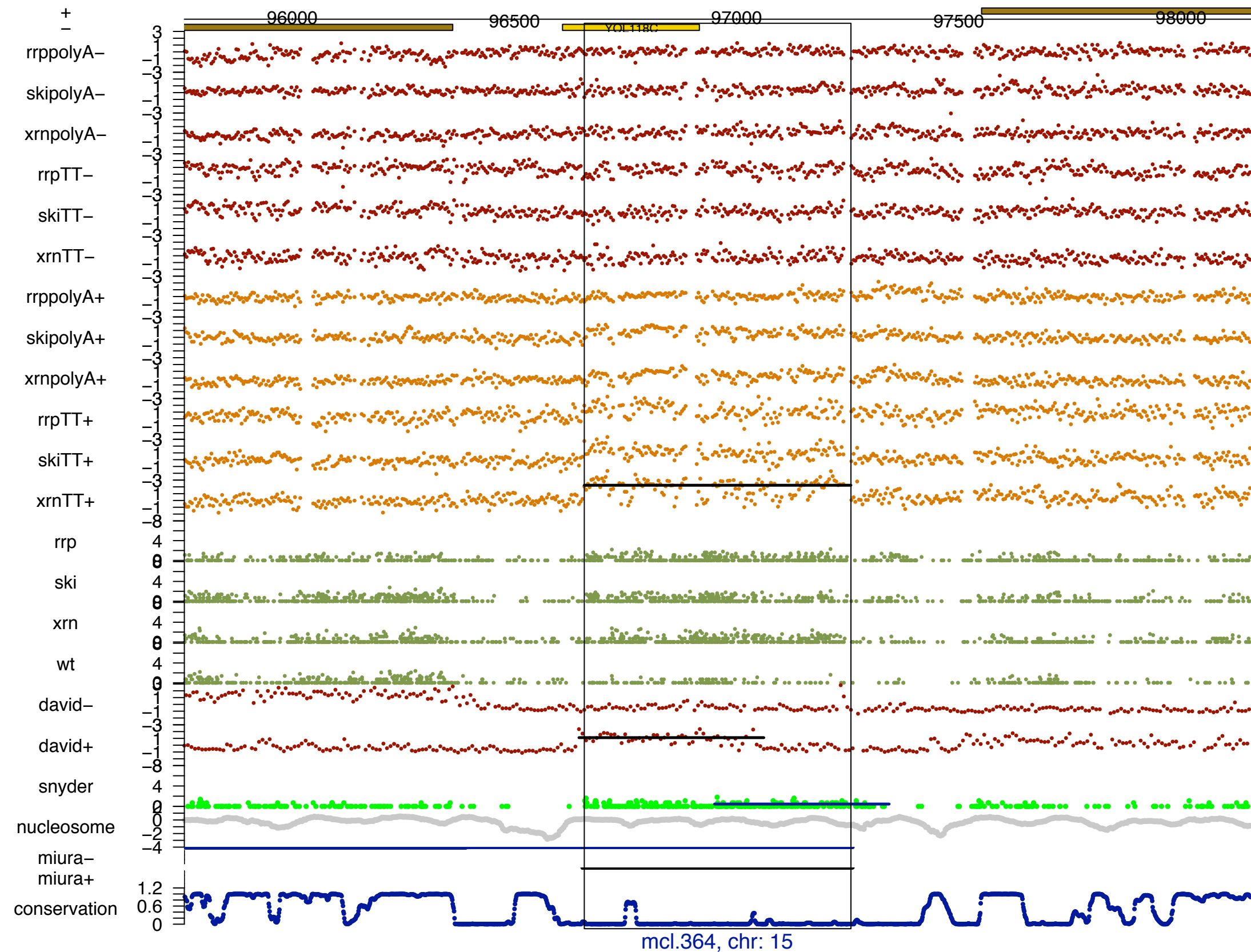

Supplement: Figure S5 — Unannotated non-intergenic transcripts found in this study. Each page shows one transcript, with the following information tracks from top to bottom: SGD annotation on the Watson and Crick strands), our tiling microarray data from the Crick and Watson strands (poly A+ RNA above total RNA), our UHTS data for the mutant and wild-type strains, tiling microarray data from David et al. for the Crick and Watson strands, UHTS data from Nagalakshmi et al., nucleosome position, data from Miura et al., and degree of conservation. The name and chromosome of origin of each transcript are indicated below each panel. For the UHTS data, each point plotted corresponds to the 5′ end of sequence reads, and the y position of the plotted point above the axis indicates (on a log scale) how many reads mapped to that position. Horizontal lines in a track indicate novel segments found in the corresponding study (black for forward strand and blue for reverse strand). (6.83 MB PDF) [file pgen.1000299.s005.pdf]

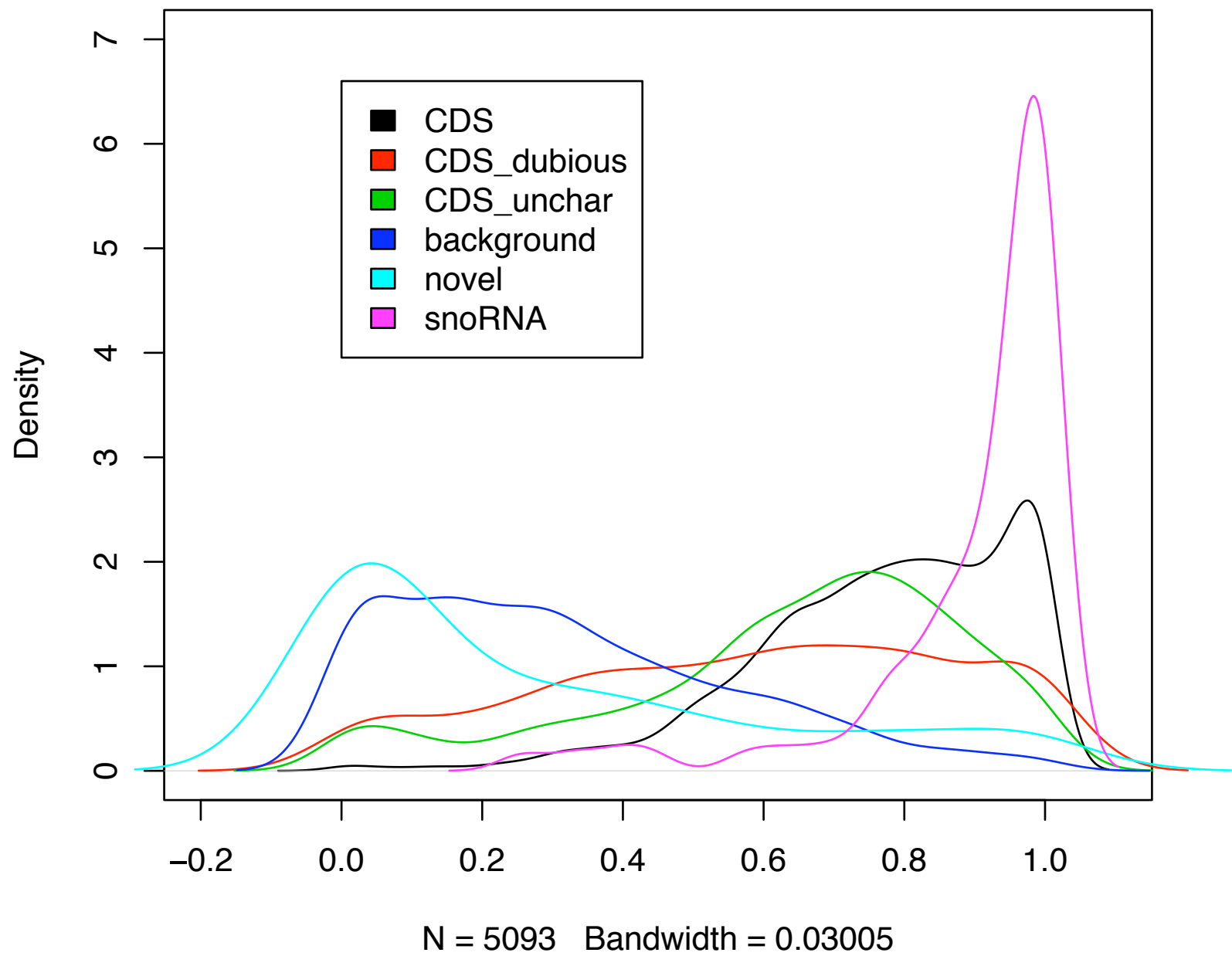

Figure S7

Supplement: Figure S7 — Density plot of conservation scores for different categories of segment. (0.04 MB PDF) [file pgen.1000299.s007.pdf]
