# Supplementary material for: Novel Low Abundance and Transient RNAs in Yeast Revealed by Tiling Microarrays and Ultra High–Throughput Sequencing Are Not Conserved Across Closely Related Yeast Species
Source: PLoS Genet. 2008 Dec 19;4(12):e1000299. doi: 10.1371/journal.pgen.1000299 (PMC2601015; doi:10.1371/journal.pgen.1000299)
Supplement: Text S1 — Supplementary File Descriptions. (0.09 MB DOC) [file pgen.1000299.s011.doc]

**Supplementary File Descriptions**

=========================

**Supplementary Table 1** (TableS1_validated_transcripts.tab): Table of validated transcripts. Here there are 566 rows, each corresponding to an individual cluster. The column metaName groups the clusters together into transcripts, so that there are 365 unique different metaNames. The columns are as follows:

| metaName | The identifier of the meta cluster |
| --- | --- |
| isNovel | Is the meta cluster novel in this study? |
| clusters | The identifier of the clusters in the meta cluster |
| nrsegments | The number of segments joined together to form the cluster |
| mutname | the name of the mutant strain |
| chr | chromosome |
| strand | strand |
| start | start position |
| end | end position |
| length | length, when taking non-unique bases into account |
| lengthWithoutMask | end - start |
| nrprobes | number of microarray probes in the cluster |
| regulation | is the cluster ``up'' or ``down regulated. |
| inAnno | is the cluster wholy in SGD annotation |
| isIntergenic | Is the cluster intergenic |
| overlapsAnno | Does the cluster overlap SGD annotation |
| hasOverlapDavid | Does the cluster overlaps a segment from David et al |
| hasOverlapSnyder | Does the cluster overlaps a segment from Nagalakshmi et al |
| hasOverlapMiura | Does the cluster overlaps a segment from Miura et al |
| hasOverlapAres | Does the cluster overlaps a segment from Davis and Ares |
| validated | has the cluster validated? |
| marraymmomed | The median fold-change for the microarray, corrected by the overall array median fold-change |
| marraymedian | The median fold-change for the microarray |
| highExpAndNoOppositeStrand | Is the cluster ``highly'' expressed with low expression on the opposite strand |
| oppositeStrandMarraymedian | The median fold-change for the microarray for the other strand region corresponding to the cluster |
| oppositeStrandMarraymmomed | The median fold-change for the microarray for the other strand region corresponding to the cluster, corrected for the overall array median fold change |
| diffExpWT | The t-statistic for the test for differential expression in the mutant vs. the wild-type, for the sequence data |
| isDiffExpWT | Is the cluster differentially expressed in the sequence data relative to the wild-type. |
| seqCounts | The number of reads mapped to the cluster in the mutant experiment |
| seqCountsWT | The number of reads mapped to the cluster in the wild-type experiment |
| lambda | The lambda of the cluster (number of reads per base, corrected for lane counts) |
| lambdaWT | The lambda for the wild-type experiment for the cluster |
| background5 | Is the cluster detected at background 5% level |
| background20 | Is the cluster detected at background 20% level |
| introns5 | Is the cluster detected at intron 5% level |
| introns20 | Is the cluster detected at intron 20% level |
| diffExpRegion | The t-statistic for test for differential expression in the cluster vs. its up- and down-region |
| isDiffExpRegion | Is the cluster differentially expressed compared to its up- and down region |
| upLength | Length of the up-region using only unique bases. |
| seqCountsUp | Number of reads in the up-region |
| downLength | Length of the down-region using only unique bases. |
| seqCountsDown | Number of reads in the down-region |

**Supplementary Table 2** (TableS2_detection_of_other_studies.tab):

This table shows which previously reported unannotated transcripts were above background level in this study (use the column background20, with TRUE meaning that the transcript was detected). The columns are as follows:

| **column** | **Description** |
| --- | --- |
| chr | Chromosome of the unannotated transcript |
| start | Start of the unannotated transcript |
| end | End of the unannotated transcript |
| strand | Strand of the unannotated transcript |
| lengthWithoutMask | Length of the unannotated transcript |
| length | Length of the unannotated transcript, taking unmappable bases into account |
| RPKMmask | RPKM for the unannotated transcript in the relevant experiment, taking unmappable bases into account using length |
| RPKM | RPKM for the unannotated transcript in the relevant experiment, using lengthWithoutMask |
| background5 | Is the unannotated transcript above background at a 5% cutoff |
| background20 | Is the unannotated transcript above background at a 20% cutoff (used in the paper) |
| reads | The number of reads in the unannotated transcript in the relevant experiment |
| study | Which study did the unannotated transcript originate from. |

We obtained the novel transcripts from David et al., Nagalakshmi et al., Miura et al., and Davis and Ares in the following manner:

**David et al.**

For David we took Supplementary table 3, and 4 and kept all transcripts that had one of the following values in the "category"

column :

"unannotated antisense - filtered" or

"unannotated isolated - filtered".

**Nagalakshmi et al.**

We obtained a gbrowse track from the URL

http://www.yale.edu/snyder/Naga2008sup/novel_annotations.track

We only included unannotated transcripts from chromosome 1 to 16.

**Miura et al.**

We obtained their "Supporting Table 1: 51,026 cDNA clones analyzed in this study". This table includes all clones hence also clones corresponding to annotated regions. Everything was kept. These clones were used to compute the overlap between any cluster which we had validated as well as used to define background regions.

In addition, we downloaded their Supporting Table 8: 667 Novel regions. This table was used only in the final section of the results when we validated Miura et al.'s segments using our sequencing data. These are the relevant segments for table S2.

**Davis and Ares**

We obtained their Supplemental Table 1, having 13143 rows including header. We were unable to find a column clearly indicating what transcripts they considered novel, so we replicated their analysis. We determined the overall Average LogRatio to be 0.3 and the overall standard deviation to be 1. We then selected transcripts with a "Average.LogRatio" > 0.3 + 1. This selecttion was futher subselected by only keeping transcripts with "i" in their ID. The result of this was a set of novel unannoated transcript slightly larger (about 10) than the set reported in the paper.

**Supplementary Table 3** (TableS3_feature_rpkm.tab):

Table of RPKMs for our different datasets for SGD annotated features. The columns are as follows:

| **Column** | **Description** |
| --- | --- |
| chr | Chromosome of the feature |
| strand | Strand of the feature |
| start | Start of the feature |
| end | End of the feature |
| name | Name of the feature |
| feature | Feature type (CDS = Verified CDS, CDS_dubious = dubious CDS, CDS_unchar = uncharacterized CDS as well as various RNAs) |
| orf_classification | The ORF classification |
| gene | The common name of the feature |
| lengthWithoutMask | Length of the feature |
| length | Length of the feature with unmappable bases removed |
| wt.reads | Number of reads in the feature in the wild-type experiment |
| rrp.reads | Number of reads in the feature in the rrp experiment |
| ski.reads | Number of reads in the feature in the ski experiment |
| xrn.reads | Number of reads in the feature in the xrn experiment |
| wt.rpkm | RPKM for the feature in the wild-type experiment, using lengthWithoutMask as length |
| rrp.rpkm | RPKM for the feature in the rrp experiment, using lengthWithoutMask as length |
| ski.rpkm | RPKM for the feature in the ski experiment, using lengthWithoutMask as length |
| xrn.rpkm | RPKM for the feature in the xrn experiment, using lengthWithoutMask as length |
| wt.rpkmMask | RPKM for the feature in the wild-type experiment, using length as length, thus taking the unmappable bases into account |
| rrp.rpkmMask | RPKM for the feature in the rrp experiment, using length as length, thus taking the unmappable bases into account |
| ski.rpkmMask | RPKM for the feature in the ski experiment, using length as length, thus taking the unmappable bases into account |
| xrn.rpkmMask | RPKM for the feature in the xrn experiment, using length as length, thus taking the unmappable bases into account |
