# Supplementary material for: Novel Low Abundance and Transient RNAs in Yeast Revealed by Tiling Microarrays and Ultra High–Throughput Sequencing Are Not Conserved Across Closely Related Yeast Species
Source: PLoS Genet. 2008 Dec 19;4(12):e1000299. doi: 10.1371/journal.pgen.1000299 (PMC2601015; doi:10.1371/journal.pgen.1000299)
Supplement: Figure S6 — As in Figure S5, but for intergenic transcripts. (13.33 MB PDF) [file pgen.1000299.s006.pdf]

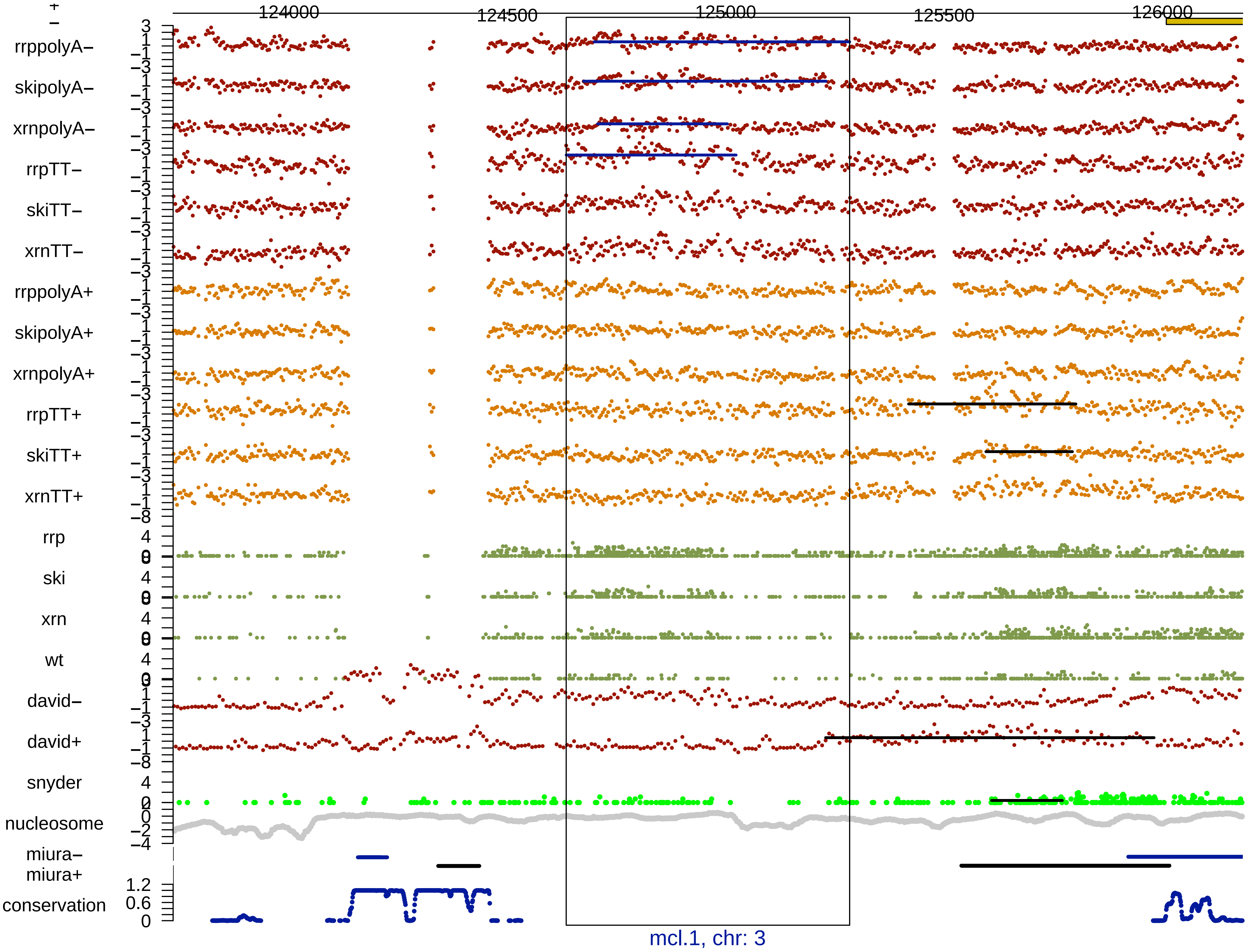
124000
124500
125000
125500
126000
+
−
rrppolyA−
skipolyA−
xrnpolyA−
rrpTT−
skiTT−
xrnTT−
rrppolyA+
skipolyA+
xrnpolyA+
rrpTT+
skiTT+
xrnTT+
rrp
ski
xrn
wt
david−
david+
snyder
nucleosome
miura−
miura+
conservation
mcl.1, chr: 3

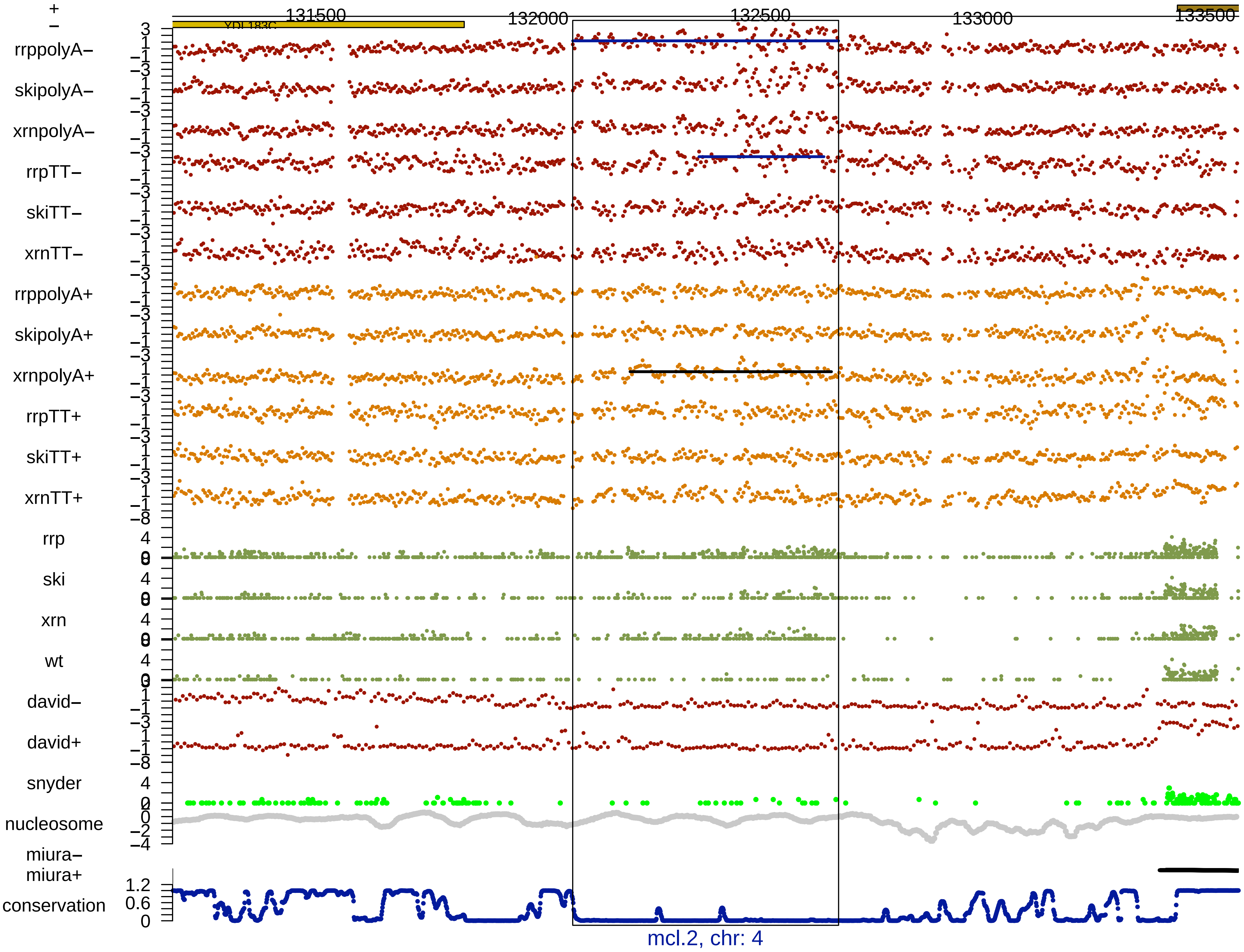

YDL183C
131500
132000
132500
133000
133500
rrppolyA−
skipolyA−
xrnpolyA−
rrpTT−
skiTT−
xrnTT−
rrppolyA+
skipolyA+
xrnpolyA+
rrpTT+
skiTT+
xrnTT+
rrp
ski
xrn
wt
david−
david+
snyder
nucleosome
miura−
miura+
conservation
mcl.2, chr: 4

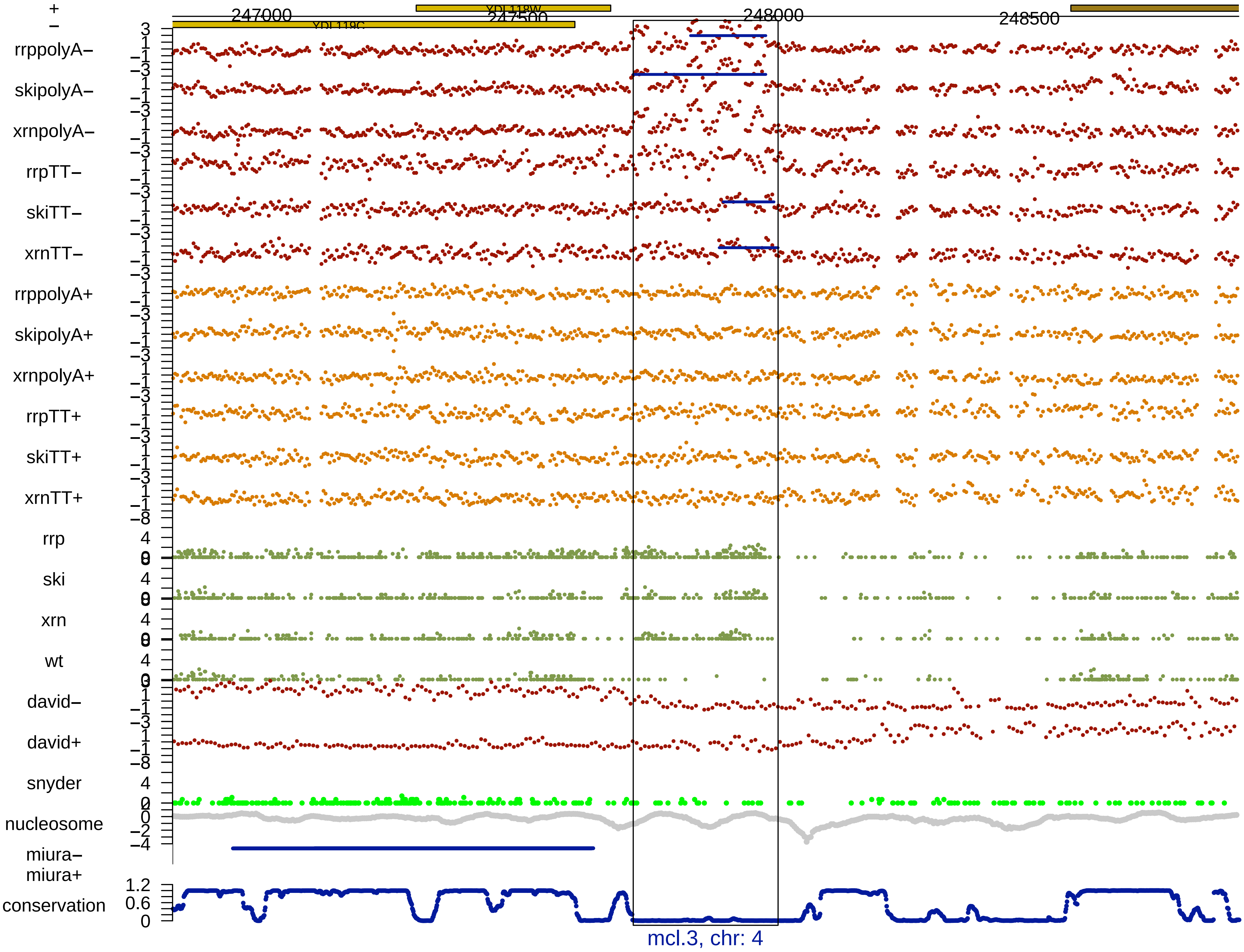

+
−
247000
247500
248000
248500
YDL118W
YDL119C
rrppolyA−
skipolyA−
xrnpolyA−
rrpTT−
skiTT−
xrnTT−
rrppolyA+
skipolyA+
xrnpolyA+
rrpTT+
skiTT+
xrnTT+
rrp
ski
xrn
wt
david−
david+
snyder
nucleosome
miura−
miura+
conservation
mcl.3, chr: 4

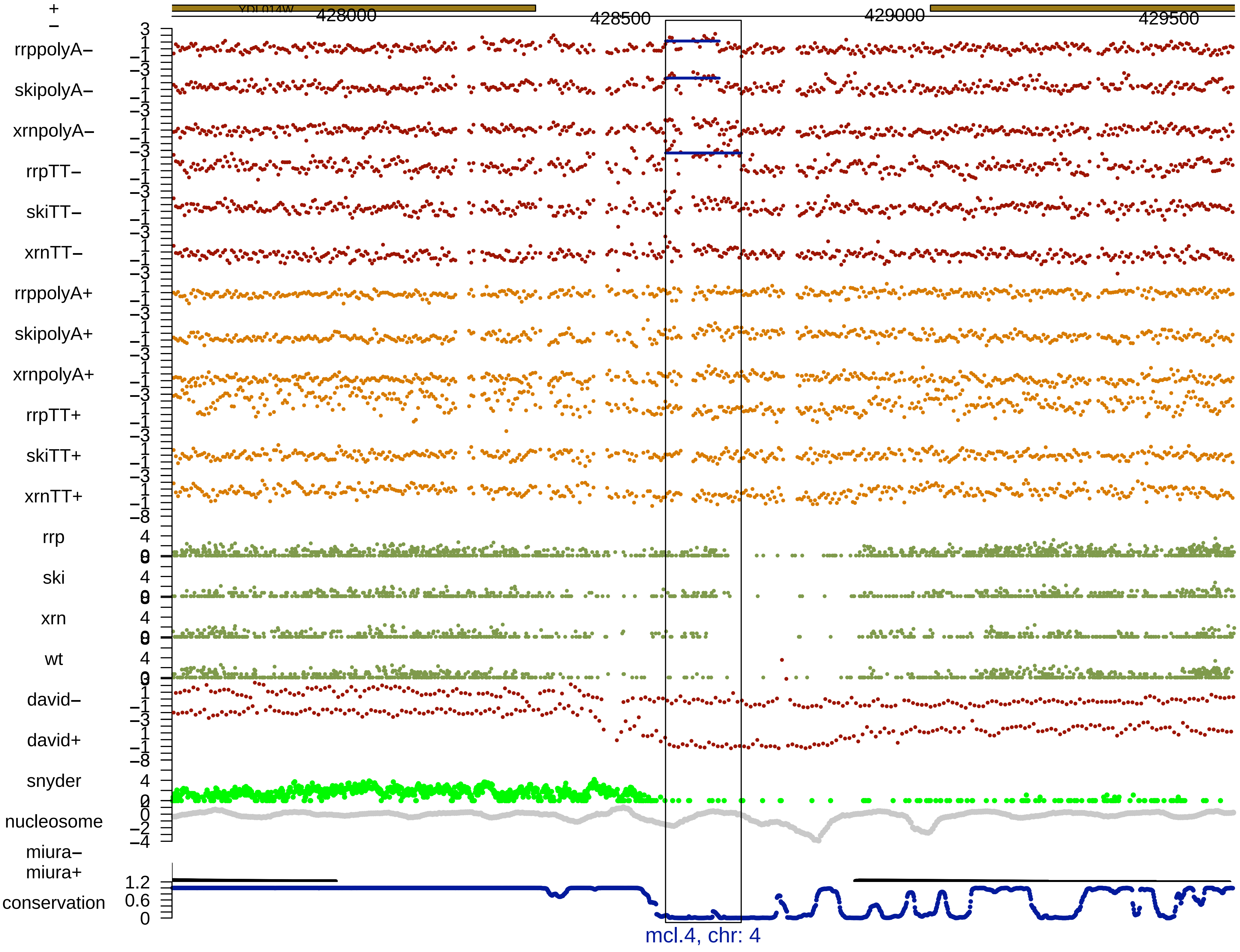
YDL014W
428000
428500
429000
429500
rrppolyA−
skipolyA−
xrnpolyA−
rrpTT−
skiTT−
xrnTT−
rrppolyA+
skipolyA+
xrnpolyA+
rrpTT+
skiTT+
xrnTT+
rrp
ski
xrn
wt
david−
david+
snyder
nucleosome
miura−
miura+
conservation
mcl.4, chr: 4

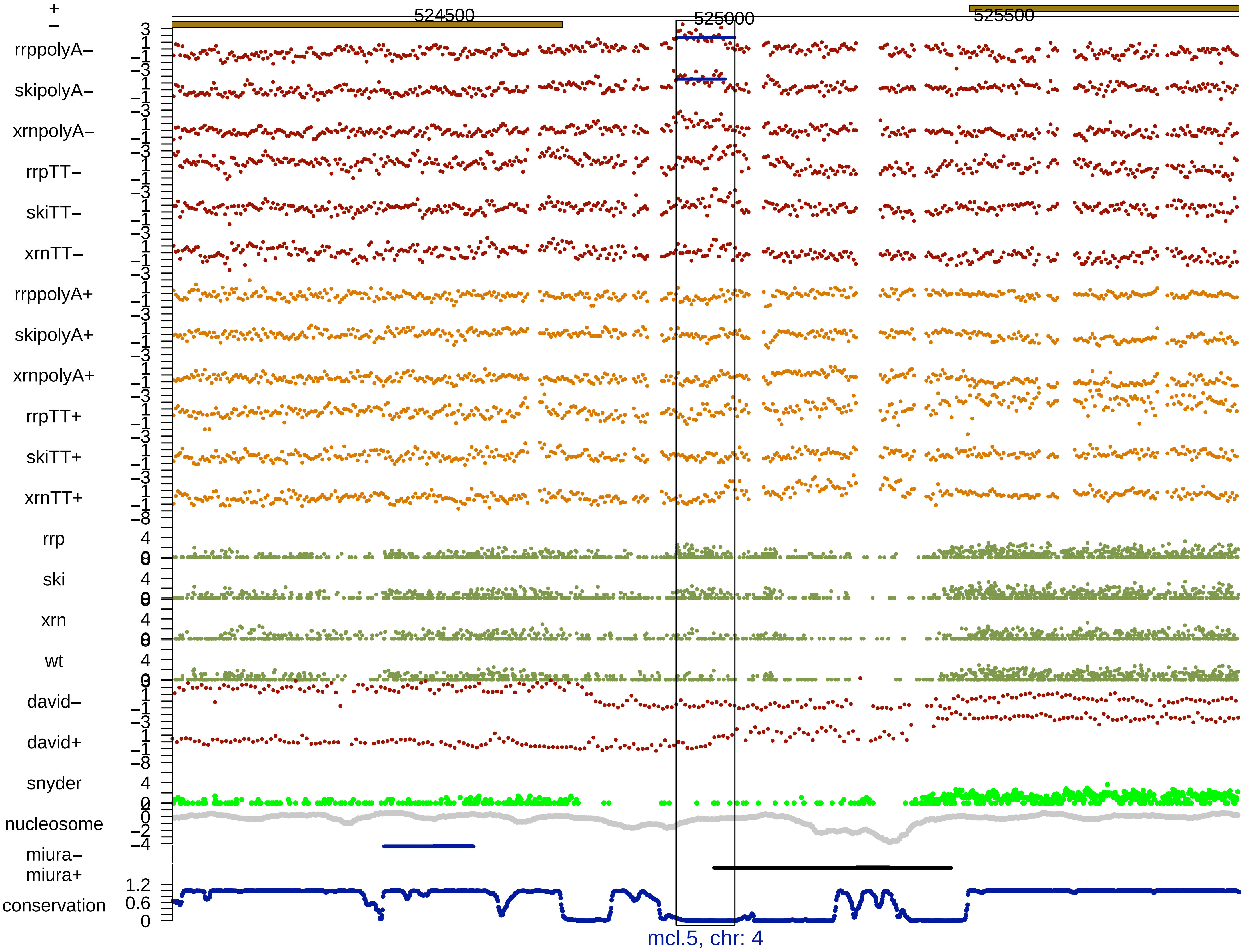

+
−
524500
525000
525500
rrppolyA−
skipolyA−
xrnpolyA−
rrpTT−
skiTT−
xrnTT−
rrppolyA+
skipolyA+
xrnpolyA+
rrpTT+
skiTT+
xrnTT+
rrp
ski
xrn
wt
david−
david+
snyder
nucleosome
miura−
miura+
conservation
mcl.5, chr: 4

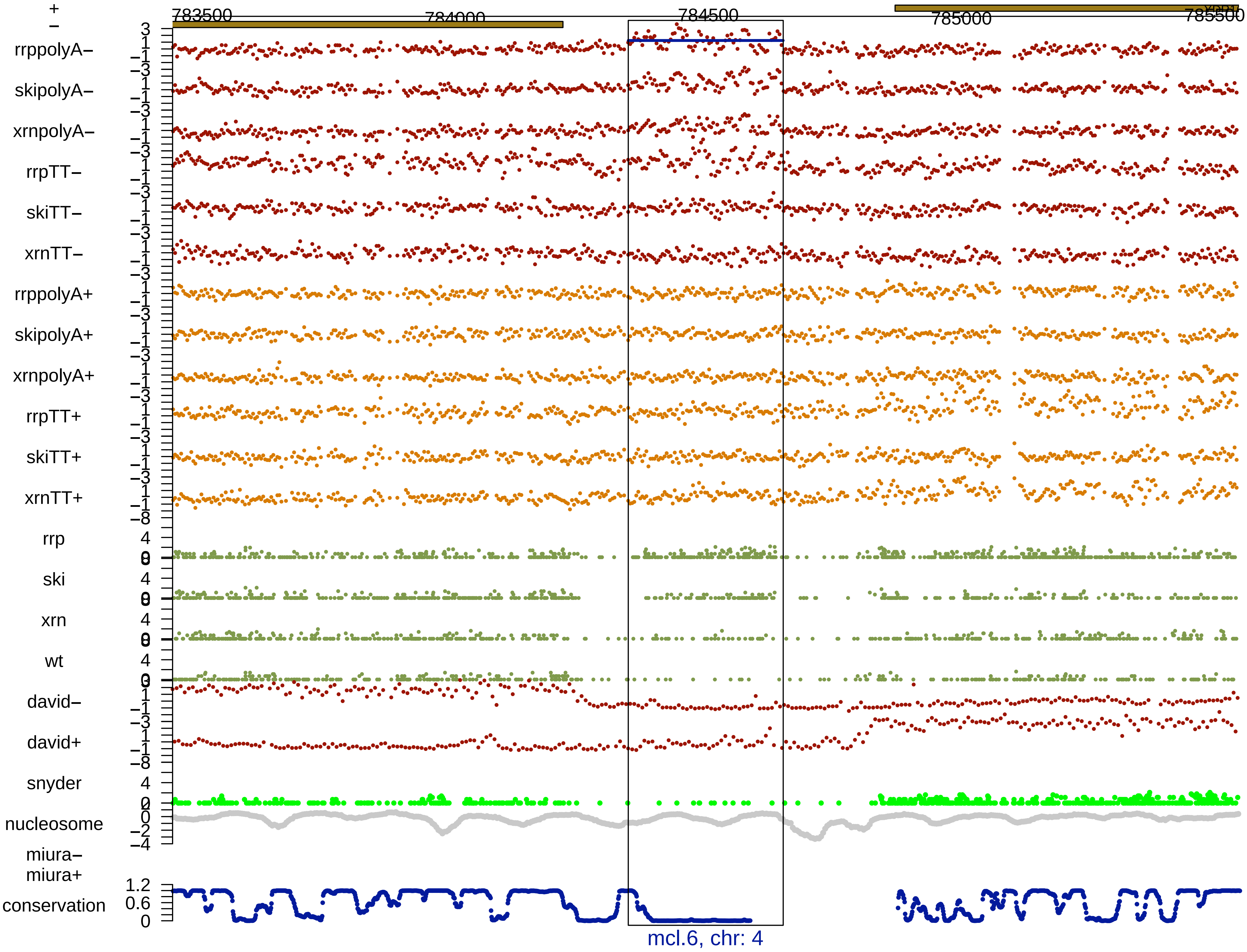

783500
784000
784500
785000
785500
rrppolyA−
skipolyA−
xrnpolyA−
rrpTT−
skiTT−
xrnTT−
rrppolyA+
skipolyA+
xrnpolyA+
rrpTT+
skiTT+
xrnTT+
rrp
ski
xrn
wt
david−
david+
snyder
nucleosome
miura−
miura+
conservation
mcl.6, chr: 4

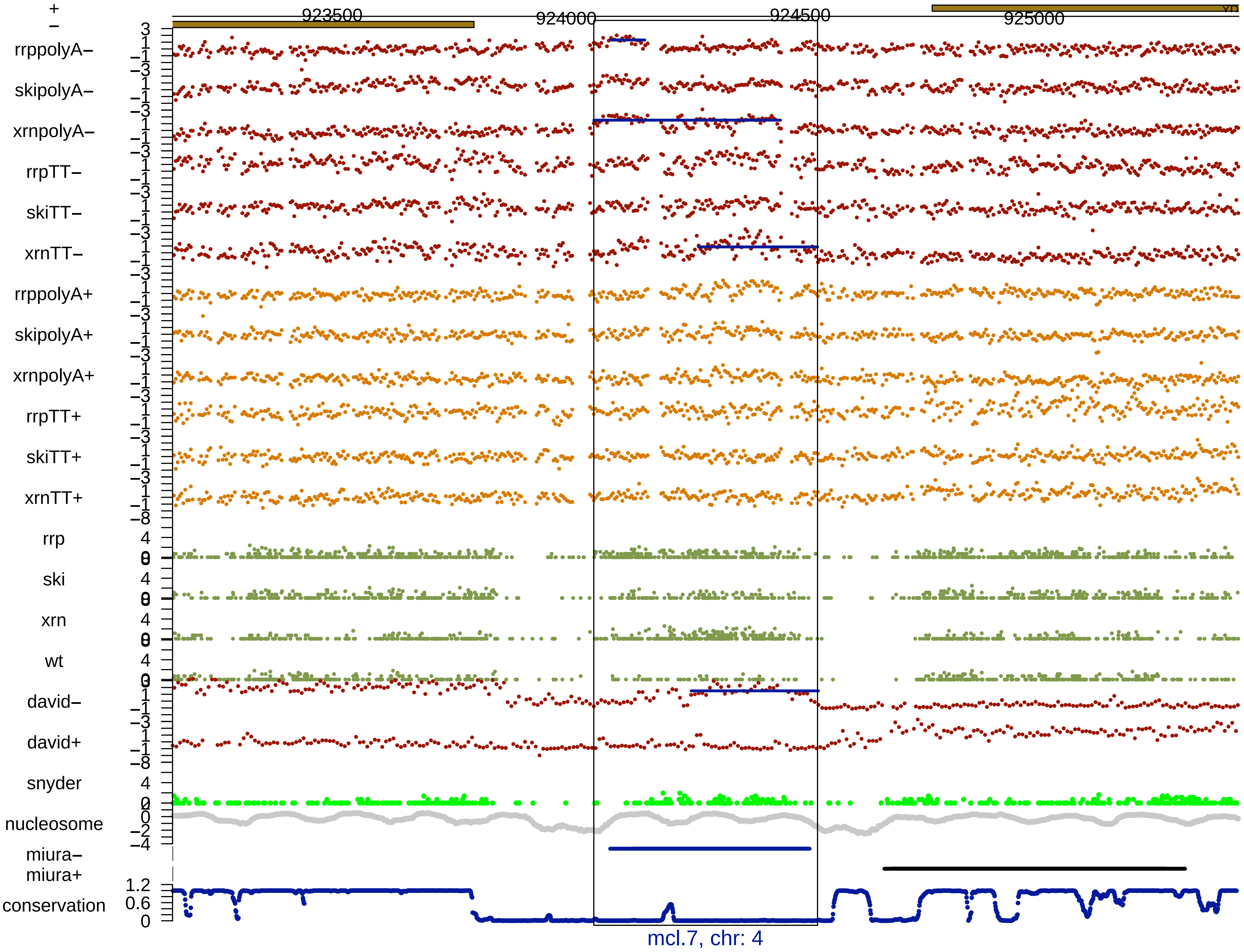

923500
924000
924500
925000
rrppolyA−
skipolyA−
xrnpolyA−
rrpTT−
skiTT−
xrnTT−
rrppolyA+
skipolyA+
xrnpolyA+
rrpTT+
skiTT+
xrnTT+
rrp
ski
xrn
wt
david−
david+
snyder
nucleosome
miura−
miura+
conservation
mcl.7, chr: 4

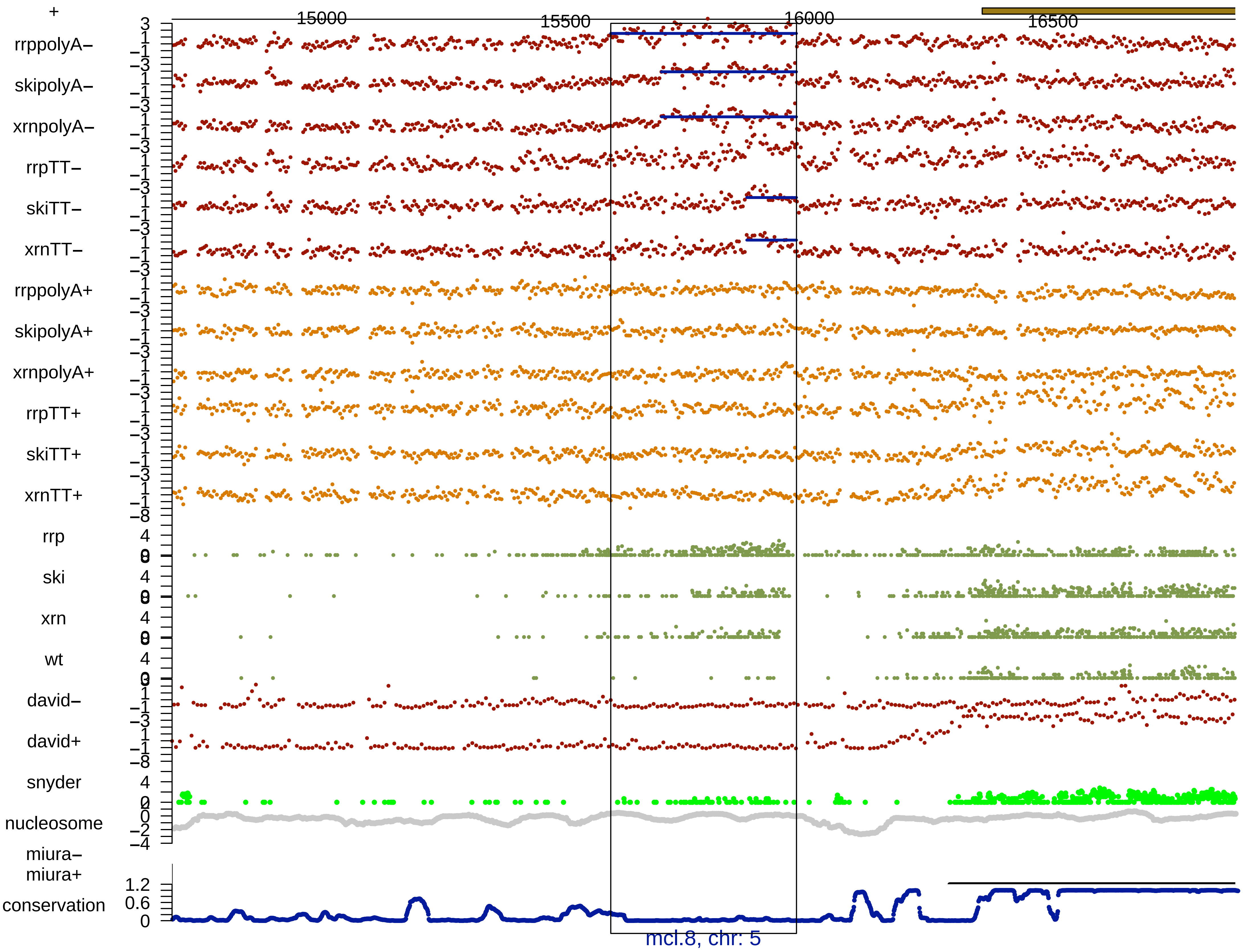
+
15000
15500
16000
16500
rrppolyA–
skipolyA–
xrnpolyA–
rrpTT–
skiTT–
xrnTT–
rrppolyA+
skipolyA+
xrnpolyA+
rrpTT+
skiTT+
xrnTT+
rrp
ski
xrn
wt
david–
david+
snyder
nucleosome
miura–
miura+
conservation
mcl.8, chr: 5

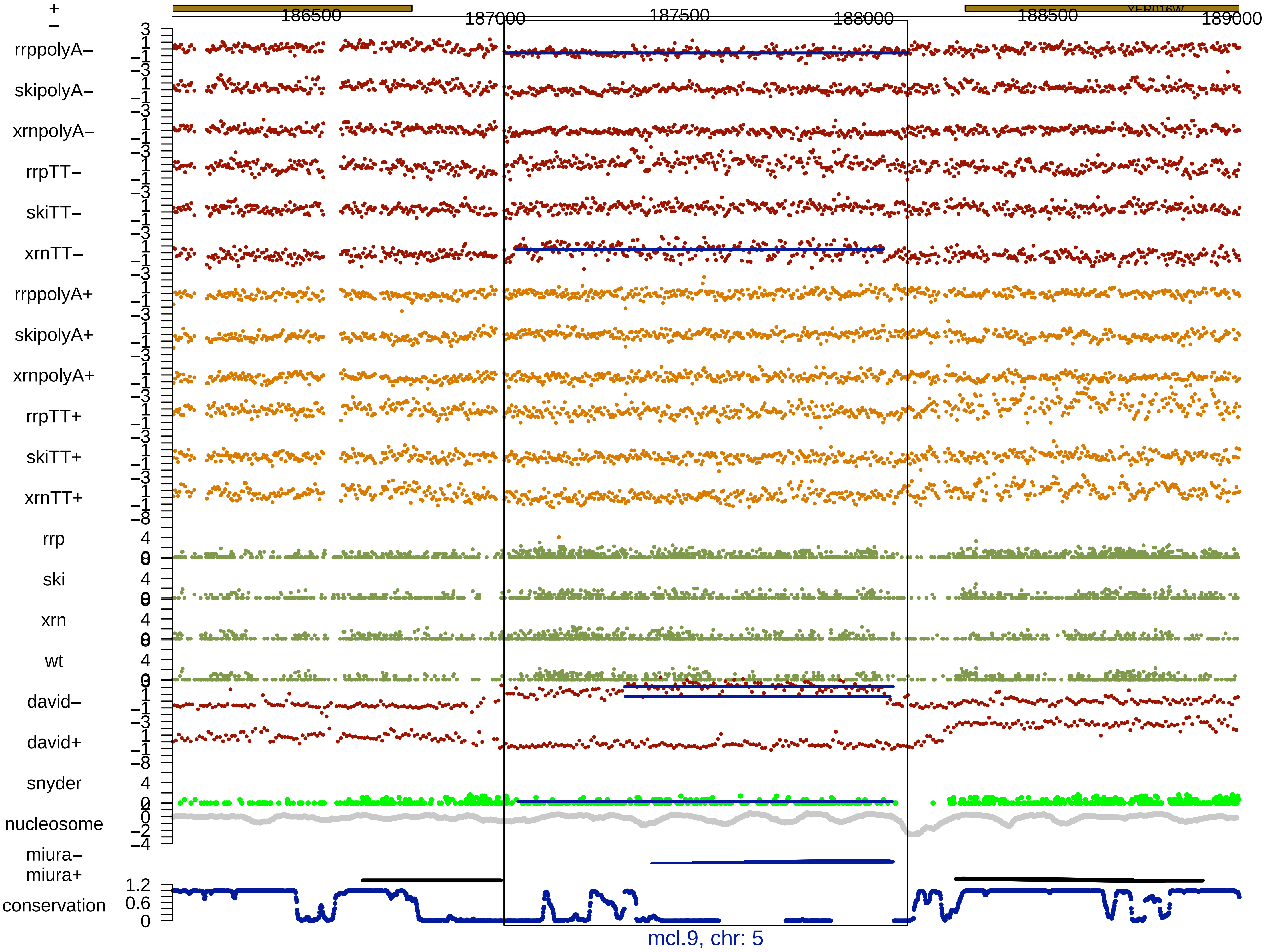

+
−
186500
187000
187500
188000
188500
YER016W
189000
rrppolyA−
skipolyA−
xrnpolyA−
rrpTT−
skiTT−
xrnTT−
rrppolyA+
skipolyA+
xrnpolyA+
rrpTT+
skiTT+
xrnTT+
rrp
ski
xrn
wt
david−
david+
snyder
nucleosome
miura−
miura+
conservation
mcl.9, chr: 5

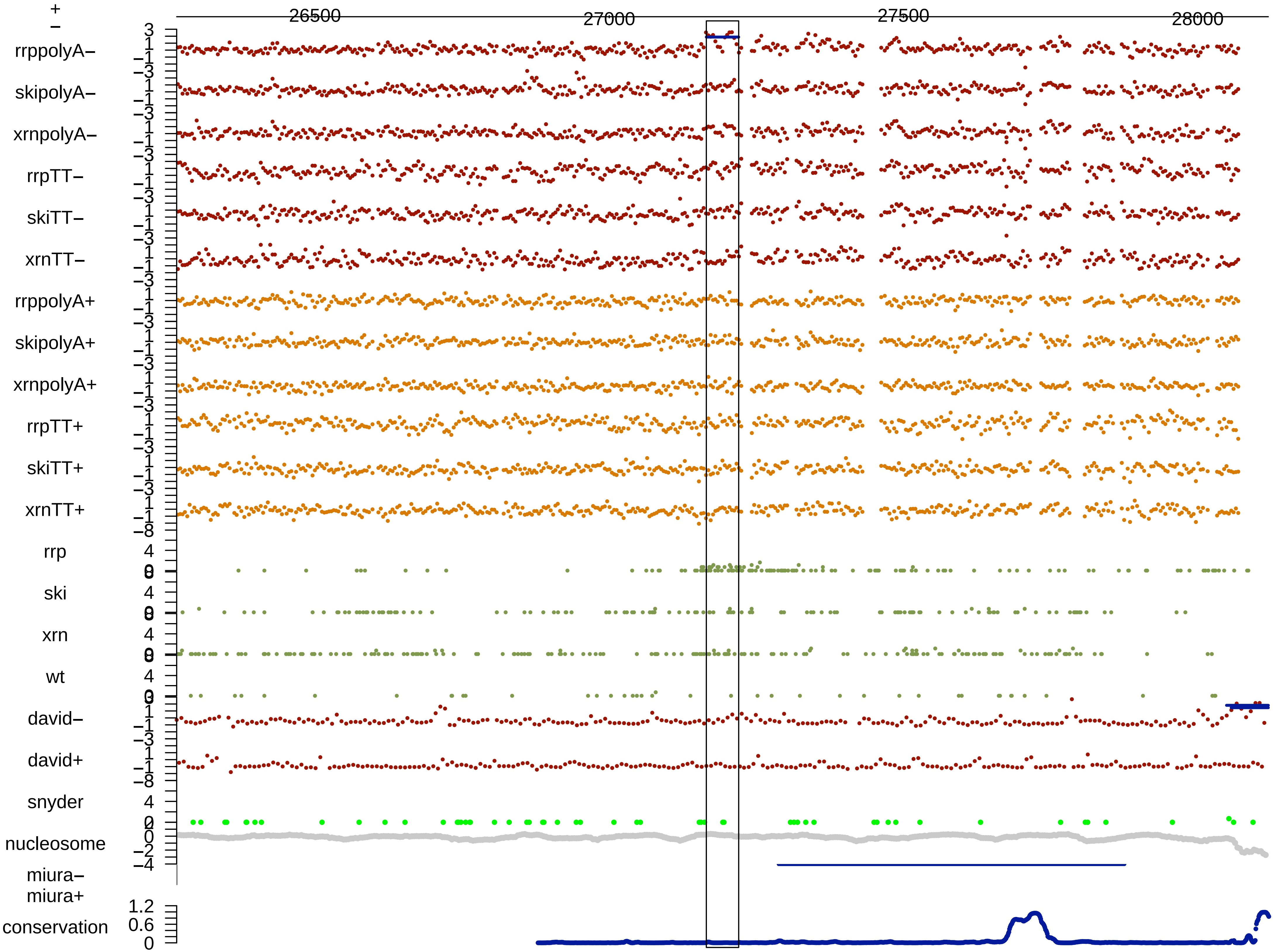

26500
27000
27500
28000
+
−
rrppolyA−
skipolyA−
xrnpolyA−
rrpTT−
skiTT−
xrnTT−
rrppolyA+
skipolyA+
xrnpolyA+
rrpTT+
skiTT+
xrnTT+
rrp
ski
xrn
wt
david−
david+
snyder
nucleosome
miura−
miura+
conservation
1.2
0.6
0

mcl.10, chr: 6

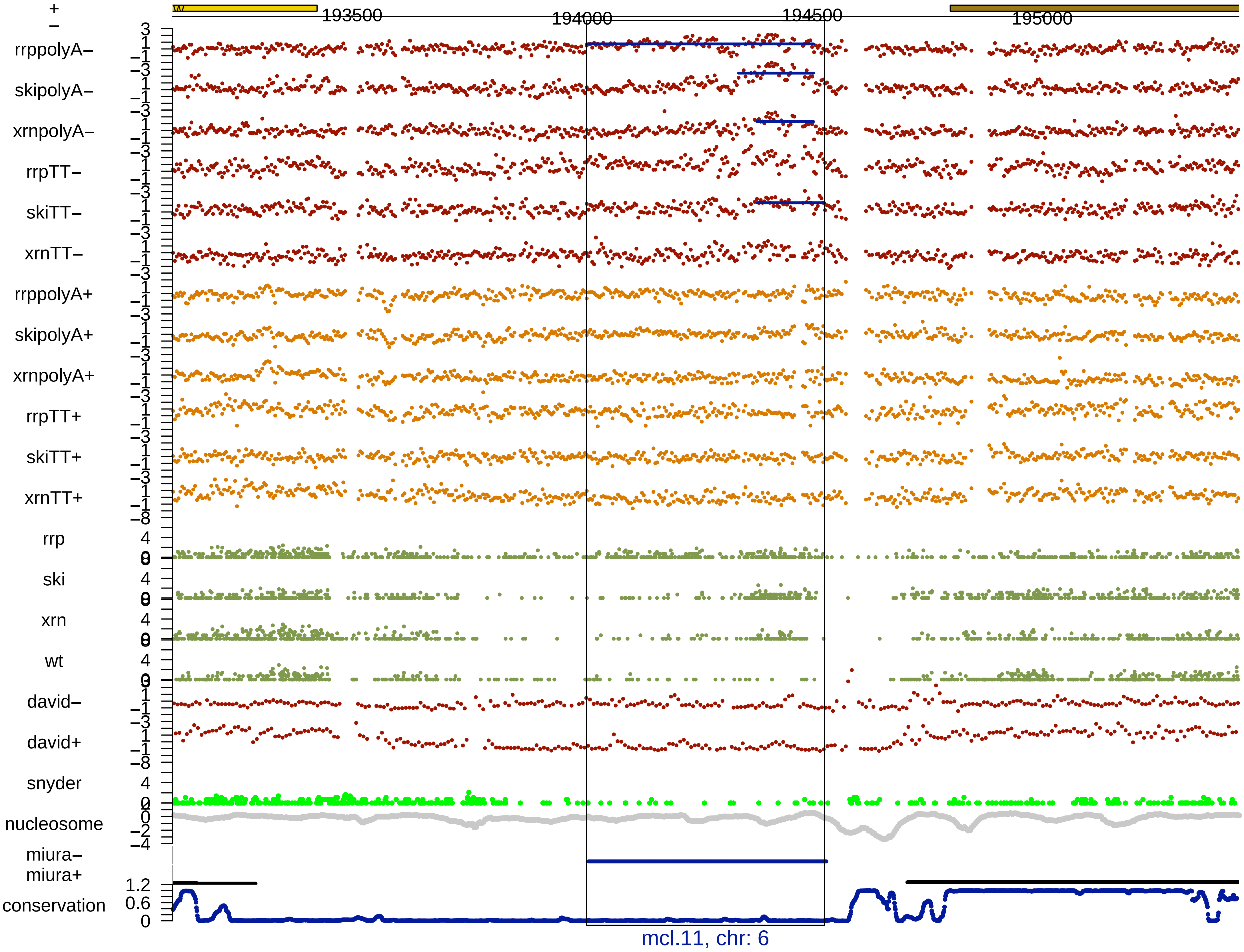

+
−
193500
194000
194500
195000
rrppolyA−
skipolyA−
xrnpolyA−
rrpTT−
skiTT−
xrnTT−
rrppolyA+
skipolyA+
xrnpolyA+
rrpTT+
skiTT+
xrnTT+
rrp
ski
xrn
wt
david−
david+
snyder
nucleosome
miura−
miura+
conservation
mcl.11, chr: 6

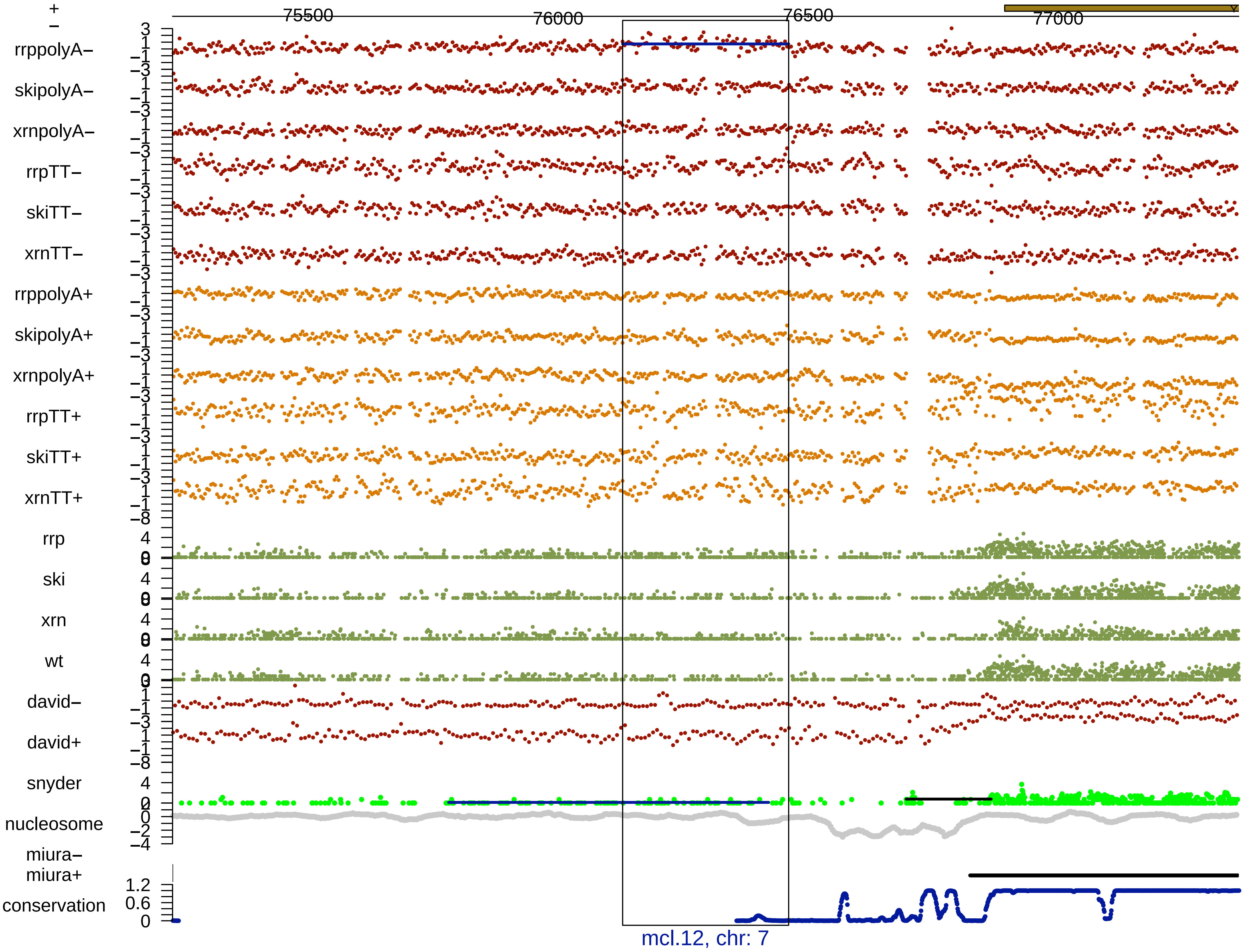

75500
76000
76500
77000
rrppolyA−
skipolyA−
xrnpolyA−
rrpTT−
skiTT−
xrnTT−
rrppolyA+
skipolyA+
xrnpolyA+
rrpTT+
skiTT+
xrnTT+
rrp
ski
xrn
wt
david−
david+
snyder
nucleosome
miura−
miura+
conservation
mcl.12, chr: 7

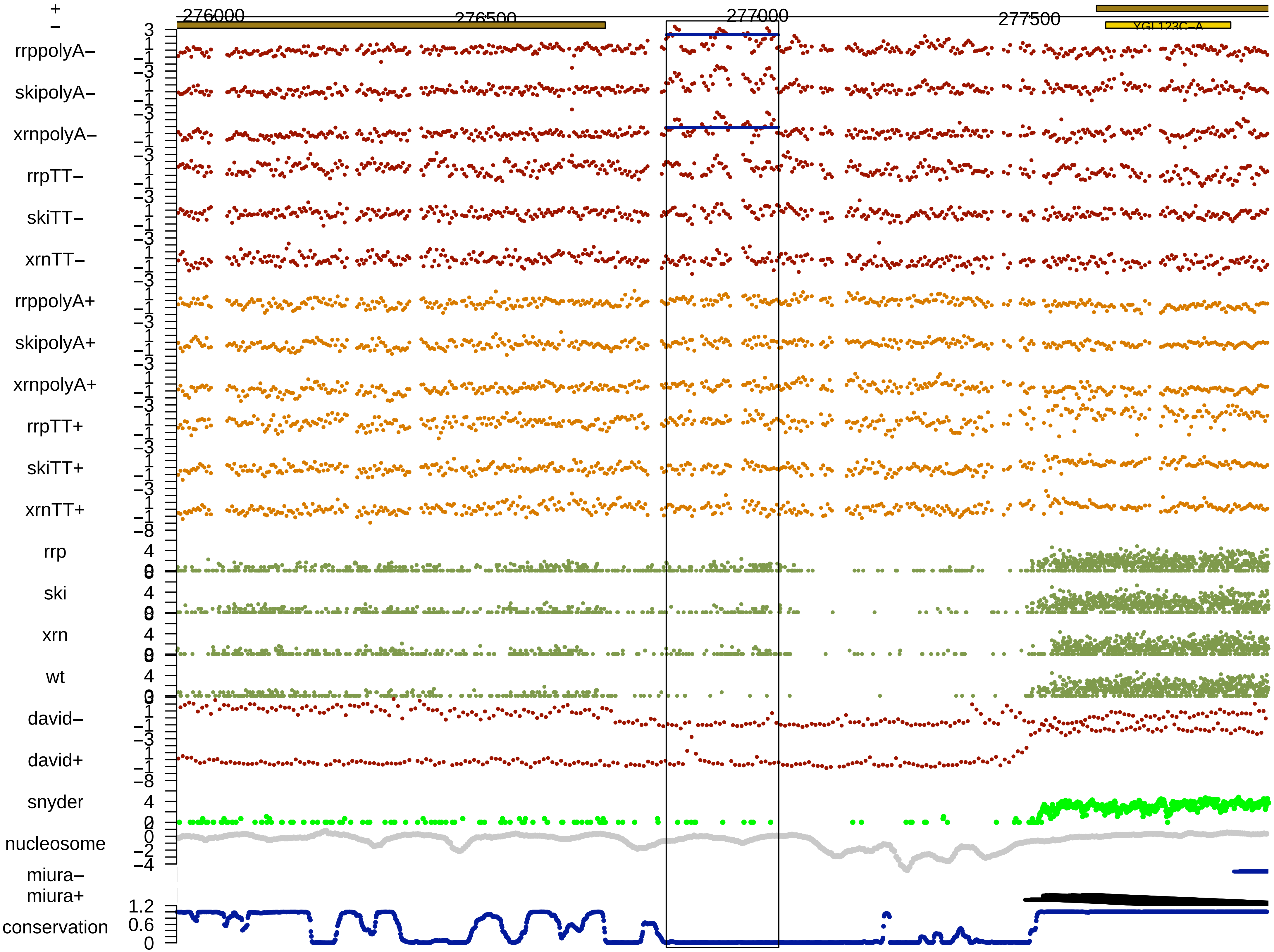

+
−
276000
276500
277000
277500
YGL123C-A
rrppolyA−
skipolyA−
xrnpolyA−
rrpTT−
skiTT−
xrnTT−
rrppolyA+
skipolyA+
xrnpolyA+
rrpTT+
skiTT+
xrnTT+
rrp
ski
xrn
wt
david−
david+
snyder
nucleosome
miura−
miura+
conservation
1.2
0.6
0

mcl.13, chr: 7

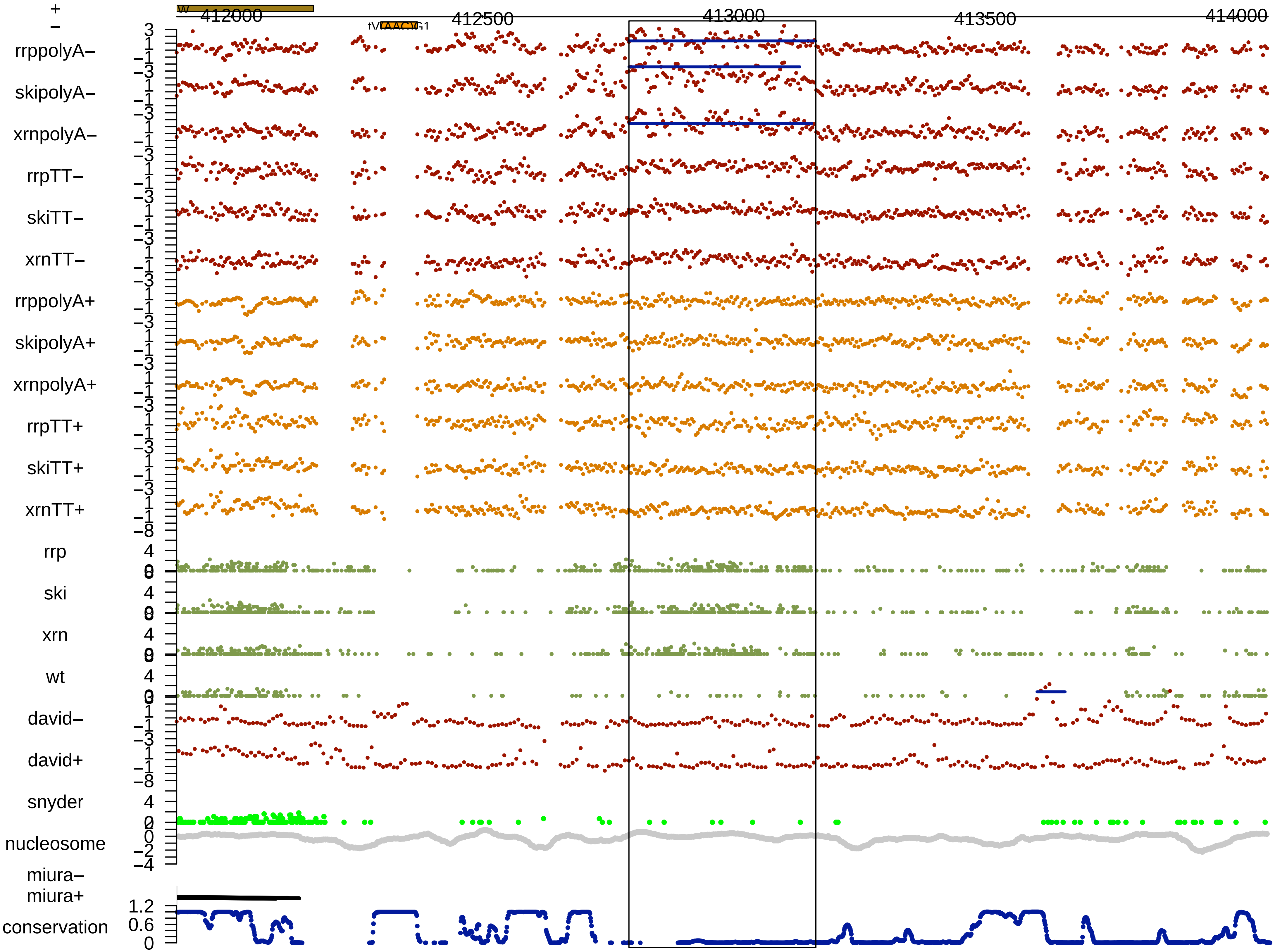
+
–
412000
412500
413000
413500
414000
rrppolyA–
skipolyA–
xrnpolyA–
rrpTT–
skiTT–
xrnTT–
rrppolyA+
skipolyA+
xrnpolyA+
rrpTT+
skiTT+
xrnTT+
rrp
ski
xrn
wt
david–
david+
snyder
nucleosome
miura–
miura+
conservation

mcl.14, chr: 7

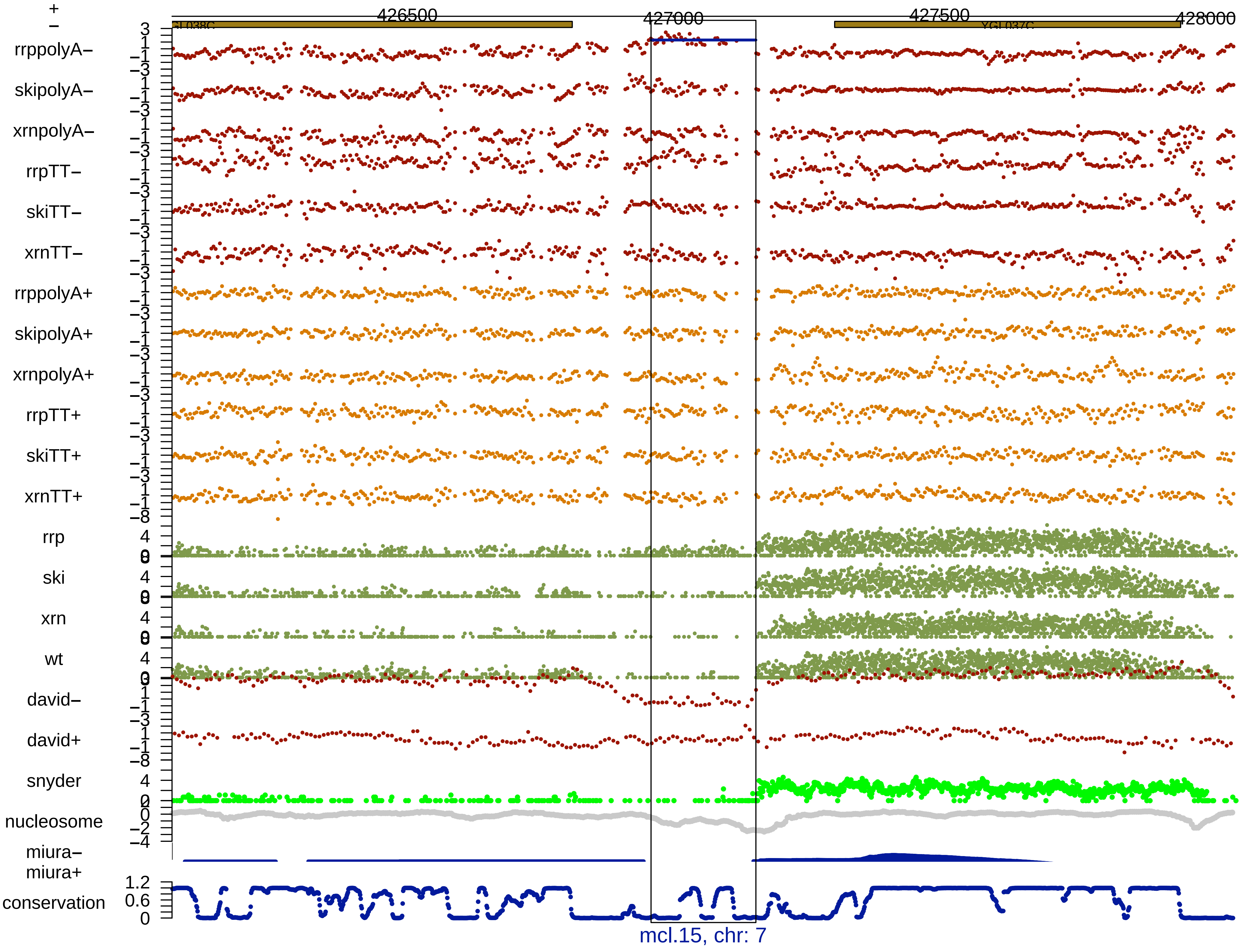

+
–
426500
427000
427500
428000
rrppolyA–
skipolyA–
xrnpolyA–
rrpTT–
skiTT–
xrnTT–
rrppolyA+
skipolyA+
xrnpolyA+
rrpTT+
skiTT+
xrnTT+
rrp
ski
xrn
wt
david–
david+
snyder
nucleosome
miura–
miura+
conservation
mcl.15, chr: 7

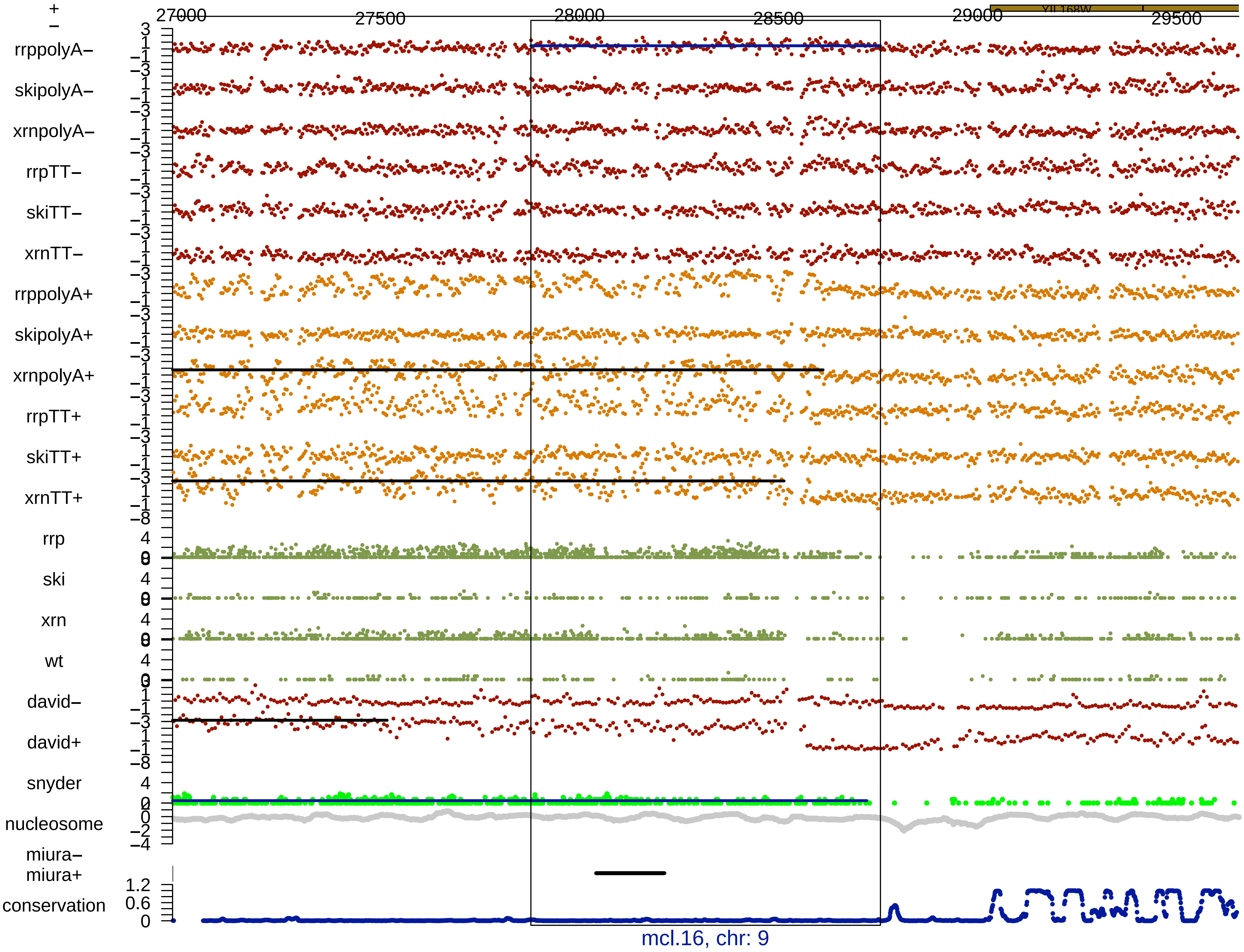

+
−
27000
27500
28000
28500
29000
29500
YIL168W
rrppolyA−
skipolyA−
xrnpolyA−
rrpTT−
skiTT−
xrnTT−
rrppolyA+
skipolyA+
xrnpolyA+
rrpTT+
skiTT+
xrnTT+
rrp
ski
xrn
wt
david−
david+
snyder
nucleosome
miura−
miura+
conservation
mcl.16, chr: 9

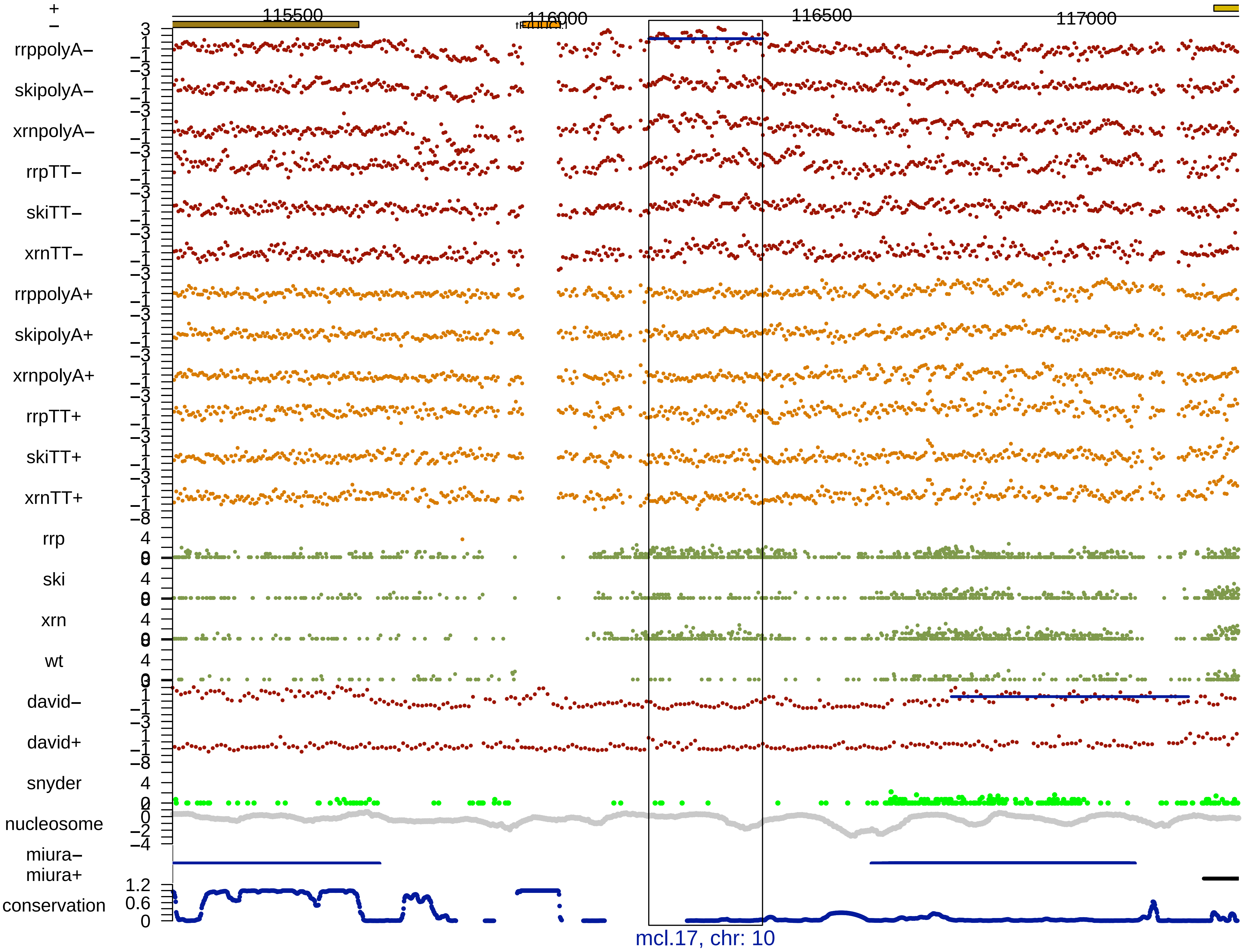
+
−
115500
116000
116500
117000
rrppolyA−
skipolyA−
xrnpolyA−
rrpTT−
skiTT−
xrnTT−
rrppolyA+
skipolyA+
xrnpolyA+
rrpTT+
skiTT+
xrnTT+
rrp
ski
xrn
wt
david−
david+
snyder
nucleosome
miura−
miura+
conservation
mcl.17, chr: 10

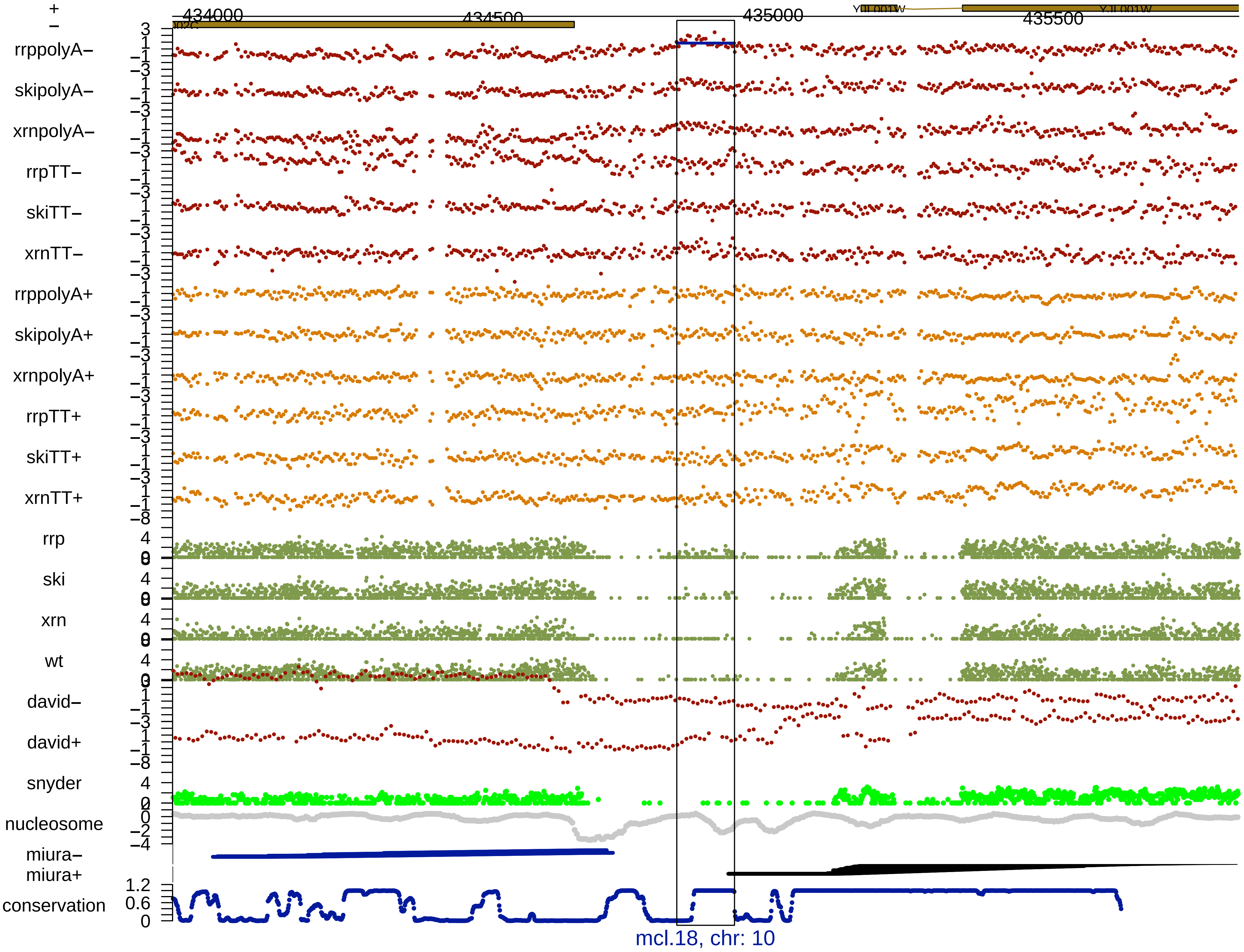

+
−
434000
434500
435000
435500
YJL001W
rrppolyA−
skipolyA−
xrnpolyA−
rrpTT−
skiTT−
xrnTT−
rrppolyA+
skipolyA+
xrnpolyA+
rrpTT+
skiTT+
xrnTT+
rrp
ski
xrn
wt
david−
david+
snyder
nucleosome
miura−
miura+
conservation
mcl.18, chr: 10

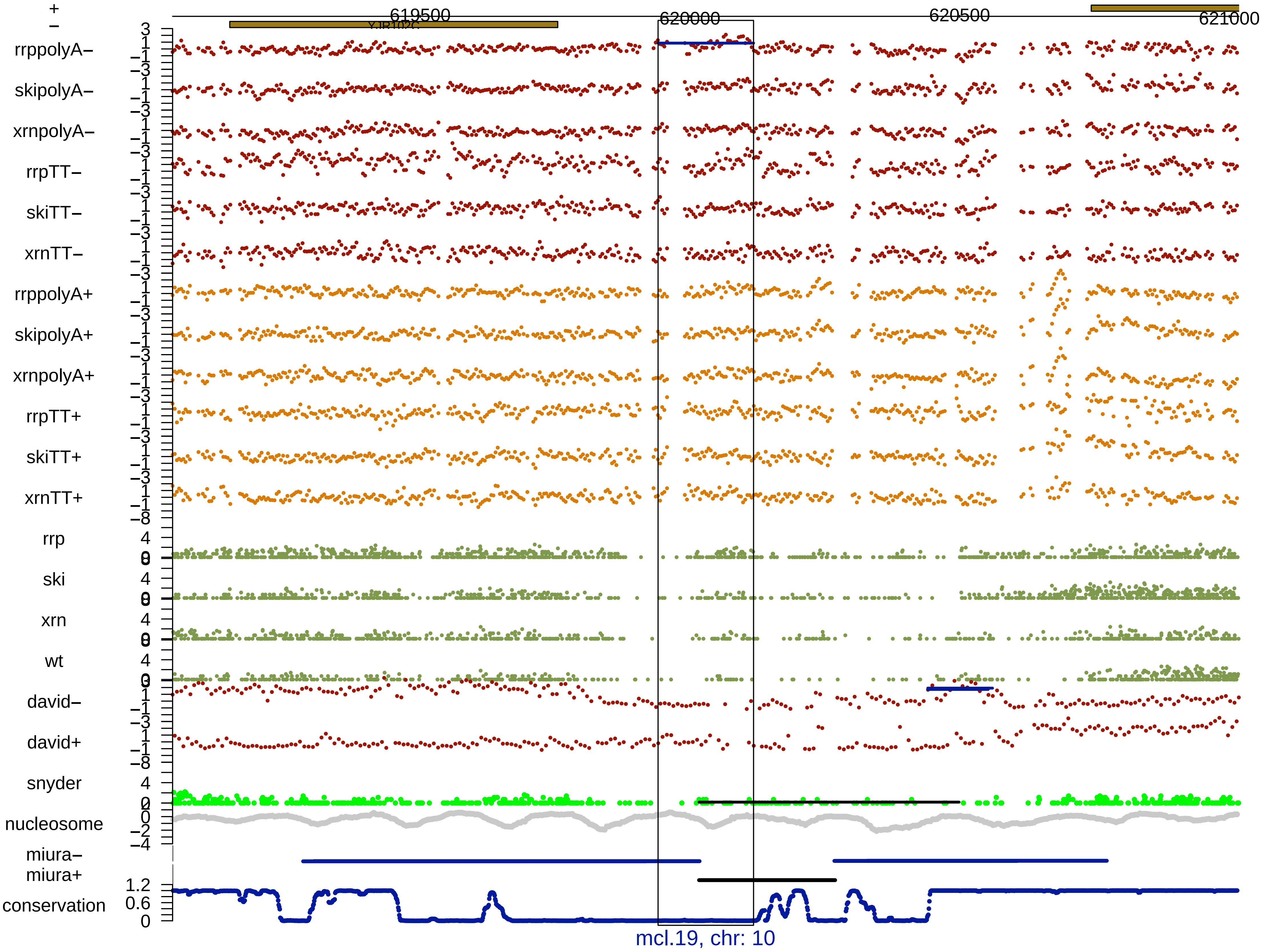

+
−
619500
620000
620500
621000
rrppolyA−
skipolyA−
xrnpolyA−
rrpTT−
skiTT−
xrnTT−
rrppolyA+
skipolyA+
xrnpolyA+
rrpTT+
skiTT+
xrnTT+
rrp
ski
xrn
wt
david−
david+
snyder
nucleosome
miura−
miura+
conservation
mcl.19, chr: 10

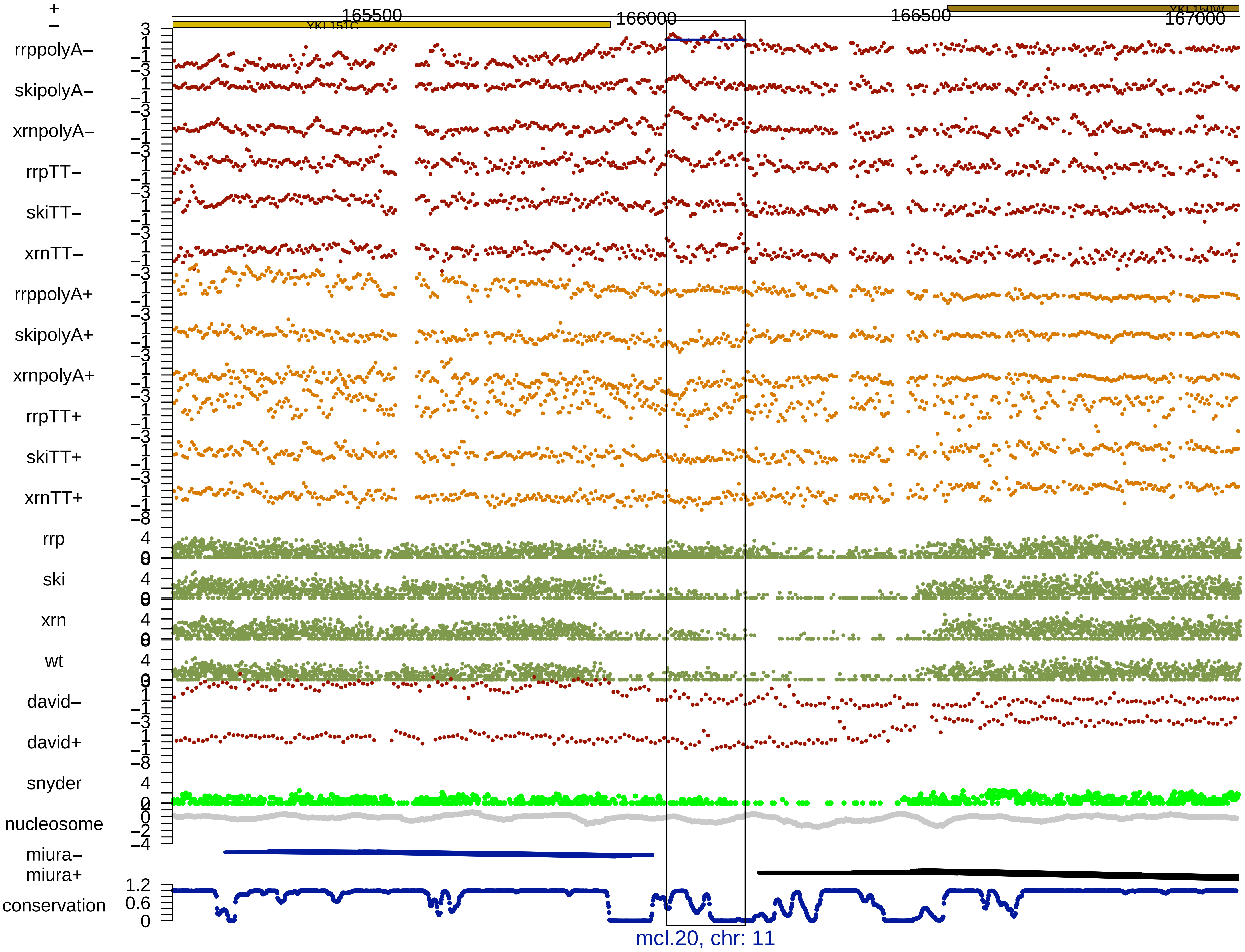

165500
166000
166500
167000
YKL151C
YKL150W
rrppolyA−
skipolyA−
xrnpolyA−
rrpTT−
skiTT−
xrnTT−
rrppolyA+
skipolyA+
xrnpolyA+
rrpTT+
skiTT+
xrnTT+
rrp
ski
xrn
wt
david−
david+
snyder
nucleosome
miura−
miura+
conservation
mcl.20, chr: 11

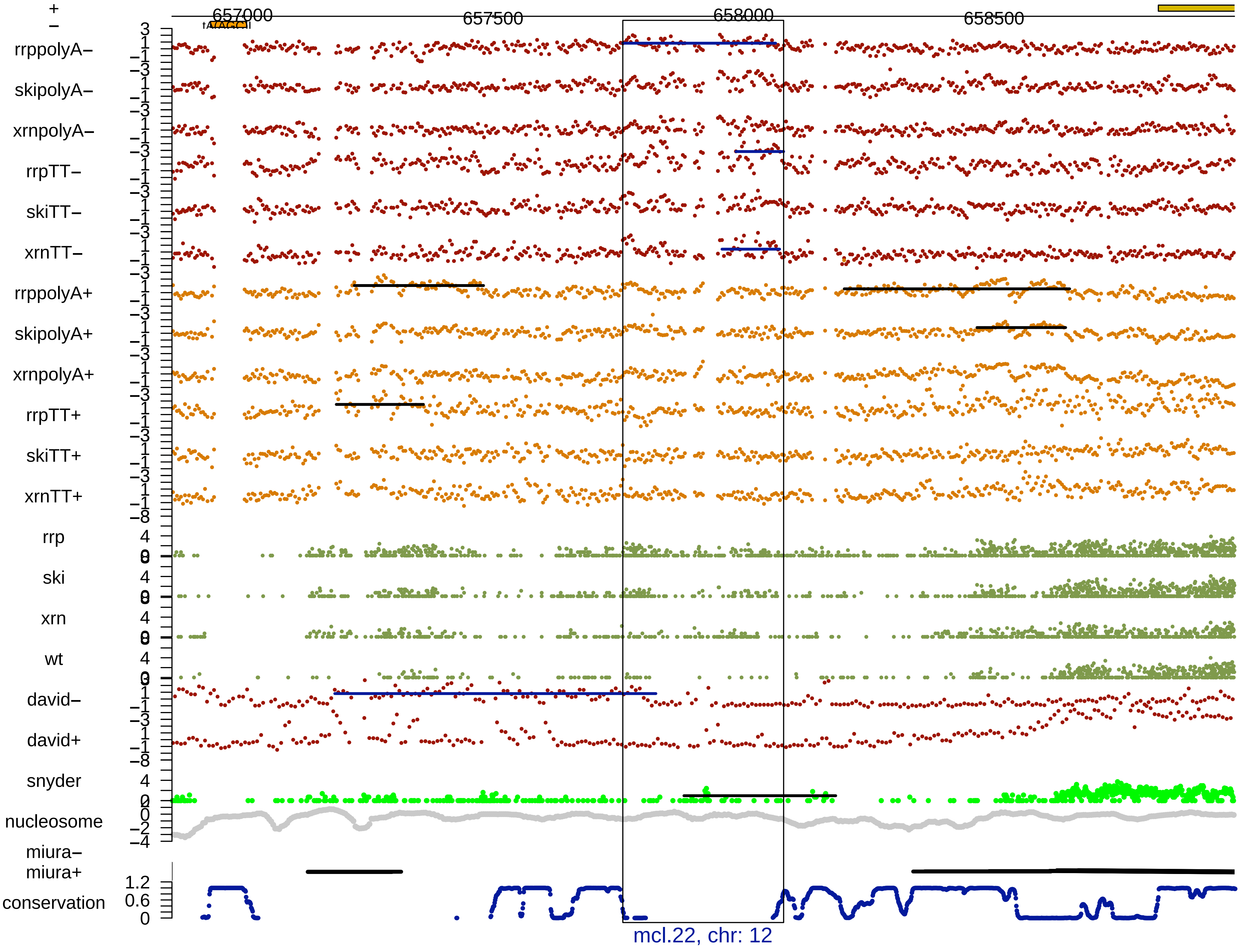

657000
657500
658000
658500
rrppolyA−
skipolyA−
xrnpolyA−
rrpTT−
skiTT−
xrnTT−
rrppolyA+
skipolyA+
xrnpolyA+
rrpTT+
skiTT+
xrnTT+
rrp
ski
xrn
wt
david−
david+
snyder
nucleosome
miura−
miura+
conservation
mcl.22, chr: 12

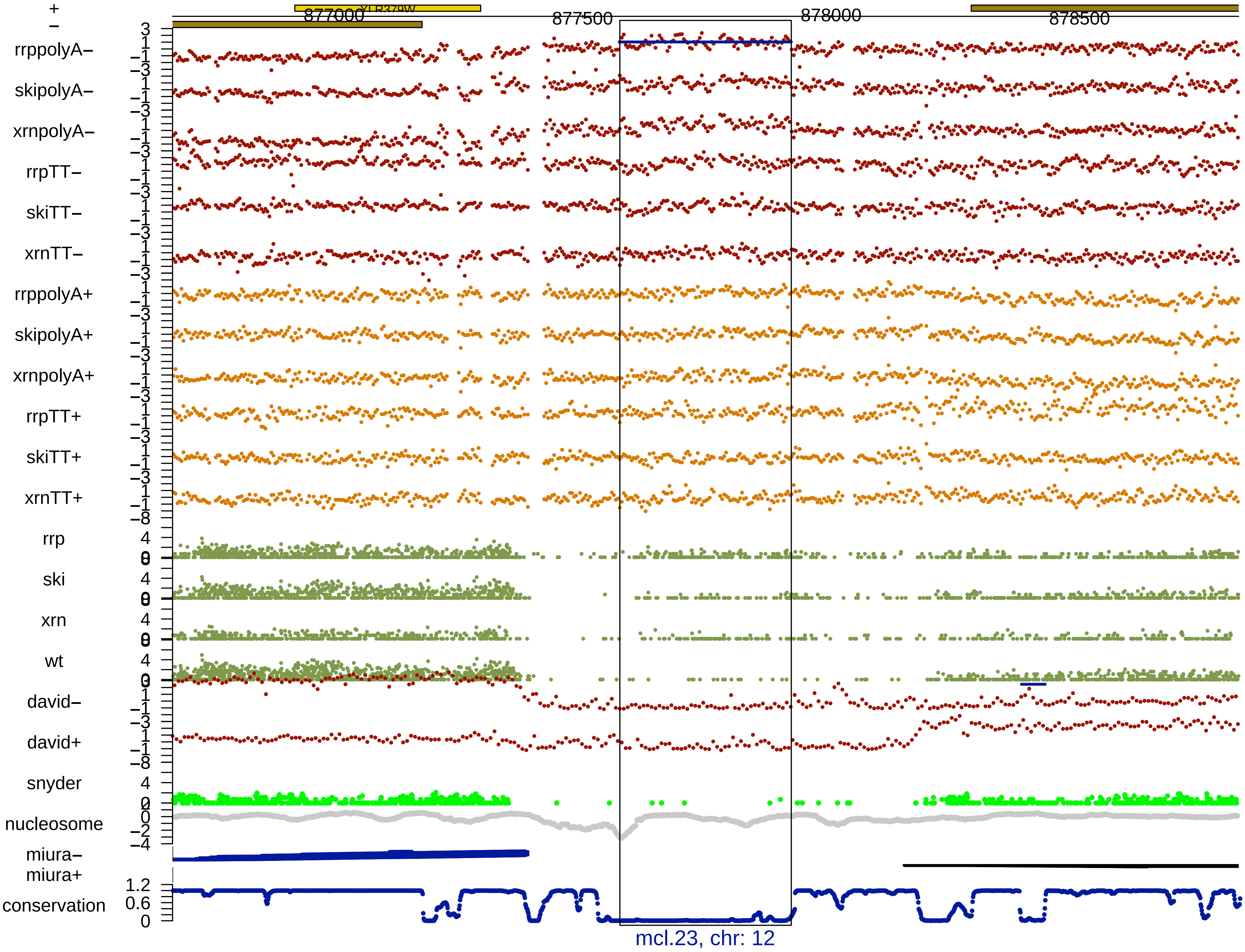

YLR379W
877000
877500
878000
878500
rrppolyA−
skipolyA−
xrnpolyA−
rrpTT−
skiTT−
xrnTT−
rrppolyA+
skipolyA+
xrnpolyA+
rrpTT+
skiTT+
xrnTT+
rrp
ski
xrn
wt
david−
david+
snyder
nucleosome
miura−
miura+
conservation
mcl.23, chr: 12

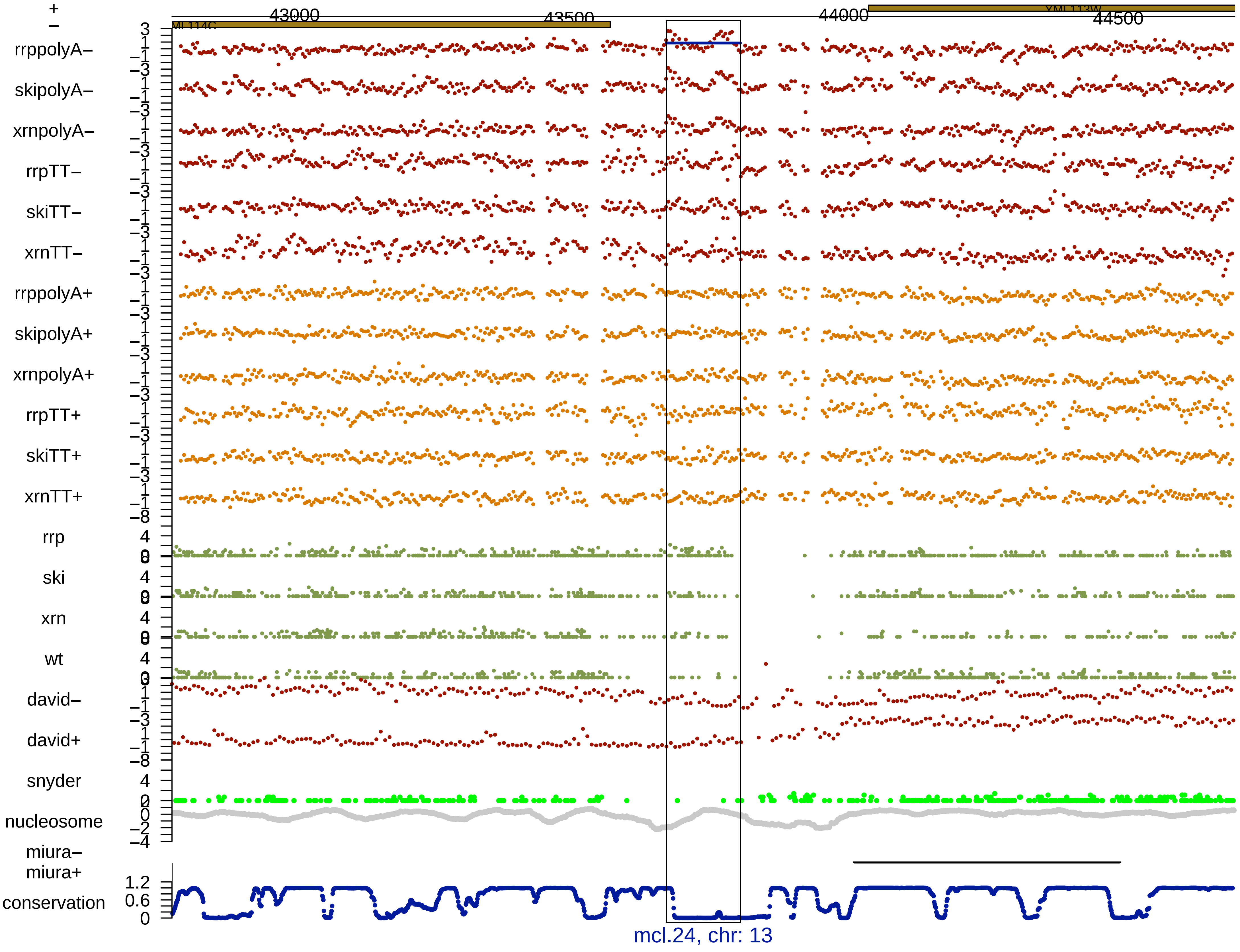

+
−
43000
43500
44000
44500
YML113W
rrppolyA−
skipolyA−
xrnpolyA−
rrpTT−
skiTT−
xrnTT−
rrppolyA+
skipolyA+
xrnpolyA+
rrpTT+
skiTT+
xrnTT+
rrp
ski
xrn
wt
david−
david+
snyder
nucleosome
miura−
miura+
conservation
mcl.24, chr: 13

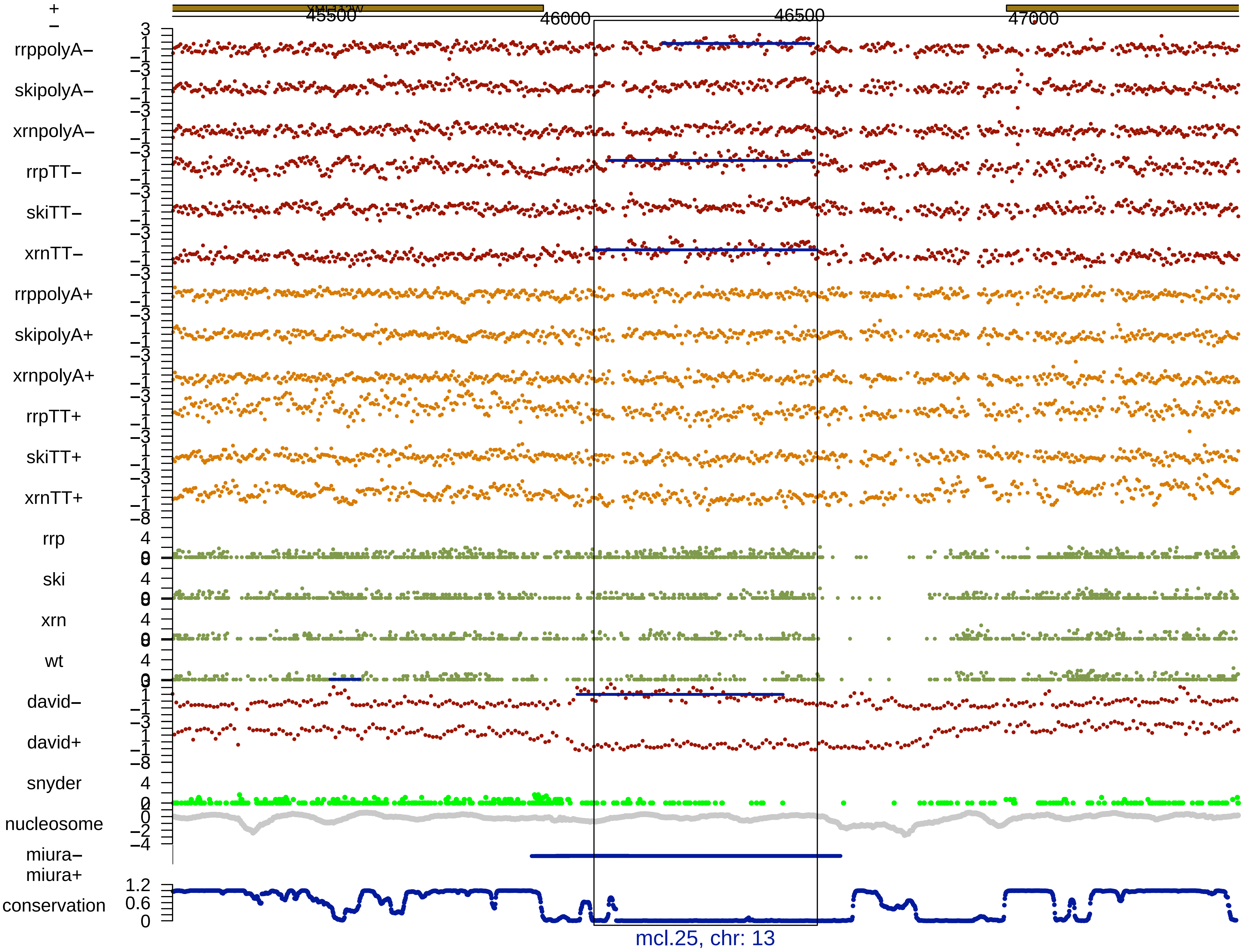
+
–
45500
46000
46500
47000
rrppolyA–
skipolyA–
xrnpolyA–
rrpTT–
skiTT–
xrnTT–
rrppolyA+
skipolyA+
xrnpolyA+
rrpTT+
skiTT+
xrnTT+
rrp
ski
xrn
wt
david–
david+
snyder
nucleosome
miura–
miura+
conservation
mcl.25, chr: 13

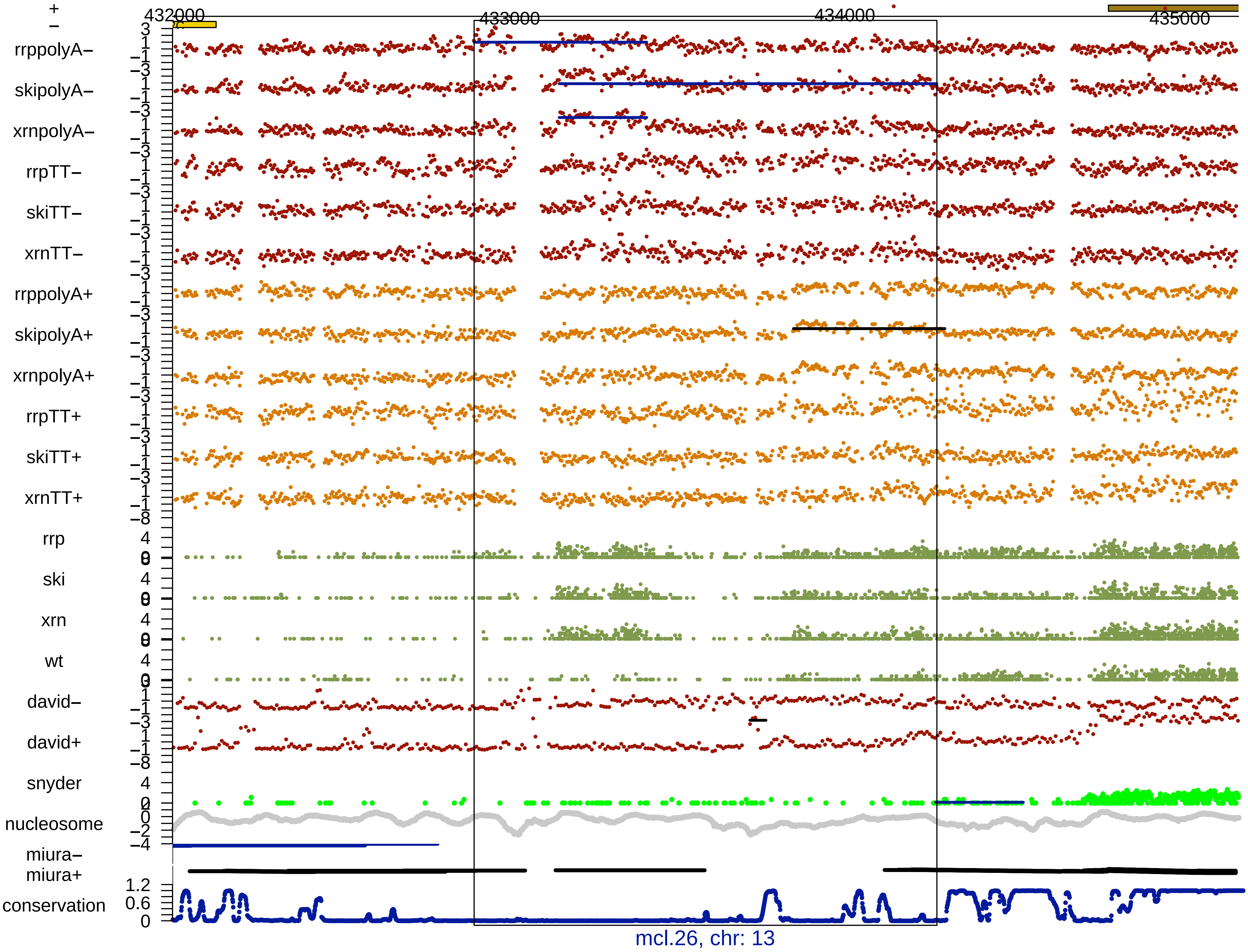

432000
433000
434000
435000
rrppolyA−
skipolyA−
xrnpolyA−
rrpTT−
skiTT−
xrnTT−
rrppolyA+
skipolyA+
xrnpolyA+
rrpTT+
skiTT+
xrnTT+
rrp
ski
xrn
wt
david−
david+
snyder
nucleosome
miura−
miura+
conservation
mcl.26, chr: 13

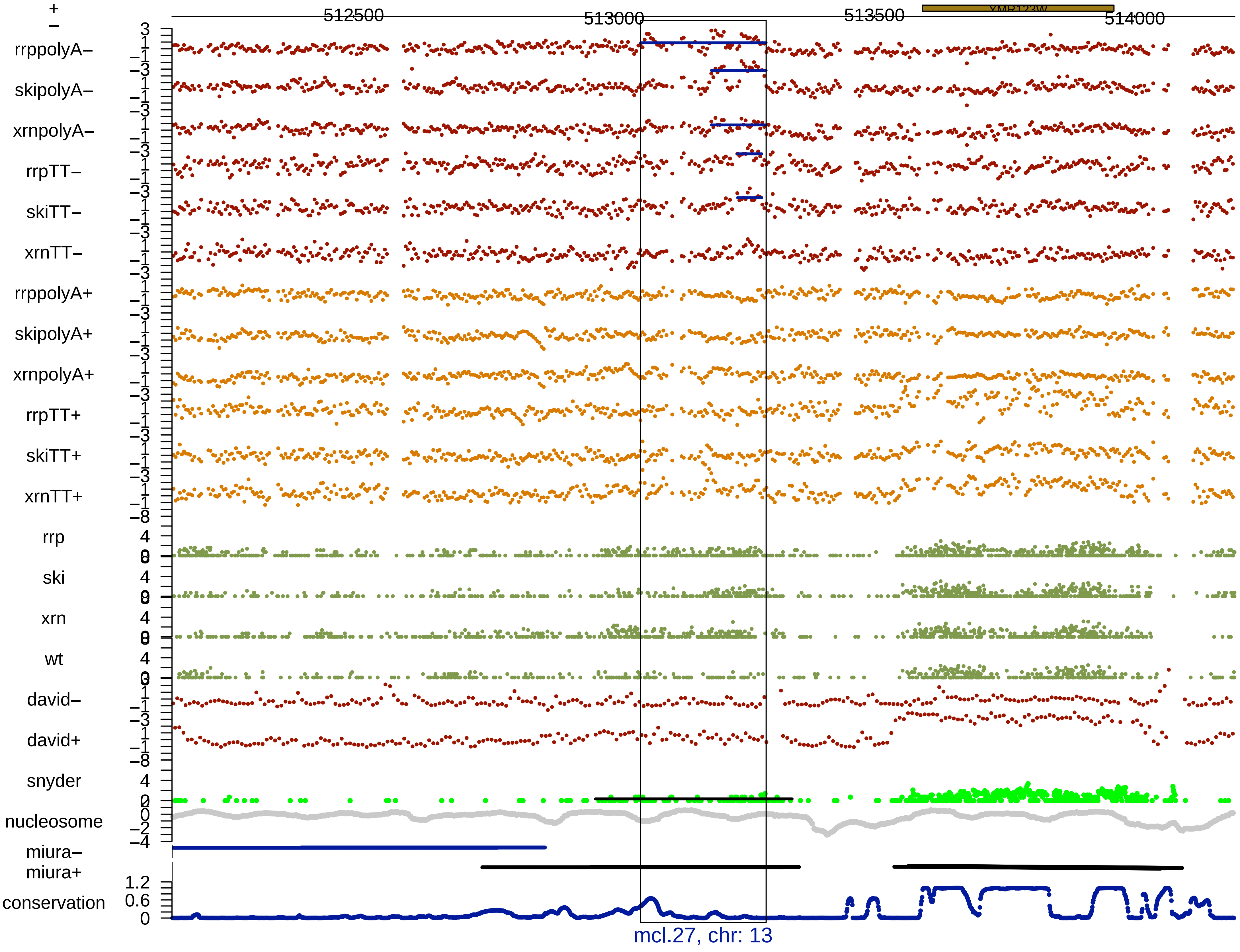

512500
513000
513500
514000
YMR123W
+
−
rrppolyA−
skipolyA−
xrnpolyA−
rrpTT−
skiTT−
xrnTT−
rrppolyA+
skipolyA+
xrnpolyA+
rrpTT+
skiTT+
xrnTT+
rrp
ski
xrn
wt
david−
david+
snyder
nucleosome
miura−
miura+
conservation
mcl.27, chr: 13

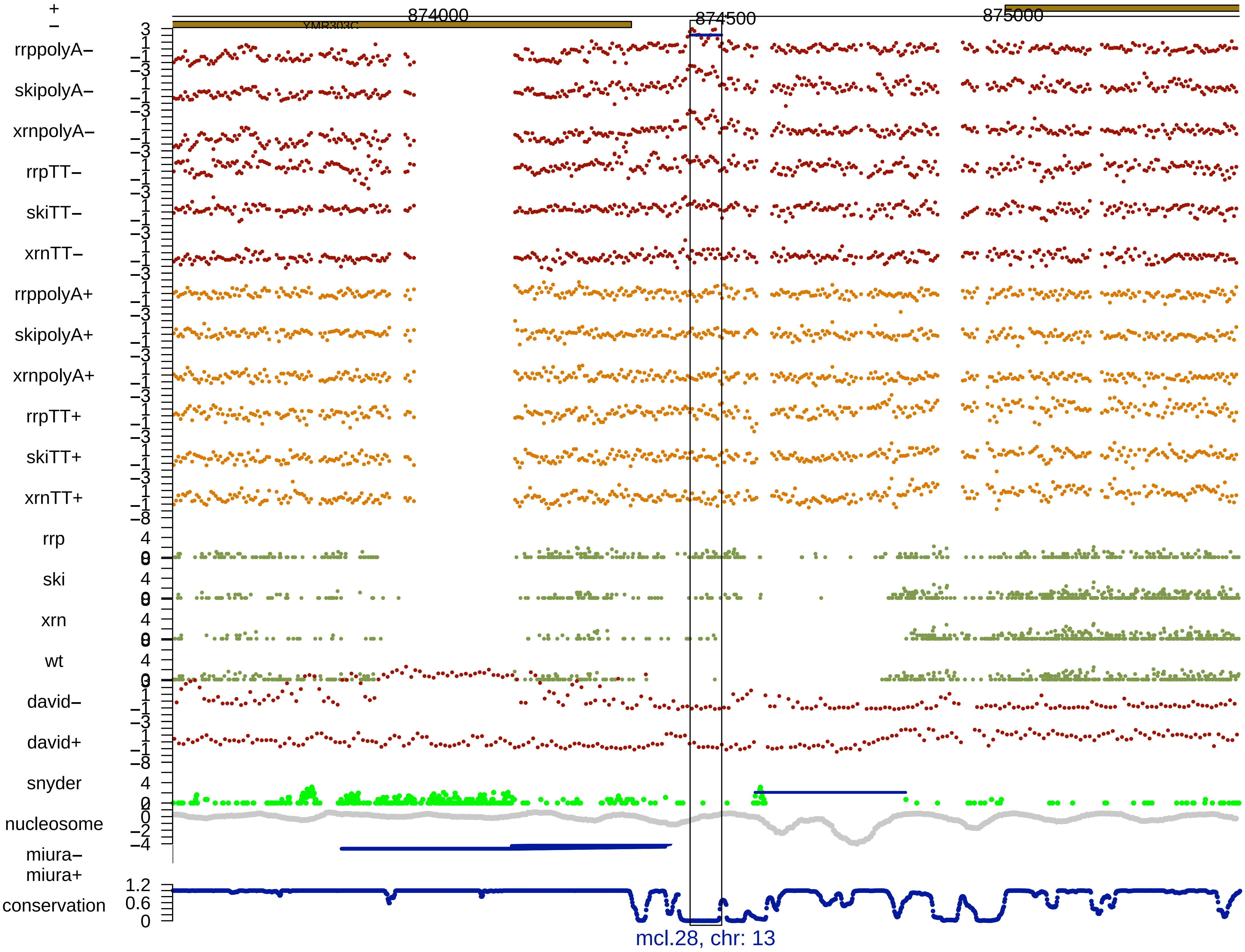

874000
874500
875000
YMR303C
rrppolyA−
skipolyA−
xrnpolyA−
rrpTT−
skiTT−
xrnTT−
rrppolyA+
skipolyA+
xrnpolyA+
rrpTT+
skiTT+
xrnTT+
rrp
ski
xrn
wt
david−
david+
snyder
nucleosome
miura−
miura+
conservation
mcl.28, chr: 13

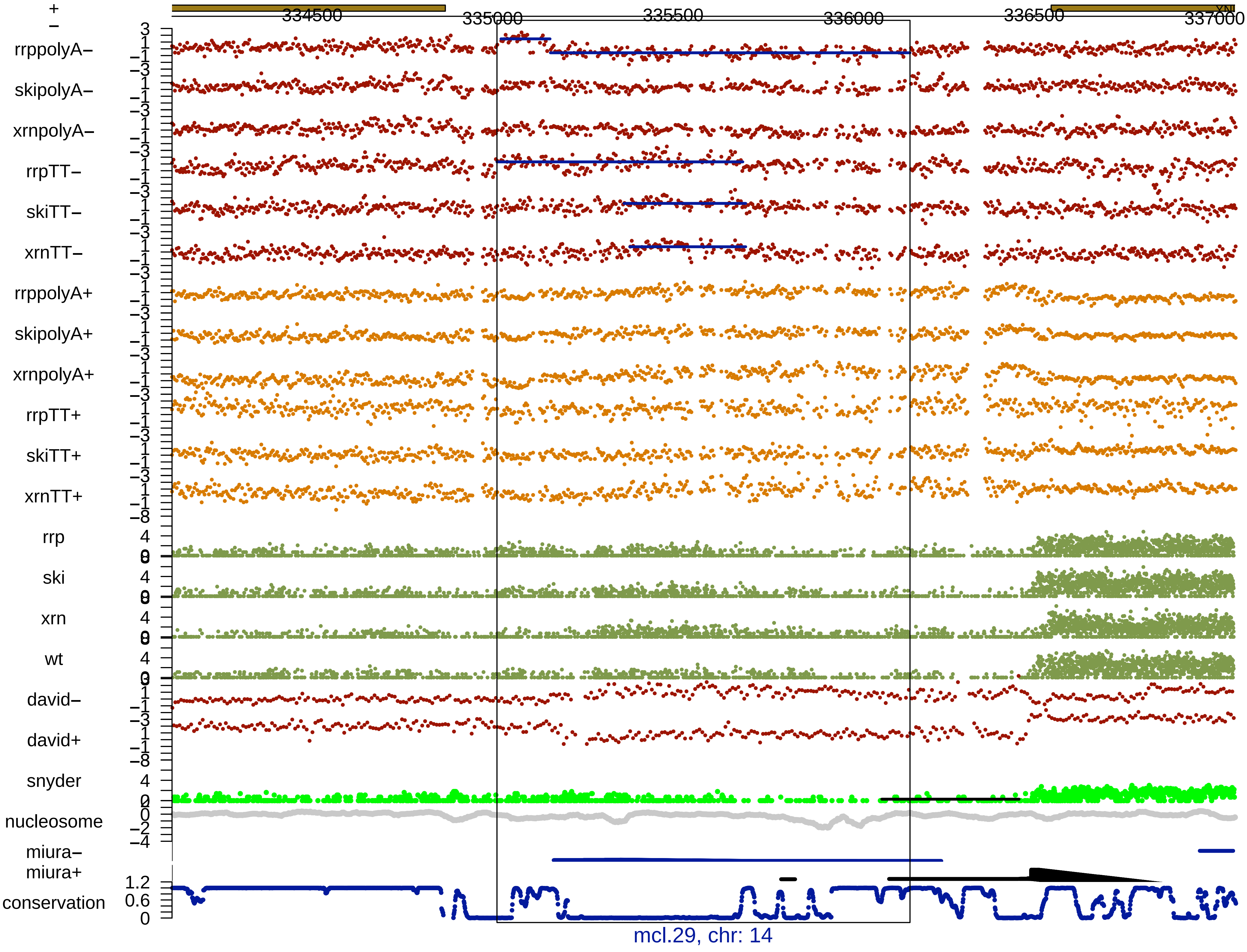

334500
335000
335500
336000
336500
337000
rrppolyA−
skipolyA−
xrnpolyA−
rrpTT−
skiTT−
xrnTT−
rrppolyA+
skipolyA+
xrnpolyA+
rrpTT+
skiTT+
xrnTT+
rrp
ski
xrn
wt
david−
david+
snyder
nucleosome
miura−
miura+
conservation
mcl.29, chr: 14

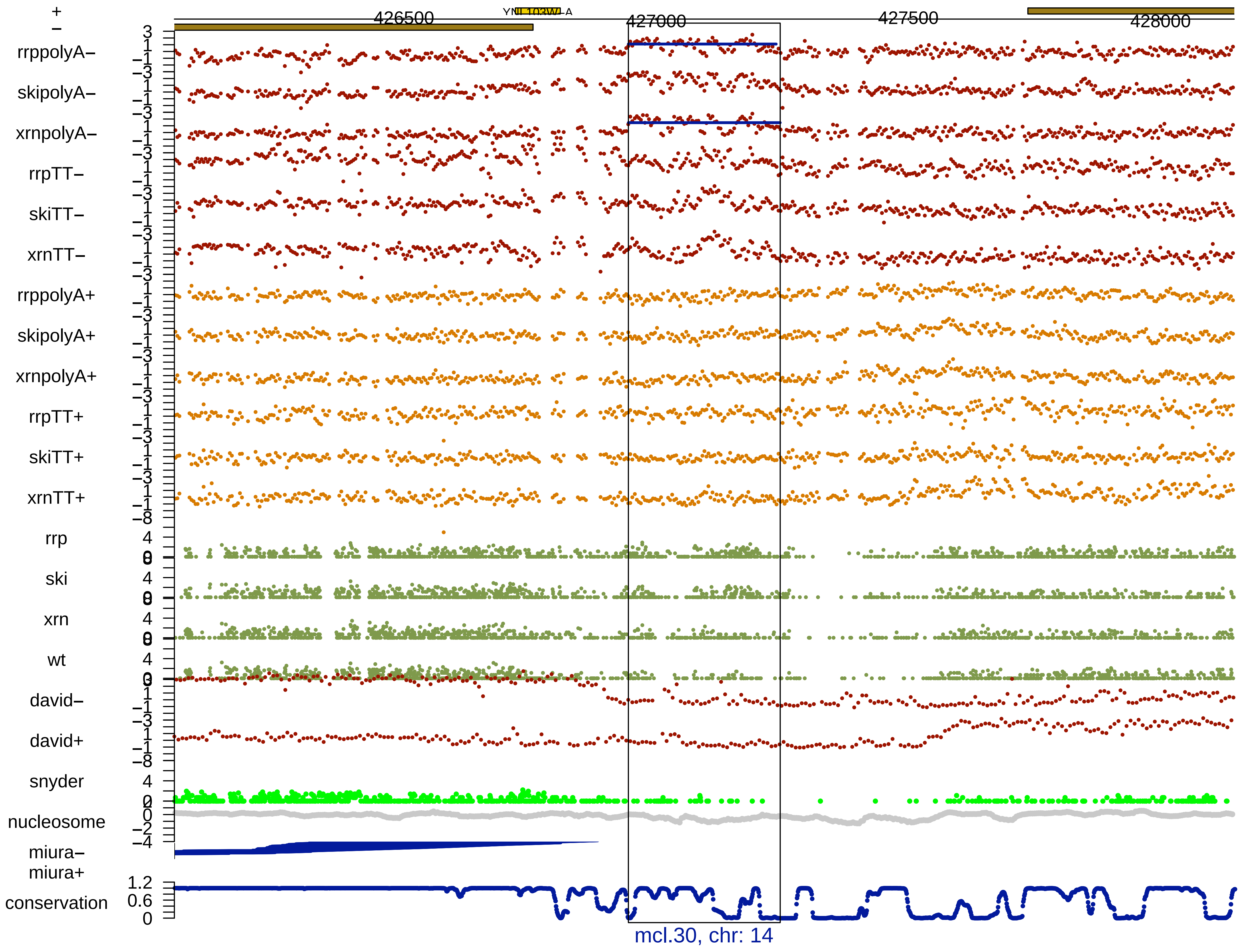
426500
427000
427500
428000
rrppolyA−
skipolyA−
xrnpolyA−
rrpTT−
skiTT−
xrnTT−
rrppolyA+
skipolyA+
xrnpolyA+
rrpTT+
skiTT+
xrnTT+
rrp
ski
xrn
wt
david−
david+
snyder
nucleosome
miura−
miura+
conservation
mcl.30, chr: 14

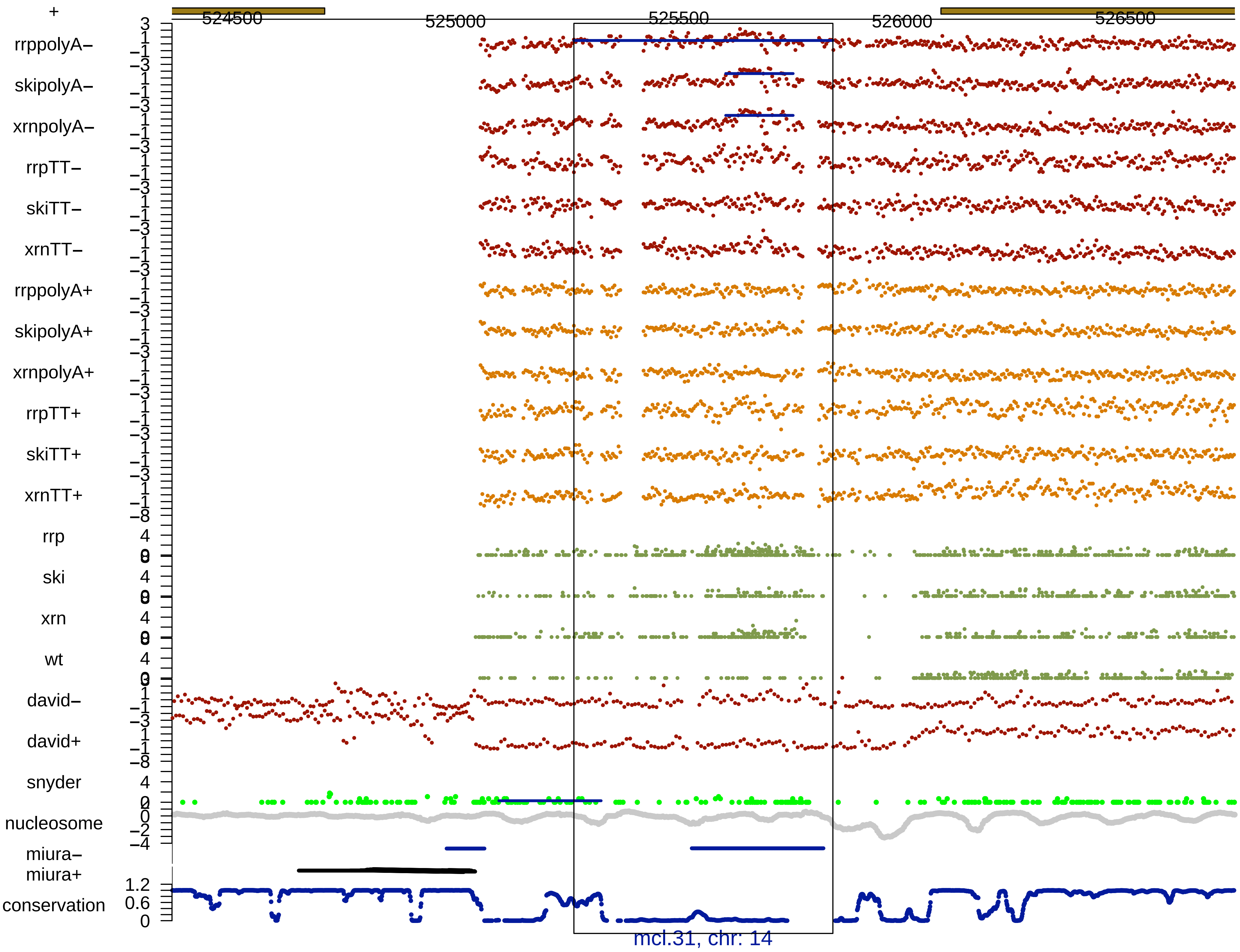

+
524500
525000
525500
526000
526500
rrppolyA−
skipolyA−
xrnpolyA−
rrpTT−
skiTT−
xrnTT−
rrppolyA+
skipolyA+
xrnpolyA+
rrpTT+
skiTT+
xrnTT+
rrp
ski
xrn
wt
david−
david+
snyder
nucleosome
miura−
miura+
conservation
mcl.31, chr: 14

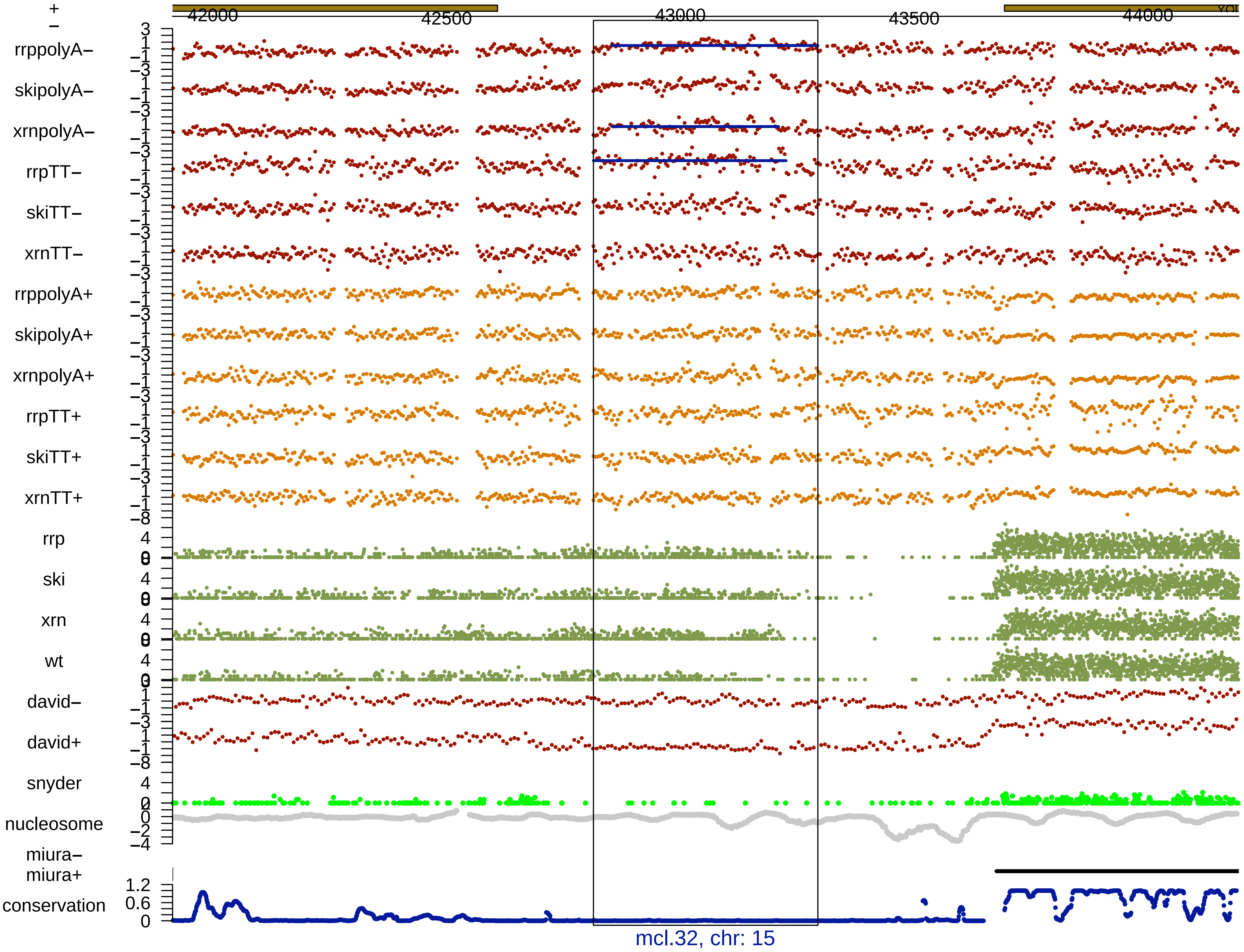

42000
42500
43000
43500
44000
rrppolyA−
skipolyA−
xrnpolyA−
rrpTT−
skiTT−
xrnTT−
rrppolyA+
skipolyA+
xrnpolyA+
rrpTT+
skiTT+
xrnTT+
rrp
ski
xrn
wt
david−
david+
snyder
nucleosome
miura−
miura+
conservation
mcl.32, chr: 15

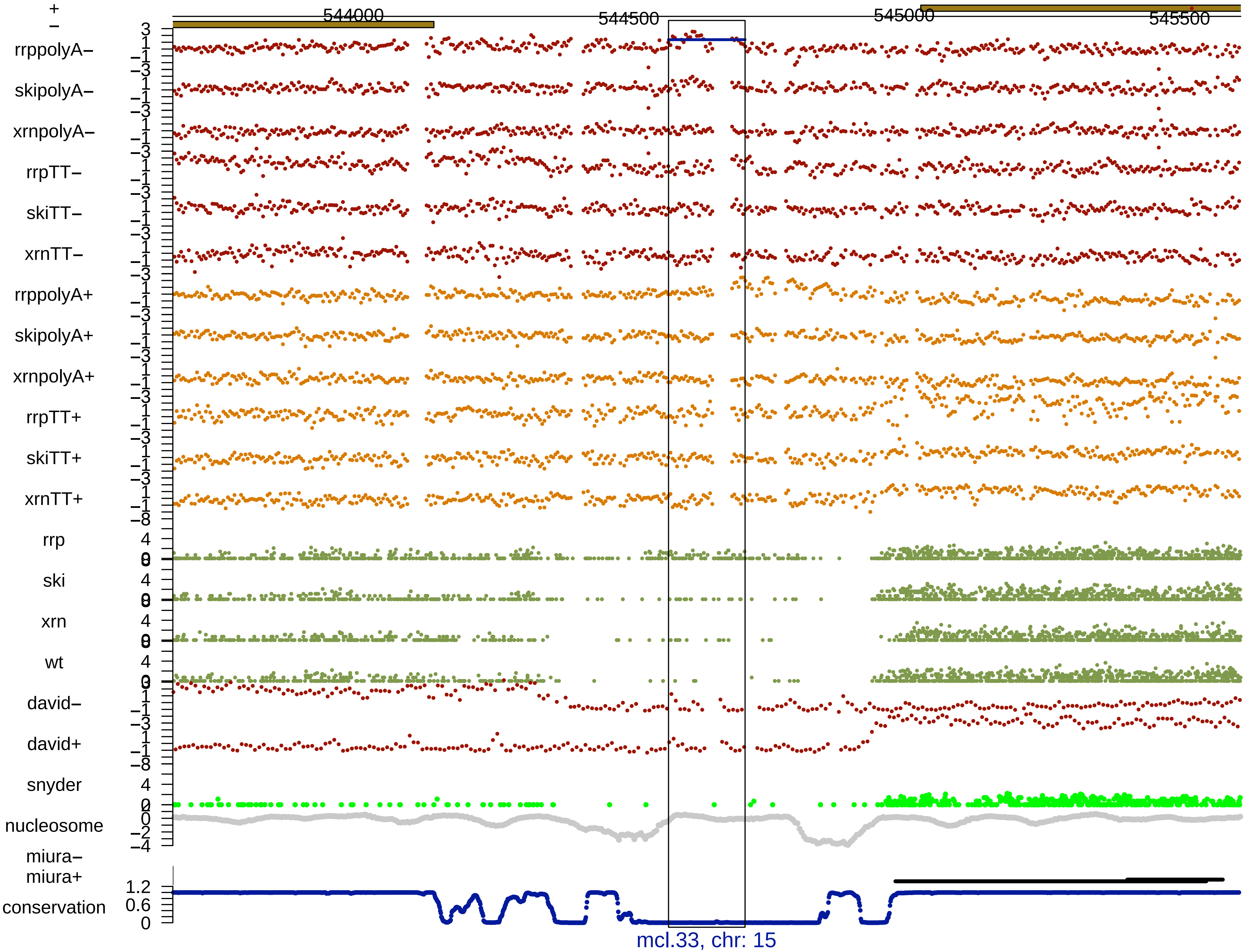

544000
544500
545000
545500
rrppolyA−
skipolyA−
xrnpolyA−
rrpTT−
skiTT−
xrnTT−
rrppolyA+
skipolyA+
xrnpolyA+
rrpTT+
skiTT+
xrnTT+
rrp
ski
xrn
wt
david−
david+
snyder
nucleosome
miura−
miura+
conservation
mcl.33, chr: 15

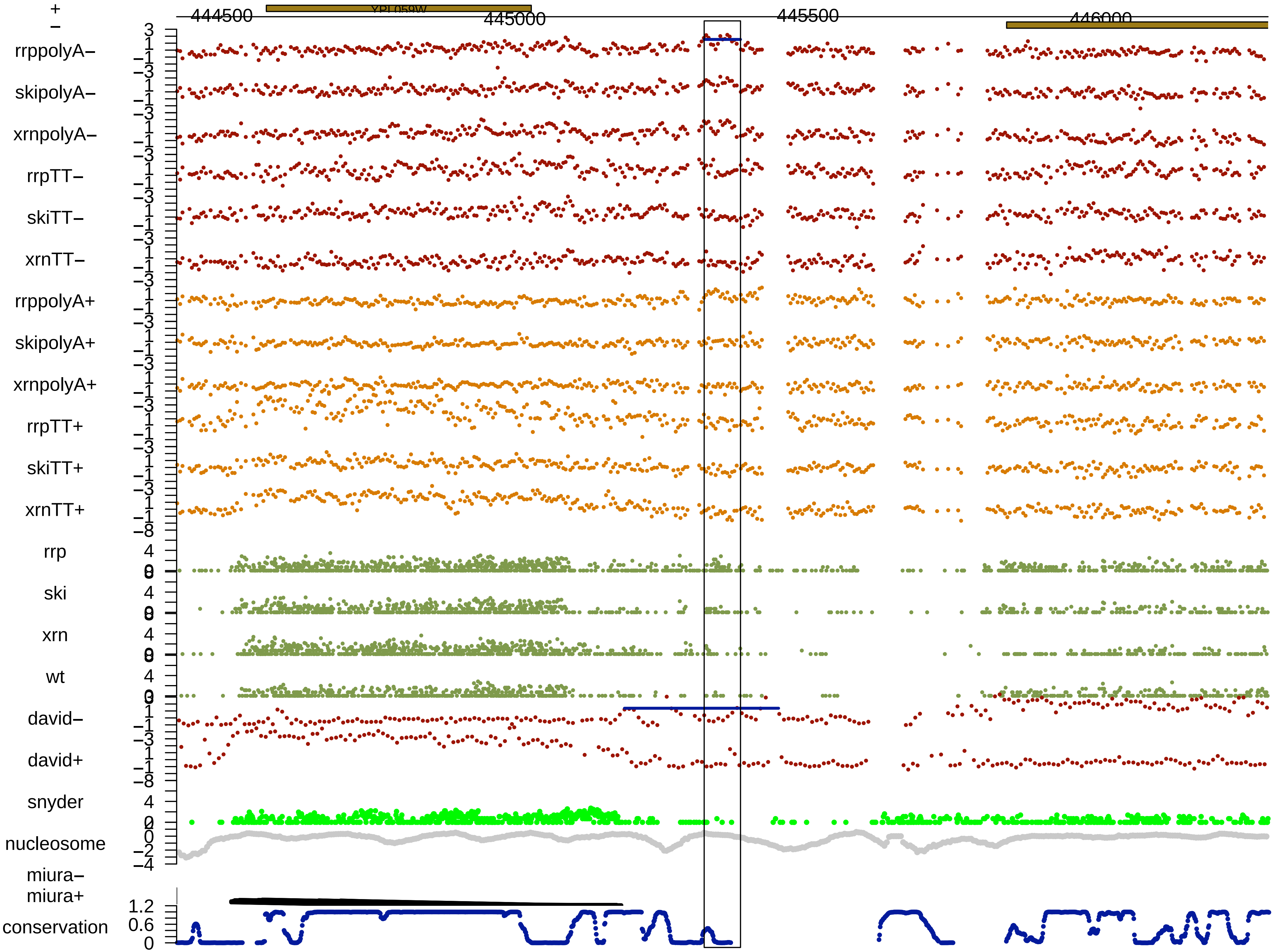
+
−
444500
445000
445500
446000
rrppolyA−
skipolyA−
xrnpolyA−
rrpTT−
skiTT−
xrnTT−
rrppolyA+
skipolyA+
xrnpolyA+
rrpTT+
skiTT+
xrnTT+
rrp
ski
xrn
wt
david−
david+
snyder
nucleosome
miura−
miura+
conservation
1.2
0.6
0

mcl.34, chr: 16

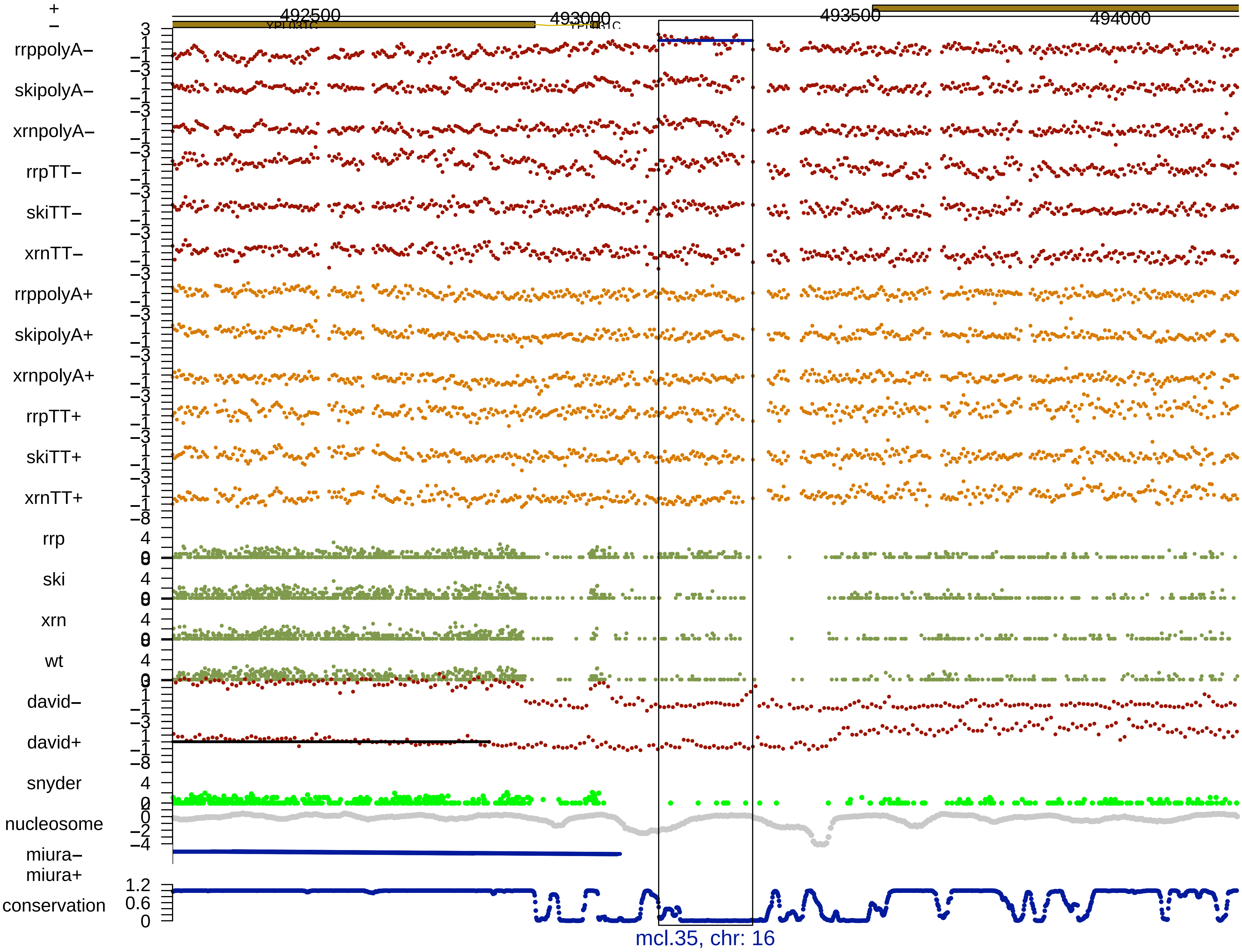

+
−
492500
493000
493500
494000
YPL031C
rrppolyA−
skipolyA−
xrnpolyA−
rrpTT−
skiTT−
xrnTT−
rrppolyA+
skipolyA+
xrnpolyA+
rrpTT+
skiTT+
xrnTT+
rrp
ski
xrn
wt
david−
david+
snyder
nucleosome
miura−
miura+
conservation
mcl.35, chr: 16

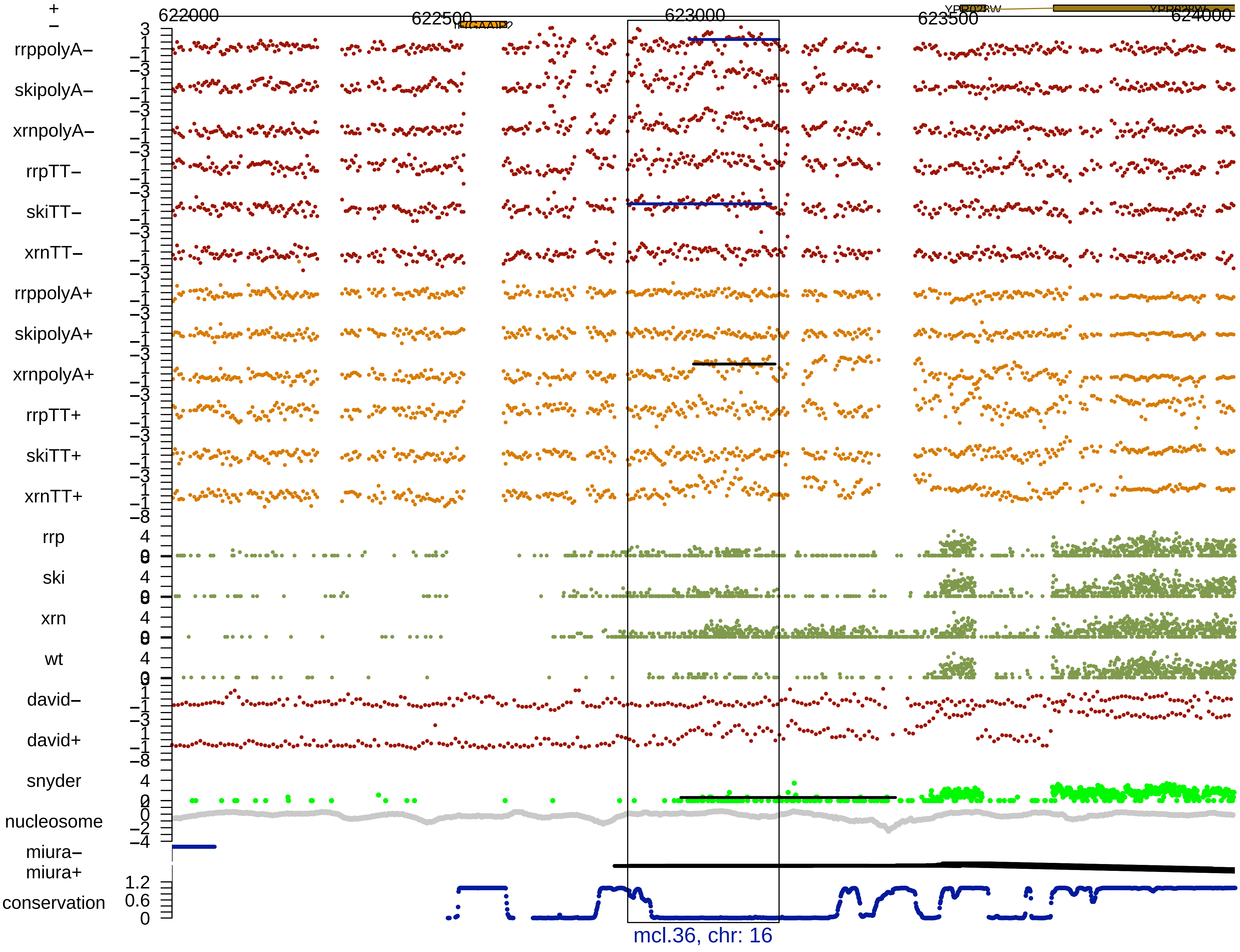

622000
622500
623000
623500
624000
rrppolyA−
skipolyA−
xrnpolyA−
rrpTT−
skiTT−
xrnTT−
rrppolyA+
skipolyA+
xrnpolyA+
rrpTT+
skiTT+
xrnTT+
rrp
ski
xrn
wt
david−
david+
snyder
nucleosome
miura−
miura+
conservation
mcl.36, chr: 16

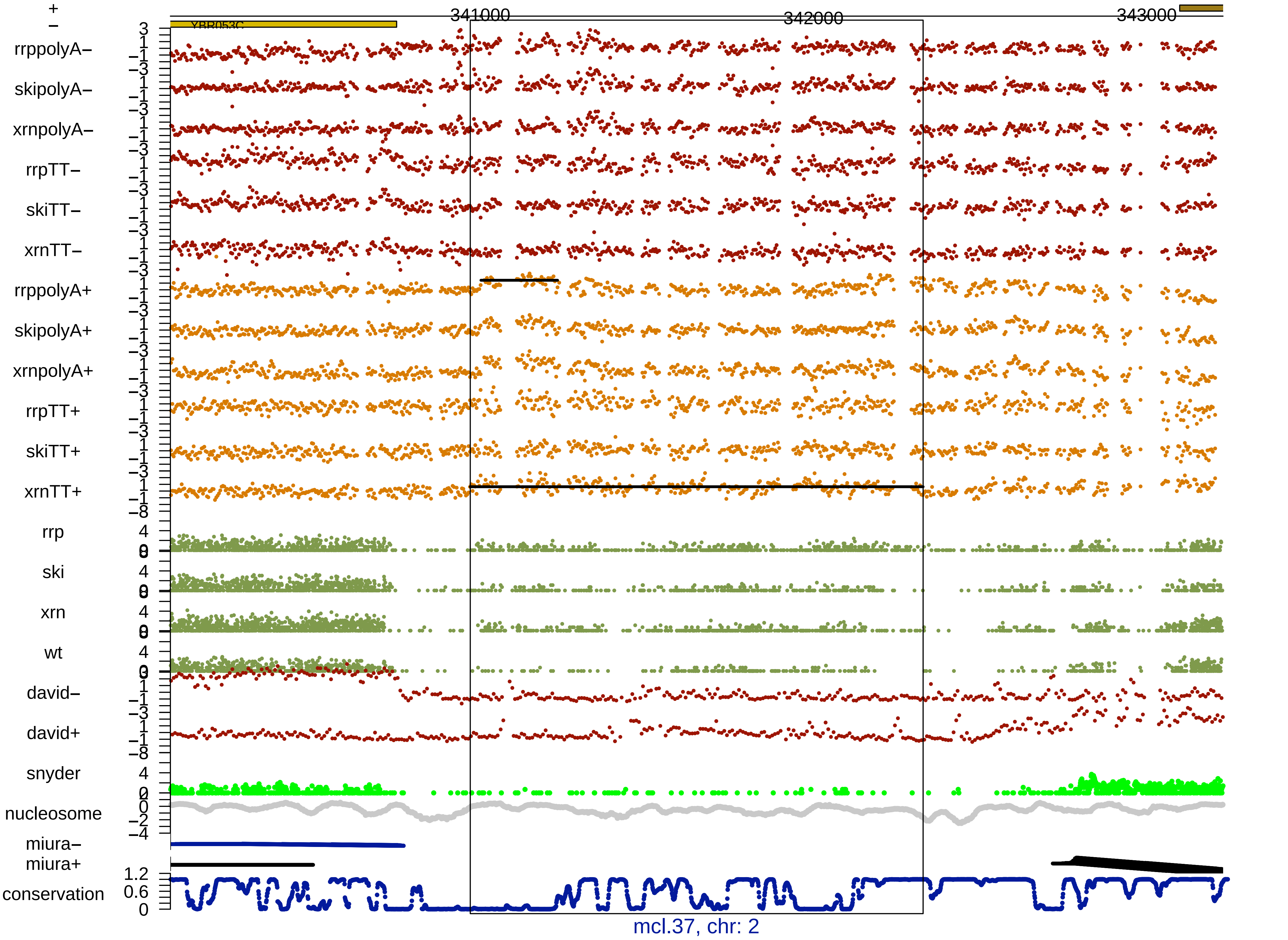

+
−
YBR053C
341000
342000
343000
rrppolyA−
skipolyA−
xrnpolyA−
rrpTT−
skiTT−
xrnTT−
rrppolyA+
skipolyA+
xrnpolyA+
rrpTT+
skiTT+
xrnTT+
rrp
ski
xrn
wt
david−
david+
snyder
nucleosome
miura−
miura+
conservation
mcl.37, chr: 2

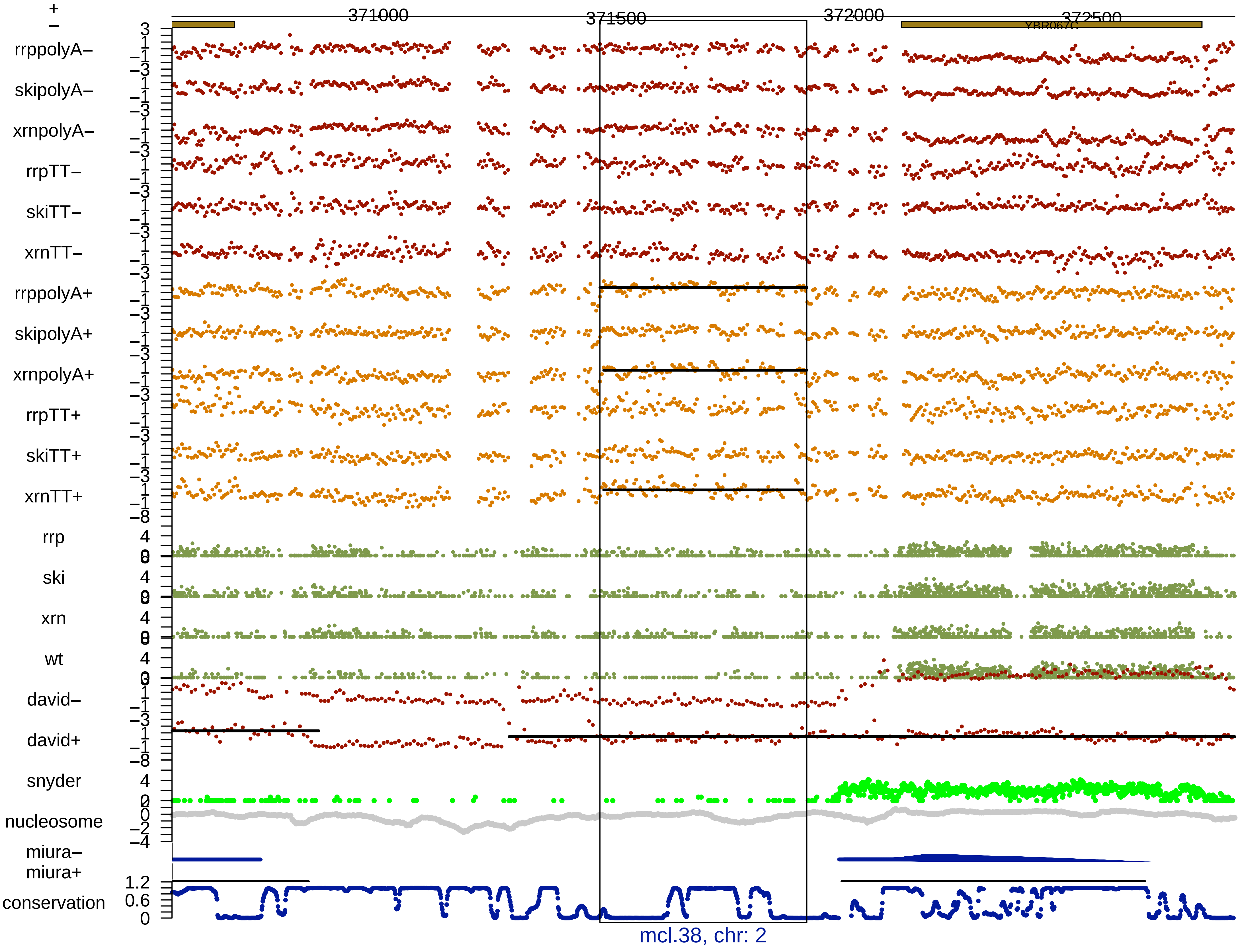
+
−
371000
371500
372000
372500
YBR067C
rrppolyA−
skipolyA−
xrnpolyA−
rrpTT−
skiTT−
xrnTT−
rrppolyA+
skipolyA+
xrnpolyA+
rrpTT+
skiTT+
xrnTT+
rrp
ski
xrn
wt
david−
david+
snyder
nucleosome
miura−
miura+
conservation
mcl.38, chr: 2

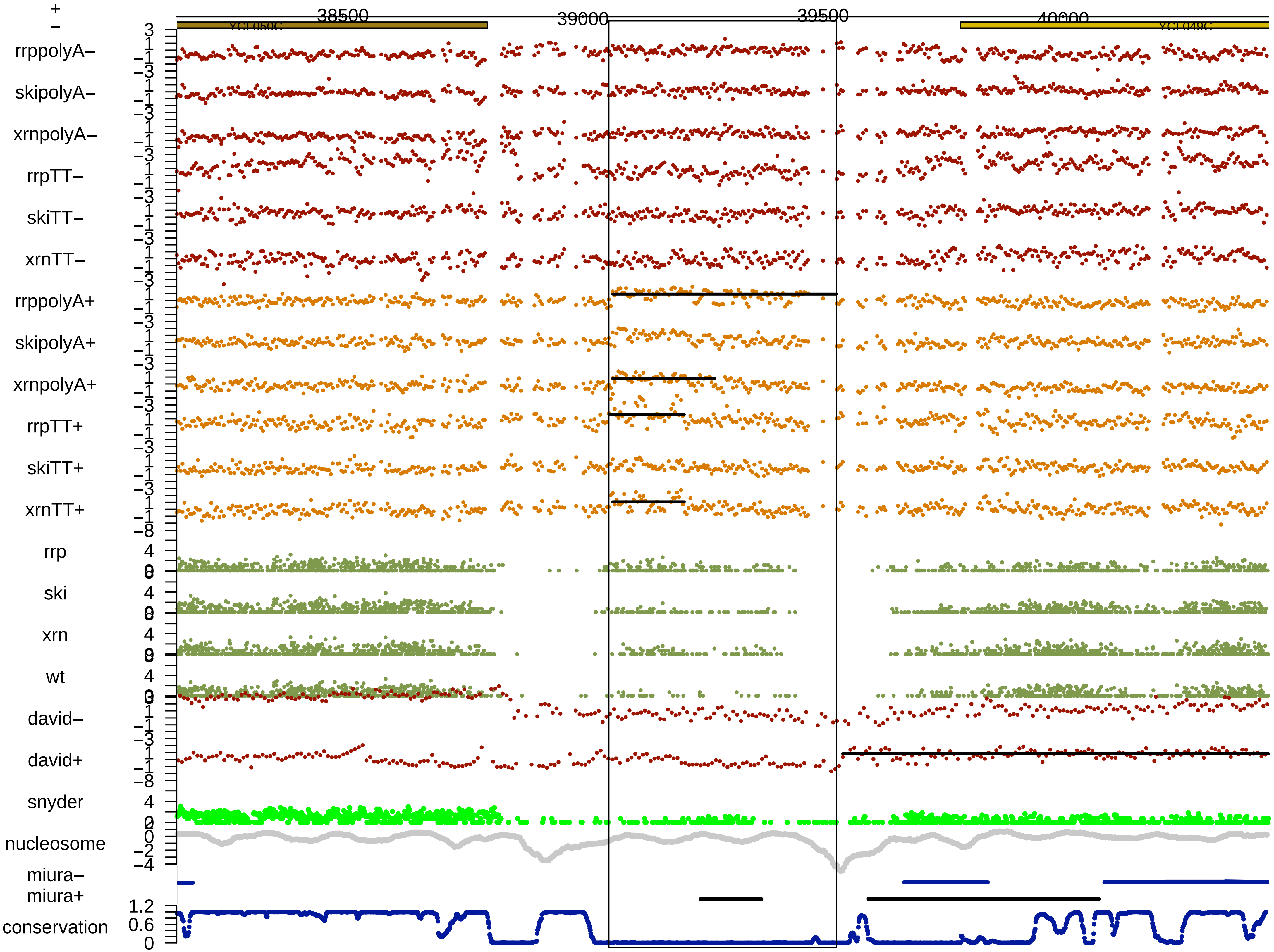
38500
39000
39500
40000
+
−
rrppolyA−
skipolyA−
xrnpolyA−
rrpTT−
skiTT−
xrnTT−
rrppolyA+
skipolyA+
xrnpolyA+
rrpTT+
skiTT+
xrnTT+
rrp
ski
xrn
wt
david−
david+
snyder
nucleosome
miura−
miura+
conservation
3
1
−1
−3
−8
4
0
2
−2
−4
1.2
0.6

mcl.40, chr: 3

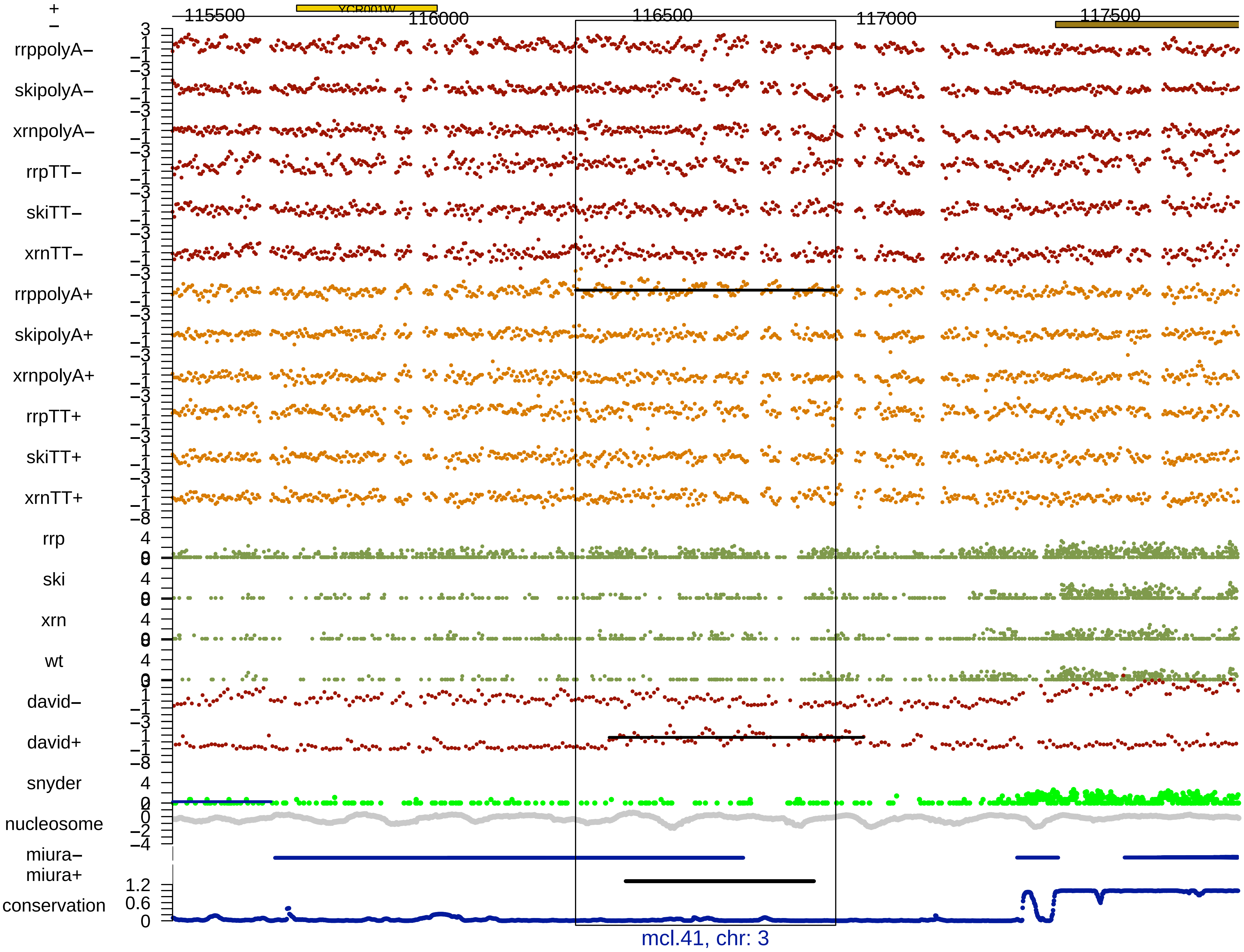
YCR001W
115500
116000
116500
117000
117500
rrppolyA−
skipolyA−
xrnpolyA−
rrpTT−
skiTT−
xrnTT−
rrppolyA+
skipolyA+
xrnpolyA+
rrpTT+
skiTT+
xrnTT+
rrp
ski
xrn
wt
david−
david+
snyder
nucleosome
miura−
miura+
conservation
mcl.41, chr: 3

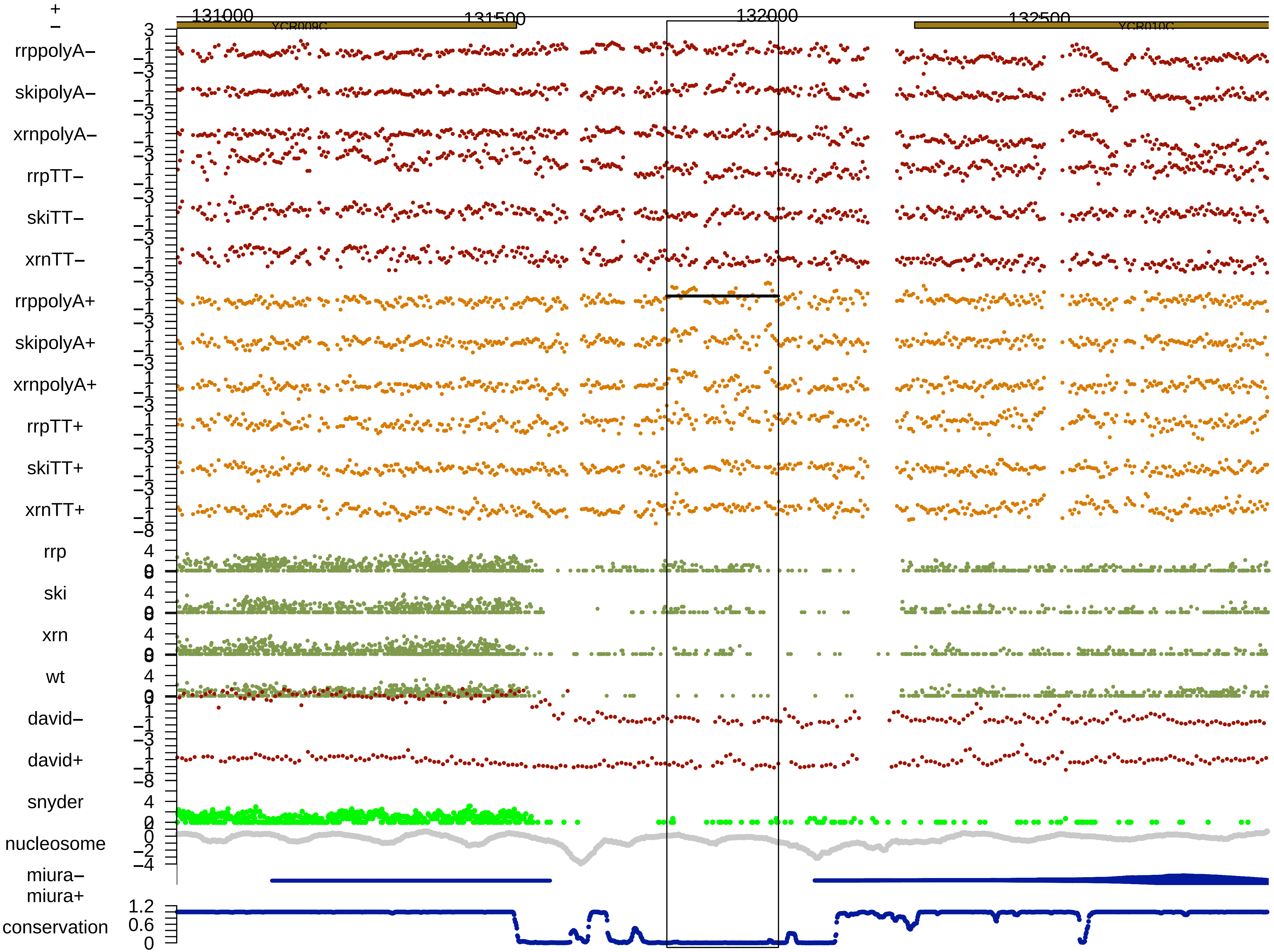

131000
131500
132000
132500
YCR009C
YCR010C
+
−
rrppolyA−
skipolyA−
xrnpolyA−
rrpTT−
skiTT−
xrnTT−
rrppolyA+
skipolyA+
xrnpolyA+
rrpTT+
skiTT+
xrnTT+
rrp
ski
xrn
wt
david−
david+
snyder
nucleosome
miura−
miura+
conservation
3
1
−1
−3
−8
4
0
2
−2
−4
1.2
0.6

mcl.42, chr: 3

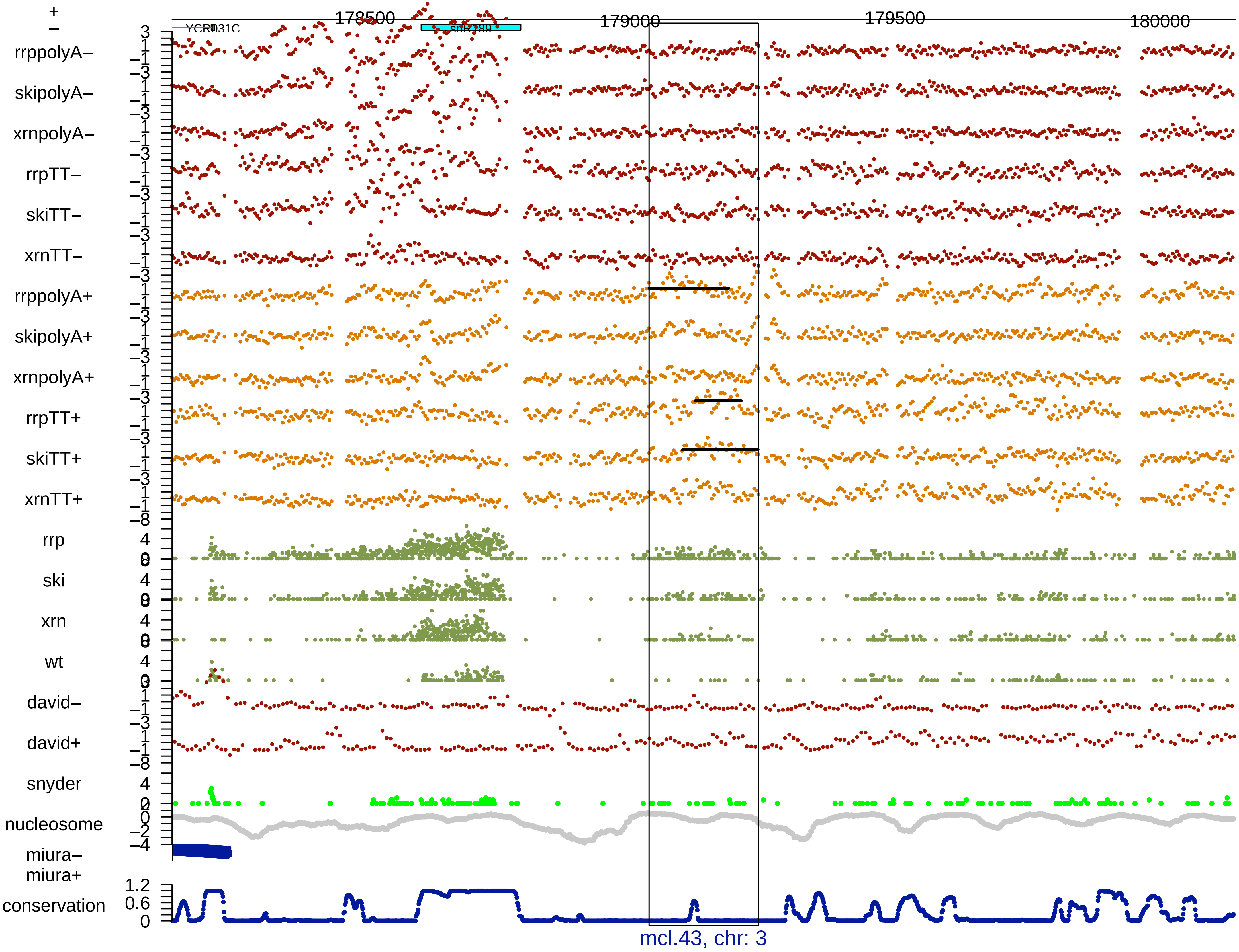

+
−
178500
179000
179500
180000
YCR031C
snR189
rrppolyA−
skipolyA−
xrnpolyA−
rrpTT−
skiTT−
xrnTT−
rrppolyA+
skipolyA+
xrnpolyA+
rrpTT+
skiTT+
xrnTT+
rrp
ski
xrn
wt
david−
david+
snyder
nucleosome
miura−
miura+
conservation
mcl.43, chr: 3

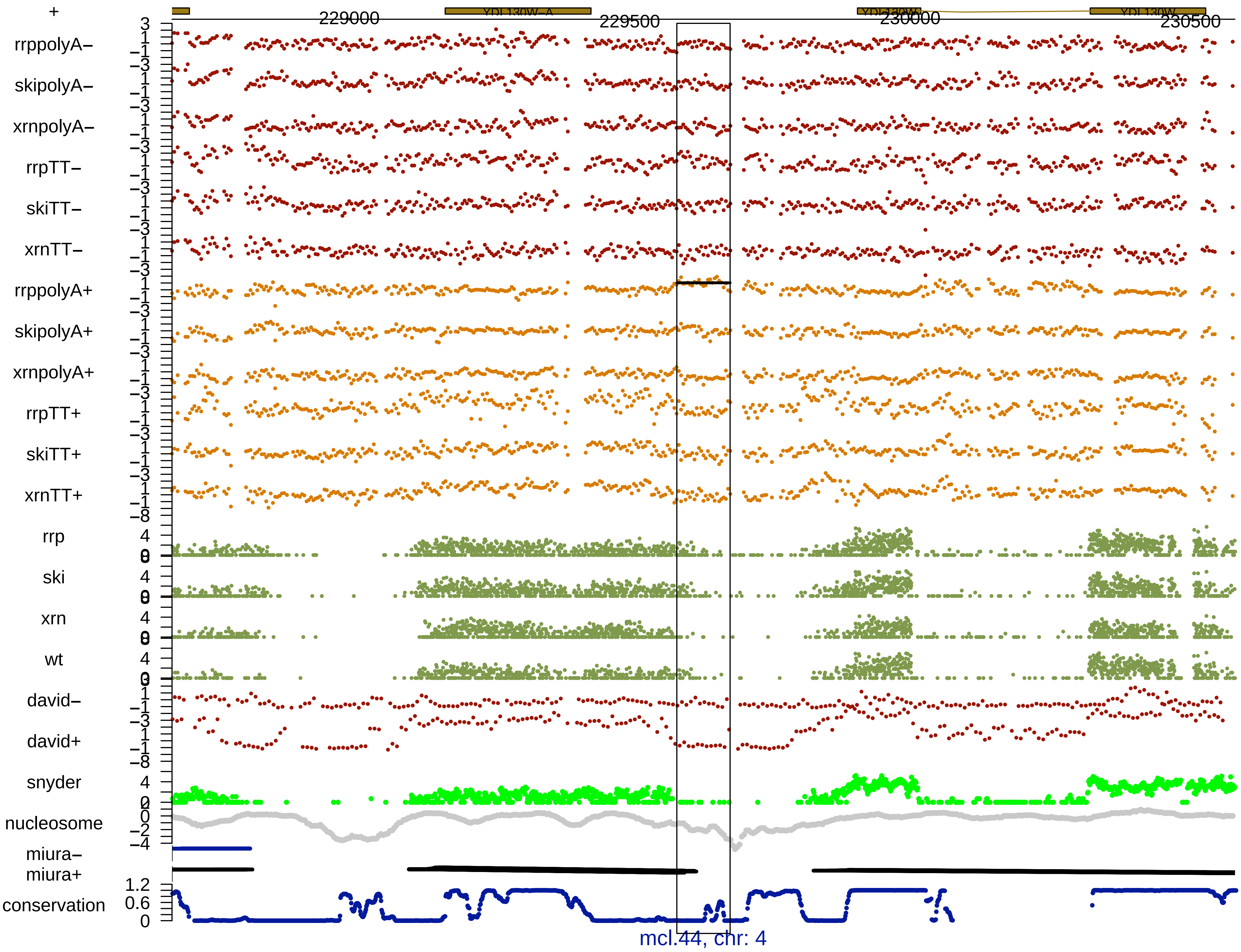

+
229000
229500
230000
230500
YDL130W-A
YDL130W
rrppolyA−
skipolyA−
xrnpolyA−
rrpTT−
skiTT−
xrnTT−
rrppolyA+
skipolyA+
xrnpolyA+
rrpTT+
skiTT+
xrnTT+
rrp
ski
xrn
wt
david−
david+
snyder
nucleosome
miura−
miura+
conservation
mcl.44, chr: 4

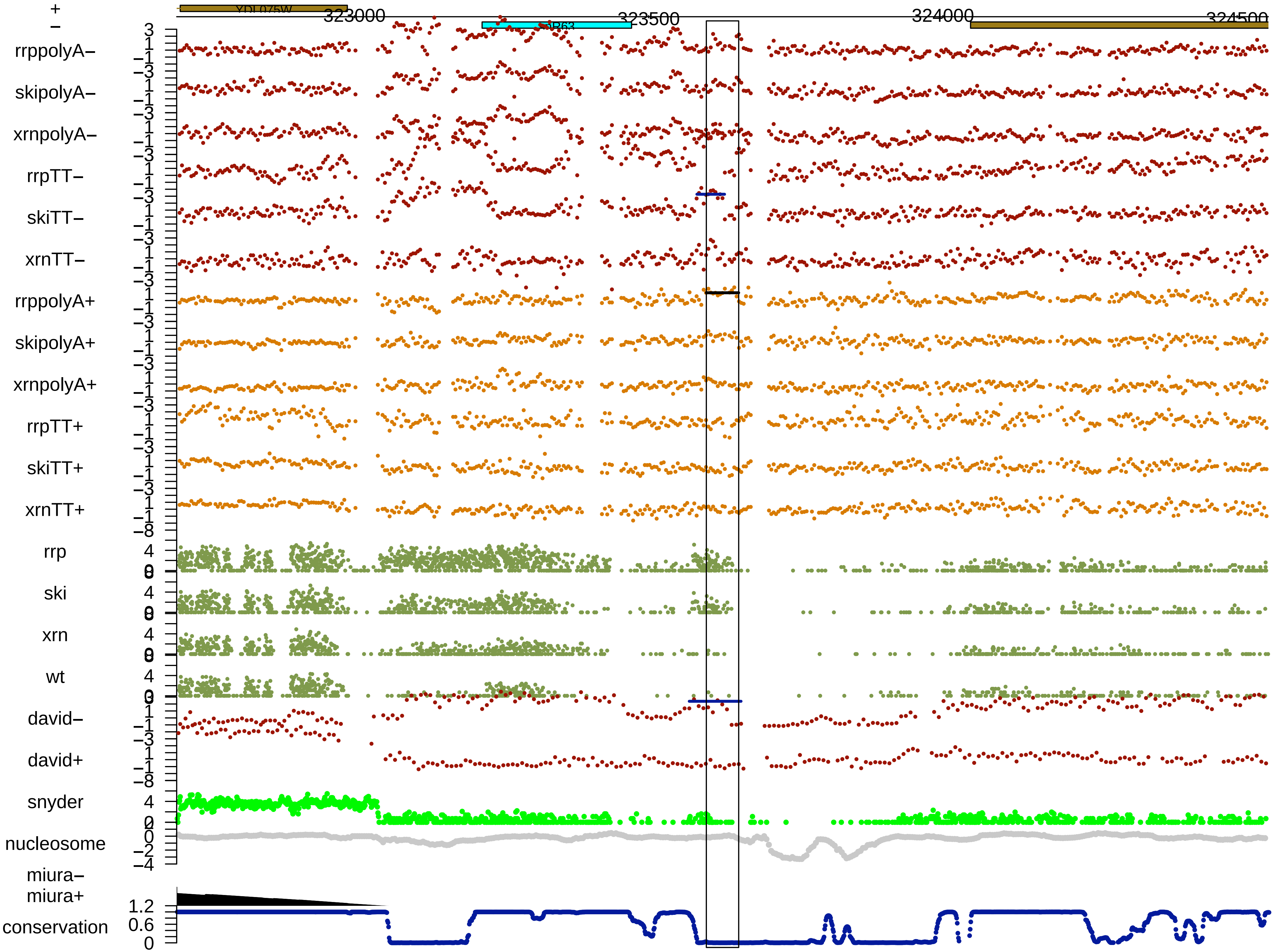
+
–
323000
323500
324000
324500
rrppolyA–
skipolyA–
xrnpolyA–
rrpTT–
skiTT–
xrnTT–
rrppolyA+
skipolyA+
xrnpolyA+
rrpTT+
skiTT+
xrnTT+
rrp
ski
xrn
wt
david–
david+
snyder
nucleosome
miura–
miura+
conservation
1.2
0.6
0
mcl.45, chr: 4

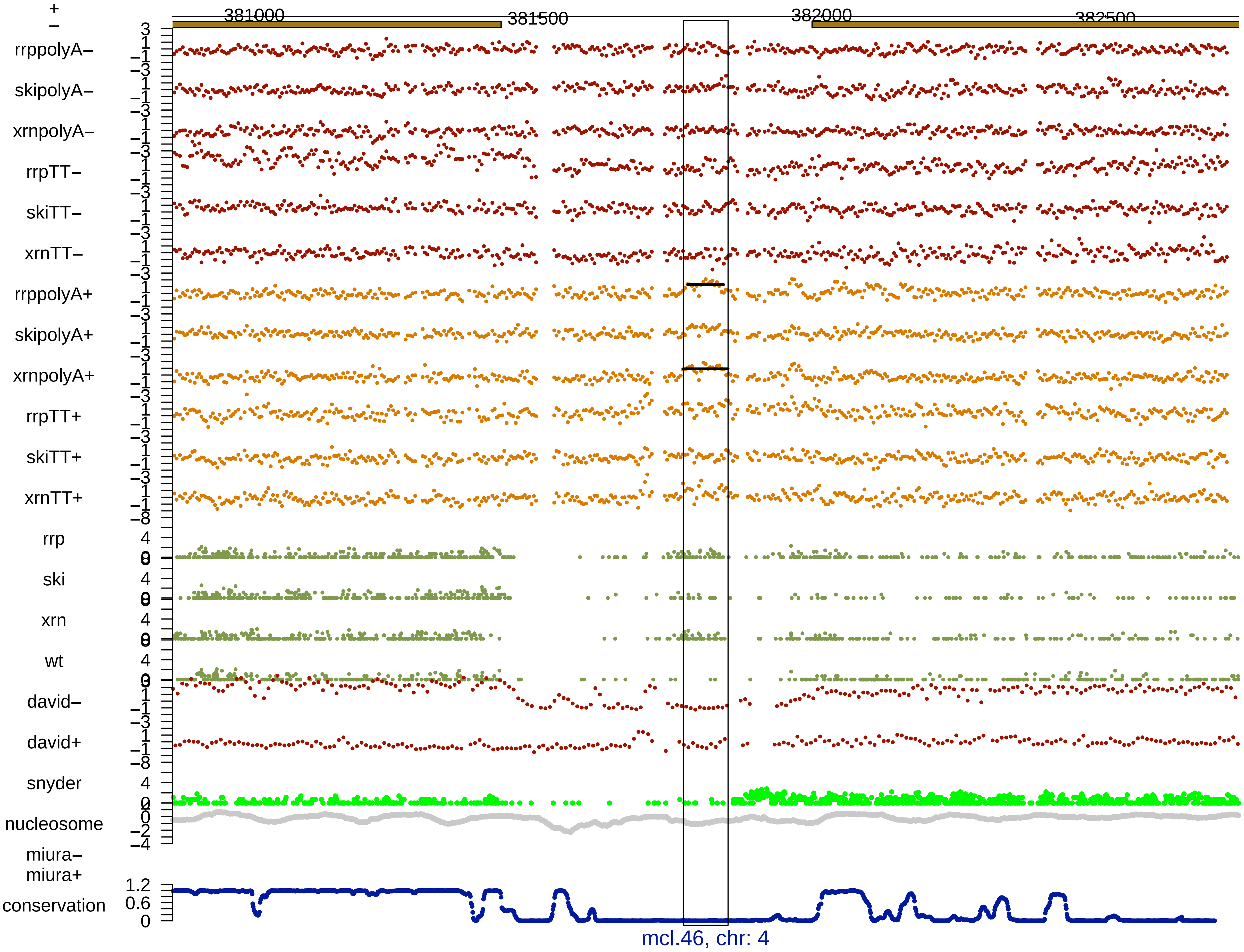
381000
381500
382000
382500
+
–
rrppolyA–
skipolyA–
xrnpolyA–
rrpTT–
skiTT–
xrnTT–
rrppolyA+
skipolyA+
xrnpolyA+
rrpTT+
skiTT+
xrnTT+
rrp
ski
xrn
wt
david–
david+
snyder
nucleosome
miura–
miura+
conservation
mcl.46, chr: 4

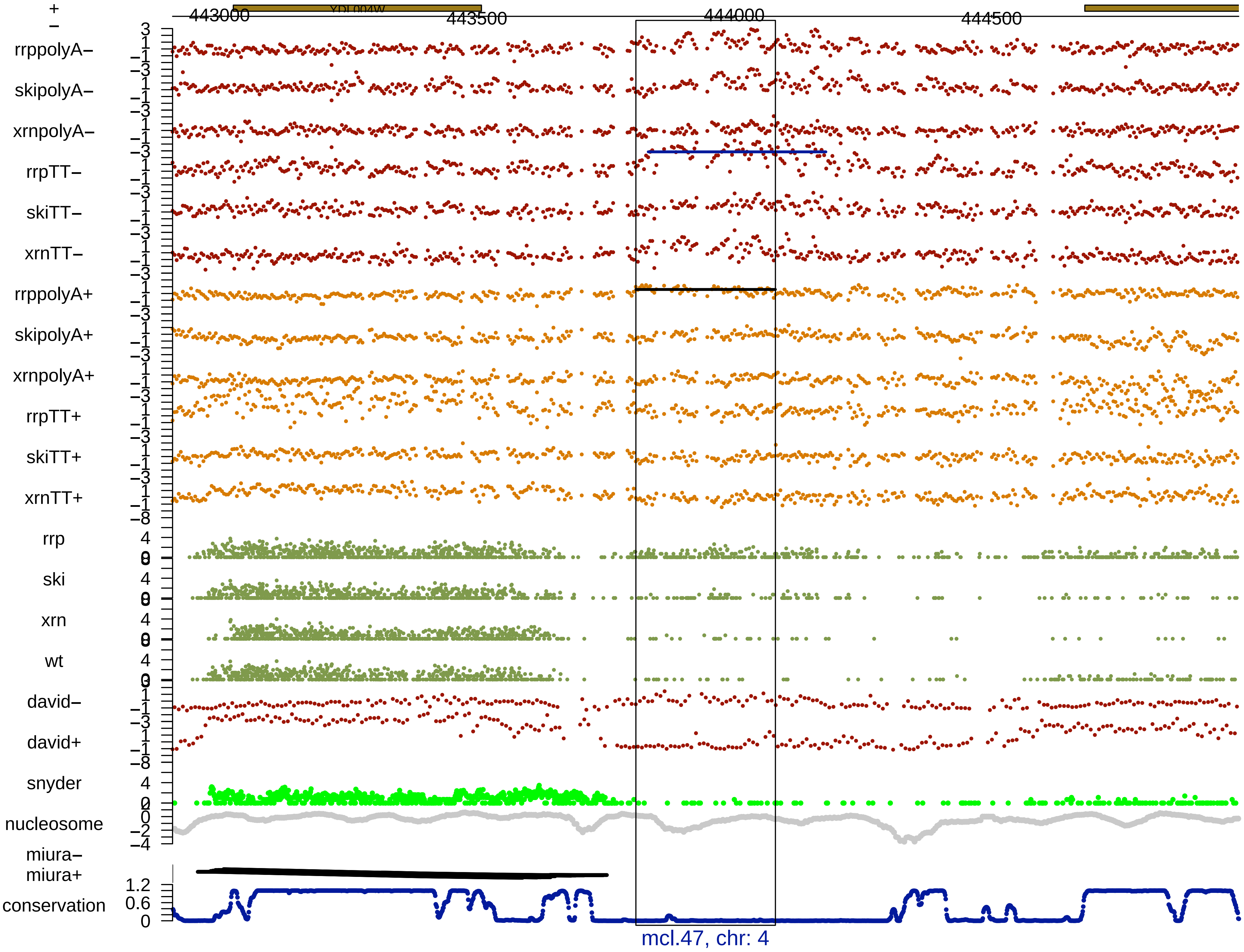
YDL004W
443000
443500
444000
444500
rrppolyA−
skipolyA−
xrnpolyA−
rrpTT−
skiTT−
xrnTT−
rrppolyA+
skipolyA+
xrnpolyA+
rrpTT+
skiTT+
xrnTT+
rrp
ski
xrn
wt
david−
david+
snyder
nucleosome
miura−
miura+
conservation
mcl.47, chr: 4

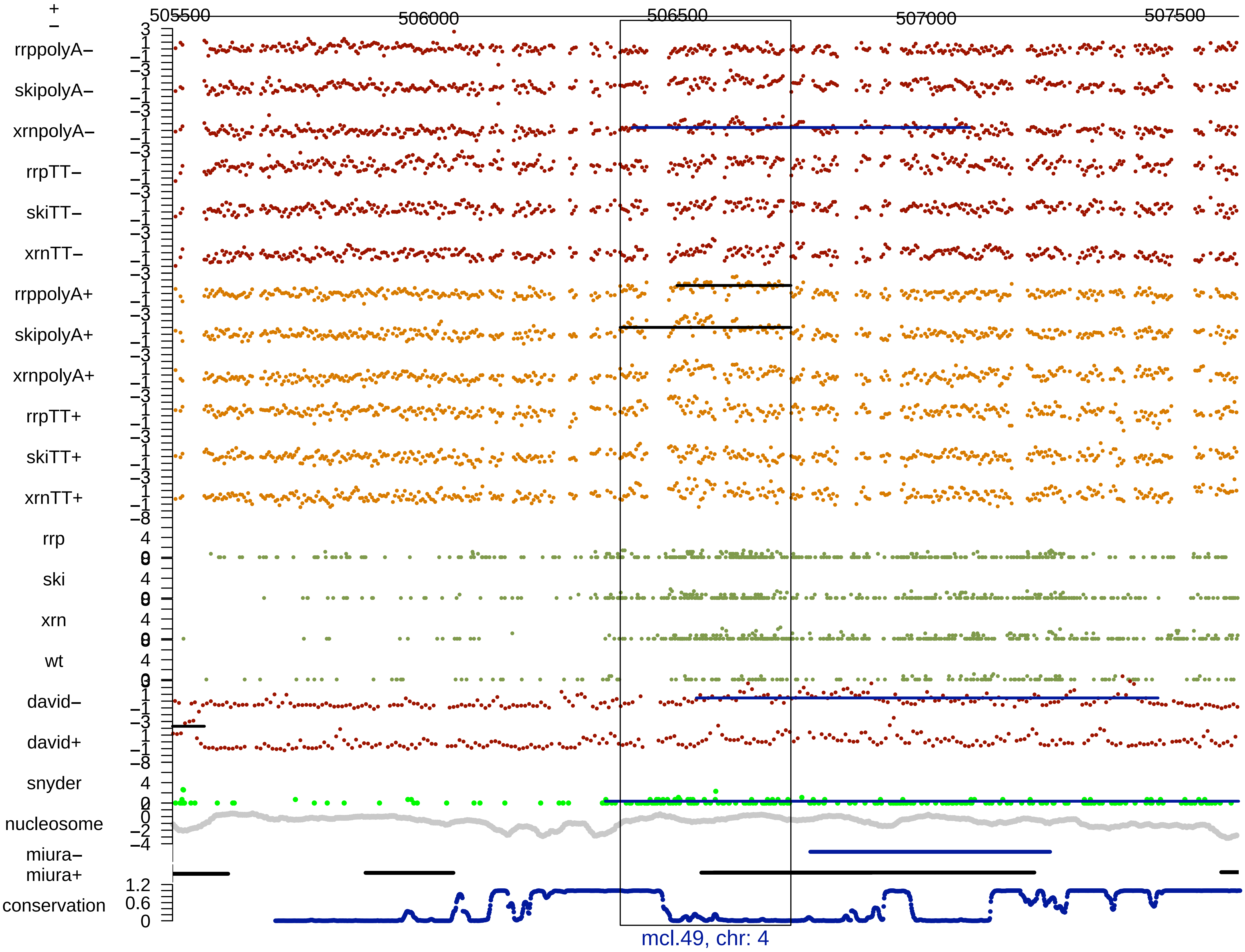

505500
506000
506500
507000
507500
rrppolyA−
skipolyA−
xrnpolyA−
rrpTT−
skiTT−
xrnTT−
rrppolyA+
skipolyA+
xrnpolyA+
rrpTT+
skiTT+
xrnTT+
rrp
ski
xrn
wt
david−
david+
snyder
nucleosome
miura−
miura+
conservation
mcl.49, chr: 4

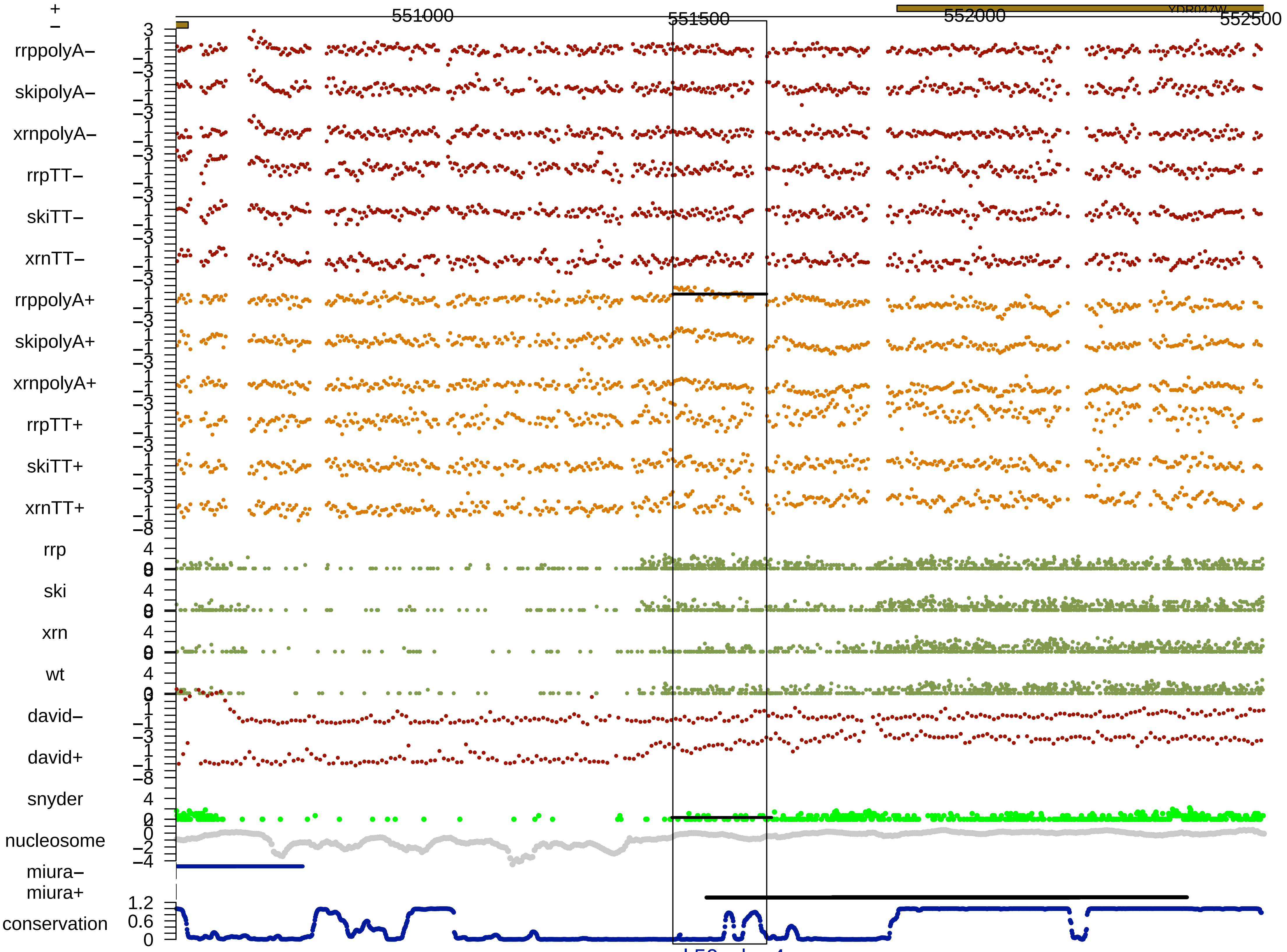

+
−
551000
551500
552000
552500
YDR047W
rrppolyA−
skipolyA−
xrnpolyA−
rrpTT−
skiTT−
xrnTT−
rrppolyA+
skipolyA+
xrnpolyA+
rrpTT+
skiTT+
xrnTT+
rrp
ski
xrn
wt
david−
david+
snyder
nucleosome
miura−
miura+
conservation
mcl.50, chr: 4

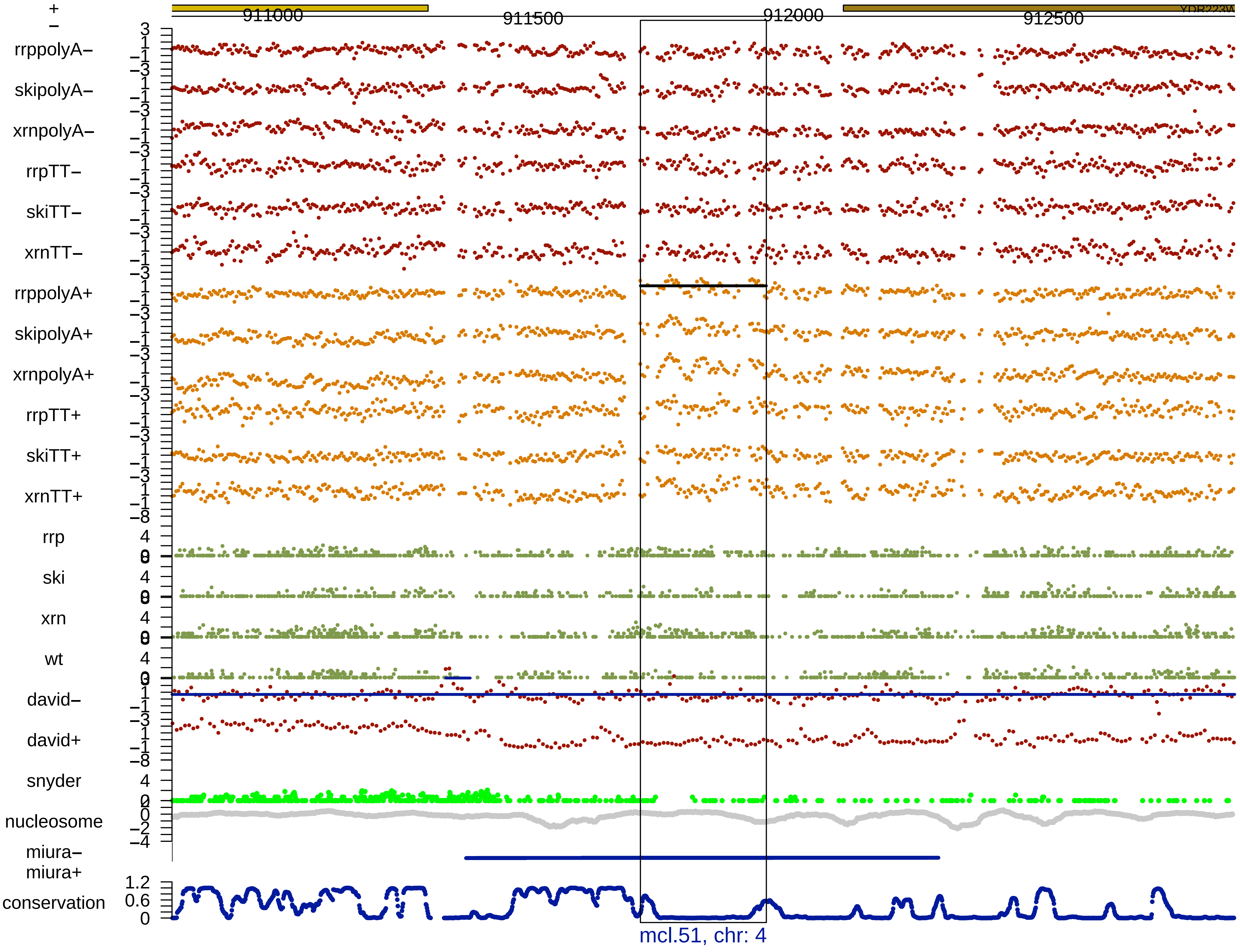

+
−
911000
911500
912000
912500
YDR223W
rrppolyA−
skipolyA−
xrnpolyA−
rrpTT−
skiTT−
xrnTT−
rrppolyA+
skipolyA+
xrnpolyA+
rrpTT+
skiTT+
xrnTT+
rrp
ski
xrn
wt
david−
david+
snyder
nucleosome
miura−
miura+
conservation
mcl.51, chr: 4

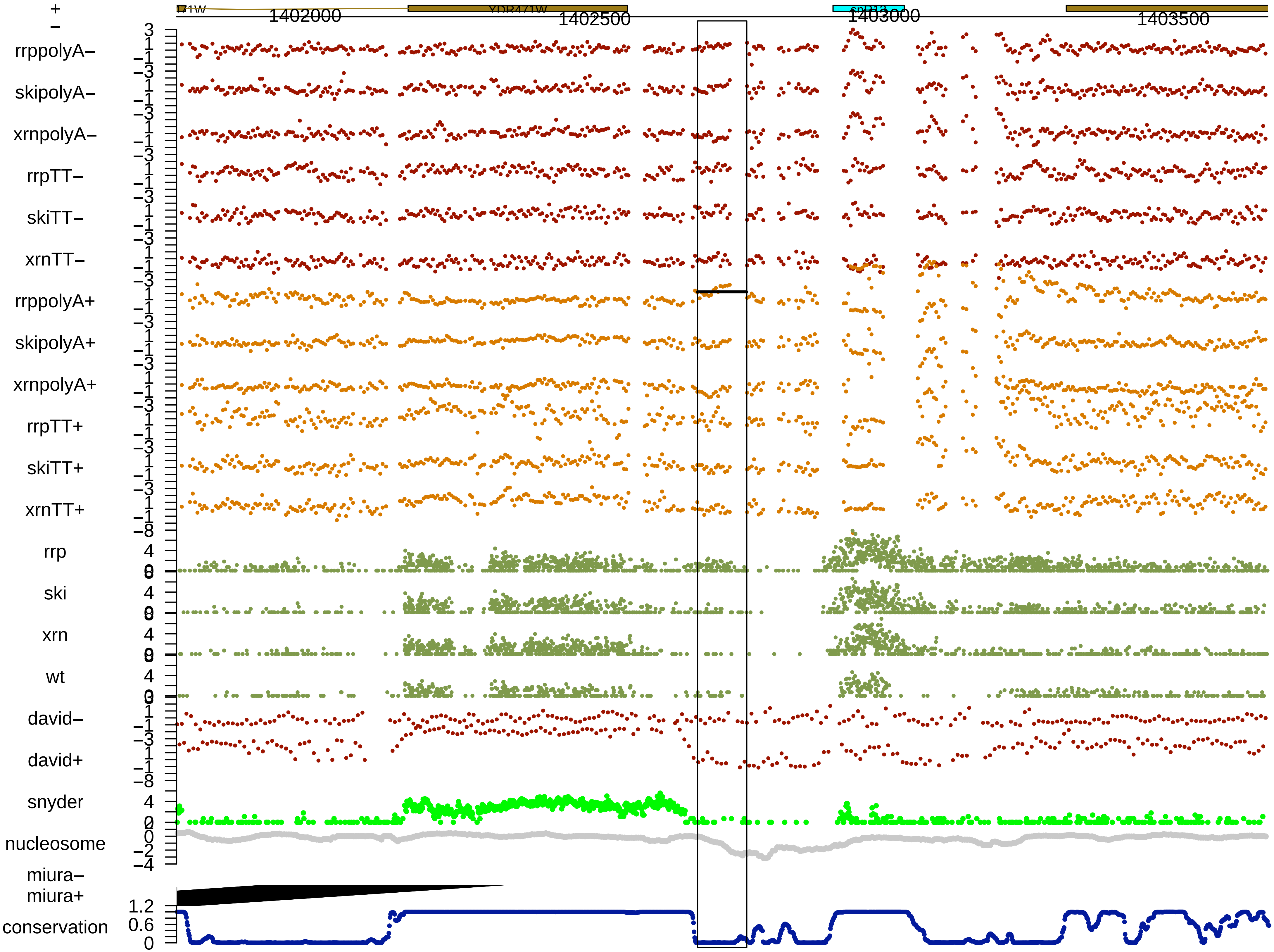
1402000
1402500
1403000
1403500
+
−
rrppolyA−
skipolyA−
xrnpolyA−
rrpTT−
skiTT−
xrnTT−
rrppolyA+
skipolyA+
xrnpolyA+
rrpTT+
skiTT+
xrnTT+
rrp
ski
xrn
wt
david−
david+
snyder
nucleosome
miura−
miura+
conservation

mcl.52, chr: 4

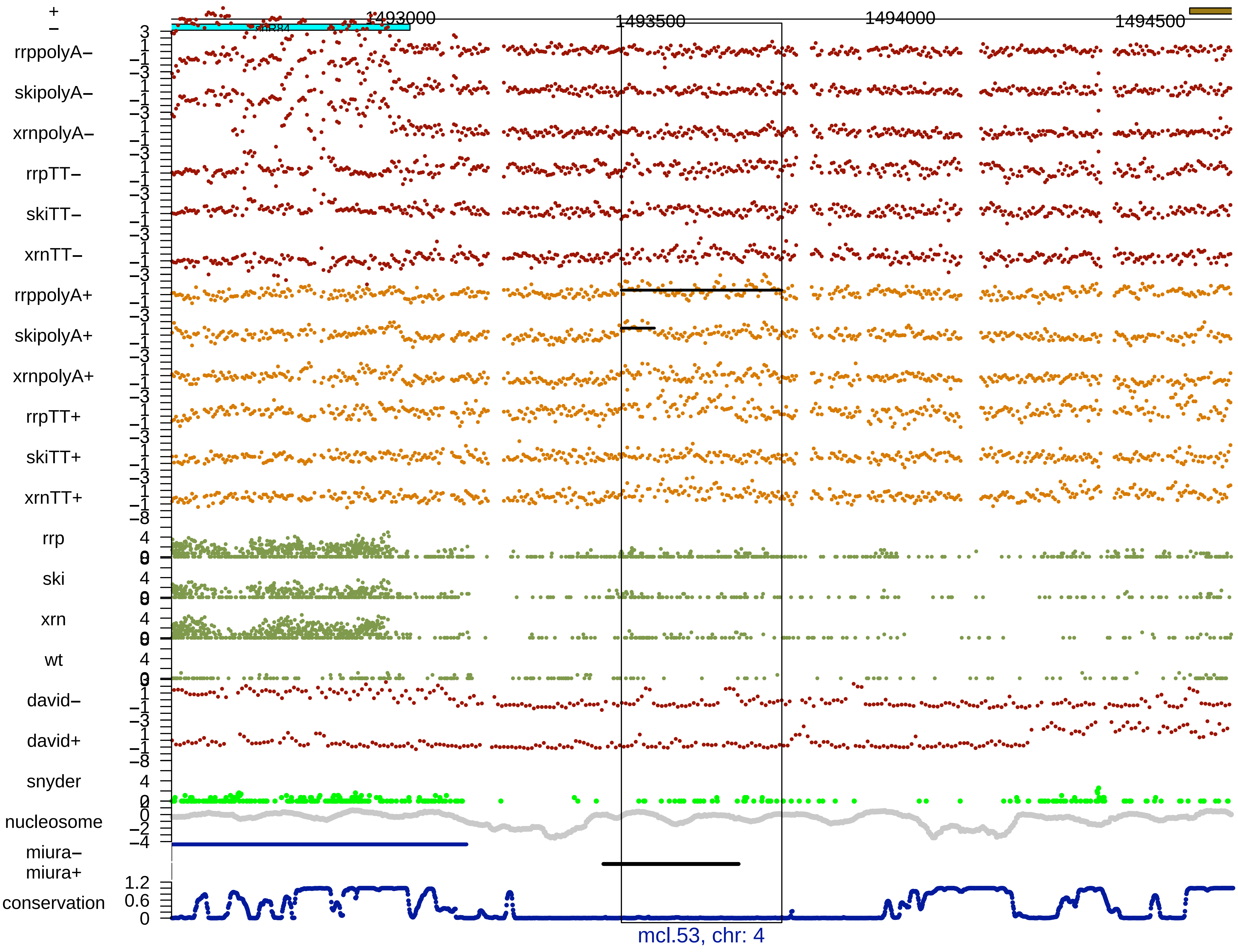
1493000
1493500
1494000
1494500
+
−
rrppolyA−
skipolyA−
xrnpolyA−
rrpTT−
skiTT−
xrnTT−
rrppolyA+
skipolyA+
xrnpolyA+
rrpTT+
skiTT+
xrnTT+
rrp
ski
xrn
wt
david−
david+
snyder
nucleosome
miura−
miura+
conservation
mcl.53, chr: 4

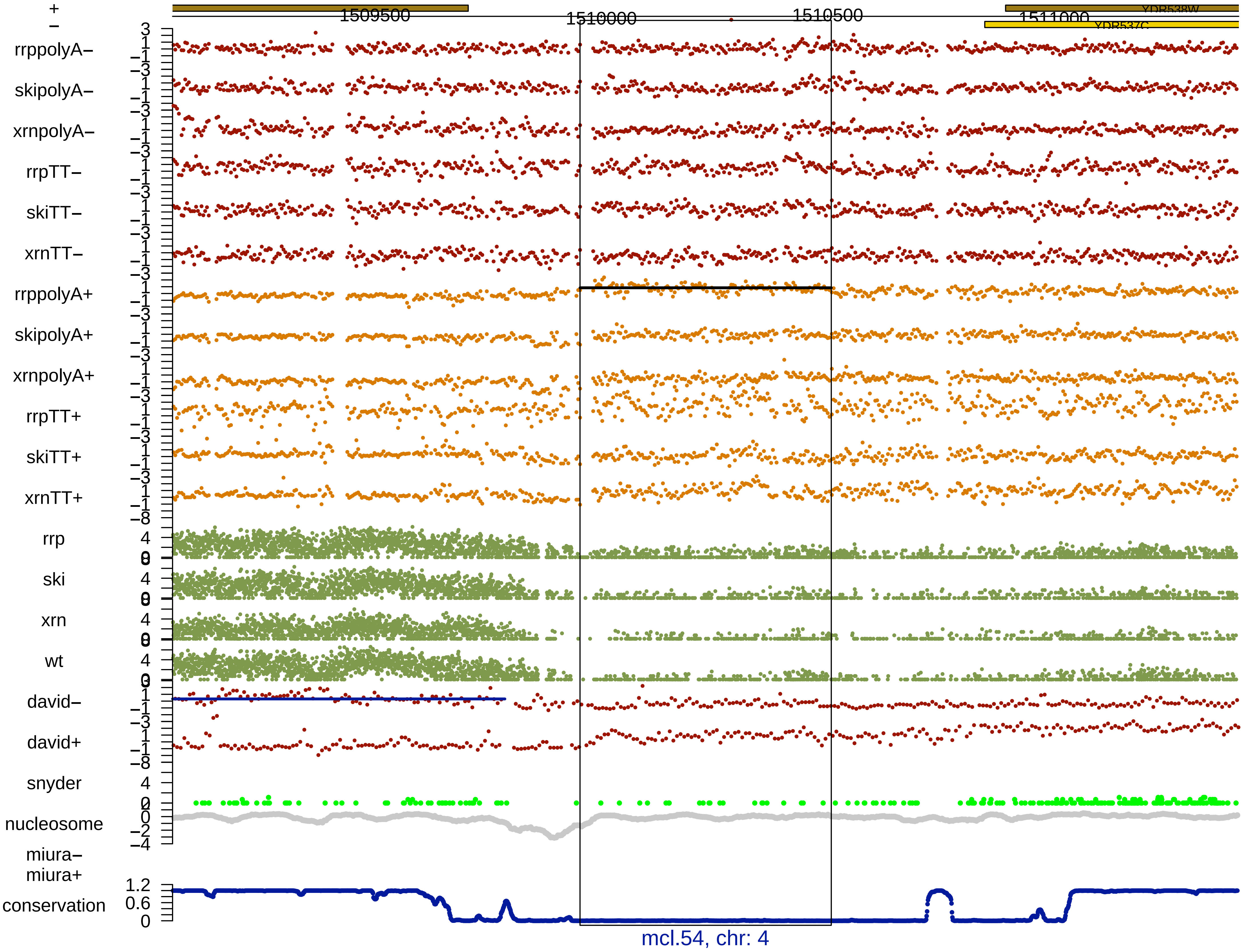

+
−
1509500
1510000
1510500
1511000
YDR538W
YDR537C
rrppolyA−
skipolyA−
xrnpolyA−
rrpTT−
skiTT−
xrnTT−
rrppolyA+
skipolyA+
xrnpolyA+
rrpTT+
skiTT+
xrnTT+
rrp
ski
xrn
wt
david−
david+
snyder
nucleosome
miura−
miura+
conservation
mcl.54, chr: 4

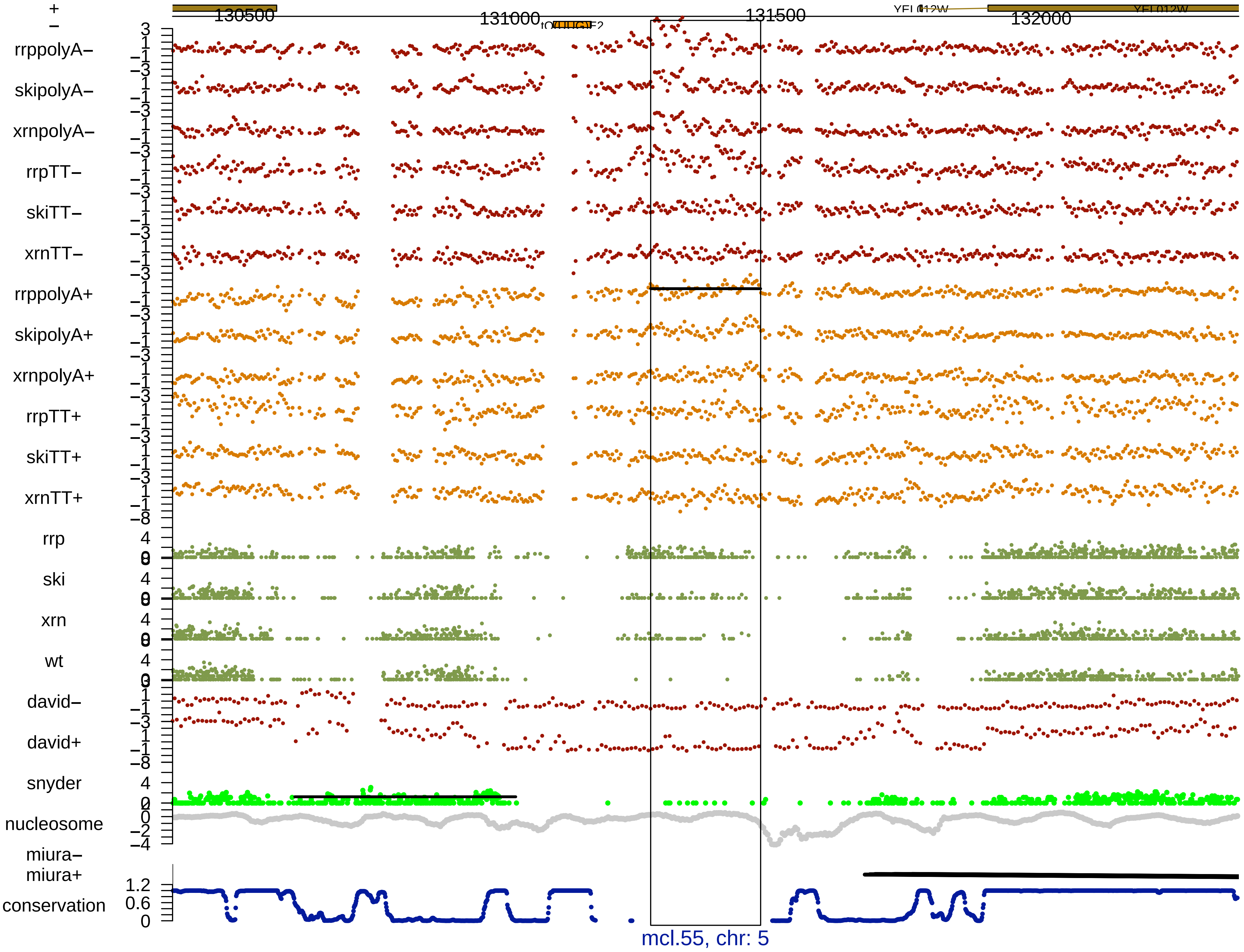

130500
131000
131500
132000
YEL012W
rrppolyA−
skipolyA−
xrnpolyA−
rrpTT−
skiTT−
xrnTT−
rrppolyA+
skipolyA+
xrnpolyA+
rrpTT+
skiTT+
xrnTT+
rrp
ski
xrn
wt
david−
david+
snyder
nucleosome
miura−
miura+
conservation
mcl.55, chr: 5

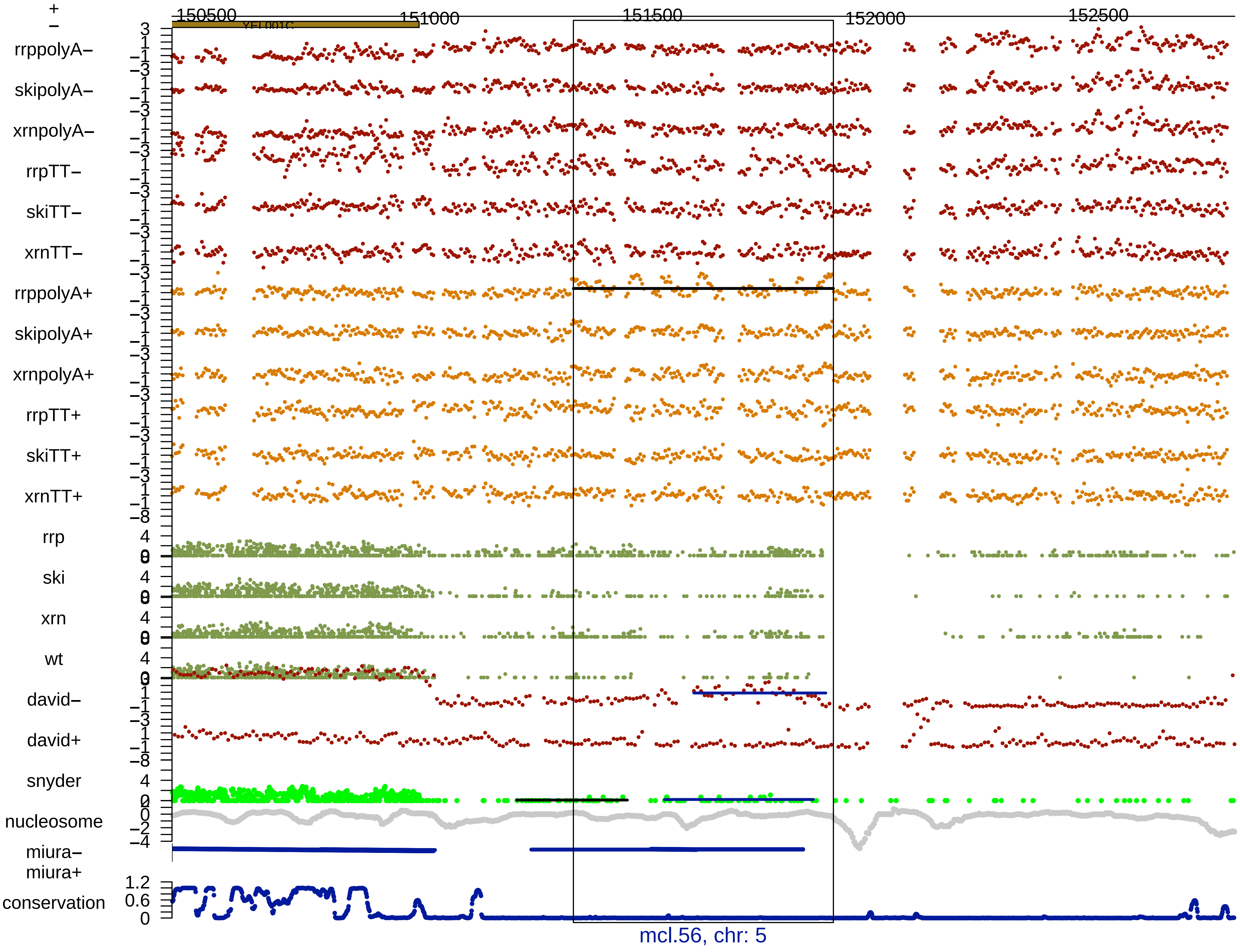

150500
151000
151500
152000
152500
YEL001C
+
−
rrppolyA−
skipolyA−
xrnpolyA−
rrpTT−
skiTT−
xrnTT−
rrppolyA+
skipolyA+
xrnpolyA+
rrpTT+
skiTT+
xrnTT+
rrp
ski
xrn
wt
david−
david+
snyder
nucleosome
miura−
miura+
conservation
mcl.56, chr: 5

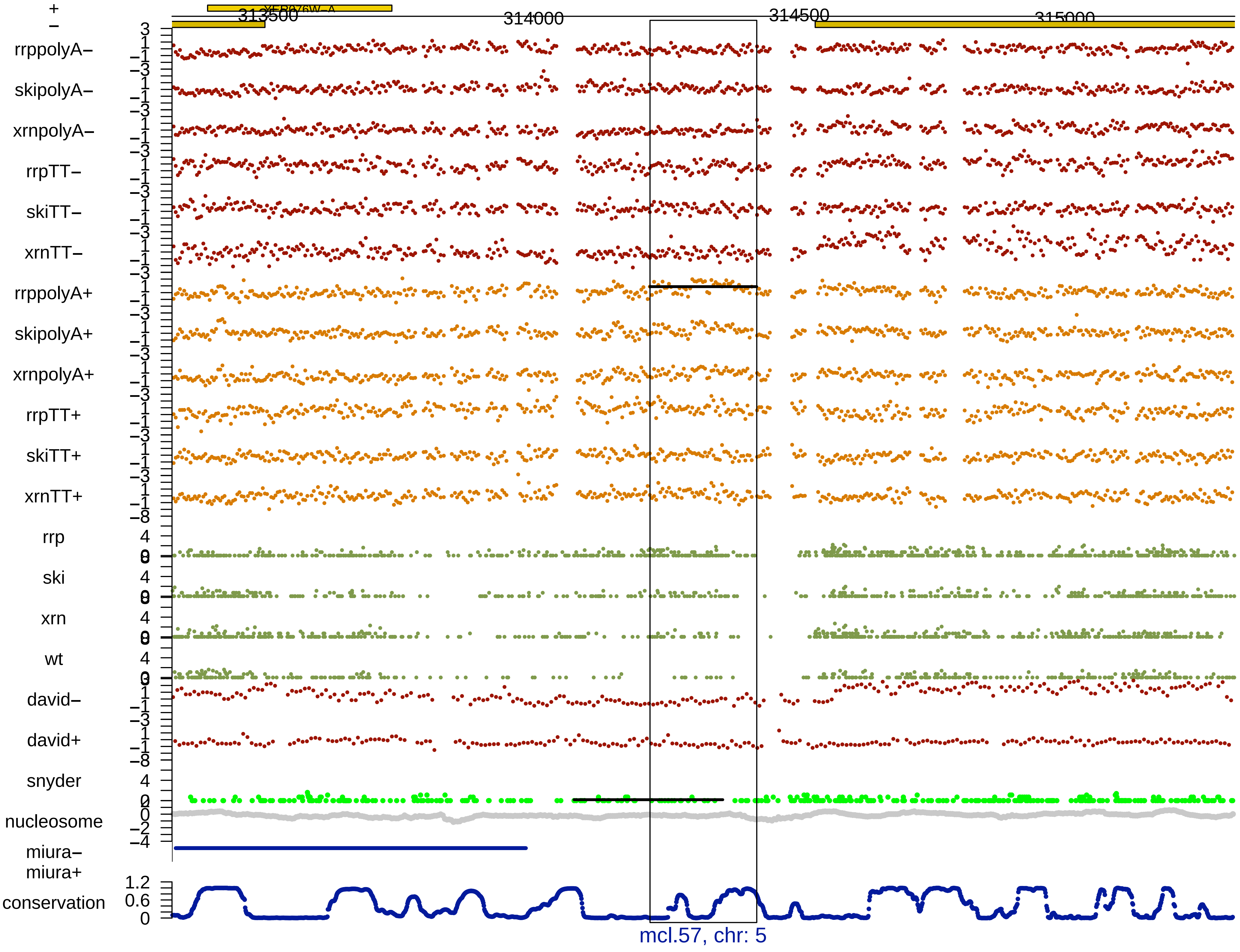

314000
314500
rrppolyA−
skipolyA−
xrnpolyA−
rrpTT−
skiTT−
xrnTT−
rrppolyA+
skipolyA+
xrnpolyA+
rrpTT+
skiTT+
xrnTT+
rrp
ski
xrn
wt
david−
david+
snyder
nucleosome
miura−
miura+
conservation
mcl.57, chr: 5

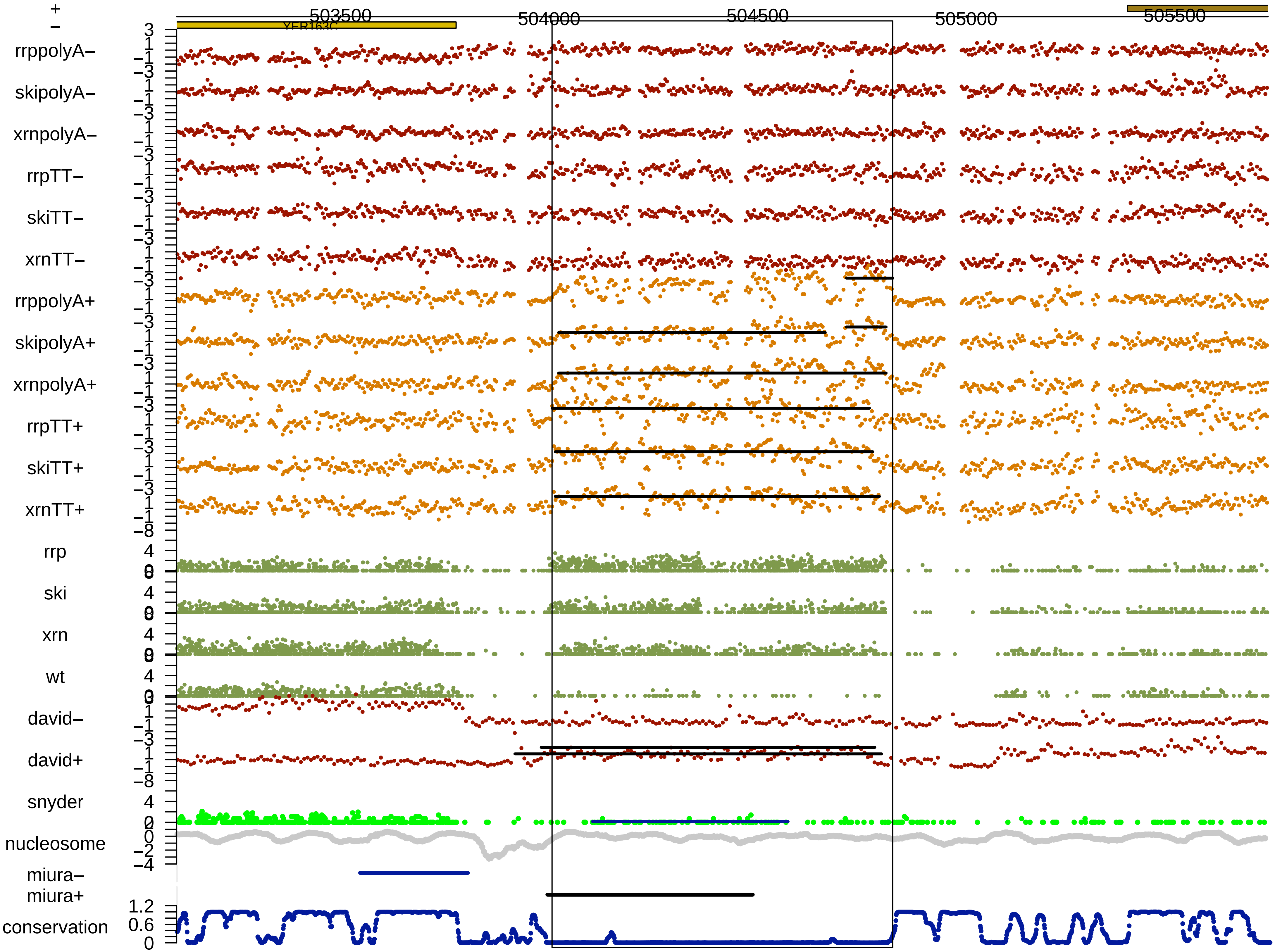
503500
504000
504500
505000
505500
YER163C
rrppolyA−
skipolyA−
xrnpolyA−
rrpTT−
skiTT−
xrnTT−
rrppolyA+
skipolyA+
xrnpolyA+
rrpTT+
skiTT+
xrnTT+
rrp
ski
xrn
wt
david−
david+
snyder
nucleosome
miura−
miura+
conservation

mcl.58, chr: 5

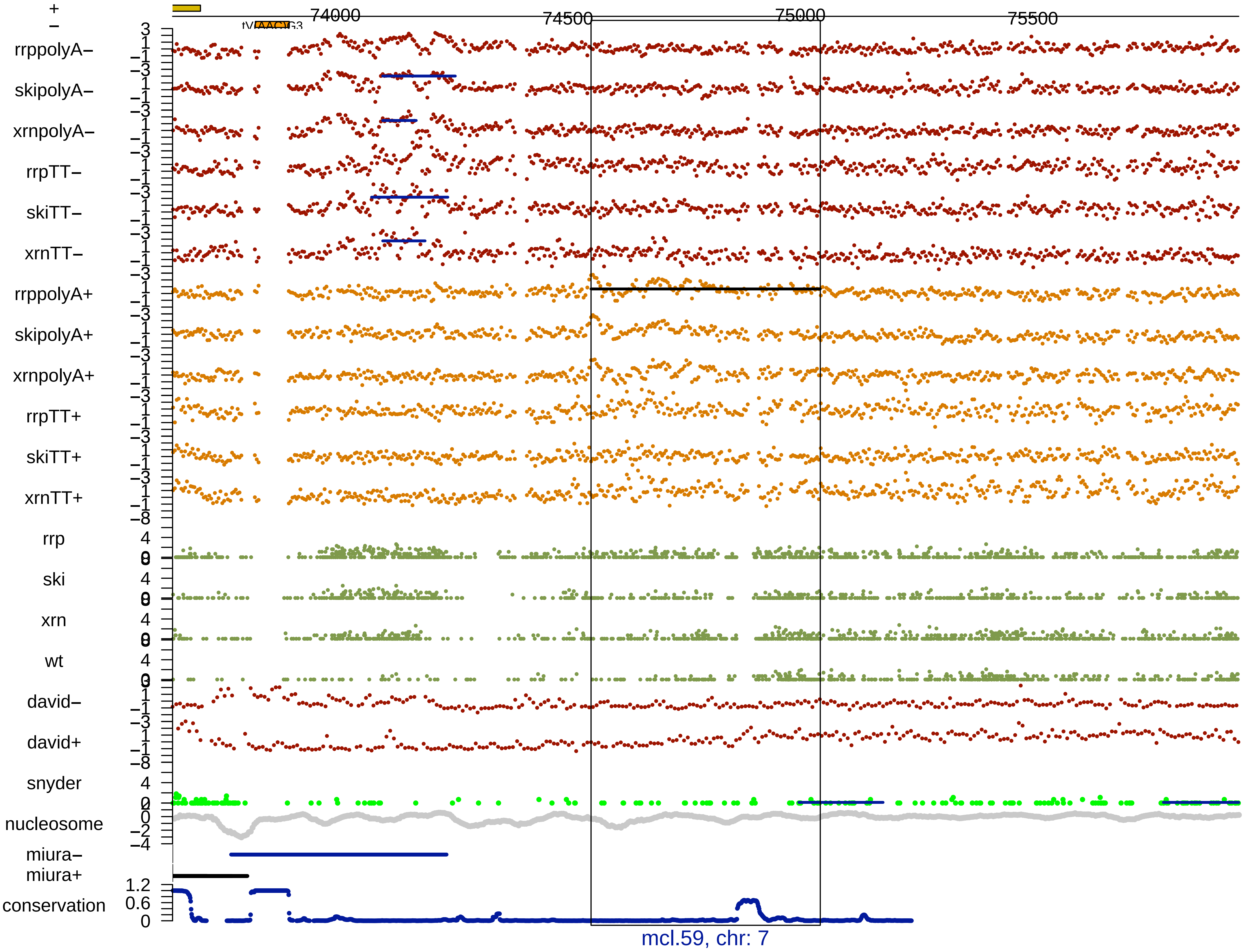

+
−
74000
74500
75000
75500
rrppolyA−
skipolyA−
xrnpolyA−
rrpTT−
skiTT−
xrnTT−
rrppolyA+
skipolyA+
xrnpolyA+
rrpTT+
skiTT+
xrnTT+
rrp
ski
xrn
wt
david−
david+
snyder
nucleosome
miura−
miura+
conservation
mcl.59, chr: 7

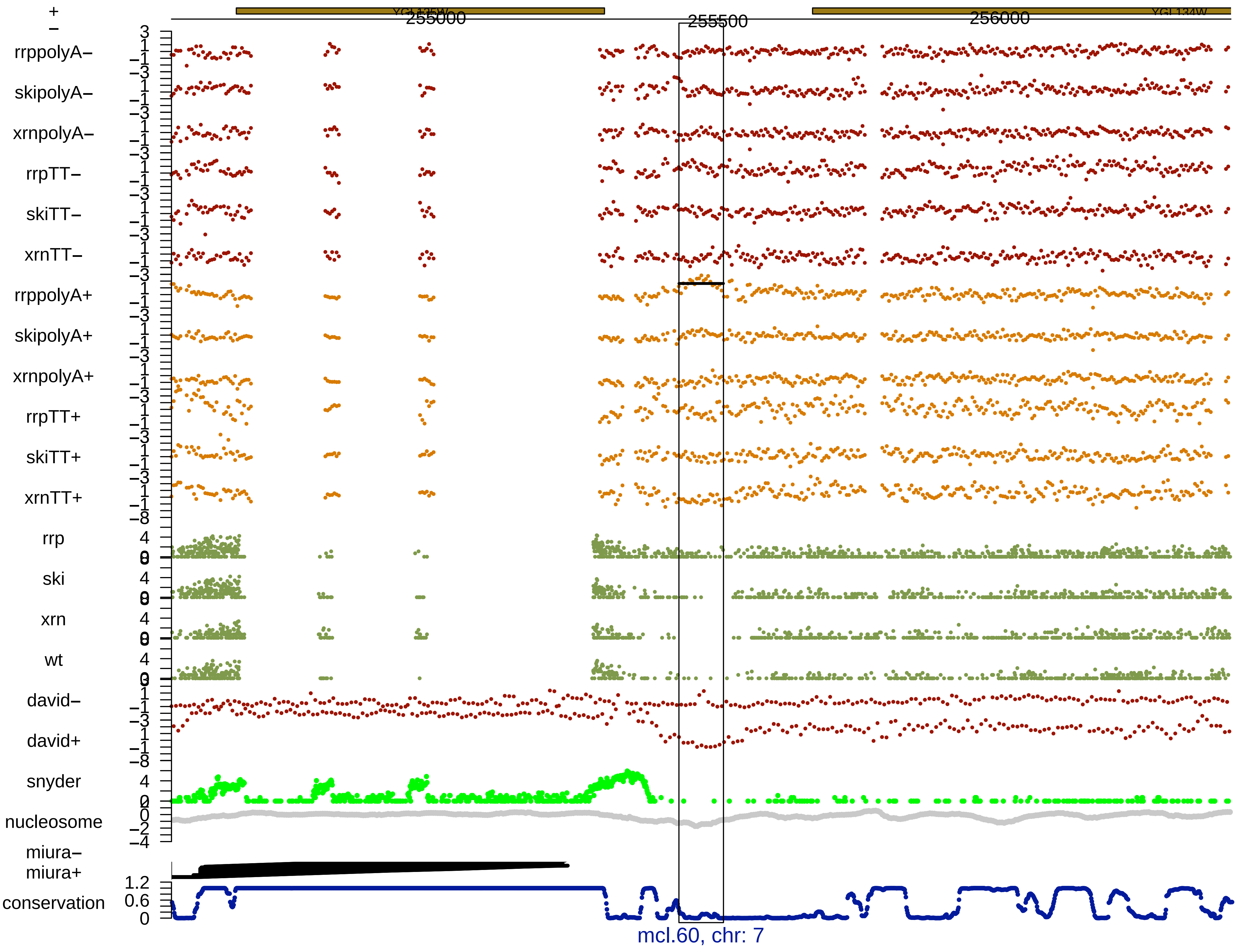

YGL135W
YGL134W
255000
255500
256000
rrppolyA−
skipolyA−
xrnpolyA−
rrpTT−
skiTT−
xrnTT−
rrppolyA+
skipolyA+
xrnpolyA+
rrpTT+
skiTT+
xrnTT+
rrp
ski
xrn
wt
david−
david+
snyder
nucleosome
miura−
miura+
conservation
mcl.60, chr: 7

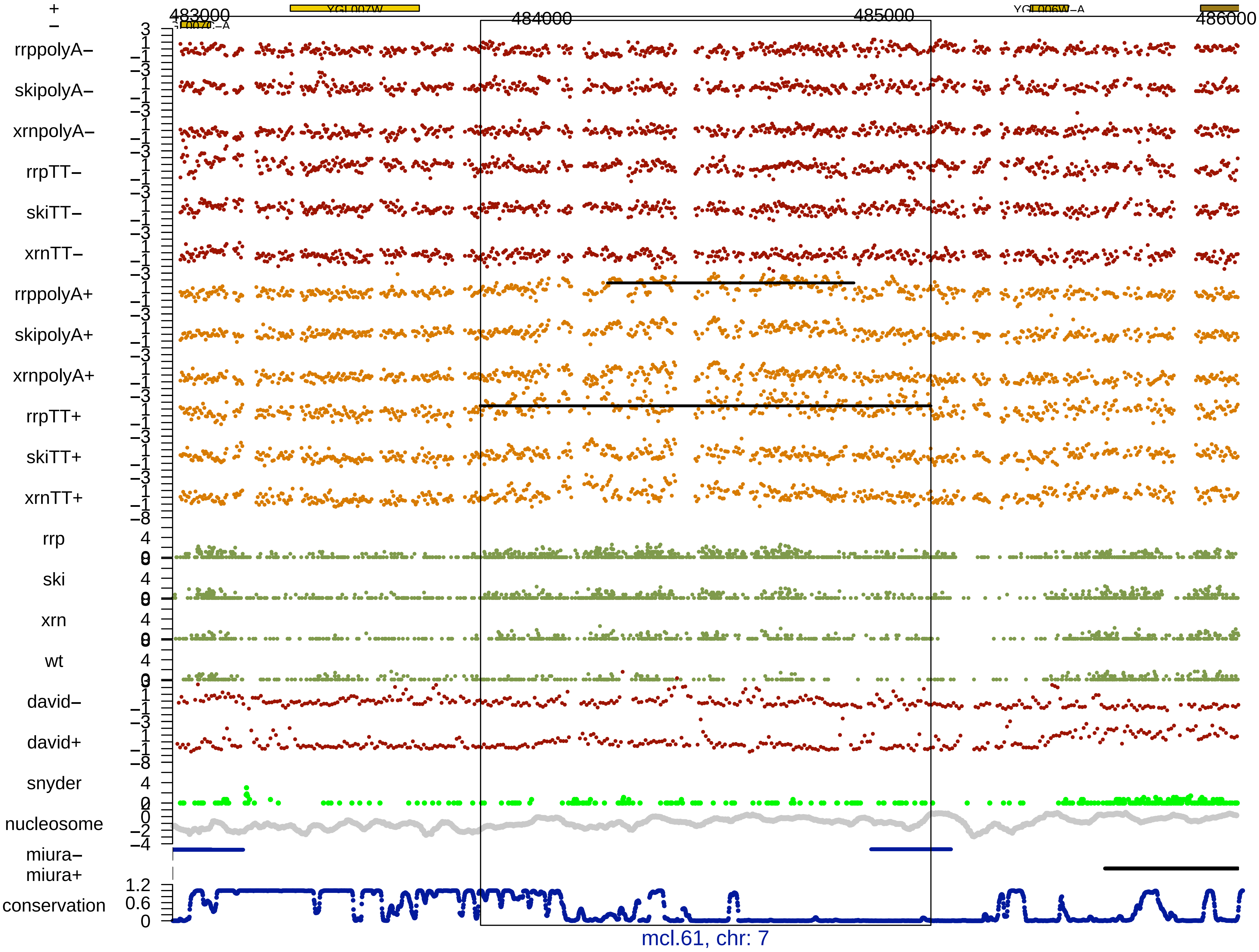

483000
484000
485000
486000
rrppolyA−
skipolyA−
xrnpolyA−
rrpTT−
skiTT−
xrnTT−
rrppolyA+
skipolyA+
xrnpolyA+
rrpTT+
skiTT+
xrnTT+
rrp
ski
xrn
wt
david−
david+
snyder
nucleosome
miura−
miura+
conservation
mcl.61, chr: 7

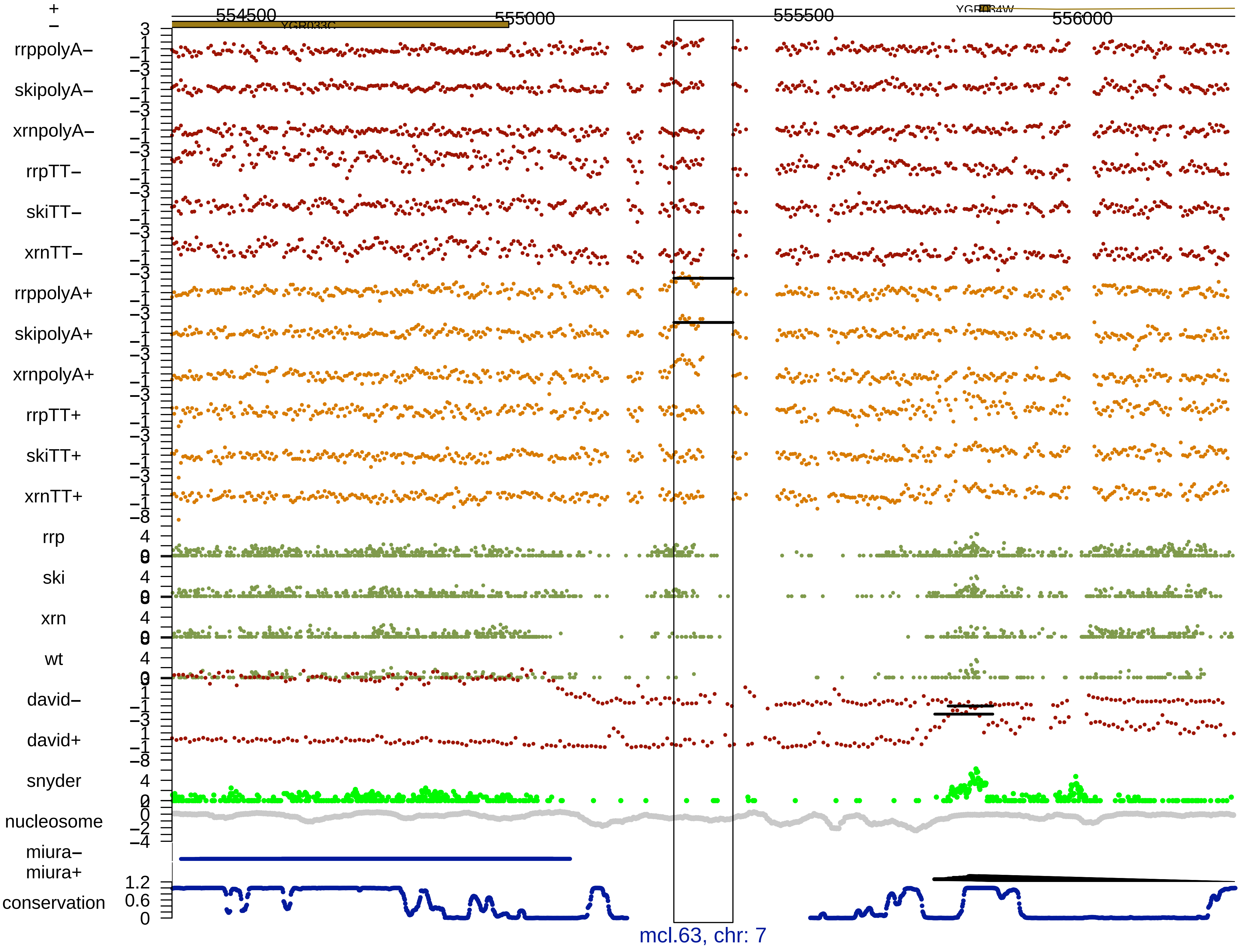

554500
555000
555500
556000
YGR033C
rrppolyA−
skipolyA−
xrnpolyA−
rrpTT−
skiTT−
xrnTT−
rrppolyA+
skipolyA+
xrnpolyA+
rrpTT+
skiTT+
xrnTT+
rrp
ski
xrn
wt
david−
david+
snyder
nucleosome
miura−
miura+
conservation
mcl.63, chr: 7

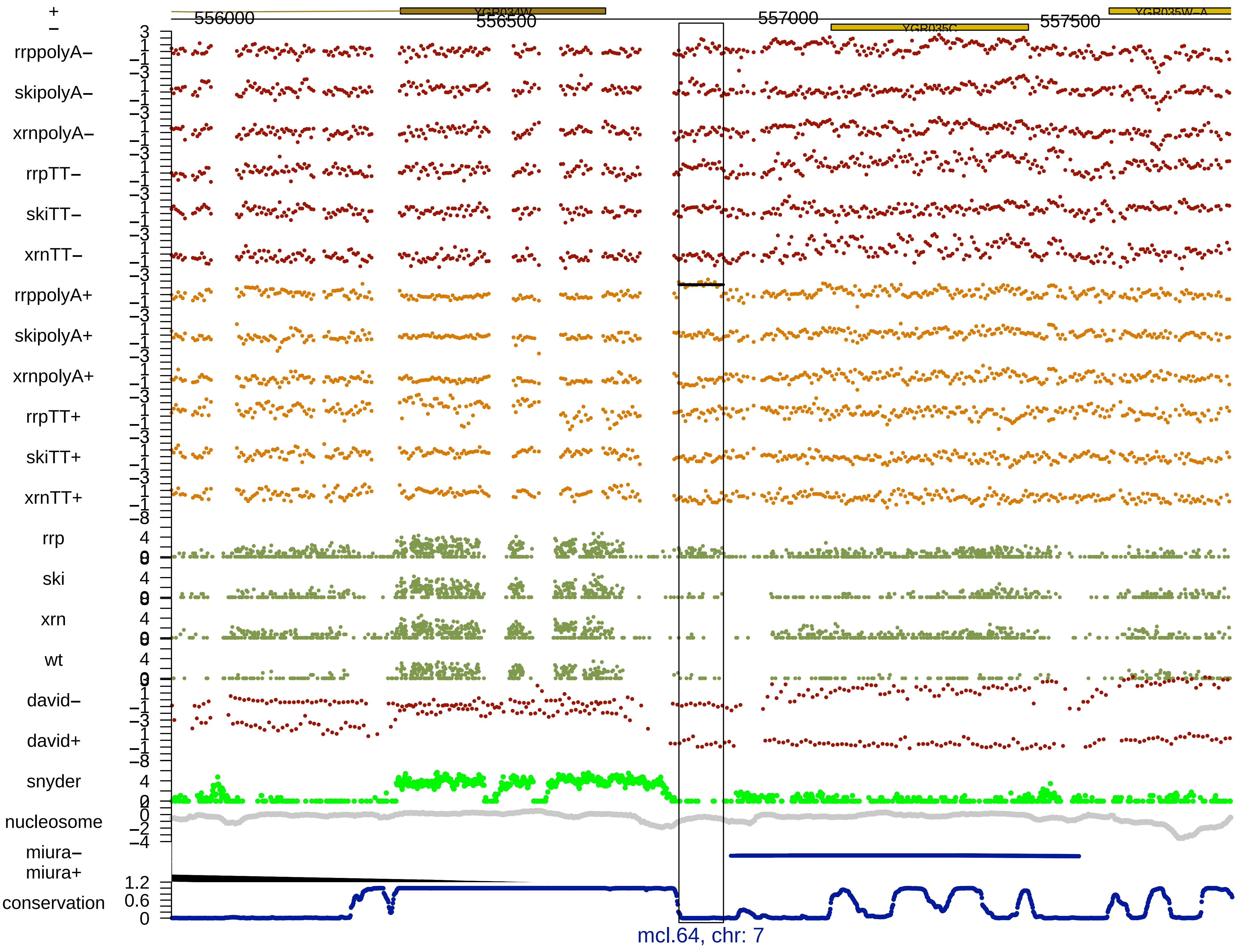

556000
556500
557000
557500
YGR034W
YGR035C
YGR035W-A
rrppolyA−
skipolyA−
xrnpolyA−
rrpTT−
skiTT−
xrnTT−
rrppolyA+
skipolyA+
xrnpolyA+
rrpTT+
skiTT+
xrnTT+
rrp
ski
xrn
wt
david−
david+
snyder
nucleosome
miura−
miura+
conservation
mcl.64, chr: 7

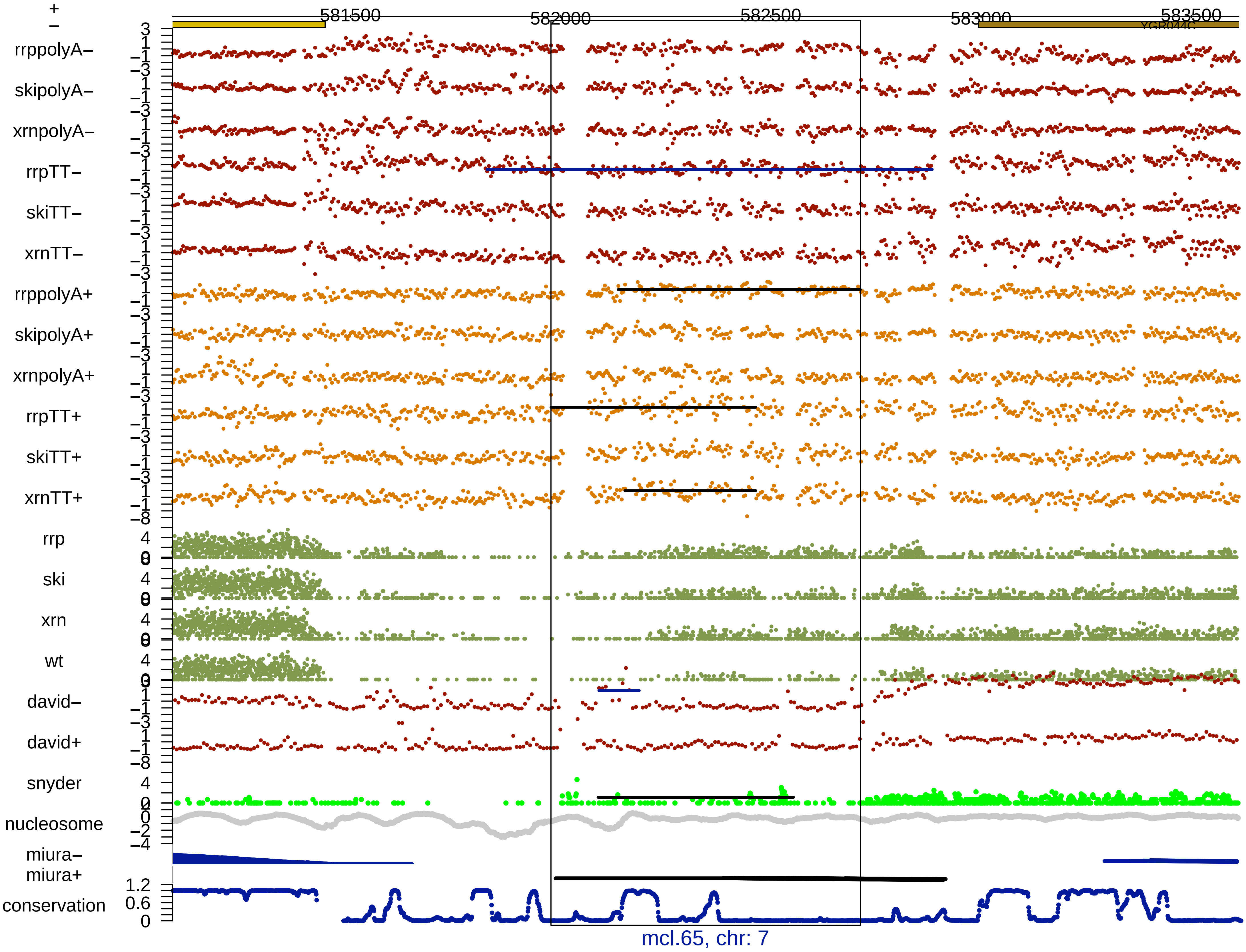

581500
582000
582500
583000
583500
rrppolyA−
skipolyA−
xrnpolyA−
rrpTT−
skiTT−
xrnTT−
rrppolyA+
skipolyA+
xrnpolyA+
rrpTT+
skiTT+
xrnTT+
rrp
ski
xrn
wt
david−
david+
snyder
nucleosome
miura−
miura+
conservation
mcl.65, chr: 7

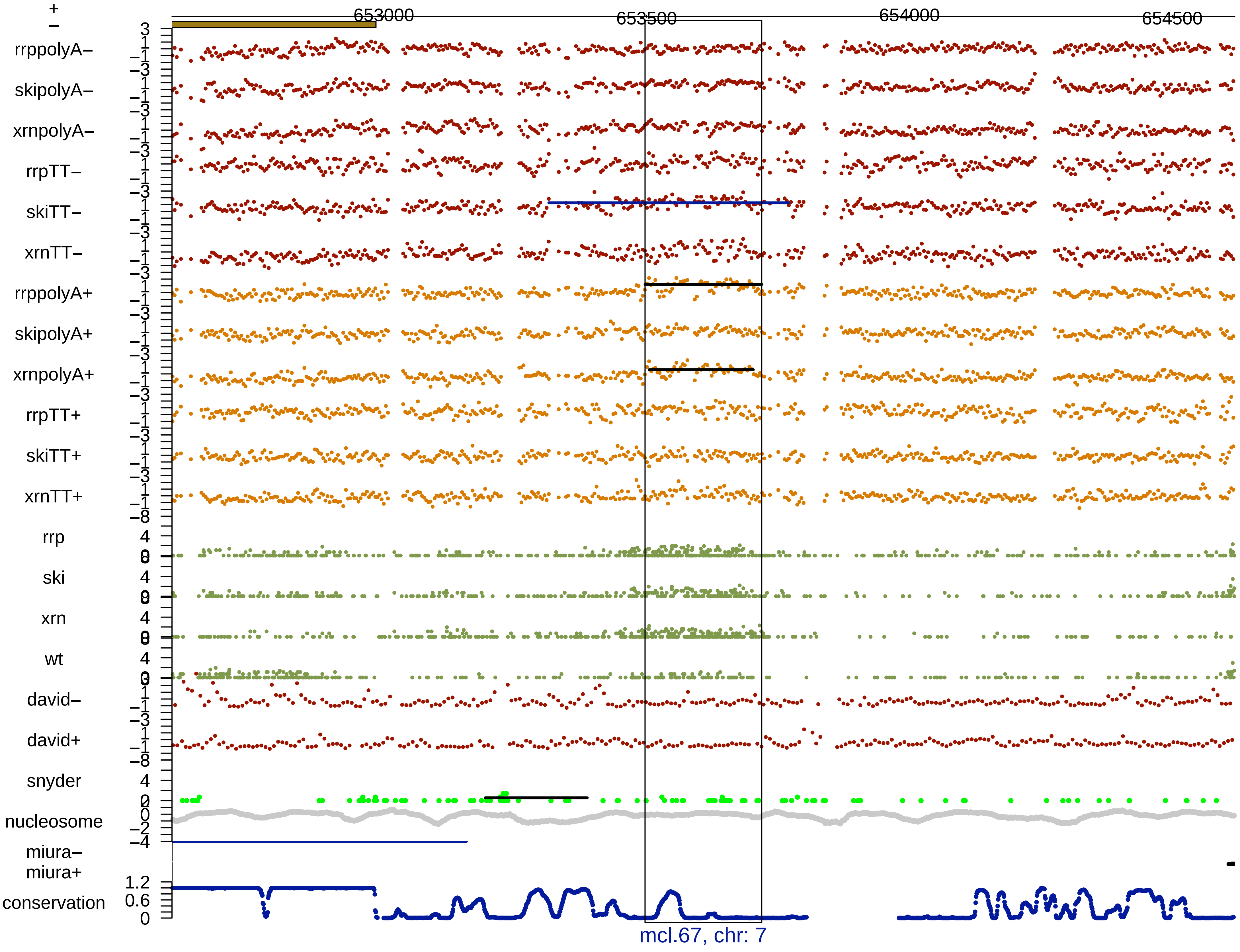

653000
653500
654000
654500
rrppolyA−
skipolyA−
xrnpolyA−
rrpTT−
skiTT−
xrnTT−
rrppolyA+
skipolyA+
xrnpolyA+
rrpTT+
skiTT+
xrnTT+
rrp
ski
xrn
wt
david−
david+
snyder
nucleosome
miura−
miura+
conservation
mcl.67, chr: 7

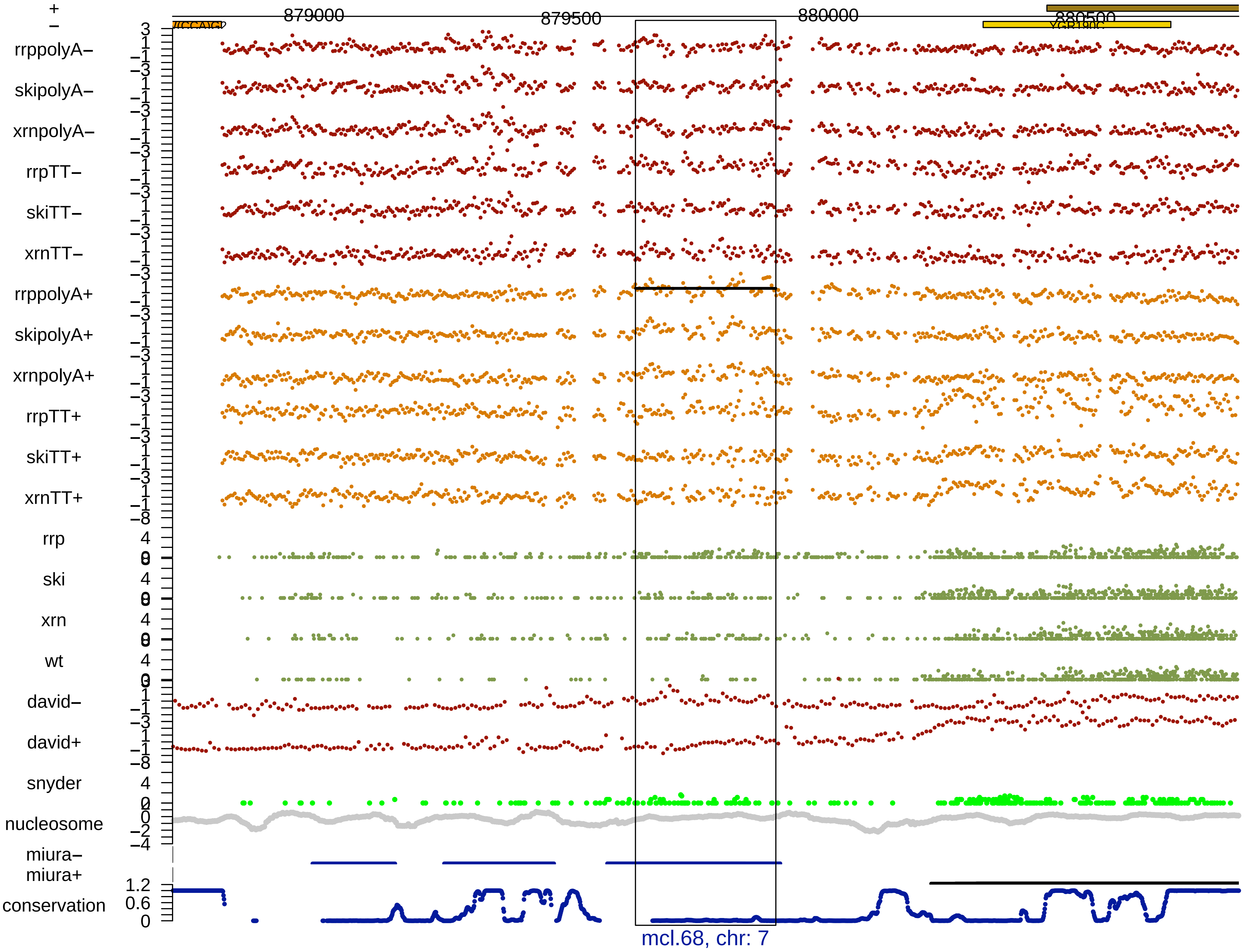
879000
879500
880000
880500
YGR190C
rrppolyA−
skipolyA−
xrnpolyA−
rrpTT−
skiTT−
xrnTT−
rrppolyA+
skipolyA+
xrnpolyA+
rrpTT+
skiTT+
xrnTT+
rrp
ski
xrn
wt
david−
david+
snyder
nucleosome
miura−
miura+
conservation
mcl.68, chr: 7

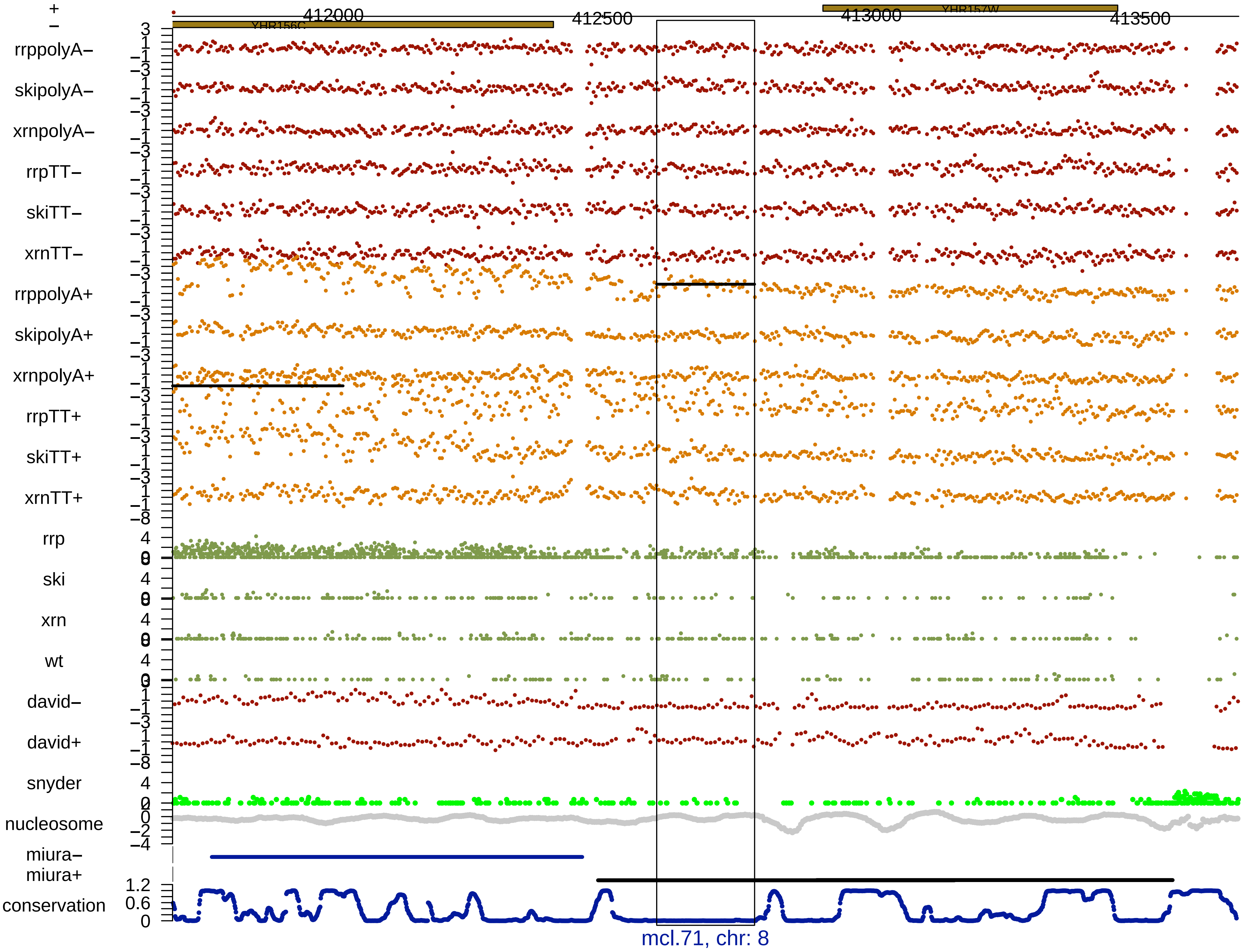

+
–
412000
412500
413000
413500
YHR156C
YHR157W
rrppolyA–
skipolyA–
xrnpolyA–
rrpTT–
skiTT–
xrnTT–
rrppolyA+
skipolyA+
xrnpolyA+
rrpTT+
skiTT+
xrnTT+
rrp
ski
xrn
wt
david–
david+
snyder
nucleosome
miura–
miura+
conservation
mcl.71, chr: 8

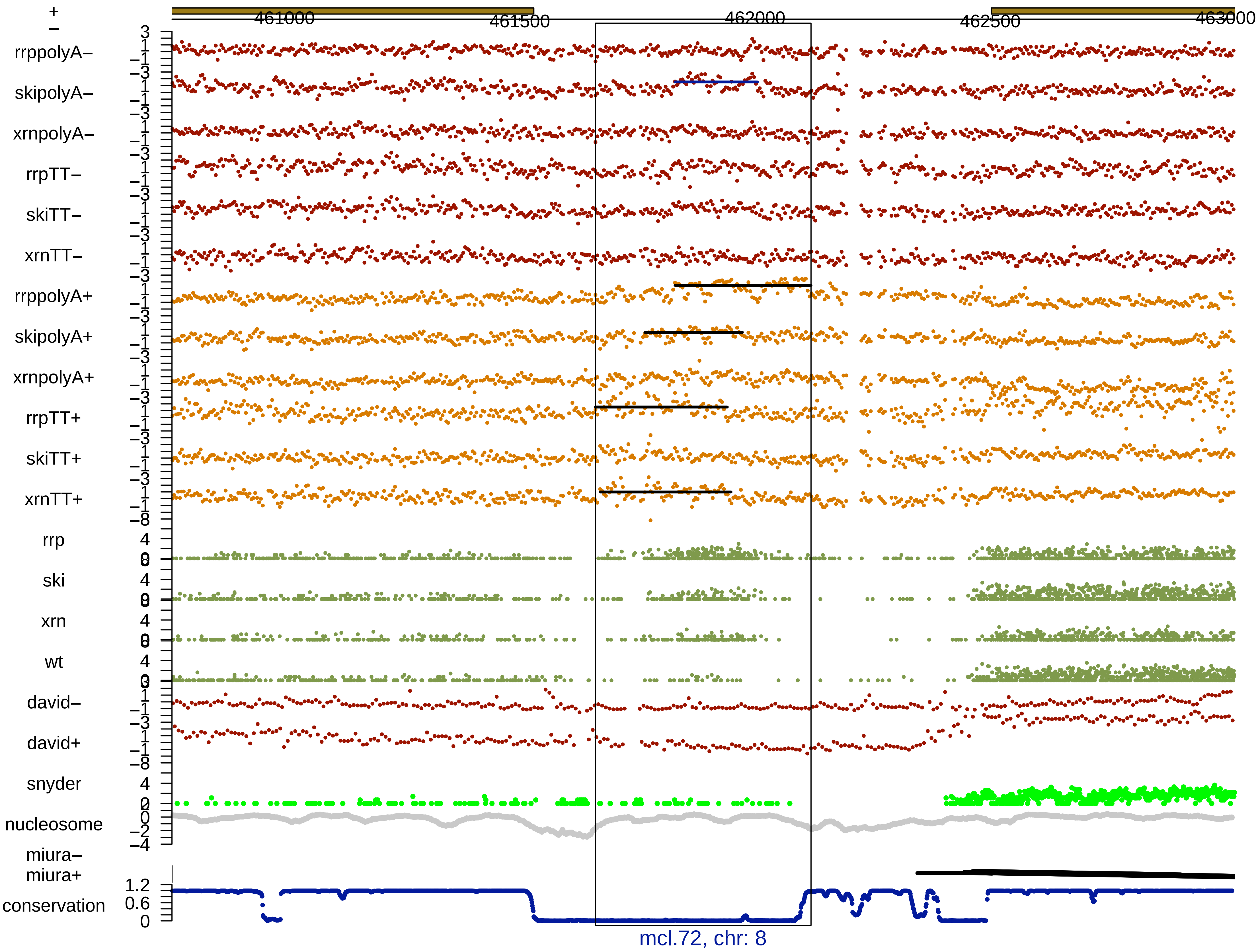

461000
461500
462000
462500
463000
rrppolyA−
skipolyA−
xrnpolyA−
rrpTT−
skiTT−
xrnTT−
rrppolyA+
skipolyA+
xrnpolyA+
rrpTT+
skiTT+
xrnTT+
rrp
ski
xrn
wt
david−
david+
snyder
nucleosome
miura−
miura+
conservation
mcl.72, chr: 8

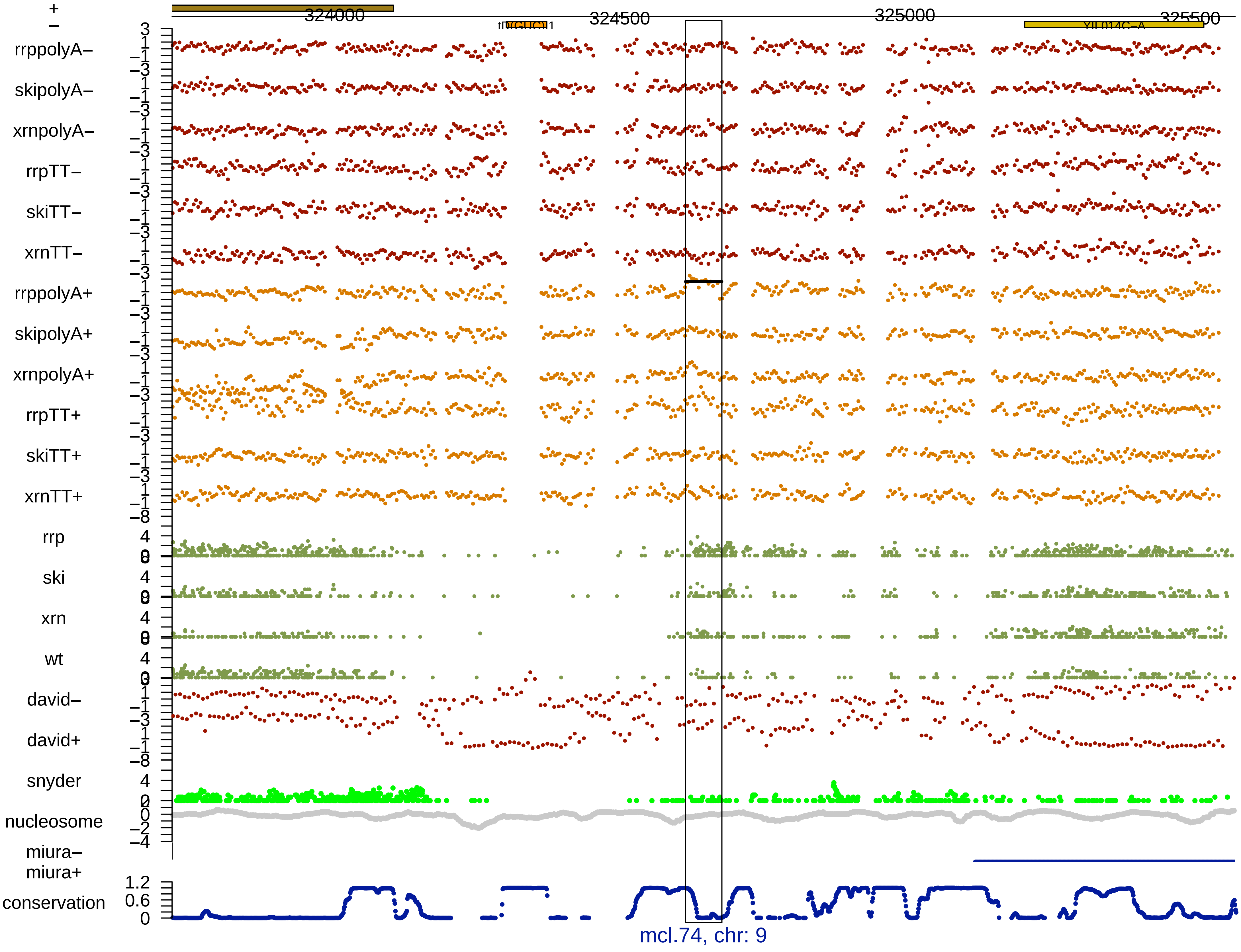

324000
324500
325000
325500
rrppolyA–
skipolyA–
xrnpolyA–
rrpTT–
skiTT–
xrnTT–
rrppolyA+
skipolyA+
xrnpolyA+
rrpTT+
skiTT+
xrnTT+
rrp
ski
xrn
wt
david–
david+
snyder
nucleosome
miura–
miura+
conservation
mcl.74, chr: 9

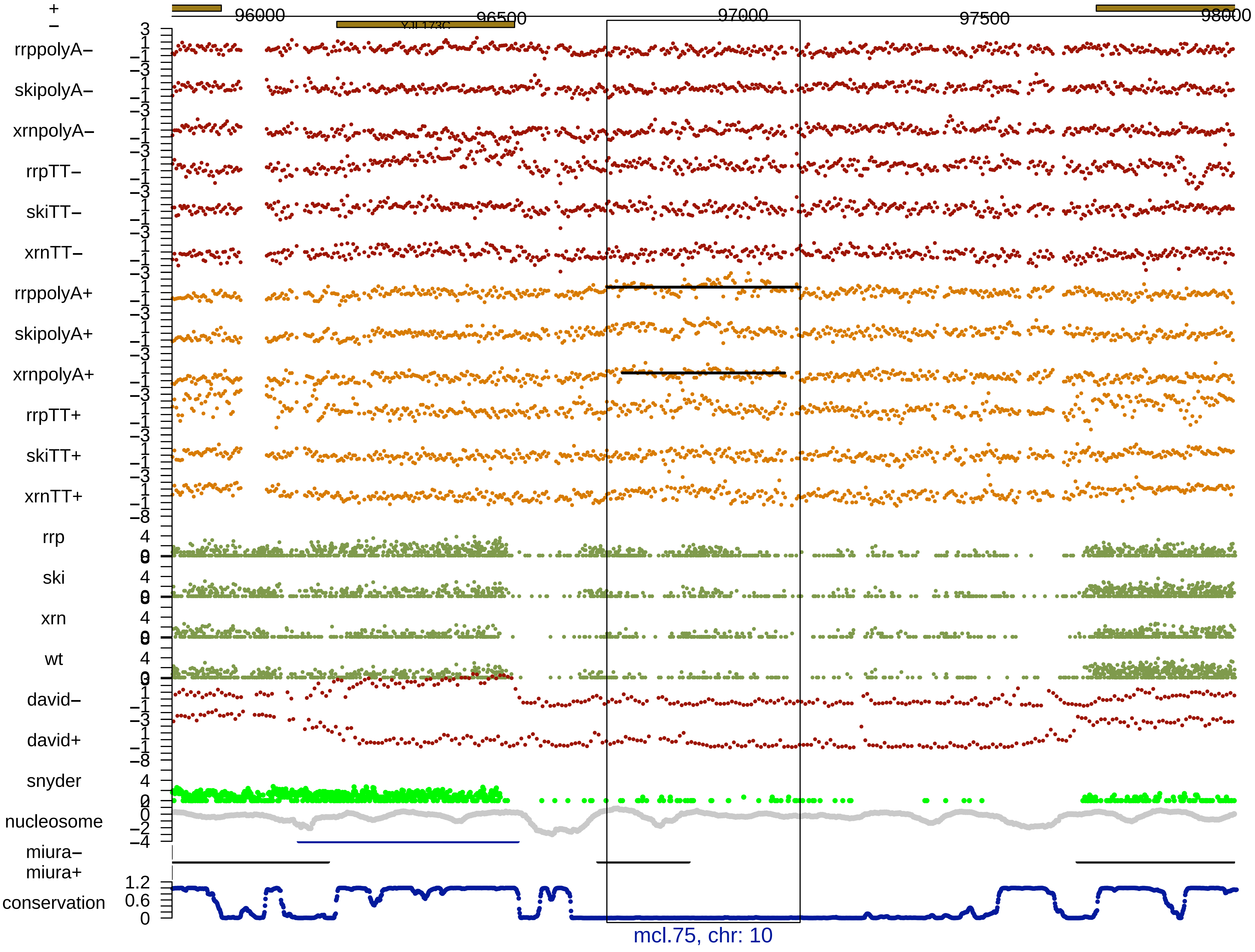
+
–
96000
96500
97000
97500
98000
rrppolyA–
skipolyA–
xrnpolyA–
rrpTT–
skiTT–
xrnTT–
rrppolyA+
skipolyA+
xrnpolyA+
rrpTT+
skiTT+
xrnTT+
rrp
ski
xrn
wt
david–
david+
snyder
nucleosome
miura–
miura+
conservation
mcl.75, chr: 10

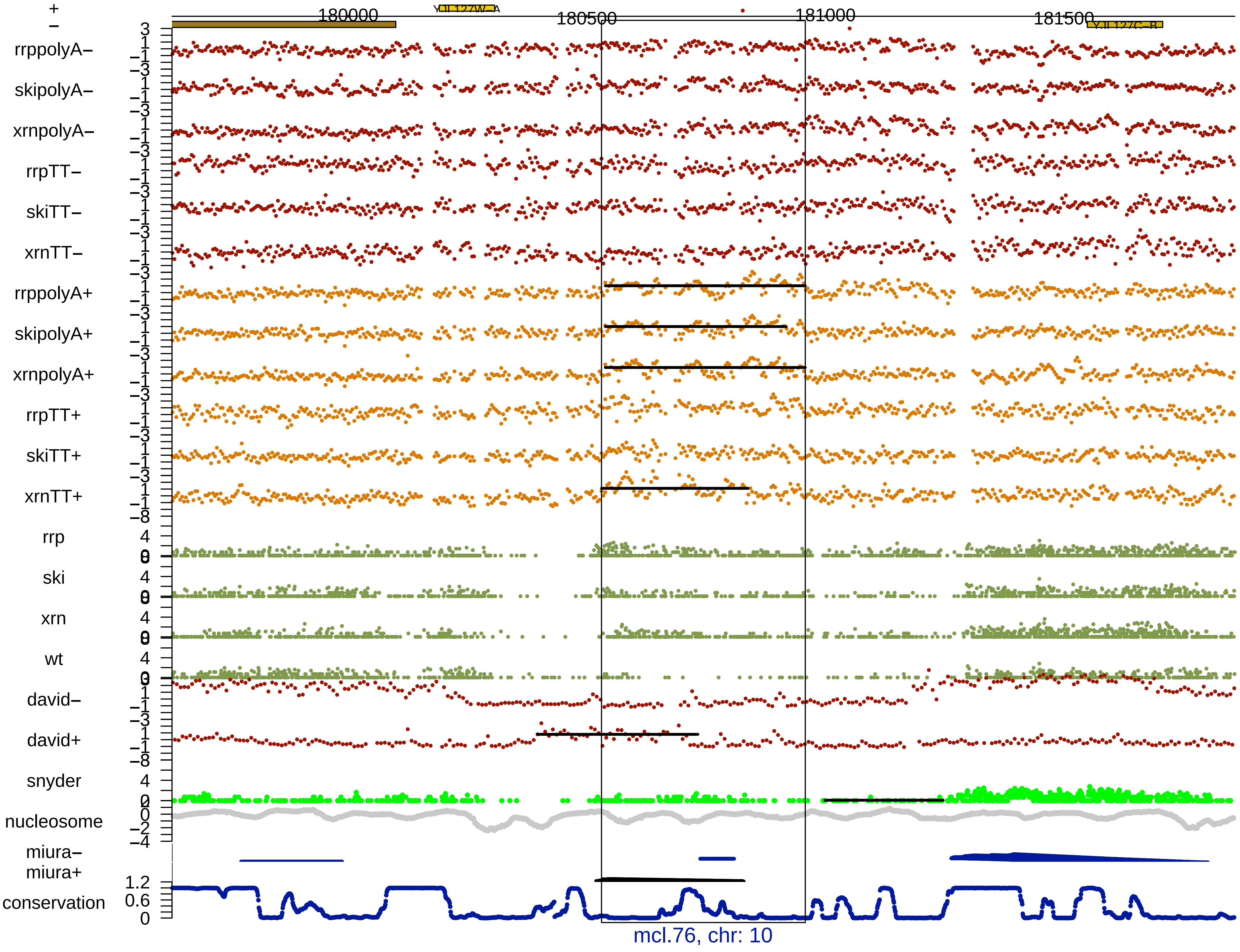

180000
180500
181000
181500
rrppolyA−
skipolyA−
xrnpolyA−
rrpTT−
skiTT−
xrnTT−
rrppolyA+
skipolyA+
xrnpolyA+
rrpTT+
skiTT+
xrnTT+
rrp
ski
xrn
wt
david−
david+
snyder
nucleosome
miura−
miura+
conservation
mcl.76, chr: 10

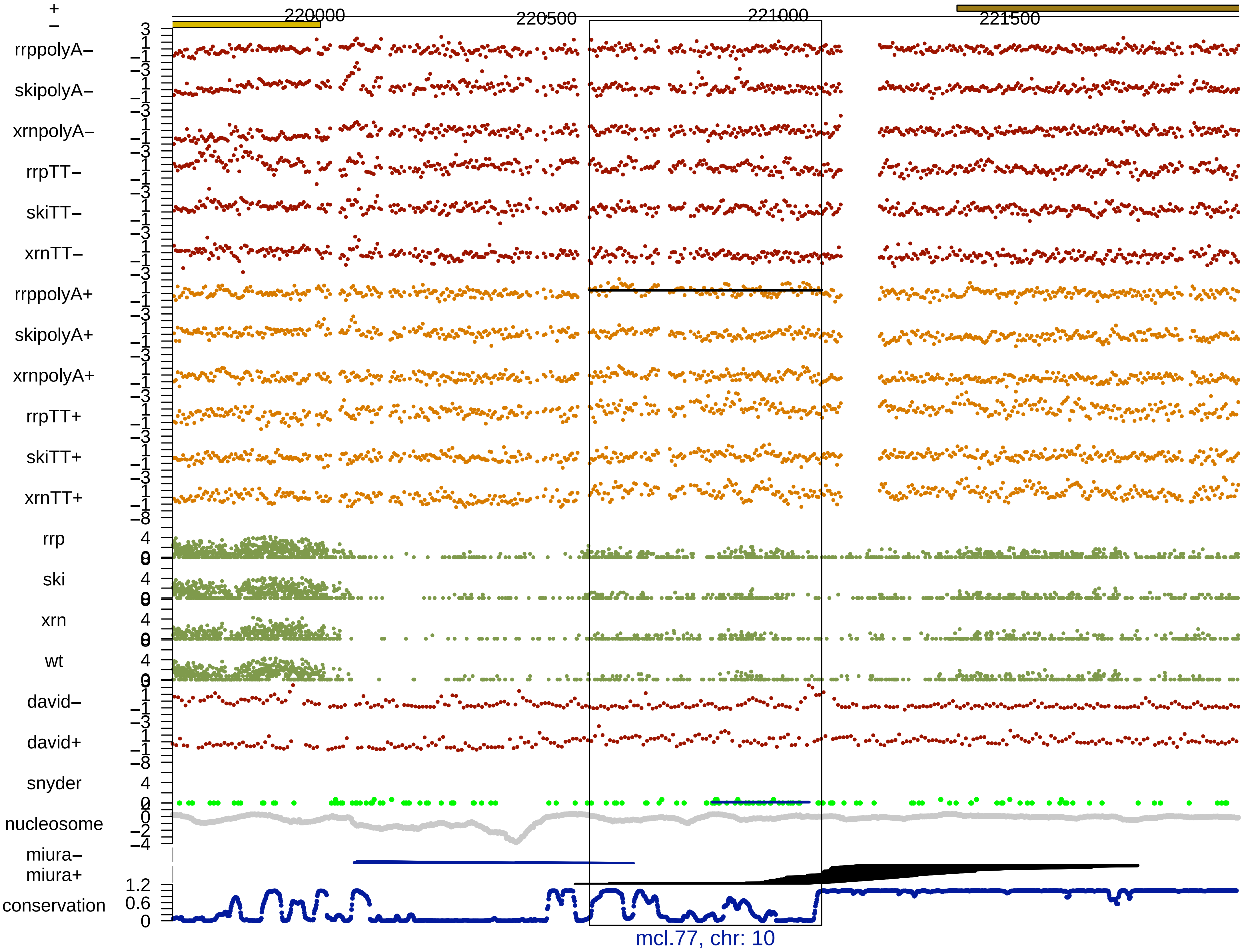

+
−
220000
220500
221000
221500
rrppolyA−
skipolyA−
xrnpolyA−
rrpTT−
skiTT−
xrnTT−
rrppolyA+
skipolyA+
xrnpolyA+
rrpTT+
skiTT+
xrnTT+
rrp
ski
xrn
wt
david−
david+
snyder
nucleosome
miura−
miura+
conservation
mcl.77, chr: 10

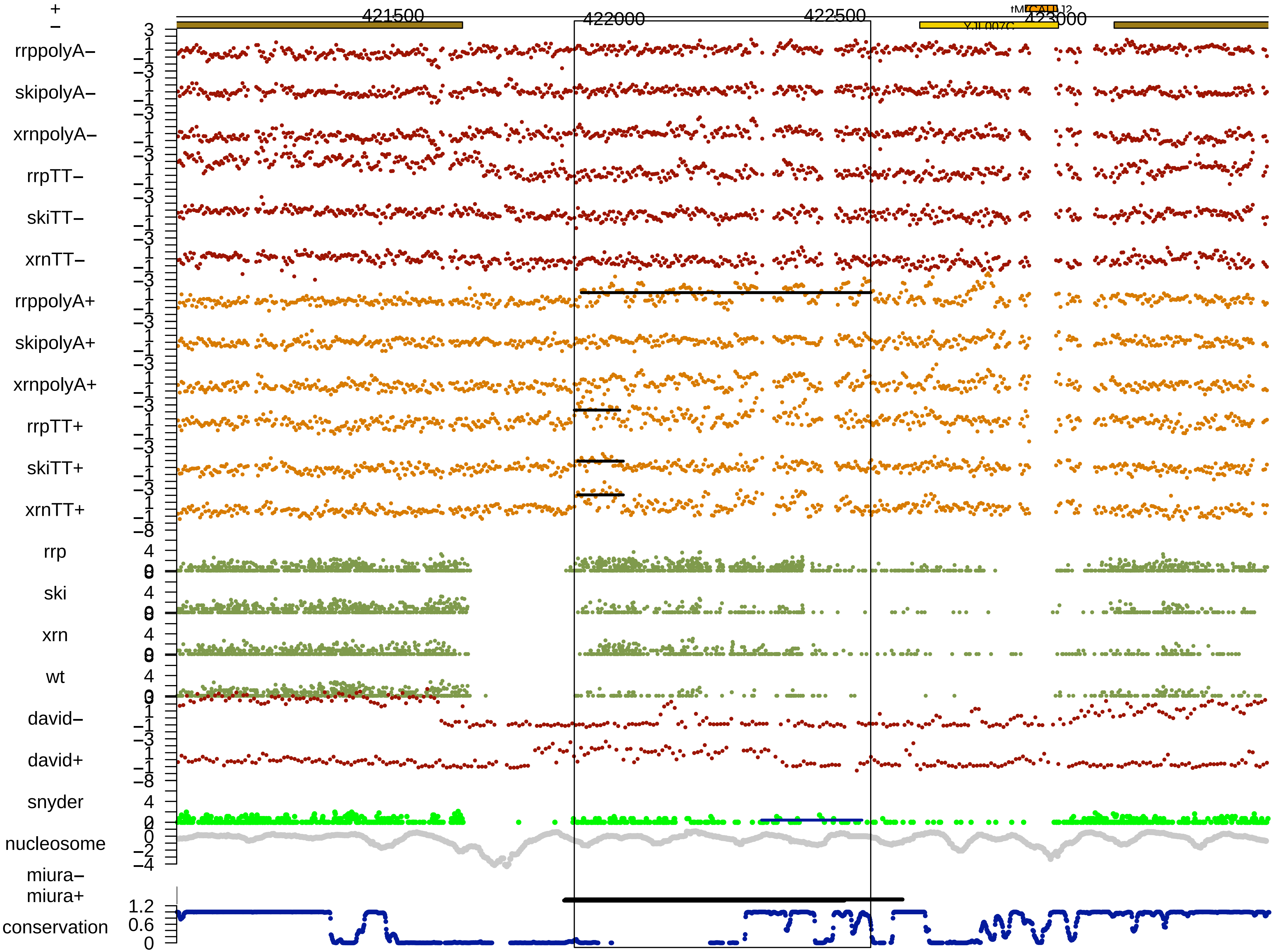

+
–
421500
422000
422500
423000
rrppolyA–
skipolyA–
xrnpolyA–
rrpTT–
skiTT–
xrnTT–
rrppolyA+
skipolyA+
xrnpolyA+
rrpTT+
skiTT+
xrnTT+
rrp
ski
xrn
wt
david–
david+
snyder
nucleosome
miura–
miura+
conservation

mcl.79, chr: 10

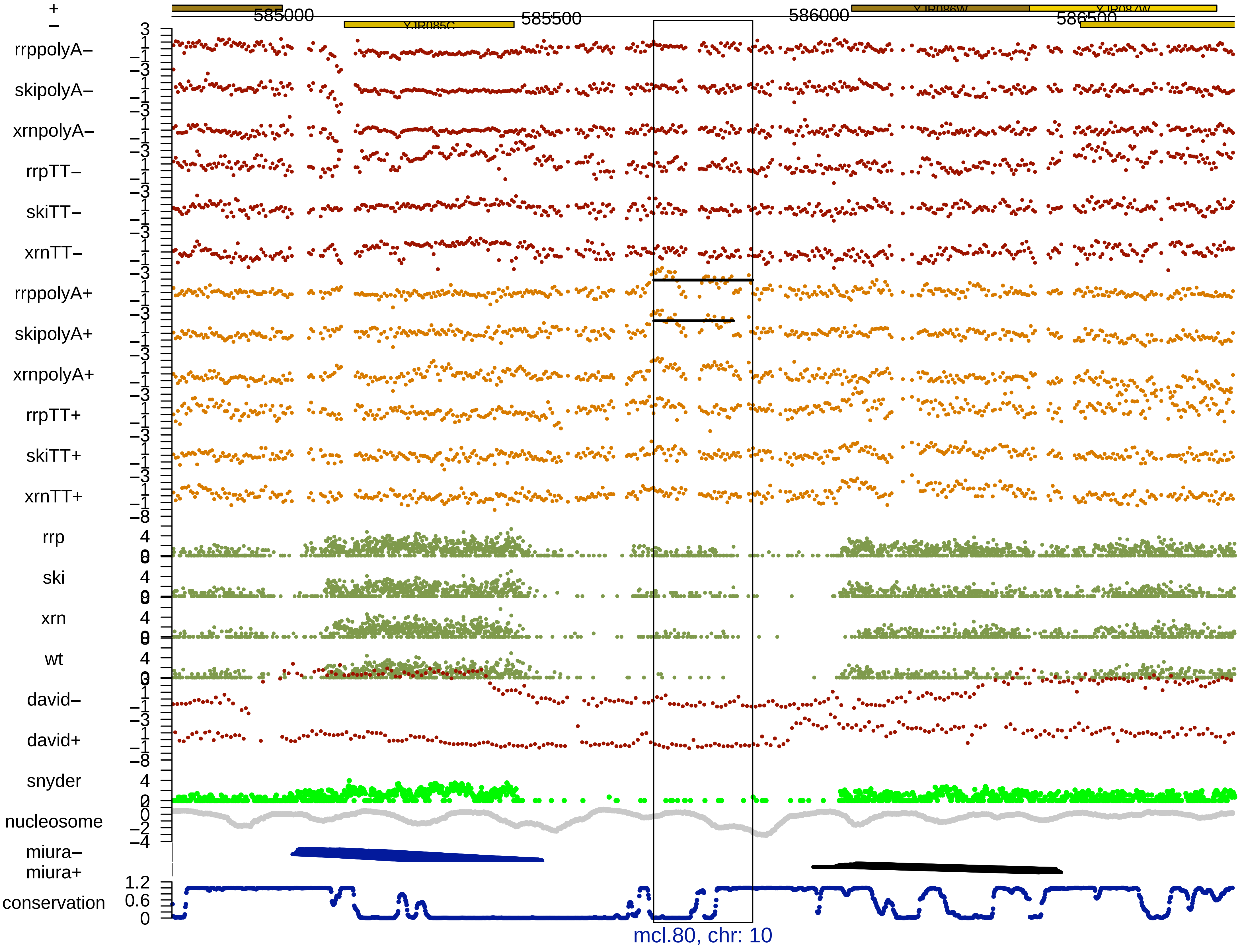
+
−
585000
585500
586000
586500
rrppolyA−
skipolyA−
xrnpolyA−
rrpTT−
skiTT−
xrnTT−
rrppolyA+
skipolyA+
xrnpolyA+
rrpTT+
skiTT+
xrnTT+
rrp
ski
xrn
wt
david−
david+
snyder
nucleosome
miura−
miura+
conservation
mcl.80, chr: 10

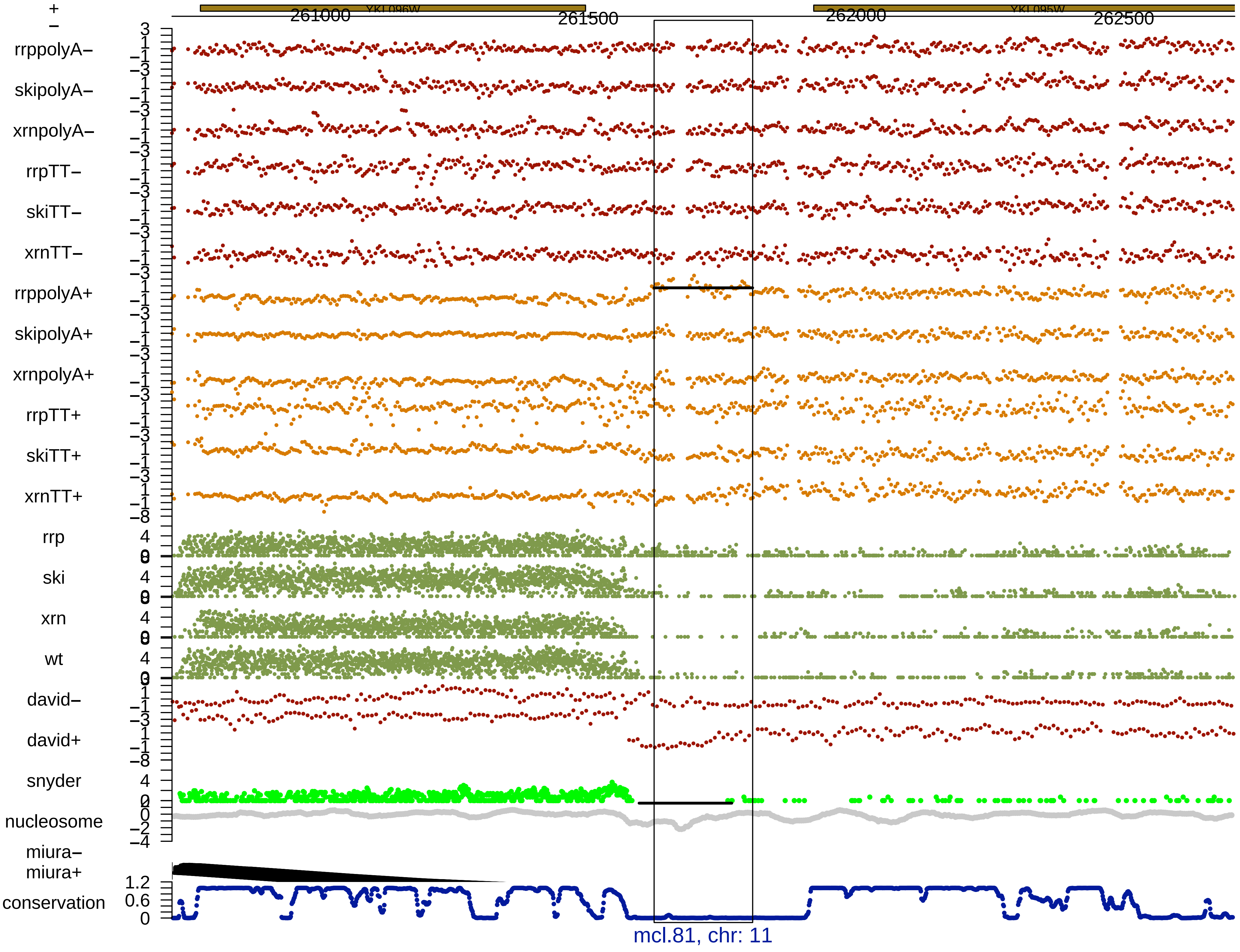

+
–
YKL096W
YKL095W
261000
261500
262000
262500
rrppolyA–
skipolyA–
xrnpolyA–
rrpTT–
skiTT–
xrnTT–
rrppolyA+
skipolyA+
xrnpolyA+
rrpTT+
skiTT+
xrnTT+
rrp
ski
xrn
wt
david–
david+
snyder
nucleosome
miura–
miura+
conservation
mcl.81, chr: 11

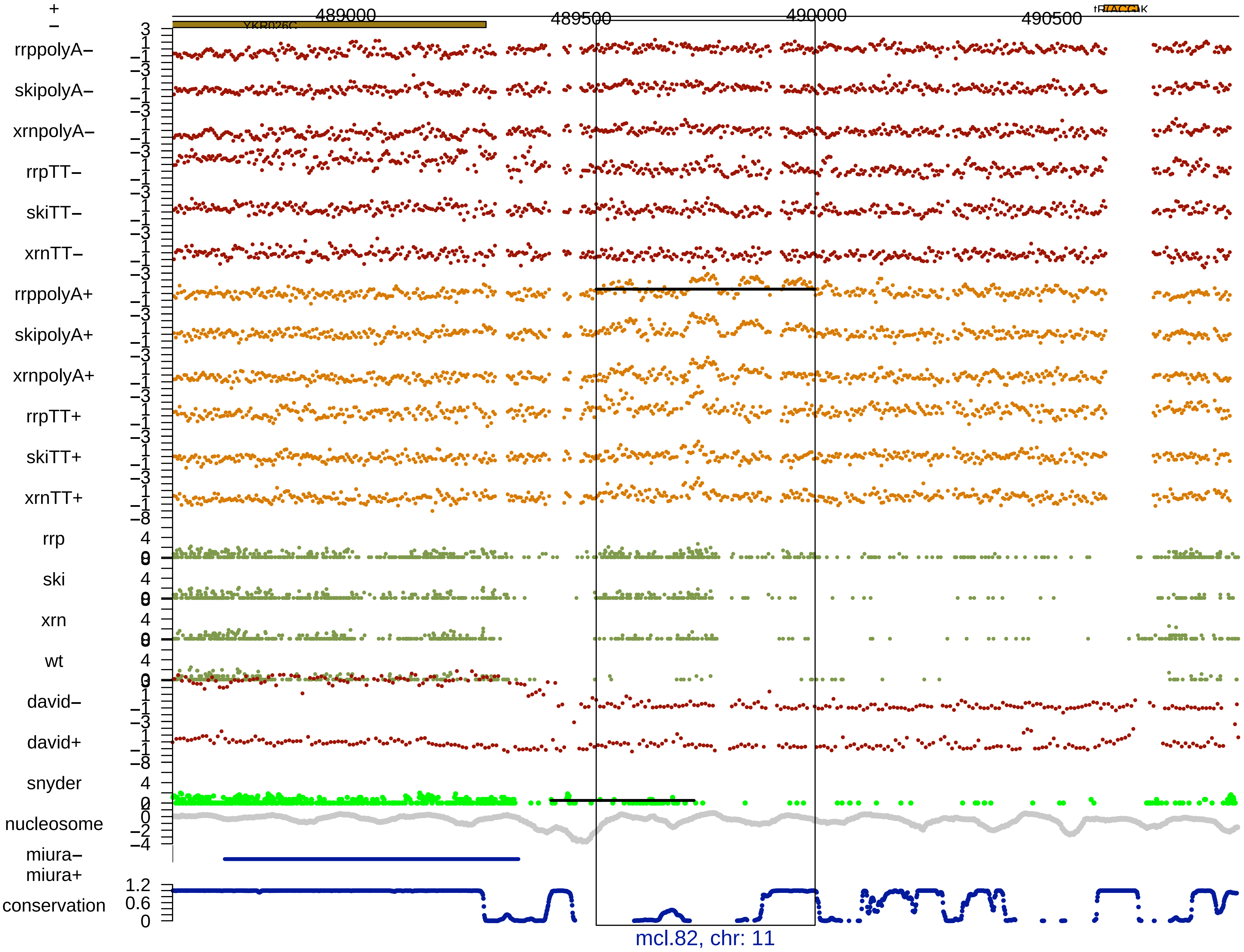

489000
489500
490000
490500
YKR026C
rrppolyA−
skipolyA−
xrnpolyA−
rrpTT−
skiTT−
xrnTT−
rrppolyA+
skipolyA+
xrnpolyA+
rrpTT+
skiTT+
xrnTT+
rrp
ski
xrn
wt
david−
david+
snyder
nucleosome
miura−
miura+
conservation
mcl.82, chr: 11

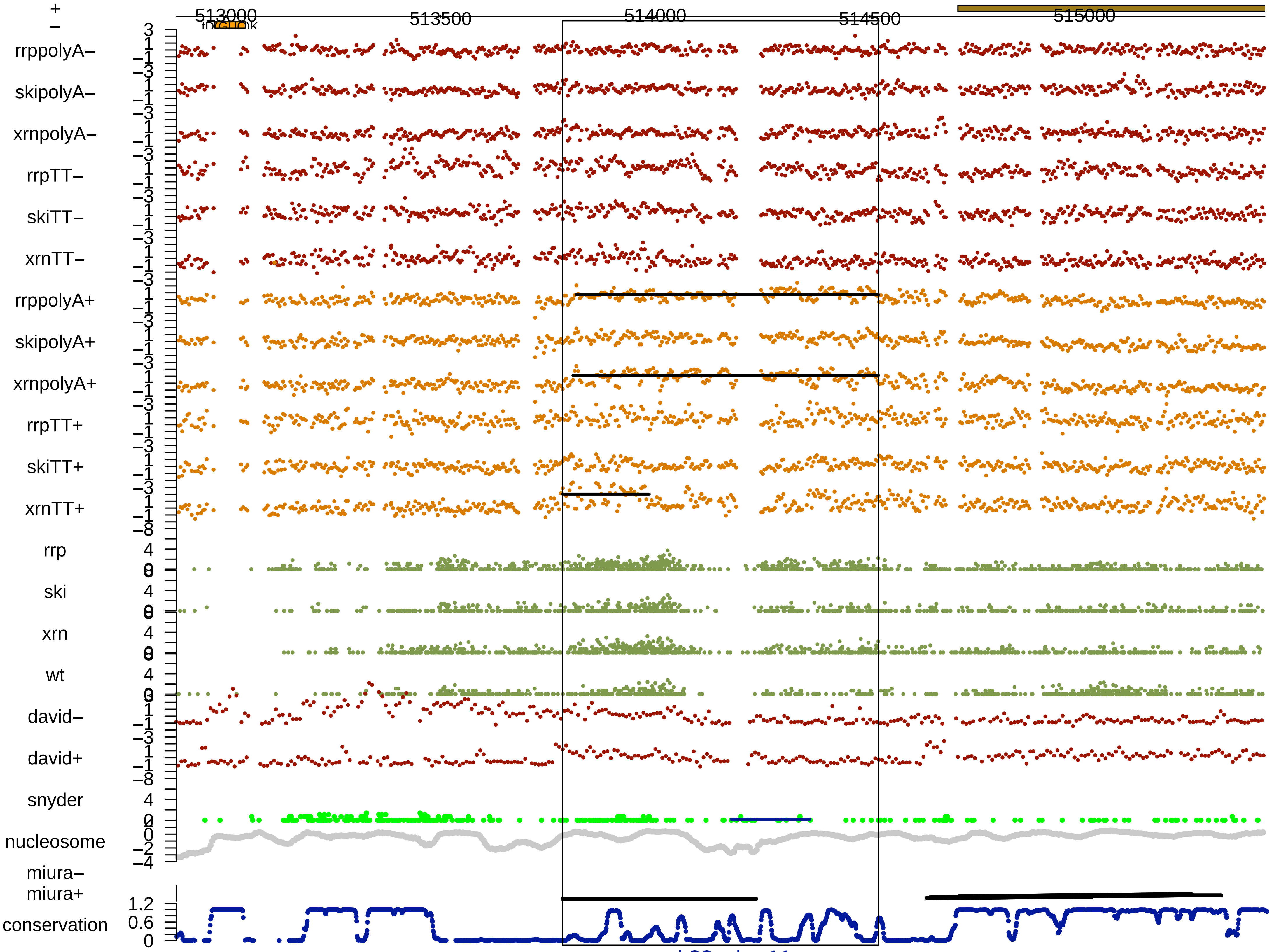

513000
513500
514000
514500
515000
rrppolyA−
skipolyA−
xrnpolyA−
rrpTT−
skiTT−
xrnTT−
rrppolyA+
skipolyA+
xrnpolyA+
rrpTT+
skiTT+
xrnTT+
rrp
ski
xrn
wt
david−
david+
snyder
nucleosome
miura−
miura+
conservation

mcl.83, chr: 11

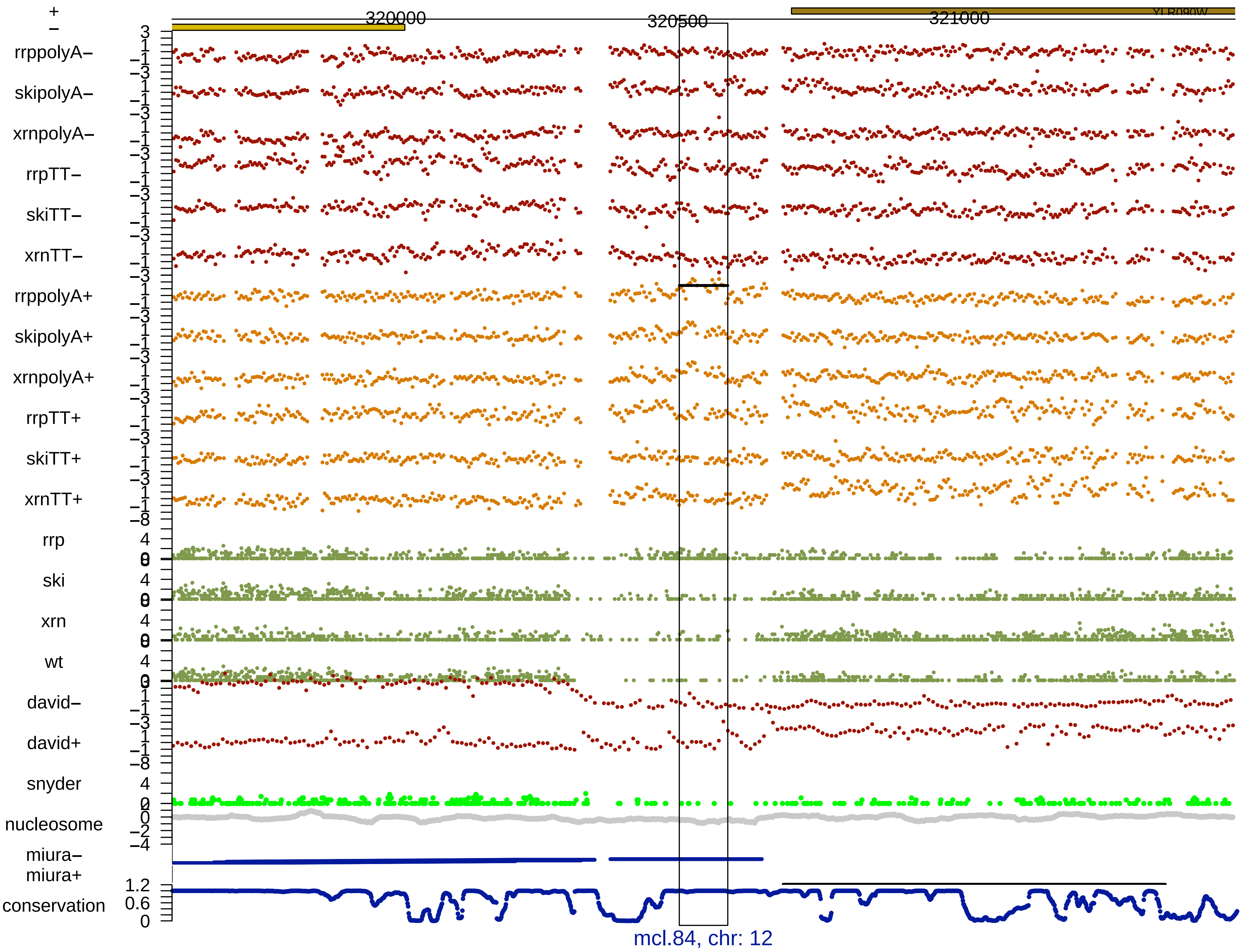

320000
320500
321000
YLR090W
rrppolyA−
skipolyA−
xrnpolyA−
rrpTT−
skiTT−
xrnTT−
rrppolyA+
skipolyA+
xrnpolyA+
rrpTT+
skiTT+
xrnTT+
rrp
ski
xrn
wt
david−
david+
snyder
nucleosome
miura−
miura+
conservation
mcl.84, chr: 12

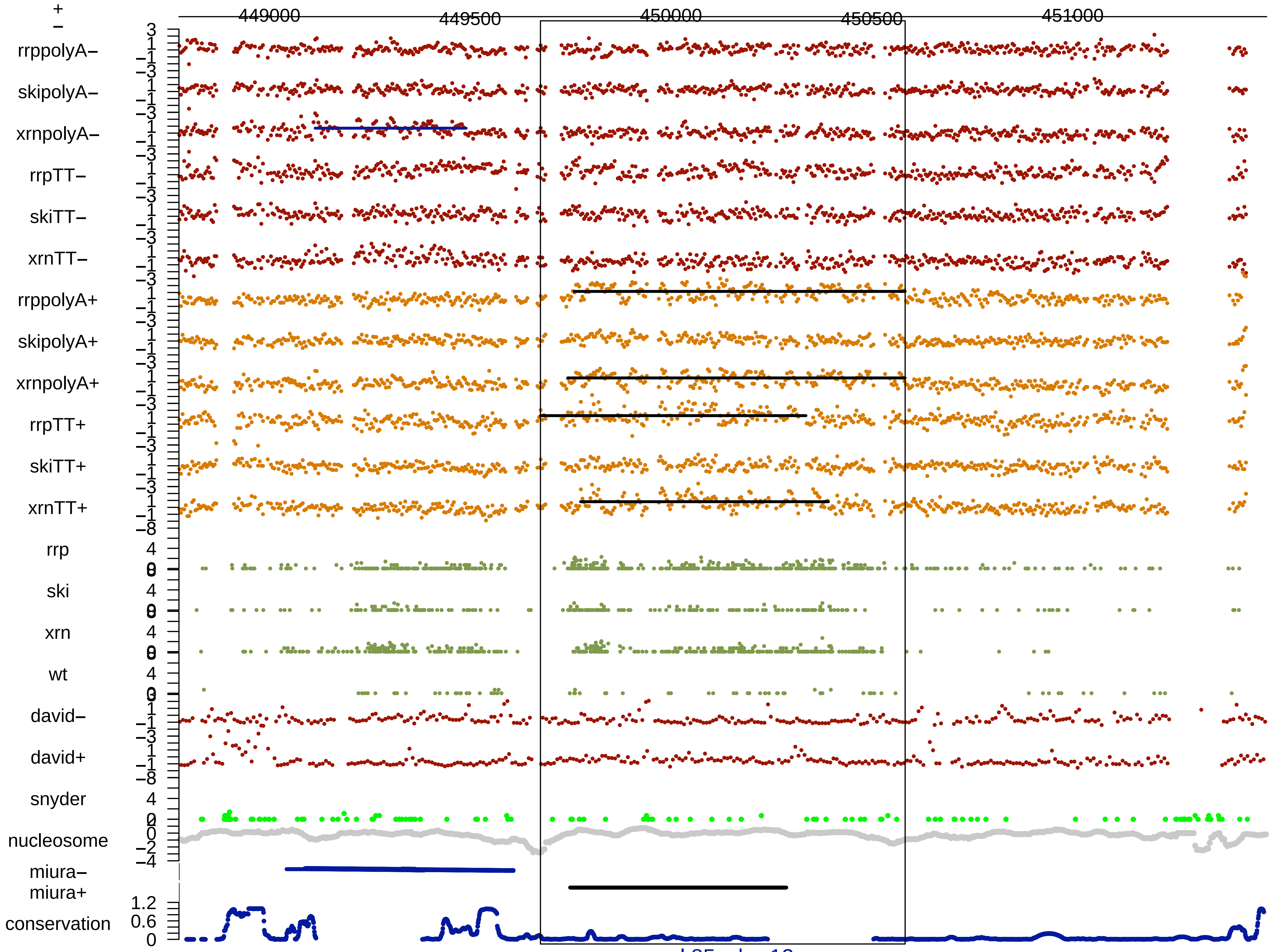
449000
449500
450000
450500
451000
rrppolyA−
skipolyA−
xrnpolyA−
rrpTT−
skiTT−
xrnTT−
rrppolyA+
skipolyA+
xrnpolyA+
rrpTT+
skiTT+
xrnTT+
rrp
ski
xrn
wt
david−
david+
snyder
nucleosome
miura−
miura+
conservation
mcl.85, chr: 12

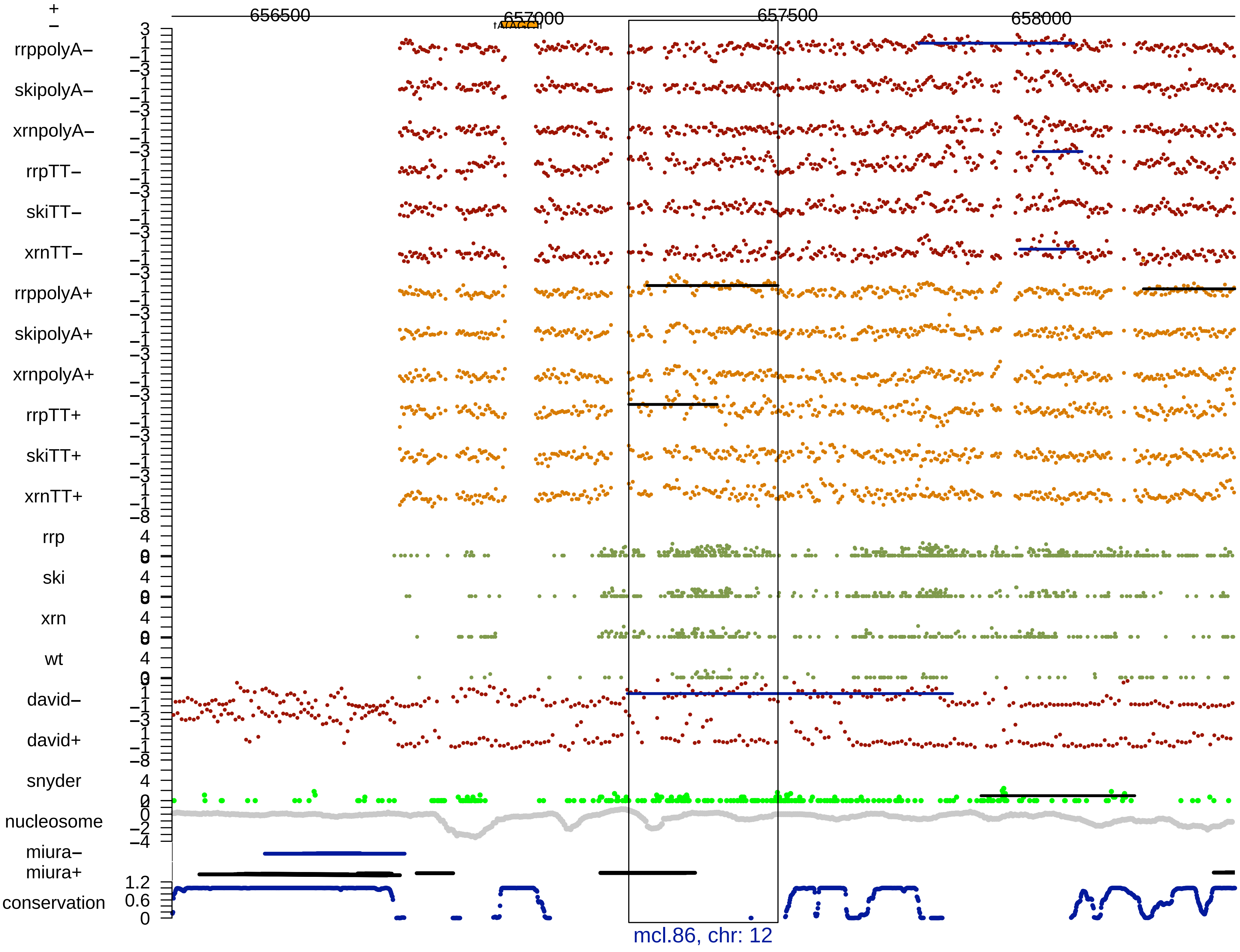

656500
657000
657500
658000
rrppolyA−
skipolyA−
xrnpolyA−
rrpTT−
skiTT−
xrnTT−
rrppolyA+
skipolyA+
xrnpolyA+
rrpTT+
skiTT+
xrnTT+
rrp
ski
xrn
wt
david−
david+
snyder
nucleosome
miura−
miura+
conservation
mcl.86, chr: 12

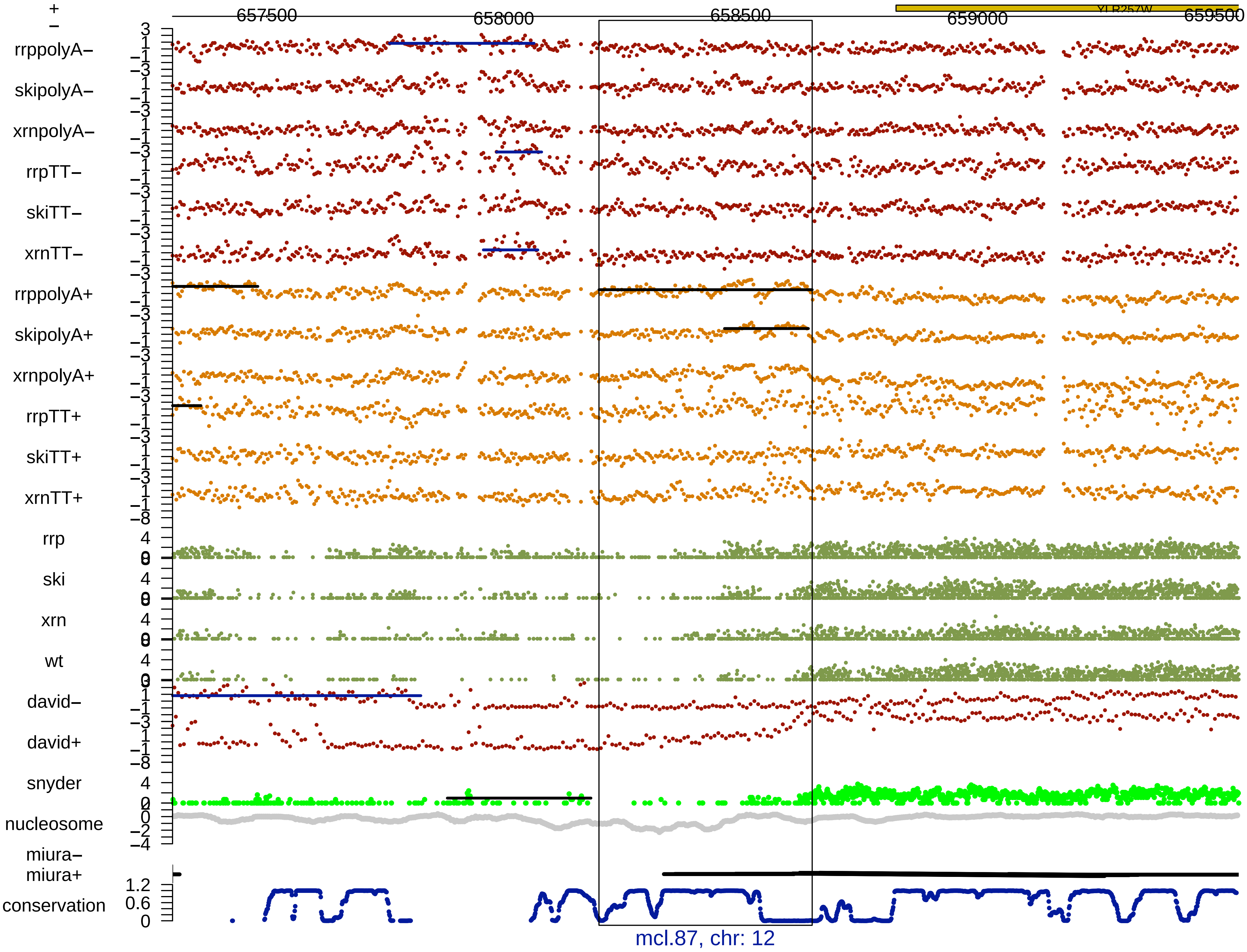

+
−
657500
658000
658500
659000
659500
YLR257W
rrppolyA−
skipolyA−
xrnpolyA−
rrpTT−
skiTT−
xrnTT−
rrppolyA+
skipolyA+
xrnpolyA+
rrpTT+
skiTT+
xrnTT+
rrp
ski
xrn
wt
david−
david+
snyder
nucleosome
miura−
miura+
conservation
mcl.87, chr: 12

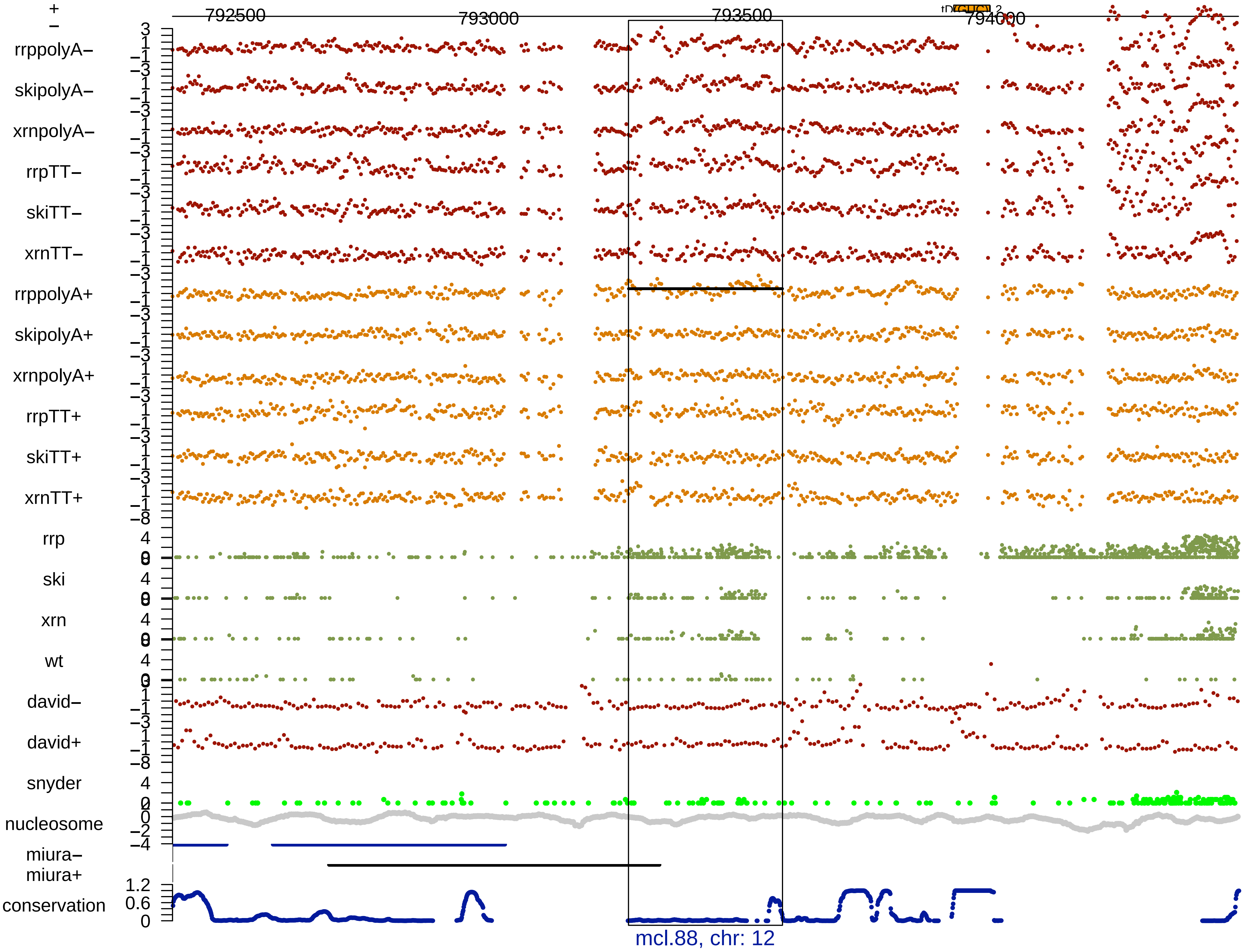
792500
793000
793500
794000
rrppolyA−
skipolyA−
xrnpolyA−
rrpTT−
skiTT−
xrnTT−
rrppolyA+
skipolyA+
xrnpolyA+
rrpTT+
skiTT+
xrnTT+
rrp
ski
xrn
wt
david−
david+
snyder
nucleosome
miura−
miura+
conservation
mcl.88, chr: 12

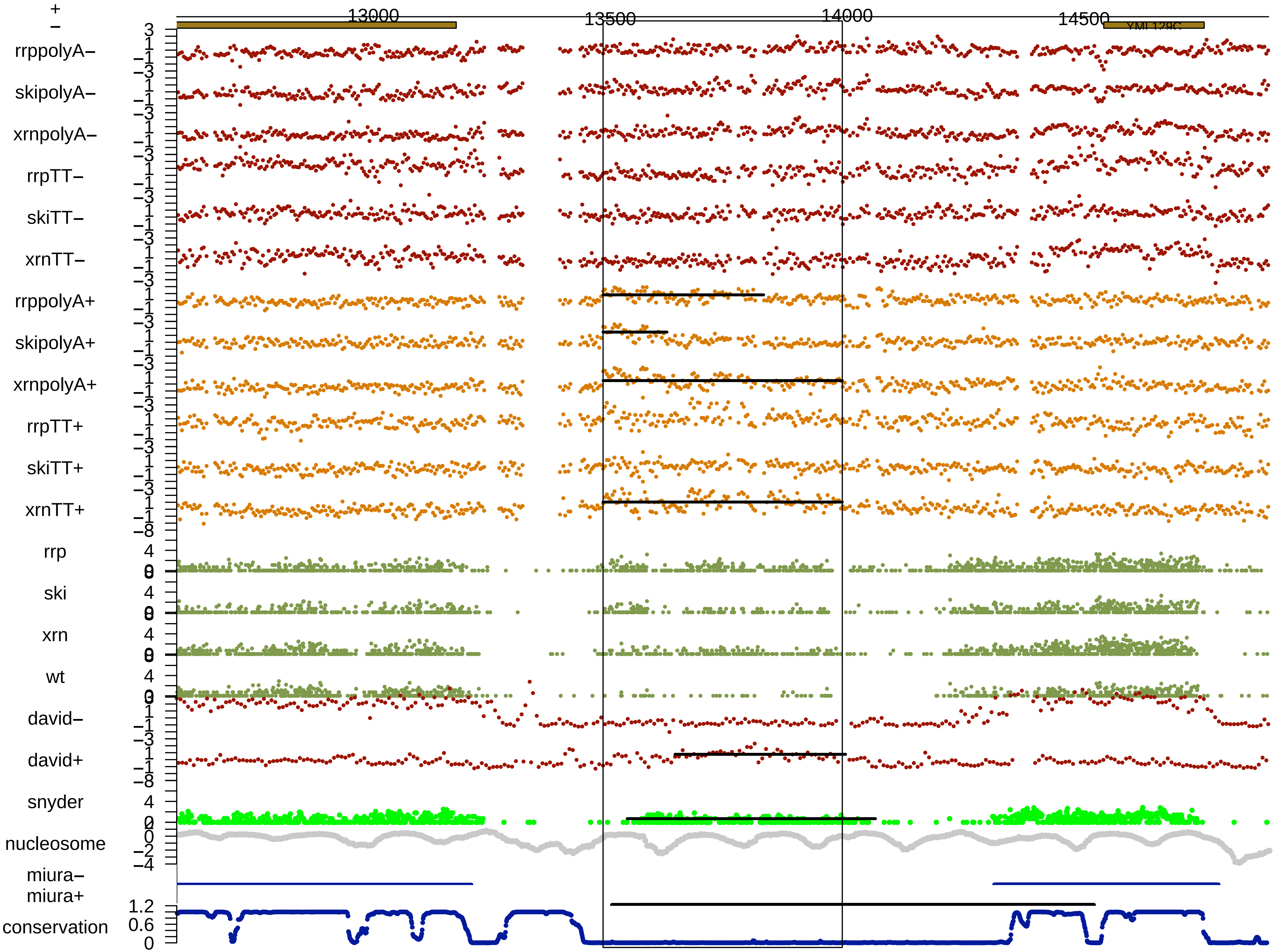

+
−
13000
13500
14000
14500
YML129C
rrppolyA−
skipolyA−
xrnpolyA−
rrpTT−
skiTT−
xrnTT−
rrppolyA+
skipolyA+
xrnpolyA+
rrpTT+
skiTT+
xrnTT+
rrp
ski
xrn
wt
david−
david+
snyder
nucleosome
miura−
miura+
conservation

mcl.89, chr: 13

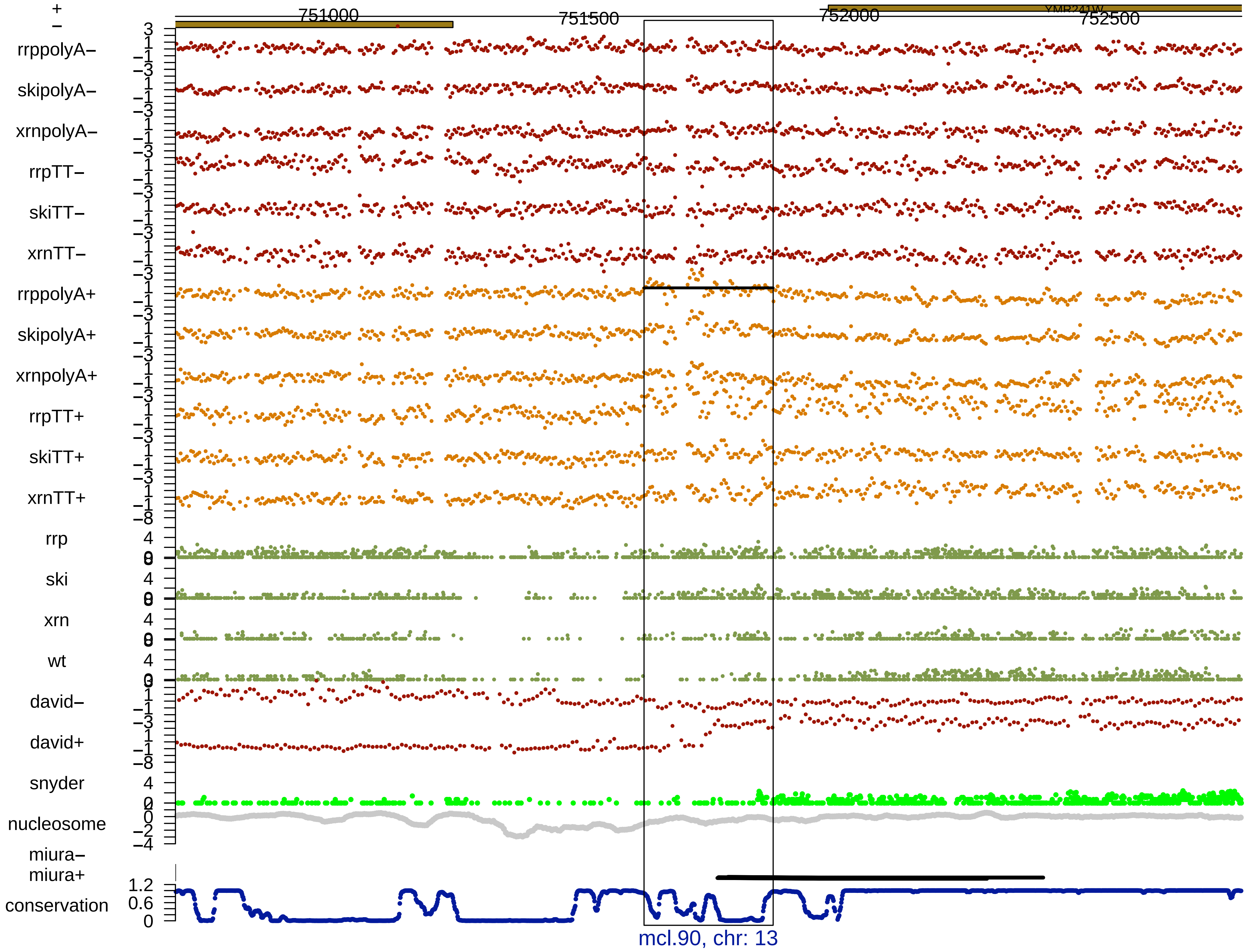
+
–
751000
751500
752000
752500
YMR241W
rrppolyA–
skipolyA–
xrnpolyA–
rrpTT–
skiTT–
xrnTT–
rrppolyA+
skipolyA+
xrnpolyA+
rrpTT+
skiTT+
xrnTT+
rrp
ski
xrn
wt
david–
david+
snyder
nucleosome
miura–
miura+
conservation
mcl.90, chr: 13

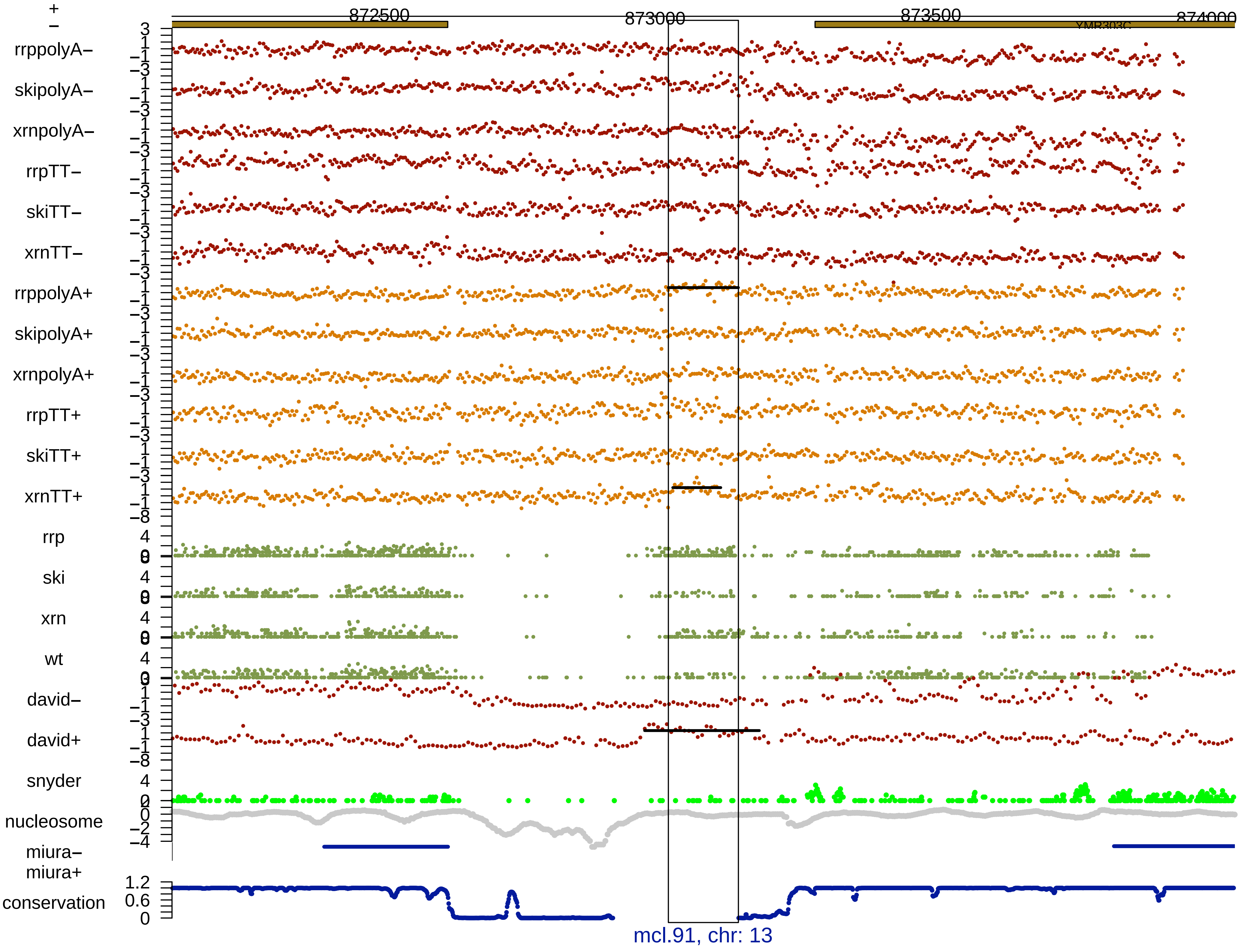

872500
873000
873500
874000
YMR303C
rrppolyA−
skipolyA−
xrnpolyA−
rrpTT−
skiTT−
xrnTT−
rrppolyA+
skipolyA+
xrnpolyA+
rrpTT+
skiTT+
xrnTT+
rrp
ski
xrn
wt
david−
david+
snyder
nucleosome
miura−
miura+
conservation
mcl.91, chr: 13

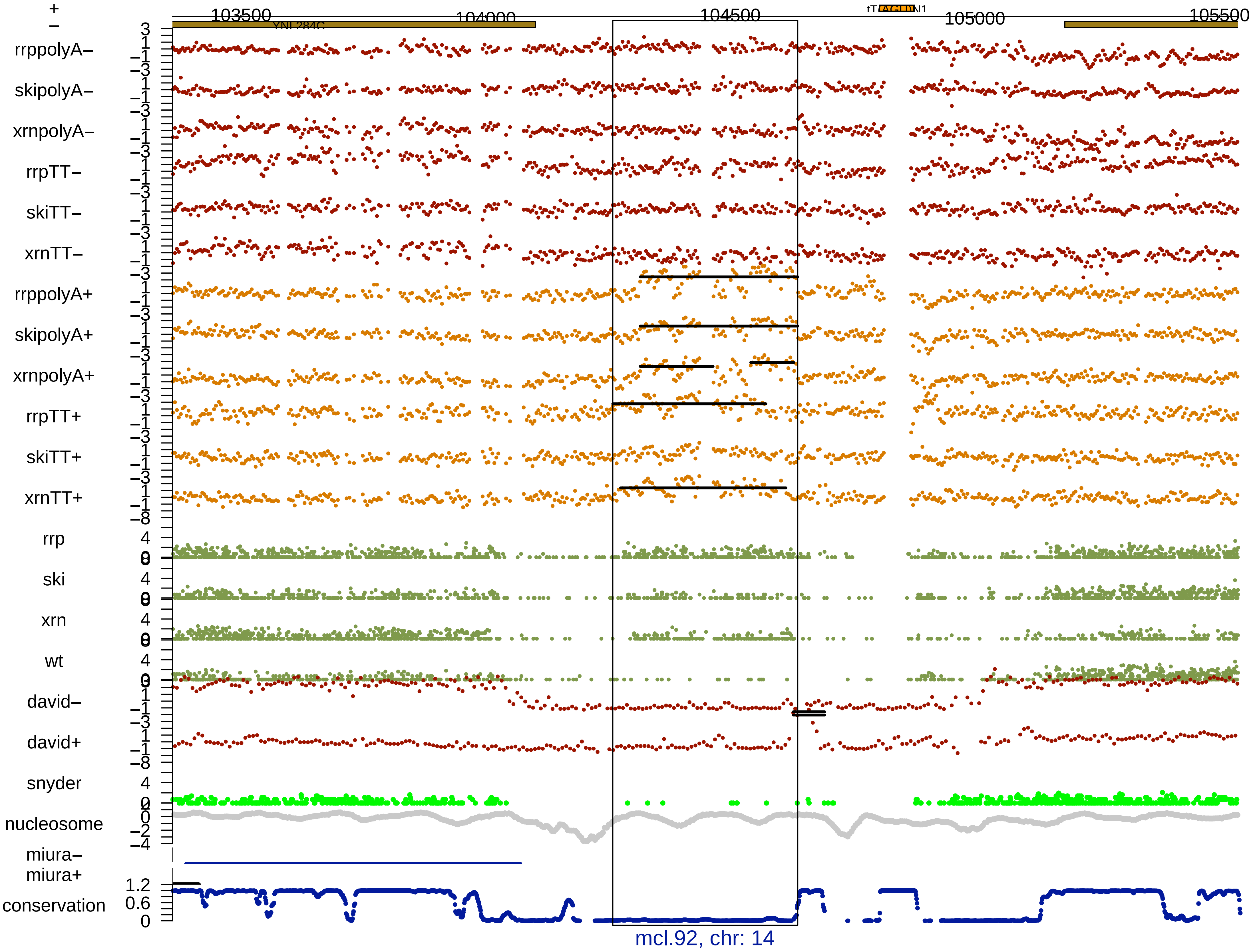

+
−
103500
104000
104500
105000
105500
YNL284C
rrppolyA−
skipolyA−
xrnpolyA−
rrpTT−
skiTT−
xrnTT−
rrppolyA+
skipolyA+
xrnpolyA+
rrpTT+
skiTT+
xrnTT+
rrp
ski
xrn
wt
david−
david+
snyder
nucleosome
miura−
miura+
conservation
mcl.92, chr: 14

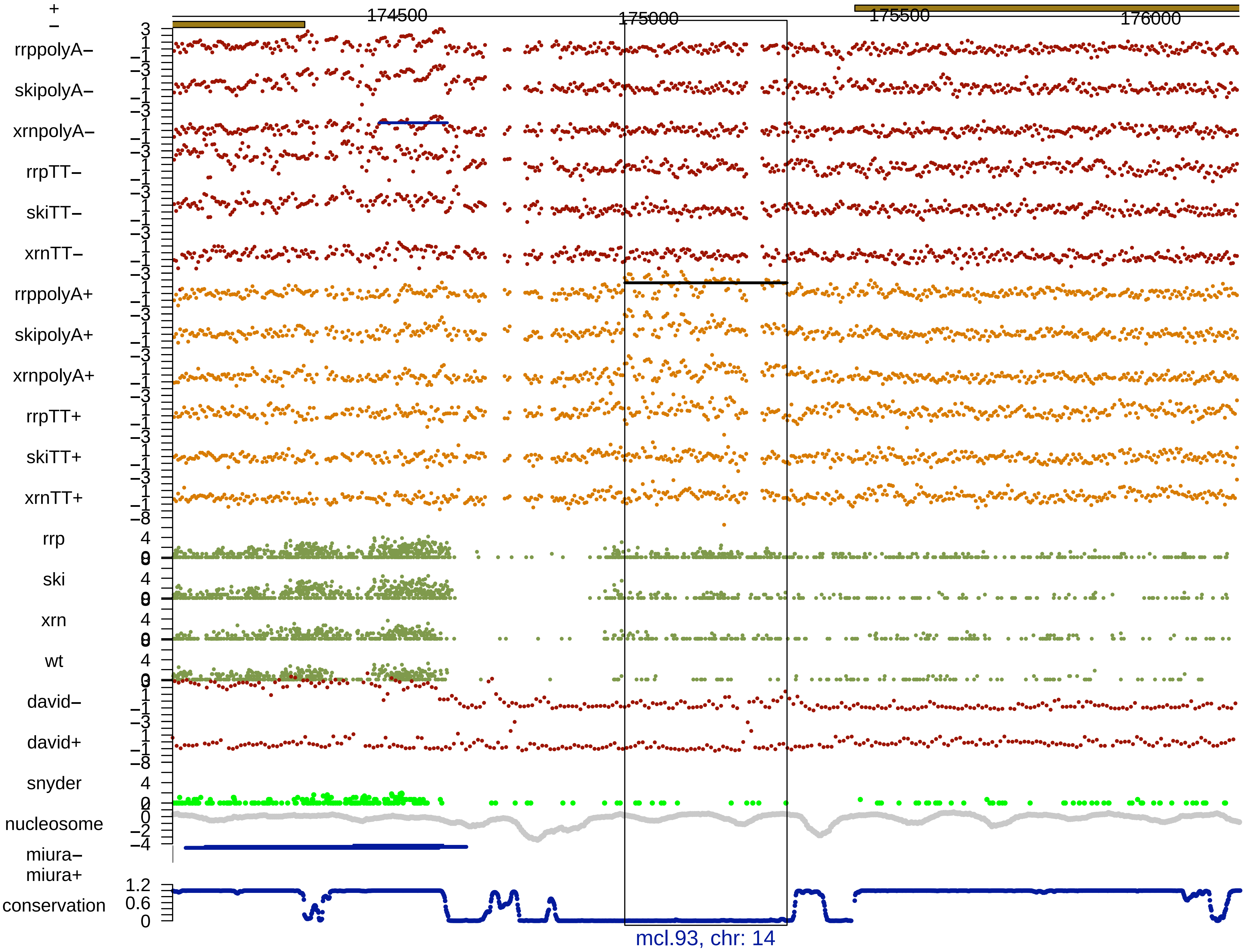

+
−
174500
175000
175500
176000
rrppolyA−
skipolyA−
xrnpolyA−
rrpTT−
skiTT−
xrnTT−
rrppolyA+
skipolyA+
xrnpolyA+
rrpTT+
skiTT+
xrnTT+
rrp
ski
xrn
wt
david−
david+
snyder
nucleosome
miura−
miura+
conservation
mcl.93, chr: 14

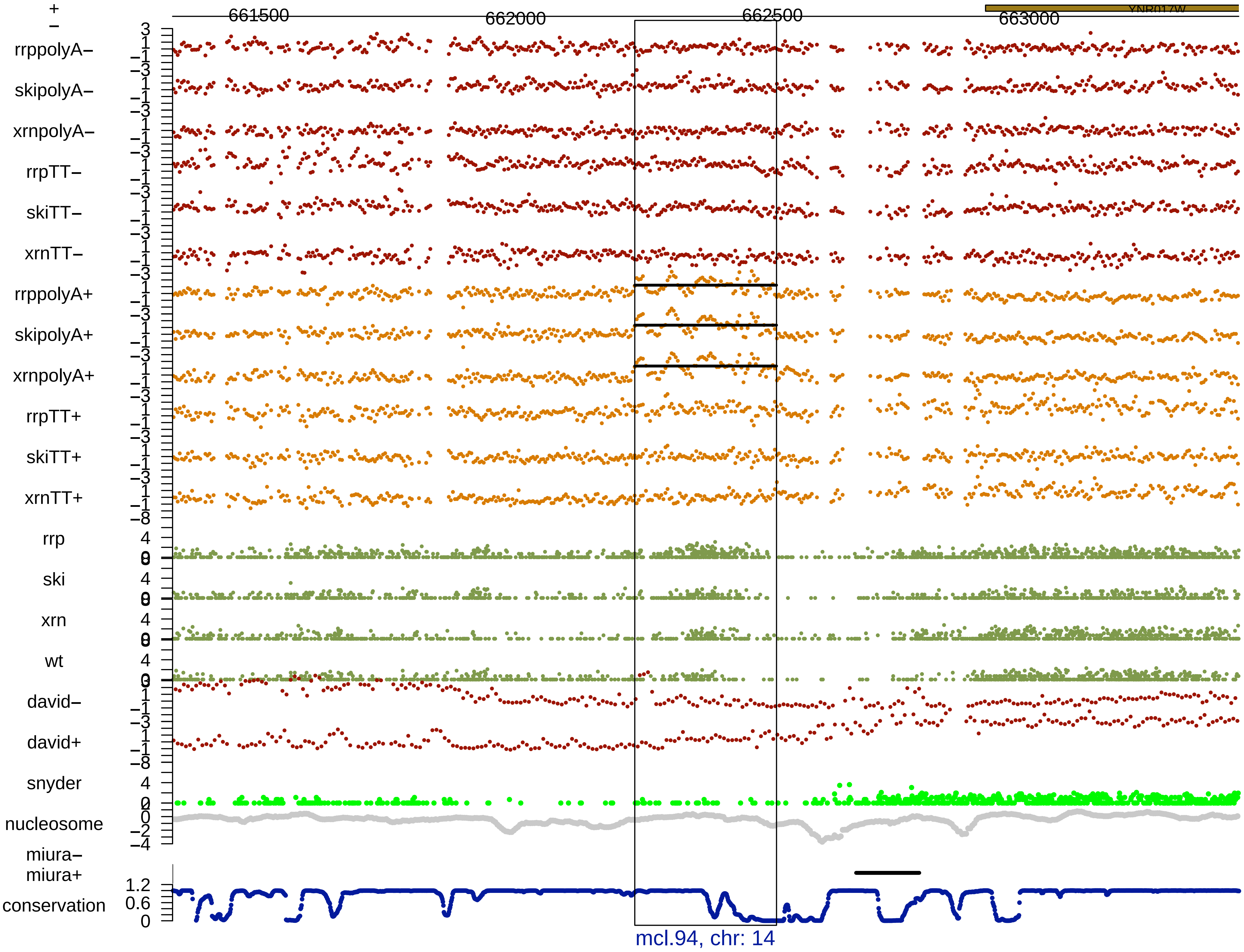

+
−
661500
662000
662500
663000
YNR017W
rrppolyA−
skipolyA−
xrnpolyA−
rrpTT−
skiTT−
xrnTT−
rrppolyA+
skipolyA+
xrnpolyA+
rrpTT+
skiTT+
xrnTT+
rrp
ski
xrn
wt
david−
david+
snyder
nucleosome
miura−
miura+
conservation
mcl.94, chr: 14

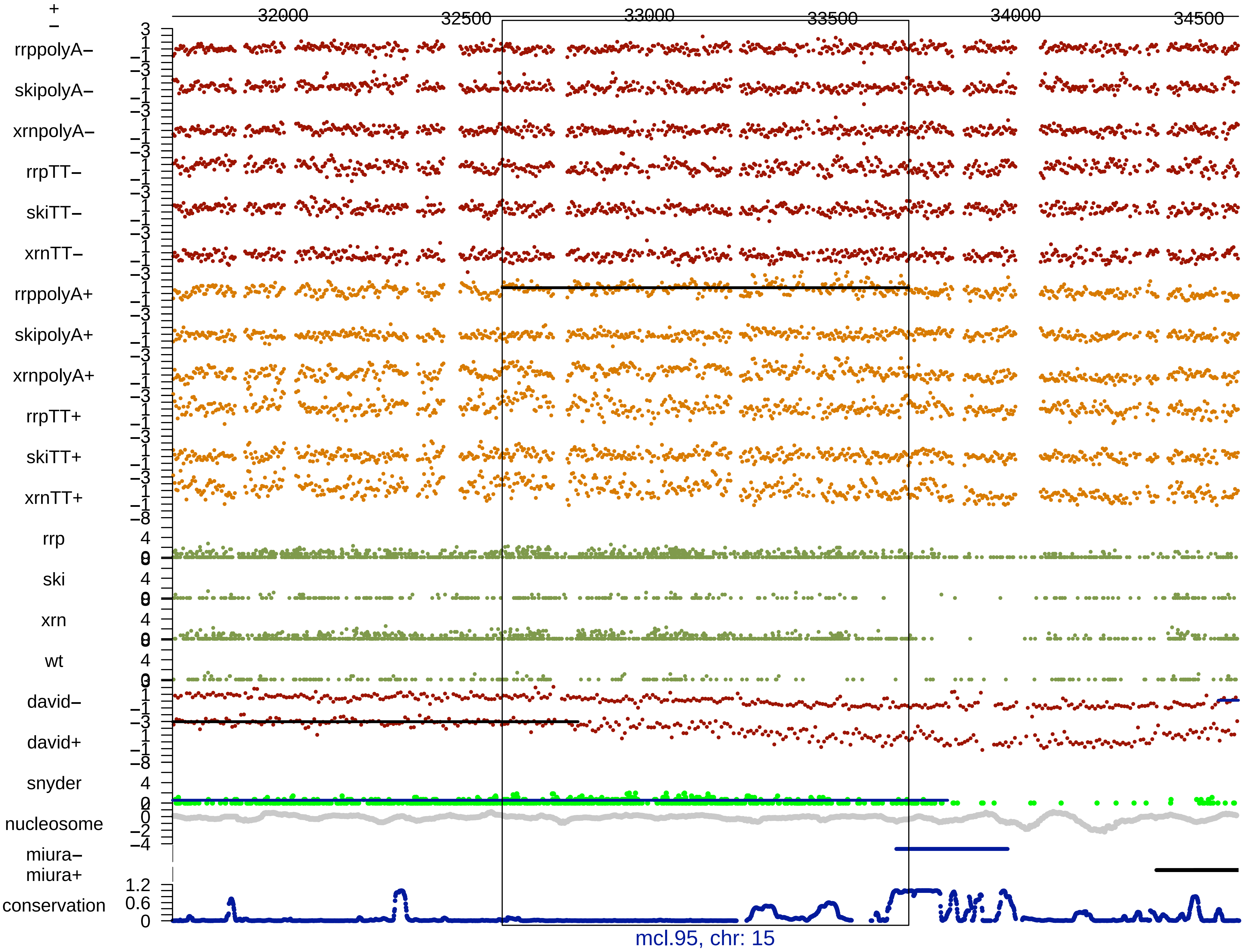

+
–
32000
32500
33000
33500
34000
34500
rrppolyA–
skipolyA–
xrnpolyA–
rrpTT–
skiTT–
xrnTT–
rrppolyA+
skipolyA+
xrnpolyA+
rrpTT+
skiTT+
xrnTT+
rrp
ski
xrn
wt
david–
david+
snyder
nucleosome
miura–
miura+
conservation
mcl.95, chr: 15

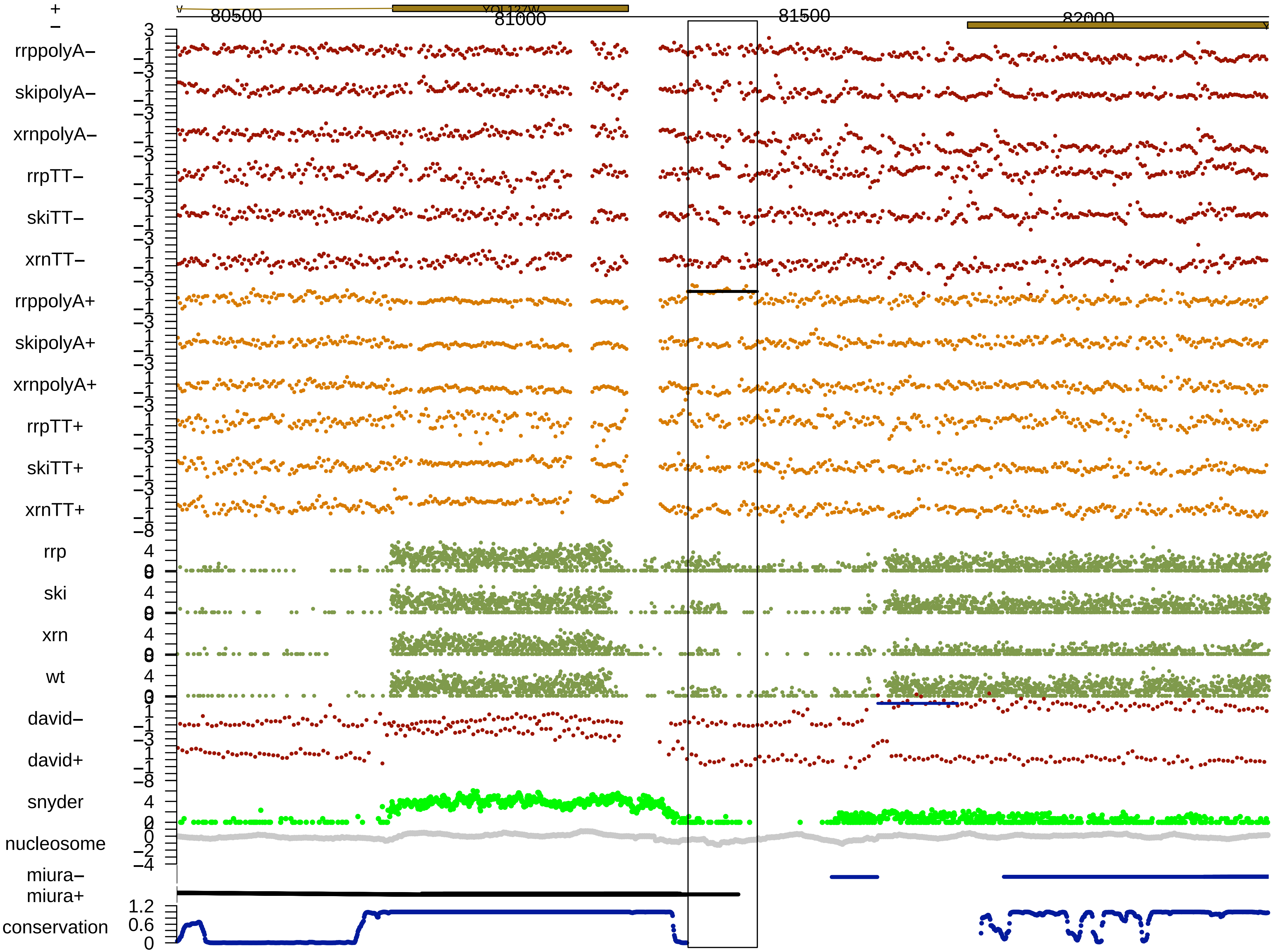
+
–
80500
81000
81500
rrppolyA–
skipolyA–
xrnpolyA–
rrpTT–
skiTT–
xrnTT–
rrppolyA+
skipolyA+
xrnpolyA+
rrpTT+
skiTT+
xrnTT+
rrp
ski
xrn
wt
david–
david+
snyder
nucleosome
miura–
miura+
conservation

mcl.96, chr: 15

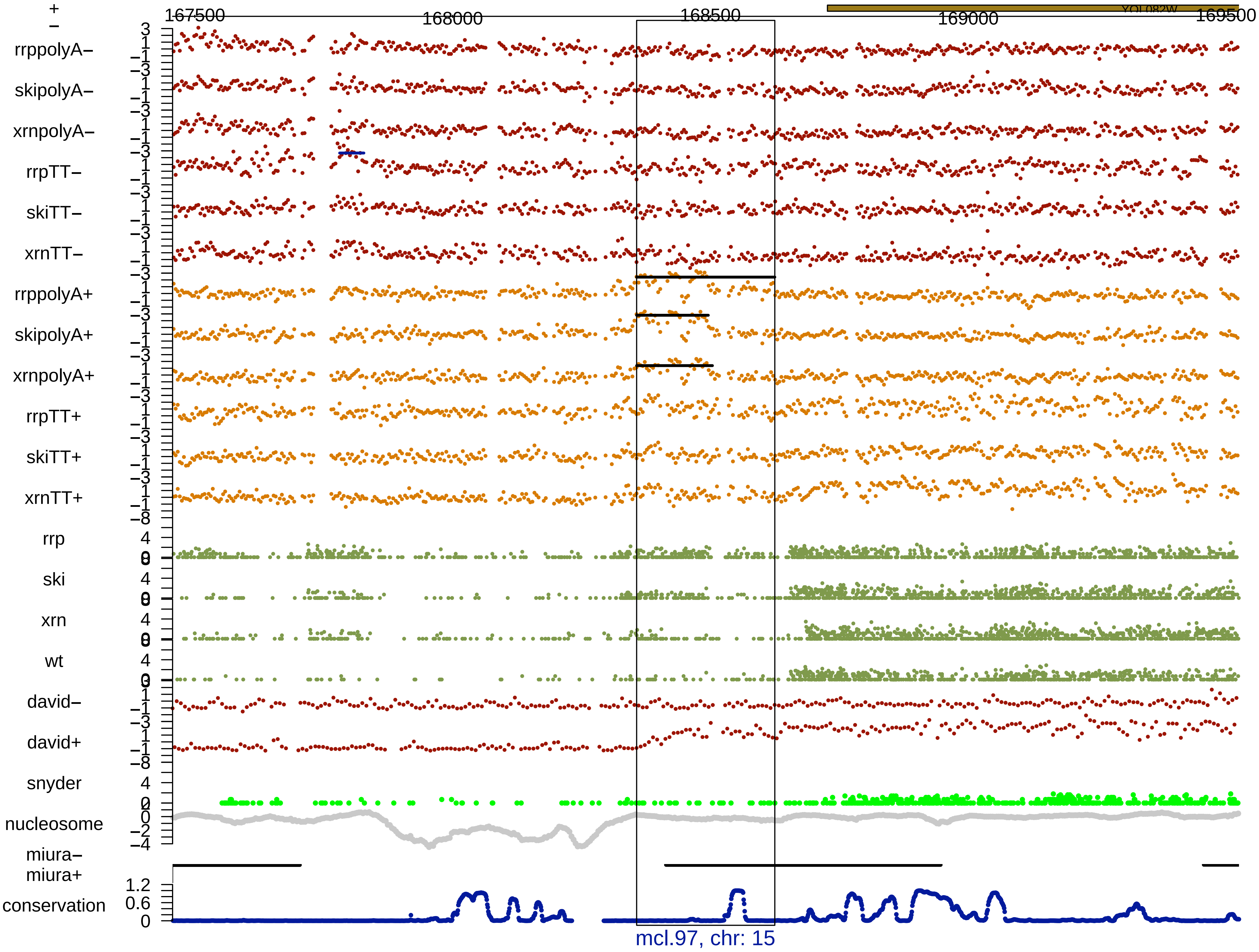

167500
168000
168500
169000
169500
YOL082W
rrppolyA–
skipolyA–
xrnpolyA–
rrpTT–
skiTT–
xrnTT–
rrppolyA+
skipolyA+
xrnpolyA+
rrpTT+
skiTT+
xrnTT+
rrp
ski
xrn
wt
david–
david+
snyder
nucleosome
miura–
miura+
conservation
mcl.97, chr: 15

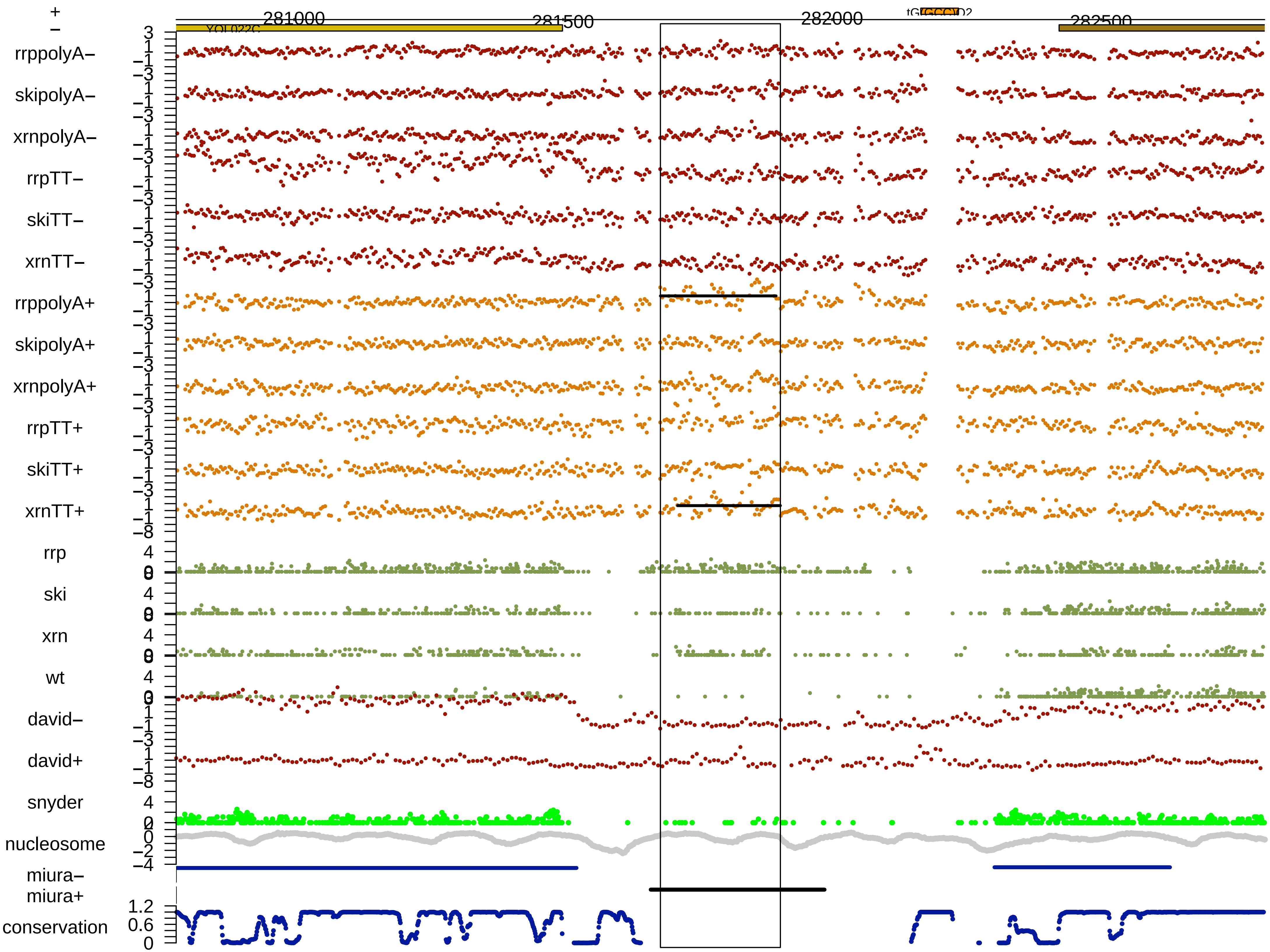

281000
281500
282000
282500
YOL022C
tG(GCC)O2
rrppolyA−
skipolyA−
xrnpolyA−
rrpTT−
skiTT−
xrnTT−
rrppolyA+
skipolyA+
xrnpolyA+
rrpTT+
skiTT+
xrnTT+
rrp
ski
xrn
wt
david−
david+
snyder
nucleosome
miura−
miura+
conservation

mcl.98, chr: 15

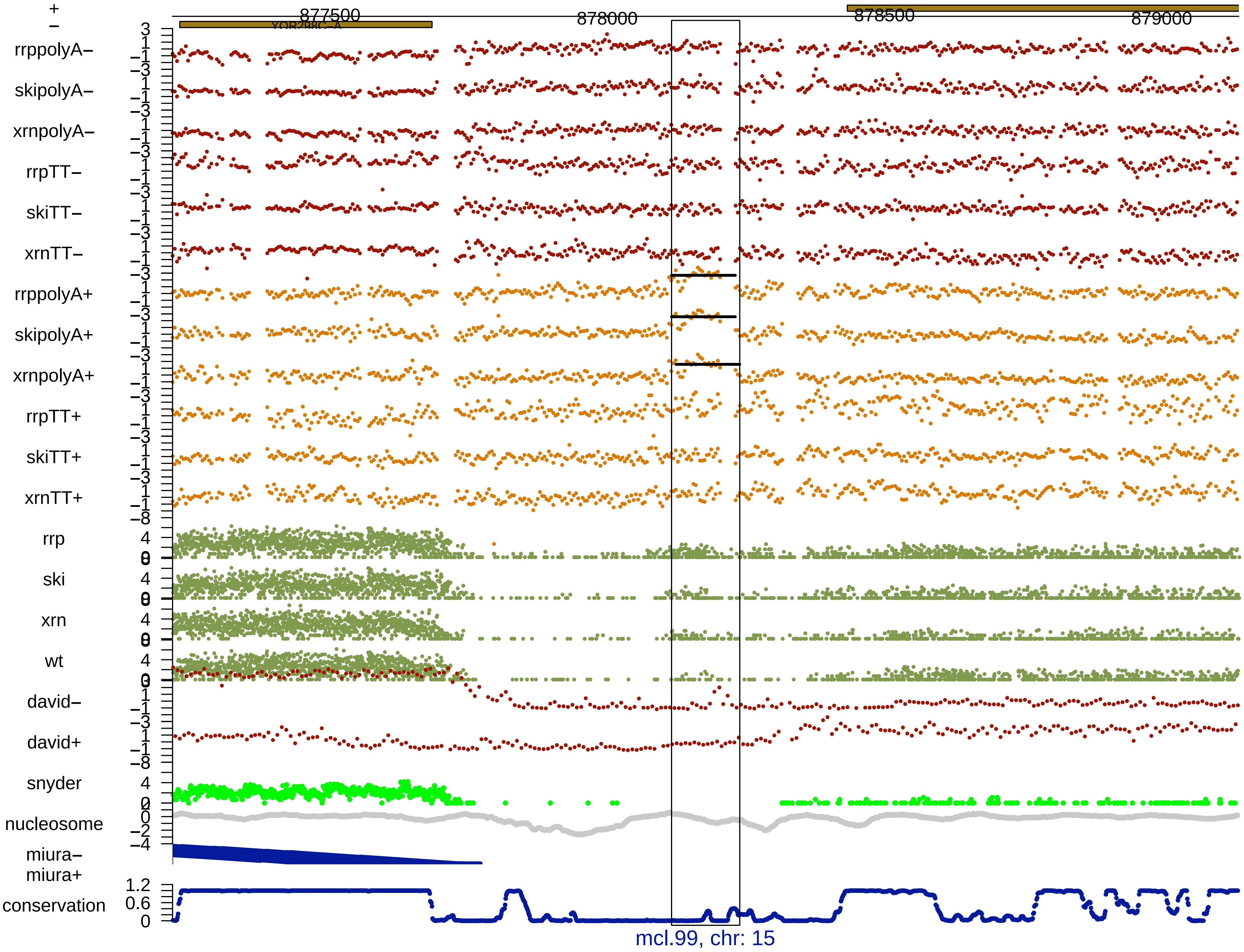

877500
878000
878500
879000
YOR298C-A
rrppolyA−
skipolyA−
xrnpolyA−
rrpTT−
skiTT−
xrnTT−
rrppolyA+
skipolyA+
xrnpolyA+
rrpTT+
skiTT+
xrnTT+
rrp
ski
xrn
wt
david−
david+
snyder
nucleosome
miura−
miura+
conservation
mcl.99, chr: 15

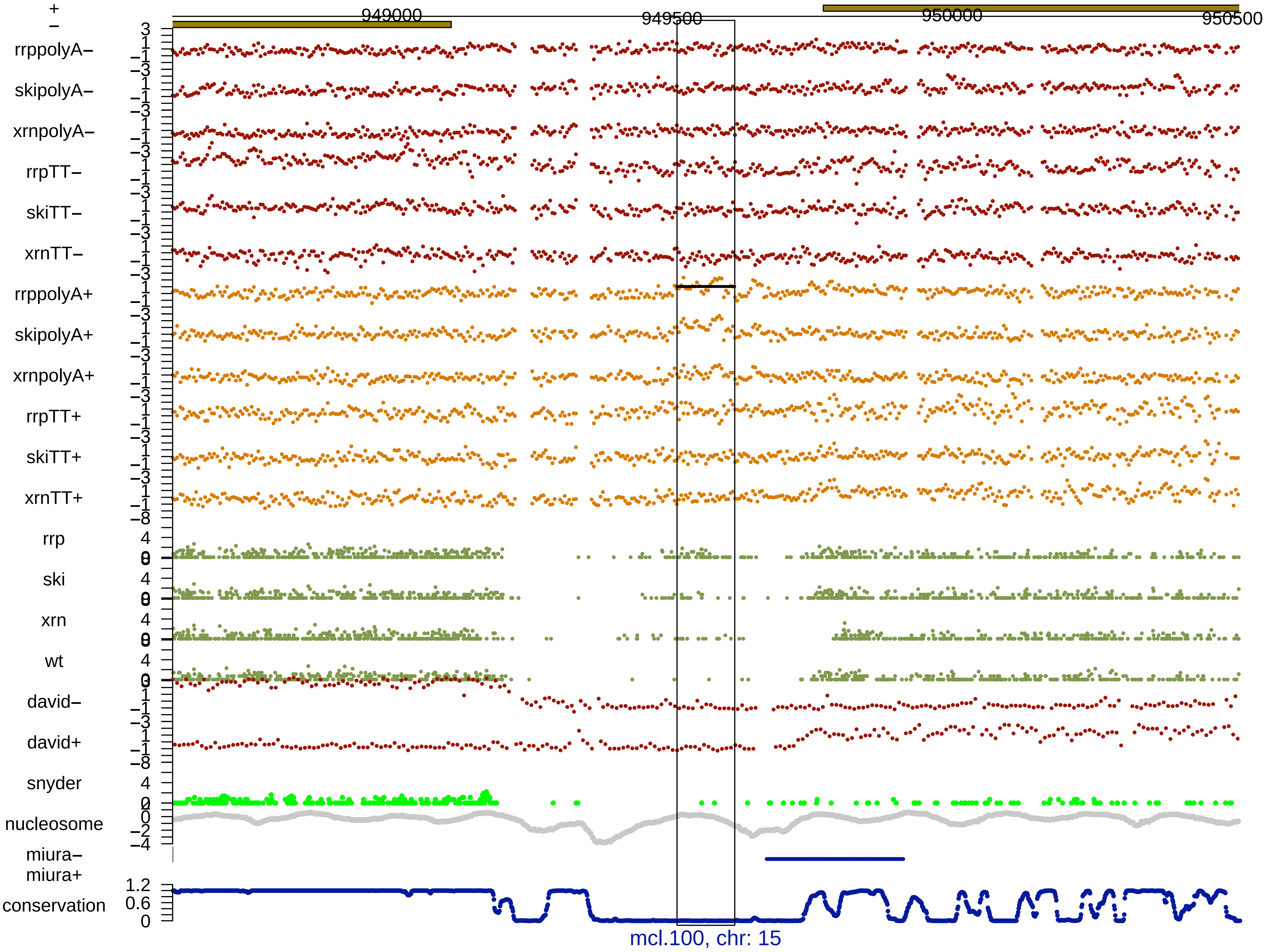
+
−
949000
949500
950000
950500
rrppolyA−
skipolyA−
xrnpolyA−
rrpTT−
skiTT−
xrnTT−
rrppolyA+
skipolyA+
xrnpolyA+
rrpTT+
skiTT+
xrnTT+
rrp
ski
xrn
wt
david−
david+
snyder
nucleosome
miura−
miura+
conservation
mcl.100, chr: 15

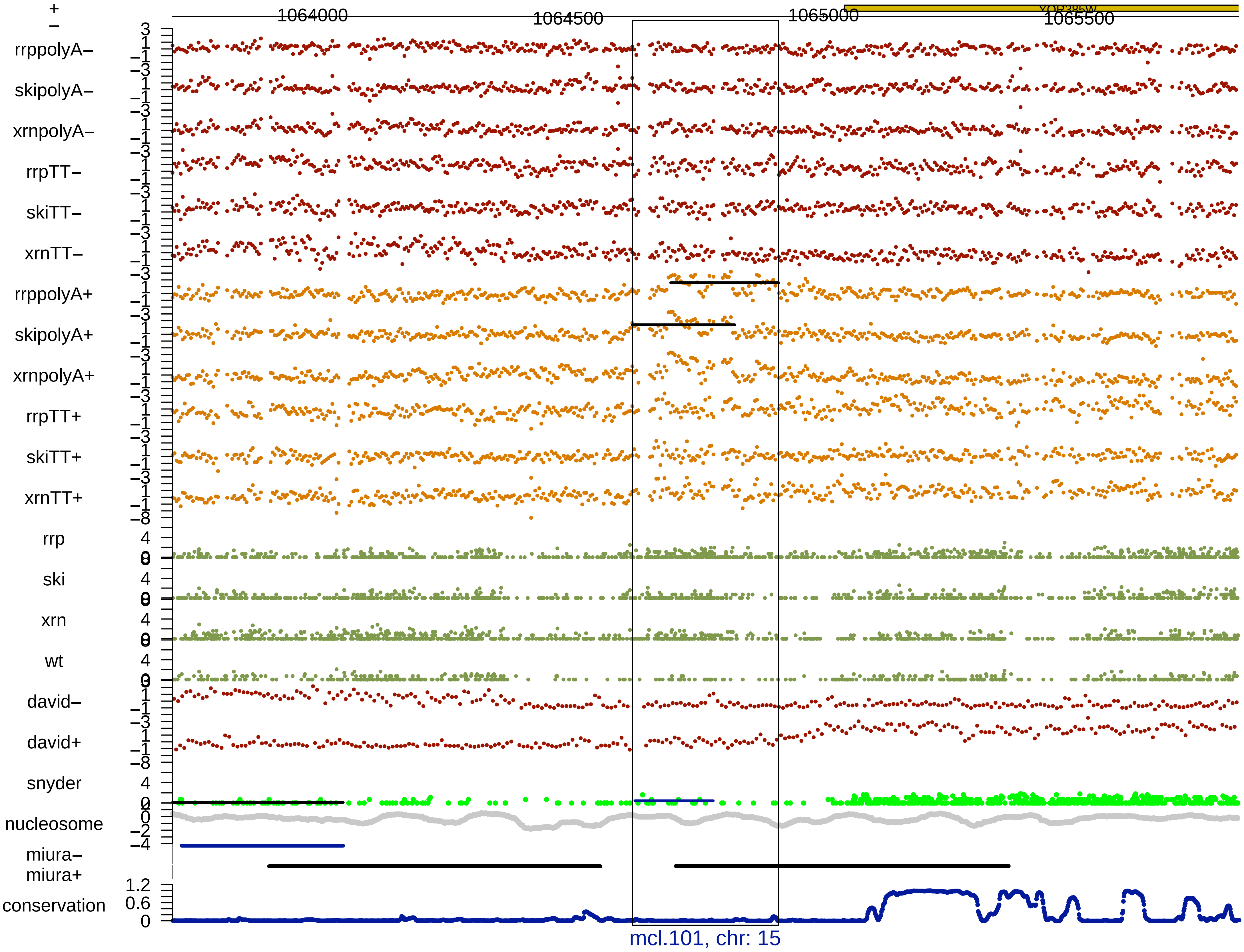

+
−
1064000
1064500
1065000
1065500
YOR385W
rrppolyA−
skipolyA−
xrnpolyA−
rrpTT−
skiTT−
xrnTT−
rrppolyA+
skipolyA+
xrnpolyA+
rrpTT+
skiTT+
xrnTT+
rrp
ski
xrn
wt
david−
david+
snyder
nucleosome
miura−
miura+
conservation
mcl.101, chr: 15

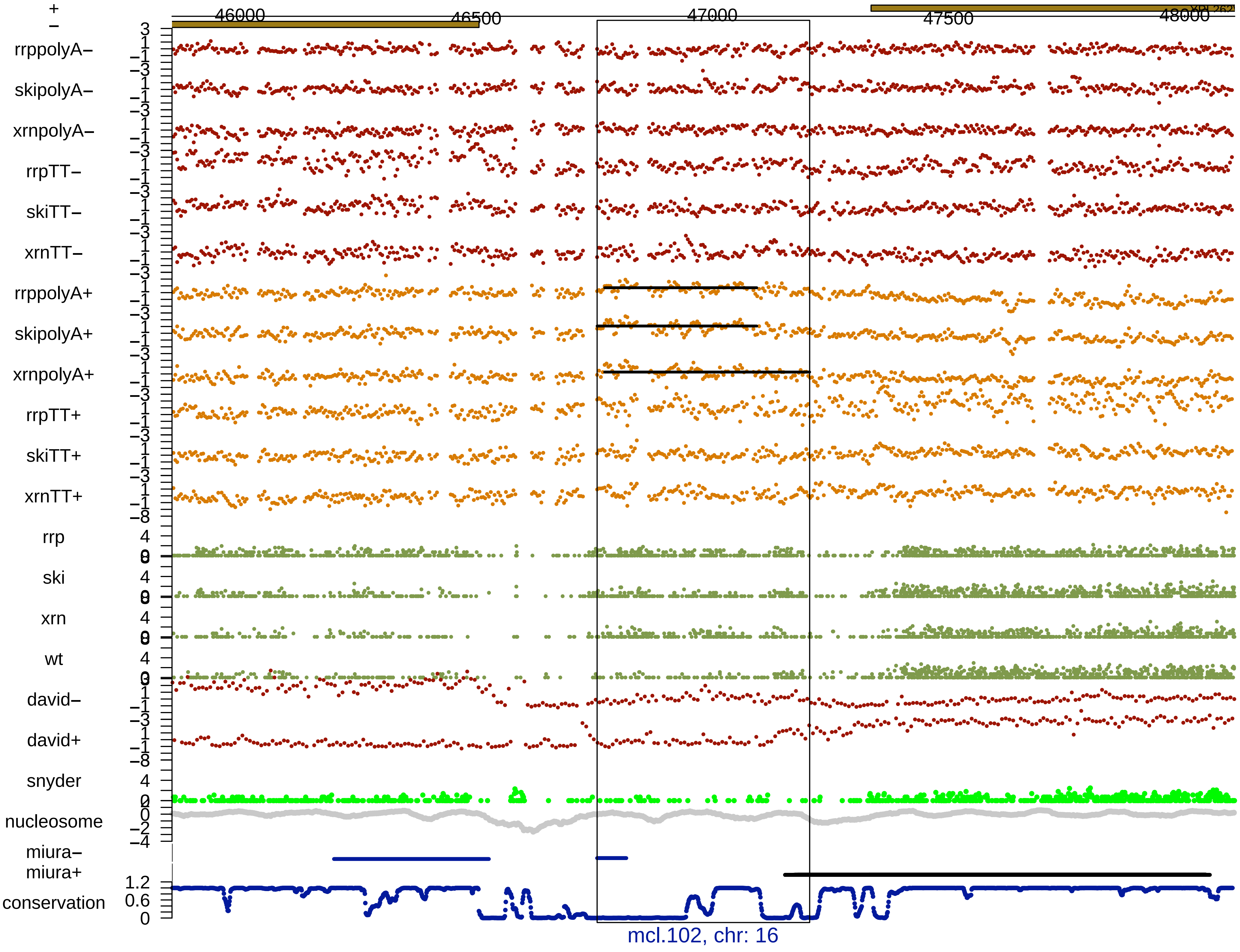

46000
46500
47000
47500
48000
rrppolyA–
skipolyA–
xrnpolyA–
rrpTT–
skiTT–
xrnTT–
rrppolyA+
skipolyA+
xrnpolyA+
rrpTT+
skiTT+
xrnTT+
rrp
ski
xrn
wt
david–
david+
snyder
nucleosome
miura–
miura+
conservation
mcl.102, chr: 16

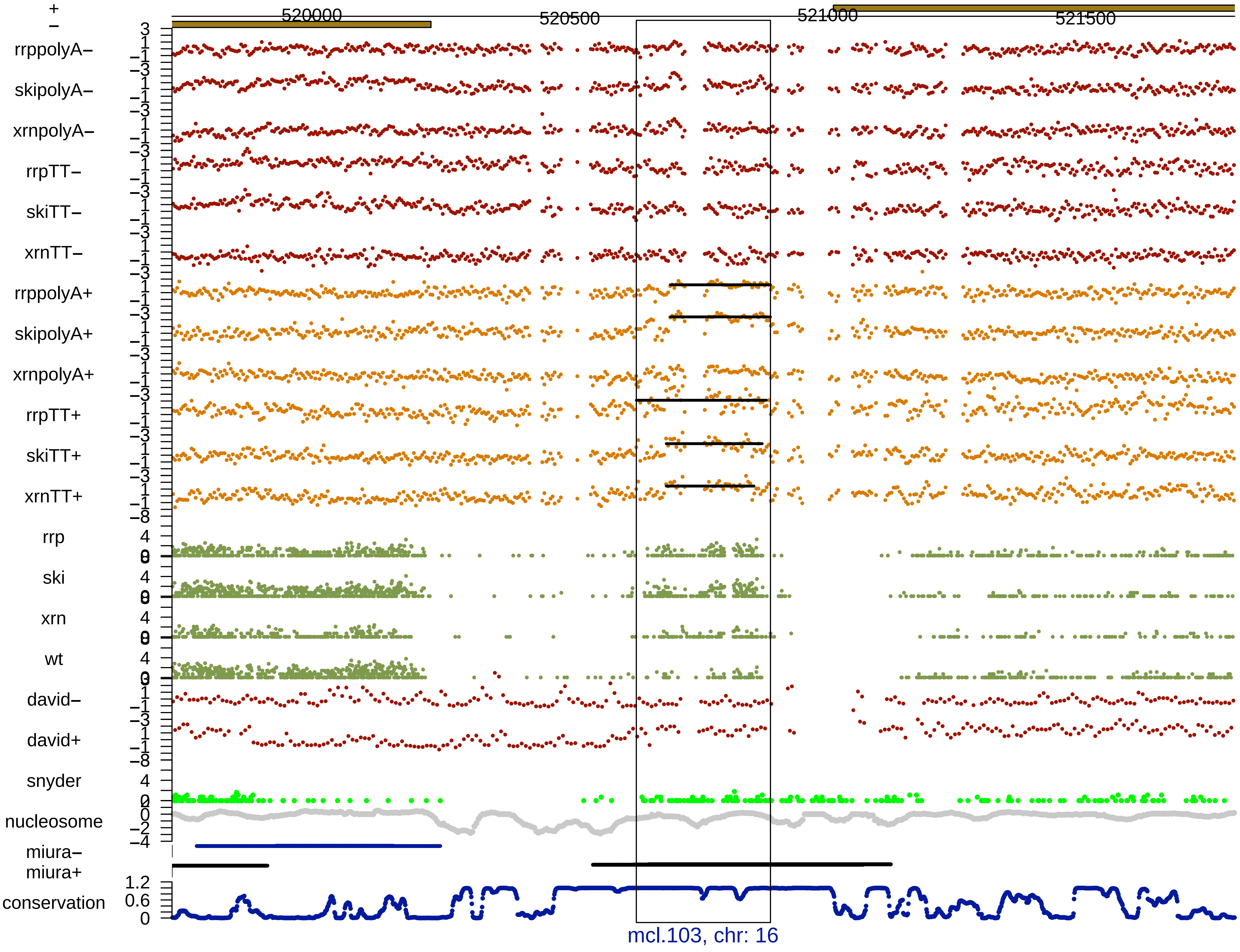

+
−
520000
520500
521000
521500
rrppolyA−
skipolyA−
xrnpolyA−
rrpTT−
skiTT−
xrnTT−
rrppolyA+
skipolyA+
xrnpolyA+
rrpTT+
skiTT+
xrnTT+
rrp
ski
xrn
wt
david−
david+
snyder
nucleosome
miura−
miura+
conservation
mcl.103, chr: 16

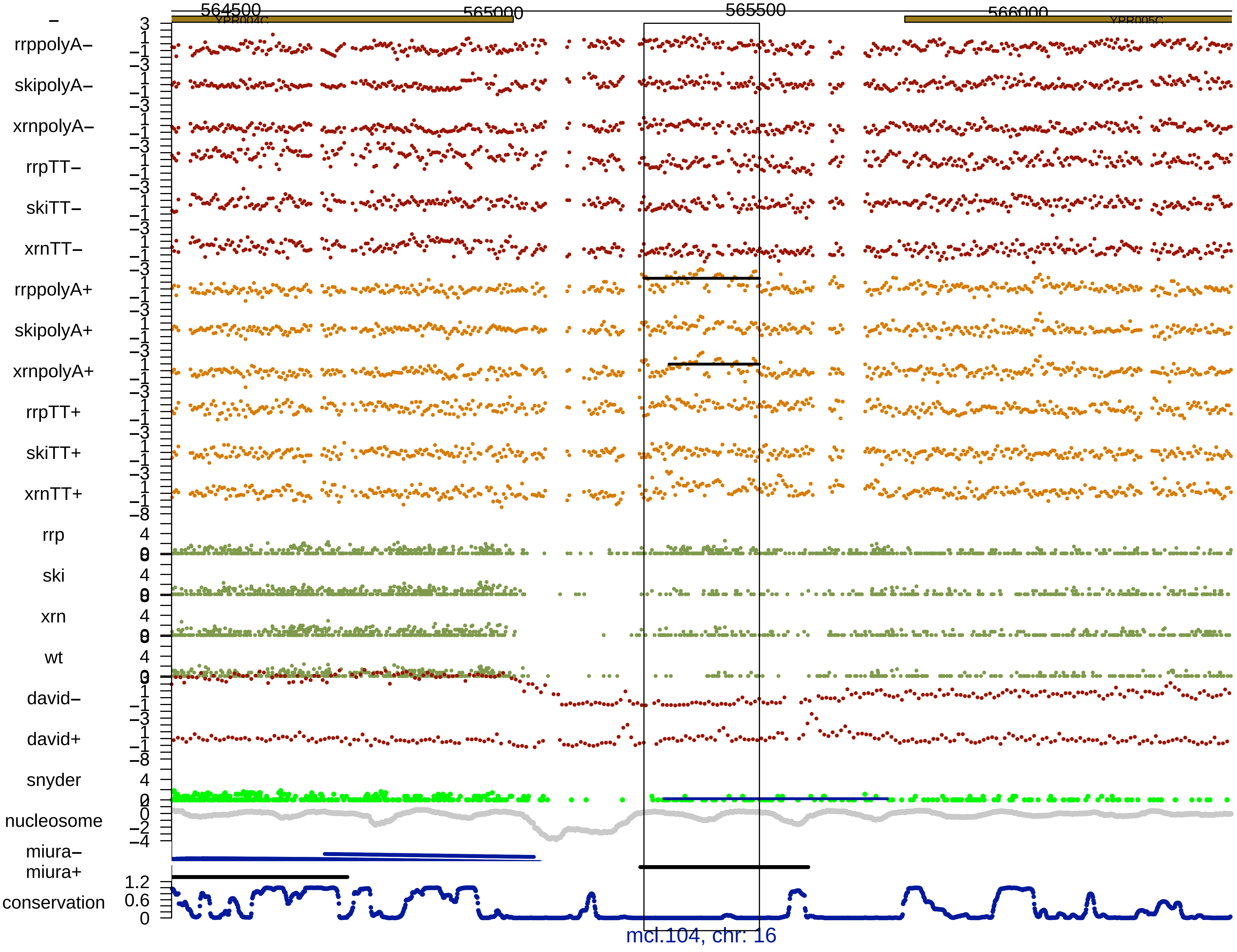

564500
565000
565500
566000
YPR004C
YPR005C
rrppolyA−
skipolyA−
xrnpolyA−
rrpTT−
skiTT−
xrnTT−
rrppolyA+
skipolyA+
xrnpolyA+
rrpTT+
skiTT+
xrnTT+
rrp
ski
xrn
wt
david−
david+
snyder
nucleosome
miura−
miura+
conservation
mcl.104, chr: 16

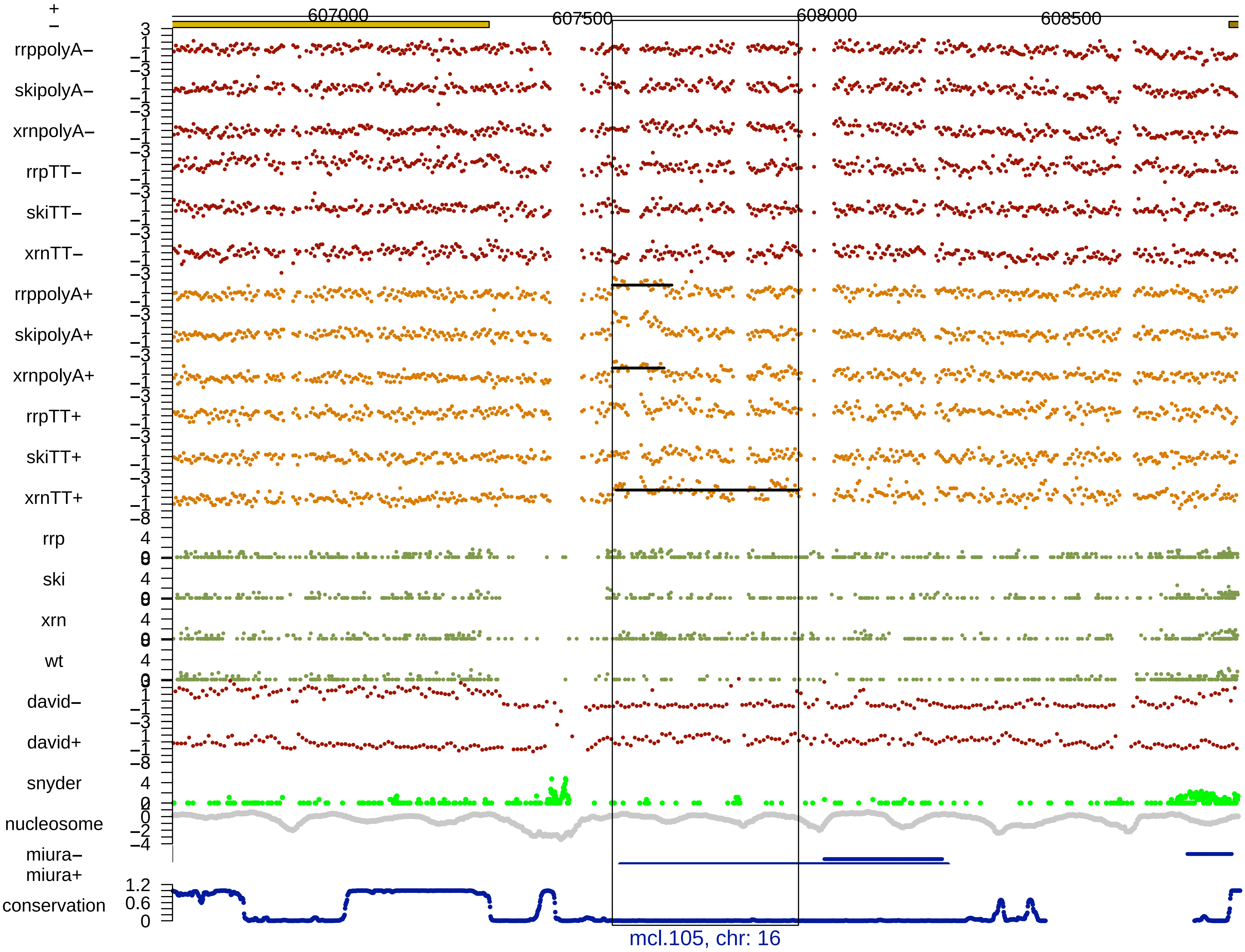

+
−
607000
607500
608000
608500
rrppolyA−
skipolyA−
xrnpolyA−
rrpTT−
skiTT−
xrnTT−
rrppolyA+
skipolyA+
xrnpolyA+
rrpTT+
skiTT+
xrnTT+
rrp
ski
xrn
wt
david−
david+
snyder
nucleosome
miura−
miura+
conservation
mcl.105, chr: 16

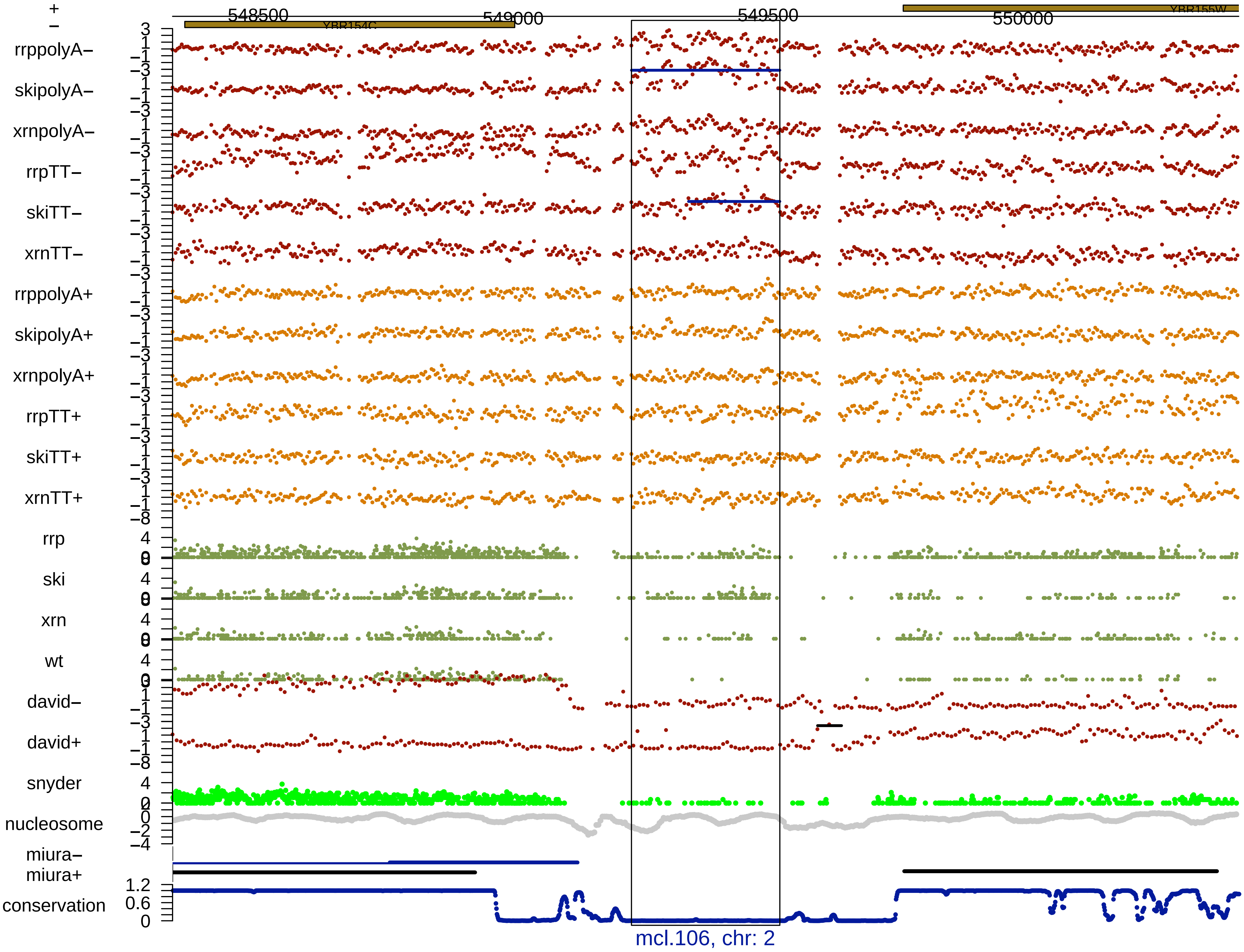

548500
549000
549500
550000
YBR154C
YBR155W
+
−
rrppolyA−
skipolyA−
xrnpolyA−
rrpTT−
skiTT−
xrnTT−
rrppolyA+
skipolyA+
xrnpolyA+
rrpTT+
skiTT+
xrnTT+
rrp
ski
xrn
wt
david−
david+
snyder
nucleosome
miura−
miura+
conservation
mcl.106, chr: 2

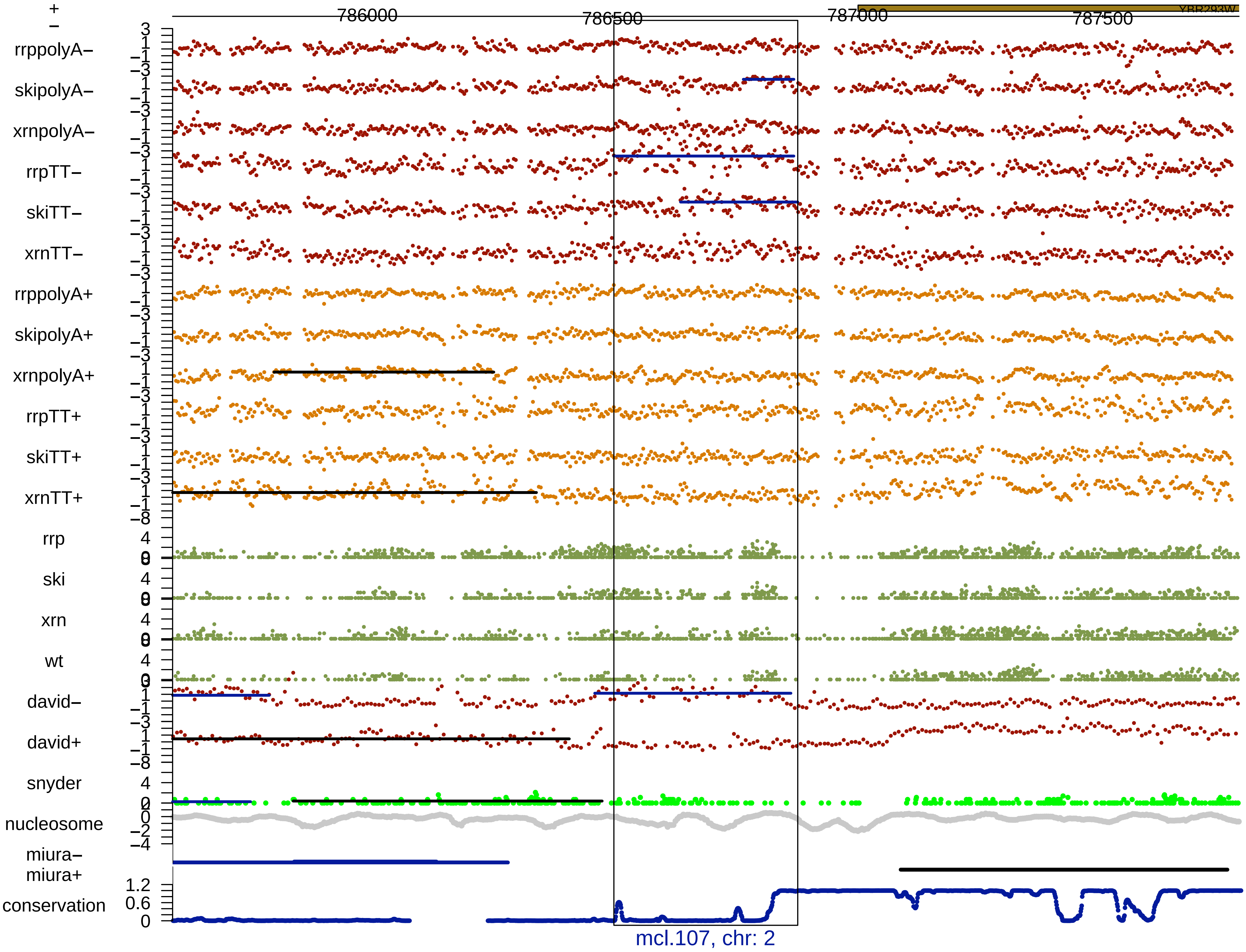

+
−
786000
786500
787000
787500
YBR293W
rrppolyA−
skipolyA−
xrnpolyA−
rrpTT−
skiTT−
xrnTT−
rrppolyA+
skipolyA+
xrnpolyA+
rrpTT+
skiTT+
xrnTT+
rrp
ski
xrn
wt
david−
david+
snyder
nucleosome
miura−
miura+
conservation
mcl.107, chr: 2

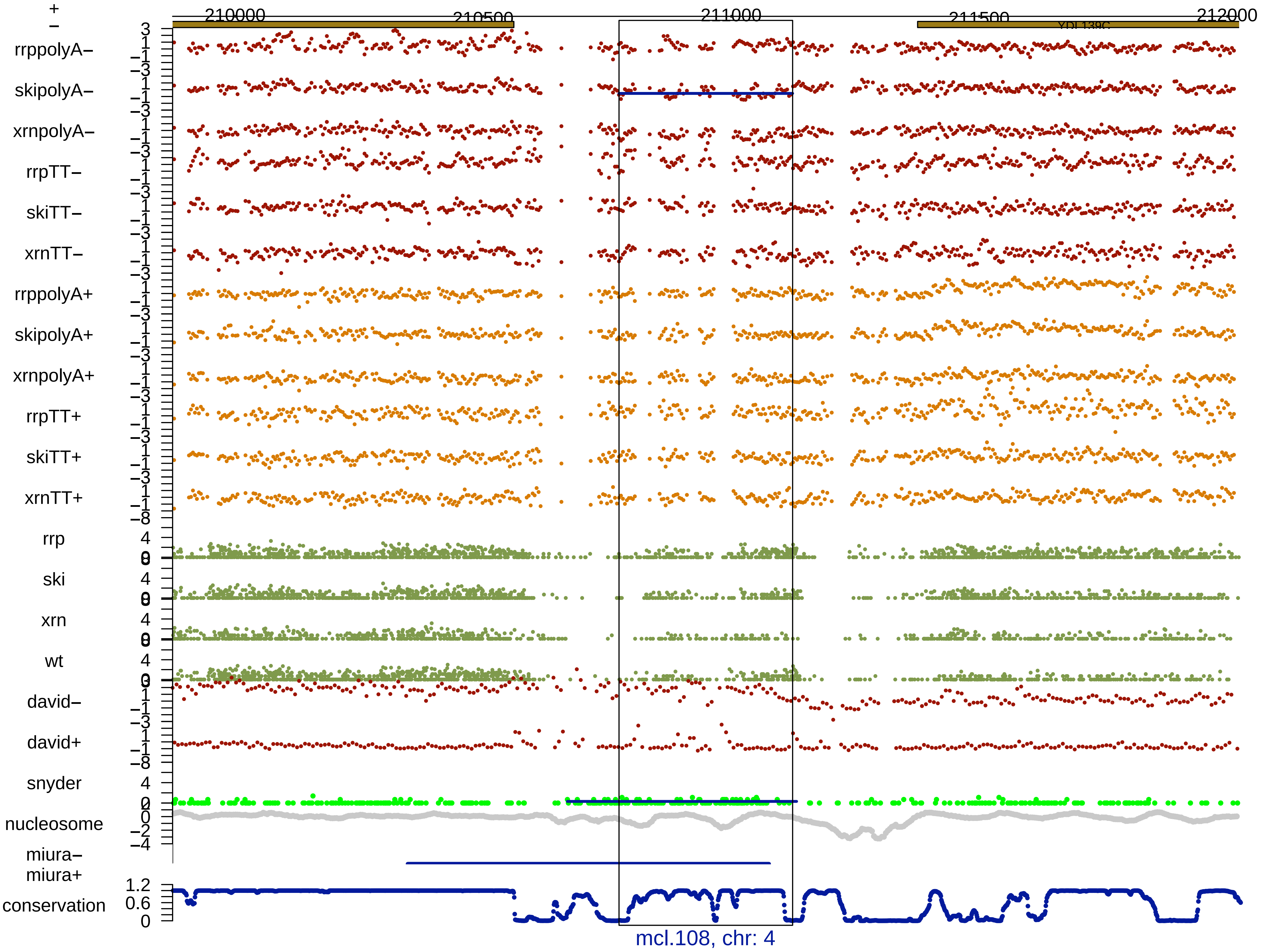
210000
210500
211000
211500
212000
YDL139C
rrppolyA−
skipolyA−
xrnpolyA−
rrpTT−
skiTT−
xrnTT−
rrppolyA+
skipolyA+
xrnpolyA+
rrpTT+
skiTT+
xrnTT+
rrp
ski
xrn
wt
david−
david+
snyder
nucleosome
miura−
miura+
conservation
mcl.108, chr: 4

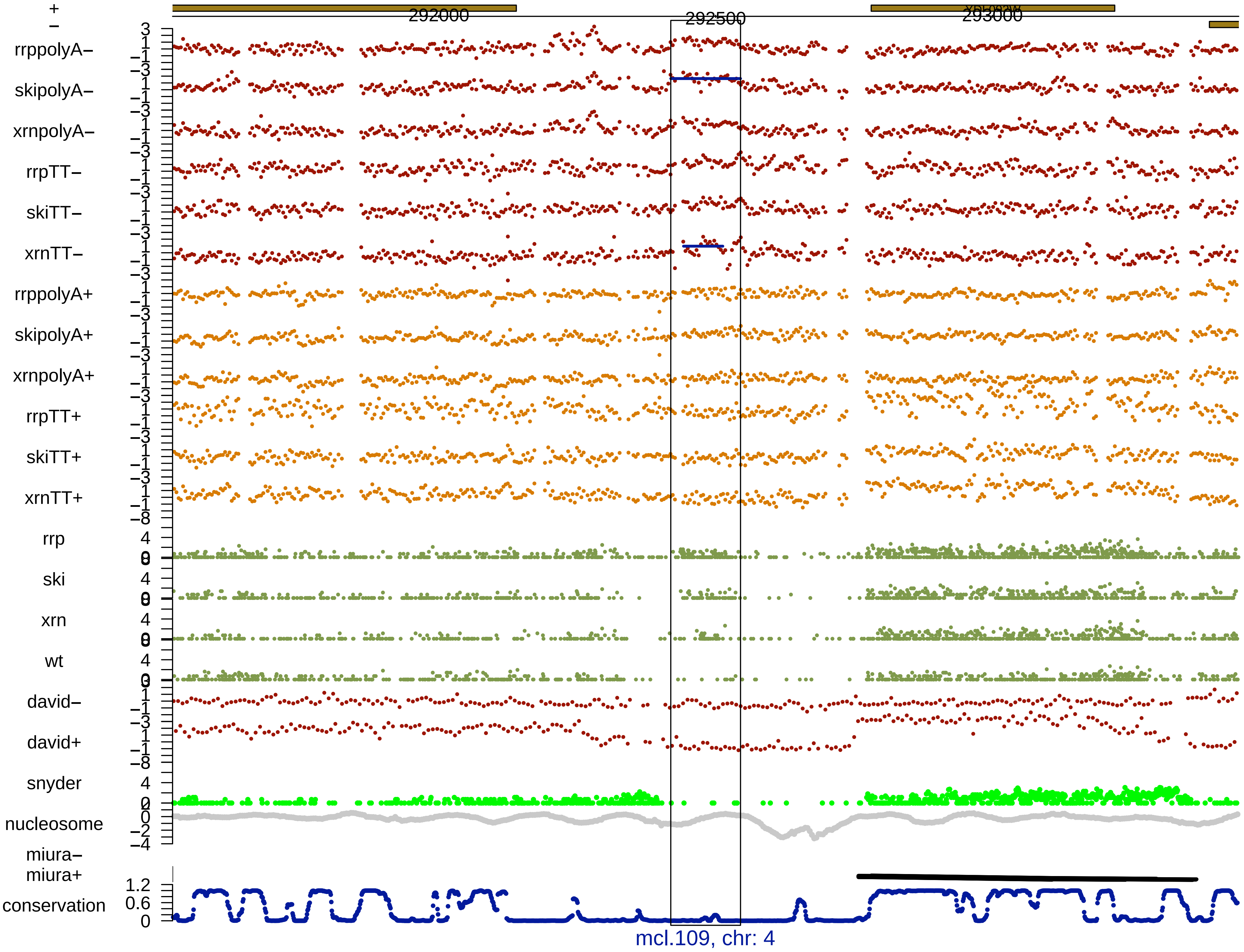

292000
292500
293000
rrppolyA−
skipolyA−
xrnpolyA−
rrpTT−
skiTT−
xrnTT−
rrppolyA+
skipolyA+
xrnpolyA+
rrpTT+
skiTT+
xrnTT+
rrp
ski
xrn
wt
david−
david+
snyder
nucleosome
miura−
miura+
conservation
mcl.109, chr: 4

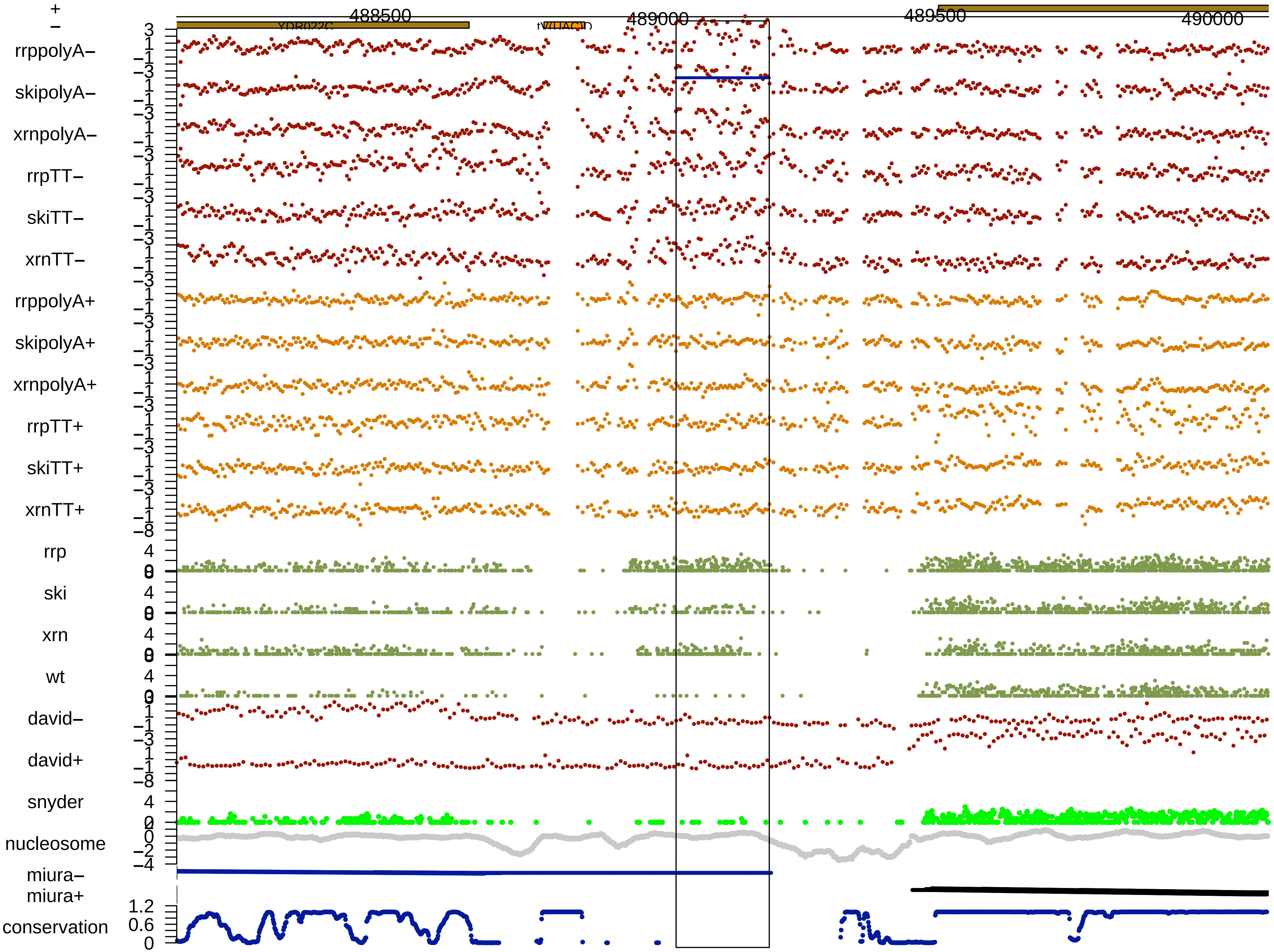
+
–
488500
489000
489500
490000
YDR022C
rrppolyA–
skipolyA–
xrnpolyA–
rrpTT–
skiTT–
xrnTT–
rrppolyA+
skipolyA+
xrnpolyA+
rrpTT+
skiTT+
xrnTT+
rrp
ski
xrn
wt
david–
david+
snyder
nucleosome
miura–
miura+
conservation
3
1
–1
–3
–8
4
0
2
–2
–4
1.2
0.6

mcl.110, chr: 4

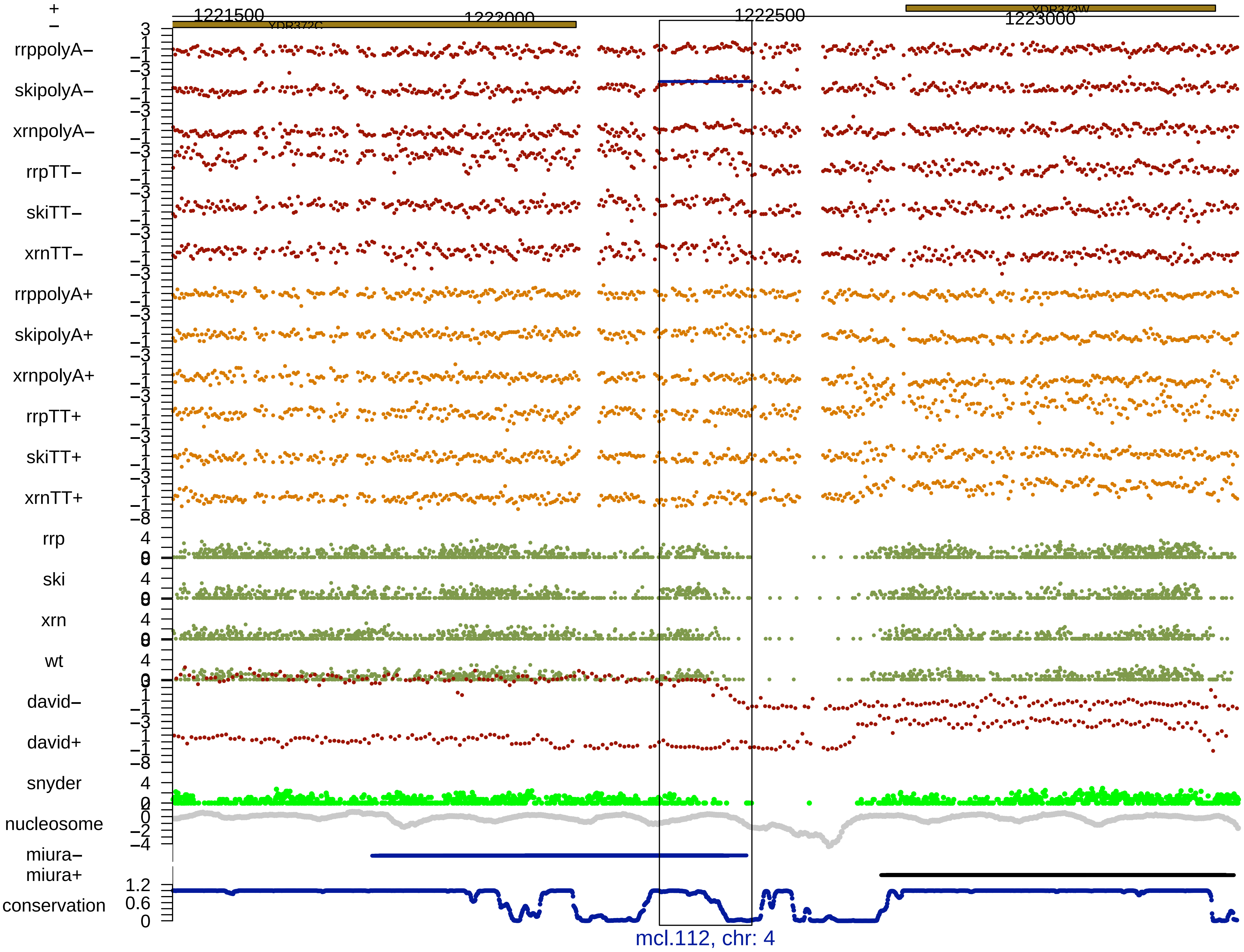

1221500
1222000
1222500
1223000
YDR372C
YDR373W
+
−
rrppolyA−
skipolyA−
xrnpolyA−
rrpTT−
skiTT−
xrnTT−
rrppolyA+
skipolyA+
xrnpolyA+
rrpTT+
skiTT+
xrnTT+
rrp
ski
xrn
wt
david−
david+
snyder
nucleosome
miura−
miura+
conservation
mcl.112, chr: 4

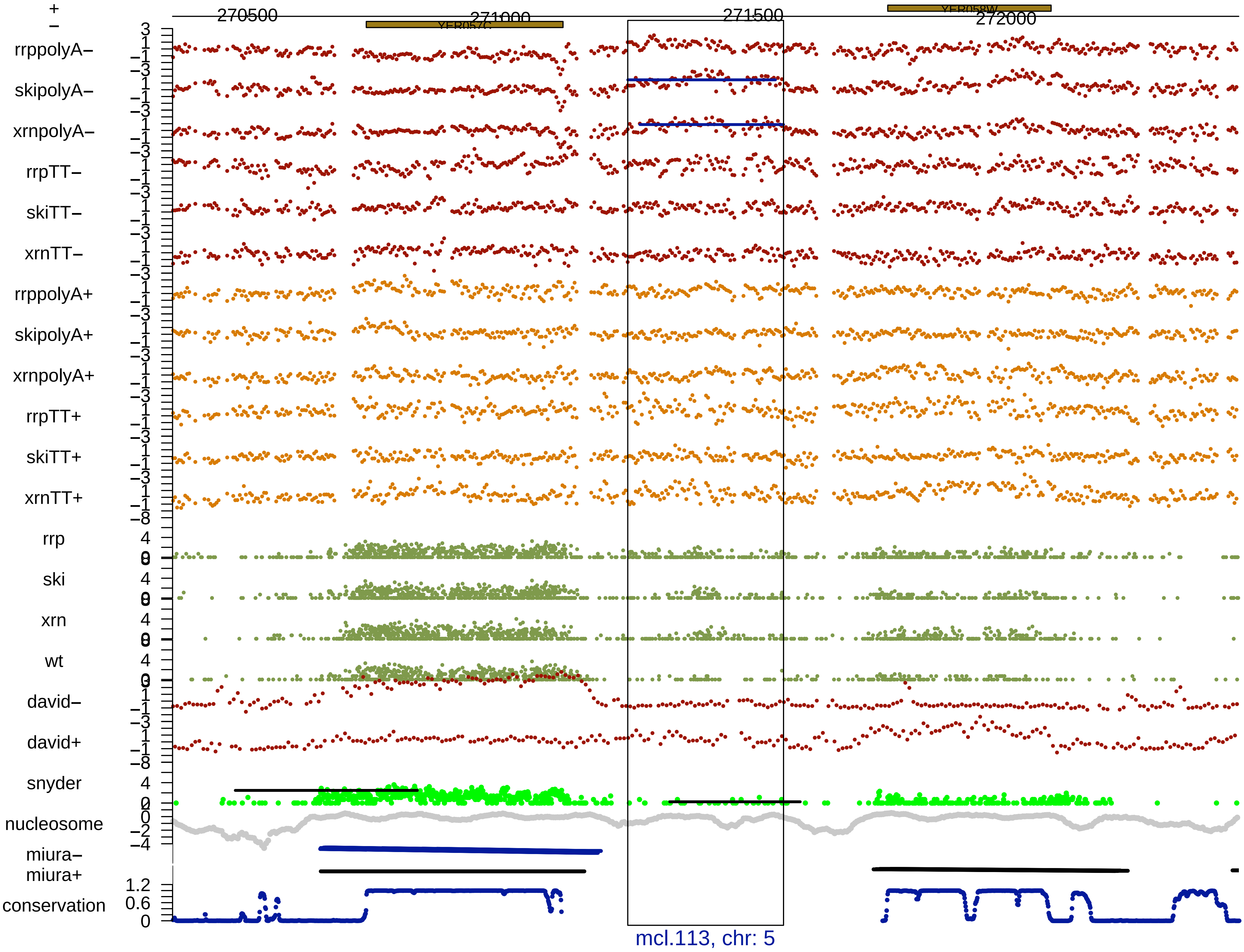

+
−
270500
271000
271500
272000
YER057C
YER058W
rrppolyA−
skipolyA−
xrnpolyA−
rrpTT−
skiTT−
xrnTT−
rrppolyA+
skipolyA+
xrnpolyA+
rrpTT+
skiTT+
xrnTT+
rrp
ski
xrn
wt
david−
david+
snyder
nucleosome
miura−
miura+
conservation
mcl.113, chr: 5

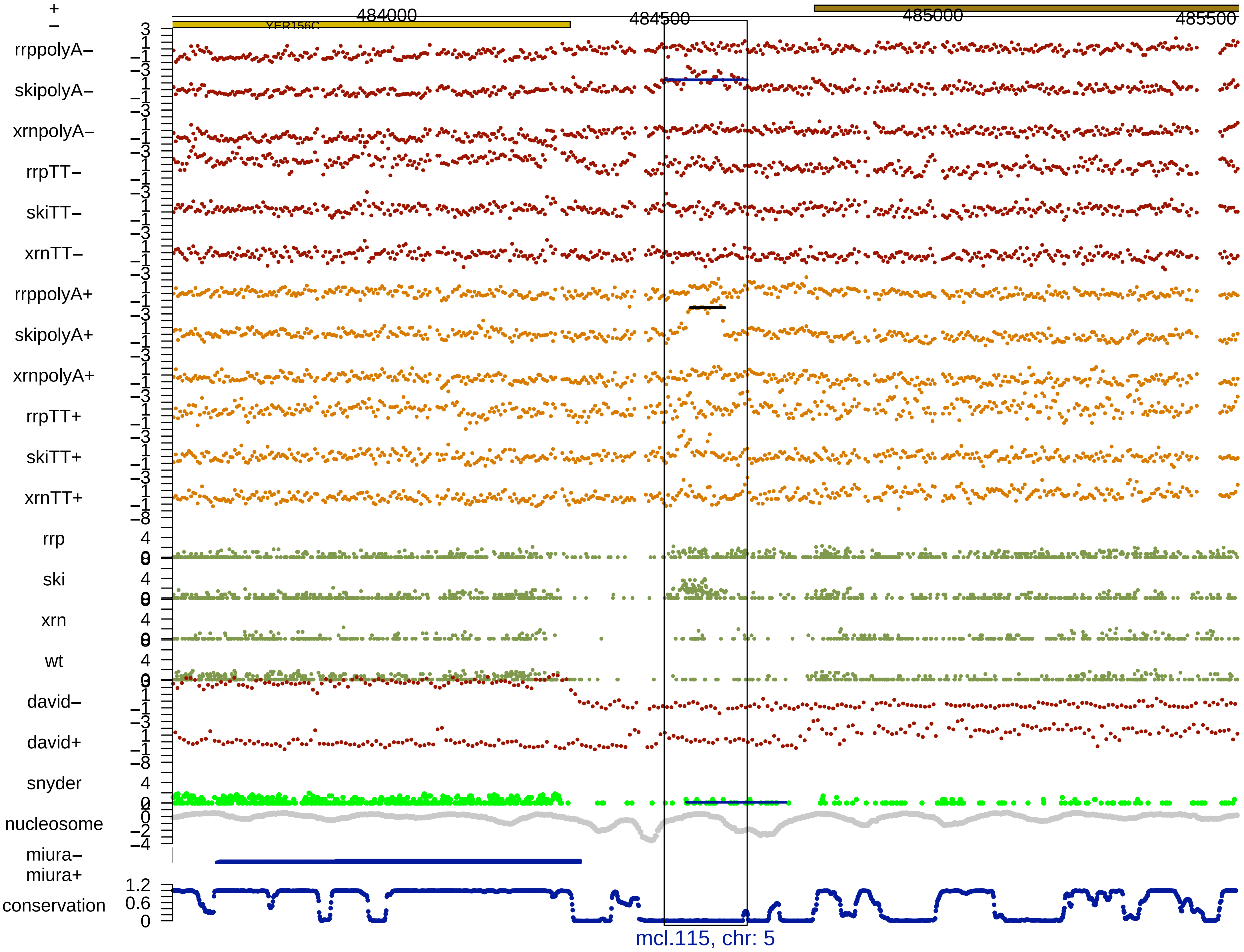

+
−
YER156C
484000
484500
485000
485500
rrppolyA−
skipolyA−
xrnpolyA−
rrpTT−
skiTT−
xrnTT−
rrppolyA+
skipolyA+
xrnpolyA+
rrpTT+
skiTT+
xrnTT+
rrp
ski
xrn
wt
david−
david+
snyder
nucleosome
miura−
miura+
conservation
mcl.115, chr: 5

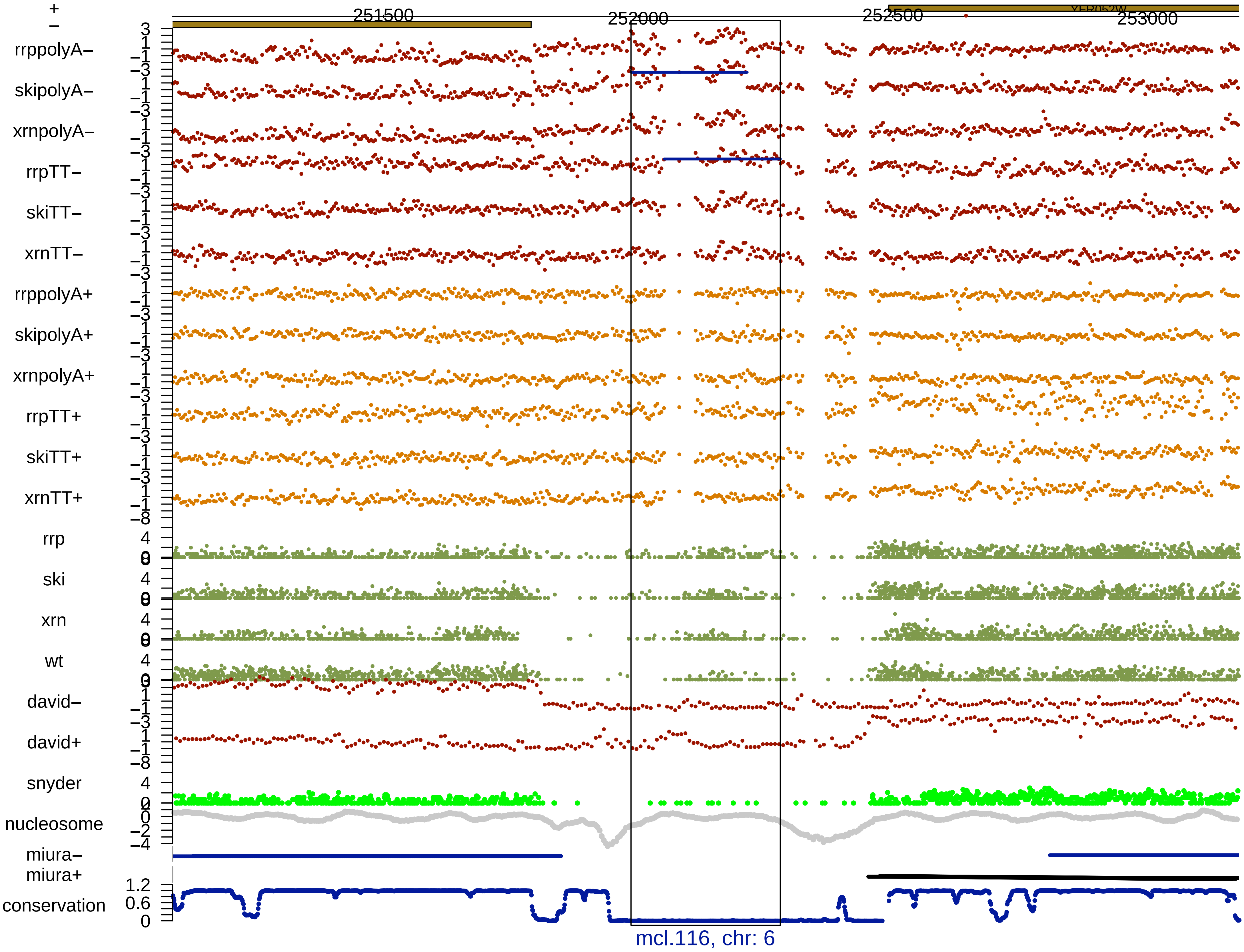

+
−
251500
252000
252500
253000
YER052W
rrppolyA−
skipolyA−
xrnpolyA−
rrpTT−
skiTT−
xrnTT−
rrppolyA+
skipolyA+
xrnpolyA+
rrpTT+
skiTT+
xrnTT+
rrp
ski
xrn
wt
david−
david+
snyder
nucleosome
miura−
miura+
conservation
mcl.116, chr: 6

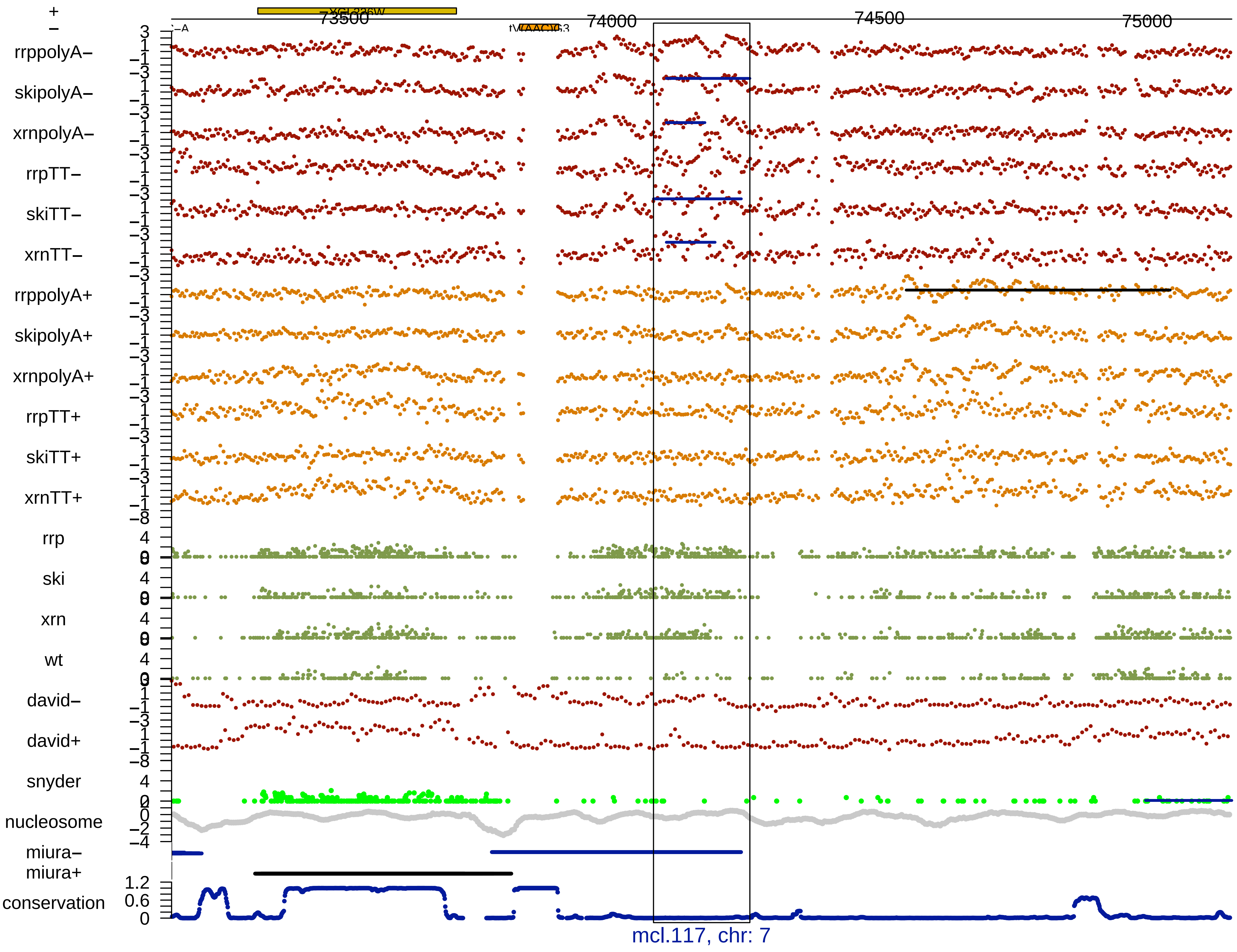

73500
74000
74500
75000
rrppolyA−
skipolyA−
xrnpolyA−
rrpTT−
skiTT−
xrnTT−
rrppolyA+
skipolyA+
xrnpolyA+
rrpTT+
skiTT+
xrnTT+
rrp
ski
xrn
wt
david−
david+
snyder
nucleosome
miura−
miura+
conservation
mcl.117, chr: 7

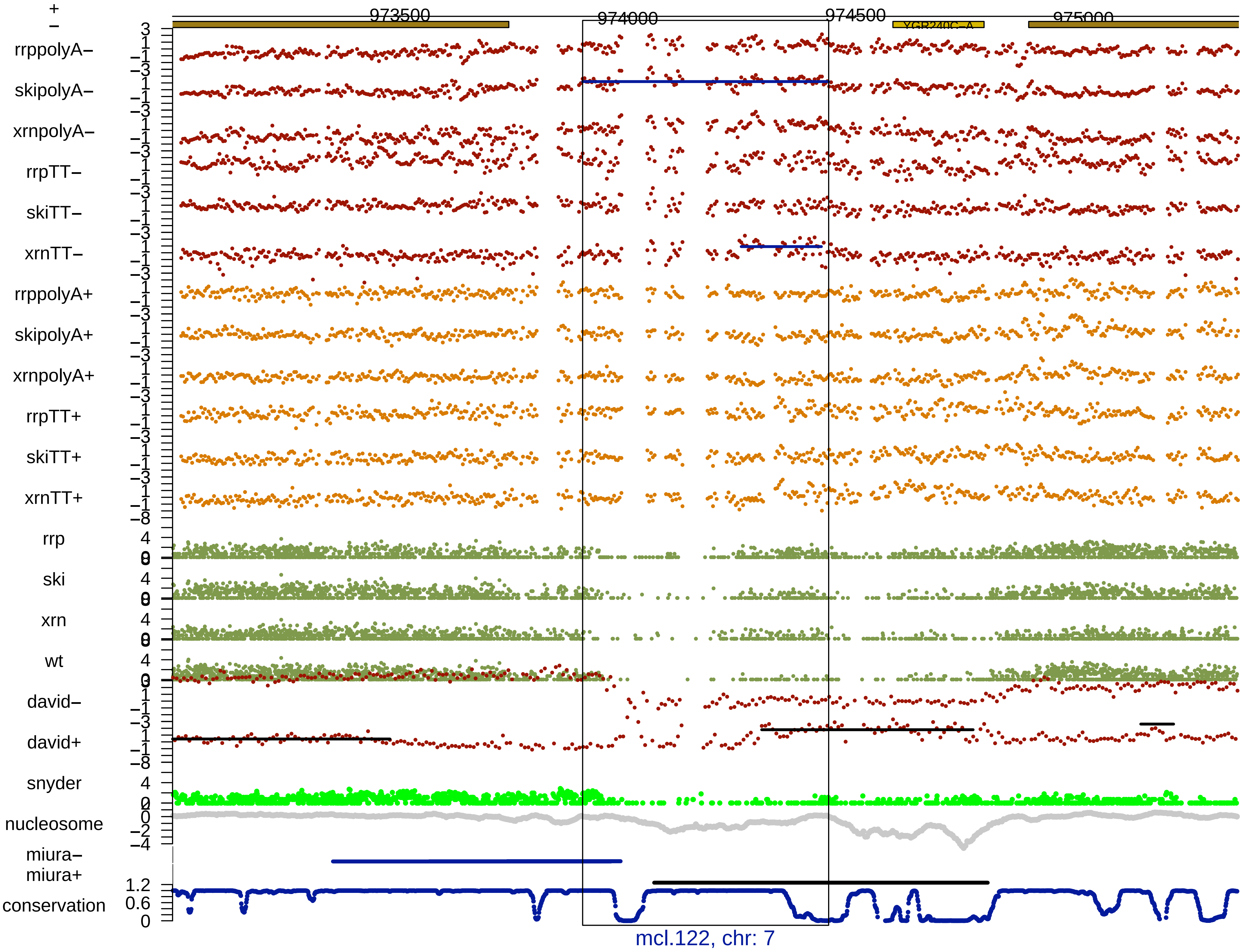

973500
974000
974500
975000
YGR240C-A
rrppolyA−
skipolyA−
xrnpolyA−
rrpTT−
skiTT−
xrnTT−
rrppolyA+
skipolyA+
xrnpolyA+
rrpTT+
skiTT+
xrnTT+
rrp
ski
xrn
wt
david−
david+
snyder
nucleosome
miura−
miura+
conservation
mcl.122, chr: 7

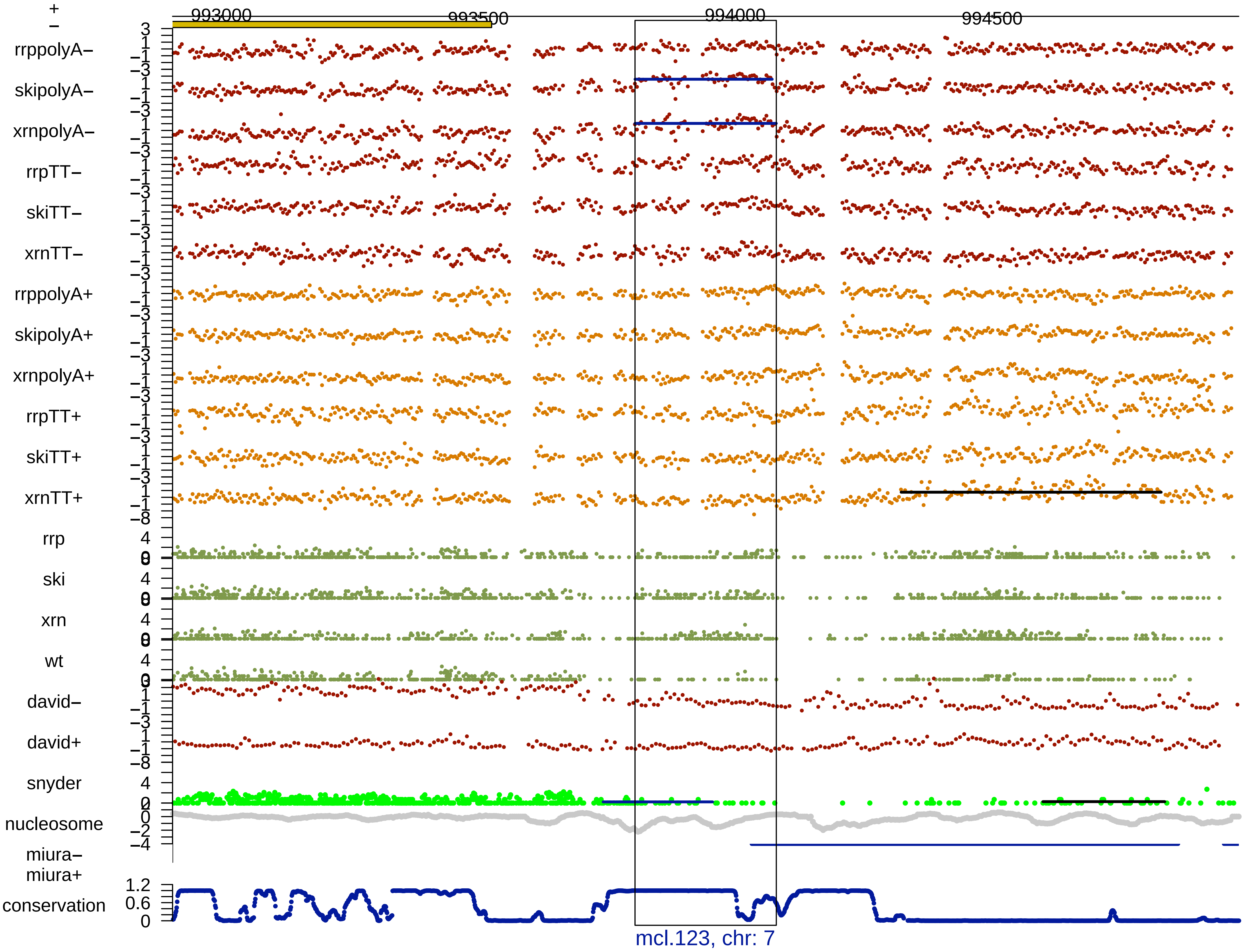

993000
993500
994000
994500
rrppolyA−
skipolyA−
xrnpolyA−
rrpTT−
skiTT−
xrnTT−
rrppolyA+
skipolyA+
xrnpolyA+
rrpTT+
skiTT+
xrnTT+
rrp
ski
xrn
wt
david−
david+
snyder
nucleosome
miura−
miura+
conservation
mcl.123, chr: 7

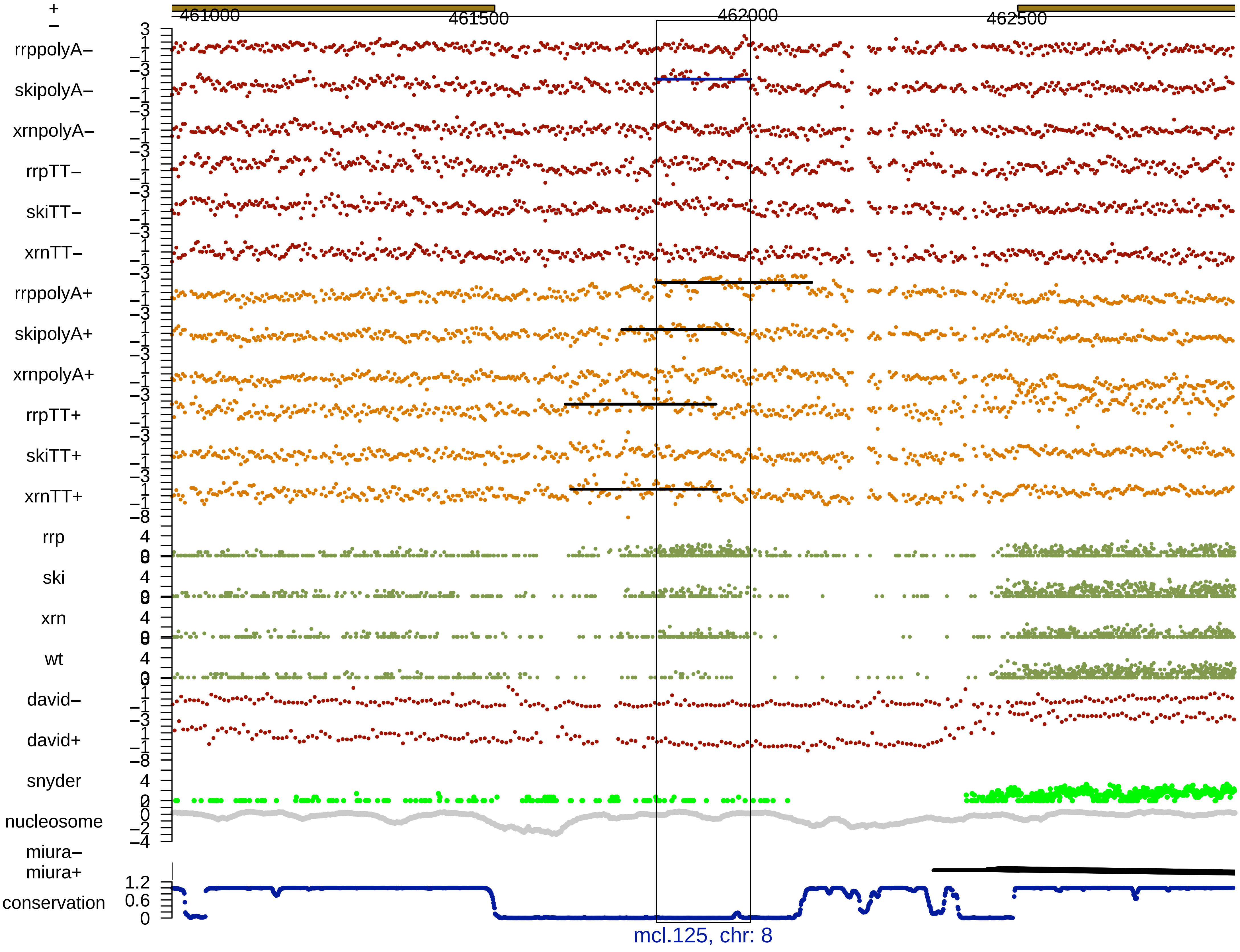
+
−
461000
461500
462000
462500
rrppolyA−
skipolyA−
xrnpolyA−
rrpTT−
skiTT−
xrnTT−
rrppolyA+
skipolyA+
xrnpolyA+
rrpTT+
skiTT+
xrnTT+
rrp
ski
xrn
wt
david−
david+
snyder
nucleosome
miura−
miura+
conservation
mcl.125, chr: 8

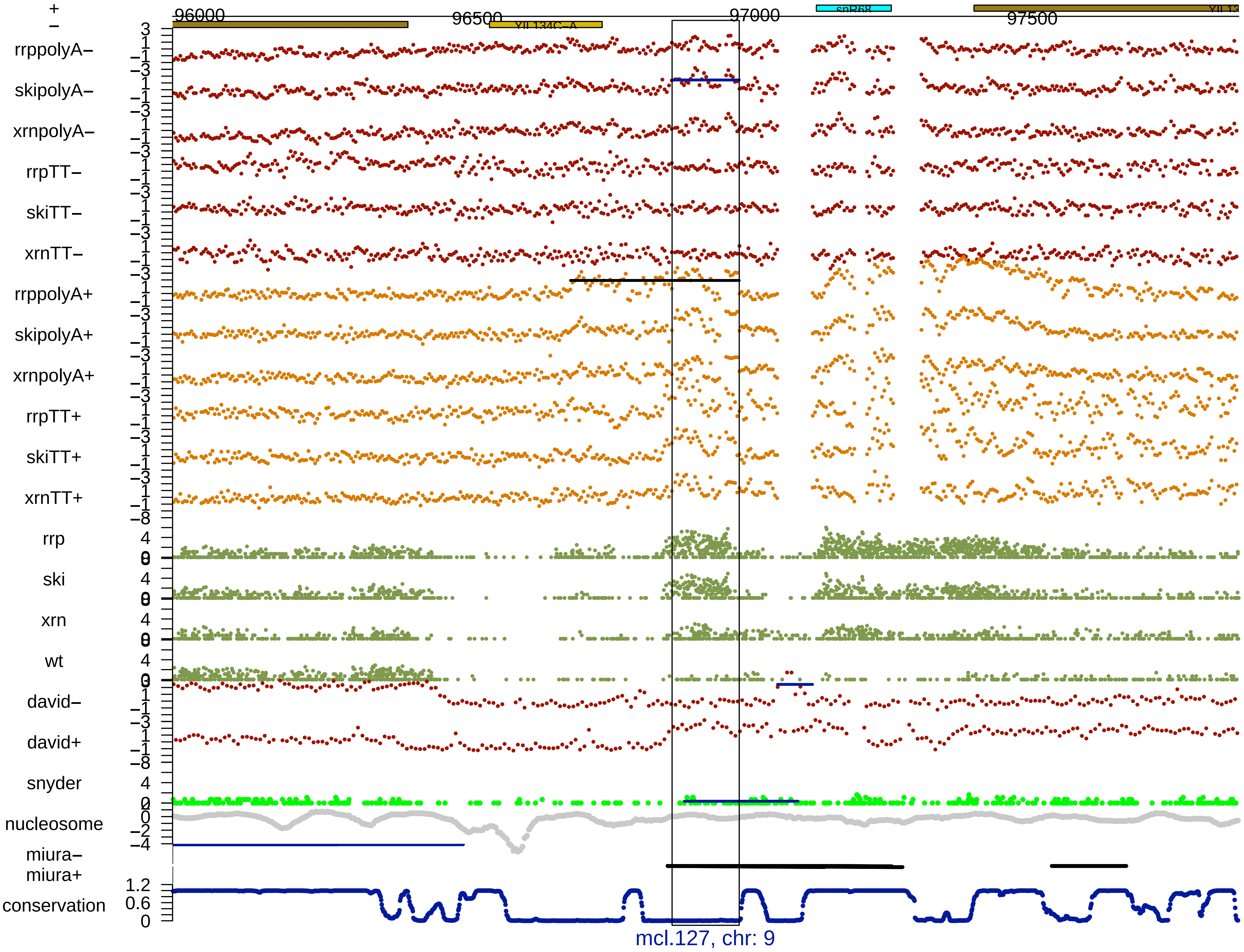

+
−
96000
96500
97000
97500
rrppolyA−
skipolyA−
xrnpolyA−
rrpTT−
skiTT−
xrnTT−
rrppolyA+
skipolyA+
xrnpolyA+
rrpTT+
skiTT+
xrnTT+
rrp
ski
xrn
wt
david−
david+
snyder
nucleosome
miura−
miura+
conservation
mcl.127, chr: 9

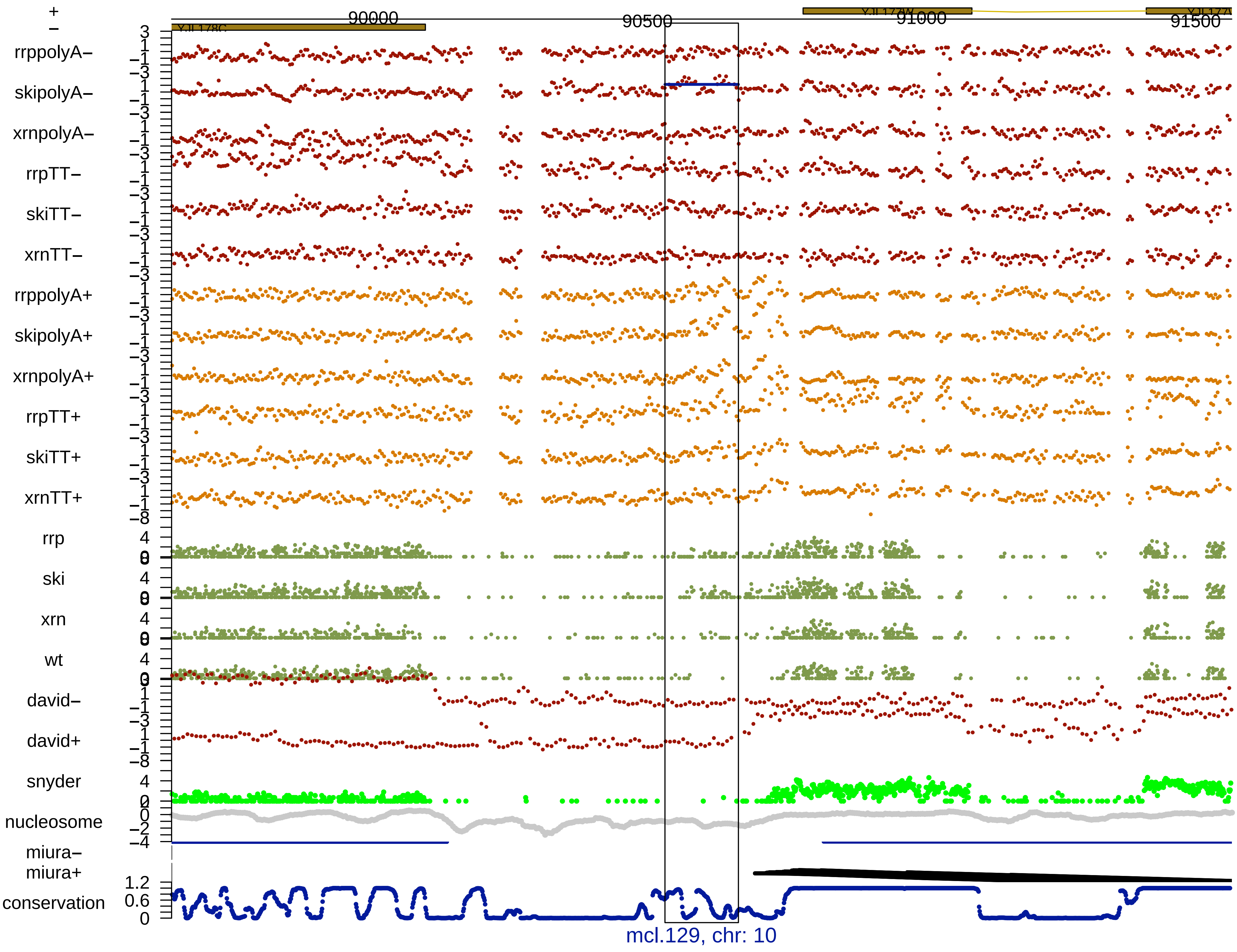
+
–
90000
90500
91000
91500
rrppolyA–
skipolyA–
xrnpolyA–
rrpTT–
skiTT–
xrnTT–
rrppolyA+
skipolyA+
xrnpolyA+
rrpTT+
skiTT+
xrnTT+
rrp
ski
xrn
wt
david–
david+
snyder
nucleosome
miura–
miura+
conservation
mcl.129, chr: 10

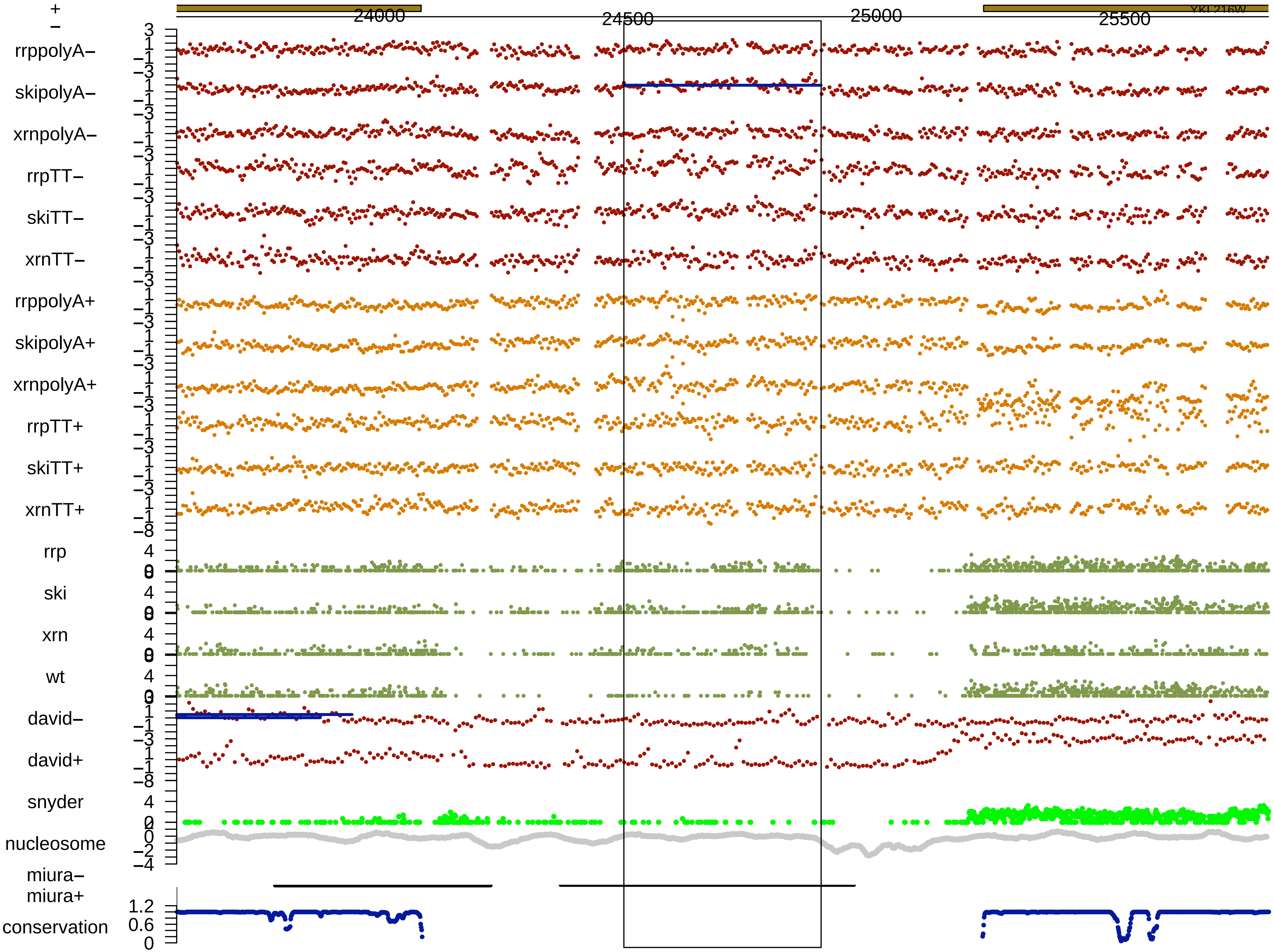

mcl.131, chr: 11

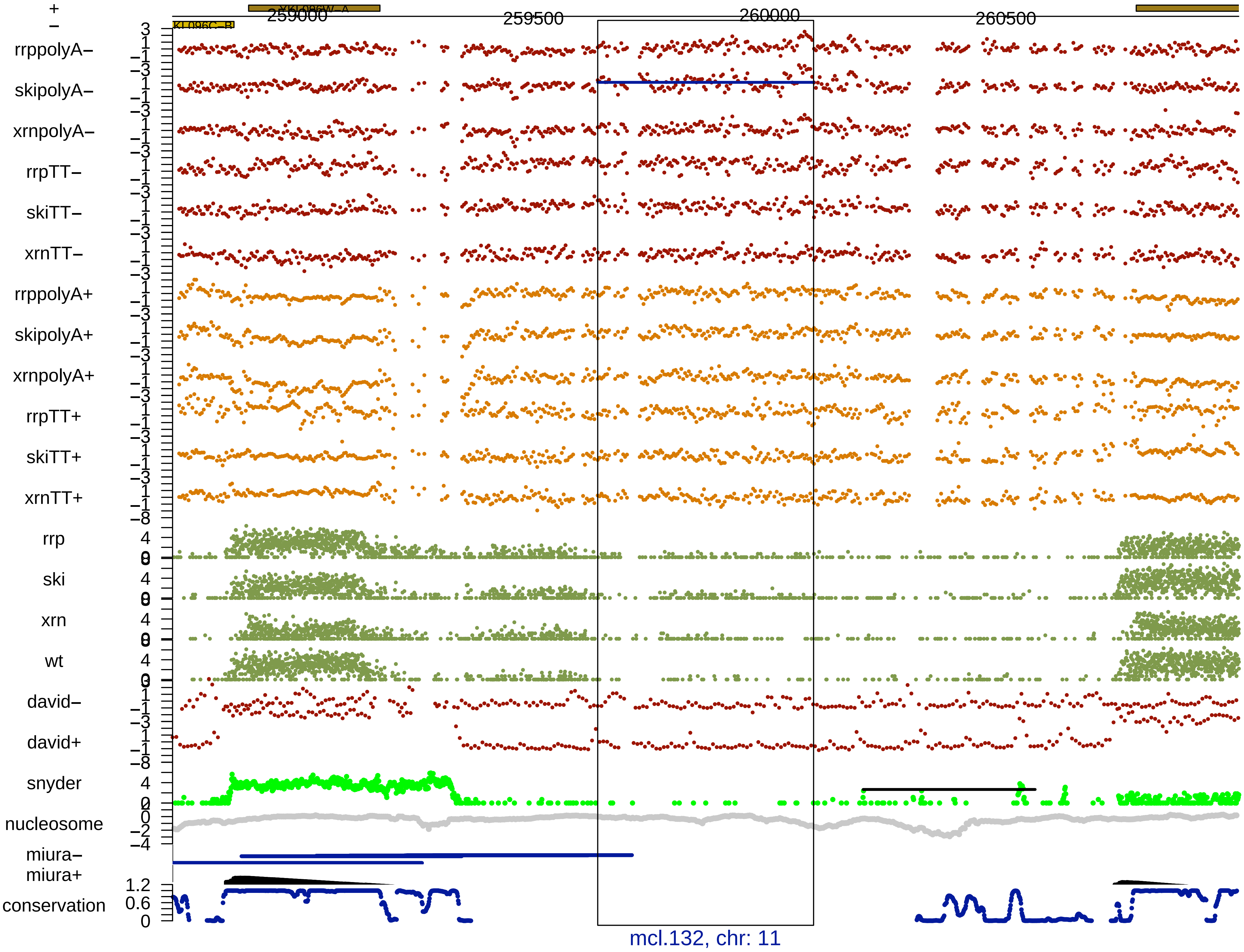

259000
259500
260000
260500
rrppolyA−
skipolyA−
xrnpolyA−
rrpTT−
skiTT−
xrnTT−
rrppolyA+
skipolyA+
xrnpolyA+
rrpTT+
skiTT+
xrnTT+
rrp
ski
xrn
wt
david−
david+
snyder
nucleosome
miura−
miura+
conservation
mcl.132, chr: 11

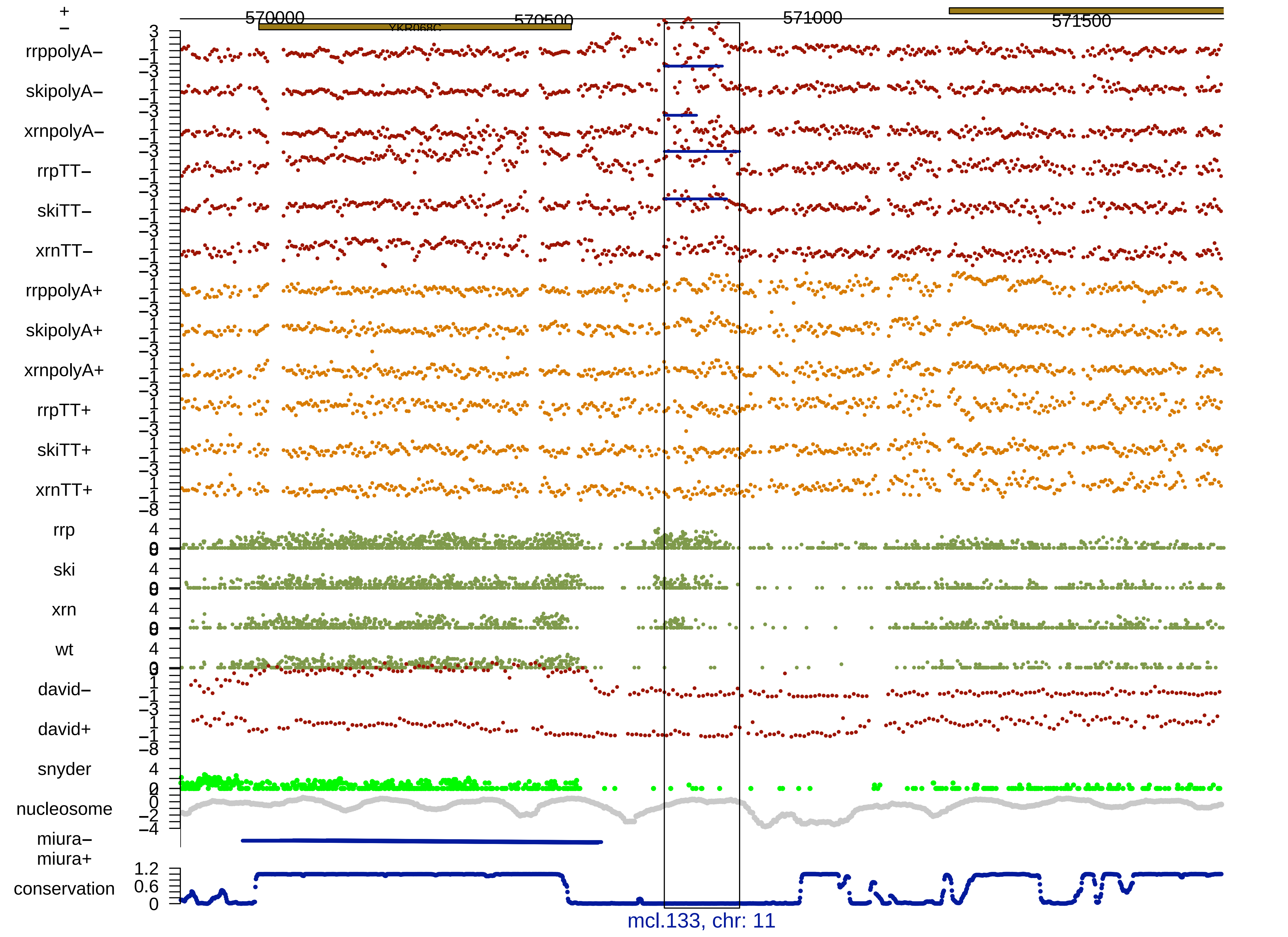

570000
570500
571000
571500
YKR068C
rrppolyA−
skipolyA−
xrnpolyA−
rrpTT−
skiTT−
xrnTT−
rrppolyA+
skipolyA+
xrnpolyA+
rrpTT+
skiTT+
xrnTT+
rrp
ski
xrn
wt
david−
david+
snyder
nucleosome
miura−
miura+
conservation
mcl.133, chr: 11

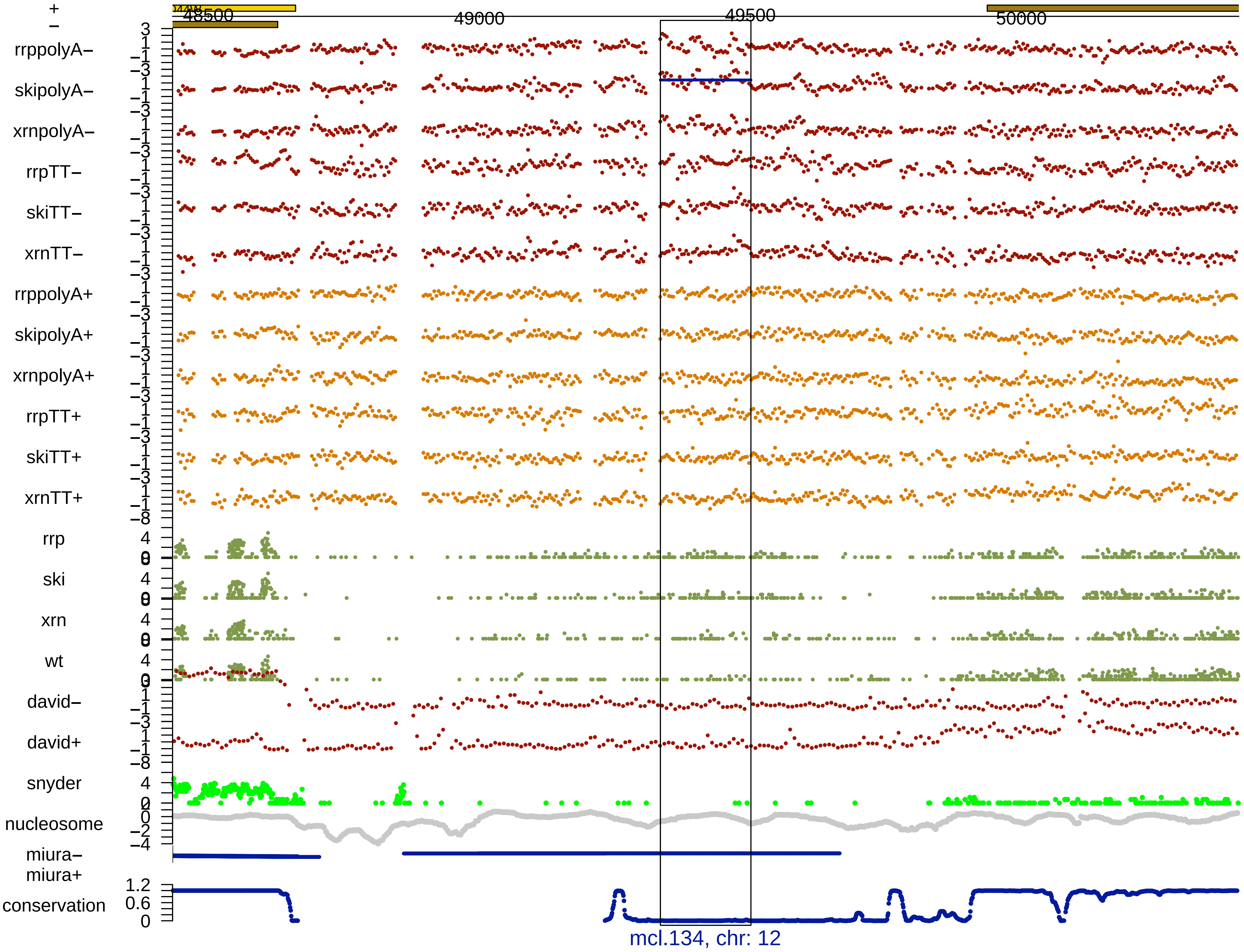

+
–
48500
49000
49500
50000
rrppolyA–
skipolyA–
xrnpolyA–
rrpTT–
skiTT–
xrnTT–
rrppolyA+
skipolyA+
xrnpolyA+
rrpTT+
skiTT+
xrnTT+
rrp
ski
xrn
wt
david–
david+
snyder
nucleosome
miura–
miura+
conservation
mcl.134, chr: 12

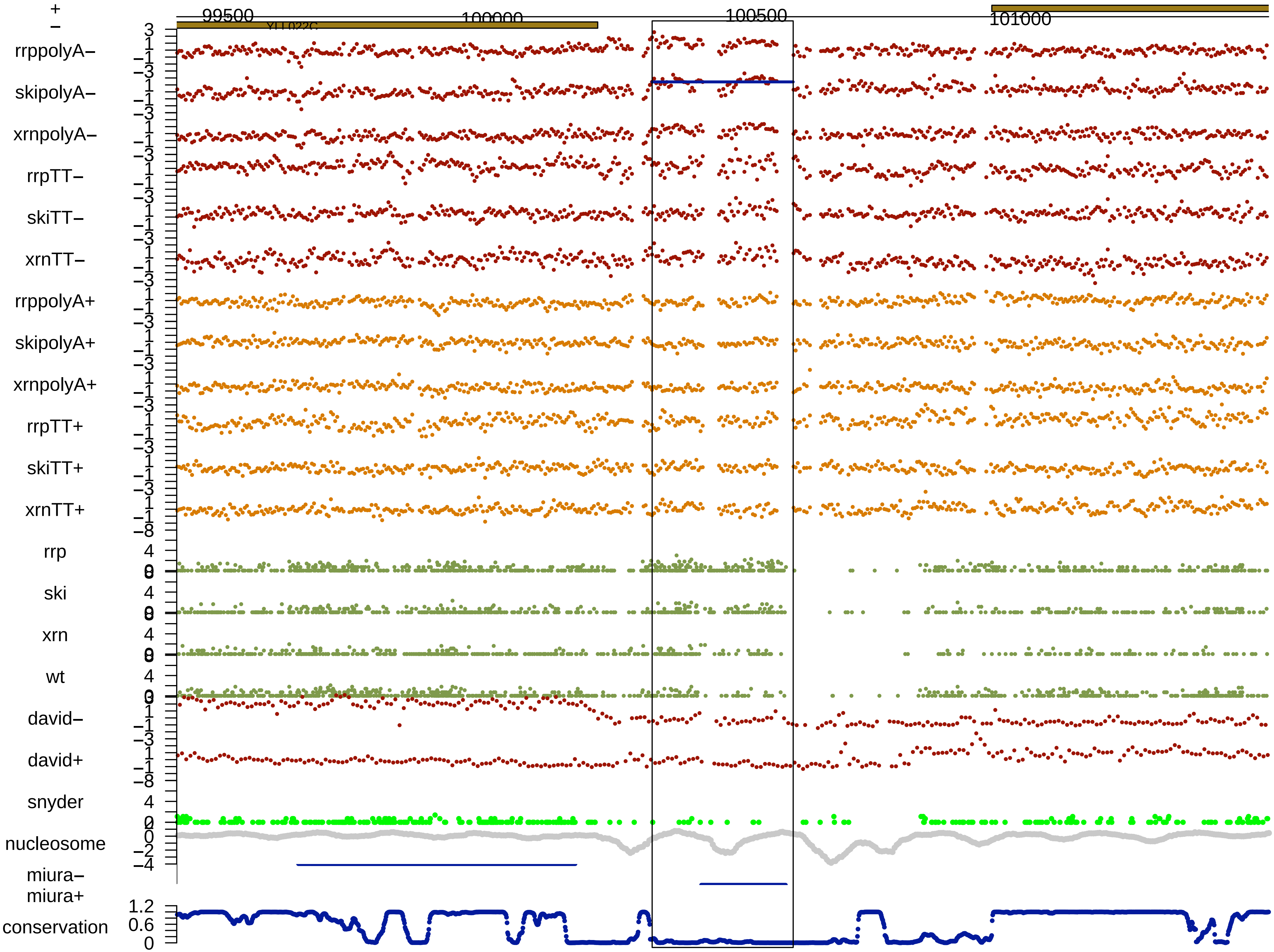

mcl.135, chr: 12

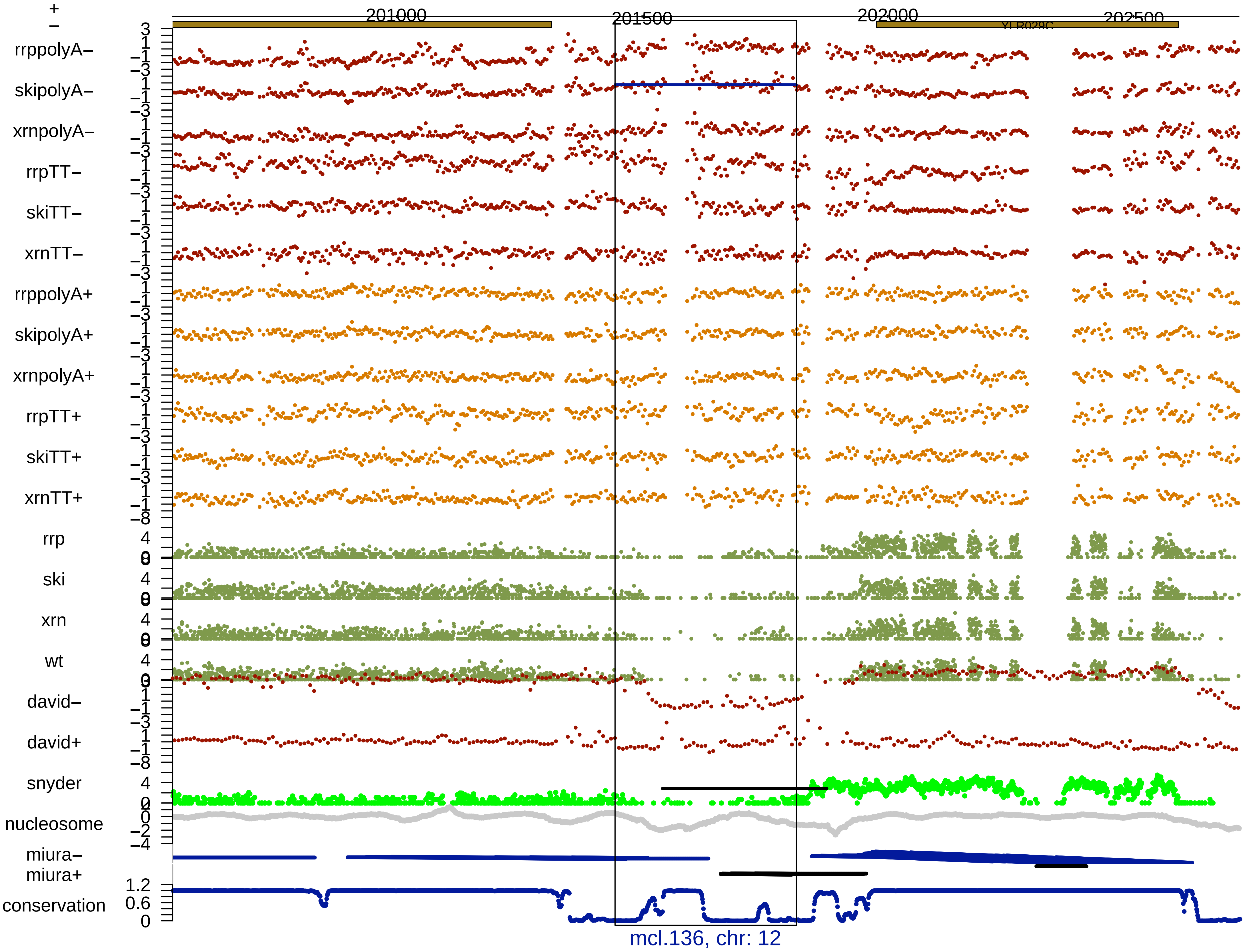

+
−
201000
201500
202000
202500
rrppolyA−
skipolyA−
xrnpolyA−
rrpTT−
skiTT−
xrnTT−
rrppolyA+
skipolyA+
xrnpolyA+
rrpTT+
skiTT+
xrnTT+
rrp
ski
xrn
wt
david−
david+
snyder
nucleosome
miura−
miura+
conservation
mcl.136, chr: 12

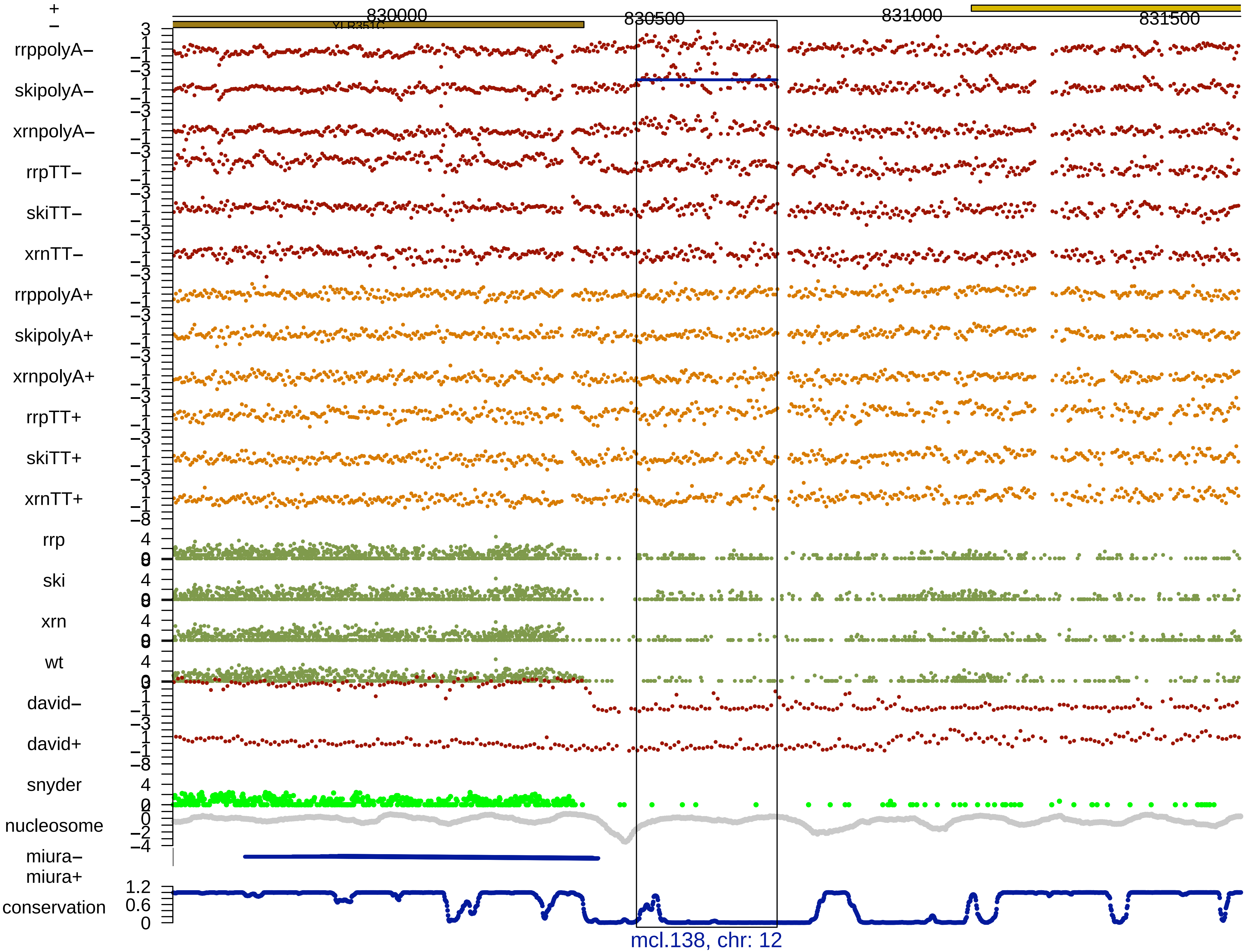

830000
830500
831000
831500
YLR351C
rrppolyA–
skipolyA–
xrnpolyA–
rrpTT–
skiTT–
xrnTT–
rrppolyA+
skipolyA+
xrnpolyA+
rrpTT+
skiTT+
xrnTT+
rrp
ski
xrn
wt
david–
david+
snyder
nucleosome
miura–
miura+
conservation
mcl.138, chr: 12

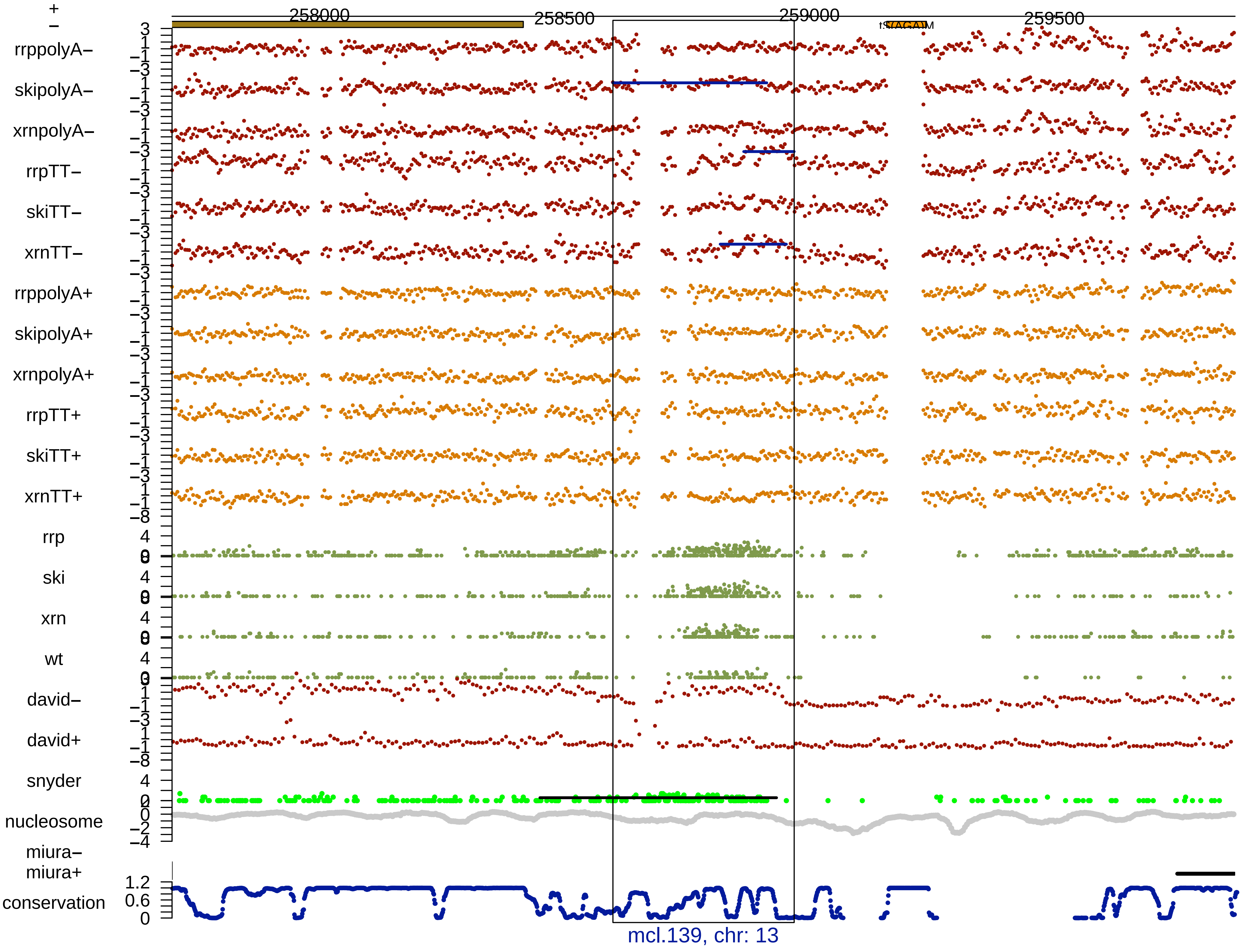

258000
258500
259000
259500
tS(AGA)M
rrppolyA−
skipolyA−
xrnpolyA−
rrpTT−
skiTT−
xrnTT−
rrppolyA+
skipolyA+
xrnpolyA+
rrpTT+
skiTT+
xrnTT+
rrp
ski
xrn
wt
david−
david+
snyder
nucleosome
miura−
miura+
conservation
mcl.139, chr: 13

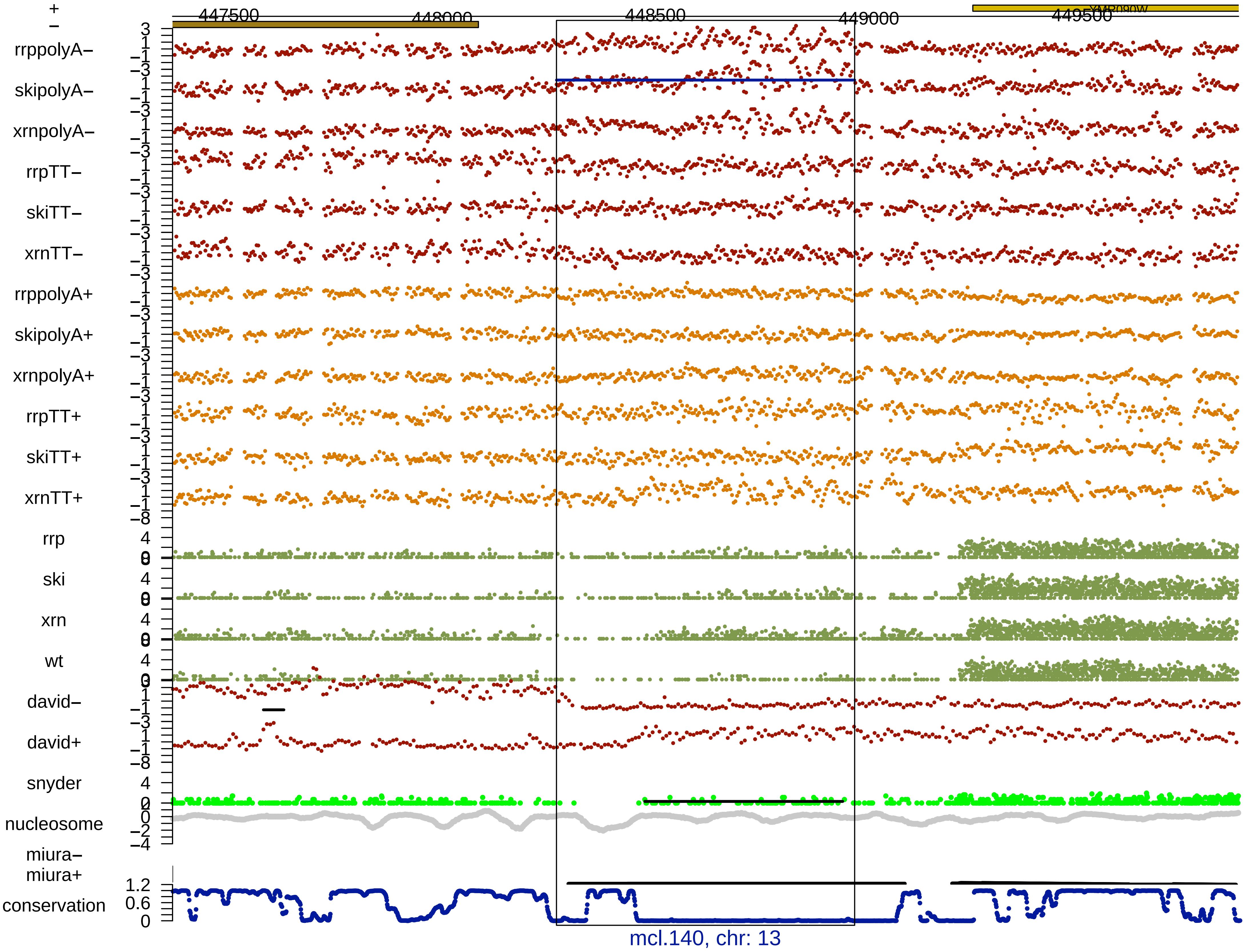
+
–
447500
448000
448500
449000
449500
YMR090W
rrppolyA–
skipolyA–
xrnpolyA–
rrpTT–
skiTT–
xrnTT–
rrppolyA+
skipolyA+
xrnpolyA+
rrpTT+
skiTT+
xrnTT+
rrp
ski
xrn
wt
david–
david+
snyder
nucleosome
miura–
miura+
conservation
mcl.140, chr: 13

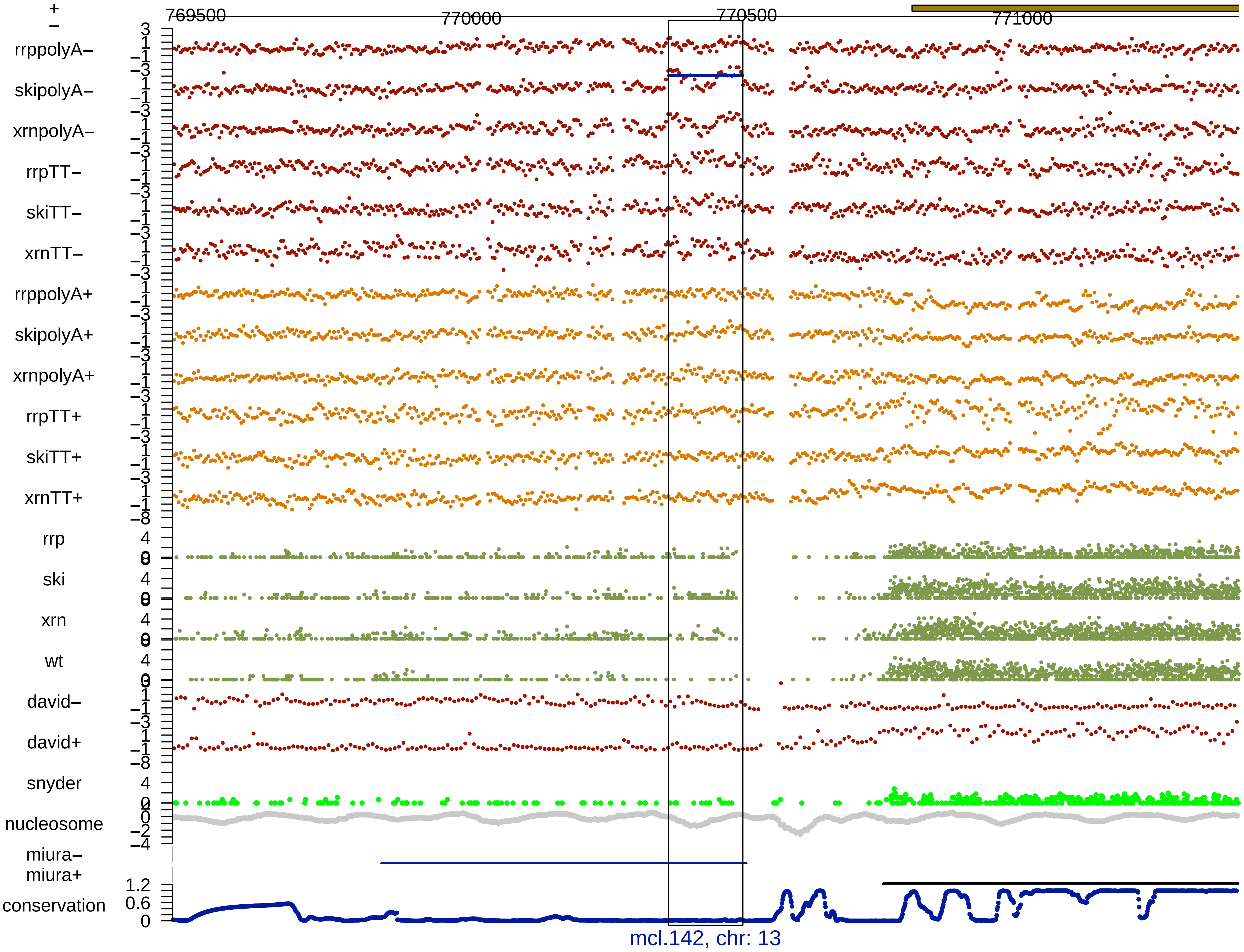

769500
770000
770500
771000
rrppolyA−
skipolyA−
xrnpolyA−
rrpTT−
skiTT−
xrnTT−
rrppolyA+
skipolyA+
xrnpolyA+
rrpTT+
skiTT+
xrnTT+
rrp
ski
xrn
wt
david−
david+
snyder
nucleosome
miura−
miura+
conservation
mcl.142, chr: 13

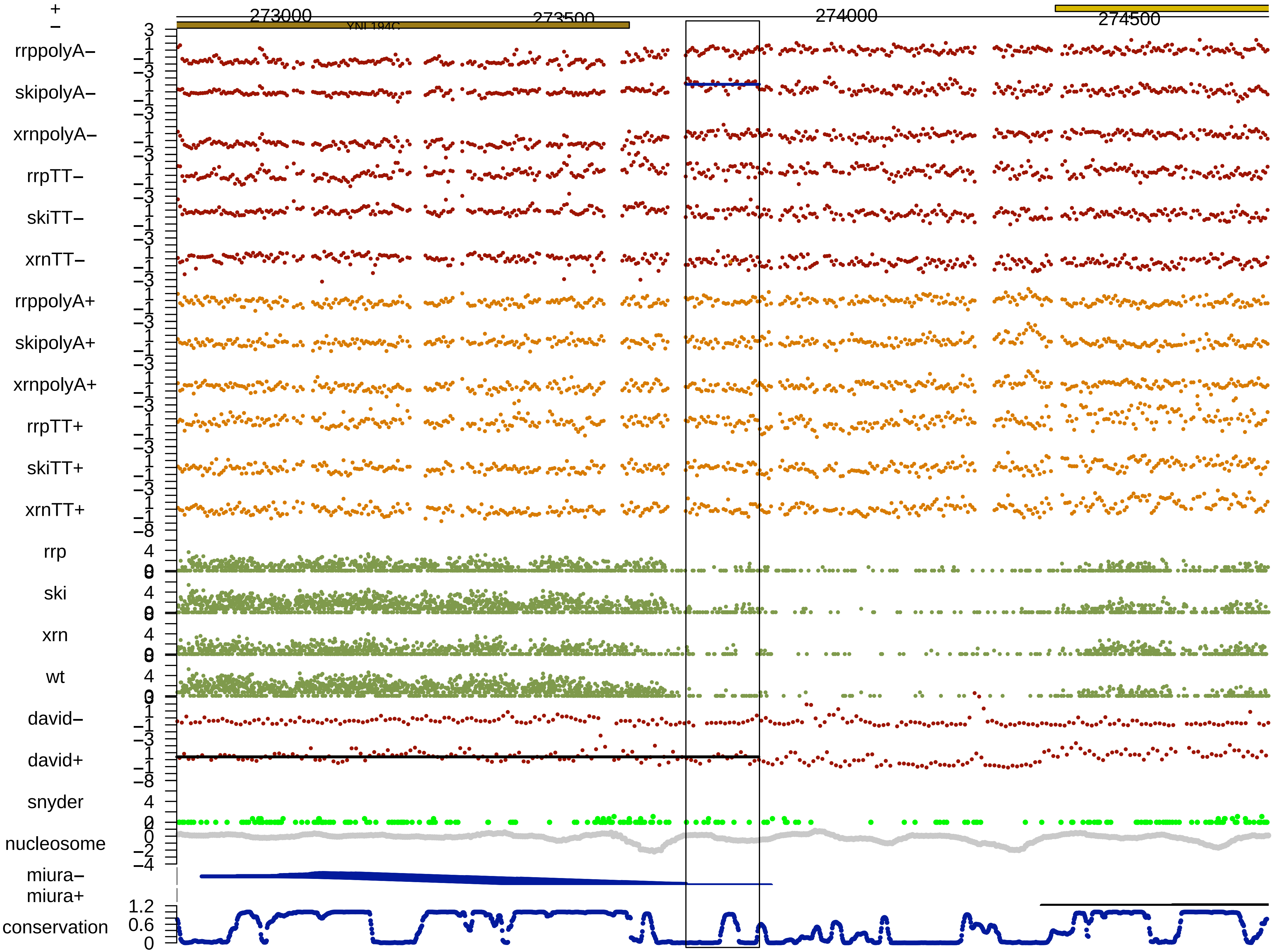

mcl.143, chr: 14

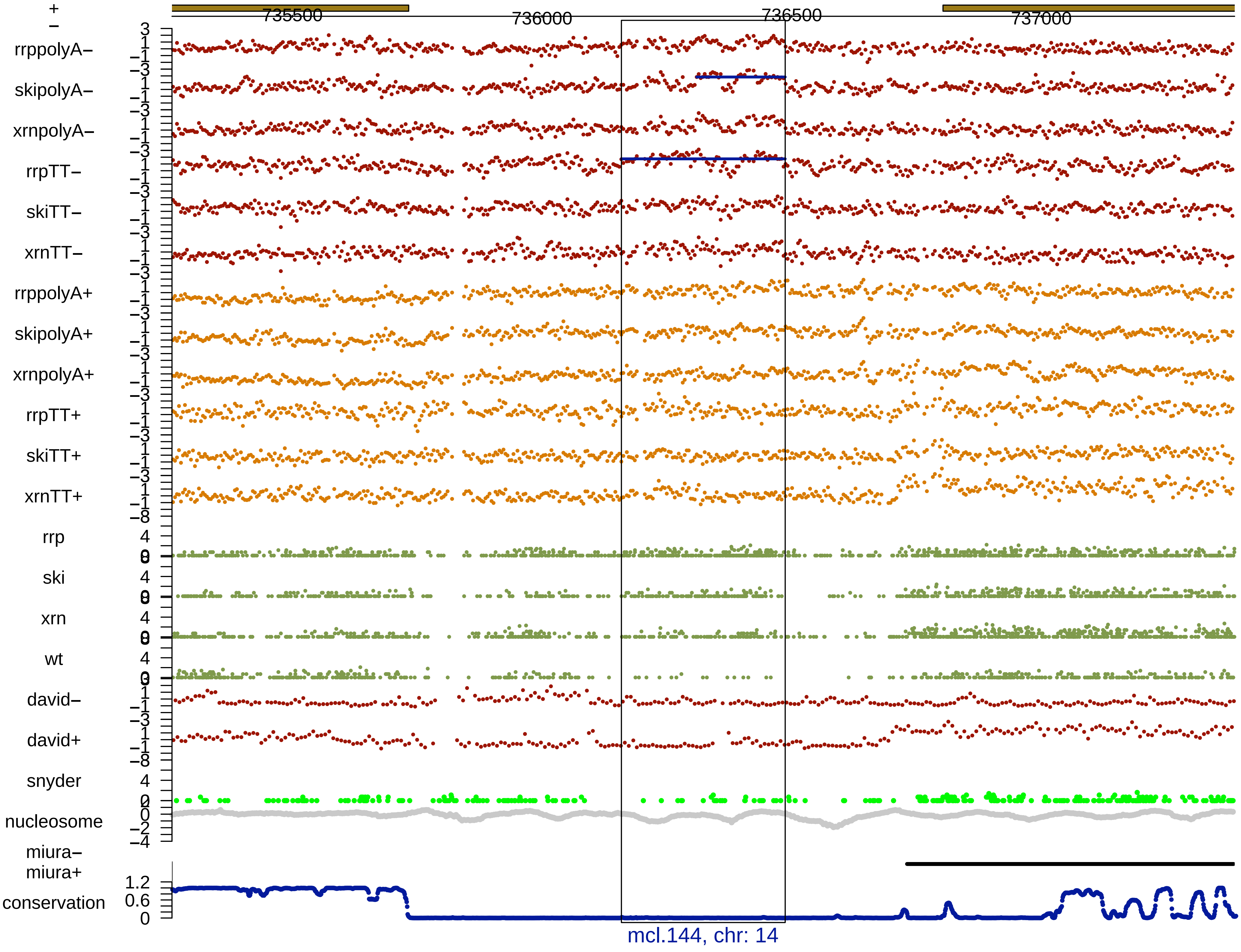
+
–
735500
736000
736500
737000
rrppolyA–
skipolyA–
xrnpolyA–
rrpTT–
skiTT–
xrnTT–
rrppolyA+
skipolyA+
xrnpolyA+
rrpTT+
skiTT+
xrnTT+
rrp
ski
xrn
wt
david–
david+
snyder
nucleosome
miura–
miura+
conservation
mcl.144, chr: 14

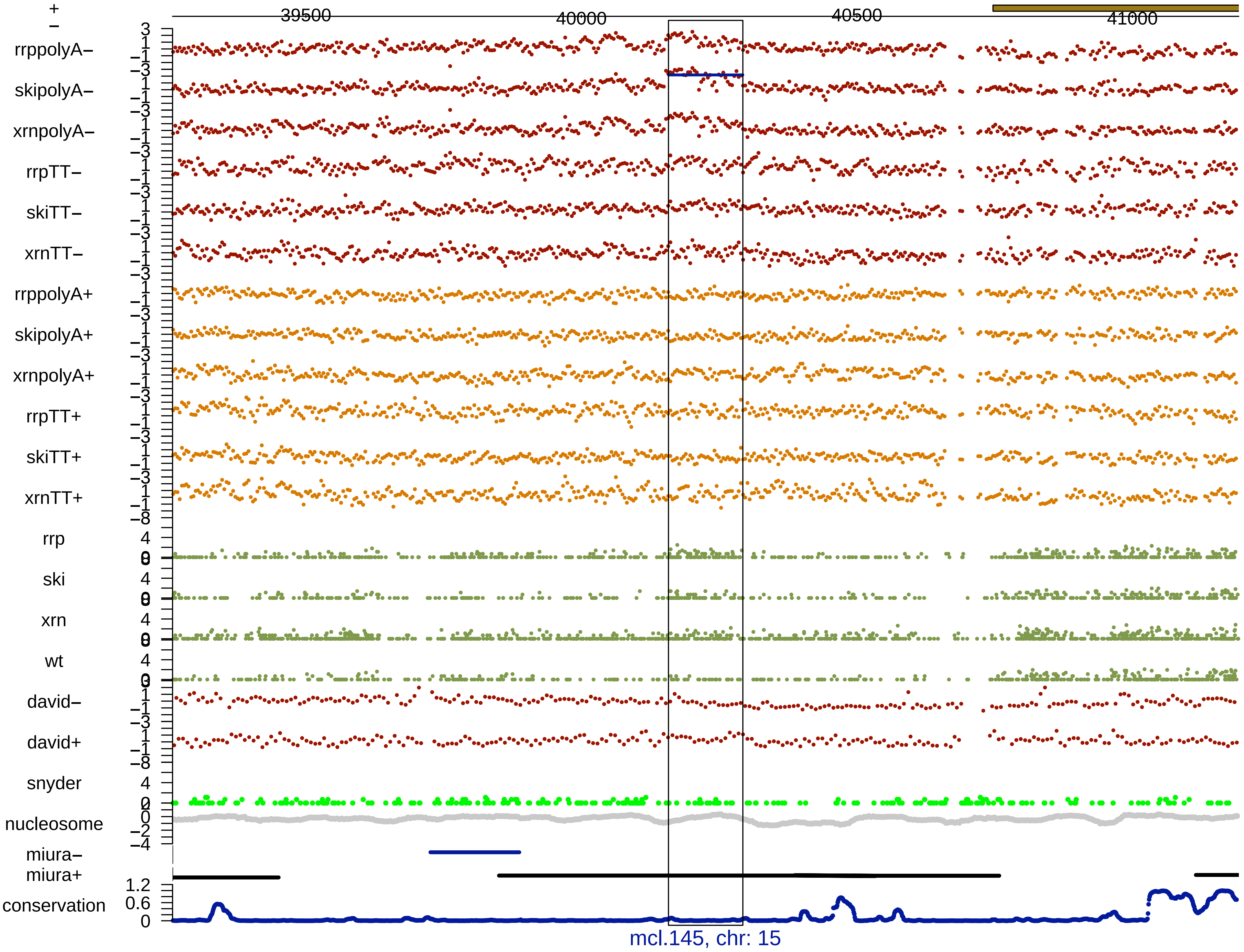

39500
40000
40500
41000
rrppolyA−
skipolyA−
xrnpolyA−
rrpTT−
skiTT−
xrnTT−
rrppolyA+
skipolyA+
xrnpolyA+
rrpTT+
skiTT+
xrnTT+
rrp
ski
xrn
wt
david−
david+
snyder
nucleosome
miura−
miura+
conservation
mcl.145, chr: 15

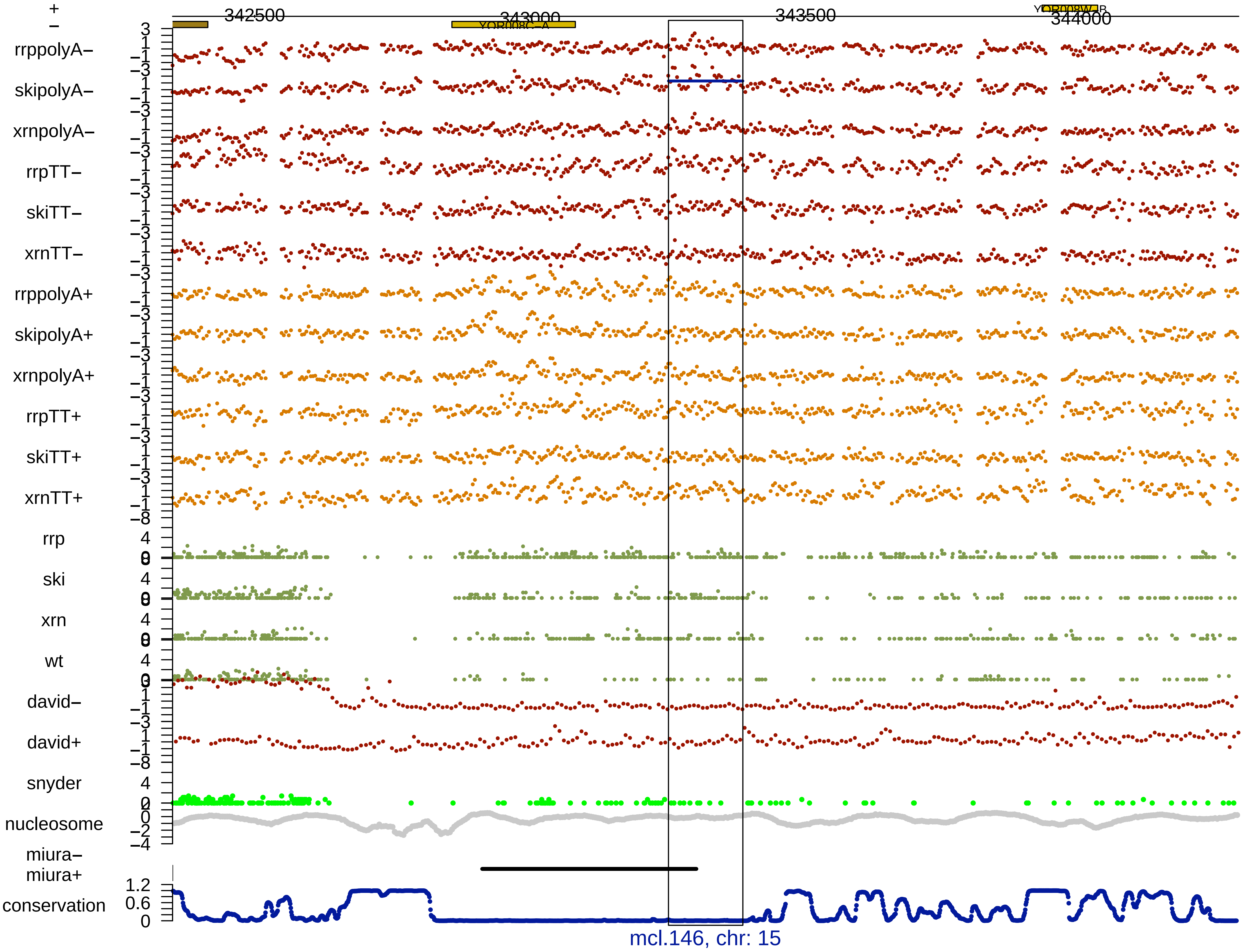

342500
343000
343500
344000
YOR008C-A
rrppolyA−
skipolyA−
xrnpolyA−
rrpTT−
skiTT−
xrnTT−
rrppolyA+
skipolyA+
xrnpolyA+
rrpTT+
skiTT+
xrnTT+
rrp
ski
xrn
wt
david−
david+
snyder
nucleosome
miura−
miura+
conservation
mcl.146, chr: 15

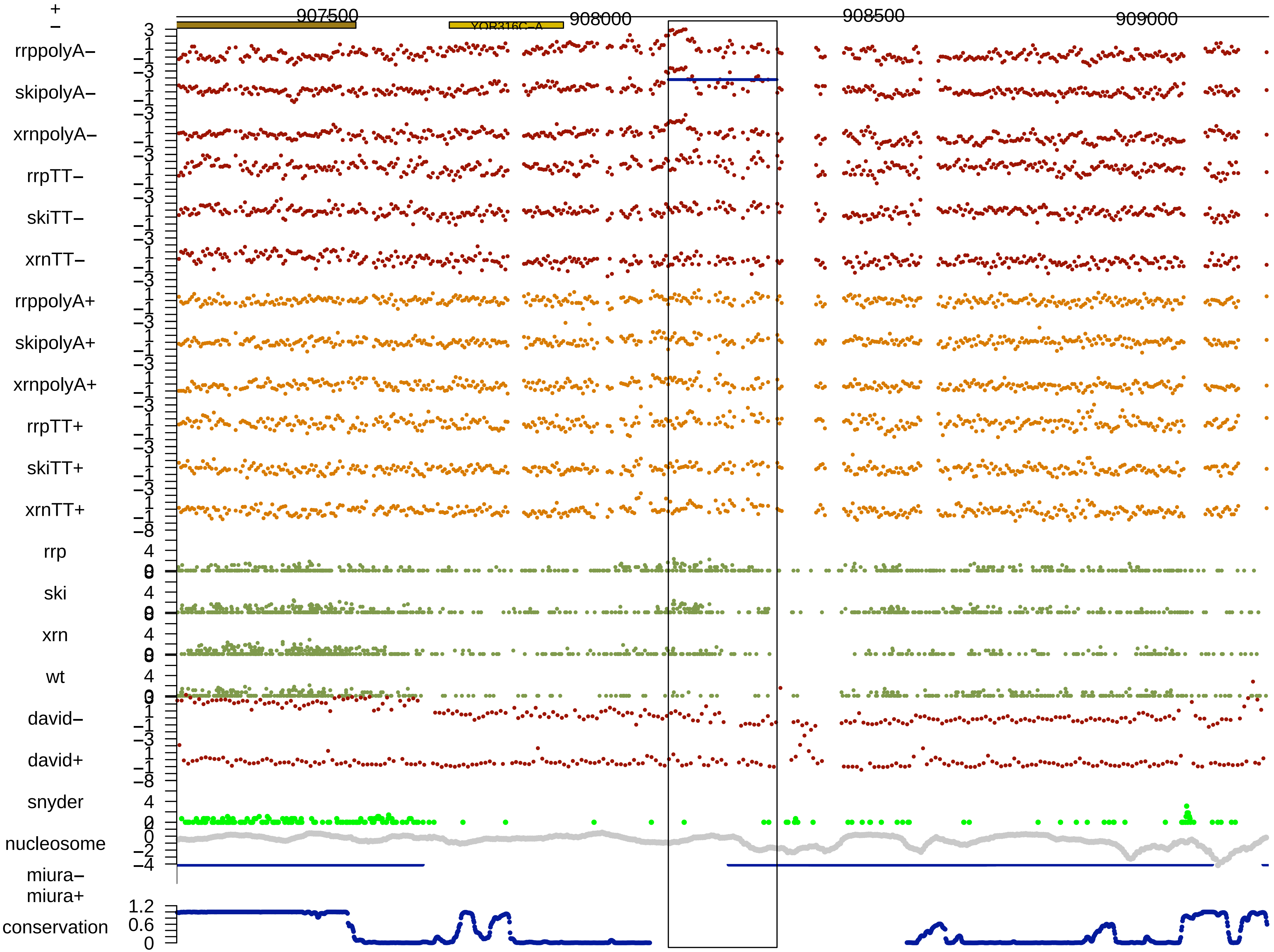

mcl.147, chr: 15

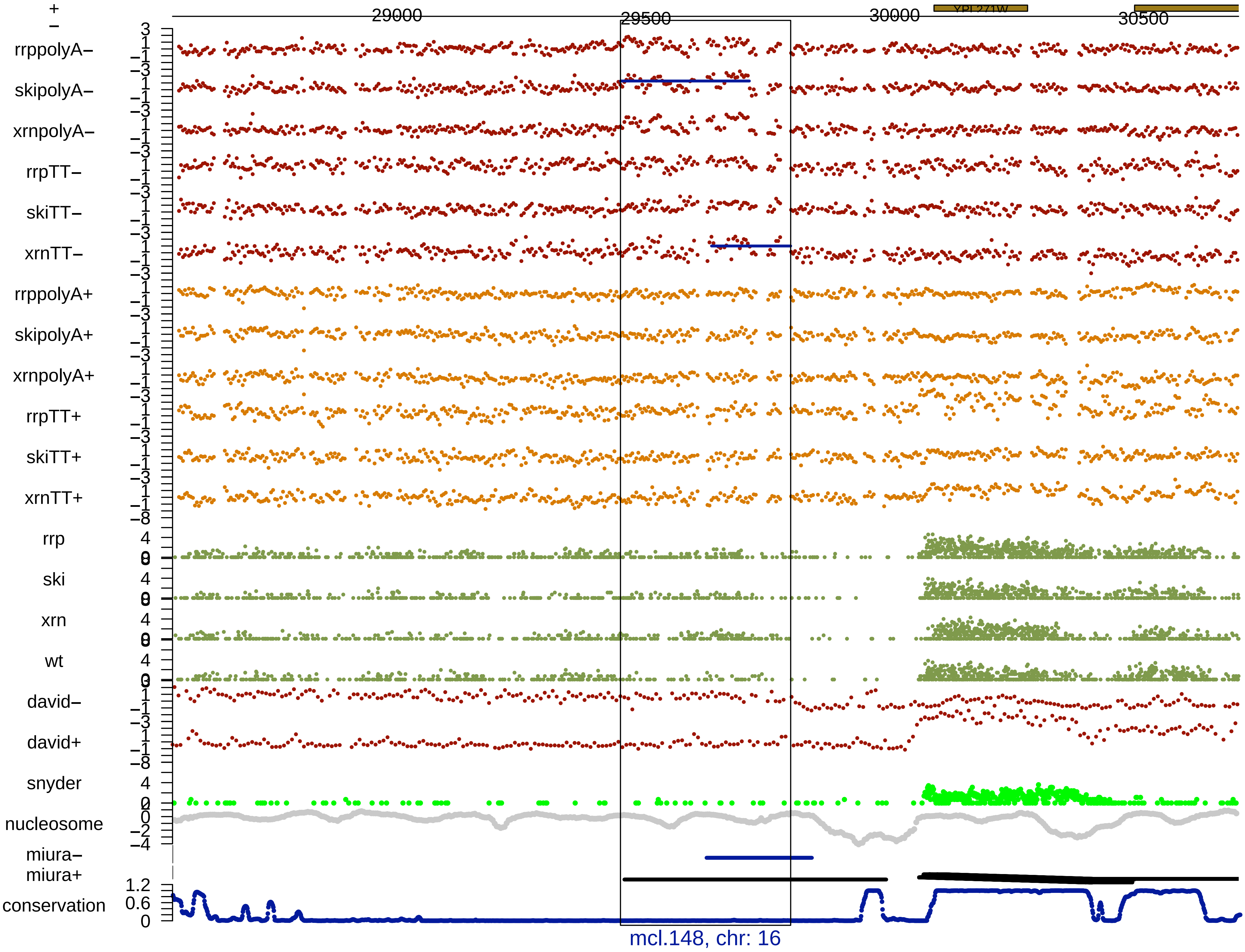

29000
29500
30000
30500
YPL271W
rrppolyA−
skipolyA−
xrnpolyA−
rrpTT−
skiTT−
xrnTT−
rrppolyA+
skipolyA+
xrnpolyA+
rrpTT+
skiTT+
xrnTT+
rrp
ski
xrn
wt
david−
david+
snyder
nucleosome
miura−
miura+
conservation
mcl.148, chr: 16

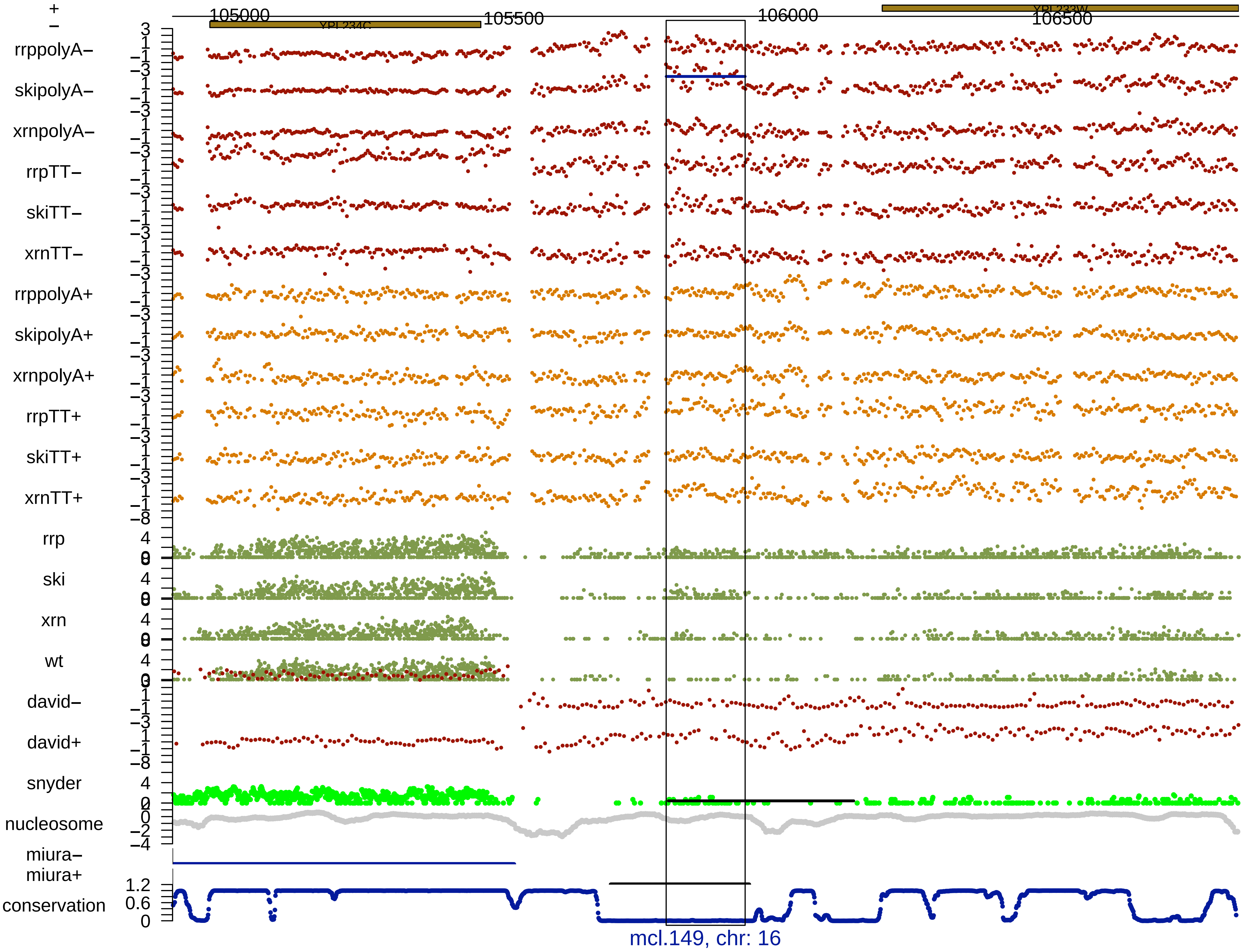

+
−
105000
105500
106000
106500
YPL234C
YPL233W
rrppolyA−
skipolyA−
xrnpolyA−
rrpTT−
skiTT−
xrnTT−
rrppolyA+
skipolyA+
xrnpolyA+
rrpTT+
skiTT+
xrnTT+
rrp
ski
xrn
wt
david−
david+
snyder
nucleosome
miura−
miura+
conservation
mcl.149, chr: 16

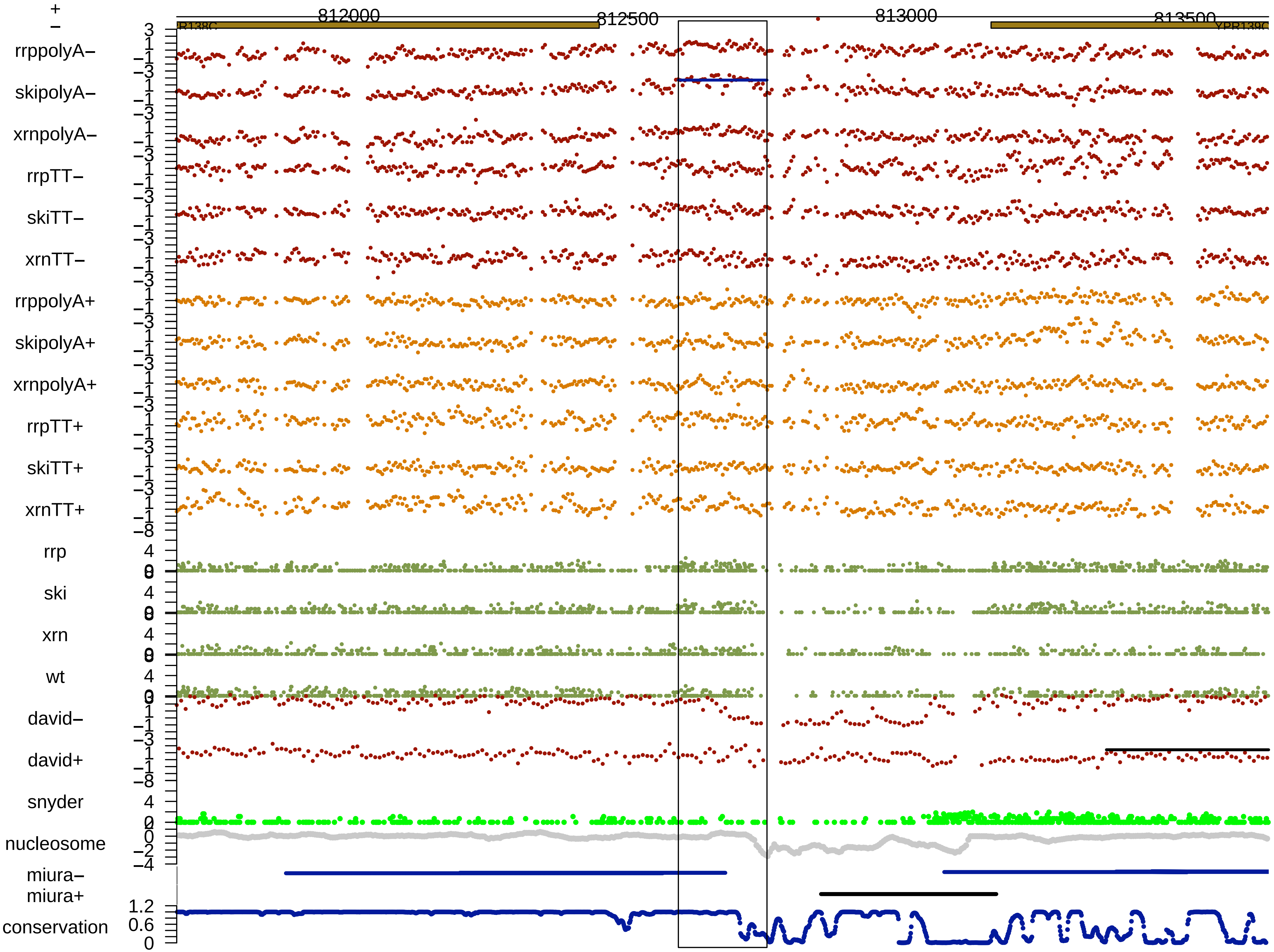

mcl.151, chr: 16

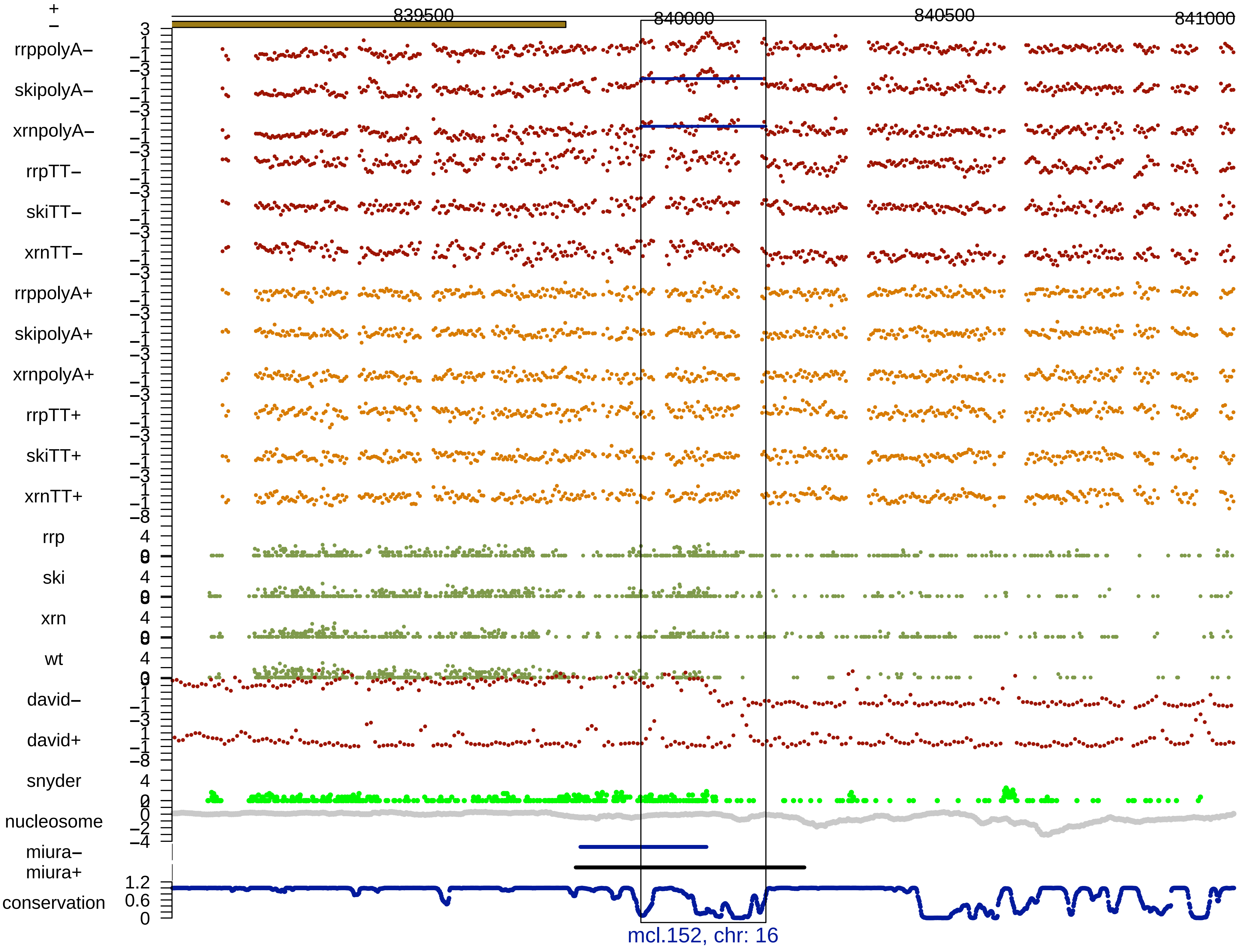
839500
840000
840500
841000
+
−
rrppolyA−
skipolyA−
xrnpolyA−
rrpTT−
skiTT−
xrnTT−
rrppolyA+
skipolyA+
xrnpolyA+
rrpTT+
skiTT+
xrnTT+
rrp
ski
xrn
wt
david−
david+
snyder
nucleosome
miura−
miura+
conservation
mcl.152, chr: 16

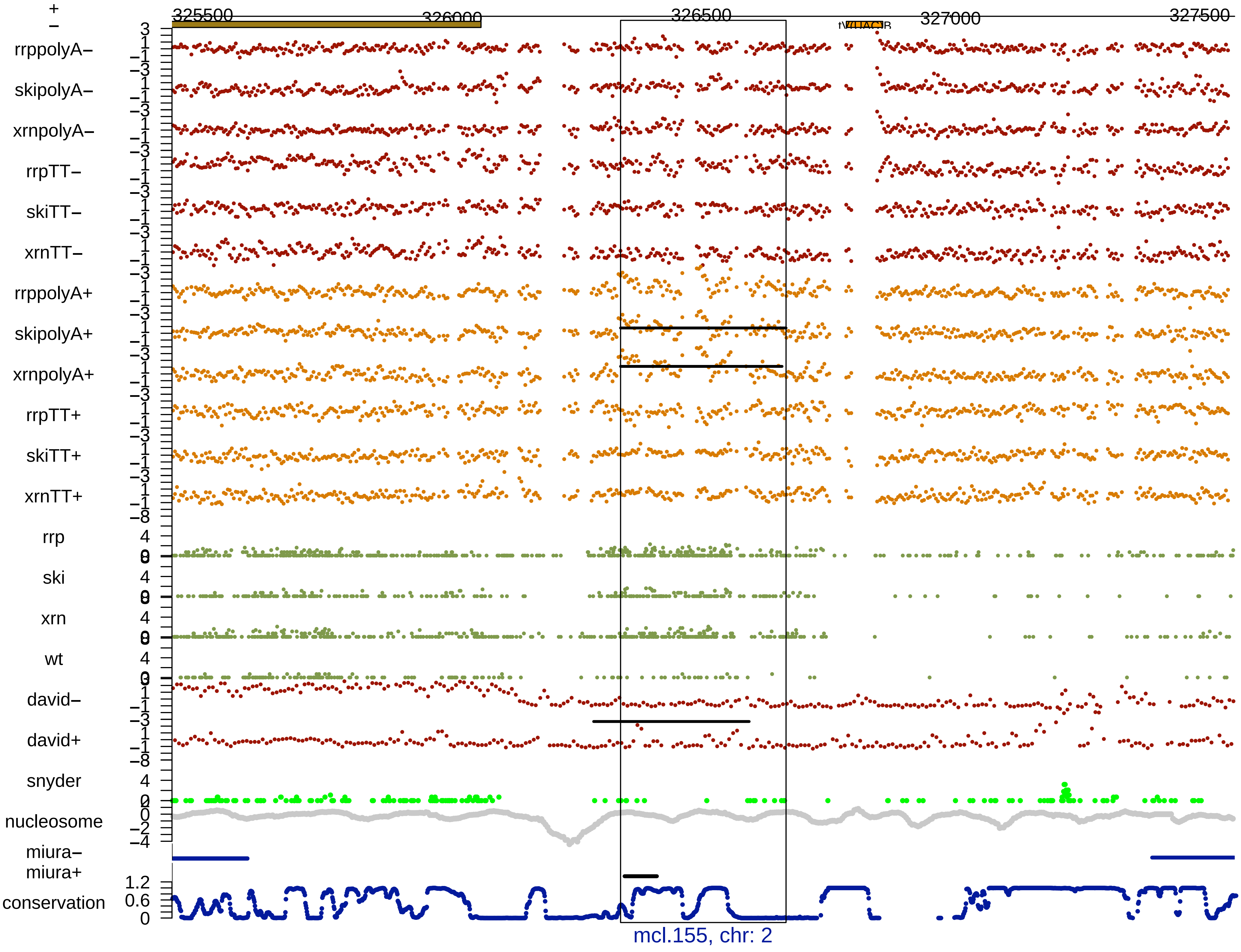
325500
326000
326500
327000
327500
rrppolyA−
skipolyA−
xrnpolyA−
rrpTT−
skiTT−
xrnTT−
rrppolyA+
skipolyA+
xrnpolyA+
rrpTT+
skiTT+
xrnTT+
rrp
ski
xrn
wt
david−
david+
snyder
nucleosome
miura−
miura+
conservation
mcl.155, chr: 2

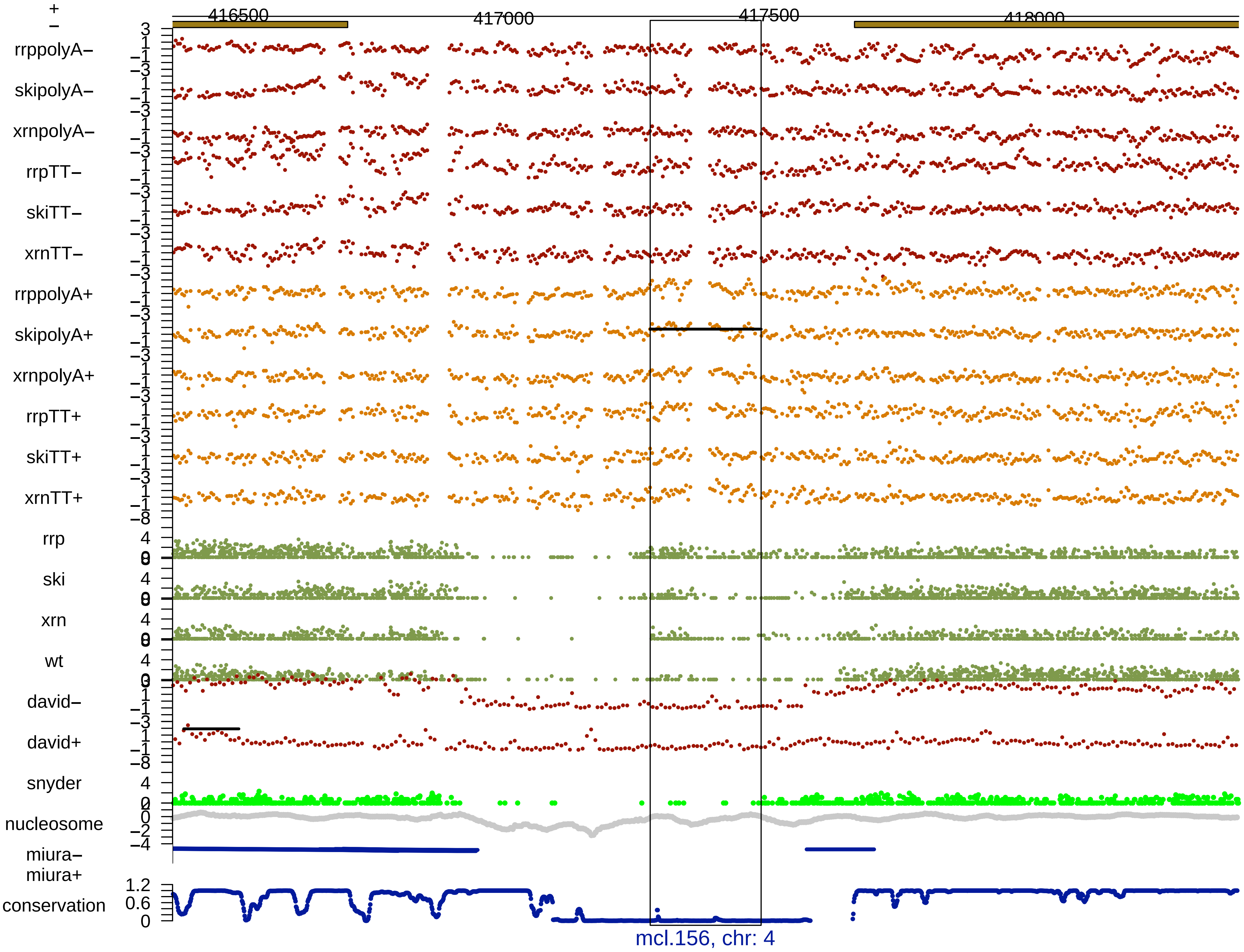

416500
417000
417500
418000
rrppolyA−
skipolyA−
xrnpolyA−
rrpTT−
skiTT−
xrnTT−
rrppolyA+
skipolyA+
xrnpolyA+
rrpTT+
skiTT+
xrnTT+
rrp
ski
xrn
wt
david−
david+
snyder
nucleosome
miura−
miura+
conservation
mcl.156, chr: 4

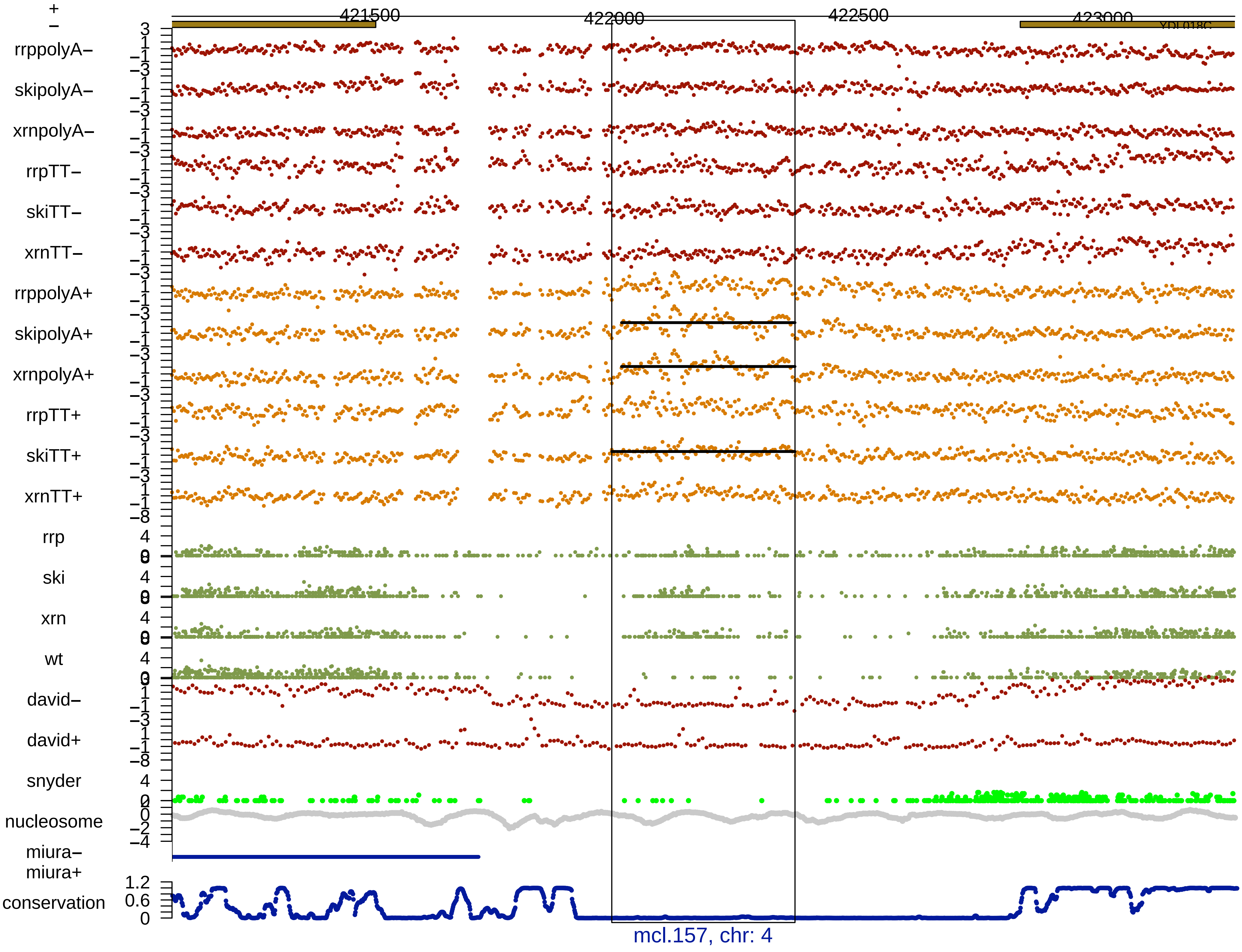

+
−
421500
422000
422500
423000
rrppolyA−
skipolyA−
xrnpolyA−
rrpTT−
skiTT−
xrnTT−
rrppolyA+
skipolyA+
xrnpolyA+
rrpTT+
skiTT+
xrnTT+
rrp
ski
xrn
wt
david−
david+
snyder
nucleosome
miura−
miura+
conservation
mcl.157, chr: 4

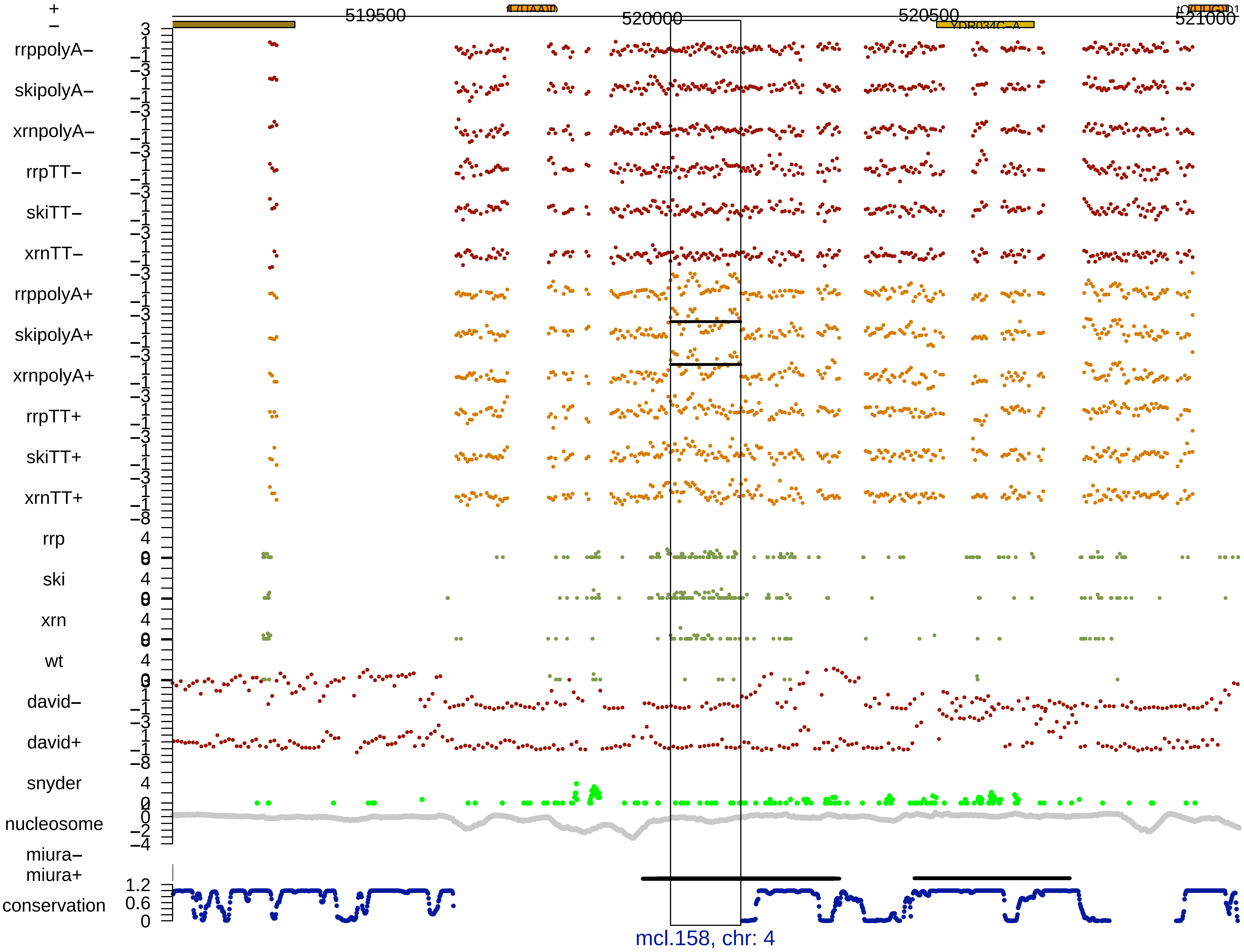

519500
520000
520500
521000
YDR034C-A
rrppolyA−
skipolyA−
xrnpolyA−
rrpTT−
skiTT−
xrnTT−
rrppolyA+
skipolyA+
xrnpolyA+
rrpTT+
skiTT+
xrnTT+
rrp
ski
xrn
wt
david−
david+
snyder
nucleosome
miura−
miura+
conservation
mcl.158, chr: 4

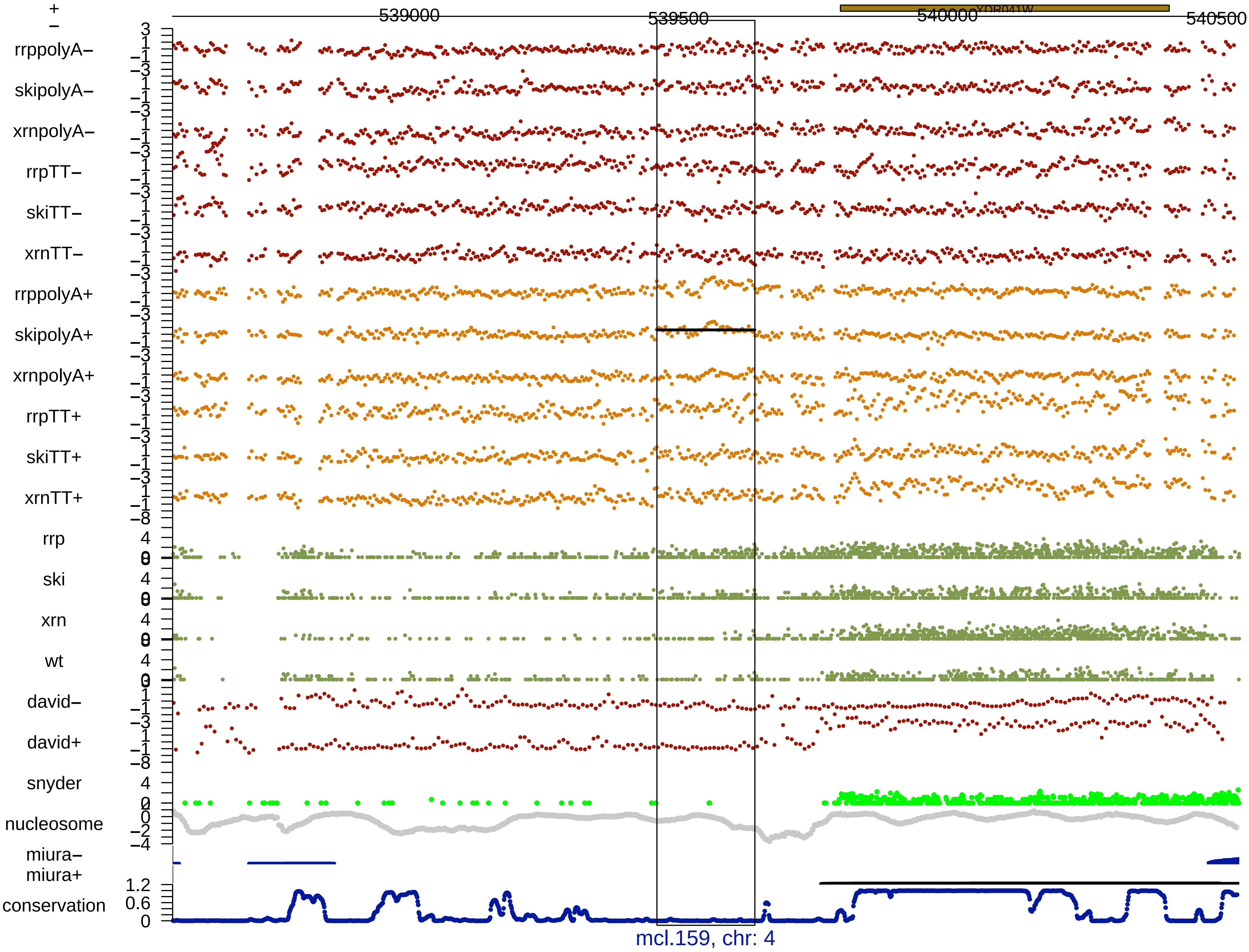

+
−
539000
539500
540000
YDR041W
540500
rrppolyA−
skipolyA−
xrnpolyA−
rrpTT−
skiTT−
xrnTT−
rrppolyA+
skipolyA+
xrnpolyA+
rrpTT+
skiTT+
xrnTT+
rrp
ski
xrn
wt
david−
david+
snyder
nucleosome
miura−
miura+
conservation
mcl.159, chr: 4

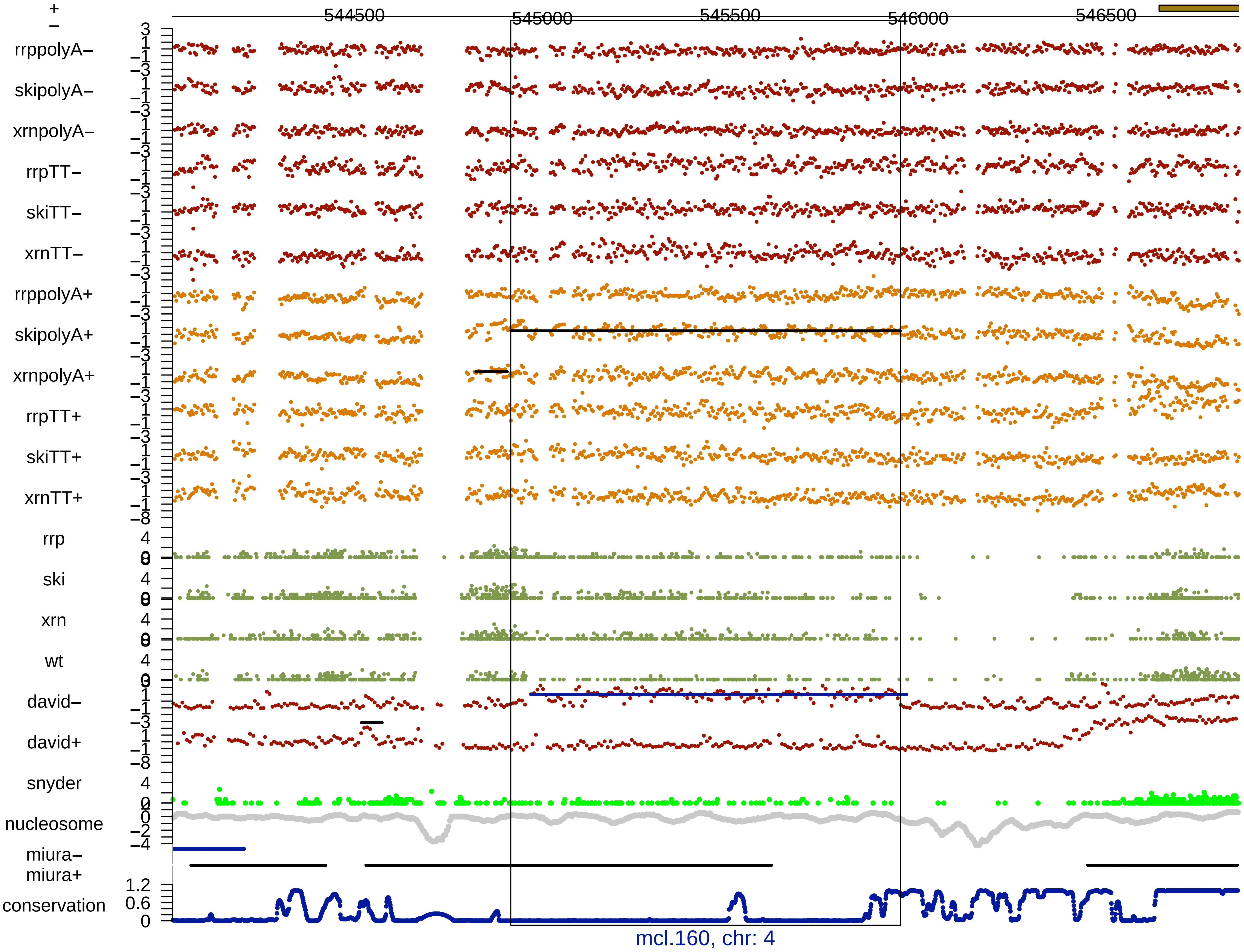

544500
545000
545500
546000
546500
rrppolyA−
skipolyA−
xrnpolyA−
rrpTT−
skiTT−
xrnTT−
rrppolyA+
skipolyA+
xrnpolyA+
rrpTT+
skiTT+
xrnTT+
rrp
ski
xrn
wt
david−
david+
snyder
nucleosome
miura−
miura+
conservation
mcl.160, chr: 4

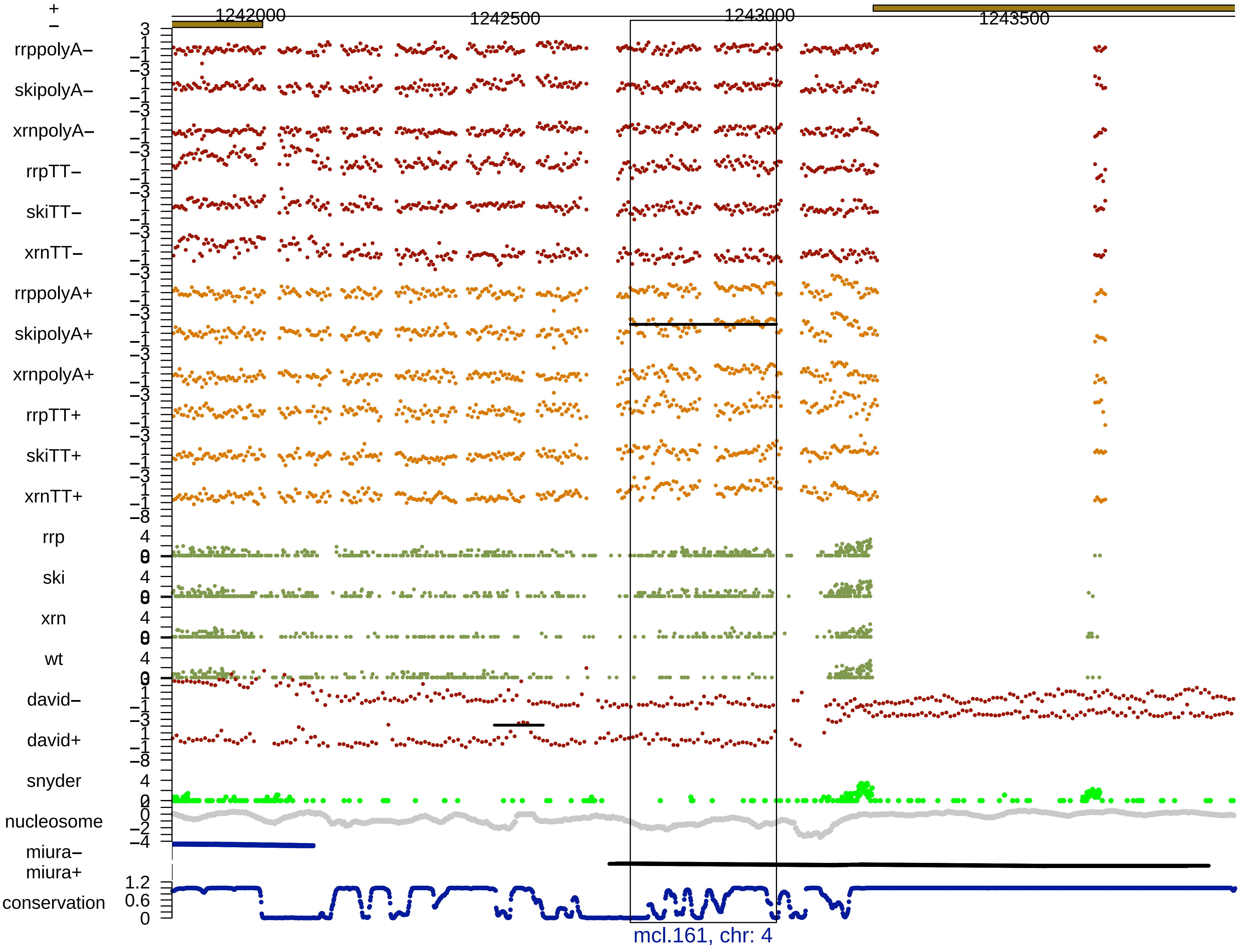

+
−
1242000
1242500
1243000
1243500
rrppolyA−
skipolyA−
xrnpolyA−
rrpTT−
skiTT−
xrnTT−
rrppolyA+
skipolyA+
xrnpolyA+
rrpTT+
skiTT+
xrnTT+
rrp
ski
xrn
wt
david−
david+
snyder
nucleosome
miura−
miura+
conservation
1.2
0.6
0
mcl.161, chr: 4

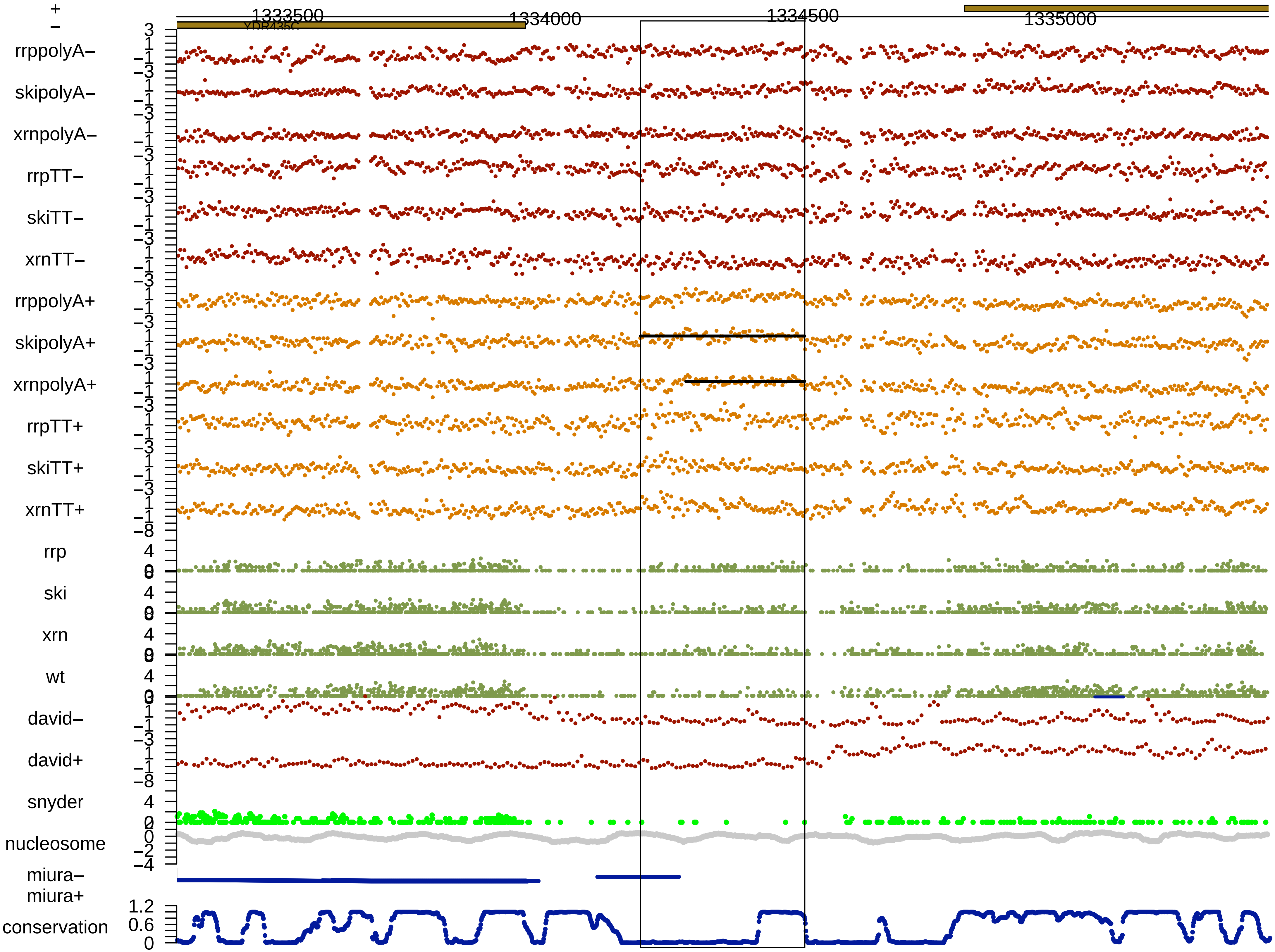
1333500
1334000
1334500
1335000
YDR435C
+
–
rrppolyA–
skipolyA–
xrnpolyA–
rrpTT–
skiTT–
xrnTT–
rrppolyA+
skipolyA+
xrnpolyA+
rrpTT+
skiTT+
xrnTT+
rrp
ski
xrn
wt
david–
david+
snyder
nucleosome
miura–
miura+
conservation
mcl.162, chr: 4

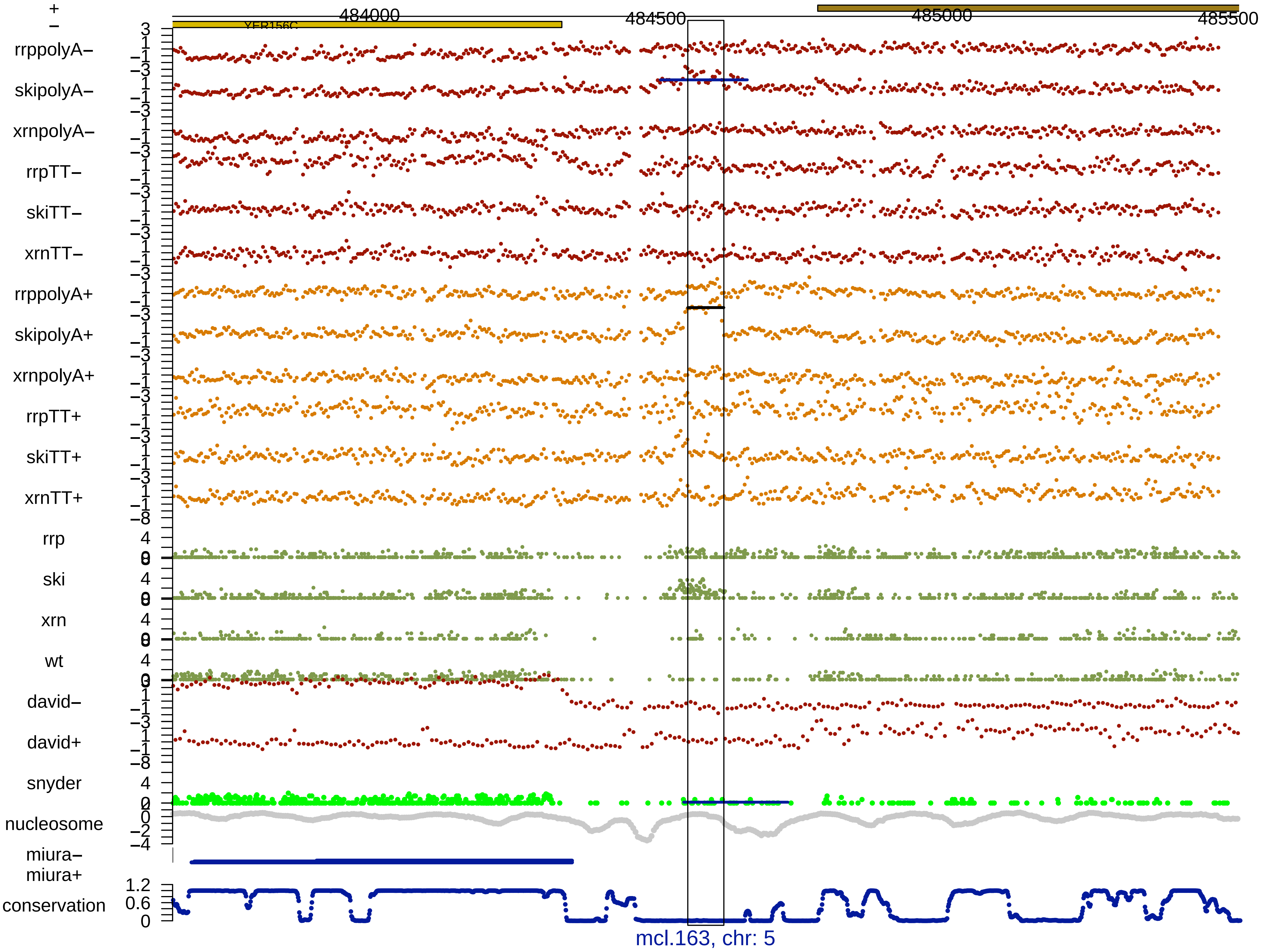

+
−
484000
484500
485000
485500
rrppolyA−
skipolyA−
xrnpolyA−
rrpTT−
skiTT−
xrnTT−
rrppolyA+
skipolyA+
xrnpolyA+
rrpTT+
skiTT+
xrnTT+
rrp
ski
xrn
wt
david−
david+
snyder
nucleosome
miura−
miura+
conservation
mcl.163, chr: 5

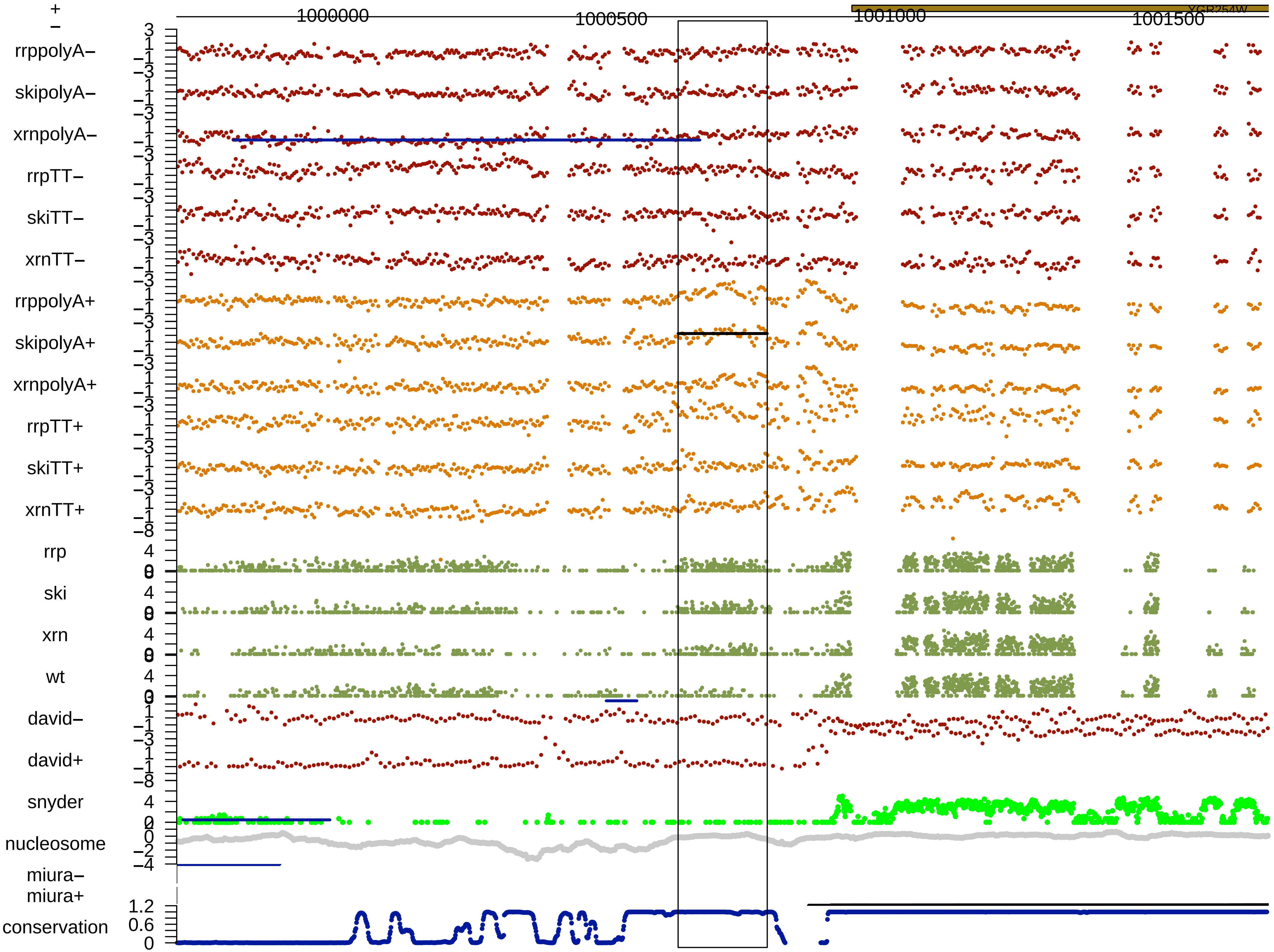
1000000
1000500
1001000
1001500
YGR254W
rrppolyA−
skipolyA−
xrnpolyA−
rrpTT−
skiTT−
xrnTT−
rrppolyA+
skipolyA+
xrnpolyA+
rrpTT+
skiTT+
xrnTT+
rrp
ski
xrn
wt
david−
david+
snyder
nucleosome
miura−
miura+
conservation

mcl.165, chr: 7

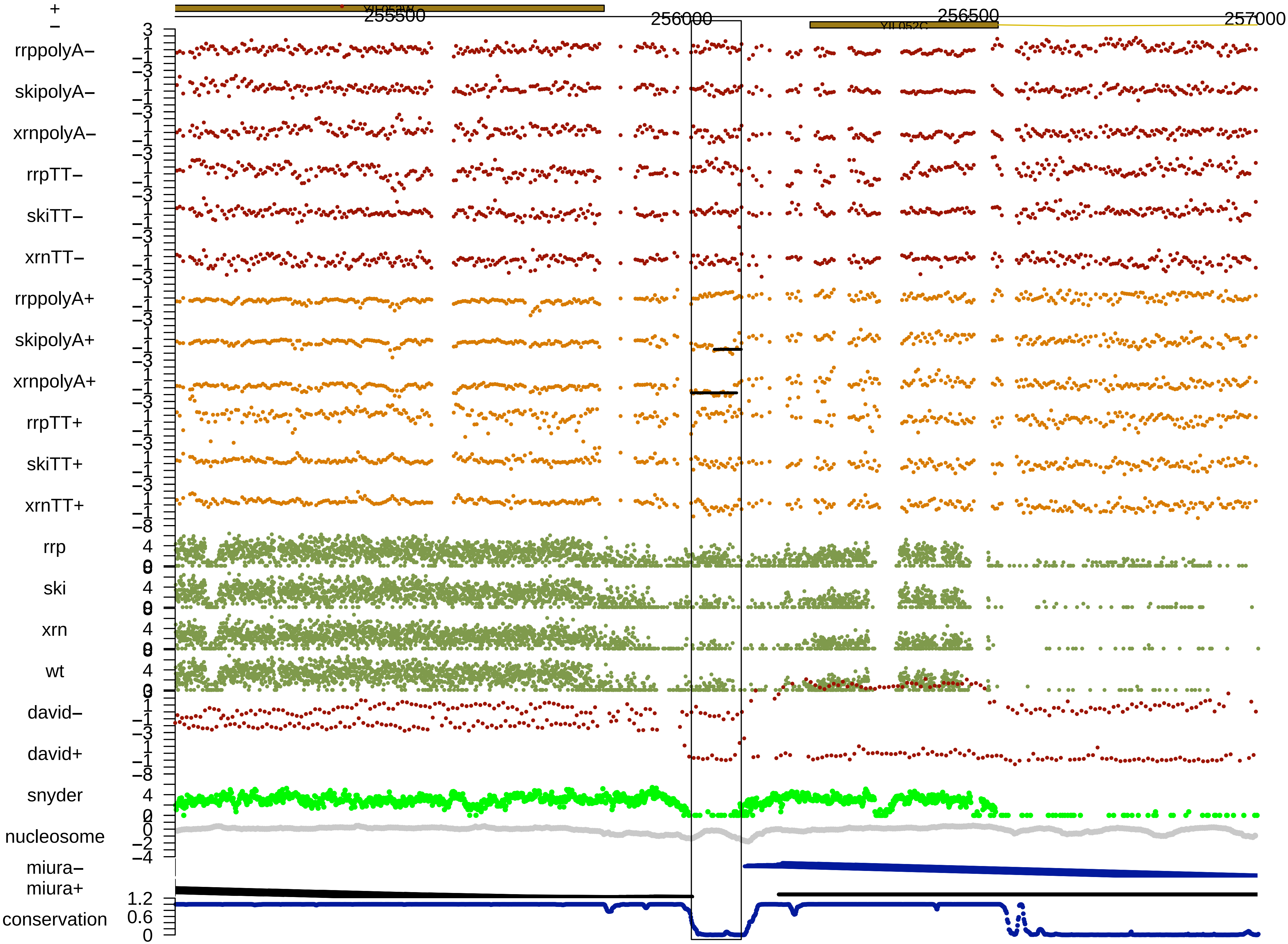
+
–
255500
256000
256500
257000
rrppolyA–
skipolyA–
xrnpolyA–
rrpTT–
skiTT–
xrnTT–
rrppolyA+
skipolyA+
xrnpolyA+
rrpTT+
skiTT+
xrnTT+
rrp
ski
xrn
wt
david–
david+
snyder
nucleosome
miura–
miura+
conservation

mcl.167, chr: 9

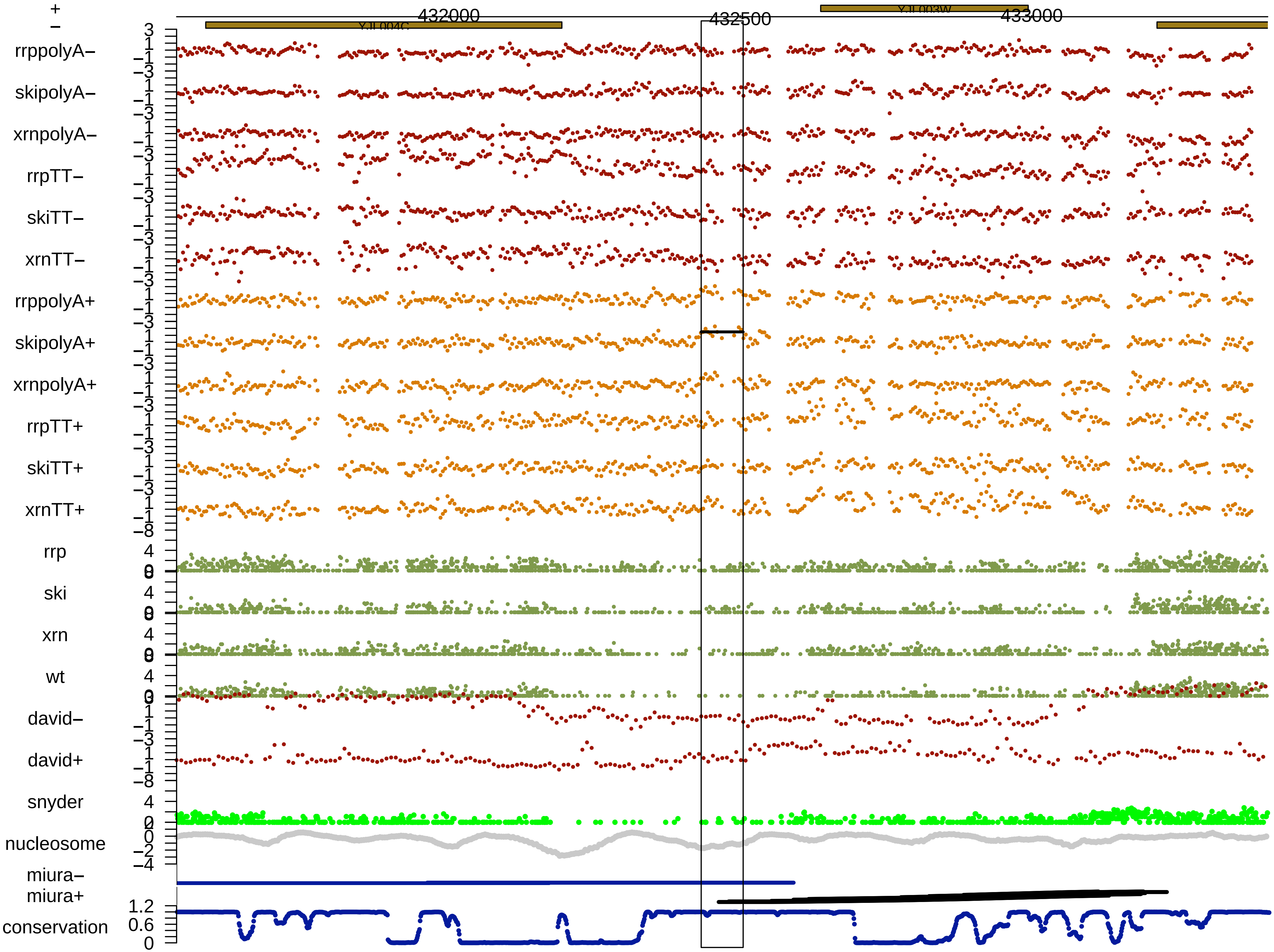

mcl.168, chr: 10

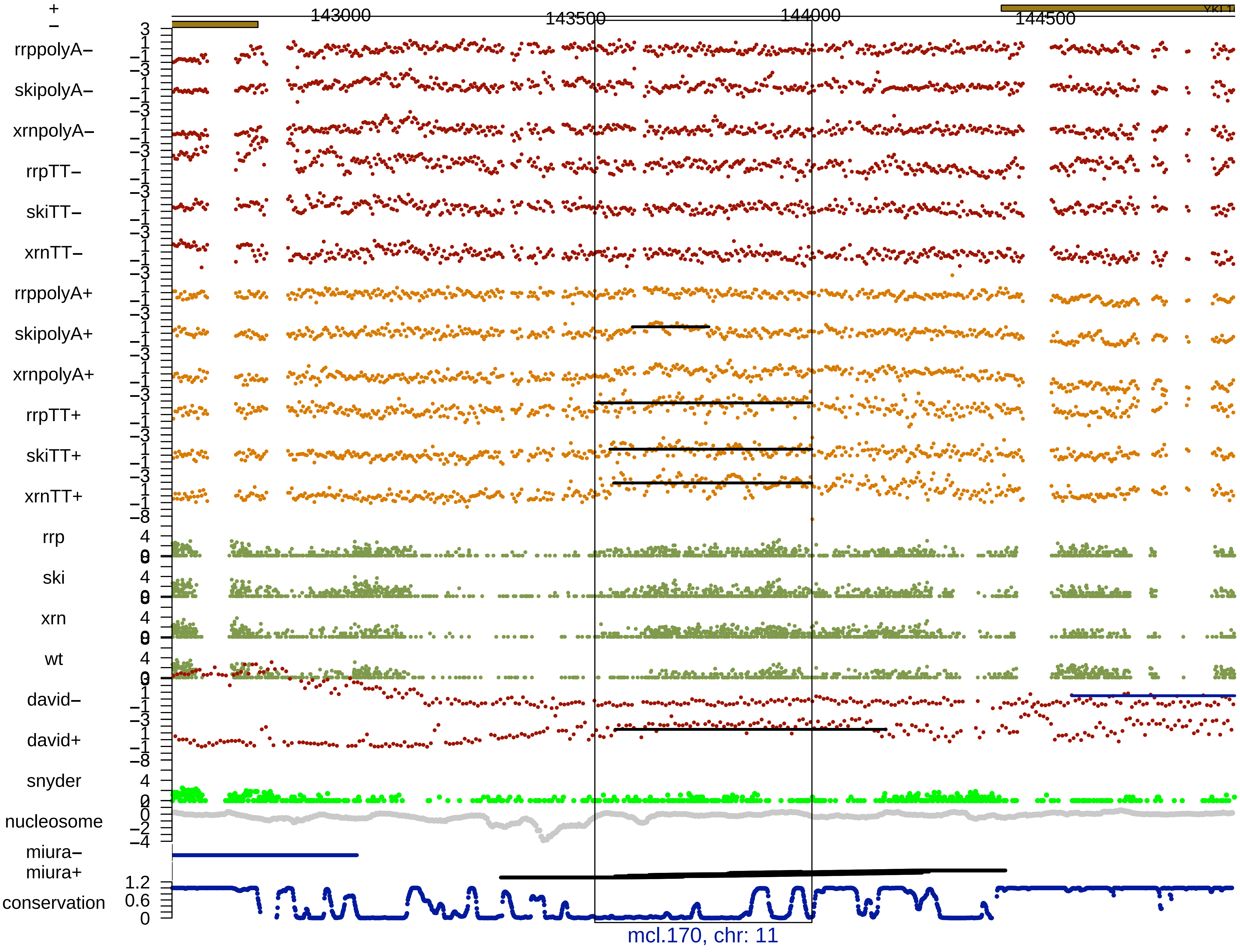

143000
143500
144000
144500
rrppolyA−
skipolyA−
xrnpolyA−
rrpTT−
skiTT−
xrnTT−
rrppolyA+
skipolyA+
xrnpolyA+
rrpTT+
skiTT+
xrnTT+
rrp
ski
xrn
wt
david−
david+
snyder
nucleosome
miura−
miura+
conservation
mcl.170, chr: 11

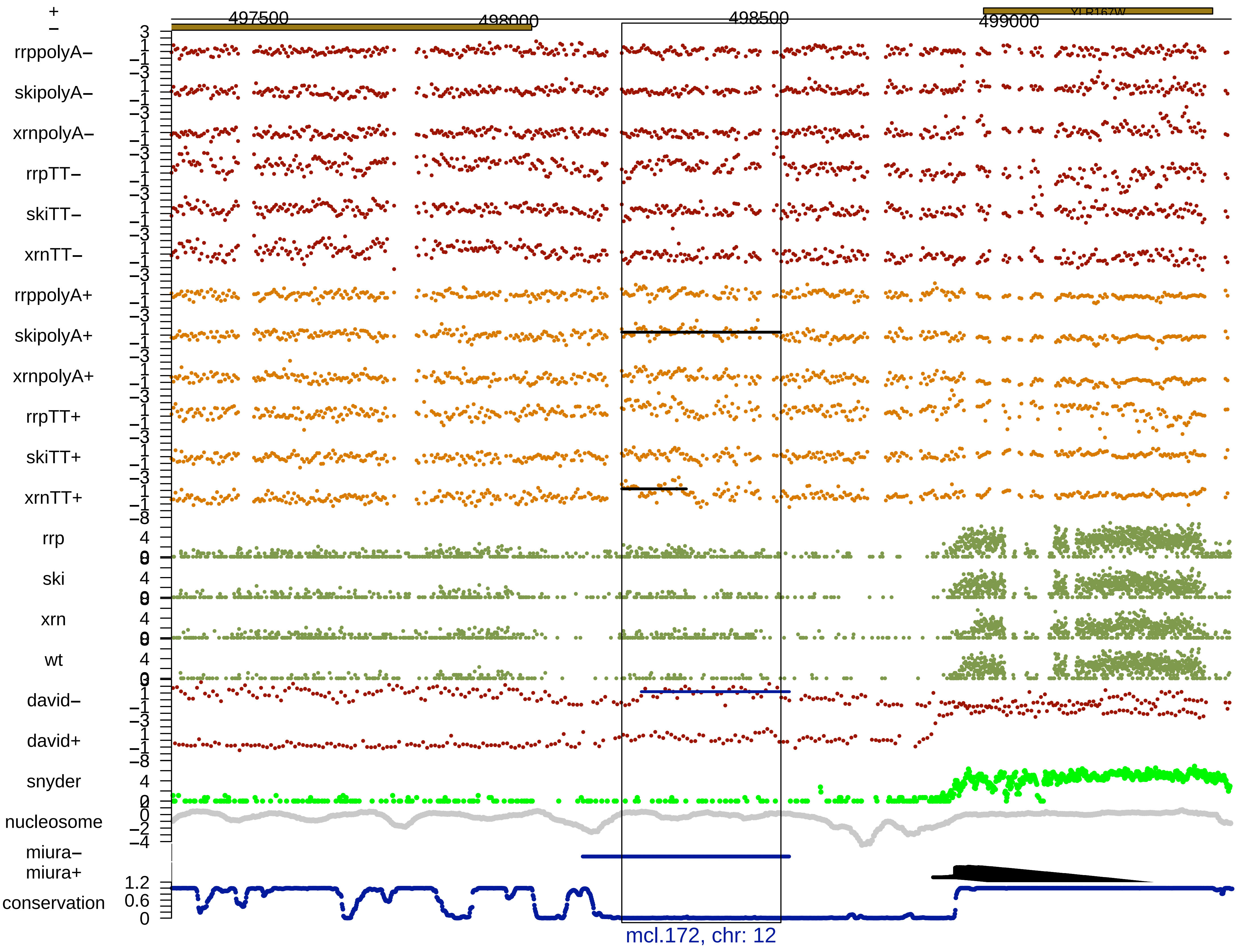

+
−
497500
498000
498500
499000
YLR167W
rrppolyA−
skipolyA−
xrnpolyA−
rrpTT−
skiTT−
xrnTT−
rrppolyA+
skipolyA+
xrnpolyA+
rrpTT+
skiTT+
xrnTT+
rrp
ski
xrn
wt
david−
david+
snyder
nucleosome
miura−
miura+
conservation
mcl.172, chr: 12

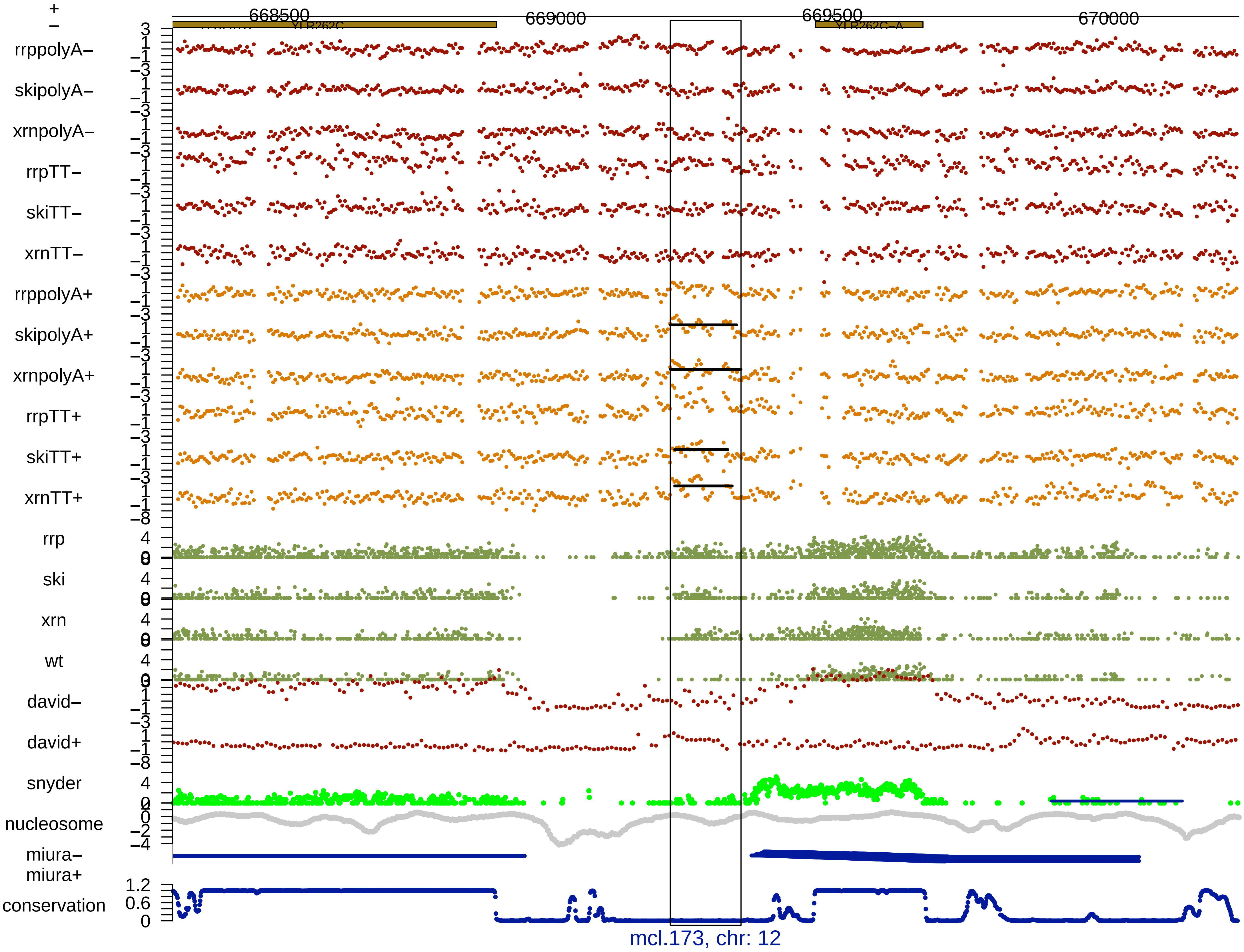

668500
669000
669500
670000
YLR262C
YLR262C-A
rrppolyA−
skipolyA−
xrnpolyA−
rrpTT−
skiTT−
xrnTT−
rrppolyA+
skipolyA+
xrnpolyA+
rrpTT+
skiTT+
xrnTT+
rrp
ski
xrn
wt
david−
david+
snyder
nucleosome
miura−
miura+
conservation
mcl.173, chr: 12

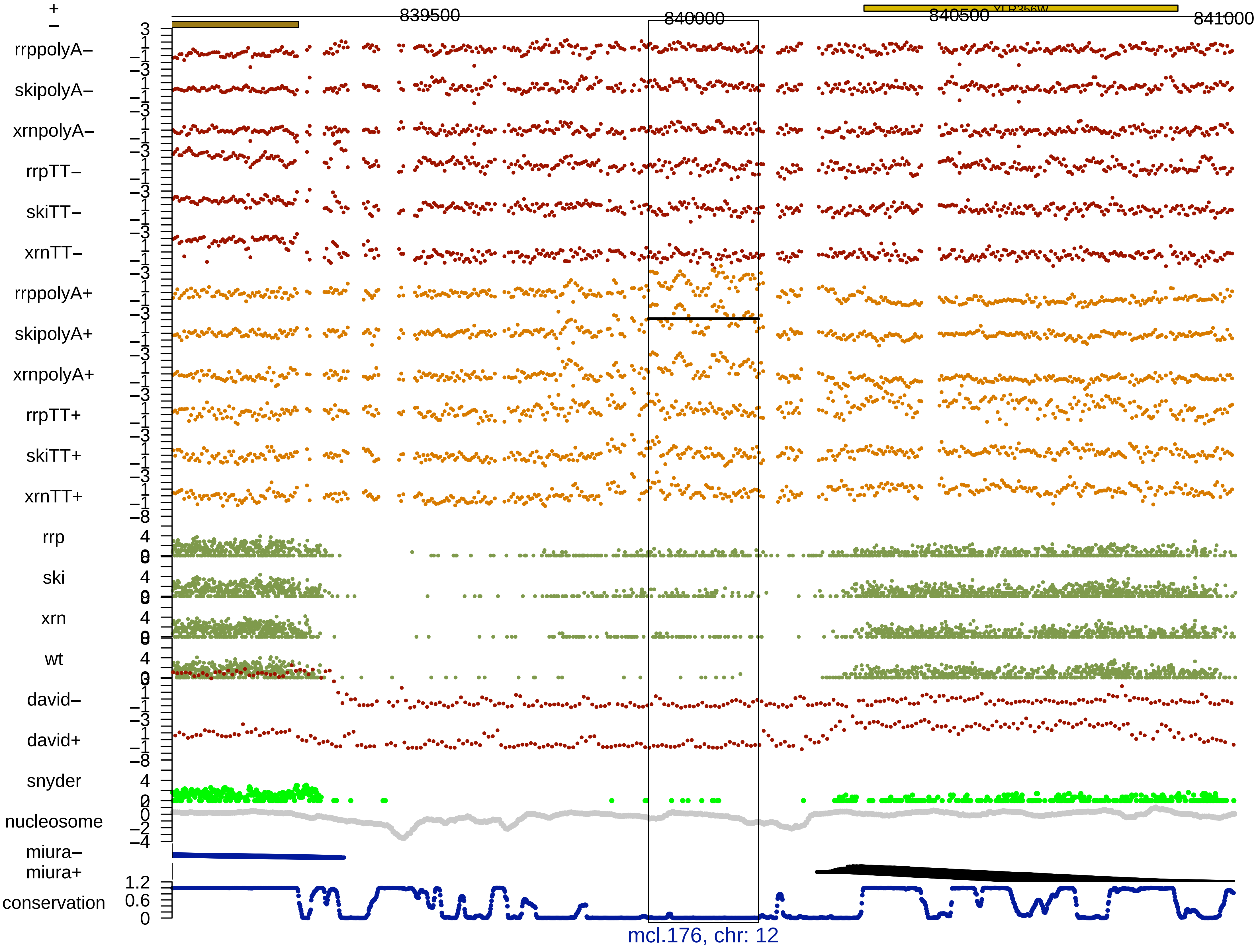

839500
840000
840500
841000
YLR356W
+
−
rrppolyA−
skipolyA−
xrnpolyA−
rrpTT−
skiTT−
xrnTT−
rrppolyA+
skipolyA+
xrnpolyA+
rrpTT+
skiTT+
xrnTT+
rrp
ski
xrn
wt
david−
david+
snyder
nucleosome
miura−
miura+
conservation
mcl.176, chr: 12

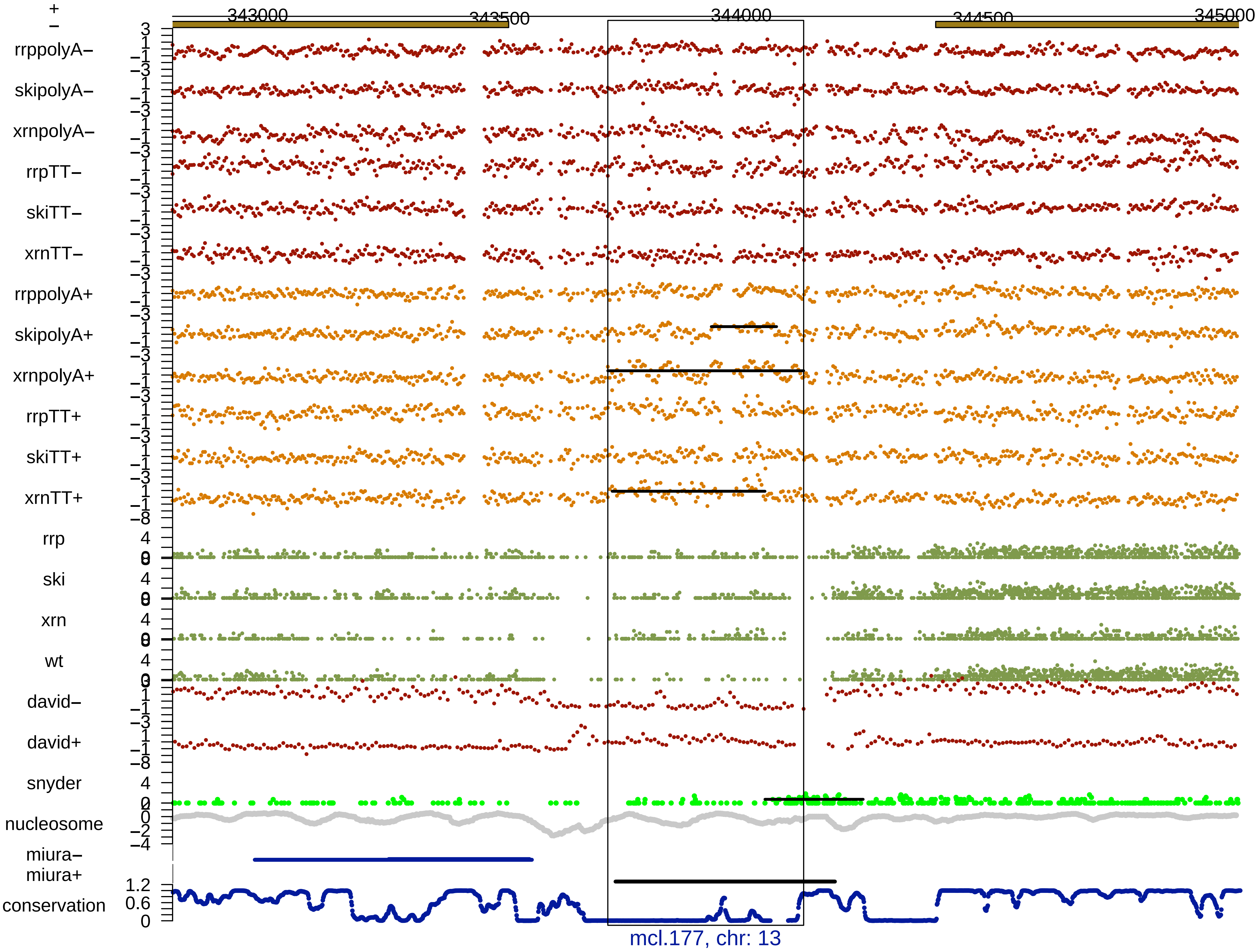

343000
343500
344000
344500
345000
rrppolyA−
skipolyA−
xrnpolyA−
rrpTT−
skiTT−
xrnTT−
rrppolyA+
skipolyA+
xrnpolyA+
rrpTT+
skiTT+
xrnTT+
rrp
ski
xrn
wt
david−
david+
snyder
nucleosome
miura−
miura+
conservation
mcl.177, chr: 13

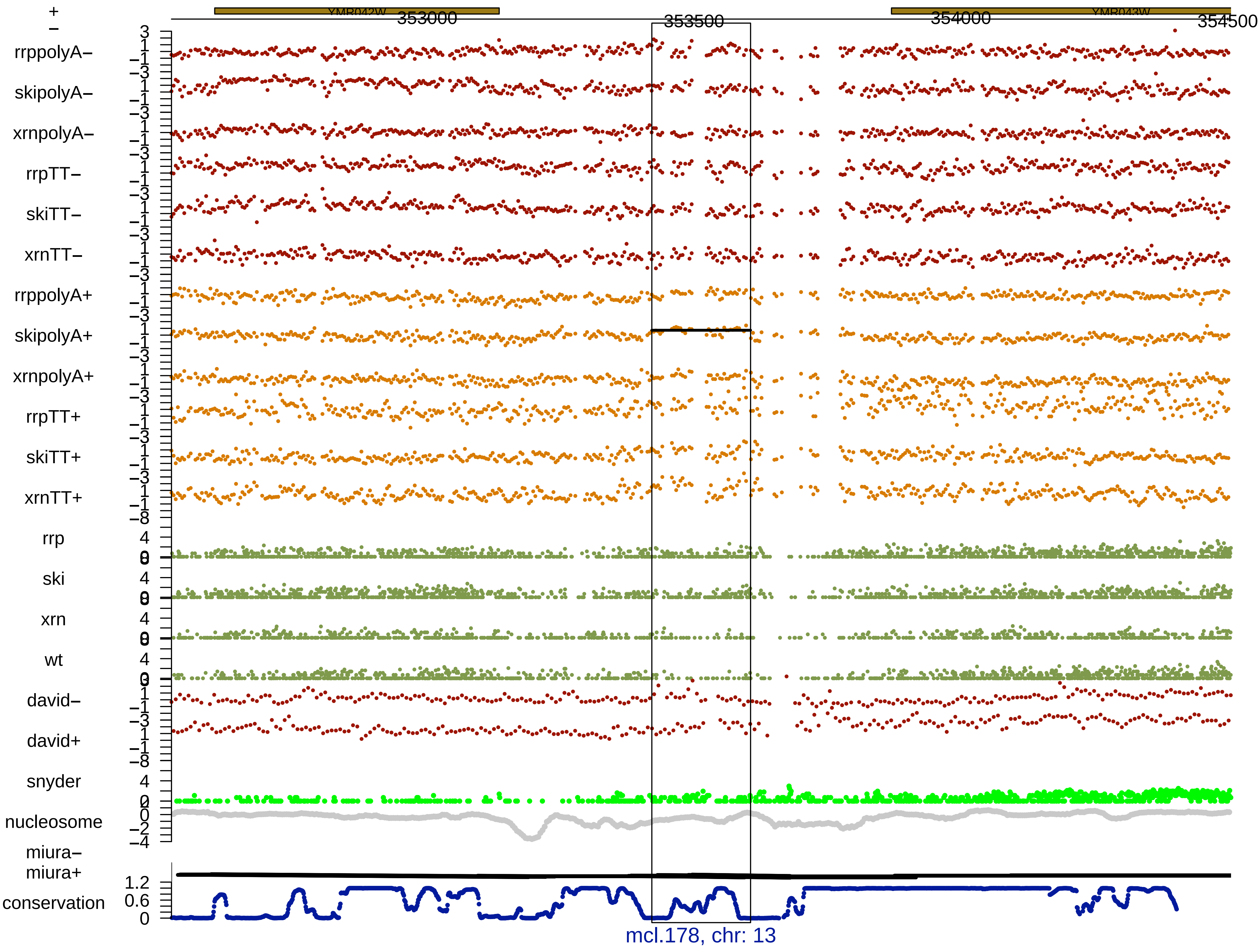

YMR042W
YMR043W
353000
353500
354000
354500
rrppolyA−
skipolyA−
xrnpolyA−
rrpTT−
skiTT−
xrnTT−
rrppolyA+
skipolyA+
xrnpolyA+
rrpTT+
skiTT+
xrnTT+
rrp
ski
xrn
wt
david−
david+
snyder
nucleosome
miura−
miura+
conservation
mcl.178, chr: 13

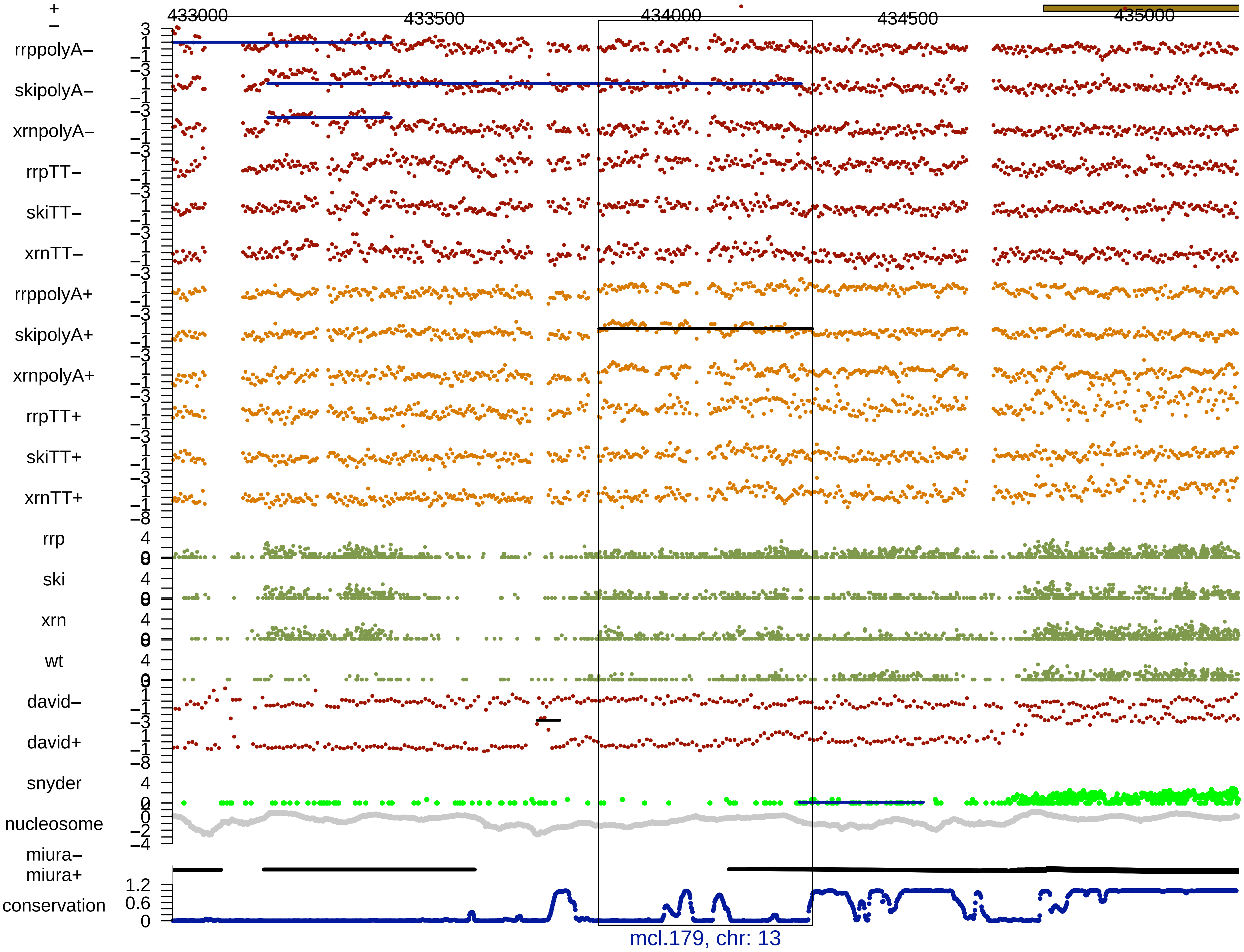

433000
433500
434000
434500
435000
rrppolyA−
skipolyA−
xrnpolyA−
rrpTT−
skiTT−
xrnTT−
rrppolyA+
skipolyA+
xrnpolyA+
rrpTT+
skiTT+
xrnTT+
rrp
ski
xrn
wt
david−
david+
snyder
nucleosome
miura−
miura+
conservation
mcl.179, chr: 13

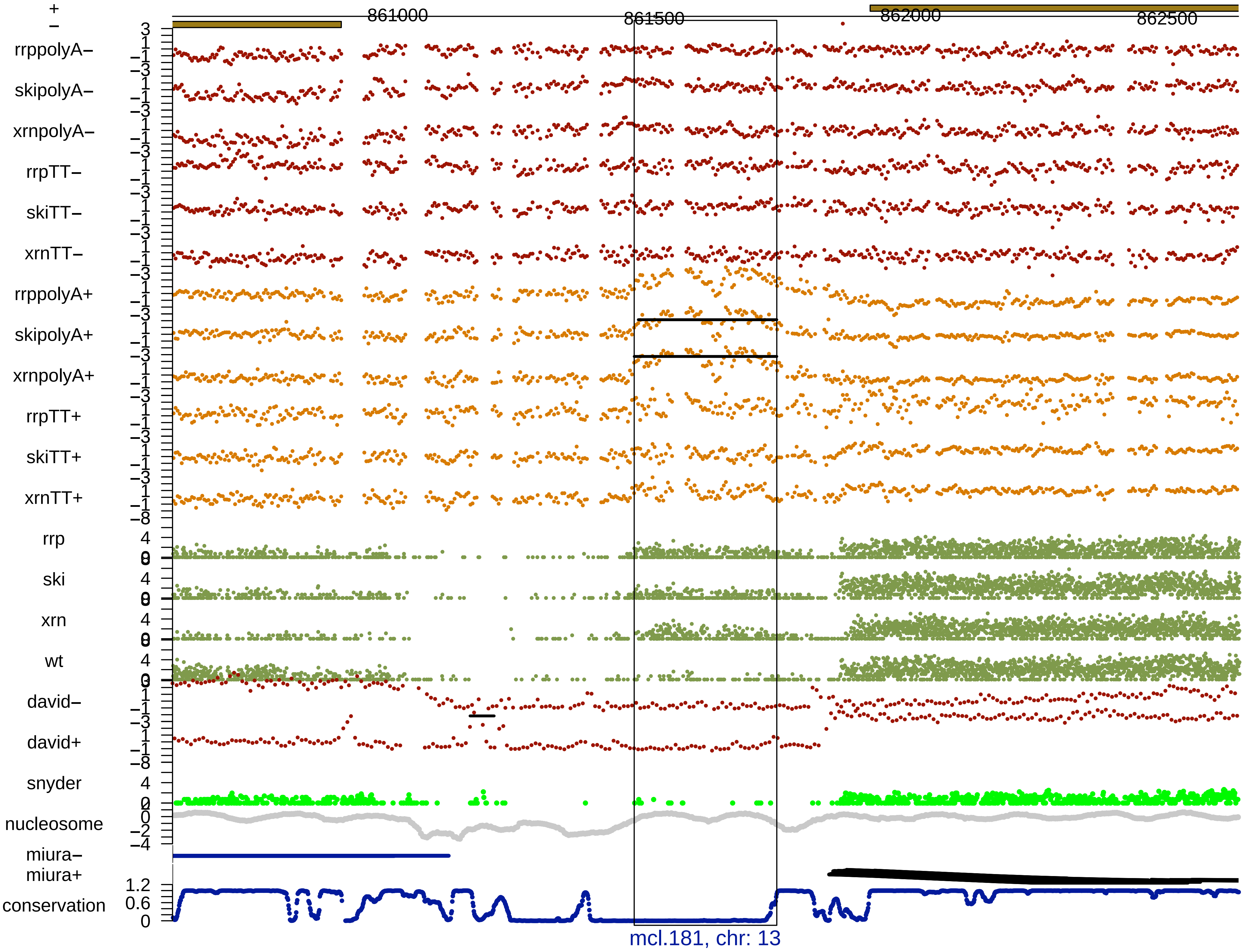

861000
861500
862000
862500
rrppolyA−
skipolyA−
xrnpolyA−
rrpTT−
skiTT−
xrnTT−
rrppolyA+
skipolyA+
xrnpolyA+
rrpTT+
skiTT+
xrnTT+
rrp
ski
xrn
wt
david−
david+
snyder
nucleosome
miura−
miura+
conservation
mcl.181, chr: 13

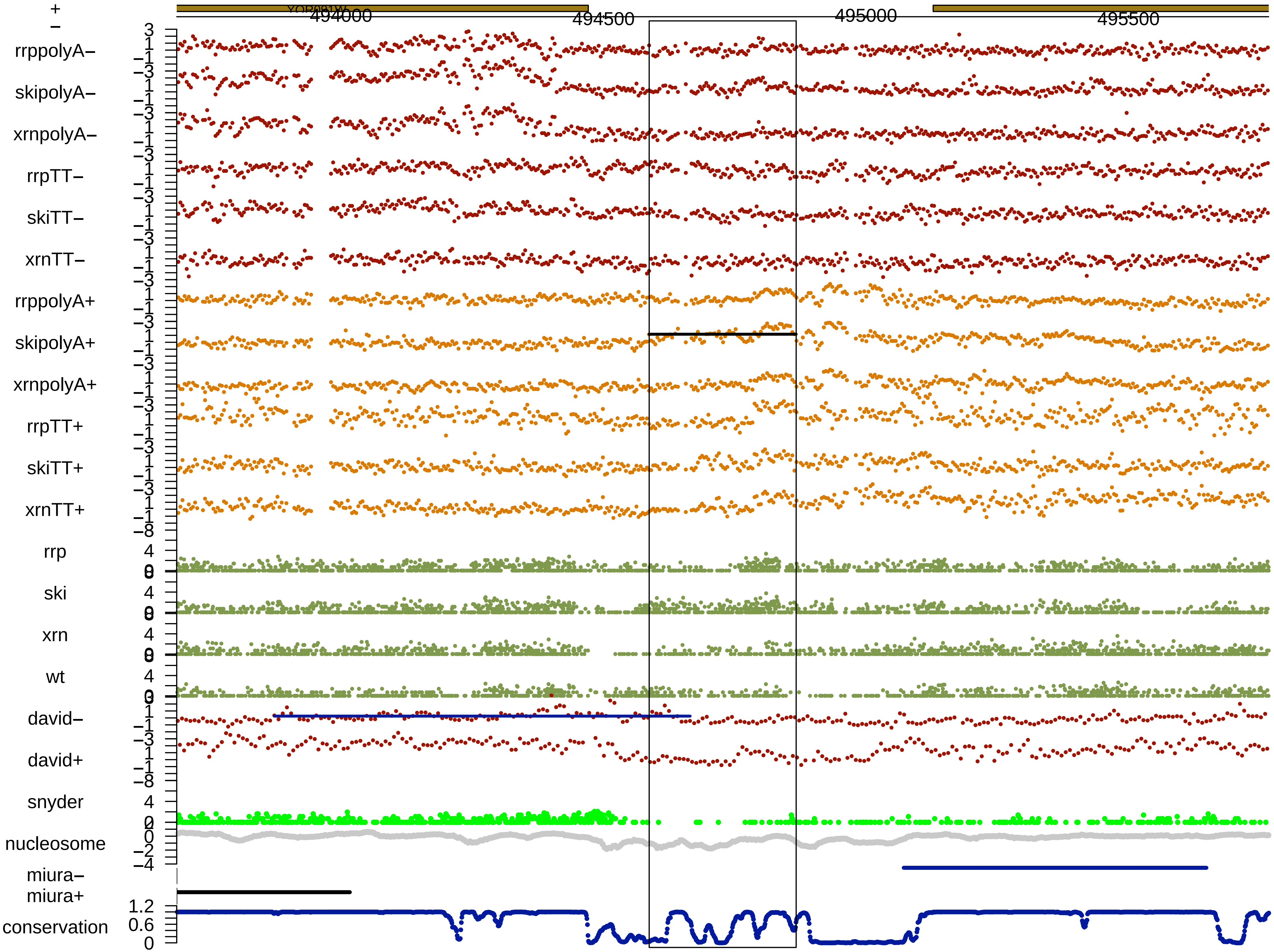

mcl.183, chr: 15

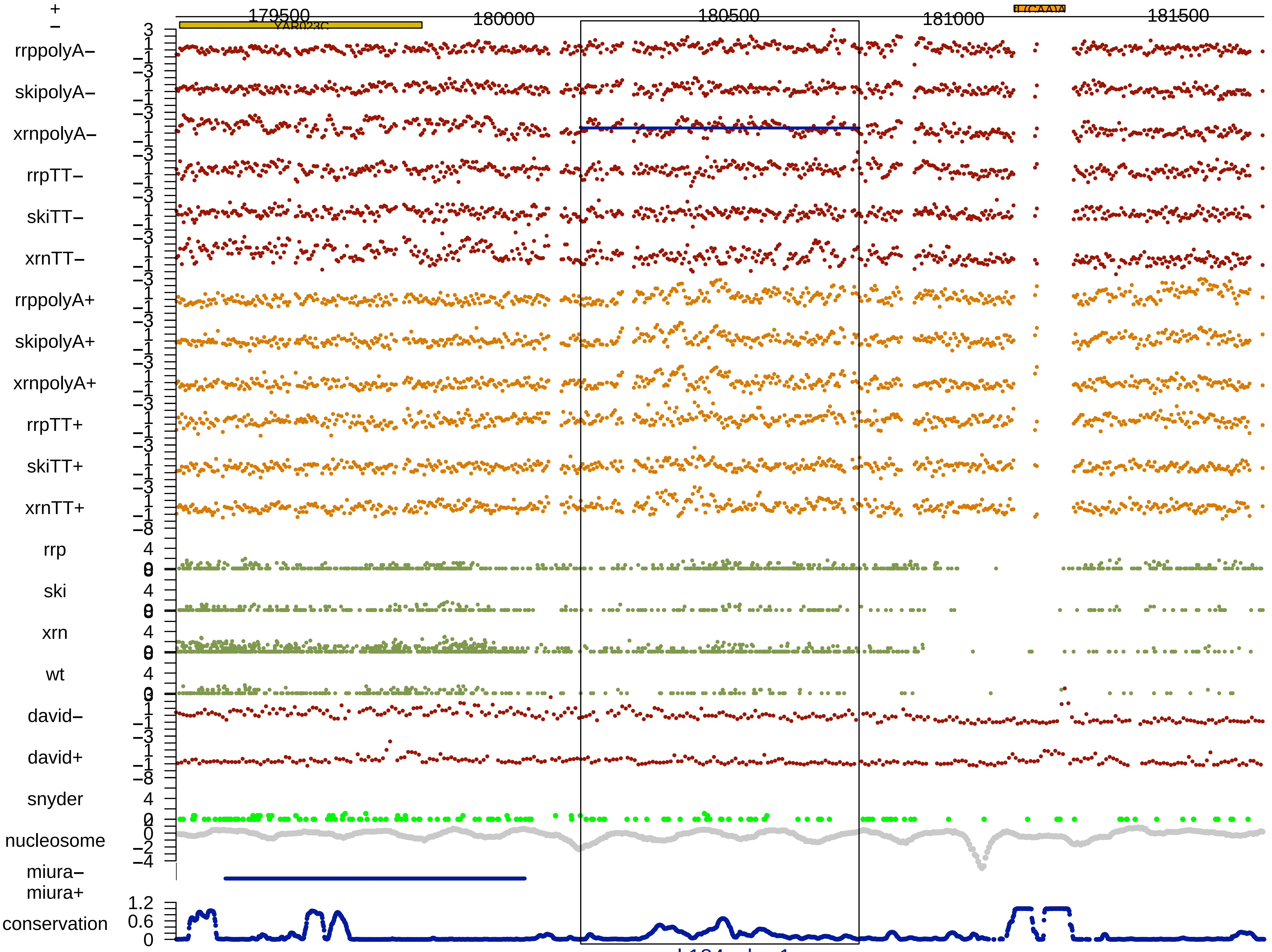

+
−
179500
180000
180500
181000
181500
YAR023C
rrppolyA−
skipolyA−
xrnpolyA−
rrpTT−
skiTT−
xrnTT−
rrppolyA+
skipolyA+
xrnpolyA+
rrpTT+
skiTT+
xrnTT+
rrp
ski
xrn
wt
david−
david+
snyder
nucleosome
miura−
miura+
conservation
3
1
−1
−3
−8
4
0
2
−2
−4
1.2
0.6
mcl.184, chr: 1

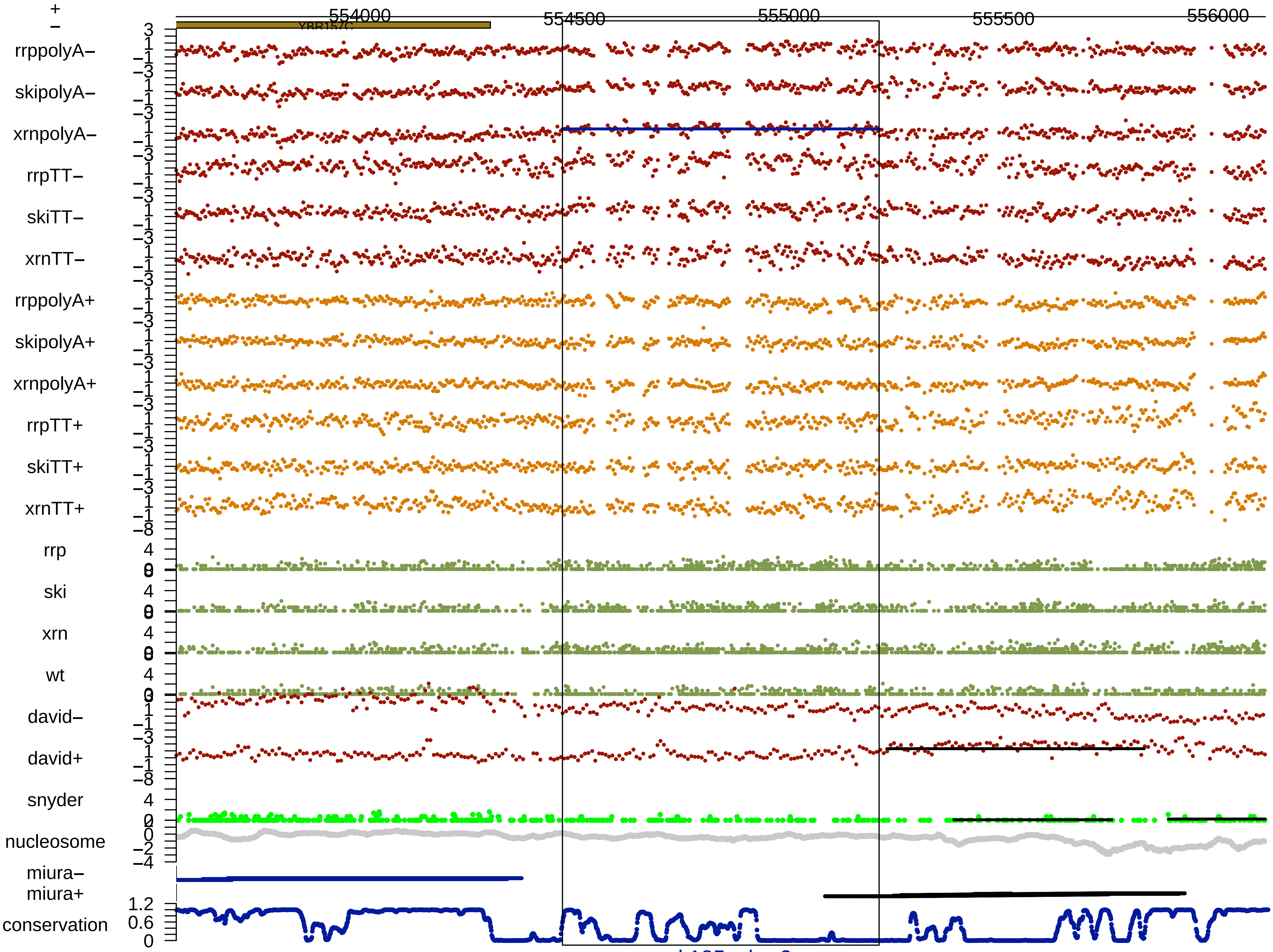
+
−
554000
554500
555000
555500
556000
rrppolyA−
skipolyA−
xrnpolyA−
rrpTT−
skiTT−
xrnTT−
rrppolyA+
skipolyA+
xrnpolyA+
rrpTT+
skiTT+
xrnTT+
rrp
ski
xrn
wt
david−
david+
snyder
nucleosome
miura−
miura+
conservation

mcl.185, chr: 2

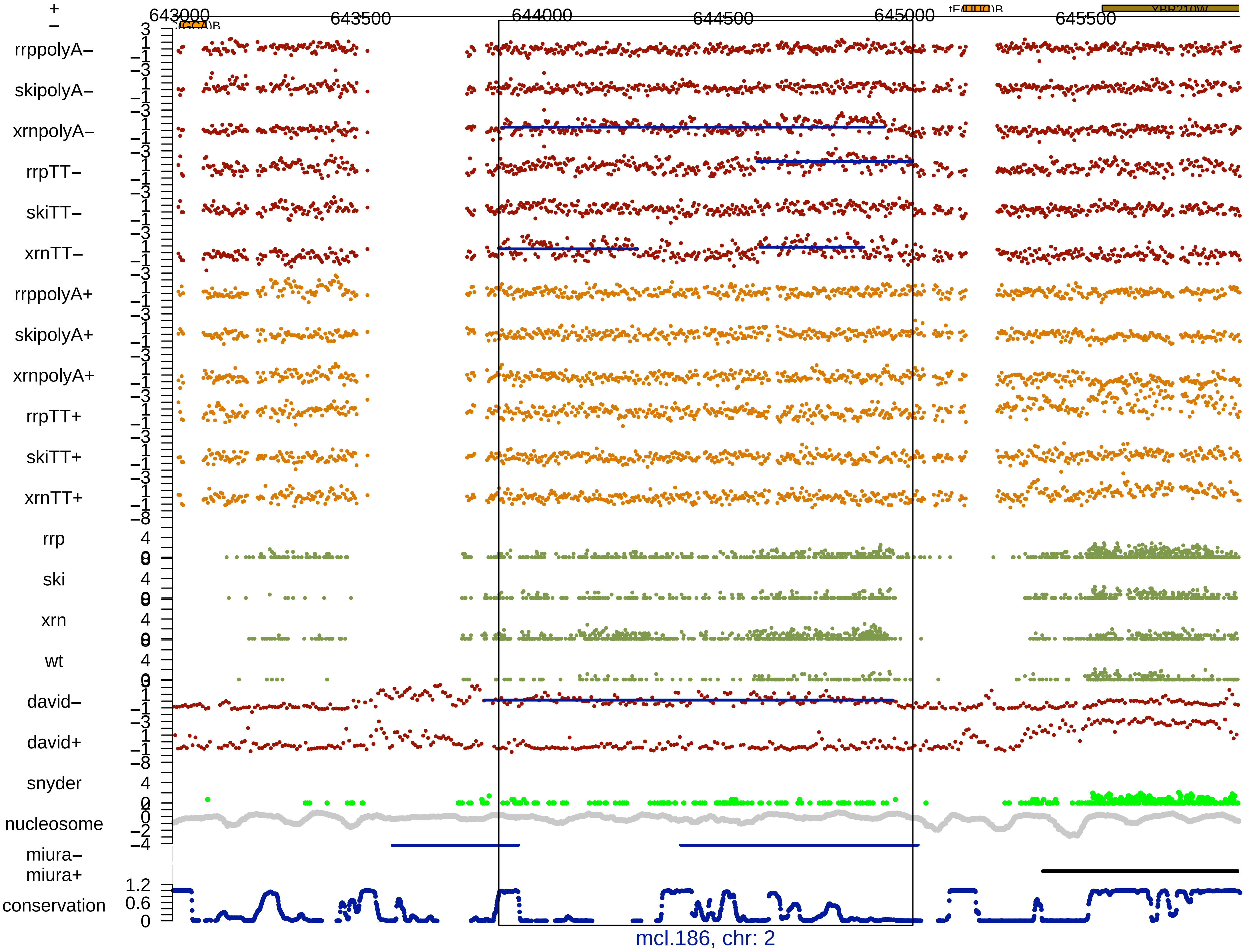

643000
643500
644000
644500
645000
645500
rrppolyA−
skipolyA−
xrnpolyA−
rrpTT−
skiTT−
xrnTT−
rrppolyA+
skipolyA+
xrnpolyA+
rrpTT+
skiTT+
xrnTT+
rrp
ski
xrn
wt
david−
david+
snyder
nucleosome
miura−
miura+
conservation
YBR210W
mcl.186, chr: 2

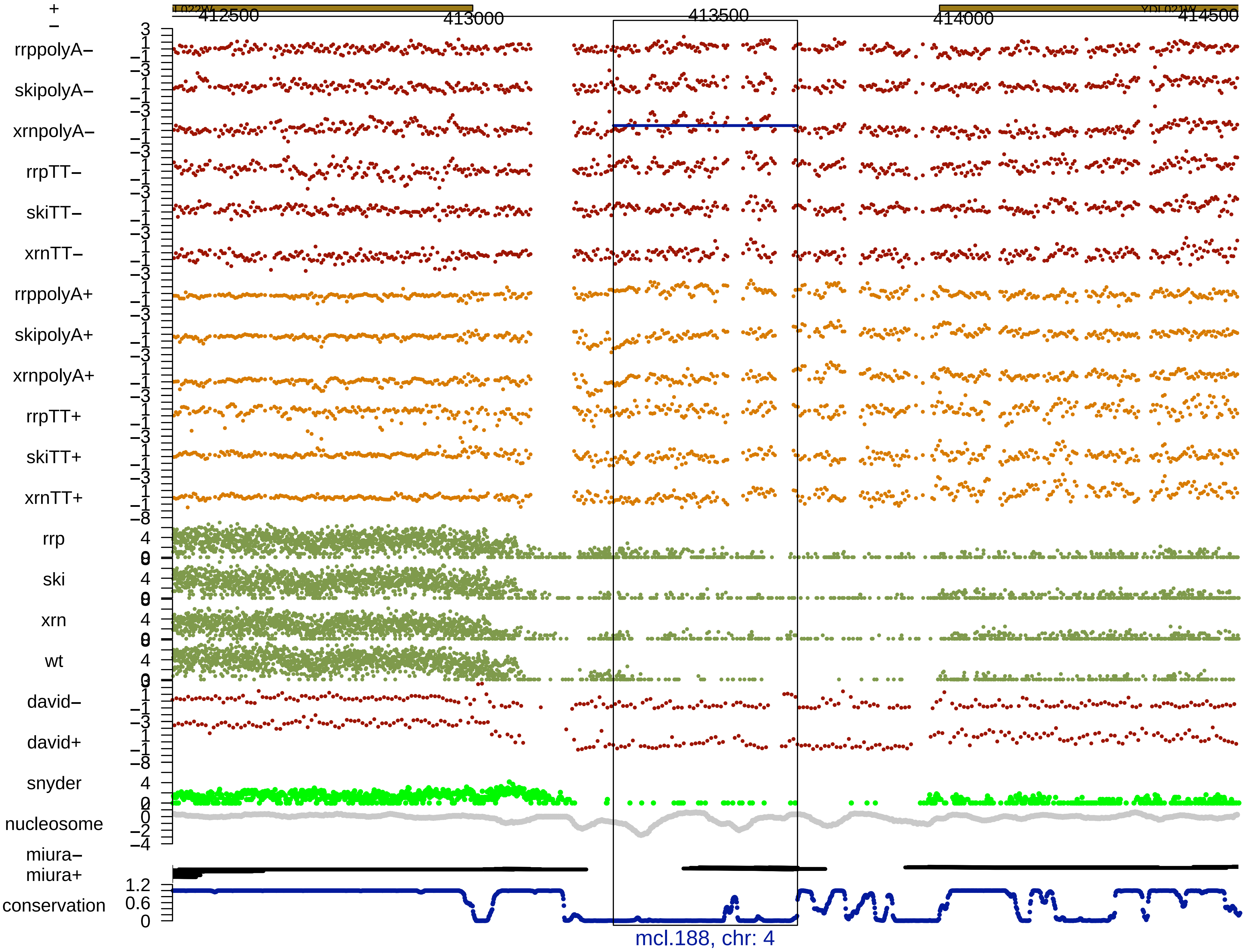

+
−
412500
413000
413500
414000
414500
rrppolyA−
skipolyA−
xrnpolyA−
rrpTT−
skiTT−
xrnTT−
rrppolyA+
skipolyA+
xrnpolyA+
rrpTT+
skiTT+
xrnTT+
rrp
ski
xrn
wt
david−
david+
snyder
nucleosome
miura−
miura+
conservation
mcl.188, chr: 4

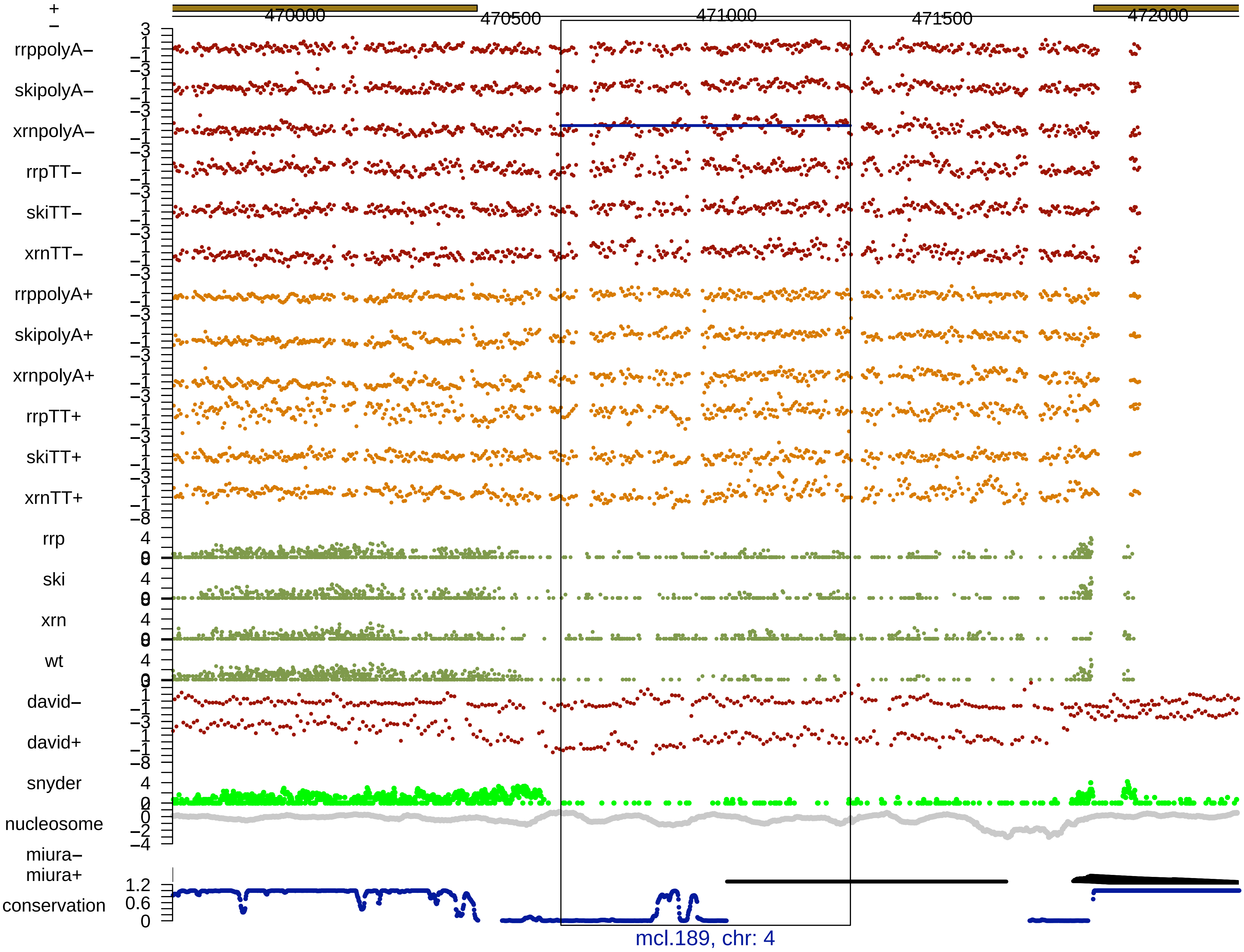

+
−
470000
470500
471000
471500
472000
rrppolyA−
skipolyA−
xrnpolyA−
rrpTT−
skiTT−
xrnTT−
rrppolyA+
skipolyA+
xrnpolyA+
rrpTT+
skiTT+
xrnTT+
rrp
ski
xrn
wt
david−
david+
snyder
nucleosome
miura−
miura+
conservation
mcl.189, chr: 4

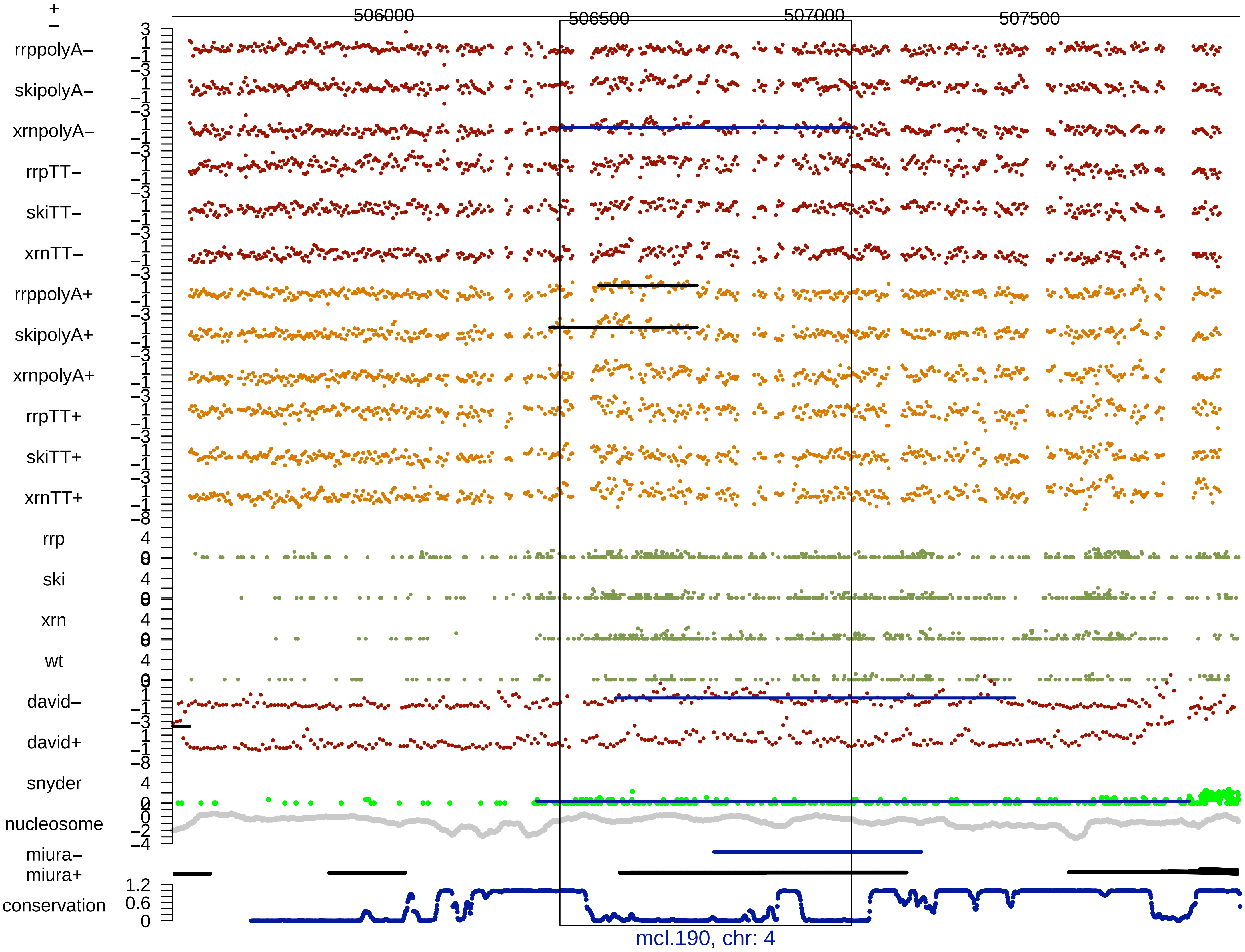

506000
506500
507000
507500
rrppolyA−
skipolyA−
xrnpolyA−
rrpTT−
skiTT−
xrnTT−
rrppolyA+
skipolyA+
xrnpolyA+
rrpTT+
skiTT+
xrnTT+
rrp
ski
xrn
wt
david−
david+
snyder
nucleosome
miura−
miura+
conservation
mcl.190, chr: 4

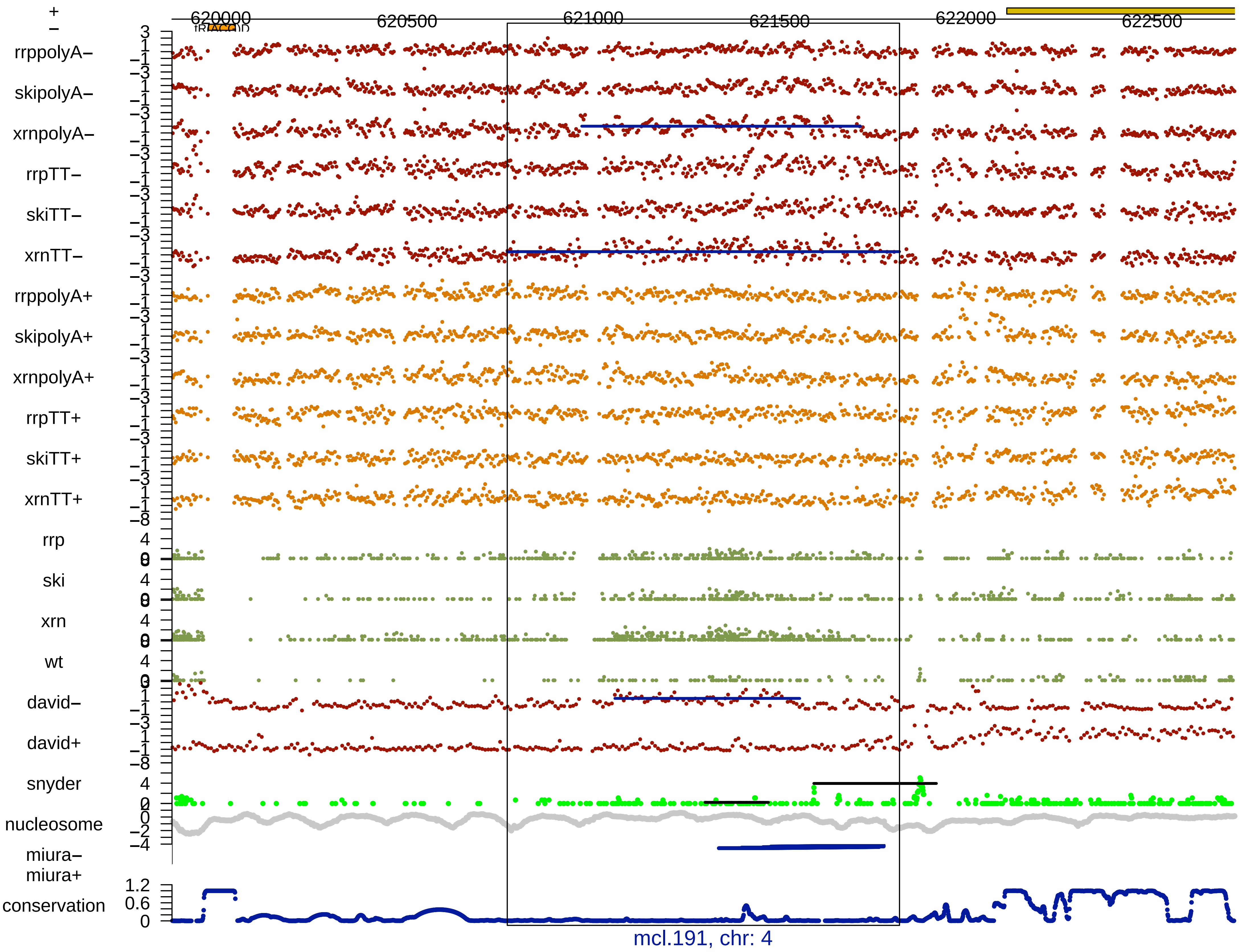
620000
620500
621000
621500
622000
622500
rrppolyA−
skipolyA−
xrnpolyA−
rrpTT−
skiTT−
xrnTT−
rrppolyA+
skipolyA+
xrnpolyA+
rrpTT+
skiTT+
xrnTT+
rrp
ski
xrn
wt
david−
david+
snyder
nucleosome
miura−
miura+
conservation
mcl.191, chr: 4

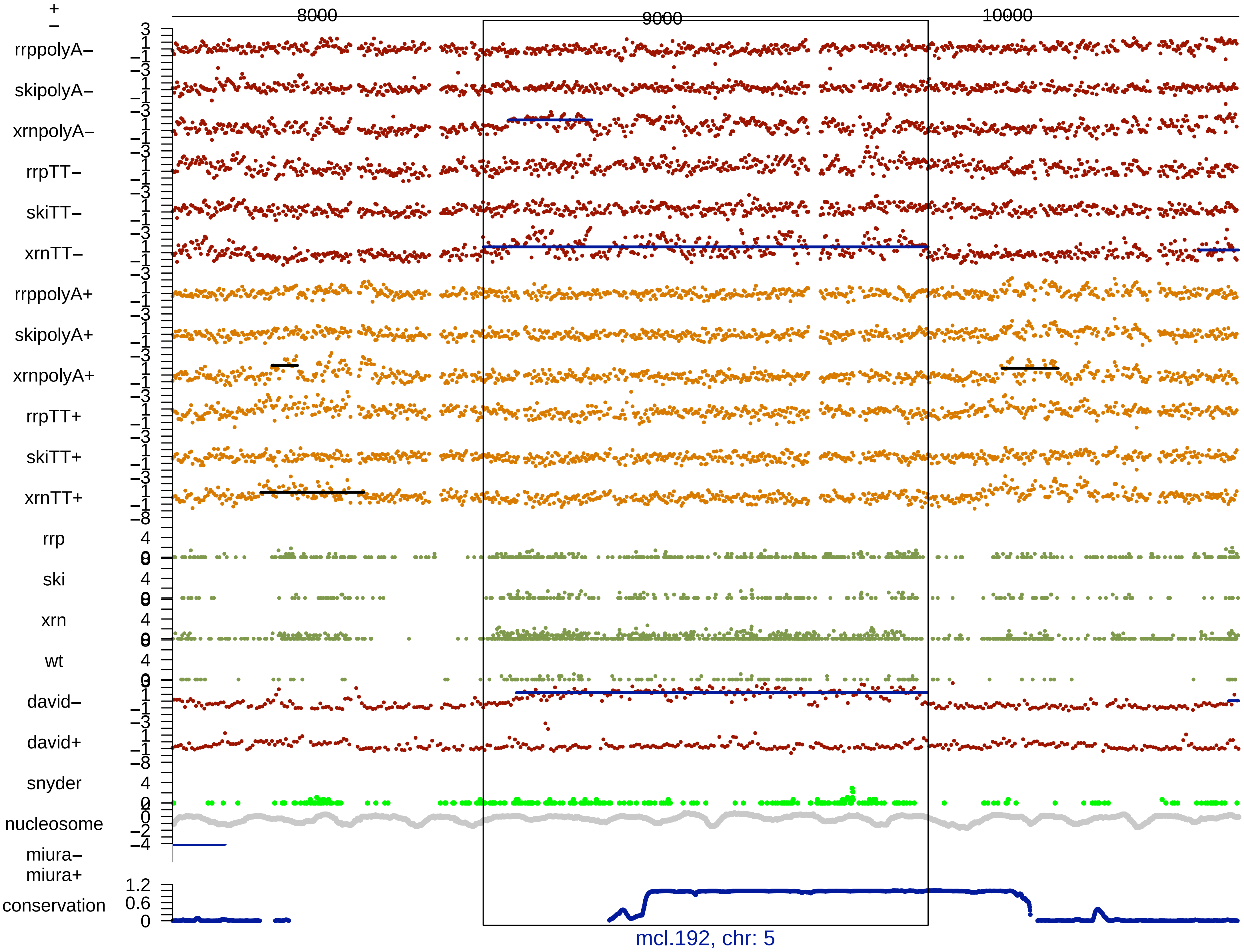

8000
9000
10000
rrppolyA−
skipolyA−
xrnpolyA−
rrpTT−
skiTT−
xrnTT−
rrppolyA+
skipolyA+
xrnpolyA+
rrpTT+
skiTT+
xrnTT+
rrp
ski
xrn
wt
david−
david+
snyder
nucleosome
miura−
miura+
conservation
mcl.192, chr: 5

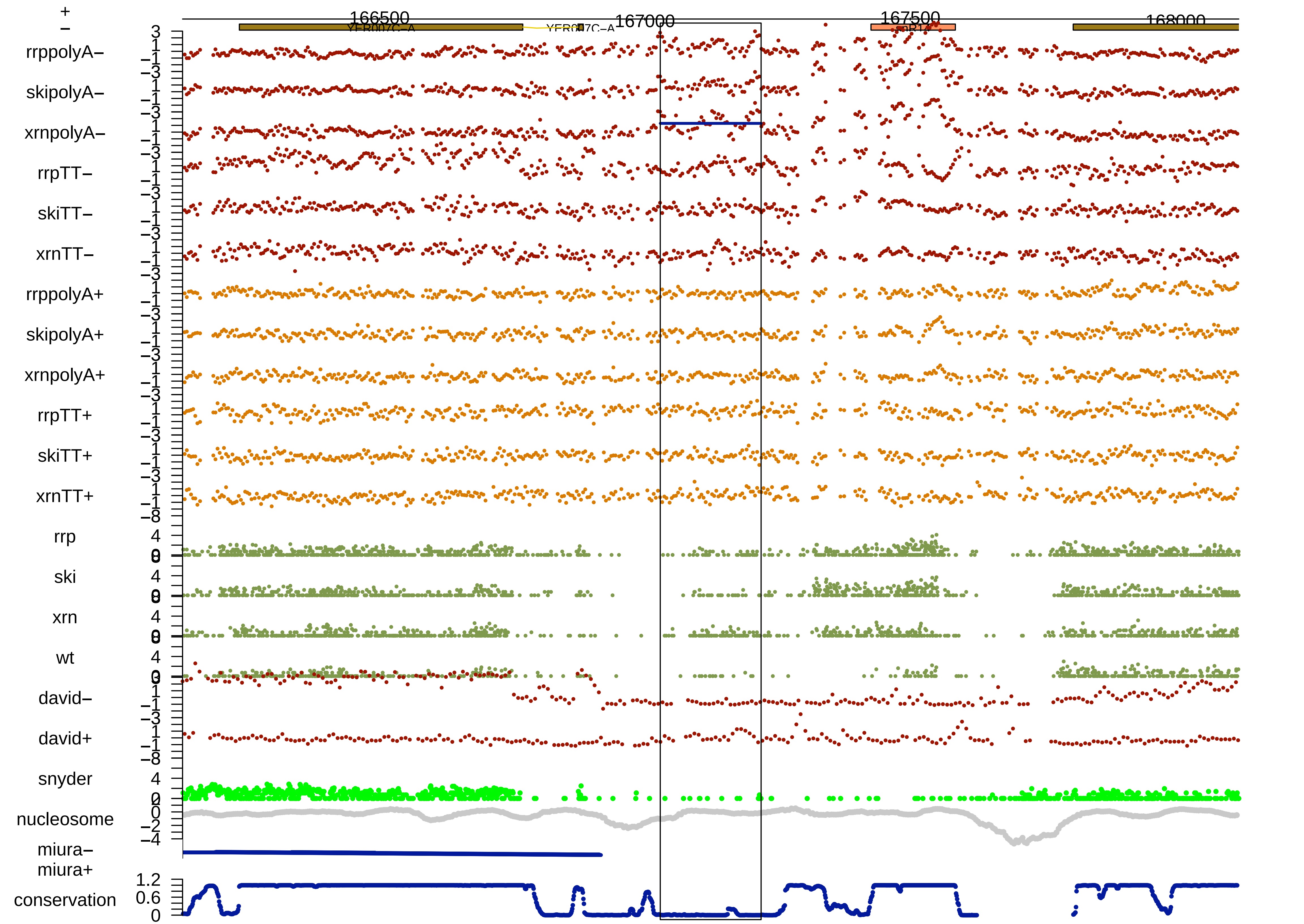

166500
167000
167500
rrppolyA–
skipolyA–
xrnpolyA–
rrpTT–
skiTT–
xrnTT–
rrppolyA+
skipolyA+
xrnpolyA+
rrpTT+
skiTT+
xrnTT+
rrp
ski
xrn
wt
david–
david+
snyder
nucleosome
miura–
miura+
conservation

mcl.193, chr: 5

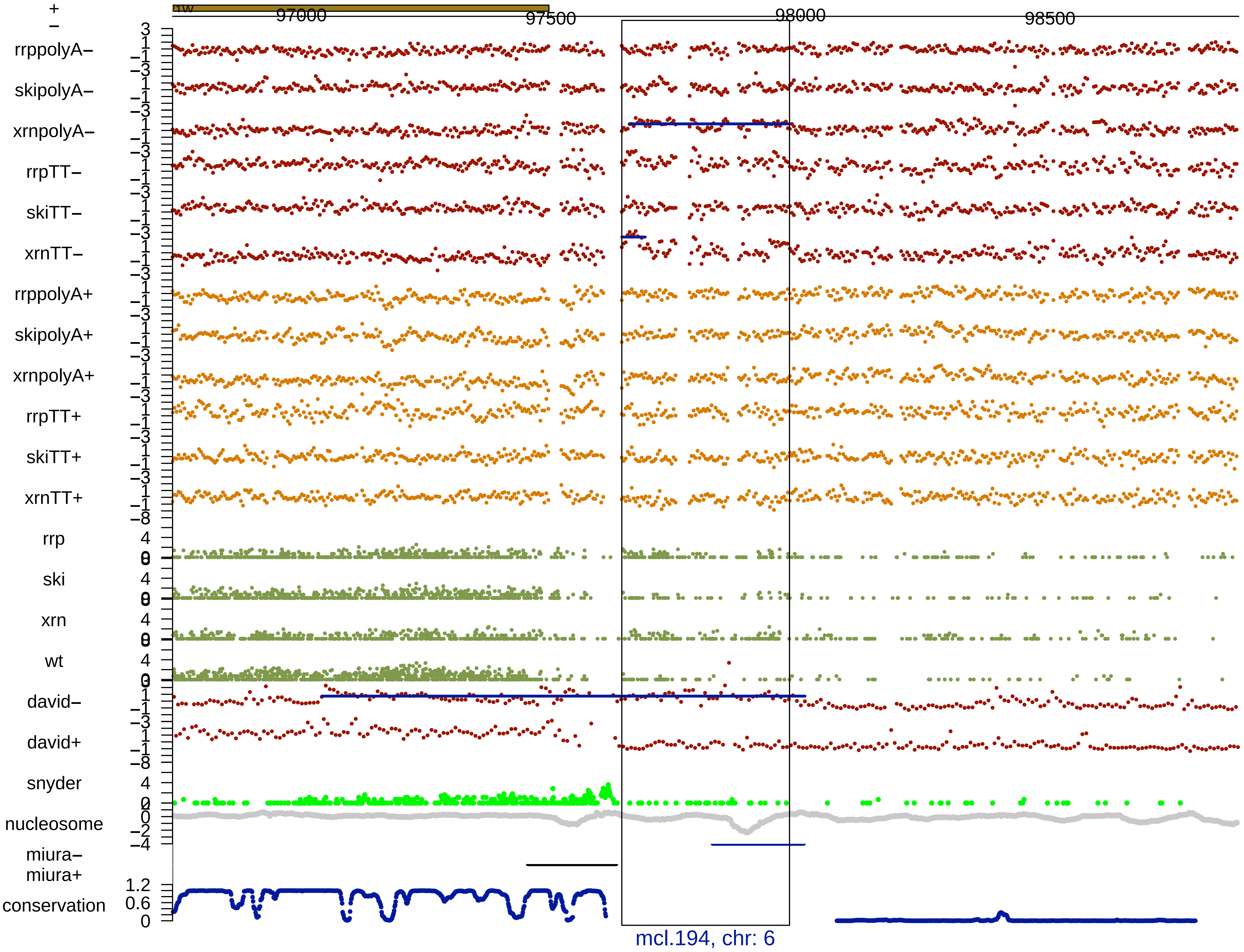

+
−
97000
97500
98000
98500
rrppolyA−
skipolyA−
xrnpolyA−
rrpTT−
skiTT−
xrnTT−
rrppolyA+
skipolyA+
xrnpolyA+
rrpTT+
skiTT+
xrnTT+
rrp
ski
xrn
wt
david−
david+
snyder
nucleosome
miura−
miura+
conservation
mcl.194, chr: 6

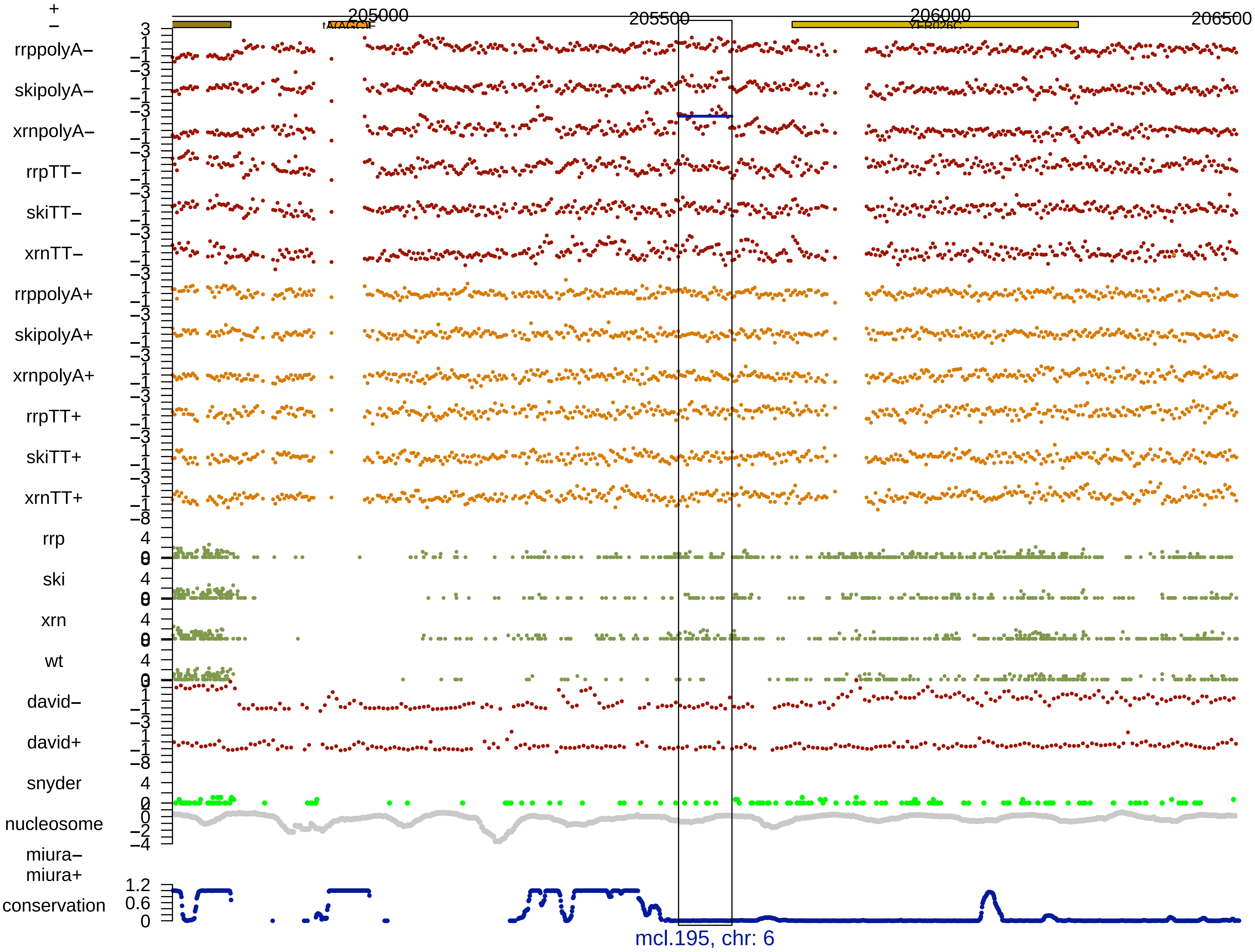

+
−
205000
205500
206000
206500
tA(AGC)F
YER026C
rrppolyA−
skipolyA−
xrnpolyA−
rrpTT−
skiTT−
xrnTT−
rrppolyA+
skipolyA+
xrnpolyA+
rrpTT+
skiTT+
xrnTT+
rrp
ski
xrn
wt
david−
david+
snyder
nucleosome
miura−
miura+
conservation
mcl.195, chr: 6

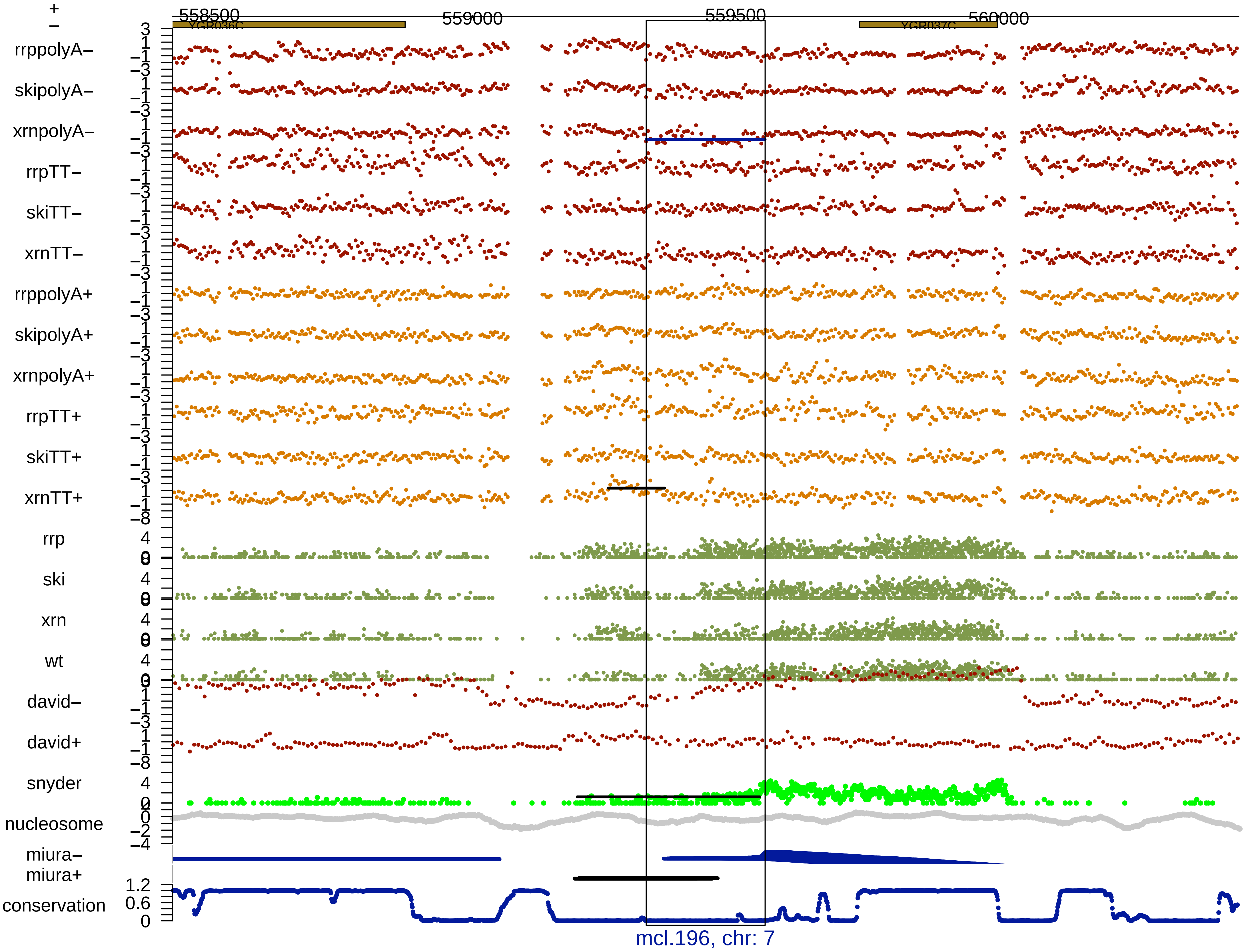

558500
559000
559500
560000
YGR036C
YGR037C
rrppolyA–
skipolyA–
xrnpolyA–
rrpTT–
skiTT–
xrnTT–
rrppolyA+
skipolyA+
xrnpolyA+
rrpTT+
skiTT+
xrnTT+
rrp
ski
xrn
wt
david–
david+
snyder
nucleosome
miura–
miura+
conservation
mcl.196, chr: 7

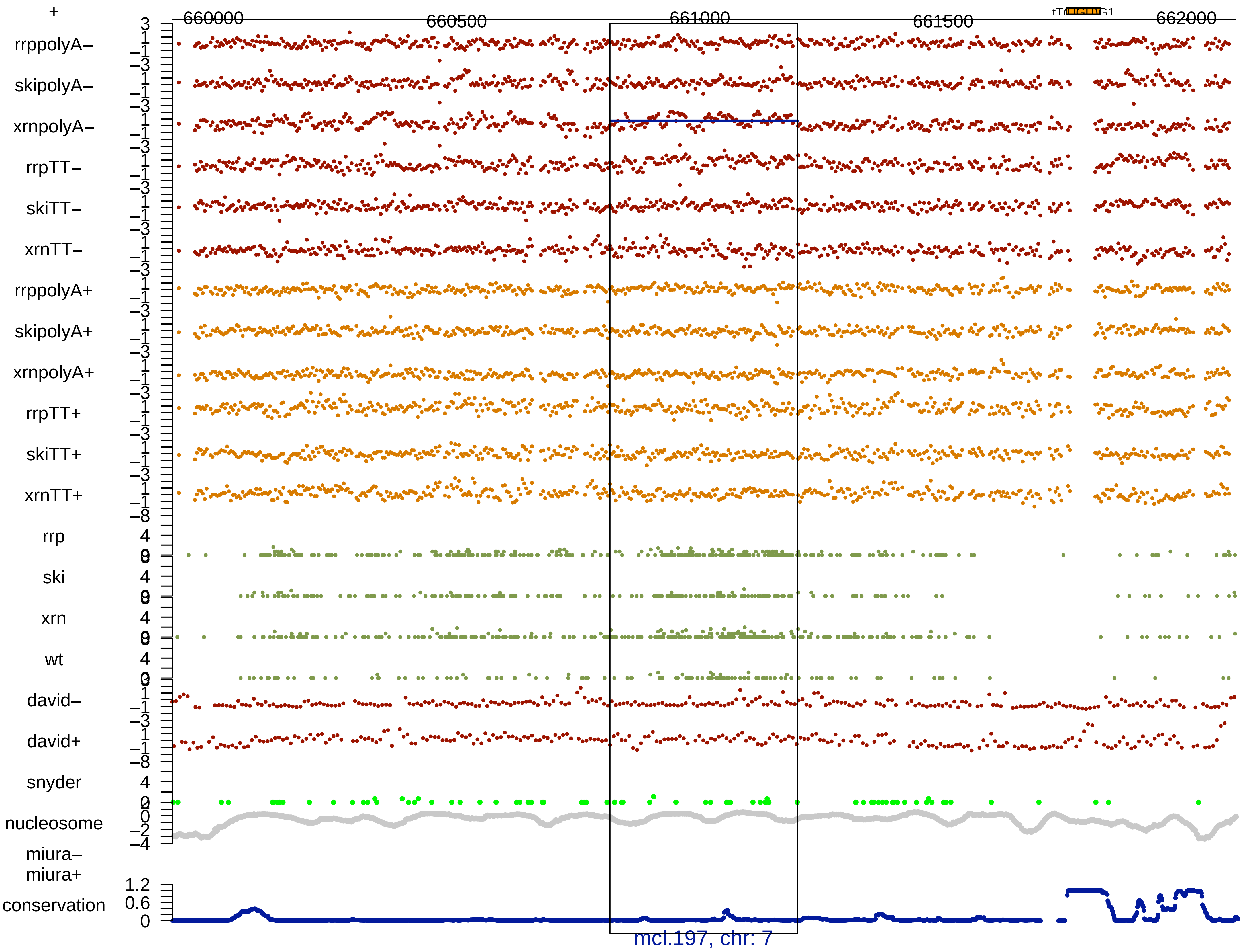
+
660000
660500
661000
661500
662000
rrppolyA−
skipolyA−
xrnpolyA−
rrpTT−
skiTT−
xrnTT−
rrppolyA+
skipolyA+
xrnpolyA+
rrpTT+
skiTT+
xrnTT+
rrp
ski
xrn
wt
david−
david+
snyder
nucleosome
miura−
miura+
conservation
mcl.197, chr: 7

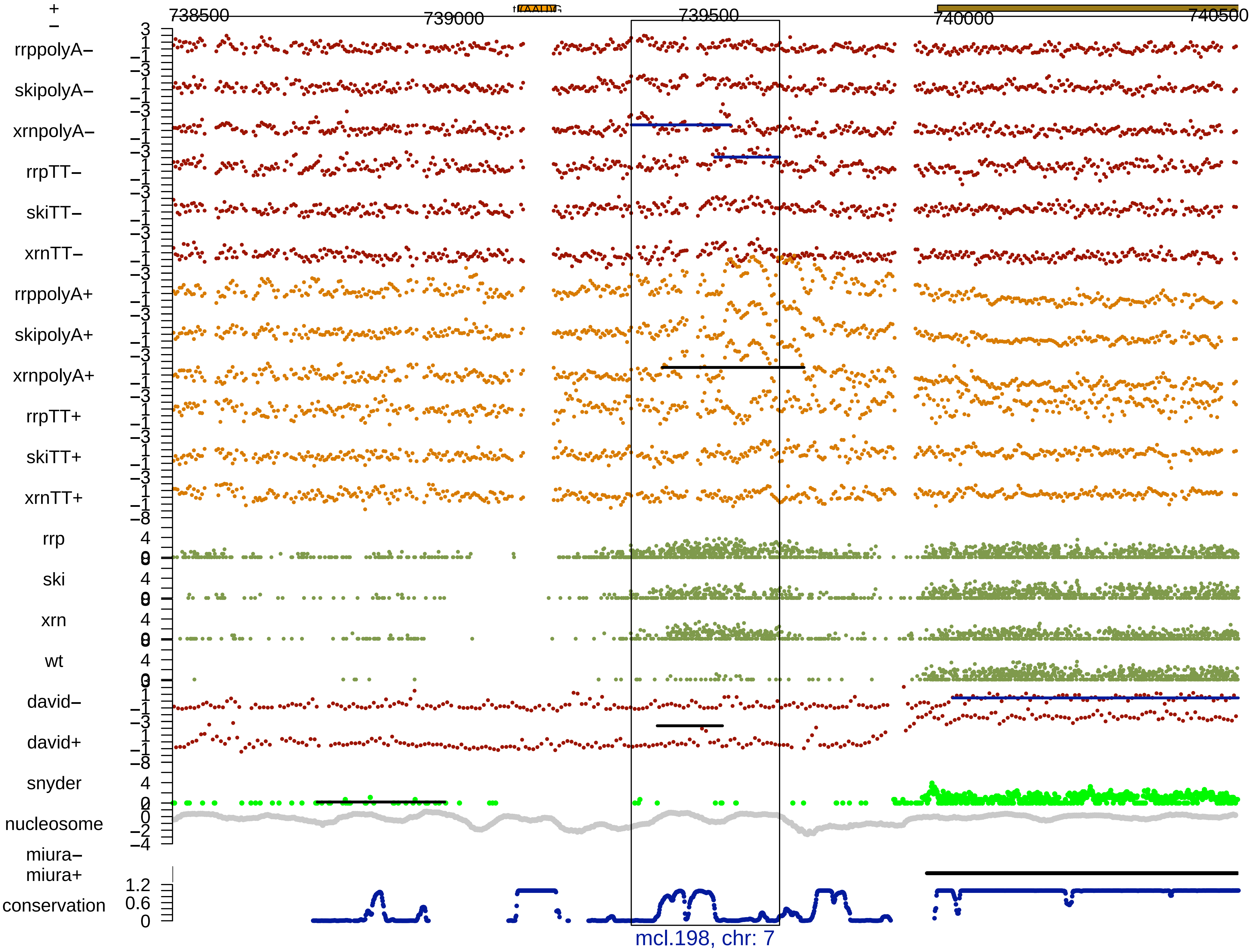

+
−
738500
739000
739500
740000
740500
rrppolyA−
skipolyA−
xrnpolyA−
rrpTT−
skiTT−
xrnTT−
rrppolyA+
skipolyA+
xrnpolyA+
rrpTT+
skiTT+
xrnTT+
rrp
ski
xrn
wt
david−
david+
snyder
nucleosome
miura−
miura+
conservation
mcl.198, chr: 7

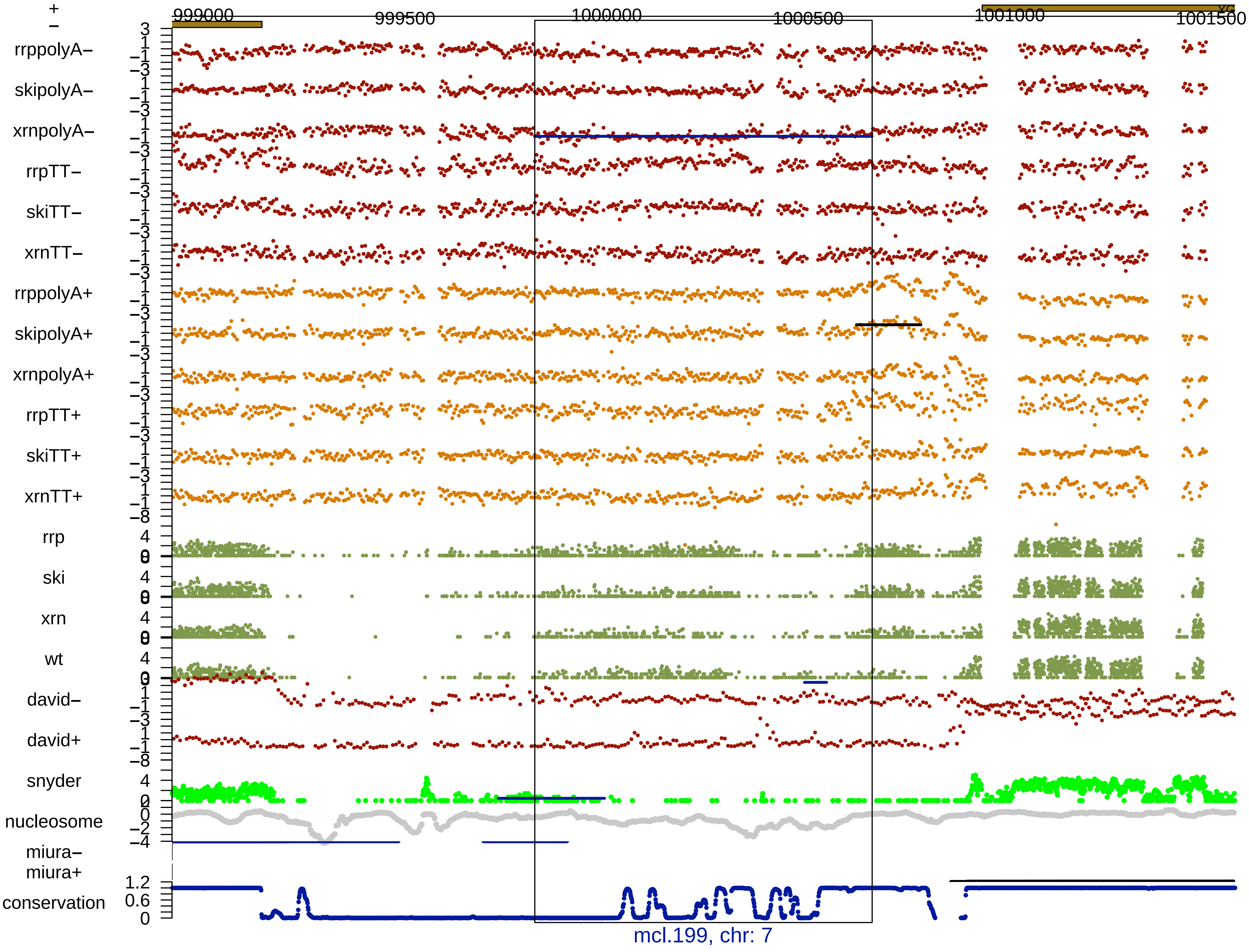
+
−
999000
999500
1000000
1000500
1001000
1001500
rrppolyA−
skipolyA−
xrnpolyA−
rrpTT−
skiTT−
xrnTT−
rrppolyA+
skipolyA+
xrnpolyA+
rrpTT+
skiTT+
xrnTT+
rrp
ski
xrn
wt
david−
david+
snyder
nucleosome
miura−
miura+
conservation
mcl.199, chr: 7

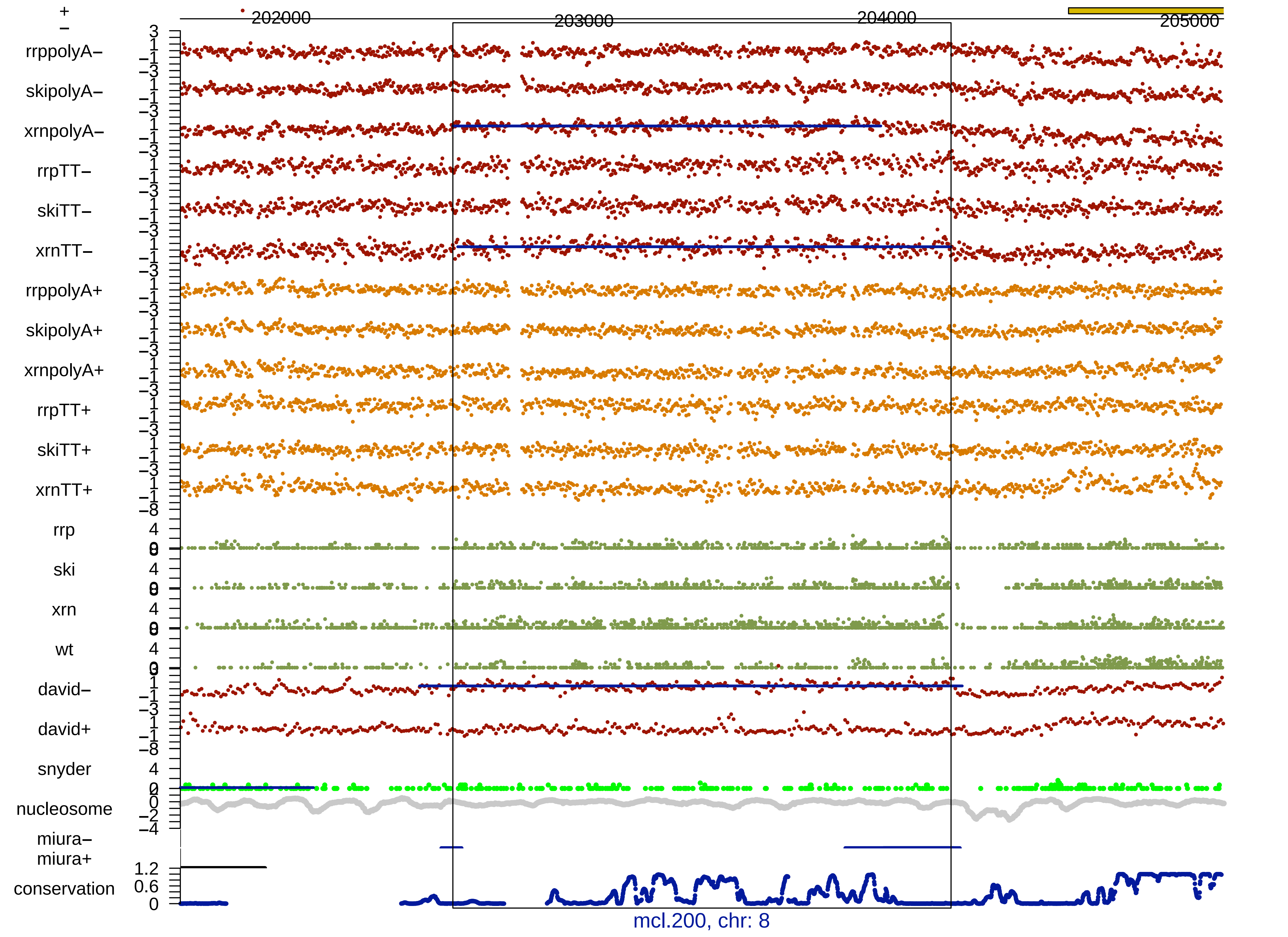

+
−
202000
203000
204000
205000
rrppolyA−
skipolyA−
xrnpolyA−
rrpTT−
skiTT−
xrnTT−
rrppolyA+
skipolyA+
xrnpolyA+
rrpTT+
skiTT+
xrnTT+
rrp
ski
xrn
wt
david−
david+
snyder
nucleosome
miura−
miura+
conservation
mcl.200, chr: 8

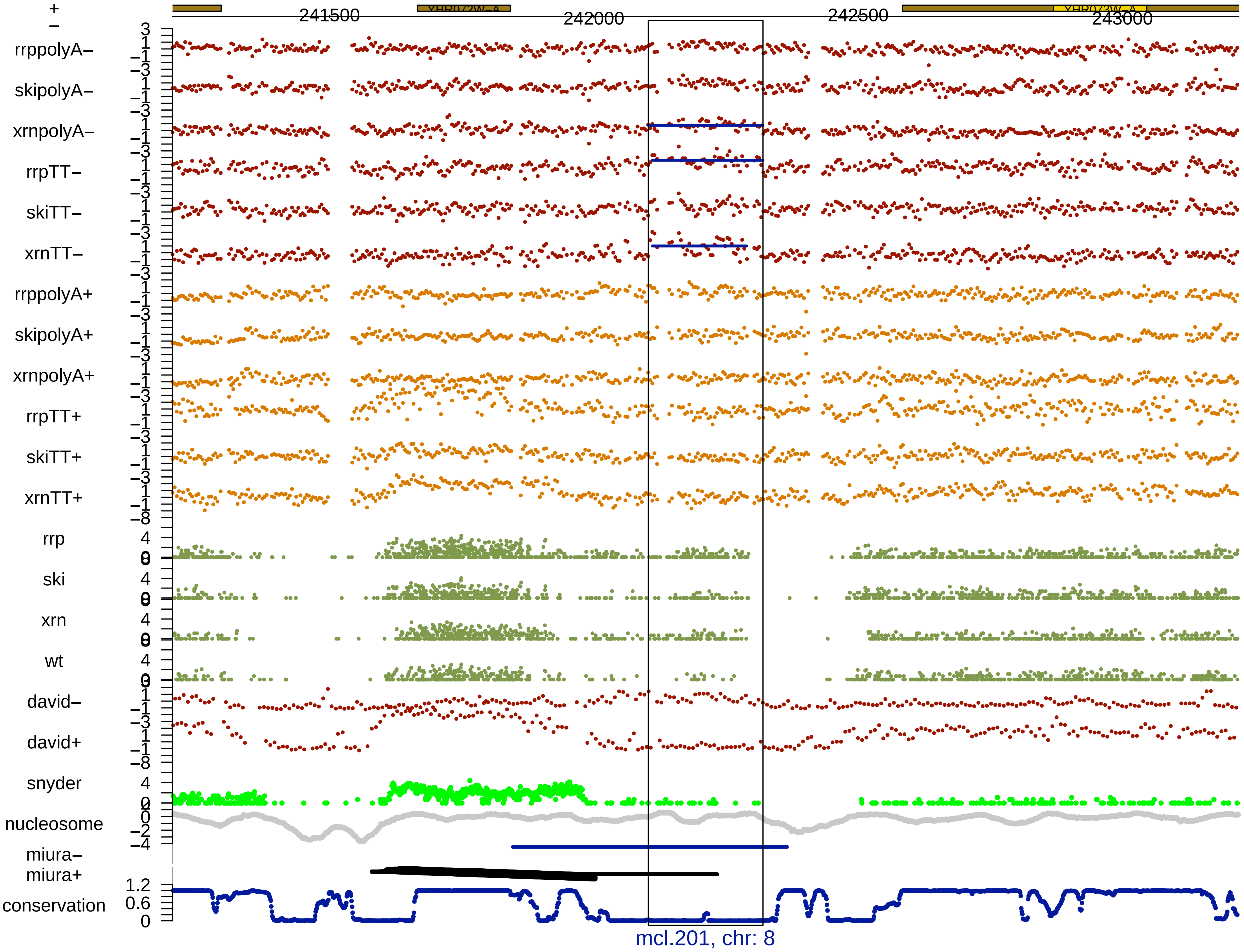
+
–
241500
YHR072W-A
242000
242500
YHR073W-A
243000
rrppolyA–
skipolyA–
xrnpolyA–
rrpTT–
skiTT–
xrnTT–
rrppolyA+
skipolyA+
xrnpolyA+
rrpTT+
skiTT+
xrnTT+
rrp
ski
xrn
wt
david–
david+
snyder
nucleosome
miura–
miura+
conservation
3
1
–1
–3
–8
4
0
2
–2
–4
1.2
0.6
mcl.201, chr: 8

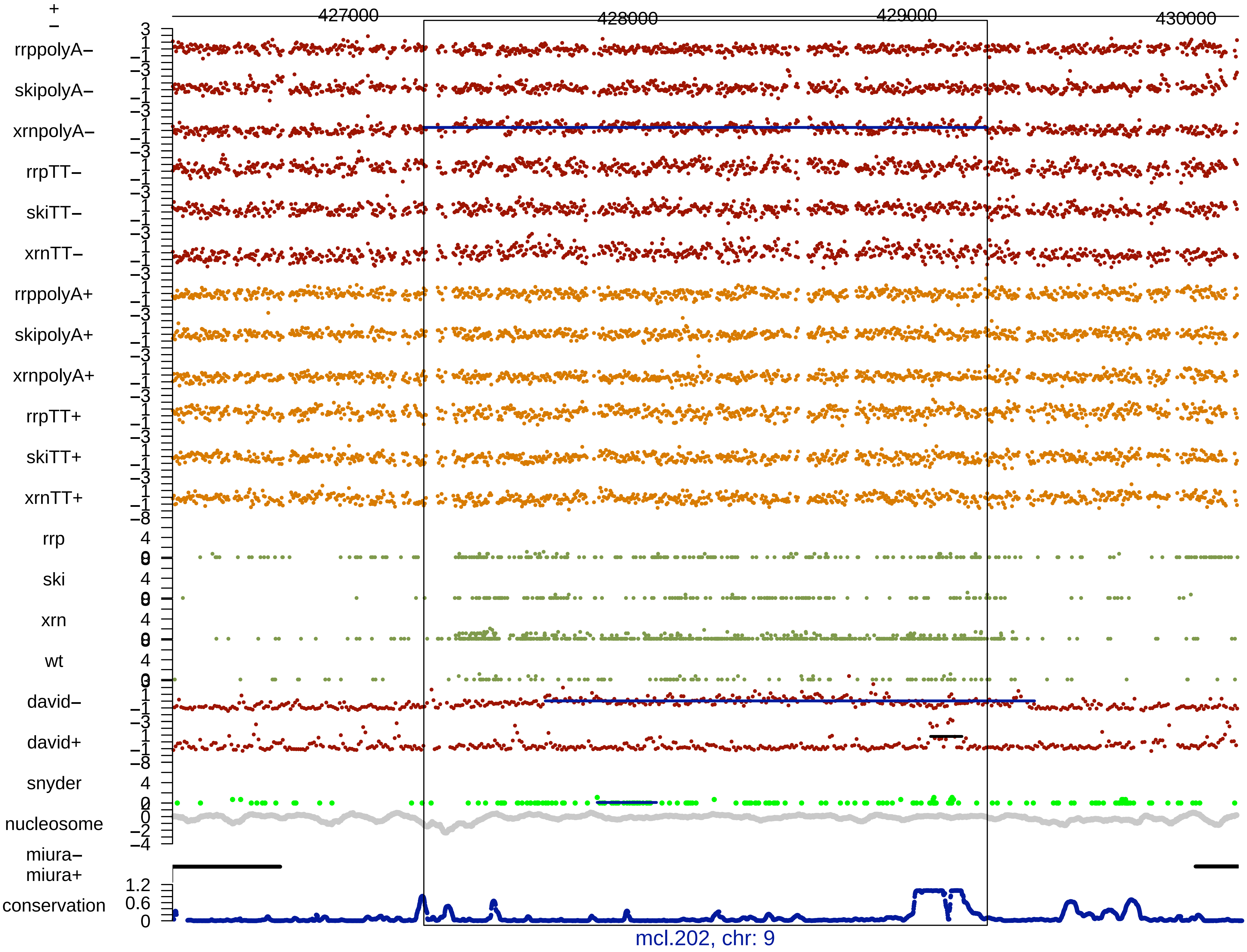

+
−
427000
428000
429000
430000
rrppolyA−
skipolyA−
xrnpolyA−
rrpTT−
skiTT−
xrnTT−
rrppolyA+
skipolyA+
xrnpolyA+
rrpTT+
skiTT+
xrnTT+
rrp
ski
xrn
wt
david−
david+
snyder
nucleosome
miura−
miura+
conservation
1.2
0.6
0
mcl.202, chr: 9

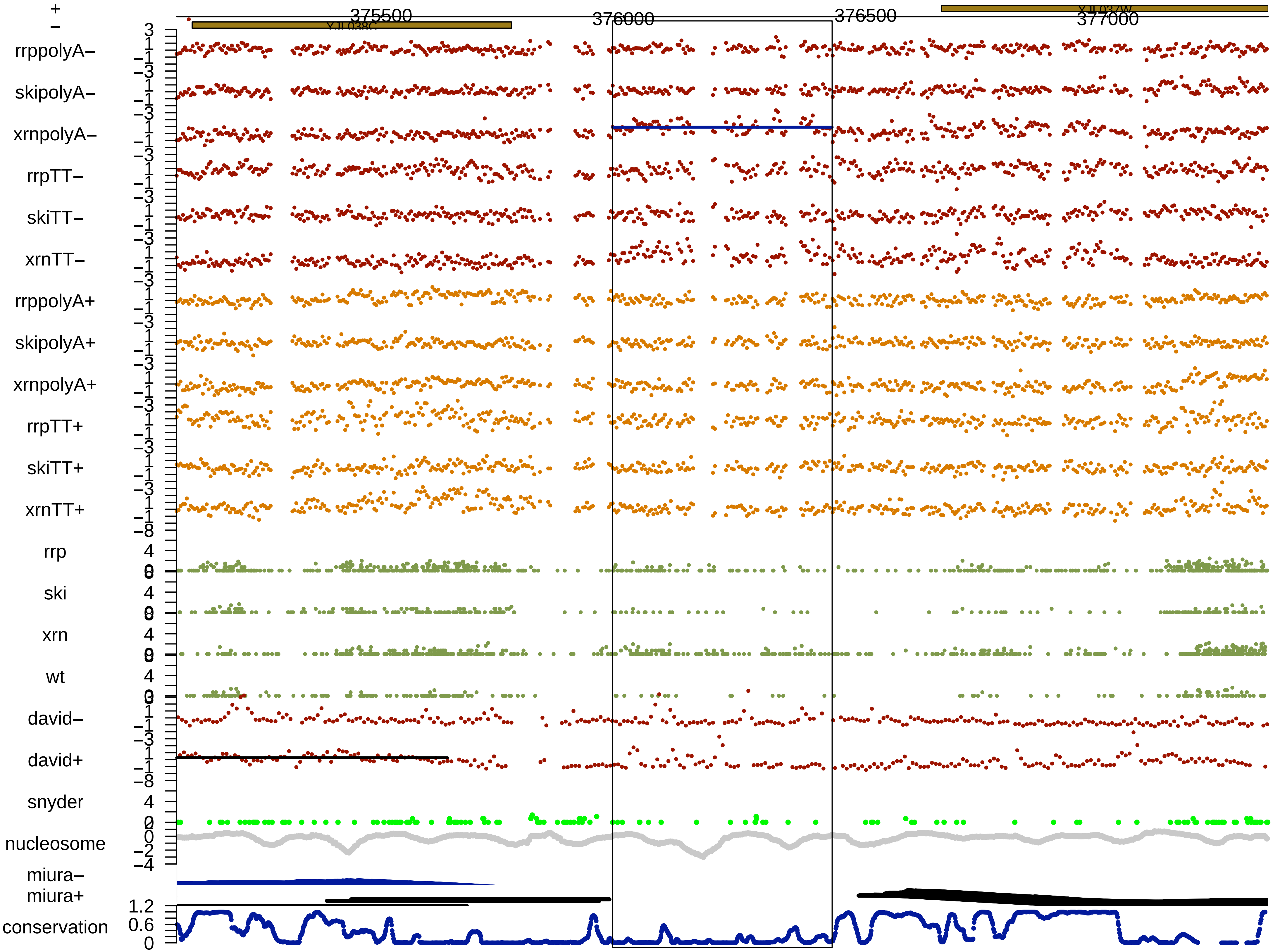

mcl.203, chr: 10

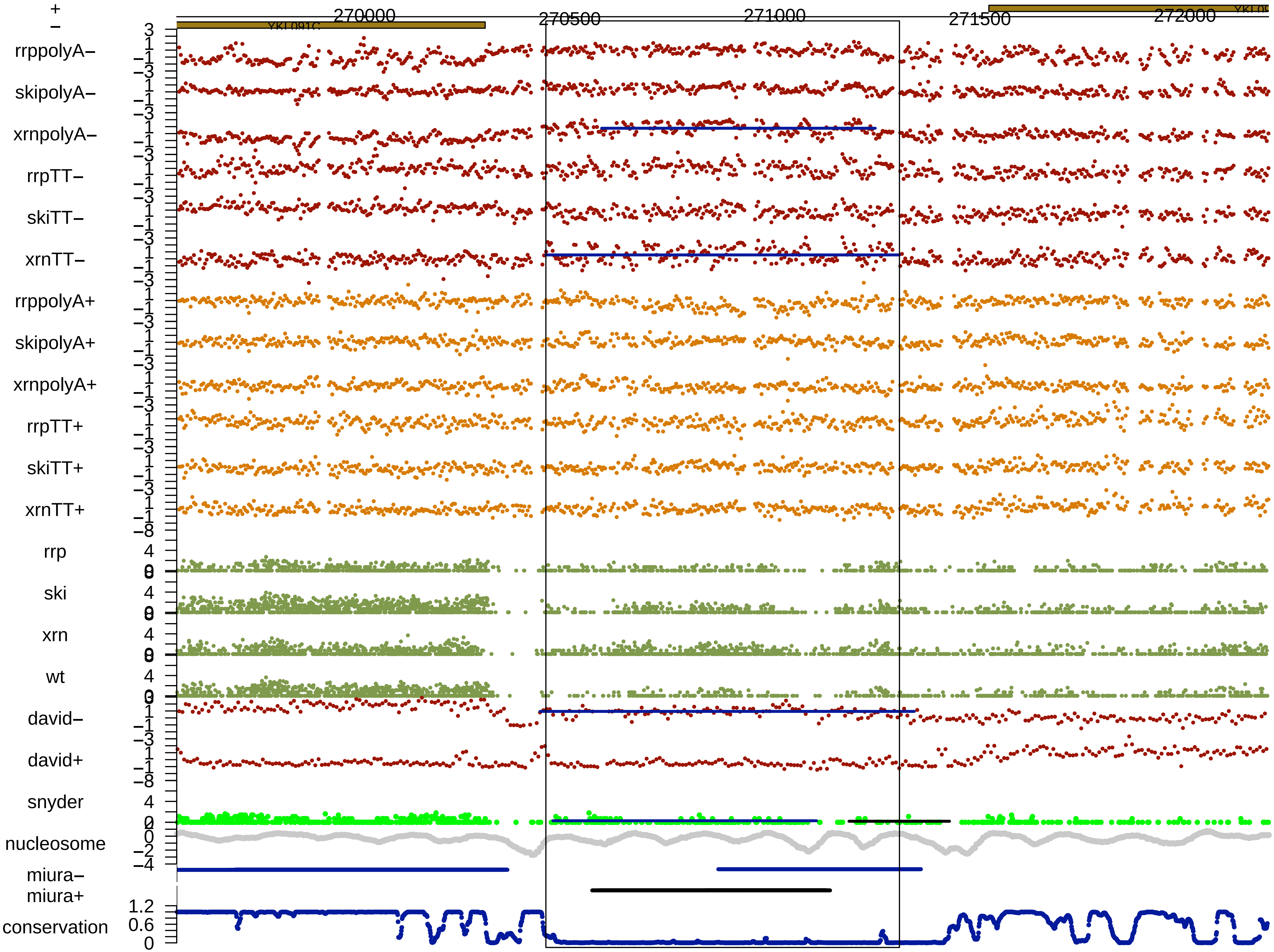

mcl.204, chr: 11

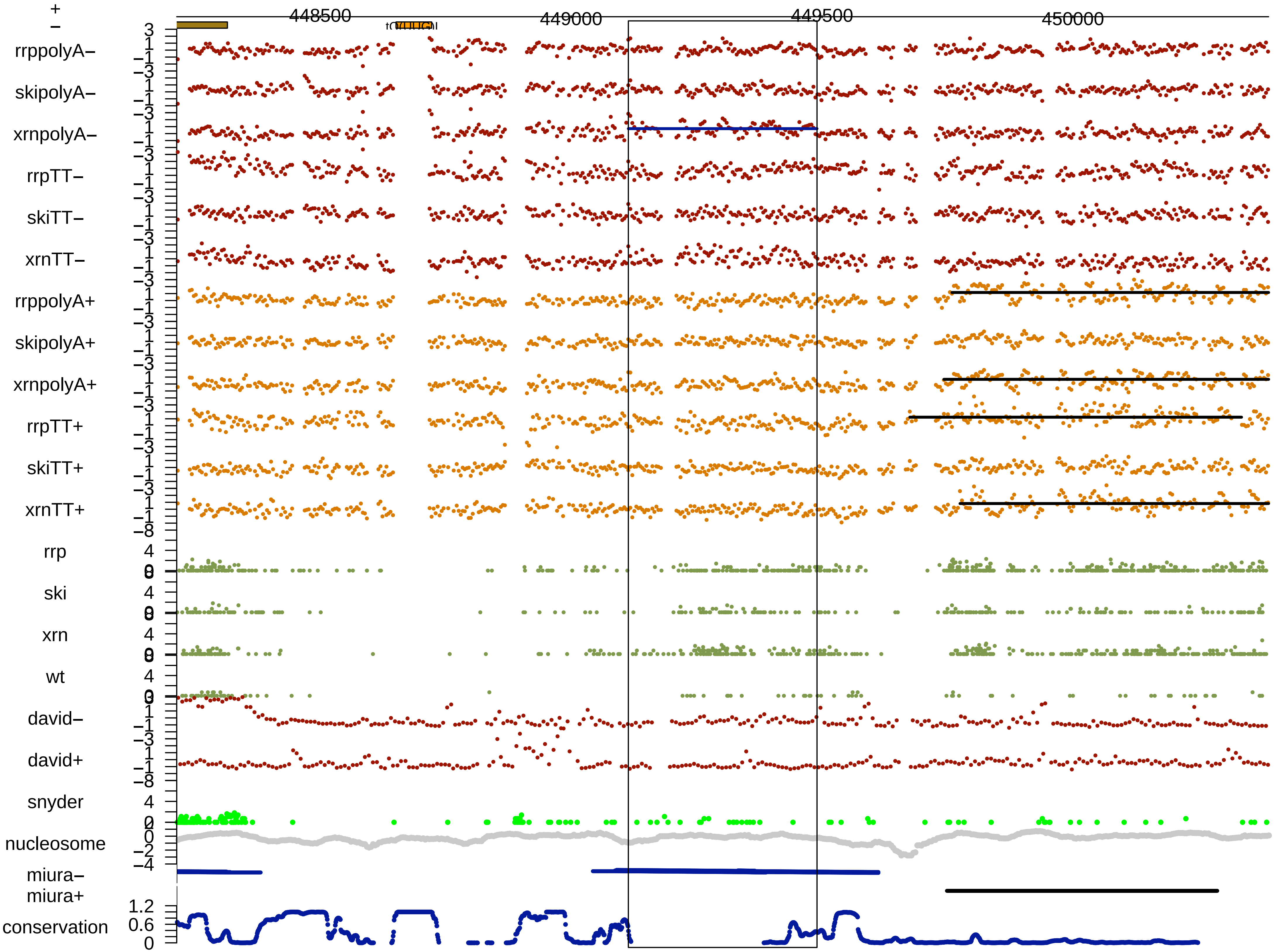

mcl.206, chr: 12

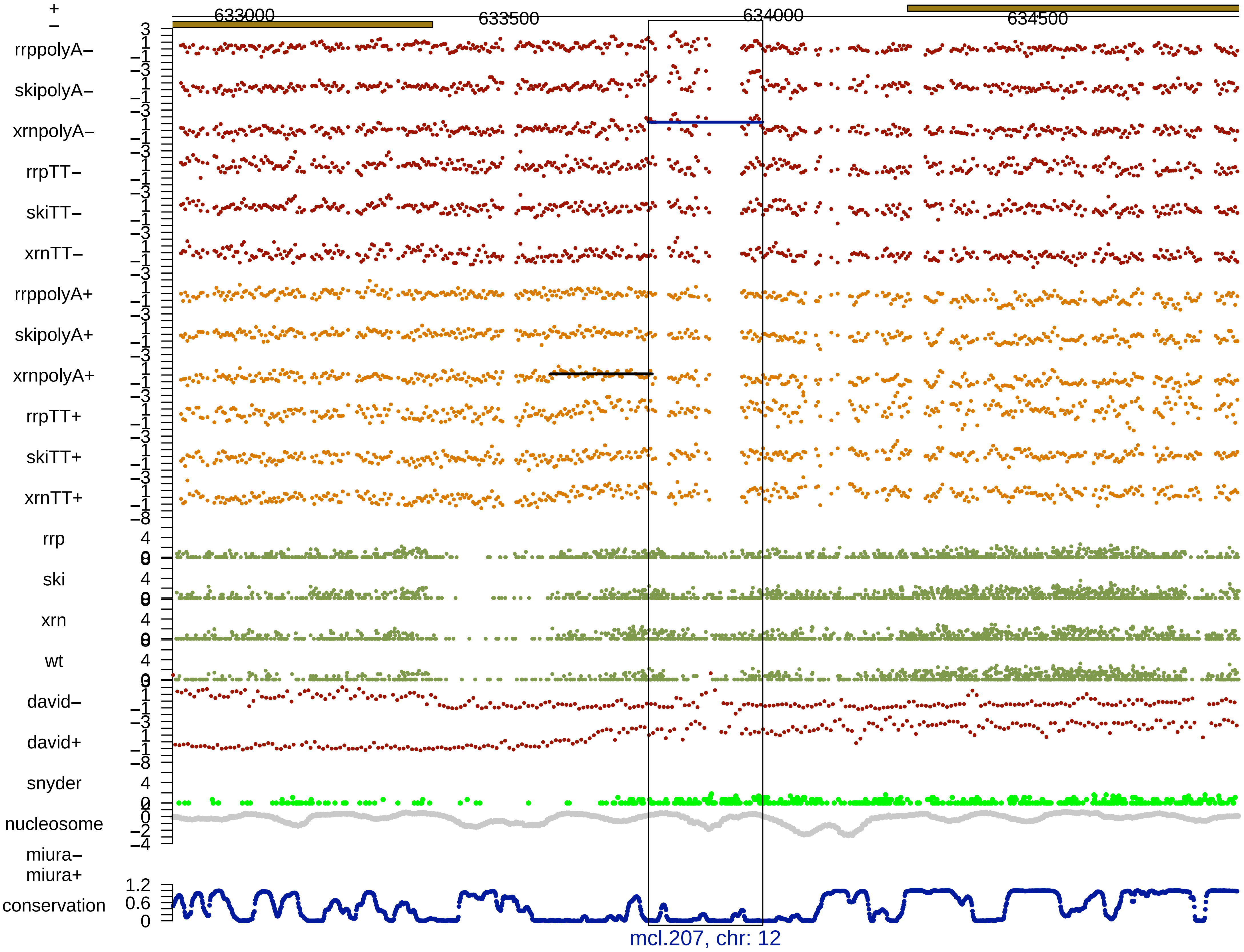
+
−
633000
633500
634000
634500
rrppolyA−
skipolyA−
xrnpolyA−
rrpTT−
skiTT−
xrnTT−
rrppolyA+
skipolyA+
xrnpolyA+
rrpTT+
skiTT+
xrnTT+
rrp
ski
xrn
wt
david−
david+
snyder
nucleosome
miura−
miura+
conservation
mcl.207, chr: 12

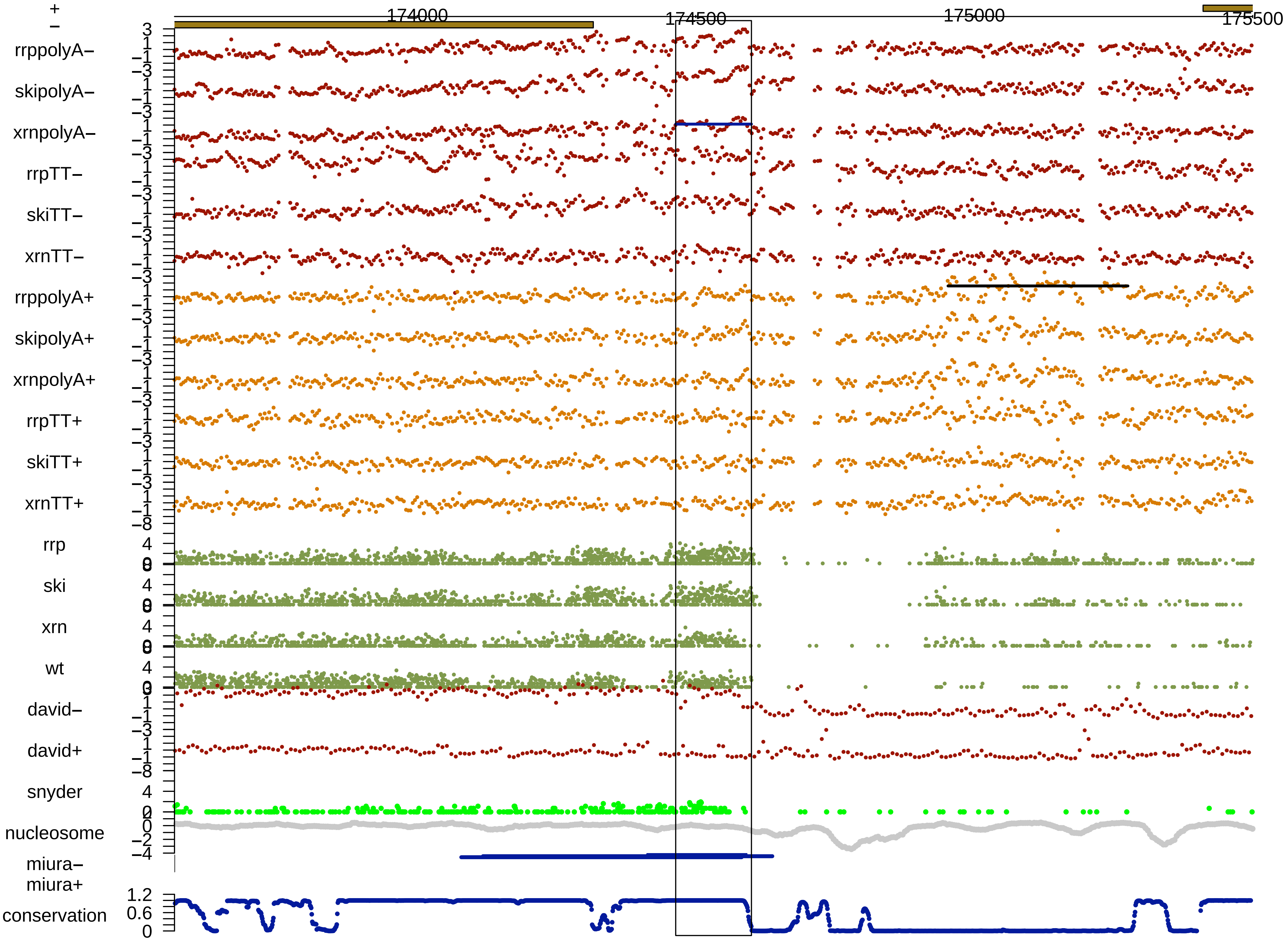

mcl.208, chr: 14

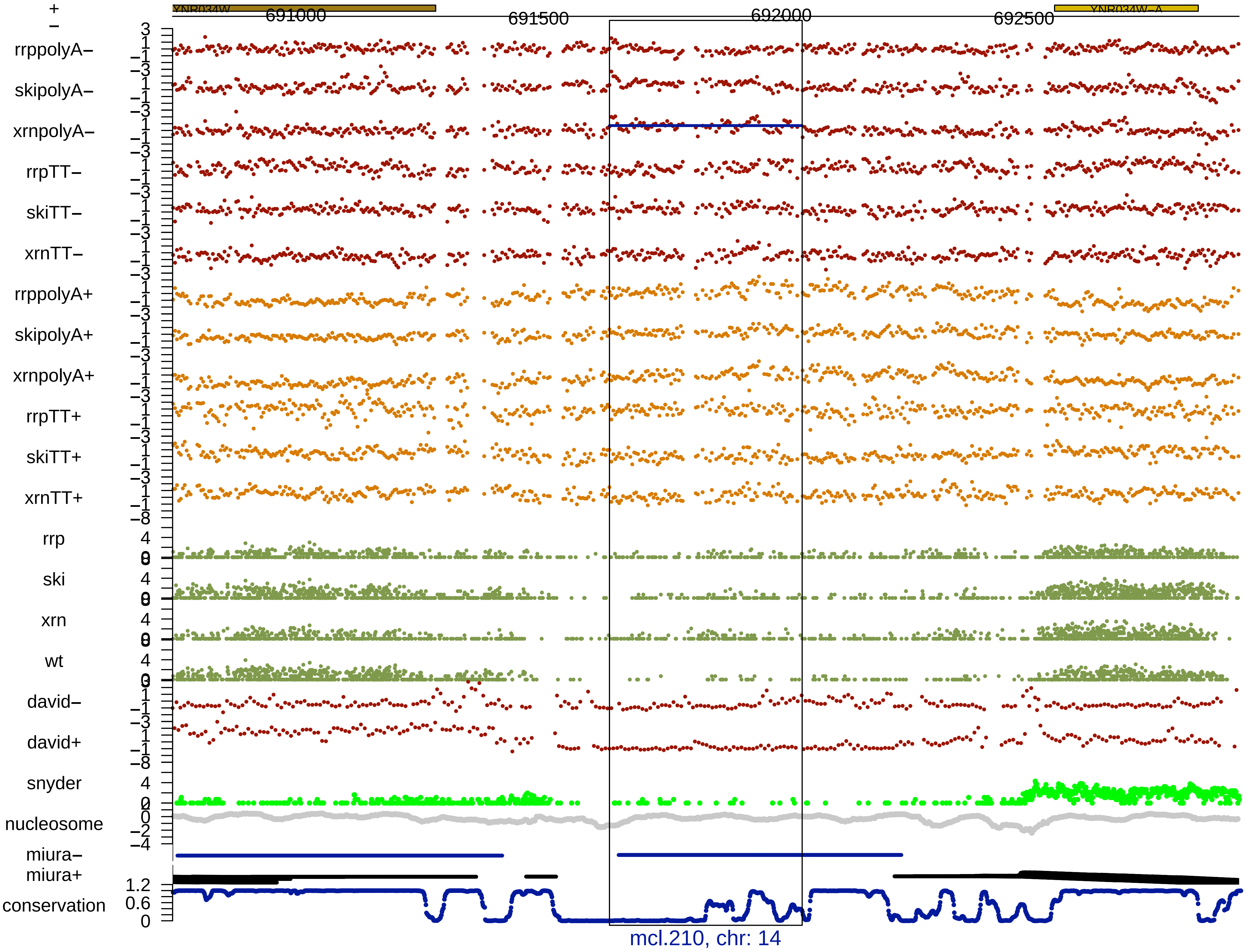

YNR034W
YNR034W-A
691000
691500
692000
692500
rrppolyA−
skipolyA−
xrnpolyA−
rrpTT−
skiTT−
xrnTT−
rrppolyA+
skipolyA+
xrnpolyA+
rrpTT+
skiTT+
xrnTT+
rrp
ski
xrn
wt
david−
david+
snyder
nucleosome
miura−
miura+
conservation
mcl.210, chr: 14

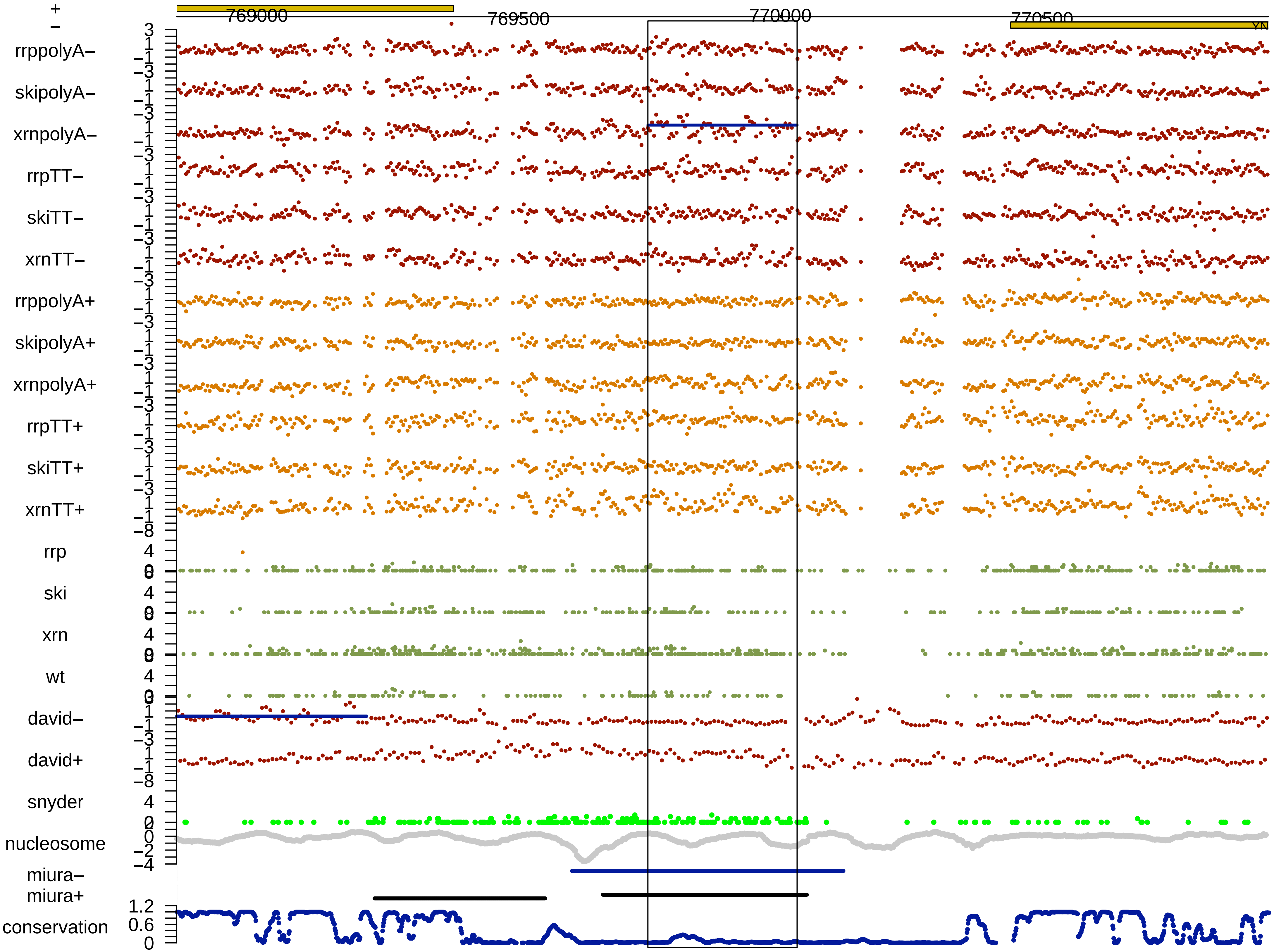

mcl.211, chr: 14

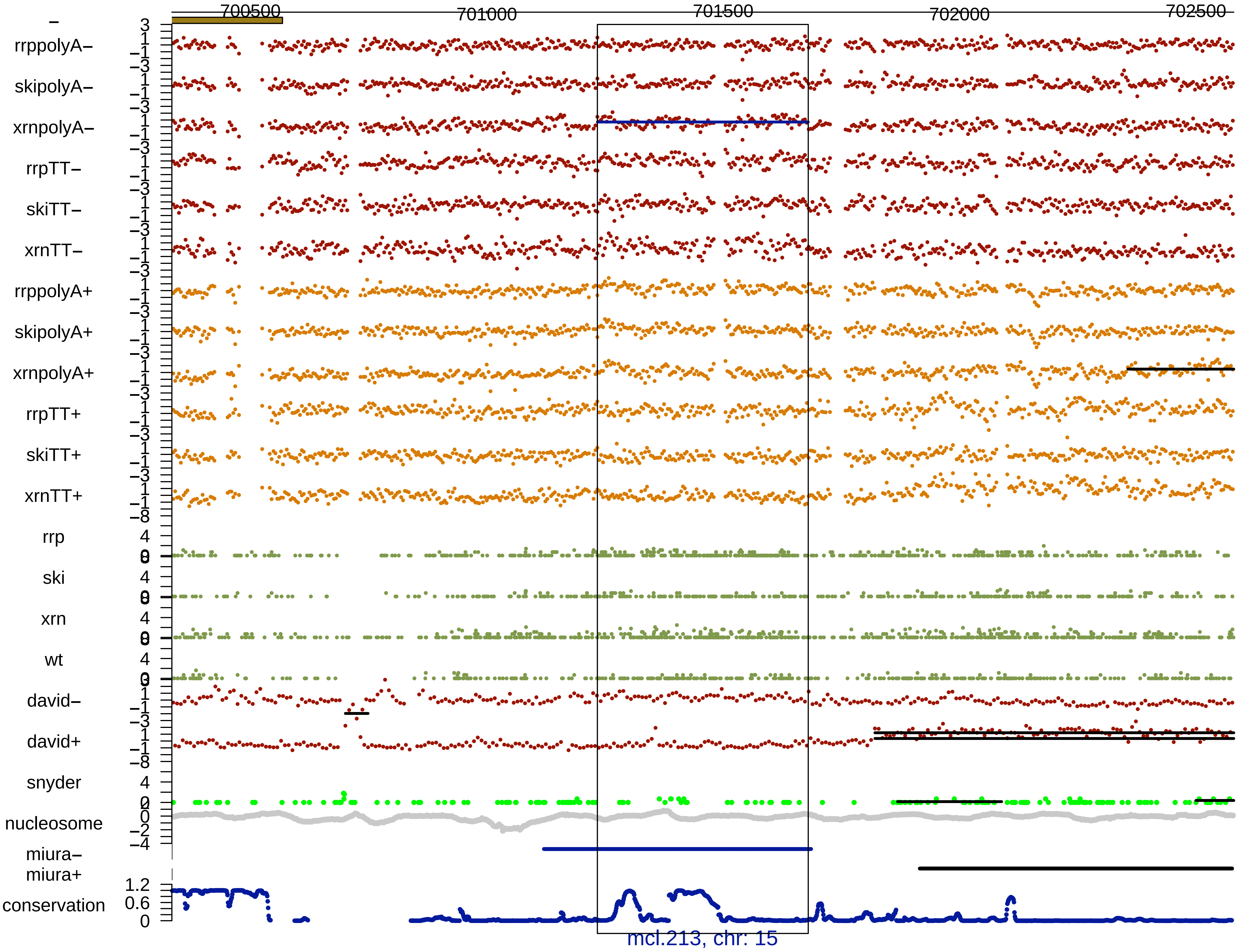
700500
701000
701500
702000
702500
–
rrppolyA–
skipolyA–
xrnpolyA–
rrpTT–
skiTT–
xrnTT–
rrppolyA+
skipolyA+
xrnpolyA+
rrpTT+
skiTT+
xrnTT+
rrp
ski
xrn
wt
david–
david+
snyder
nucleosome
miura–
miura+
conservation
mcl.213, chr: 15

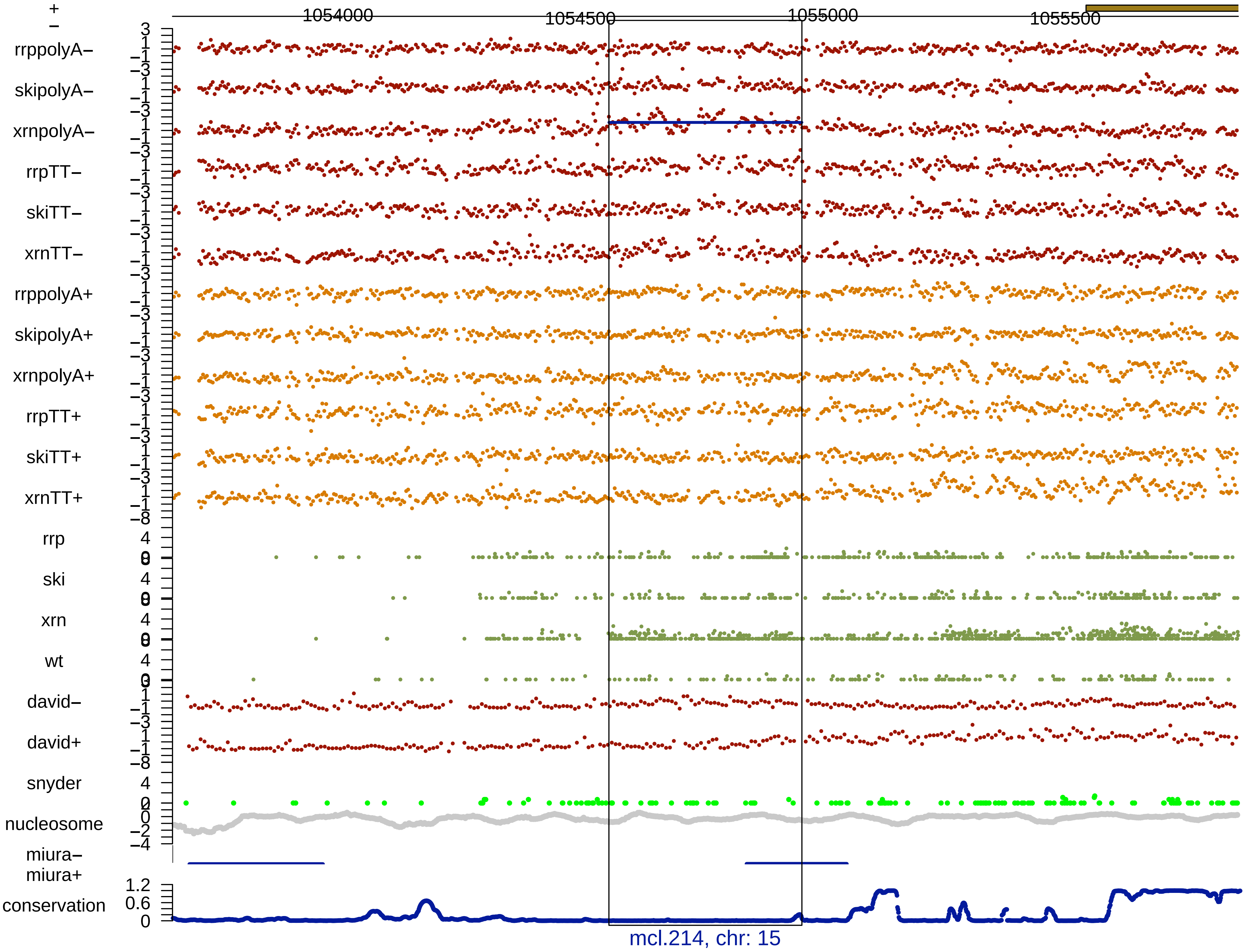

1054000
1054500
1055000
1055500
rrppolyA−
skipolyA−
xrnpolyA−
rrpTT−
skiTT−
xrnTT−
rrppolyA+
skipolyA+
xrnpolyA+
rrpTT+
skiTT+
xrnTT+
rrp
ski
xrn
wt
david−
david+
snyder
nucleosome
miura−
miura+
conservation
mcl.214, chr: 15

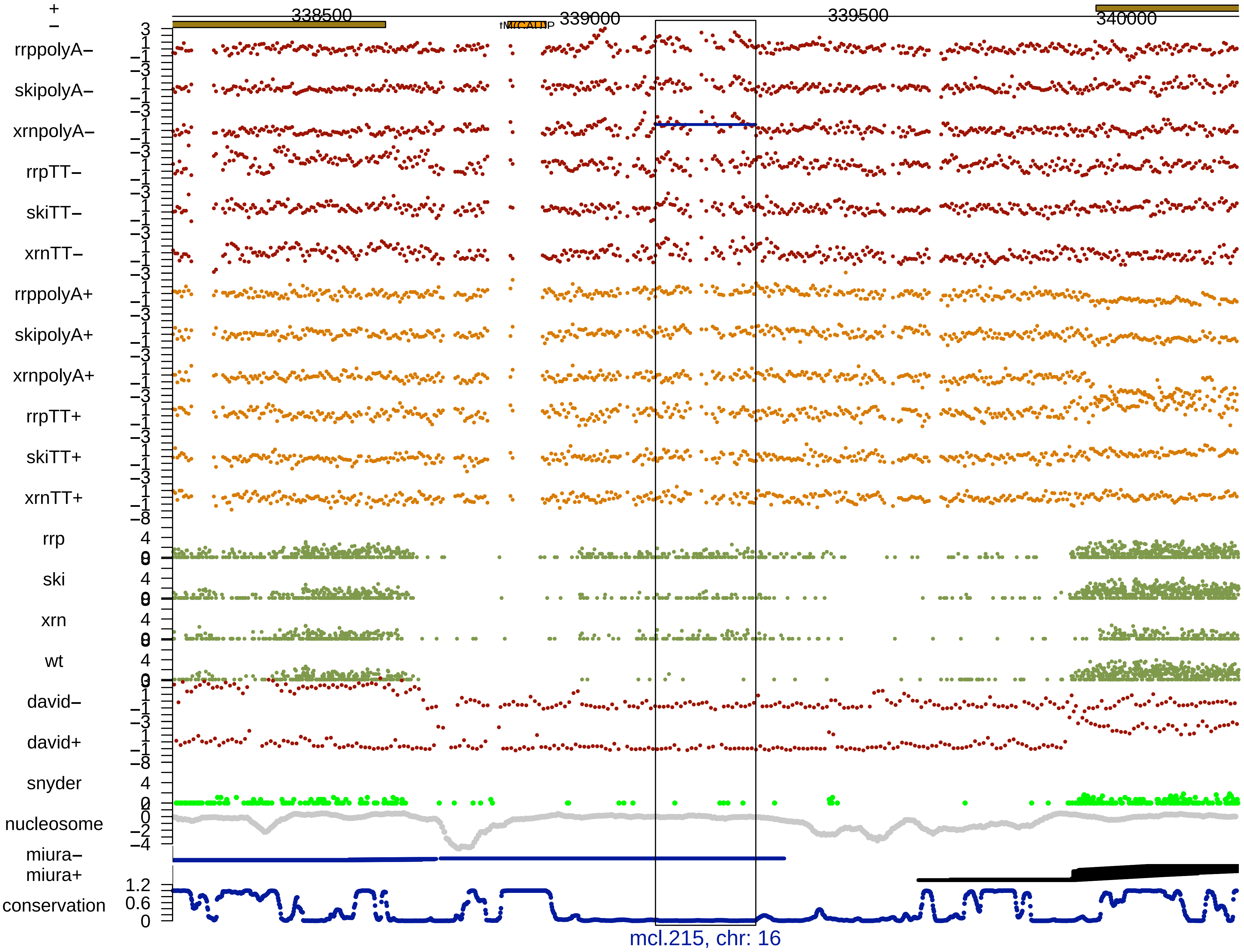

338500
339000
339500
340000
tM(CAU)P
rrppolyA−
skipolyA−
xrnpolyA−
rrpTT−
skiTT−
xrnTT−
rrppolyA+
skipolyA+
xrnpolyA+
rrpTT+
skiTT+
xrnTT+
rrp
ski
xrn
wt
david−
david+
snyder
nucleosome
miura−
miura+
conservation
mcl.215, chr: 16

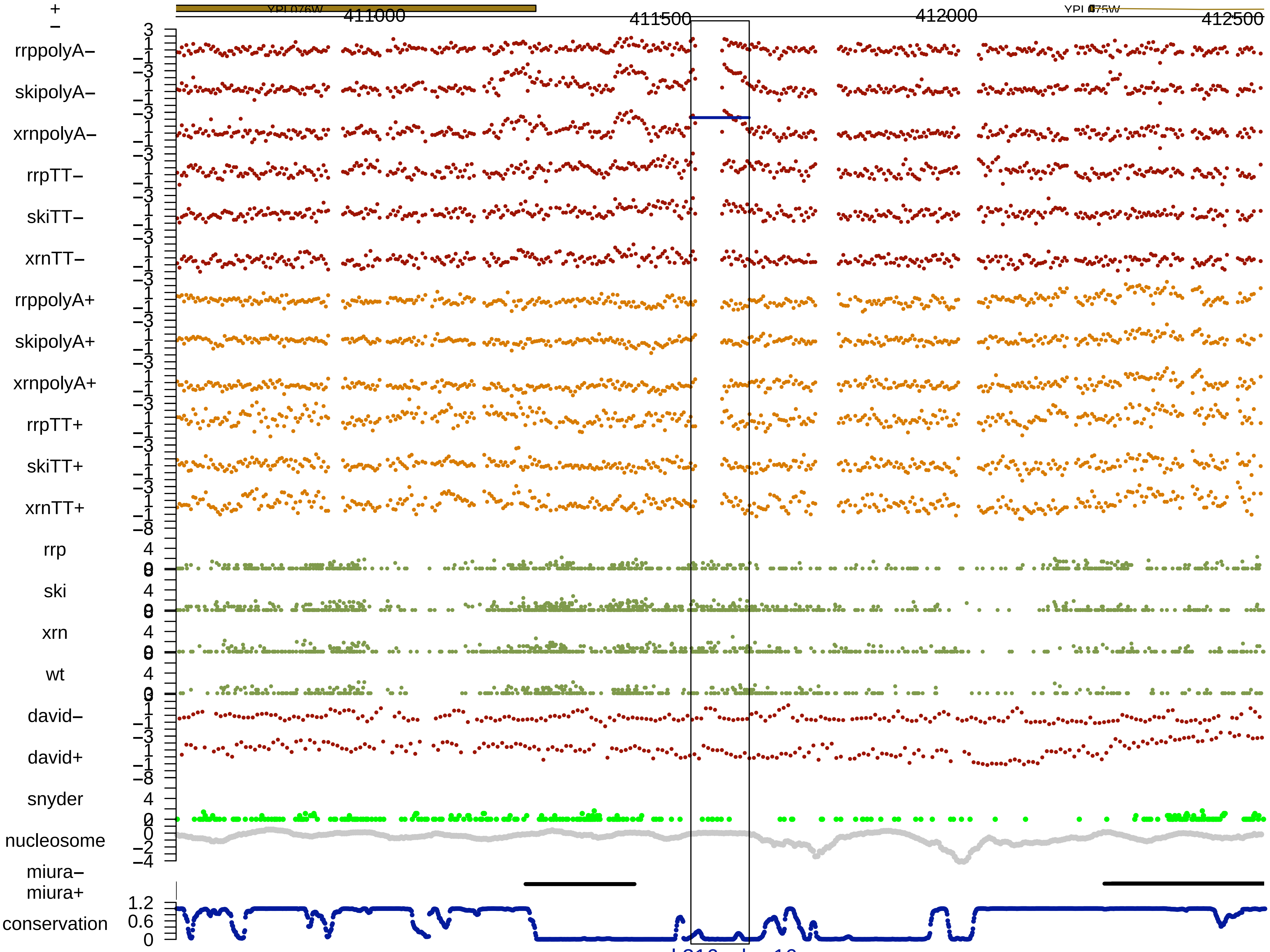

YPL076W
YPL075W
411000
411500
412000
412500
rrppolyA−
skipolyA−
xrnpolyA−
rrpTT−
skiTT−
xrnTT−
rrppolyA+
skipolyA+
xrnpolyA+
rrpTT+
skiTT+
xrnTT+
rrp
ski
xrn
wt
david−
david+
snyder
nucleosome
miura−
miura+
conservation
mcl.216, chr: 16

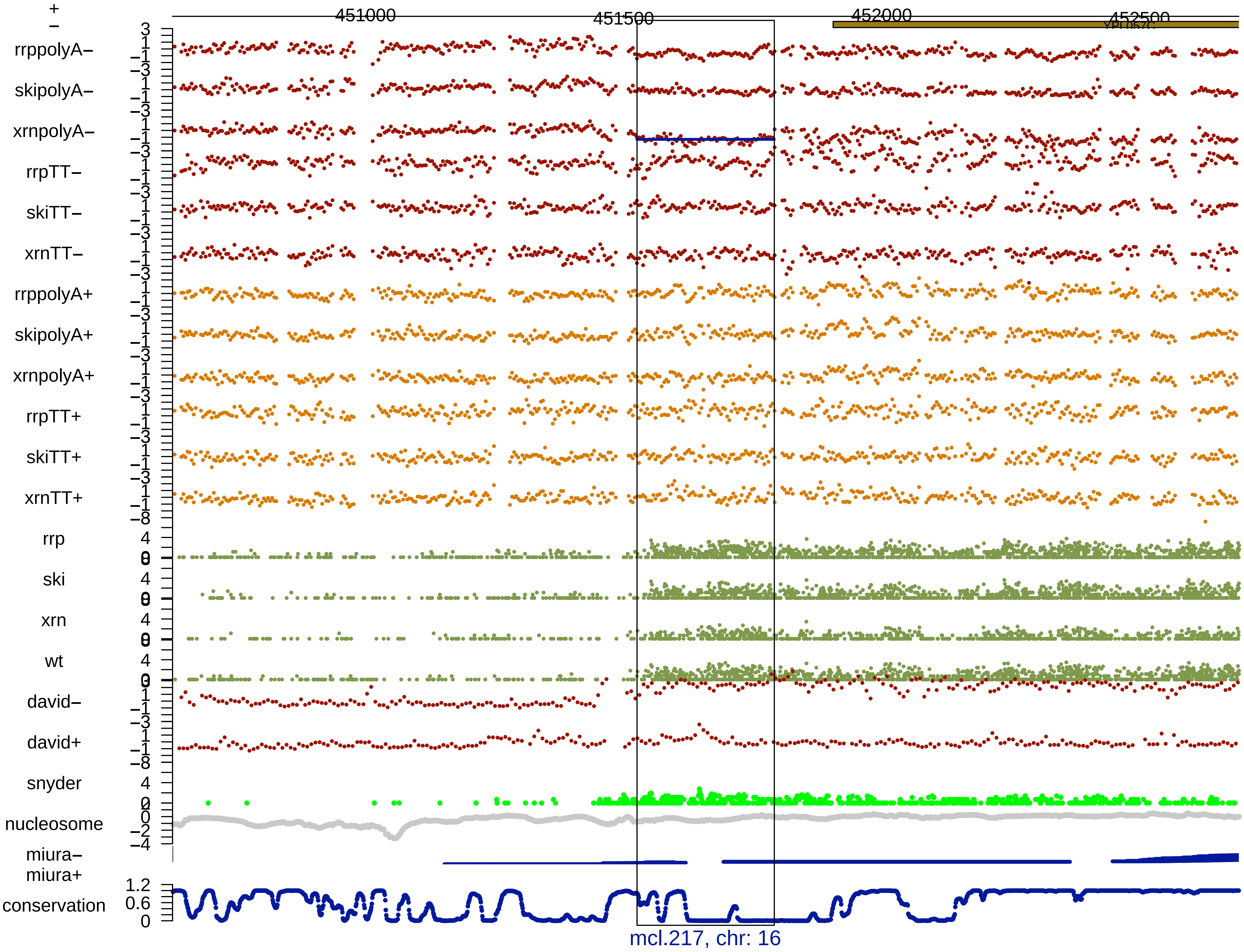

451000
451500
452000
452500
YPL057C
rrppolyA−
skipolyA−
xrnpolyA−
rrpTT−
skiTT−
xrnTT−
rrppolyA+
skipolyA+
xrnpolyA+
rrpTT+
skiTT+
xrnTT+
rrp
ski
xrn
wt
david−
david+
snyder
nucleosome
miura−
miura+
conservation
mcl.217, chr: 16

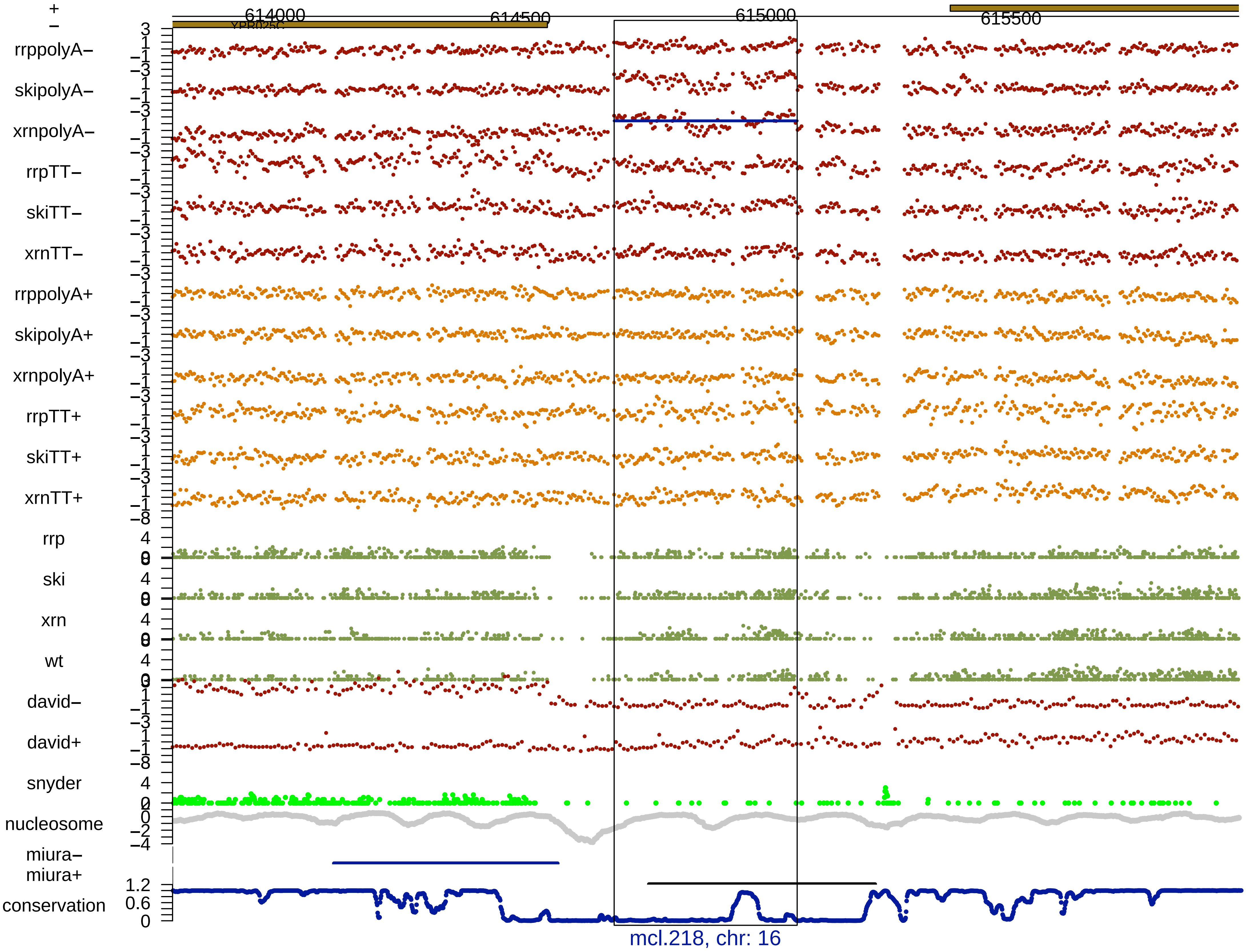

+
−
614000
614500
615000
615500
rrppolyA−
skipolyA−
xrnpolyA−
rrpTT−
skiTT−
xrnTT−
rrppolyA+
skipolyA+
xrnpolyA+
rrpTT+
skiTT+
xrnTT+
rrp
ski
xrn
wt
david−
david+
snyder
nucleosome
miura−
miura+
conservation
mcl.218, chr: 16

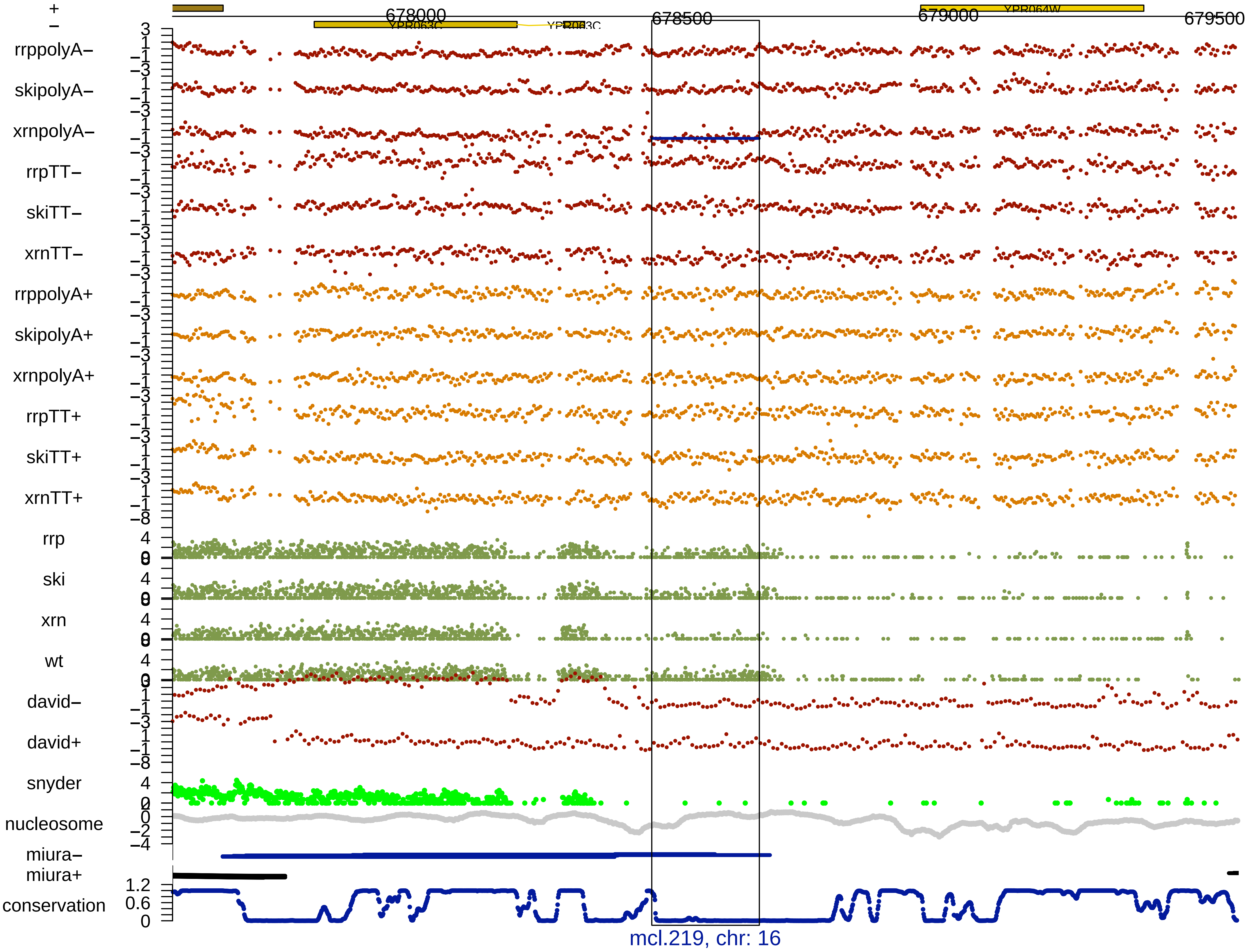

678000
678500
679000
679500
YPR063C
YPR064W
rrppolyA−
skipolyA−
xrnpolyA−
rrpTT−
skiTT−
xrnTT−
rrppolyA+
skipolyA+
xrnpolyA+
rrpTT+
skiTT+
xrnTT+
rrp
ski
xrn
wt
david−
david+
snyder
nucleosome
miura−
miura+
conservation
mcl.219, chr: 16

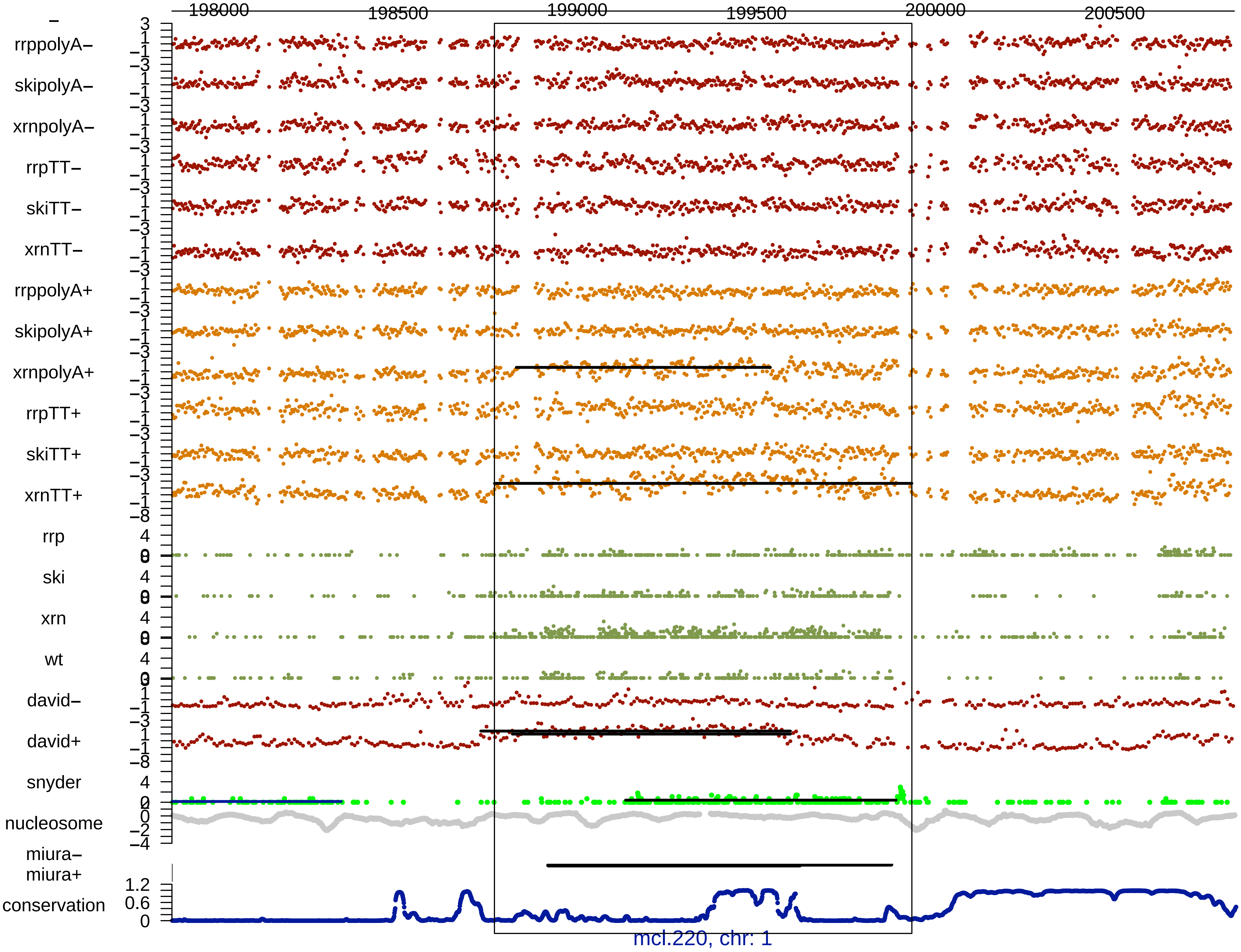

198000
198500
199000
199500
200000
200500
rrppolyA−
skipolyA−
xrnpolyA−
rrpTT−
skiTT−
xrnTT−
rrppolyA+
skipolyA+
xrnpolyA+
rrpTT+
skiTT+
xrnTT+
rrp
ski
xrn
wt
david−
david+
snyder
nucleosome
miura−
miura+
conservation
mcl.220, chr: 1

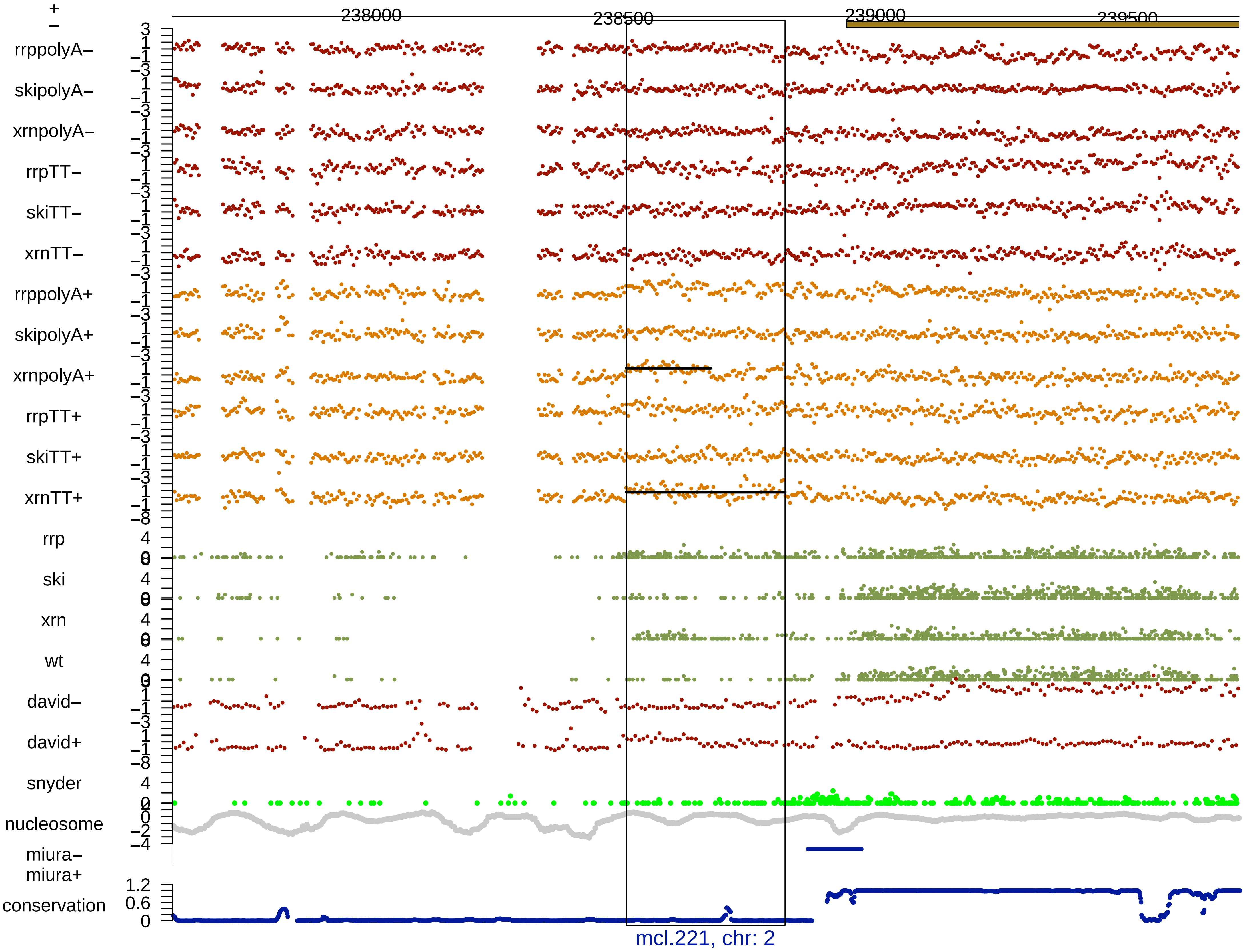
+
−
238000
238500
239000
239500
rrppolyA−
skipolyA−
xrnpolyA−
rrpTT−
skiTT−
xrnTT−
rrppolyA+
skipolyA+
xrnpolyA+
rrpTT+
skiTT+
xrnTT+
rrp
ski
xrn
wt
david−
david+
snyder
nucleosome
miura−
miura+
conservation
mcl.221, chr: 2

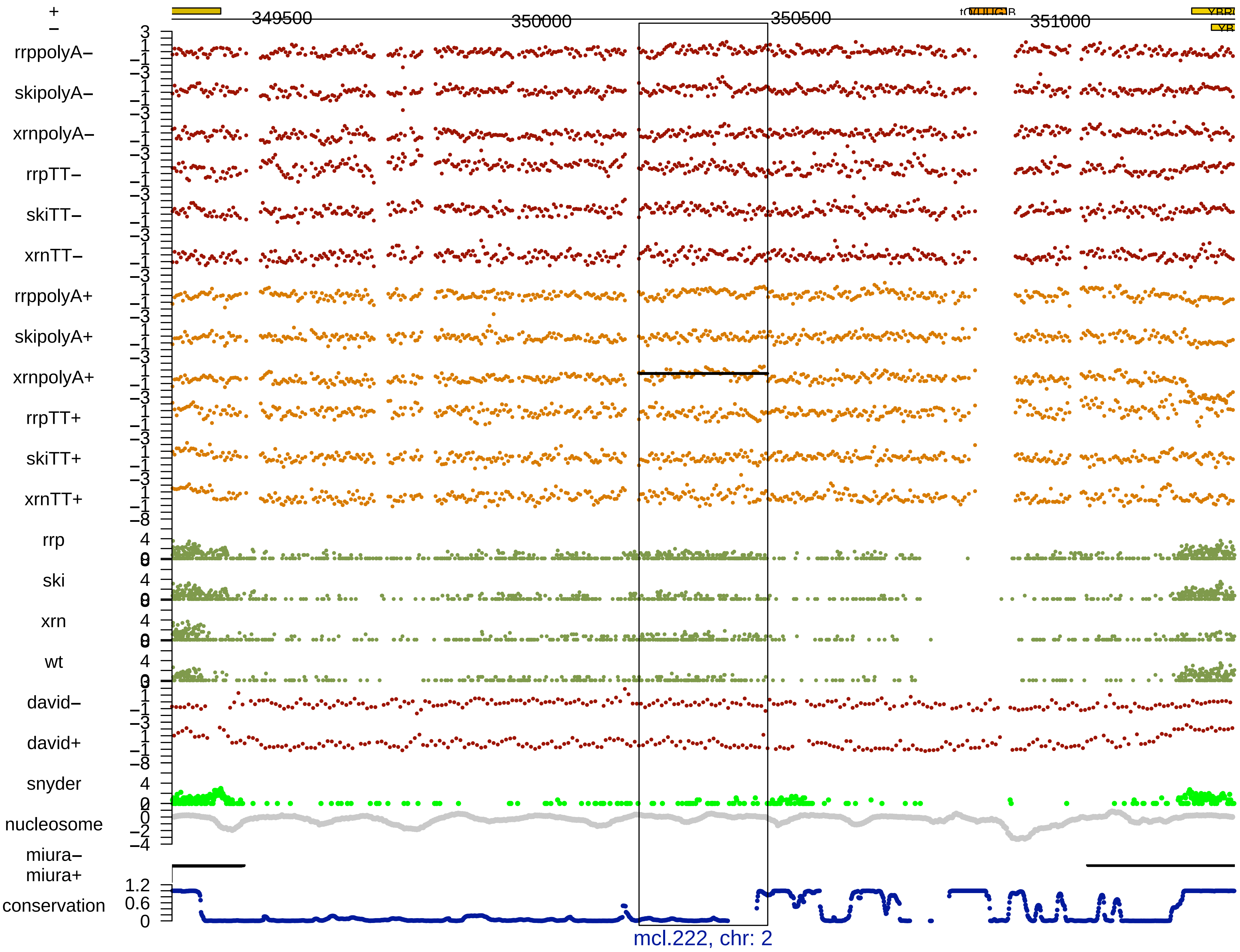
349500
350000
350500
351000
+
−
rrppolyA−
skipolyA−
xrnpolyA−
rrpTT−
skiTT−
xrnTT−
rrppolyA+
skipolyA+
xrnpolyA+
rrpTT+
skiTT+
xrnTT+
rrp
ski
xrn
wt
david−
david+
snyder
nucleosome
miura−
miura+
conservation
mcl.222, chr: 2

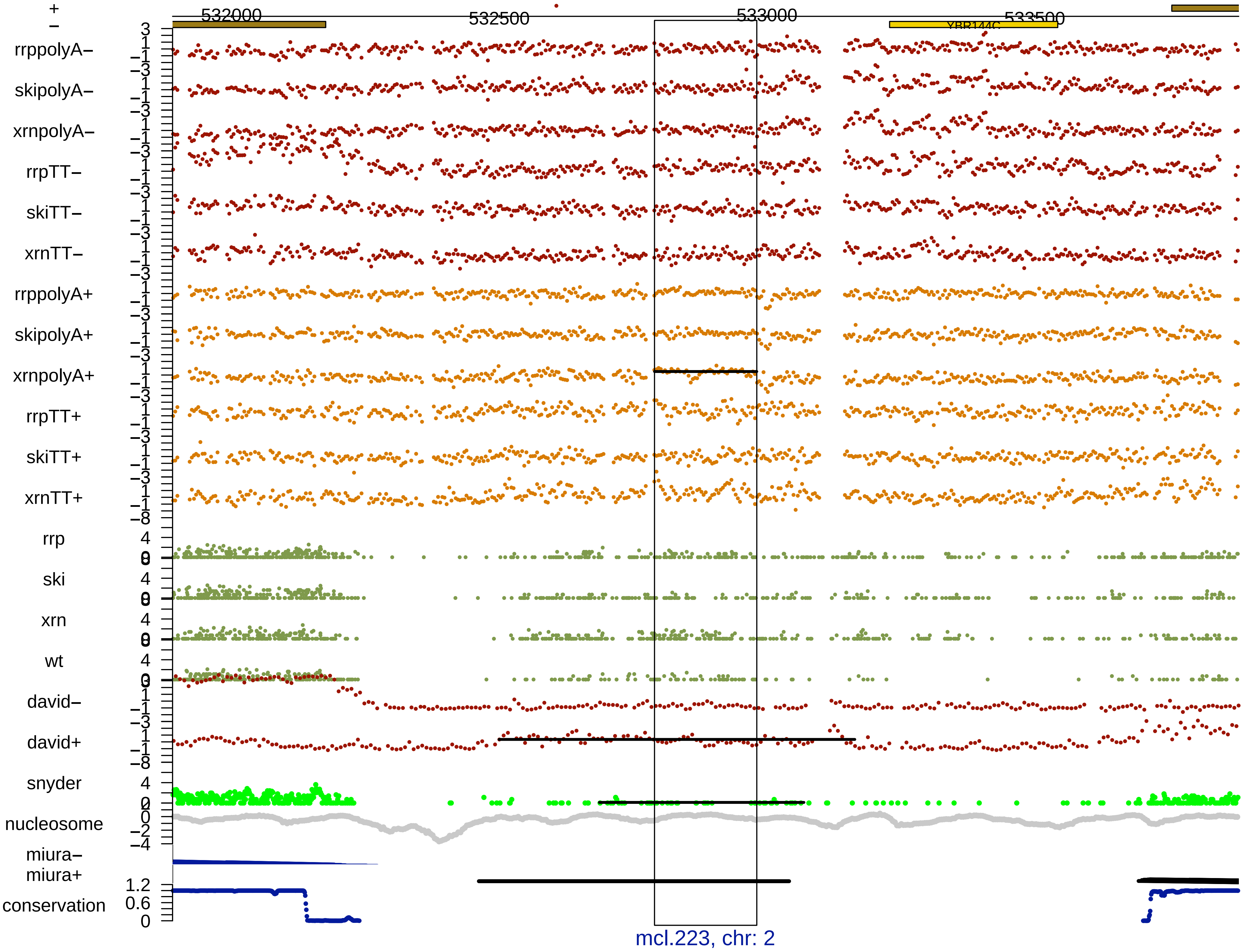

532000
532500
533000
533500
YBR144C
rrppolyA−
skipolyA−
xrnpolyA−
rrpTT−
skiTT−
xrnTT−
rrppolyA+
skipolyA+
xrnpolyA+
rrpTT+
skiTT+
xrnTT+
rrp
ski
xrn
wt
david−
david+
snyder
nucleosome
miura−
miura+
conservation
mcl.223, chr: 2

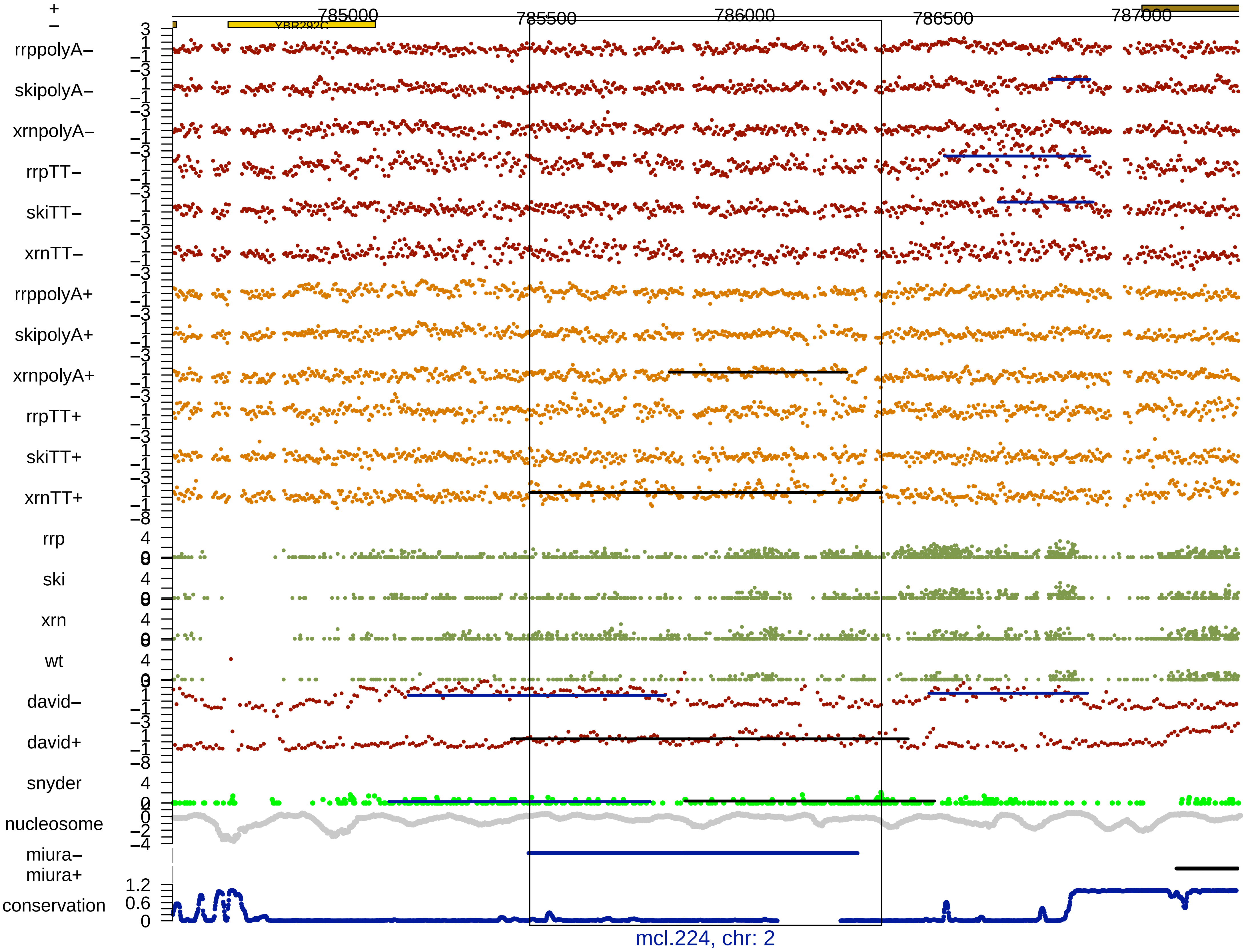

785000
785500
786000
786500
787000
YBR292C
rrppolyA−
skipolyA−
xrnpolyA−
rrpTT−
skiTT−
xrnTT−
rrppolyA+
skipolyA+
xrnpolyA+
rrpTT+
skiTT+
xrnTT+
rrp
ski
xrn
wt
david−
david+
snyder
nucleosome
miura−
miura+
conservation
mcl.224, chr: 2

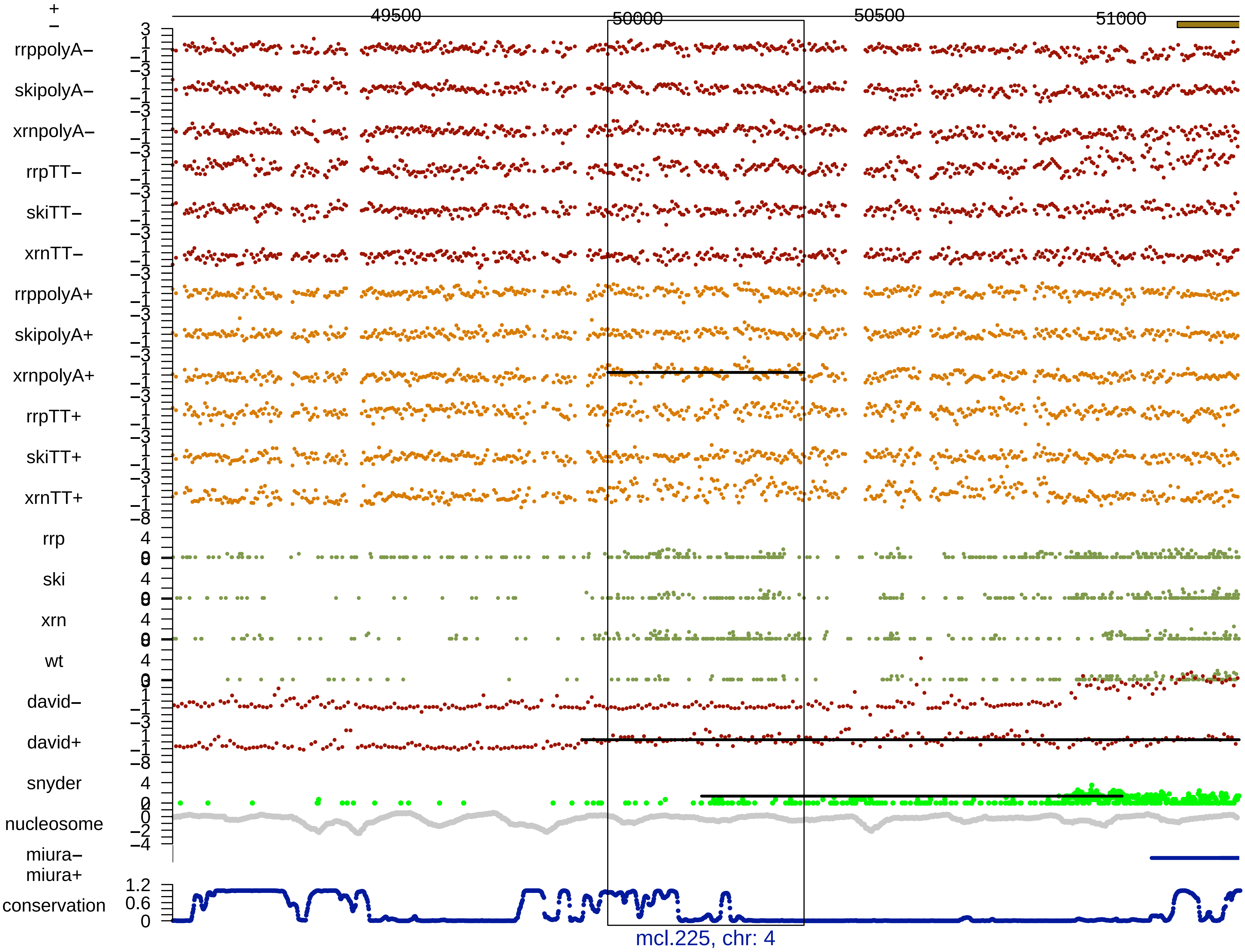

49500
50000
50500
51000
rrppolyA−
skipolyA−
xrnpolyA−
rrpTT−
skiTT−
xrnTT−
rrppolyA+
skipolyA+
xrnpolyA+
rrpTT+
skiTT+
xrnTT+
rrp
ski
xrn
wt
david−
david+
snyder
nucleosome
miura−
miura+
conservation
mcl.225, chr: 4

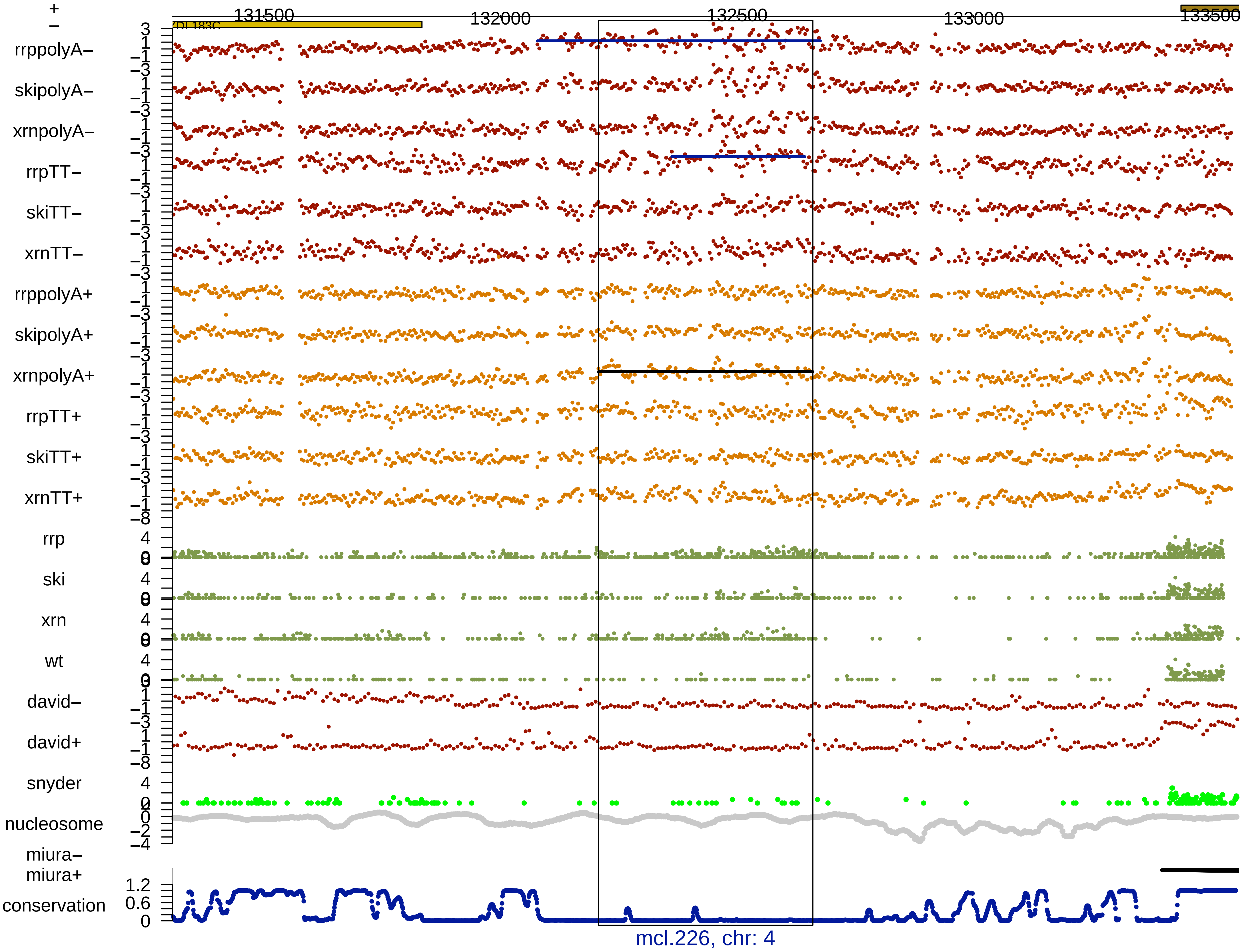
131500
132000
132500
133000
133500
rrppolyA−
skipolyA−
xrnpolyA−
rrpTT−
skiTT−
xrnTT−
rrppolyA+
skipolyA+
xrnpolyA+
rrpTT+
skiTT+
xrnTT+
rrp
ski
xrn
wt
david−
david+
snyder
nucleosome
miura−
miura+
conservation
mcl.226, chr: 4

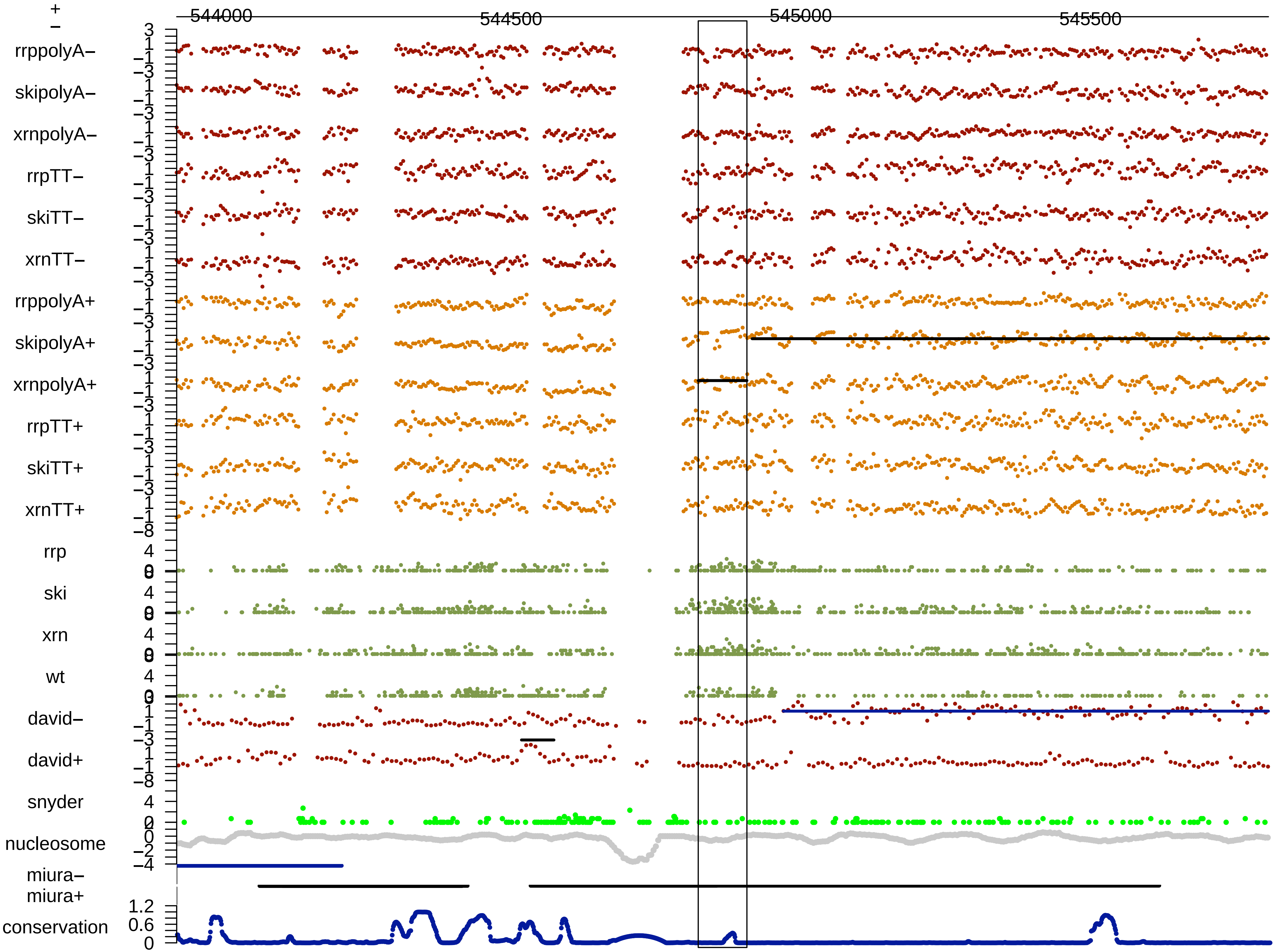

544000
544500
545000
545500
rrppolyA−
skipolyA−
xrnpolyA−
rrpTT−
skiTT−
xrnTT−
rrppolyA+
skipolyA+
xrnpolyA+
rrpTT+
skiTT+
xrnTT+
rrp
ski
xrn
wt
david−
david+
snyder
nucleosome
miura−
miura+
conservation

mcl.227, chr: 4

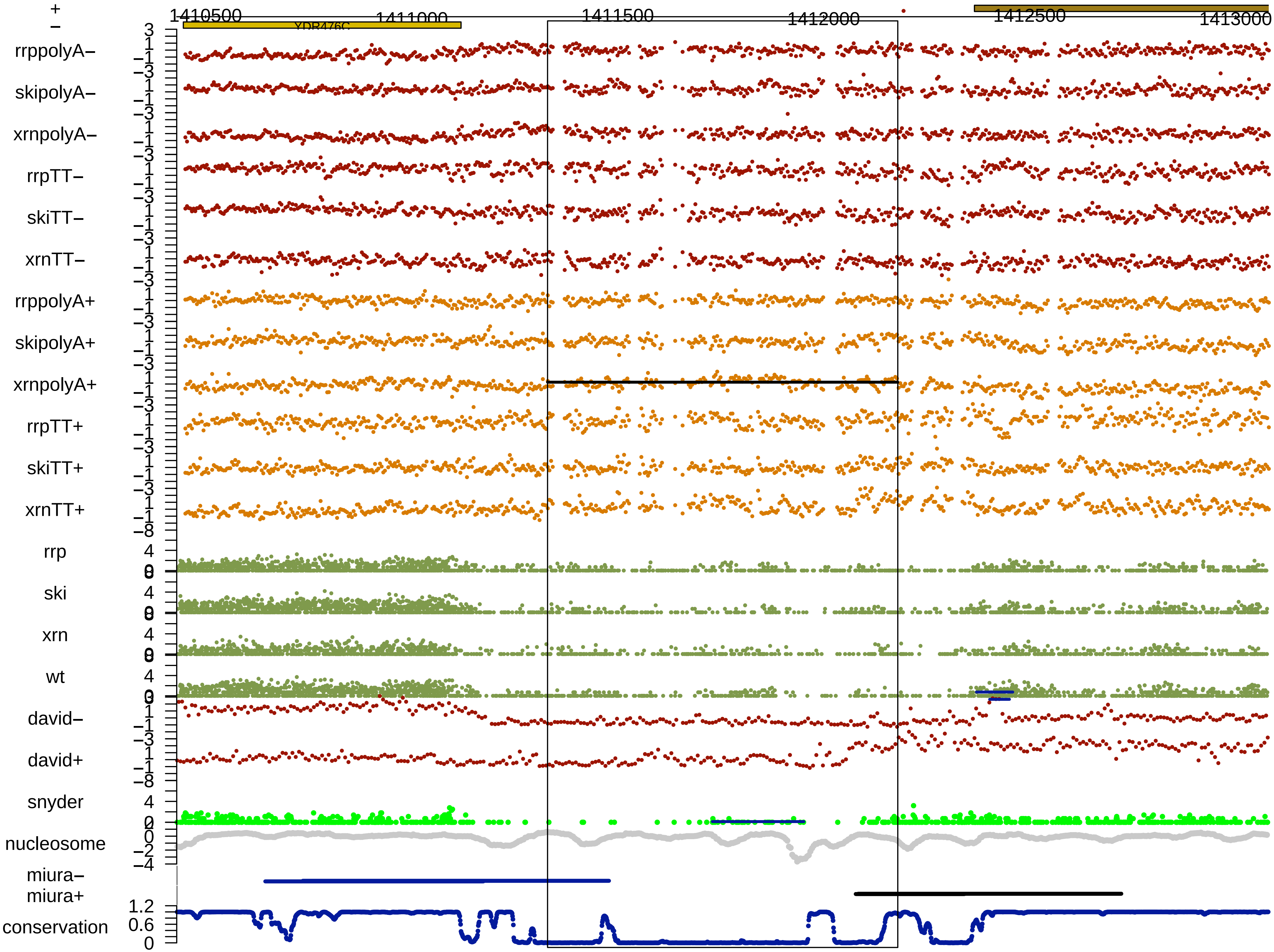
1410500
1411000
1411500
1412000
1412500
1413000
YDR476C
rrppolyA−
skipolyA−
xrnpolyA−
rrpTT−
skiTT−
xrnTT−
rrppolyA+
skipolyA+
xrnpolyA+
rrpTT+
skiTT+
xrnTT+
rrp
ski
xrn
wt
david−
david+
snyder
nucleosome
miura−
miura+
conservation

mcl.228, chr: 4

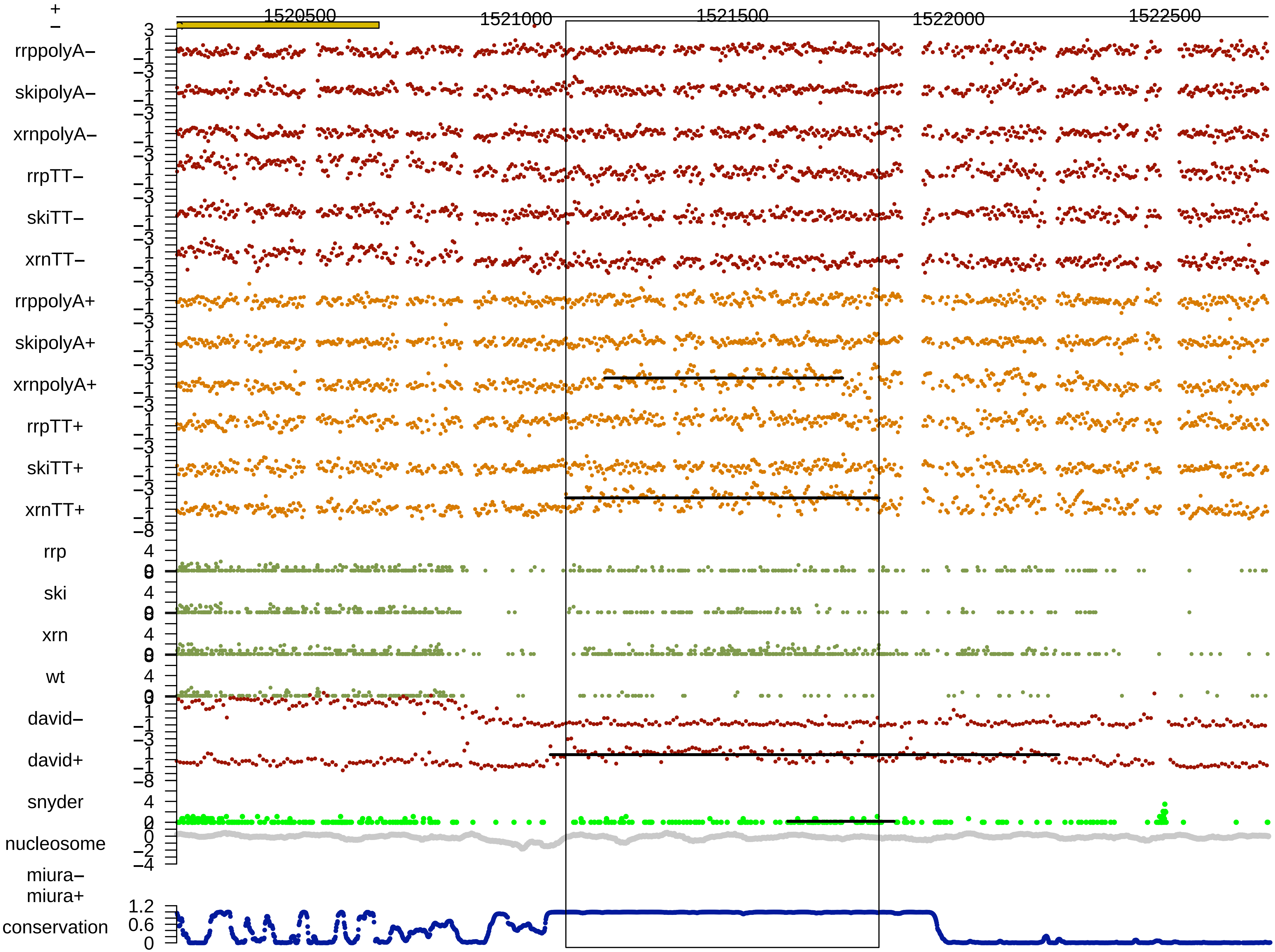

1520500
1521000
1521500
1522000
1522500
+
−
rrppolyA−
skipolyA−
xrnpolyA−
rrpTT−
skiTT−
xrnTT−
rrppolyA+
skipolyA+
xrnpolyA+
rrpTT+
skiTT+
xrnTT+
rrp
ski
xrn
wt
david−
david+
snyder
nucleosome
miura−
miura+
conservation
1.2
0.6
0

mcl.229, chr: 4

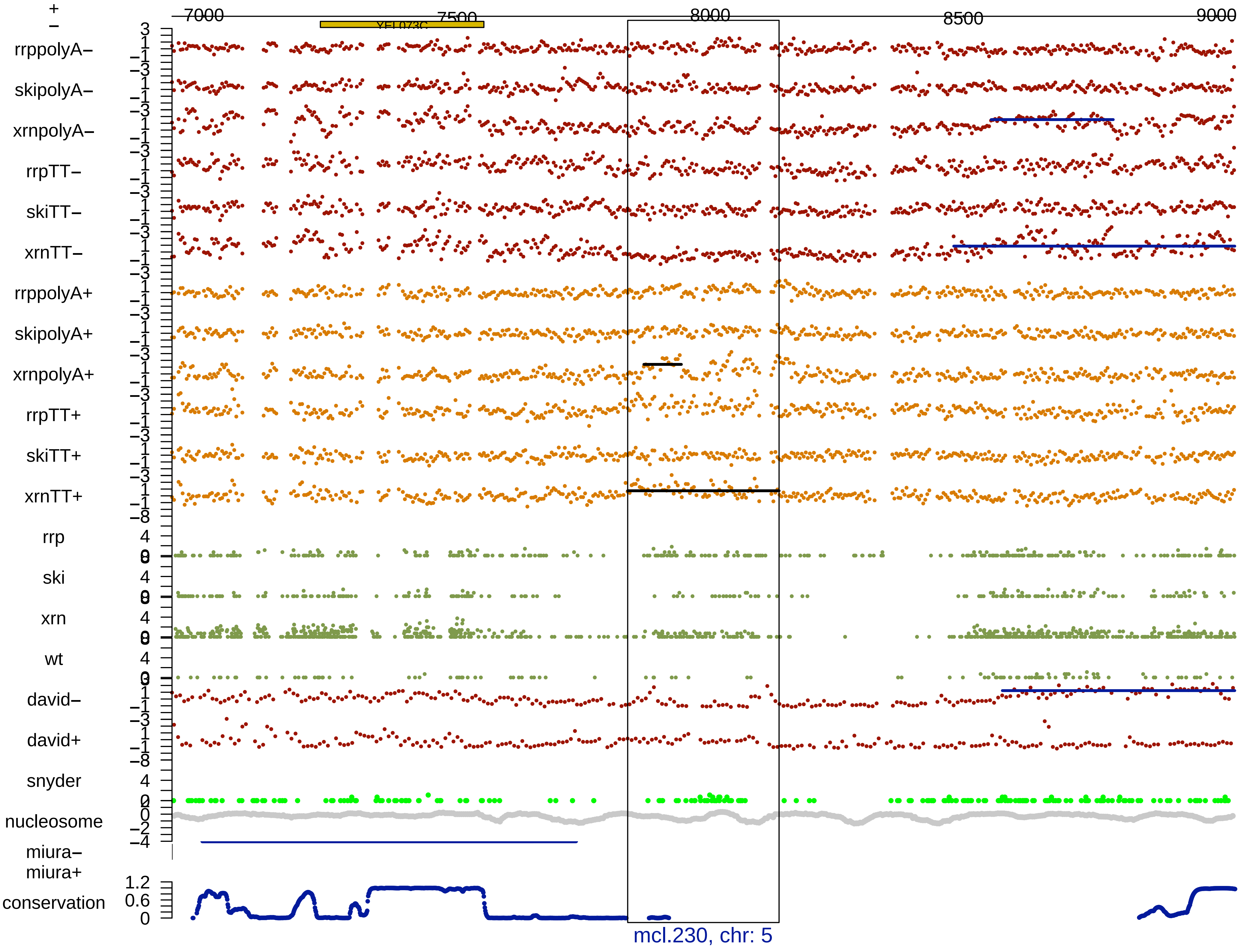

+
−
7000
7500
8000
8500
9000
YEL073C
rrppolyA−
skipolyA−
xrnpolyA−
rrpTT−
skiTT−
xrnTT−
rrppolyA+
skipolyA+
xrnpolyA+
rrpTT+
skiTT+
xrnTT+
rrp
ski
xrn
wt
david−
david+
snyder
nucleosome
miura−
miura+
conservation
mcl.230, chr: 5

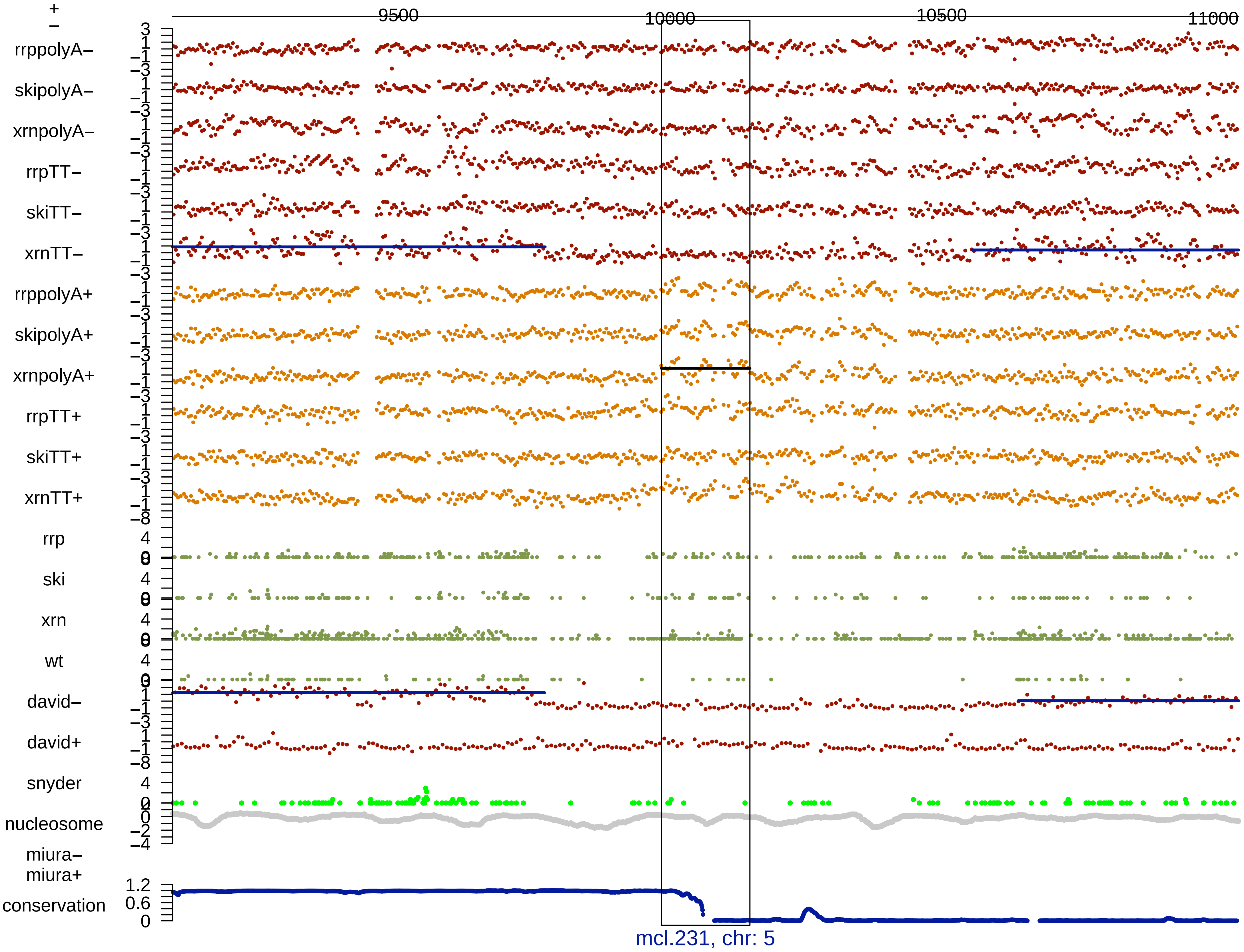

9500
10000
10500
11000
rrppolyA−
skipolyA−
xrnpolyA−
rrpTT−
skiTT−
xrnTT−
rrppolyA+
skipolyA+
xrnpolyA+
rrpTT+
skiTT+
xrnTT+
rrp
ski
xrn
wt
david−
david+
snyder
nucleosome
miura−
miura+
conservation
mcl.231, chr: 5

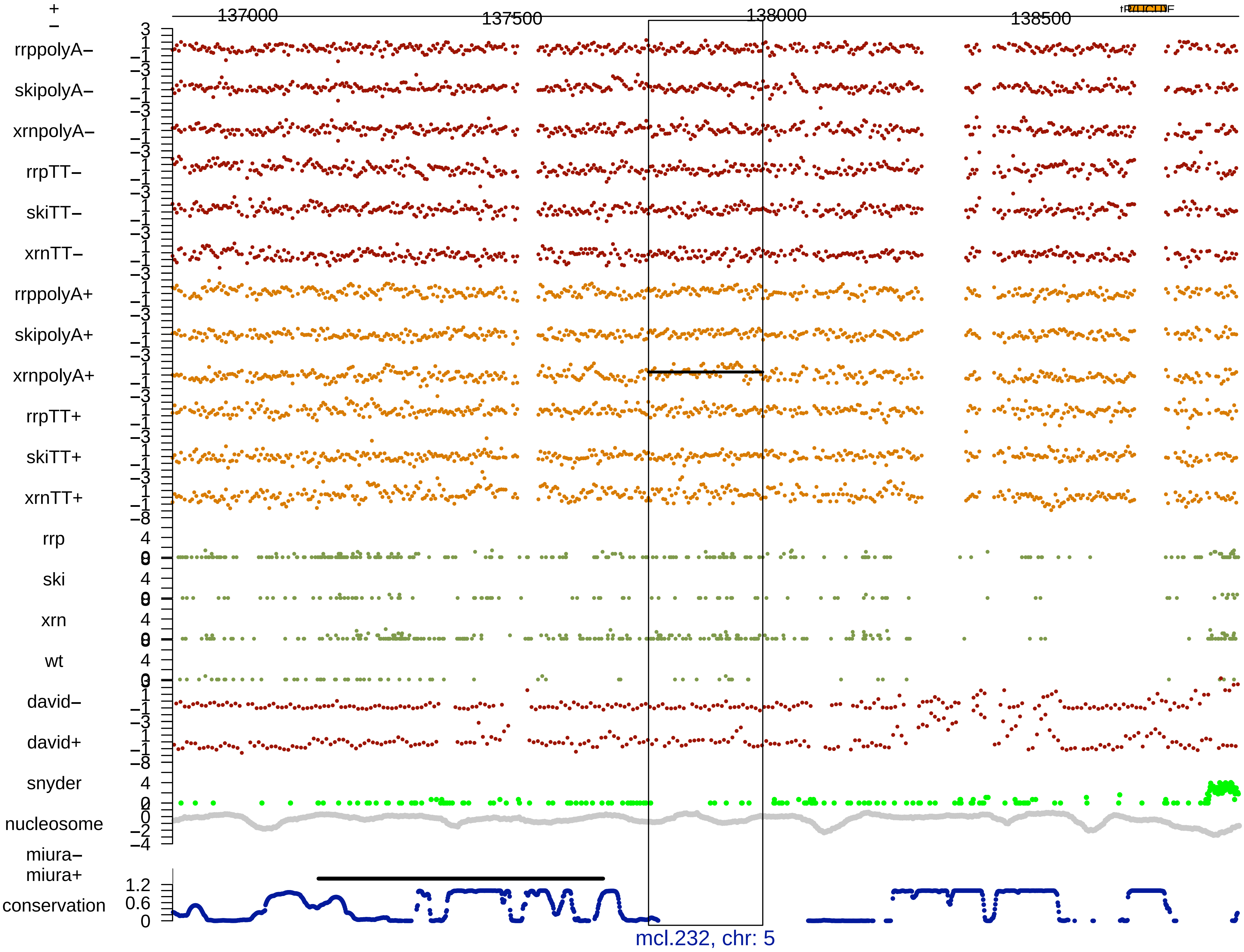

+
−
137000
137500
138000
138500
rrppolyA−
skipolyA−
xrnpolyA−
rrpTT−
skiTT−
xrnTT−
rrppolyA+
skipolyA+
xrnpolyA+
rrpTT+
skiTT+
xrnTT+
rrp
ski
xrn
wt
david−
david+
snyder
nucleosome
miura−
miura+
conservation
mcl.232, chr: 5

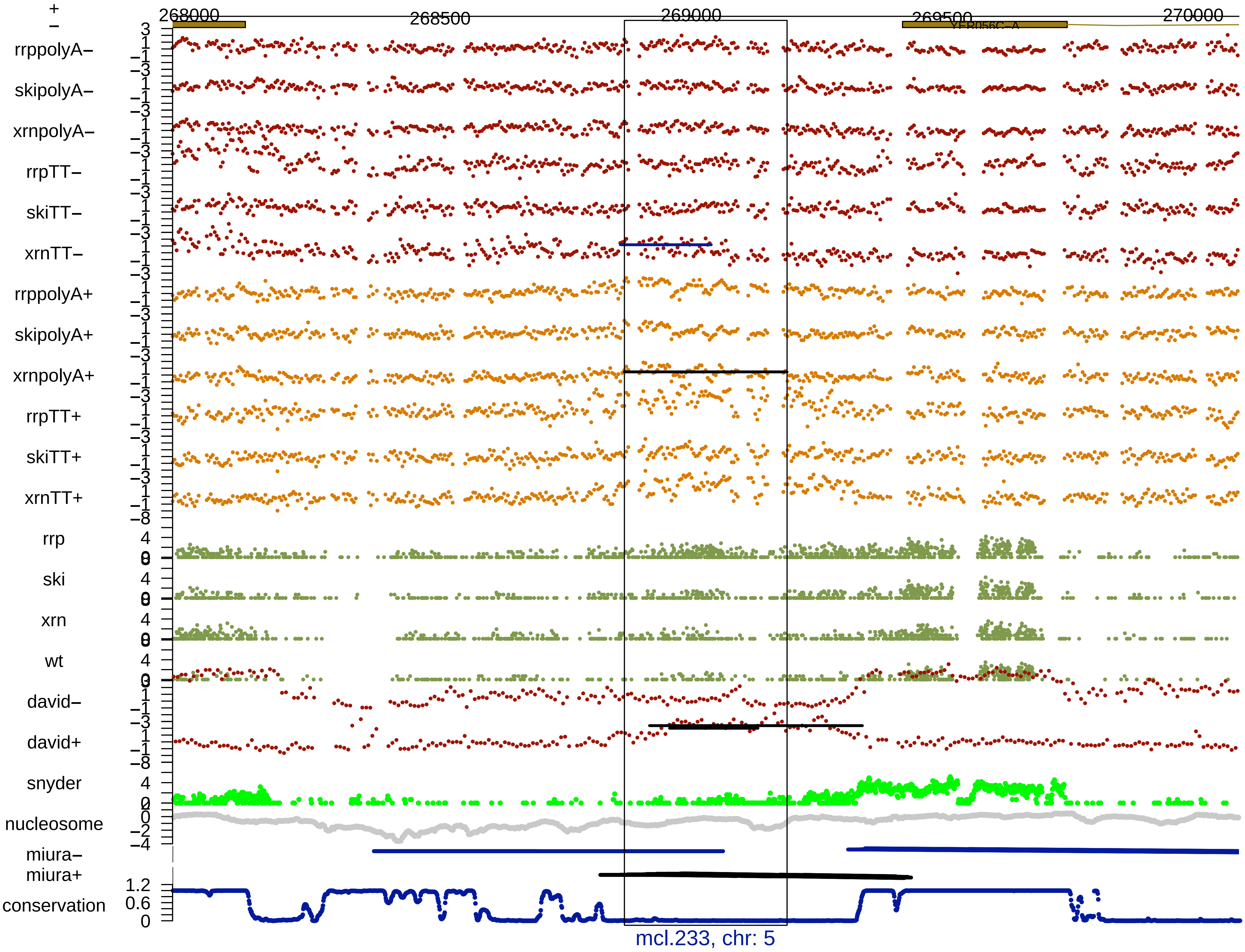
268000
268500
269000
269500
270000
YER056C-A
rrppolyA−
skipolyA−
xrnpolyA−
rrpTT−
skiTT−
xrnTT−
rrppolyA+
skipolyA+
xrnpolyA+
rrpTT+
skiTT+
xrnTT+
rrp
ski
xrn
wt
david−
david+
snyder
nucleosome
miura−
miura+
conservation
mcl.233, chr: 5

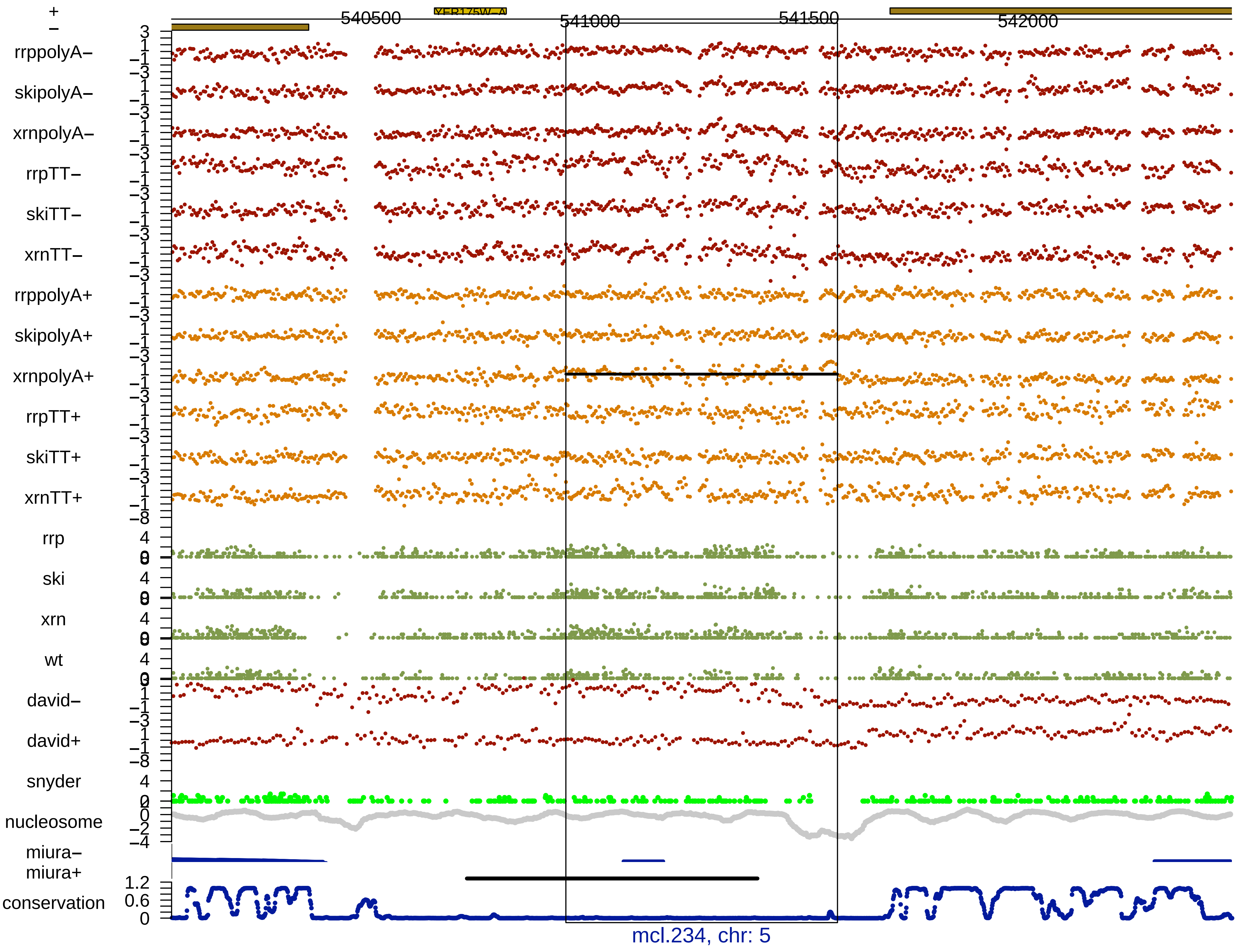

+
−
540500
541000
541500
542000
rrppolyA−
skipolyA−
xrnpolyA−
rrpTT−
skiTT−
xrnTT−
rrppolyA+
skipolyA+
xrnpolyA+
rrpTT+
skiTT+
xrnTT+
rrp
ski
xrn
wt
david−
david+
snyder
nucleosome
miura−
miura+
conservation
mcl.234, chr: 5

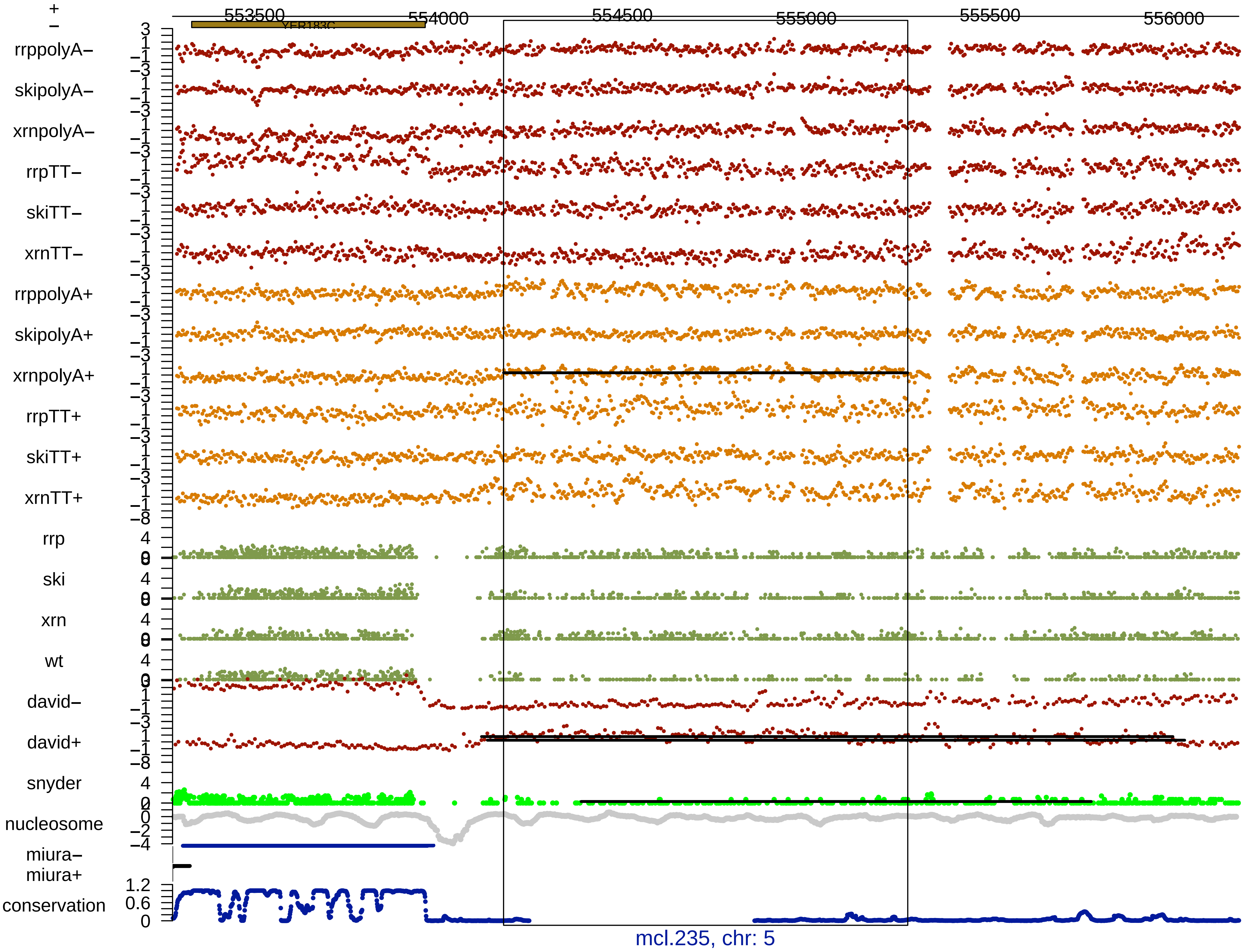

553500
554000
554500
555000
555500
556000
YER183C
rrppolyA−
skipolyA−
xrnpolyA−
rrpTT−
skiTT−
xrnTT−
rrppolyA+
skipolyA+
xrnpolyA+
rrpTT+
skiTT+
xrnTT+
rrp
ski
xrn
wt
david−
david+
snyder
nucleosome
miura−
miura+
conservation
mcl.235, chr: 5

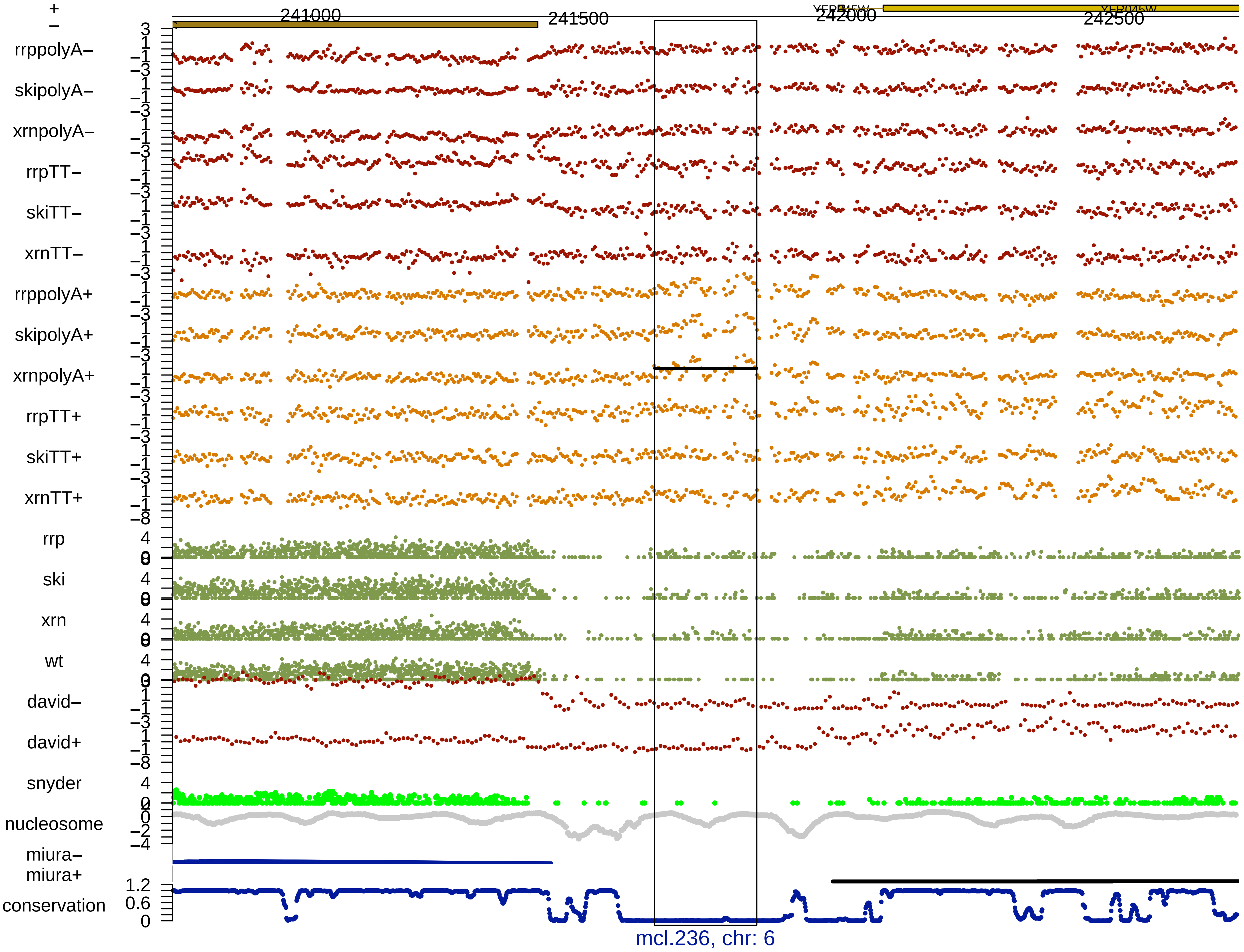

+
−
241000
241500
242000
242500
YFR045W
rrppolyA−
skipolyA−
xrnpolyA−
rrpTT−
skiTT−
xrnTT−
rrppolyA+
skipolyA+
xrnpolyA+
rrpTT+
skiTT+
xrnTT+
rrp
ski
xrn
wt
david−
david+
snyder
nucleosome
miura−
miura+
conservation
mcl.236, chr: 6

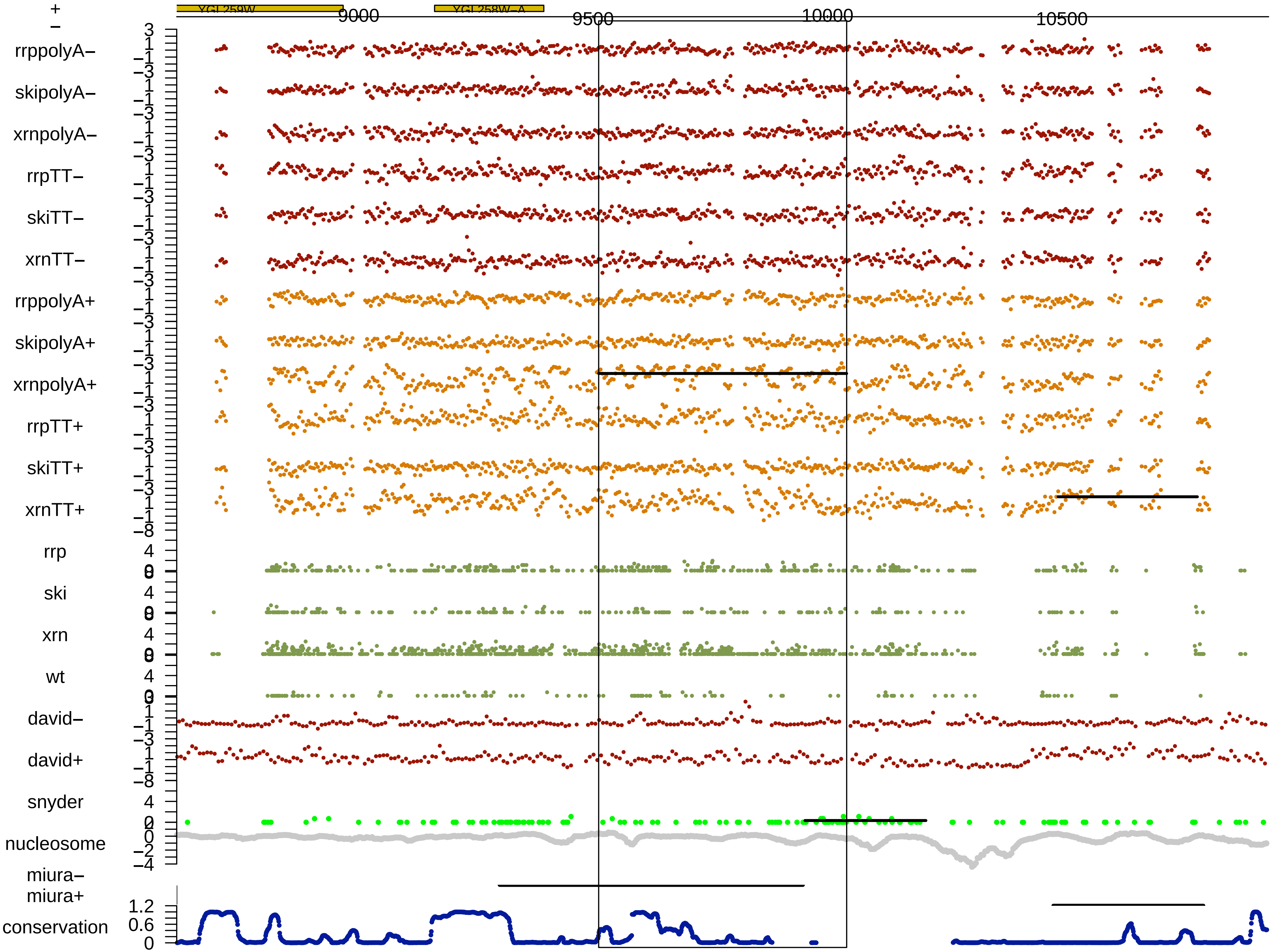

YGL259W
YGL258W-A
9000
9500
10000
10500
+
−
rrppolyA−
skipolyA−
xrnpolyA−
rrpTT−
skiTT−
xrnTT−
rrppolyA+
skipolyA+
xrnpolyA+
rrpTT+
skiTT+
xrnTT+
rrp
ski
xrn
wt
david−
david+
snyder
nucleosome
miura−
miura+
conservation
1.2
0.6
0

mcl.237, chr: 7

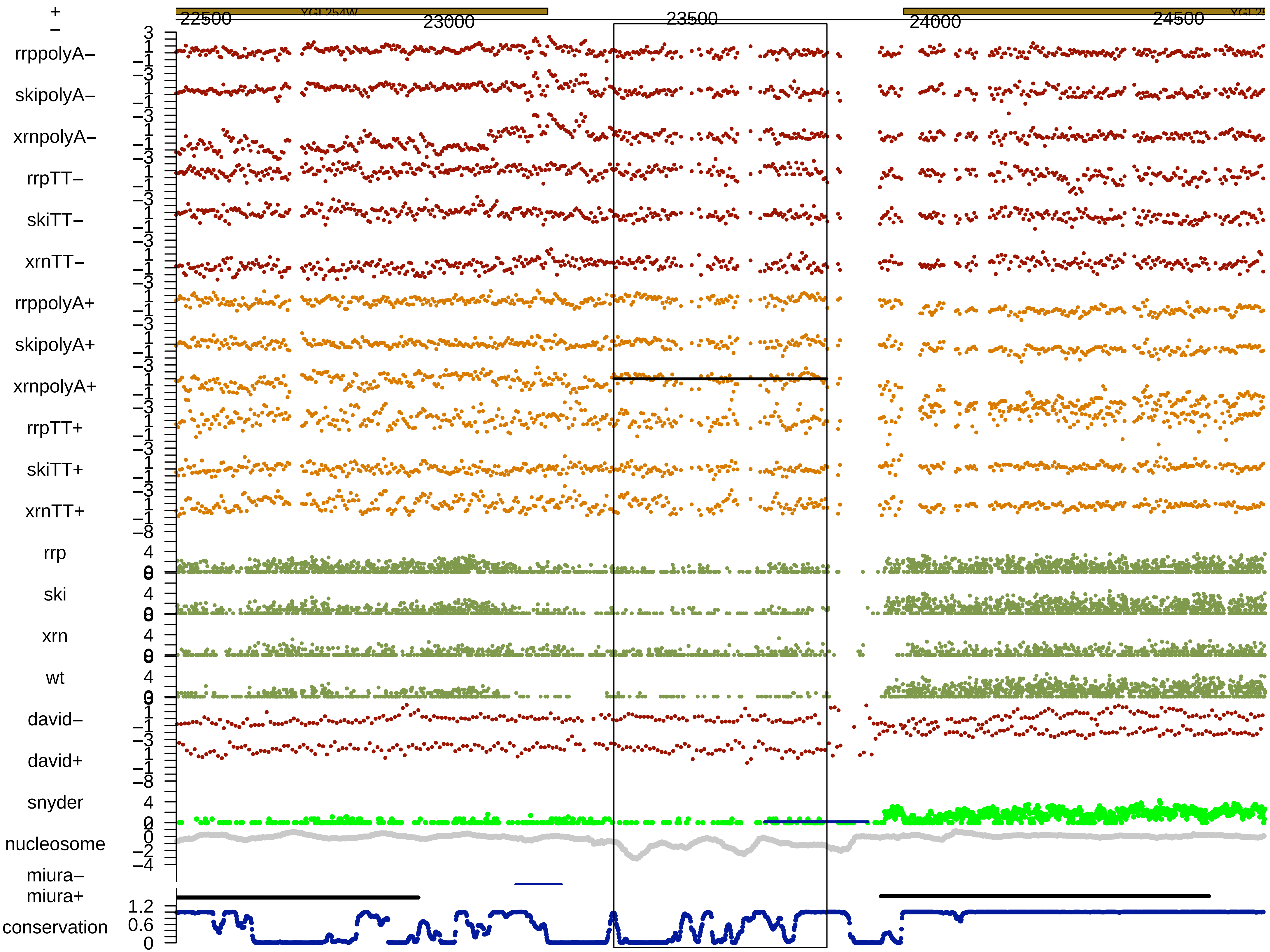

YGL254W
22500
23000
23500
24000
24500
+
−
rrppolyA−
skipolyA−
xrnpolyA−
rrpTT−
skiTT−
xrnTT−
rrppolyA+
skipolyA+
xrnpolyA+
rrpTT+
skiTT+
xrnTT+
rrp
ski
xrn
wt
david−
david+
snyder
nucleosome
miura−
miura+
conservation
1.2
0.6
0

mcl.238, chr: 7

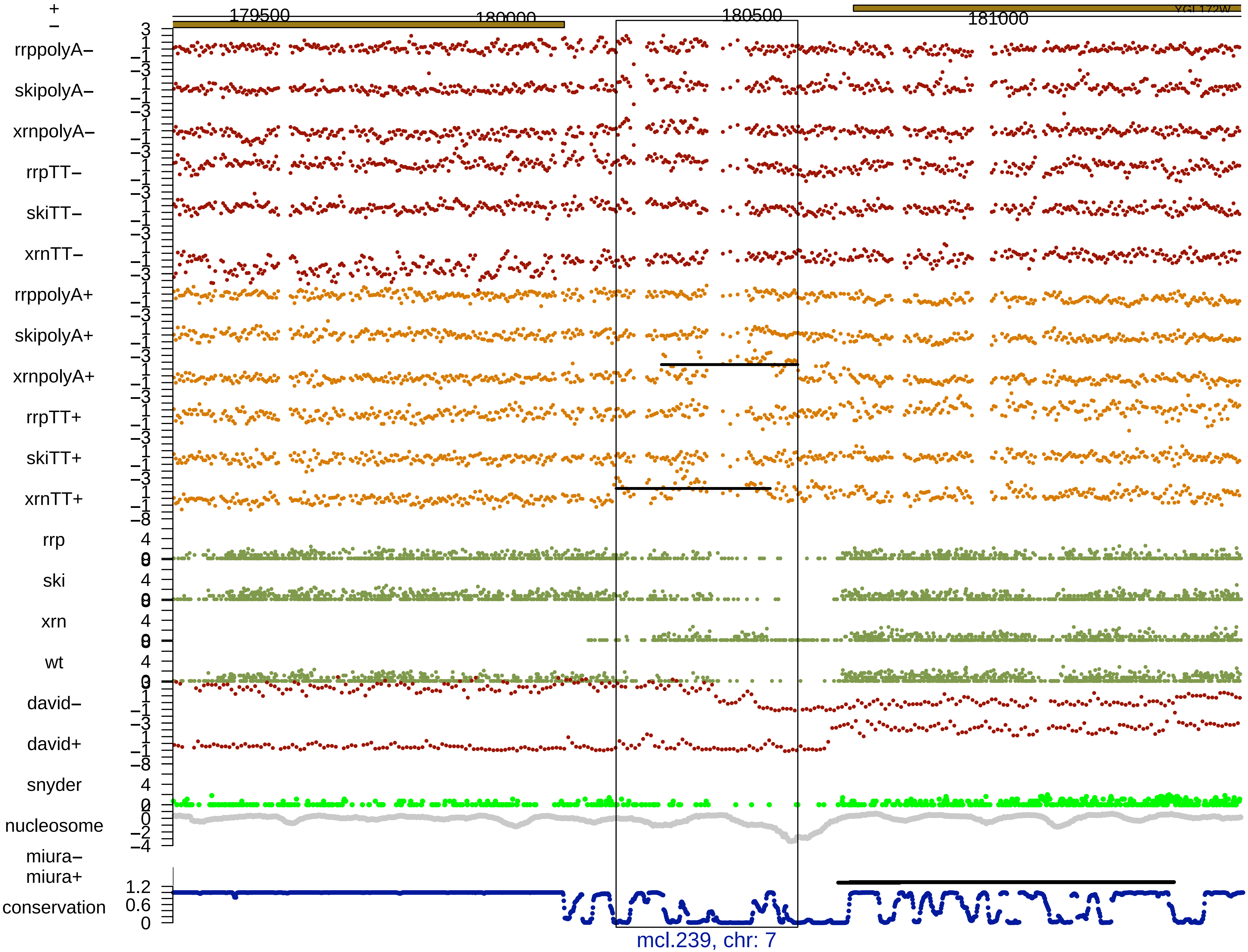

+
–
179500
180000
180500
181000
YGL172W
rrppolyA–
skipolyA–
xrnpolyA–
rrpTT–
skiTT–
xrnTT–
rrppolyA+
skipolyA+
xrnpolyA+
rrpTT+
skiTT+
xrnTT+
rrp
ski
xrn
wt
david–
david+
snyder
nucleosome
miura–
miura+
conservation
mcl.239, chr: 7

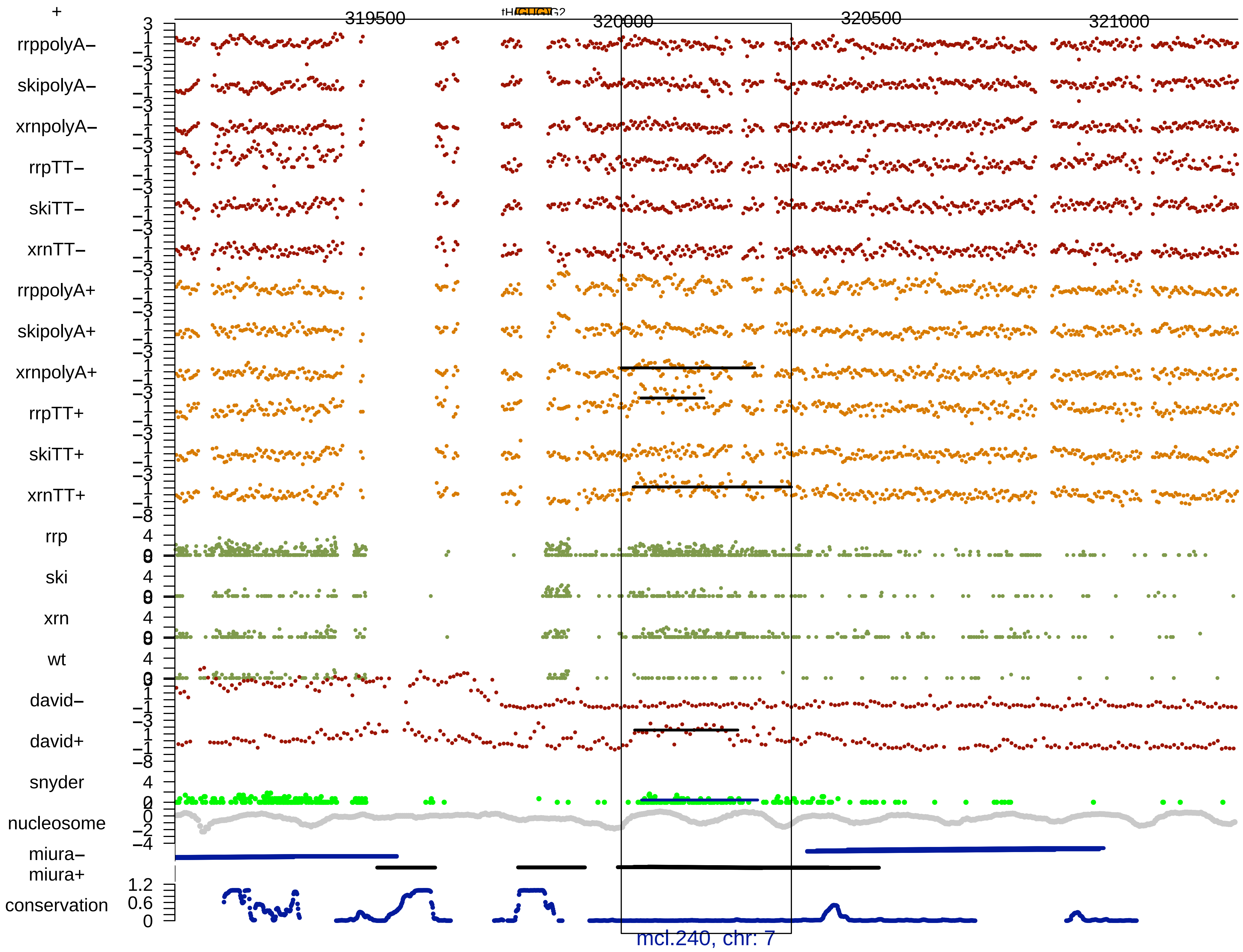

+
319500
320000
320500
321000
rrppolyA−
skipolyA−
xrnpolyA−
rrpTT−
skiTT−
xrnTT−
rrppolyA+
skipolyA+
xrnpolyA+
rrpTT+
skiTT+
xrnTT+
rrp
ski
xrn
wt
david−
david+
snyder
nucleosome
miura−
miura+
conservation
mcl.240, chr: 7

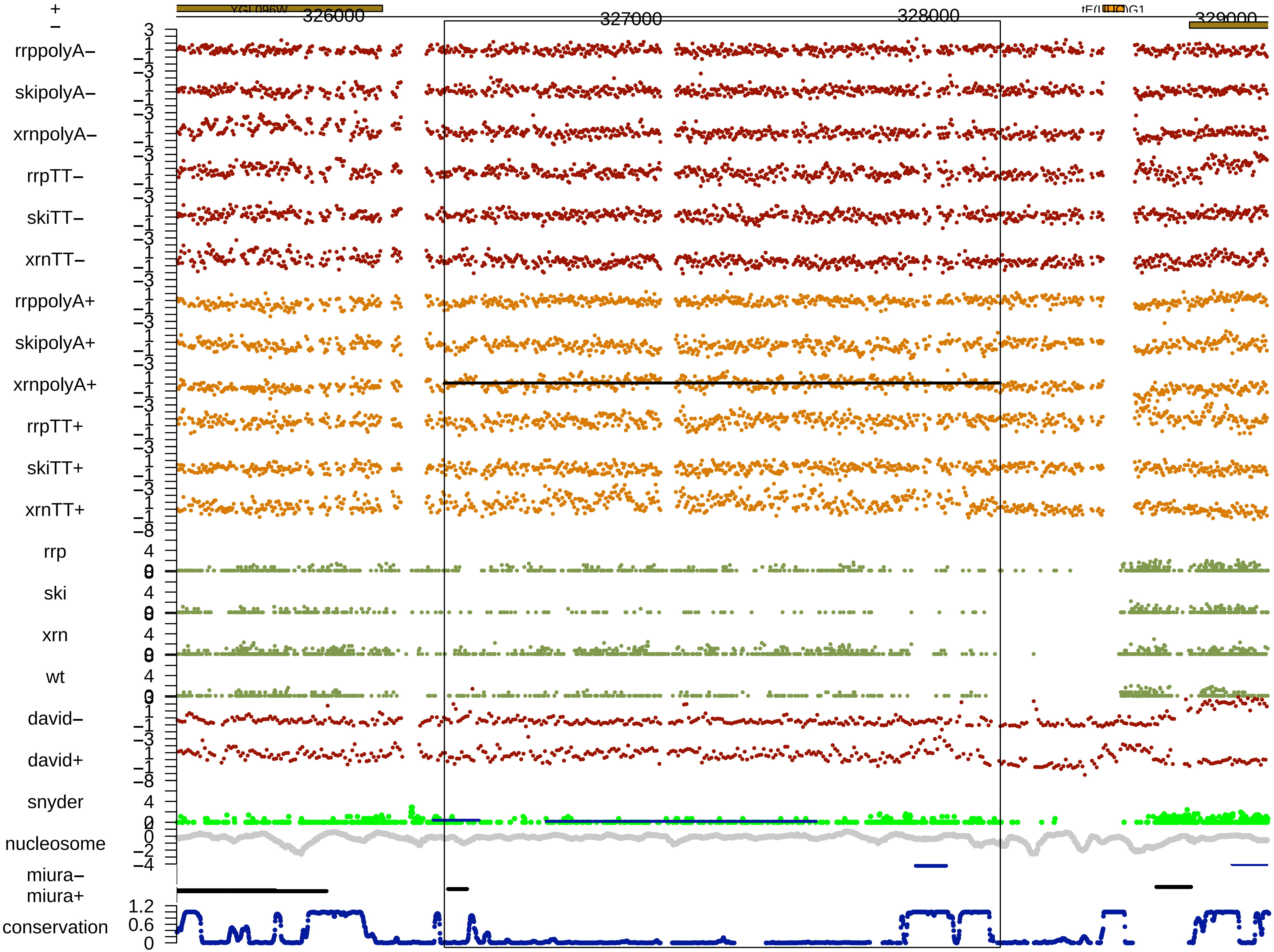
326000
327000
328000
329000
+
−
rrppolyA−
skipolyA−
xrnpolyA−
rrpTT−
skiTT−
xrnTT−
rrppolyA+
skipolyA+
xrnpolyA+
rrpTT+
skiTT+
xrnTT+
rrp
ski
xrn
wt
david−
david+
snyder
nucleosome
miura−
miura+
conservation

mcl.241, chr: 7

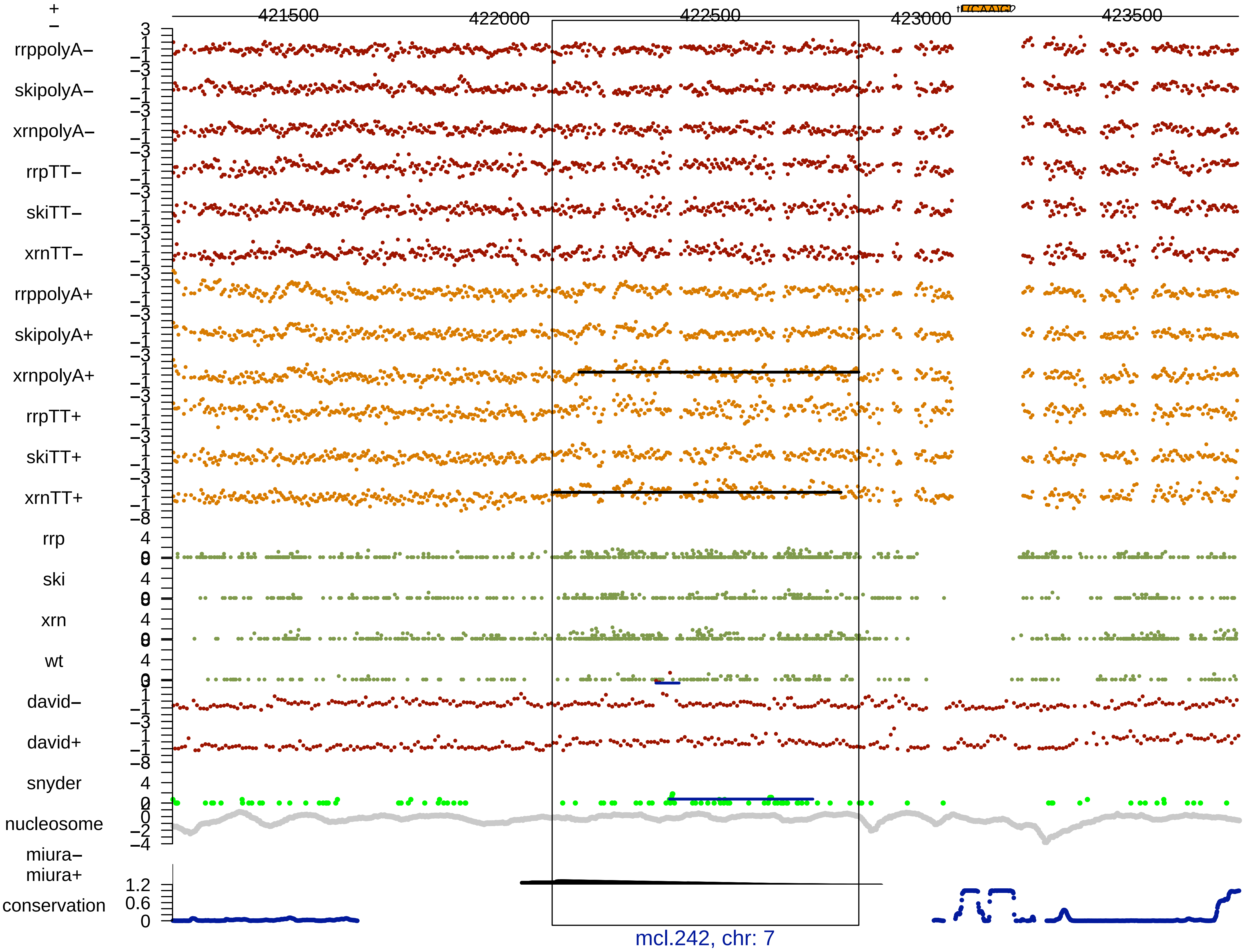

421500
422000
422500
423000
423500
rrppolyA−
skipolyA−
xrnpolyA−
rrpTT−
skiTT−
xrnTT−
rrppolyA+
skipolyA+
xrnpolyA+
rrpTT+
skiTT+
xrnTT+
rrp
ski
xrn
wt
david−
david+
snyder
nucleosome
miura−
miura+
conservation
mcl.242, chr: 7

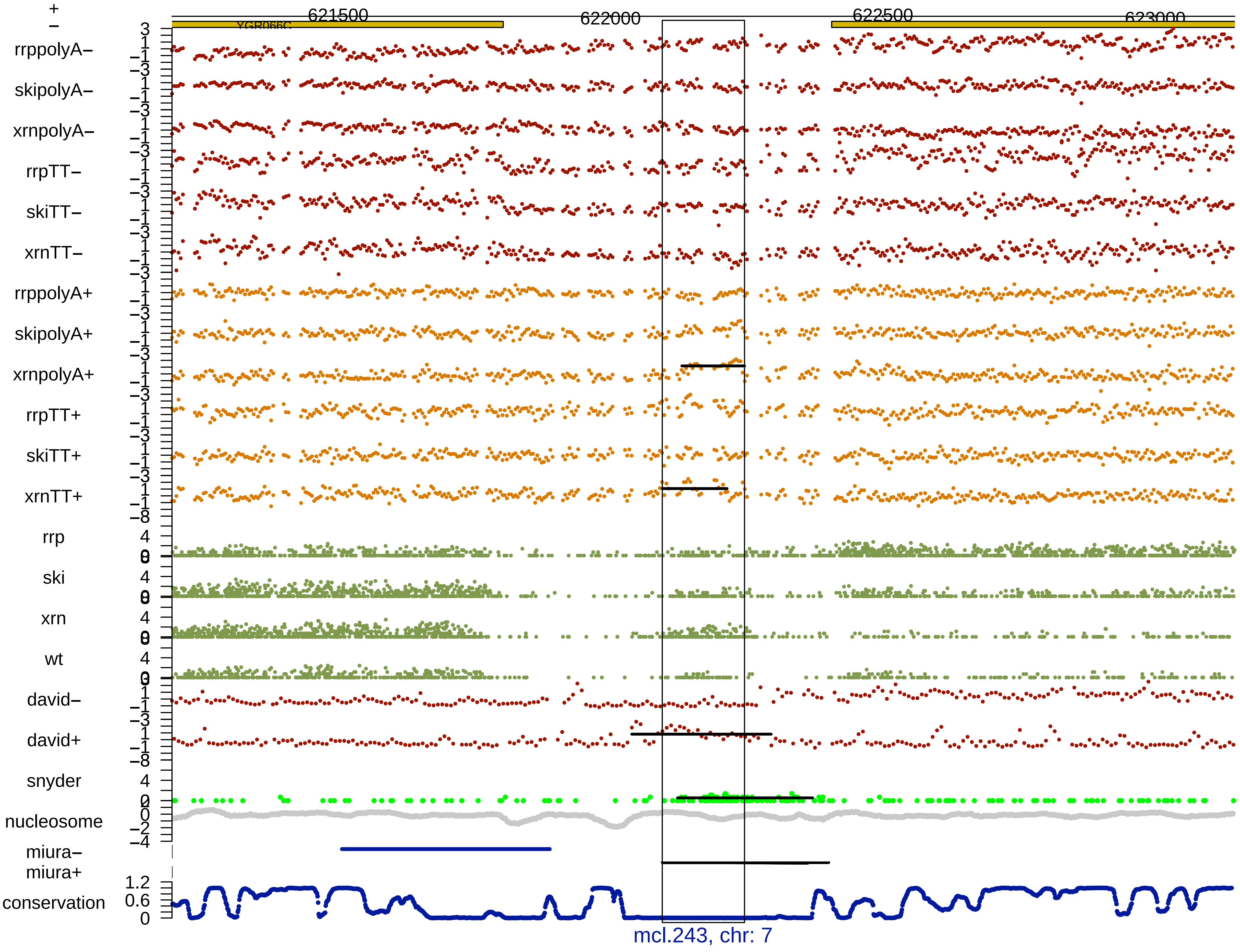

621500
622000
622500
623000
YGR066C
rrppolyA−
skipolyA−
xrnpolyA−
rrpTT−
skiTT−
xrnTT−
rrppolyA+
skipolyA+
xrnpolyA+
rrpTT+
skiTT+
xrnTT+
rrp
ski
xrn
wt
david−
david+
snyder
nucleosome
miura−
miura+
conservation
mcl.243, chr: 7

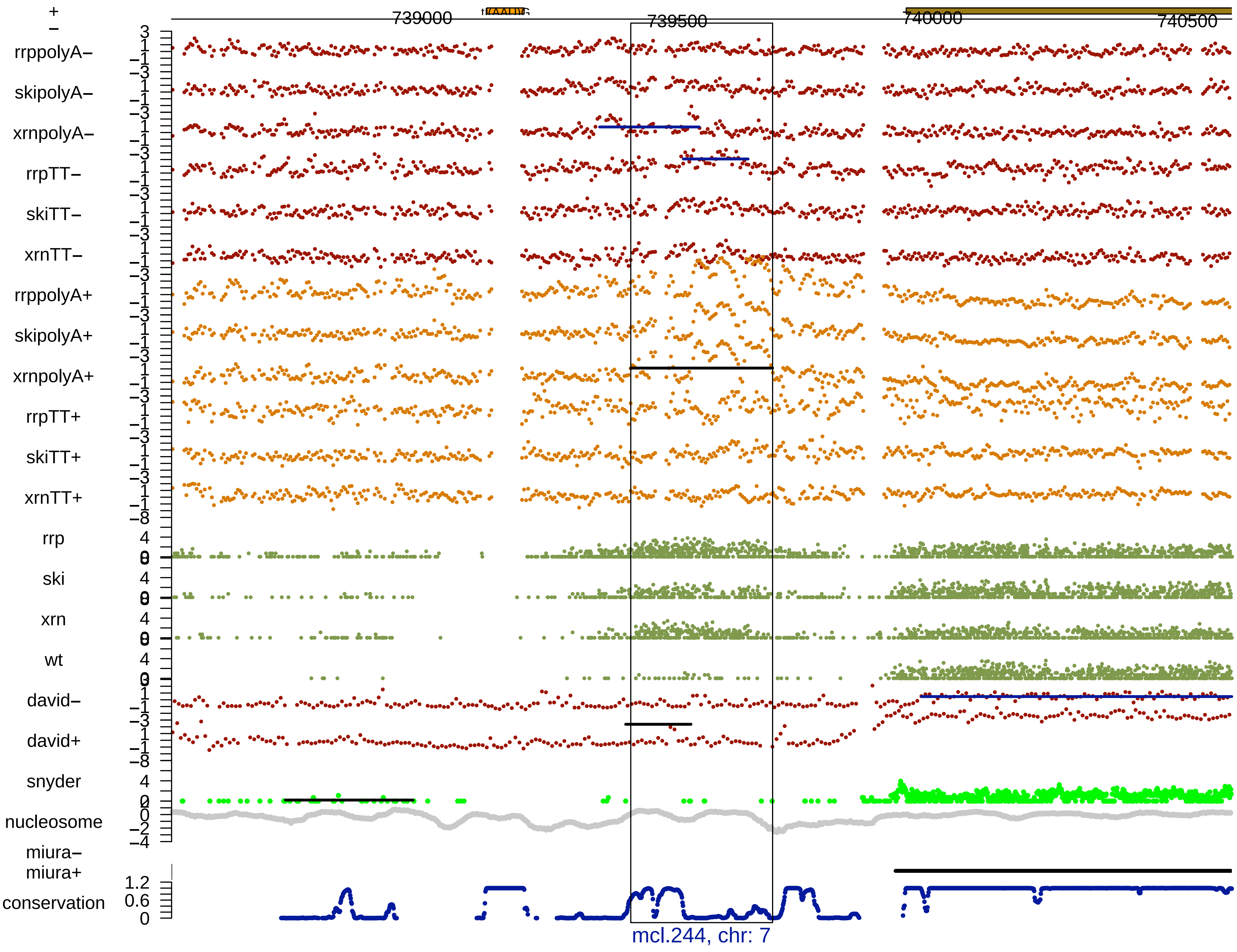

739000
739500
740000
740500
rrppolyA−
skipolyA−
xrnpolyA−
rrpTT−
skiTT−
xrnTT−
rrppolyA+
skipolyA+
xrnpolyA+
rrpTT+
skiTT+
xrnTT+
rrp
ski
xrn
wt
david−
david+
snyder
nucleosome
miura−
miura+
conservation
mcl.244, chr: 7

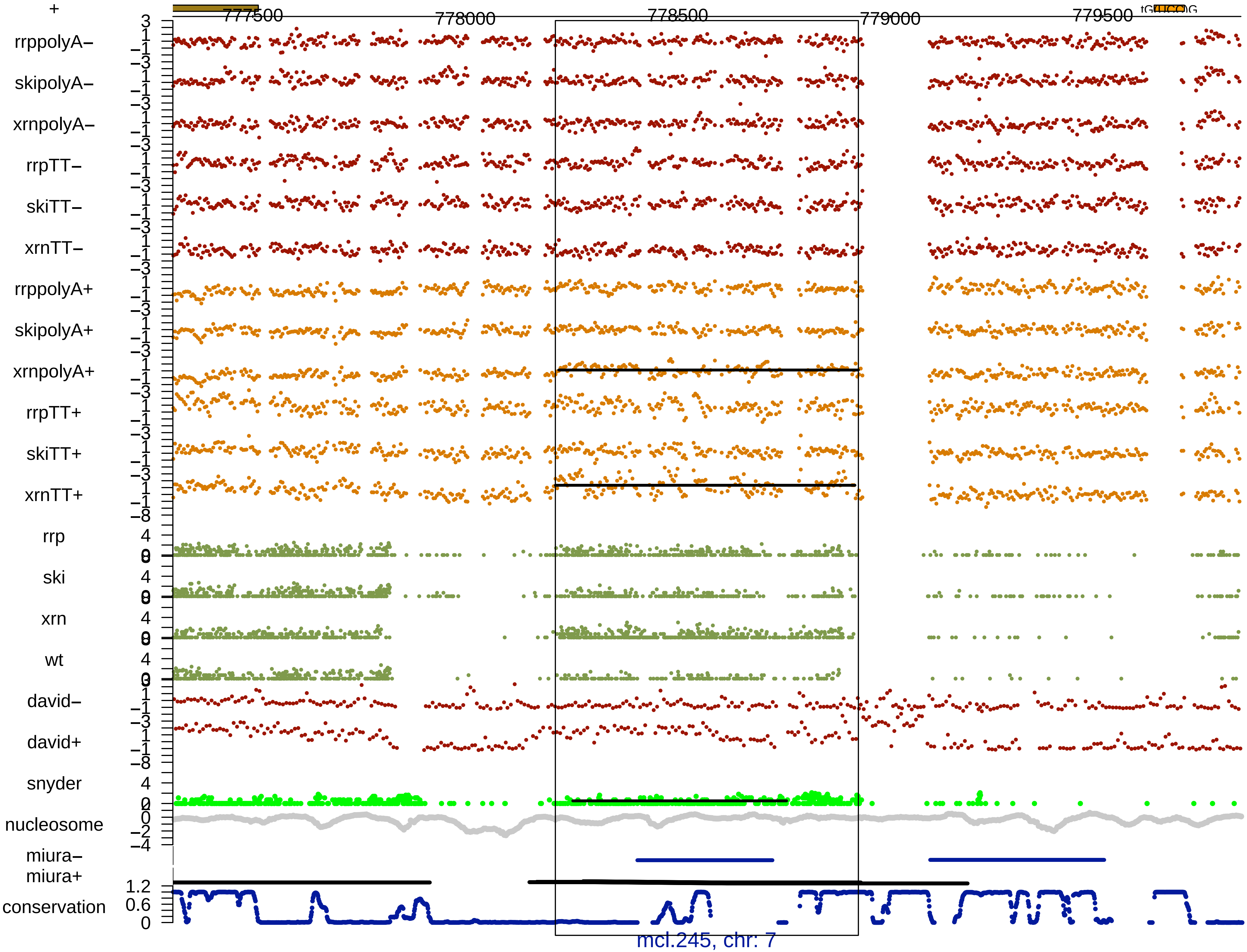

+
777500
778000
778500
779000
779500
rrppolyA−
skipolyA−
xrnpolyA−
rrpTT−
skiTT−
xrnTT−
rrppolyA+
skipolyA+
xrnpolyA+
rrpTT+
skiTT+
xrnTT+
rrp
ski
xrn
wt
david−
david+
snyder
nucleosome
miura−
miura+
conservation
mcl.245, chr: 7

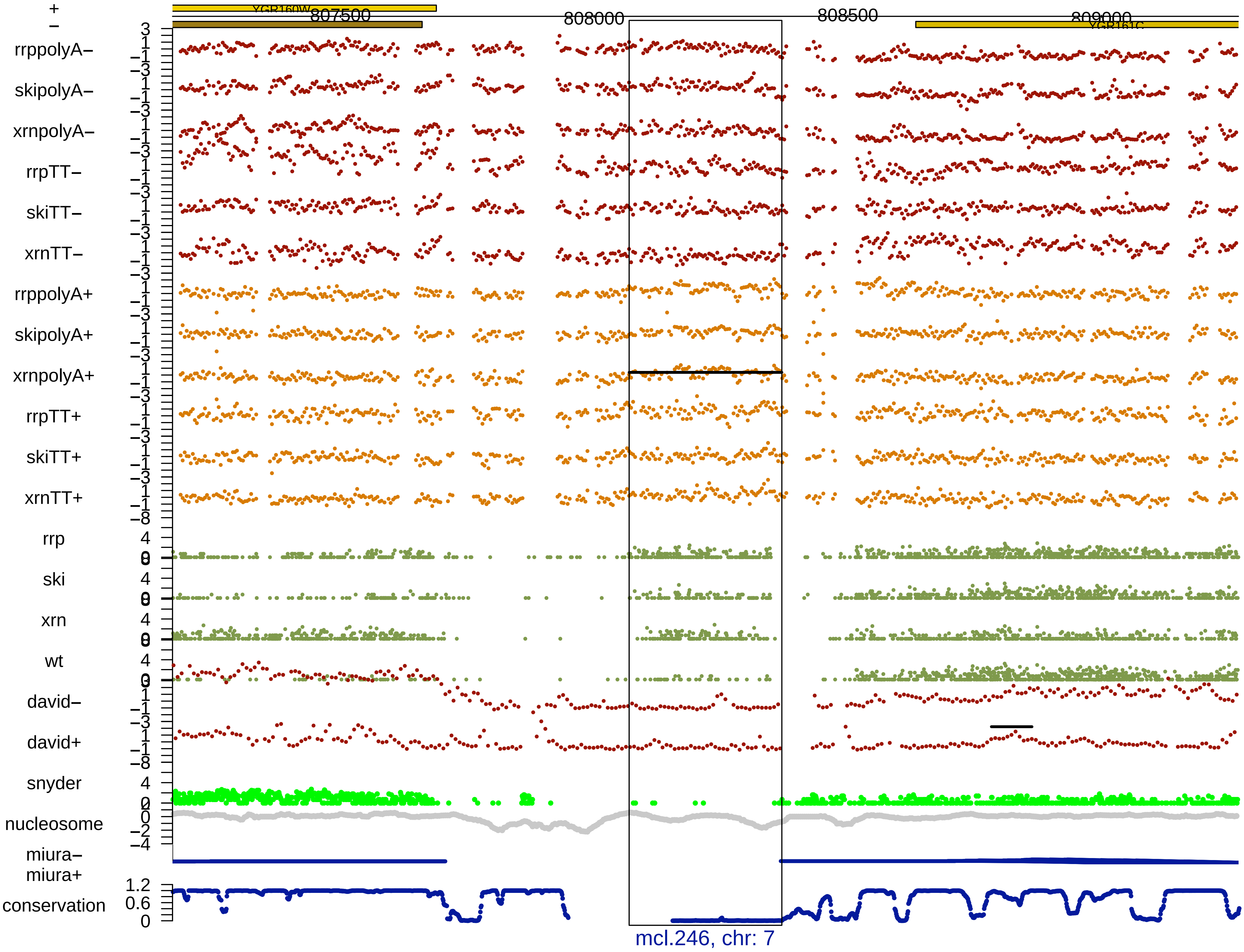

YGR160W
807500
808000
808500
809000
YGR161C
rrppolyA−
skipolyA−
xrnpolyA−
rrpTT−
skiTT−
xrnTT−
rrppolyA+
skipolyA+
xrnpolyA+
rrpTT+
skiTT+
xrnTT+
rrp
ski
xrn
wt
david−
david+
snyder
nucleosome
miura−
miura+
conservation
mcl.246, chr: 7

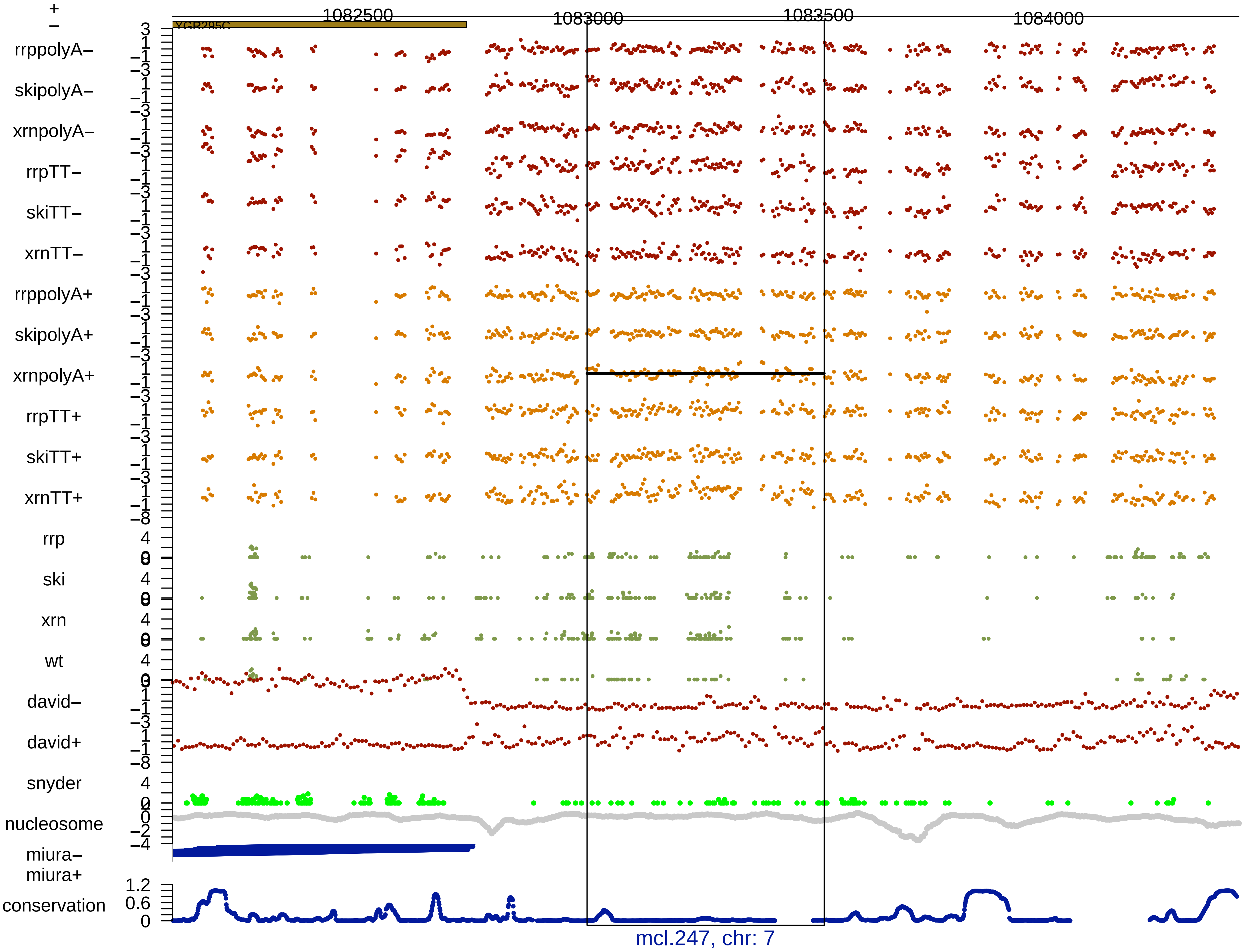

1082500
1083000
1083500
1084000
YGR295C
rrppolyA−
skipolyA−
xrnpolyA−
rrpTT−
skiTT−
xrnTT−
rrppolyA+
skipolyA+
xrnpolyA+
rrpTT+
skiTT+
xrnTT+
rrp
ski
xrn
wt
david−
david+
snyder
nucleosome
miura−
miura+
conservation
mcl.247, chr: 7

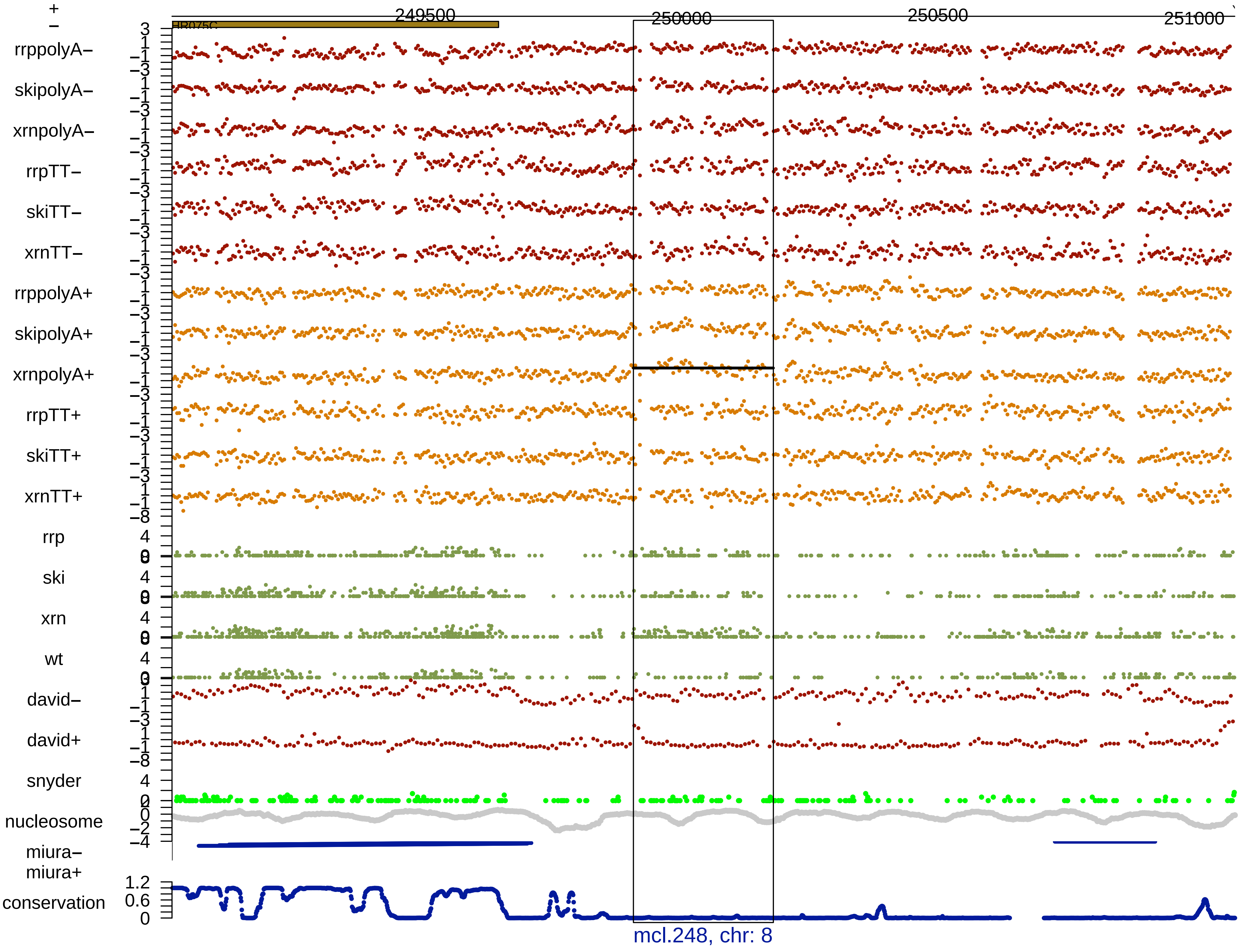

+
−
249500
250000
250500
251000
rrppolyA−
skipolyA−
xrnpolyA−
rrpTT−
skiTT−
xrnTT−
rrppolyA+
skipolyA+
xrnpolyA+
rrpTT+
skiTT+
xrnTT+
rrp
ski
xrn
wt
david−
david+
snyder
nucleosome
miura−
miura+
conservation
mcl.248, chr: 8

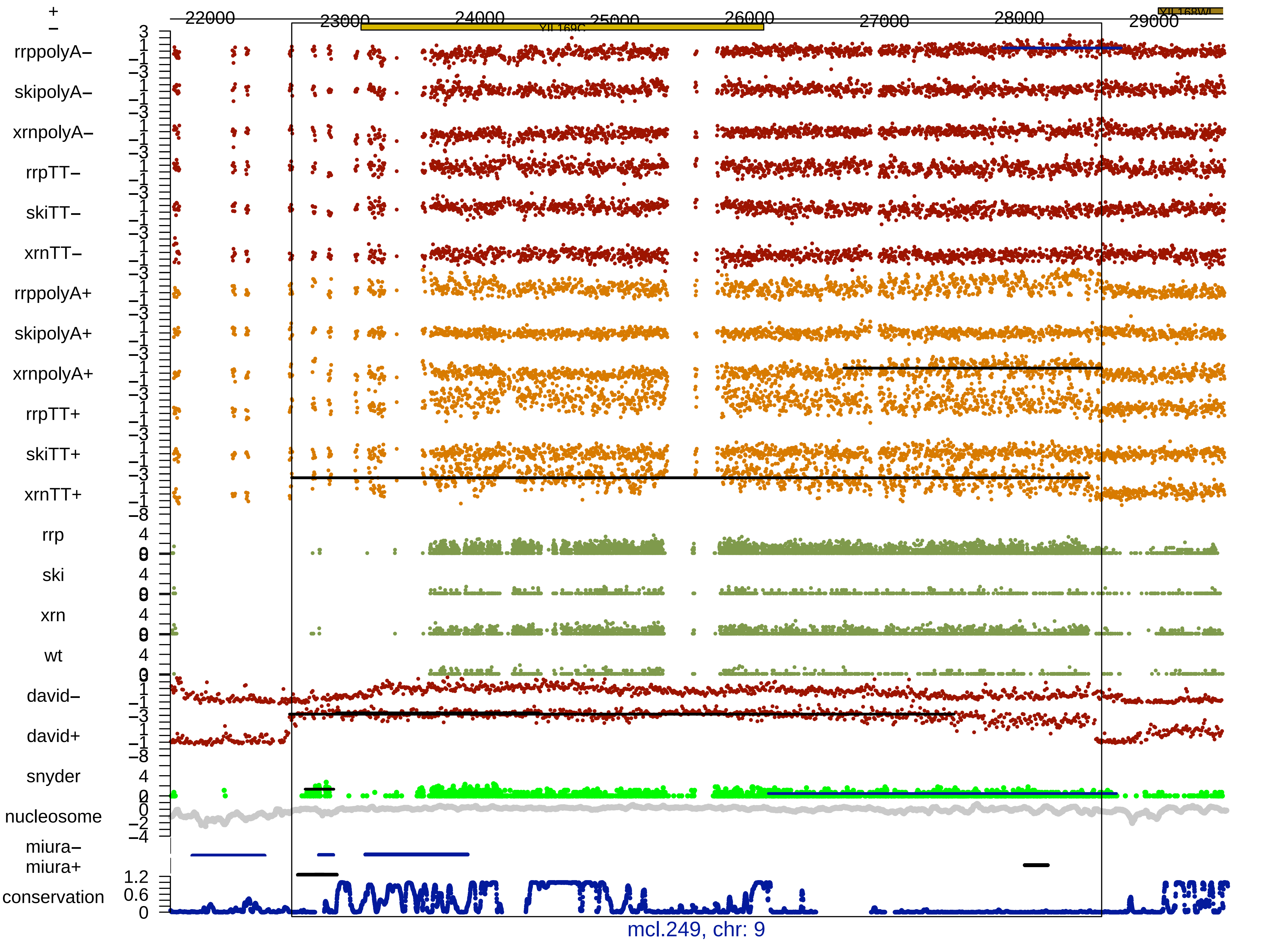

+
−
22000
23000
24000
25000
26000
27000
28000
29000
YIL169C
YIL168W
rrppolyA−
skipolyA−
xrnpolyA−
rrpTT−
skiTT−
xrnTT−
rrppolyA+
skipolyA+
xrnpolyA+
rrpTT+
skiTT+
xrnTT+
rrp
ski
xrn
wt
david−
david+
snyder
nucleosome
miura−
miura+
conservation
mcl.249, chr: 9

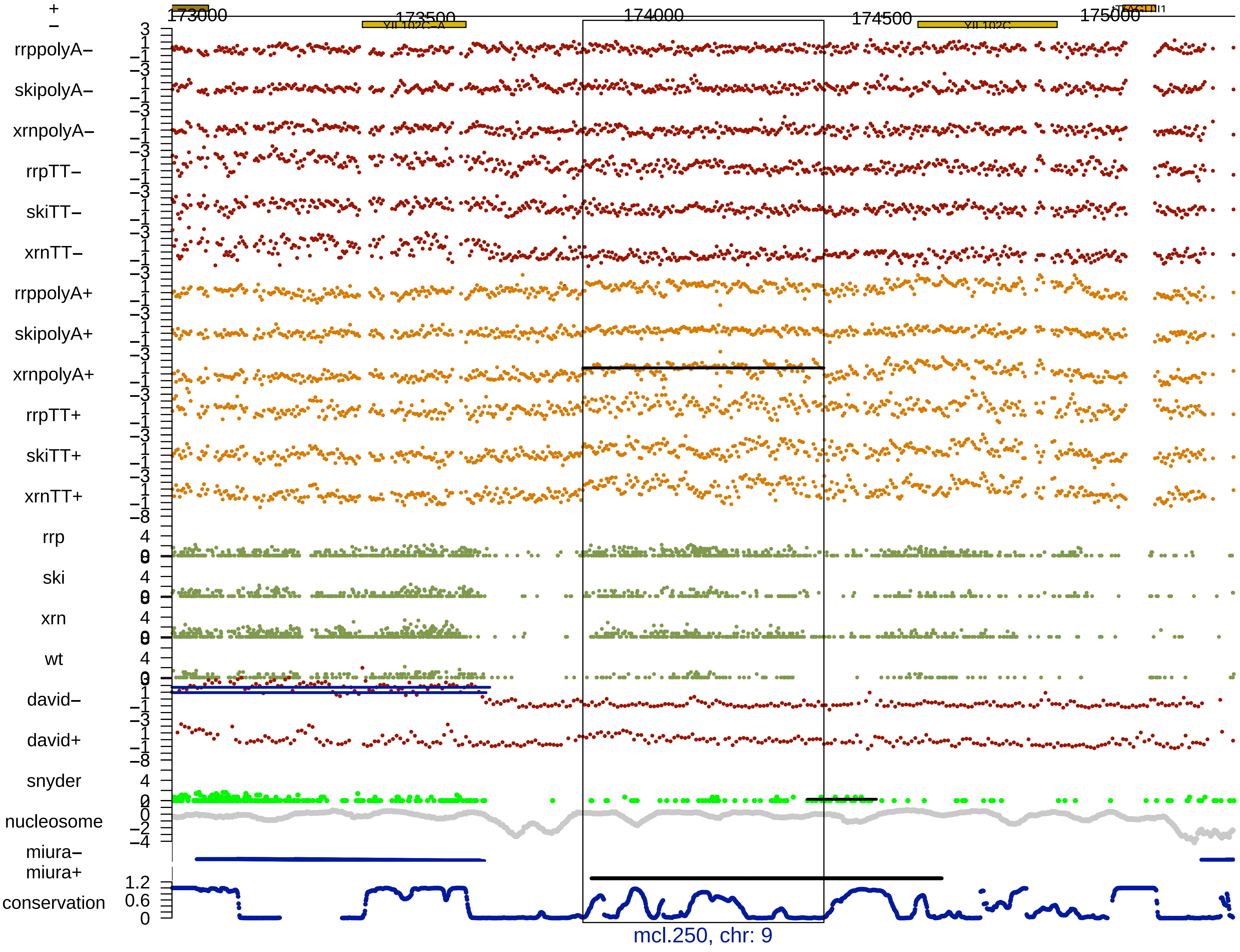

+
–
173000
173500
174000
174500
175000
rrppolyA–
skipolyA–
xrnpolyA–
rrpTT–
skiTT–
xrnTT–
rrppolyA+
skipolyA+
xrnpolyA+
rrpTT+
skiTT+
xrnTT+
rrp
ski
xrn
wt
david–
david+
snyder
nucleosome
miura–
miura+
conservation
1.2
0.6
0
mcl.250, chr: 9

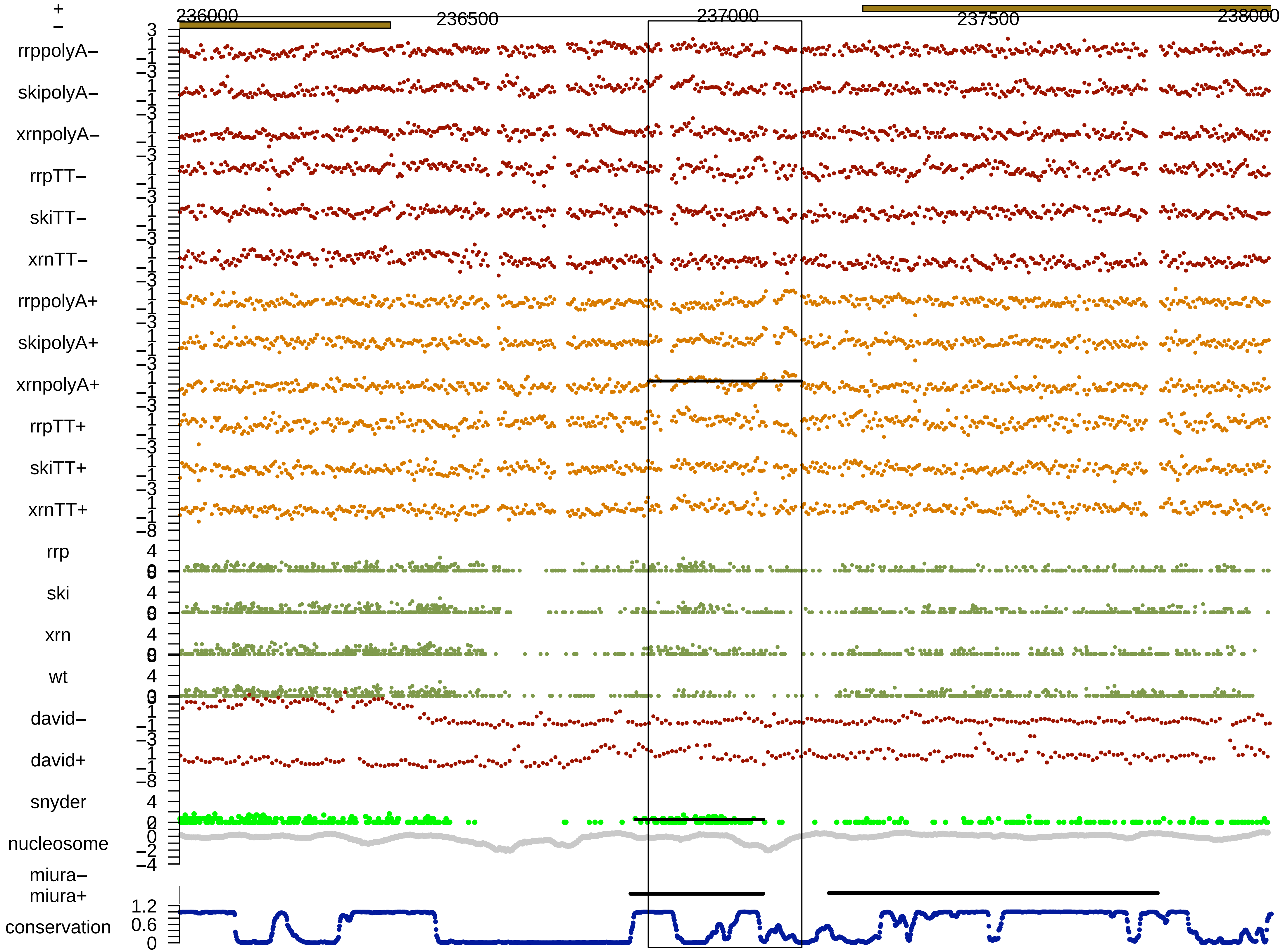

mcl.251, chr: 10

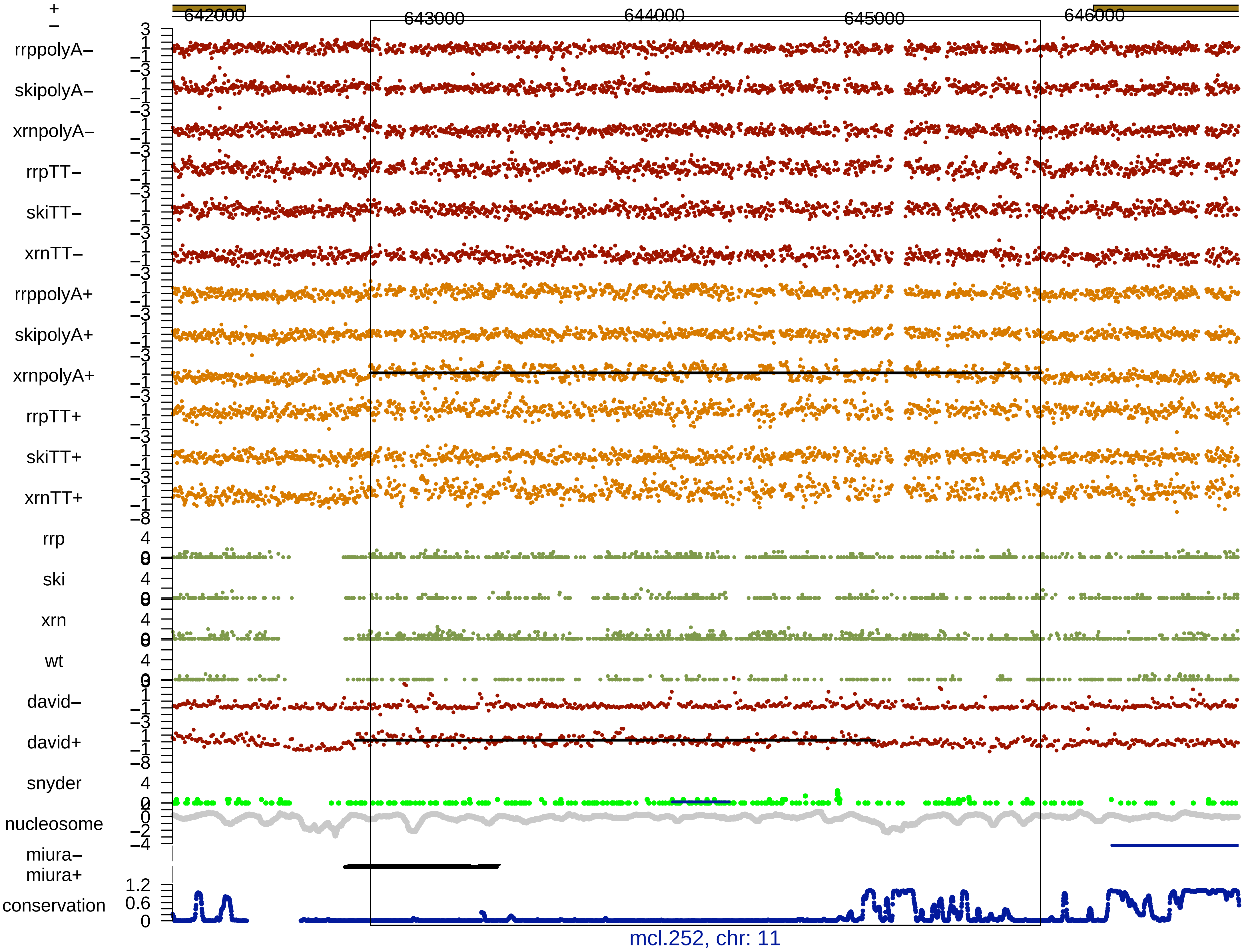
642000
643000
644000
645000
646000
rrppolyA−
skipolyA−
xrnpolyA−
rrpTT−
skiTT−
xrnTT−
rrppolyA+
skipolyA+
xrnpolyA+
rrpTT+
skiTT+
xrnTT+
rrp
ski
xrn
wt
david−
david+
snyder
nucleosome
miura−
miura+
conservation
mcl.252, chr: 11

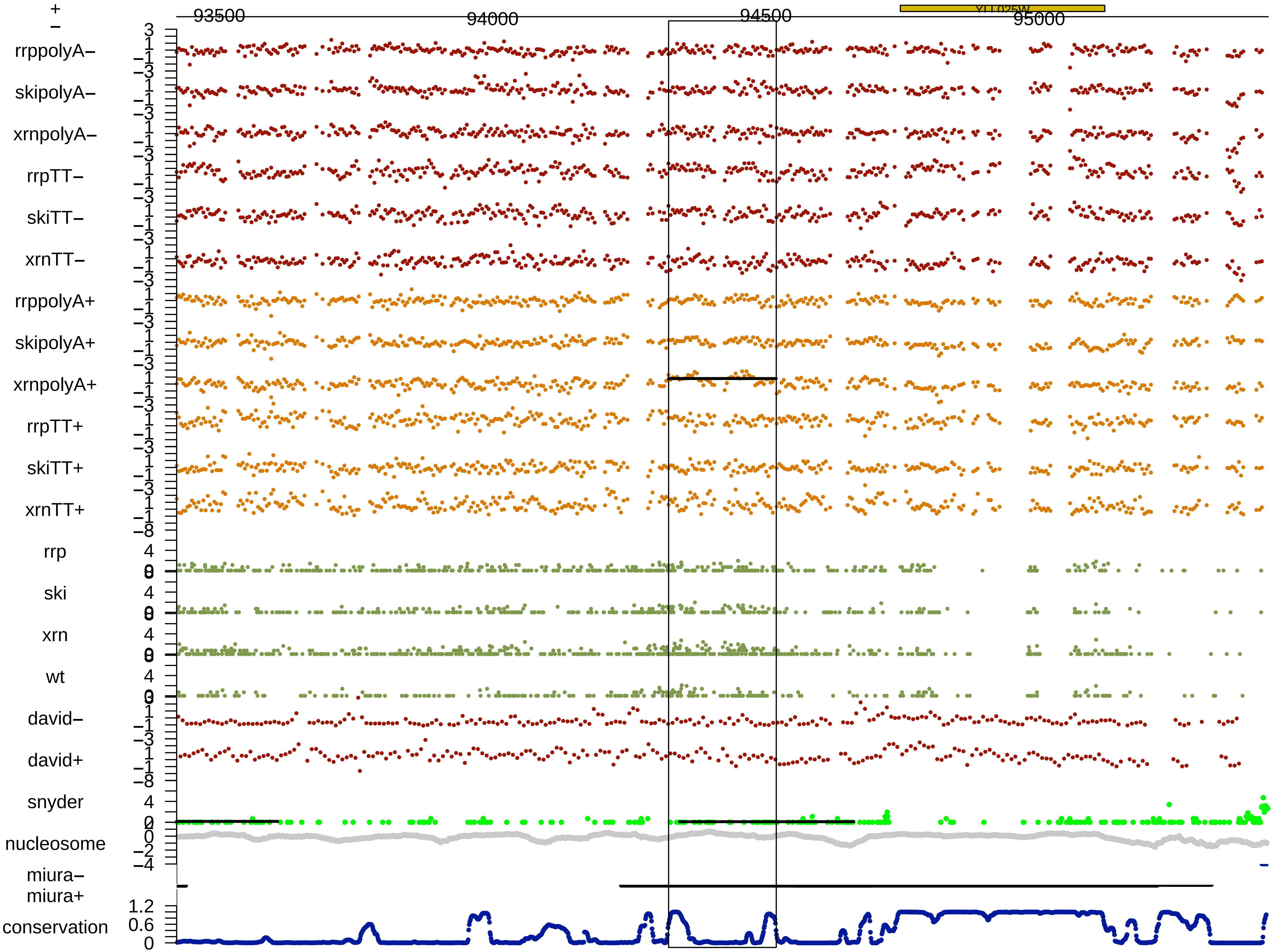

mcl.253, chr: 12

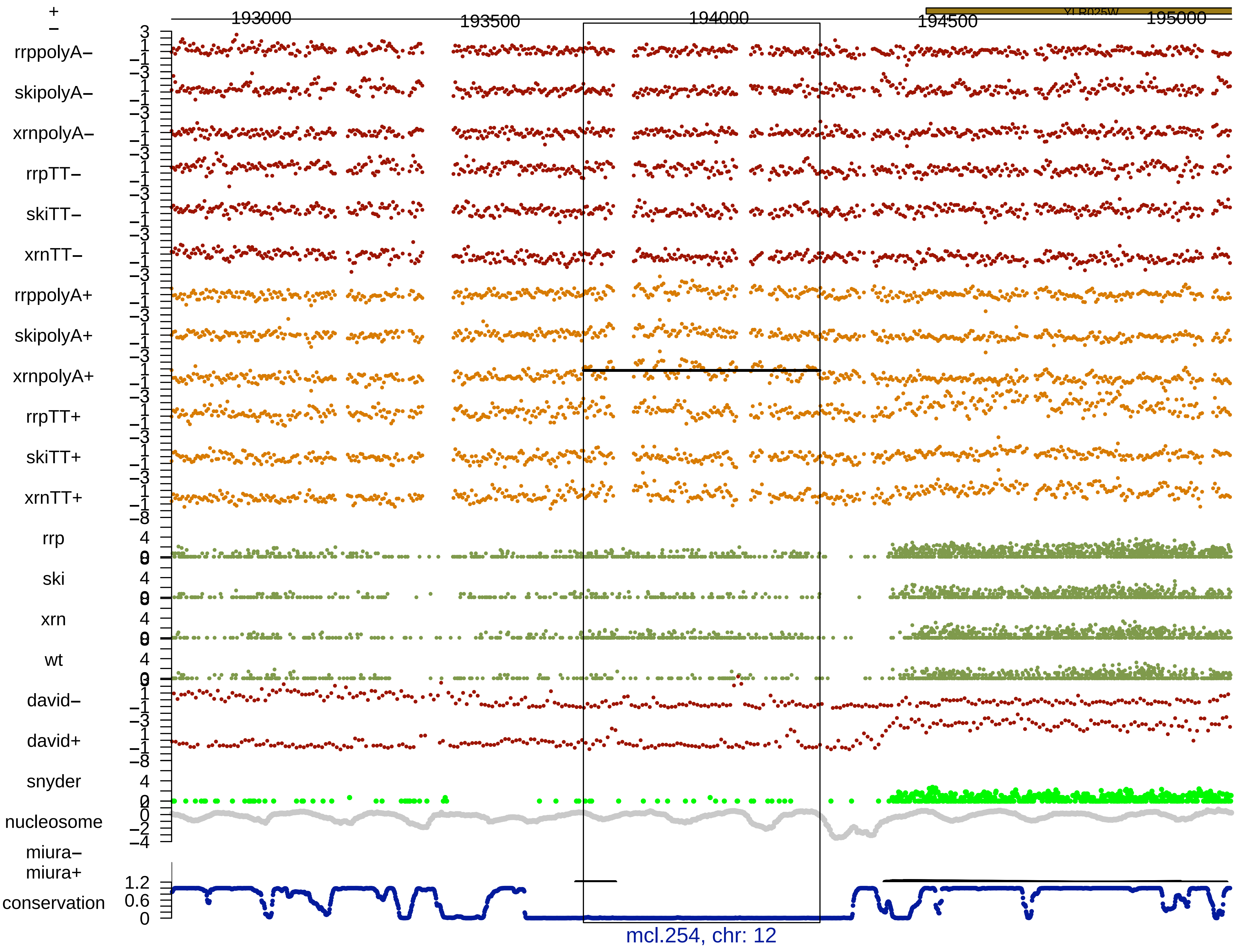

193000
193500
194000
194500
195000
YLR025W
rrppolyA−
skipolyA−
xrnpolyA−
rrpTT−
skiTT−
xrnTT−
rrppolyA+
skipolyA+
xrnpolyA+
rrpTT+
skiTT+
xrnTT+
rrp
ski
xrn
wt
david−
david+
snyder
nucleosome
miura−
miura+
conservation
mcl.254, chr: 12

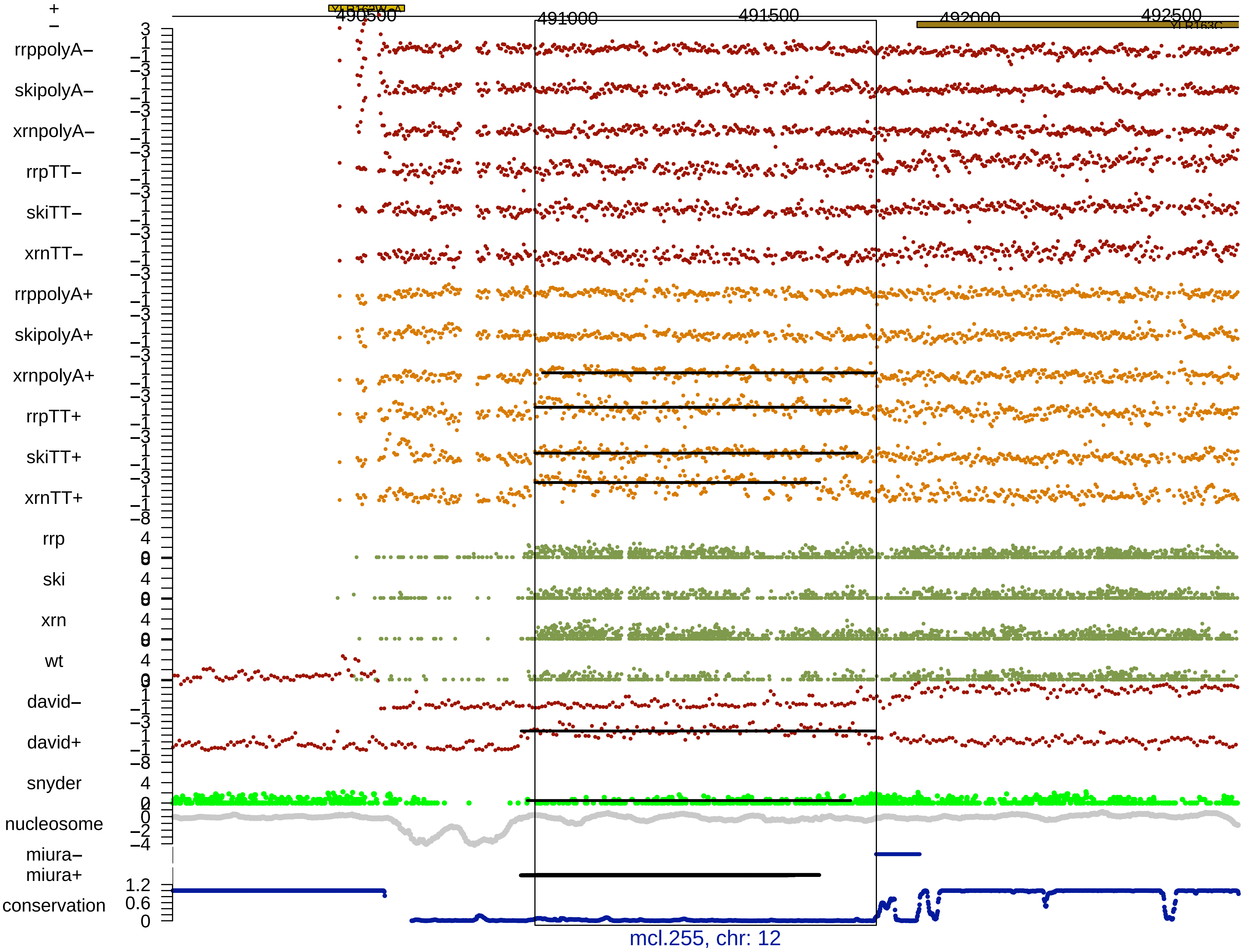

YLR162W-A
490500
491000
491500
492000
492500
YLR163C
rrppolyA−
skipolyA−
xrnpolyA−
rrpTT−
skiTT−
xrnTT−
rrppolyA+
skipolyA+
xrnpolyA+
rrpTT+
skiTT+
xrnTT+
rrp
ski
xrn
wt
david−
david+
snyder
nucleosome
miura−
miura+
conservation
mcl.255, chr: 12

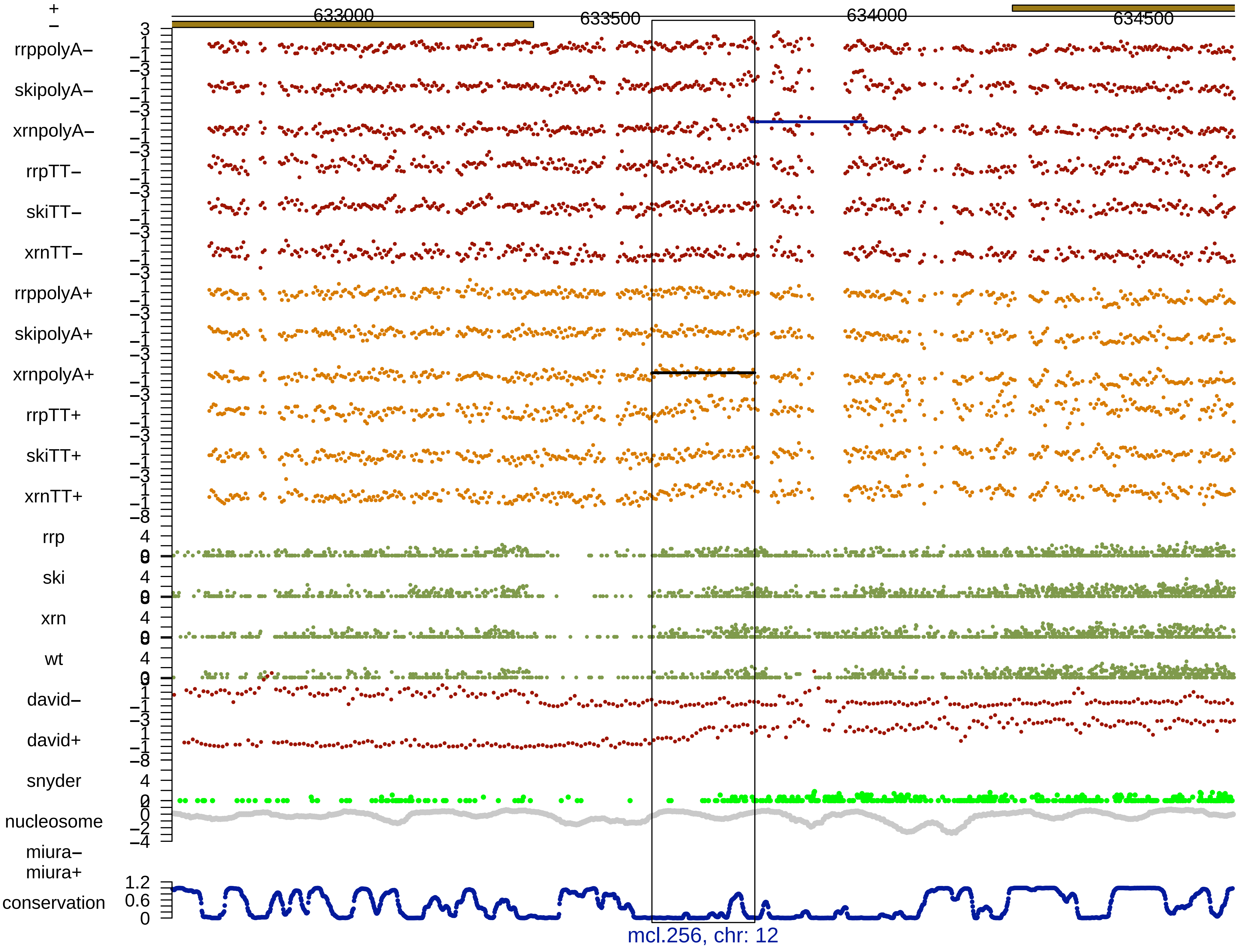

633000
633500
634000
634500
rrppolyA−
skipolyA−
xrnpolyA−
rrpTT−
skiTT−
xrnTT−
rrppolyA+
skipolyA+
xrnpolyA+
rrpTT+
skiTT+
xrnTT+
rrp
ski
xrn
wt
david−
david+
snyder
nucleosome
miura−
miura+
conservation
mcl.256, chr: 12

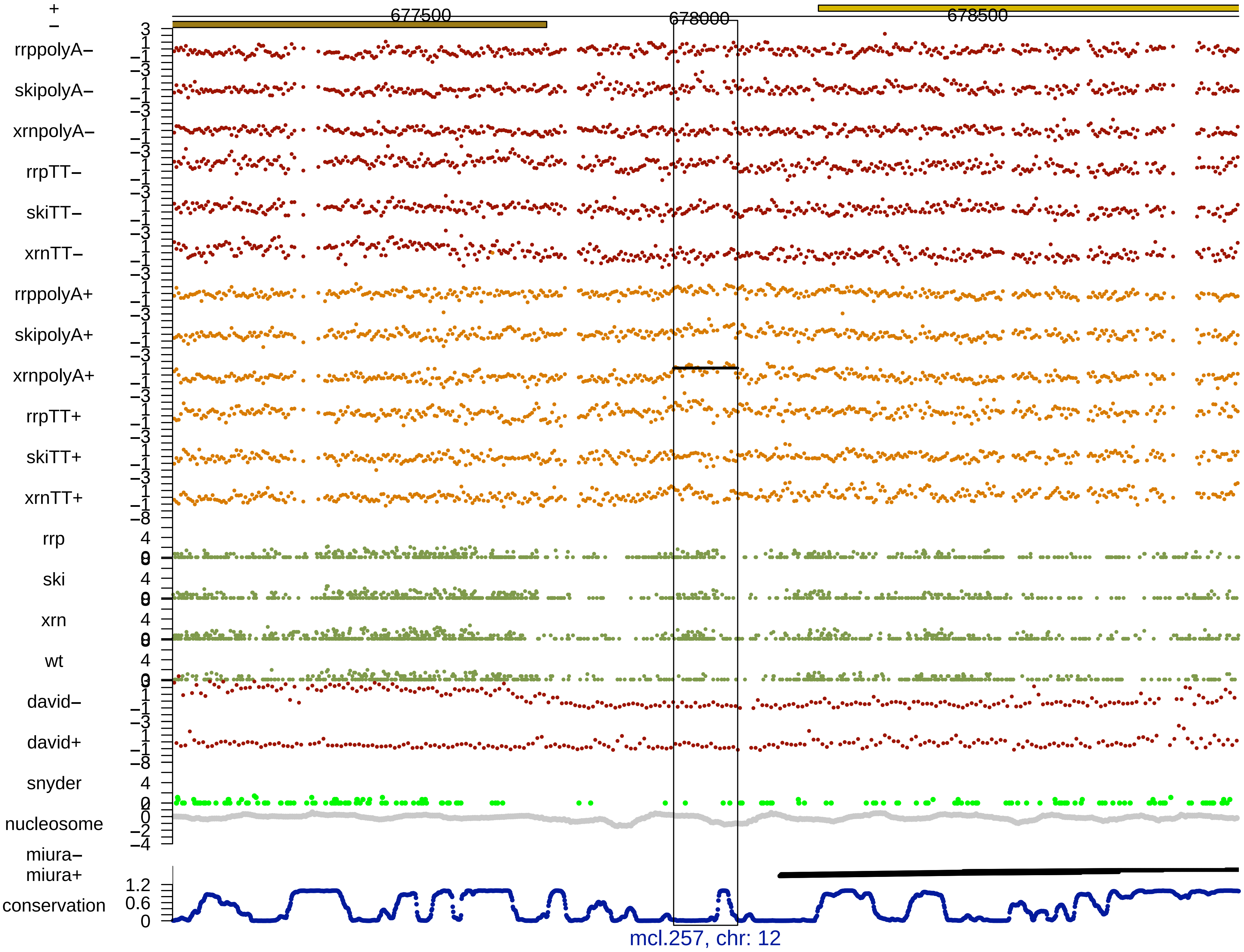

677500
678000
678500
rrppolyA−
skipolyA−
xrnpolyA−
rrpTT−
skiTT−
xrnTT−
rrppolyA+
skipolyA+
xrnpolyA+
rrpTT+
skiTT+
xrnTT+
rrp
ski
xrn
wt
david−
david+
snyder
nucleosome
miura−
miura+
conservation
mcl.257, chr: 12

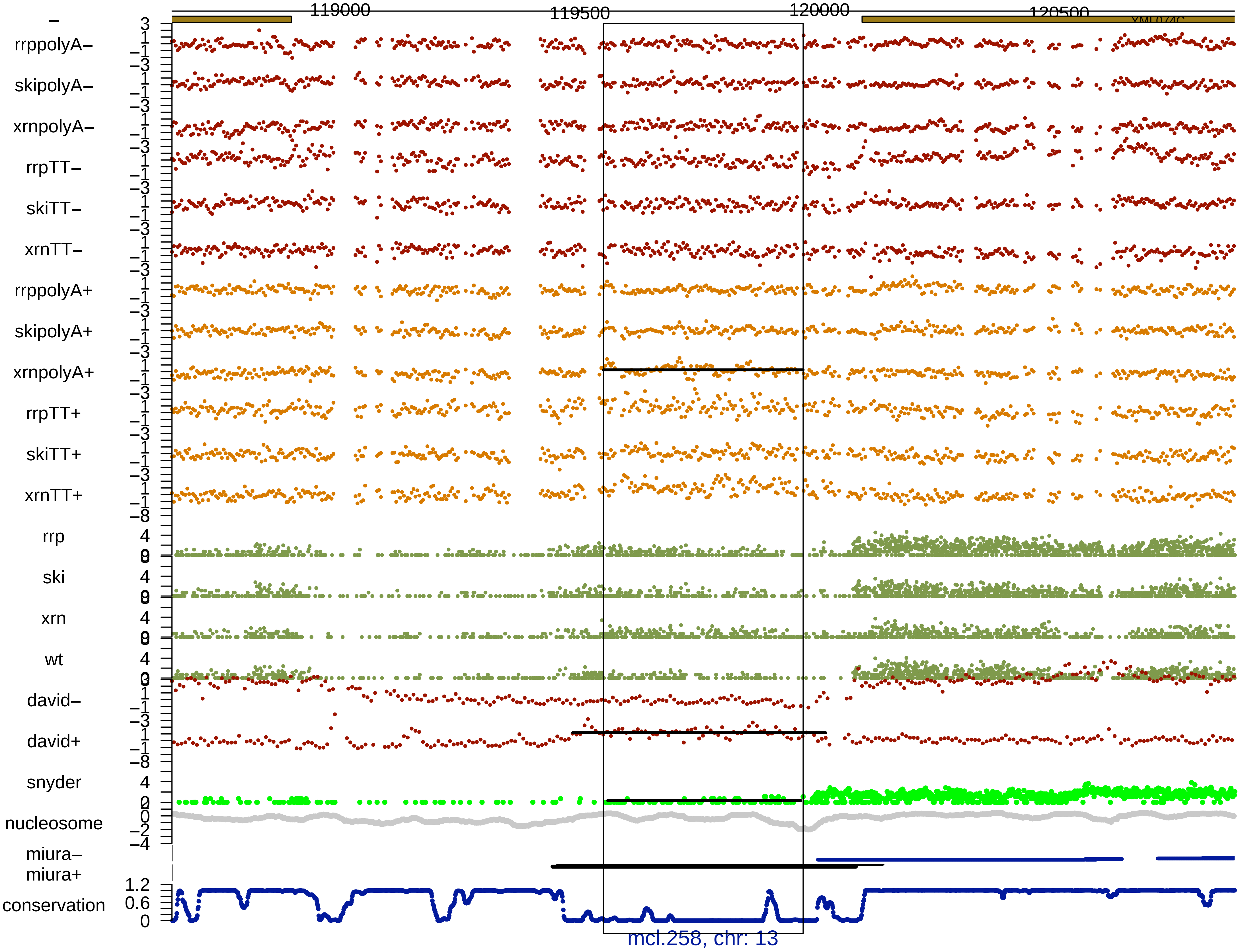

119000
119500
120000
120500
YML074C
rrppolyA−
skipolyA−
xrnpolyA−
rrpTT−
skiTT−
xrnTT−
rrppolyA+
skipolyA+
xrnpolyA+
rrpTT+
skiTT+
xrnTT+
rrp
ski
xrn
wt
david−
david+
snyder
nucleosome
miura−
miura+
conservation
mcl.258, chr: 13

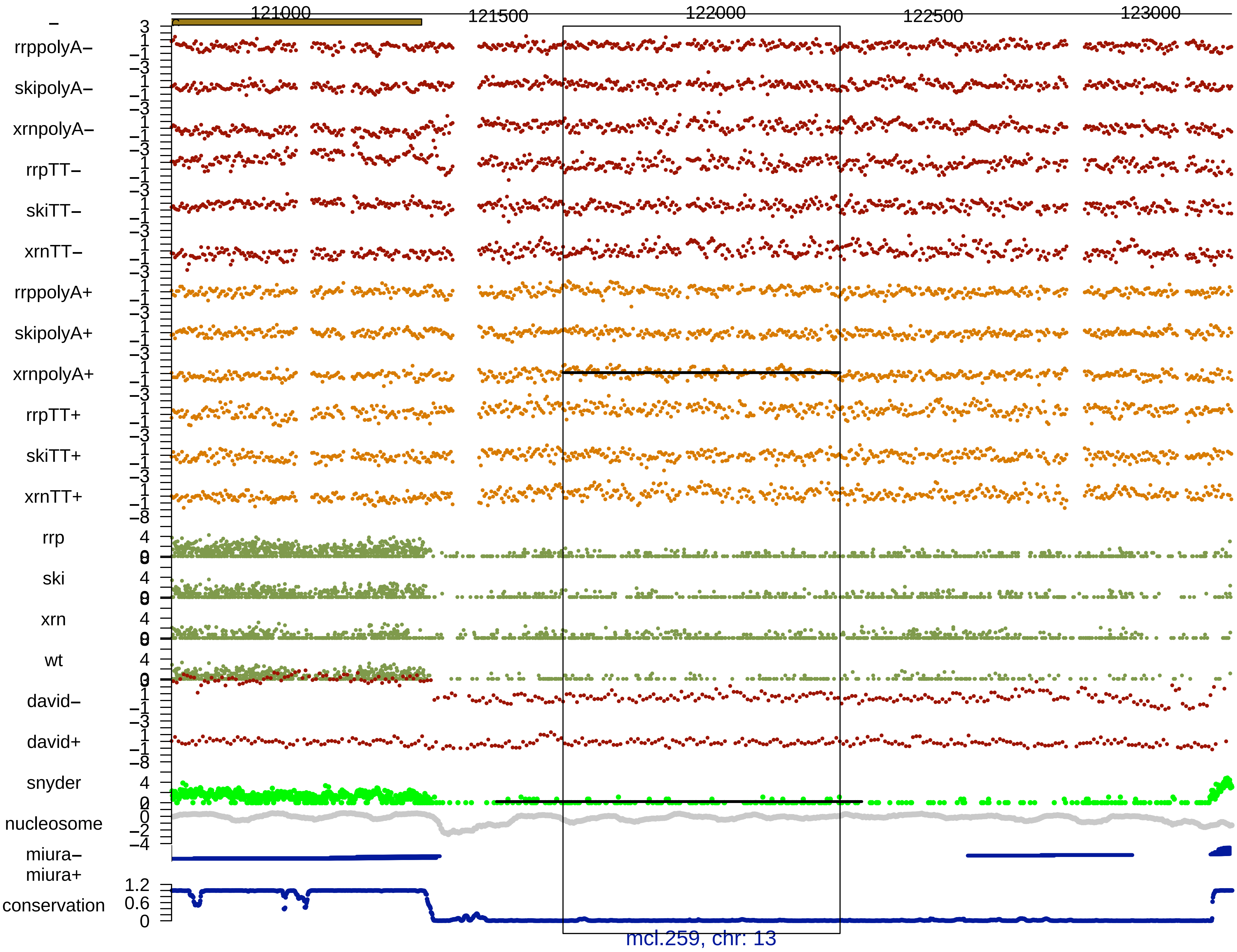

121000
121500
122000
122500
123000
rrppolyA–
skipolyA–
xrnpolyA–
rrpTT–
skiTT–
xrnTT–
rrppolyA+
skipolyA+
xrnpolyA+
rrpTT+
skiTT+
xrnTT+
rrp
ski
xrn
wt
david–
david+
snyder
nucleosome
miura–
miura+
conservation
mcl.259, chr: 13

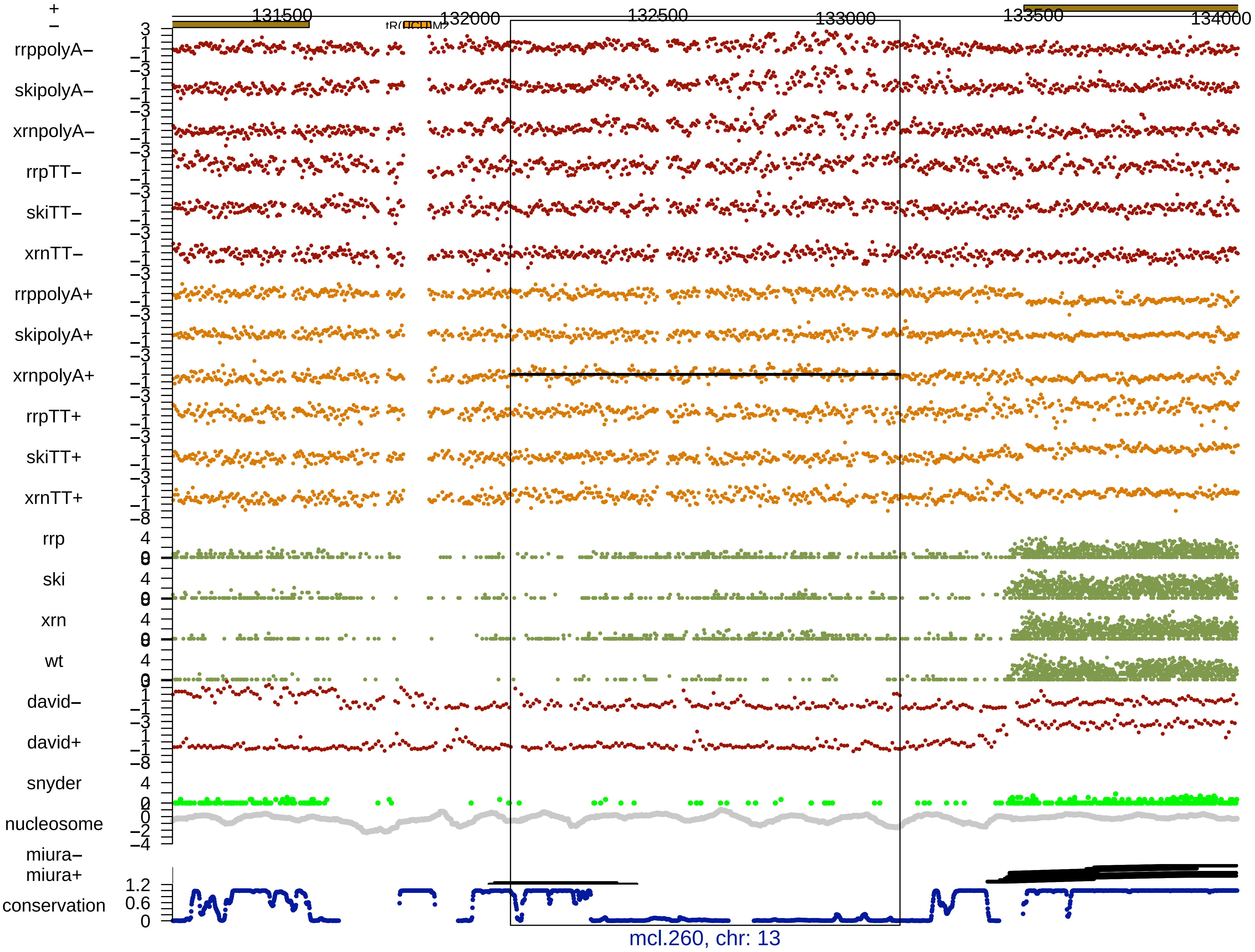

131500
132000
132500
133000
133500
134000
rrppolyA−
skipolyA−
xrnpolyA−
rrpTT−
skiTT−
xrnTT−
rrppolyA+
skipolyA+
xrnpolyA+
rrpTT+
skiTT+
xrnTT+
rrp
ski
xrn
wt
david−
david+
snyder
nucleosome
miura−
miura+
conservation
mcl.260, chr: 13

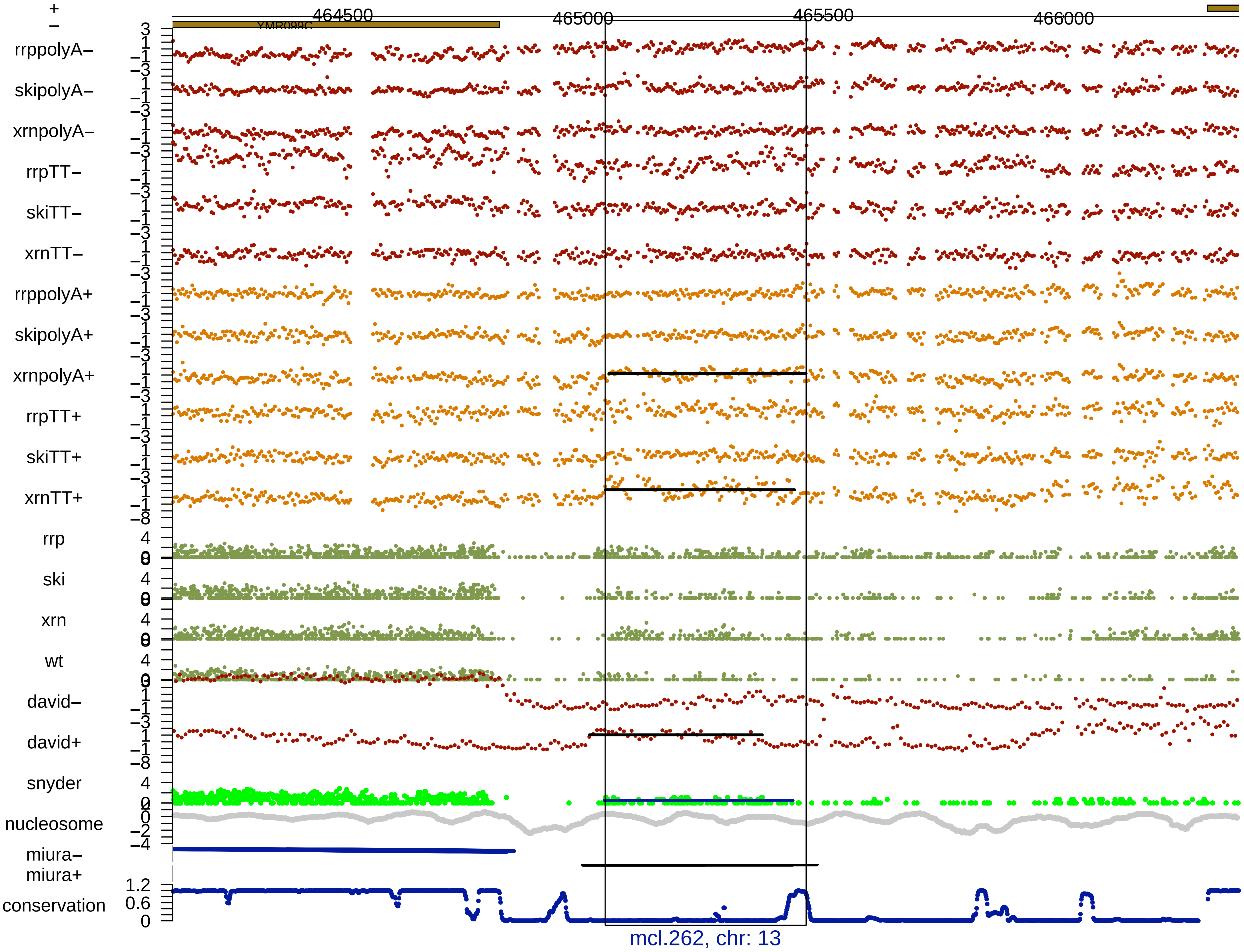

+
−
YMR099C
464500
465000
465500
466000
rrppolyA−
skipolyA−
xrnpolyA−
rrpTT−
skiTT−
xrnTT−
rrppolyA+
skipolyA+
xrnpolyA+
rrpTT+
skiTT+
xrnTT+
rrp
ski
xrn
wt
david−
david+
snyder
nucleosome
miura−
miura+
conservation
mcl.262, chr: 13

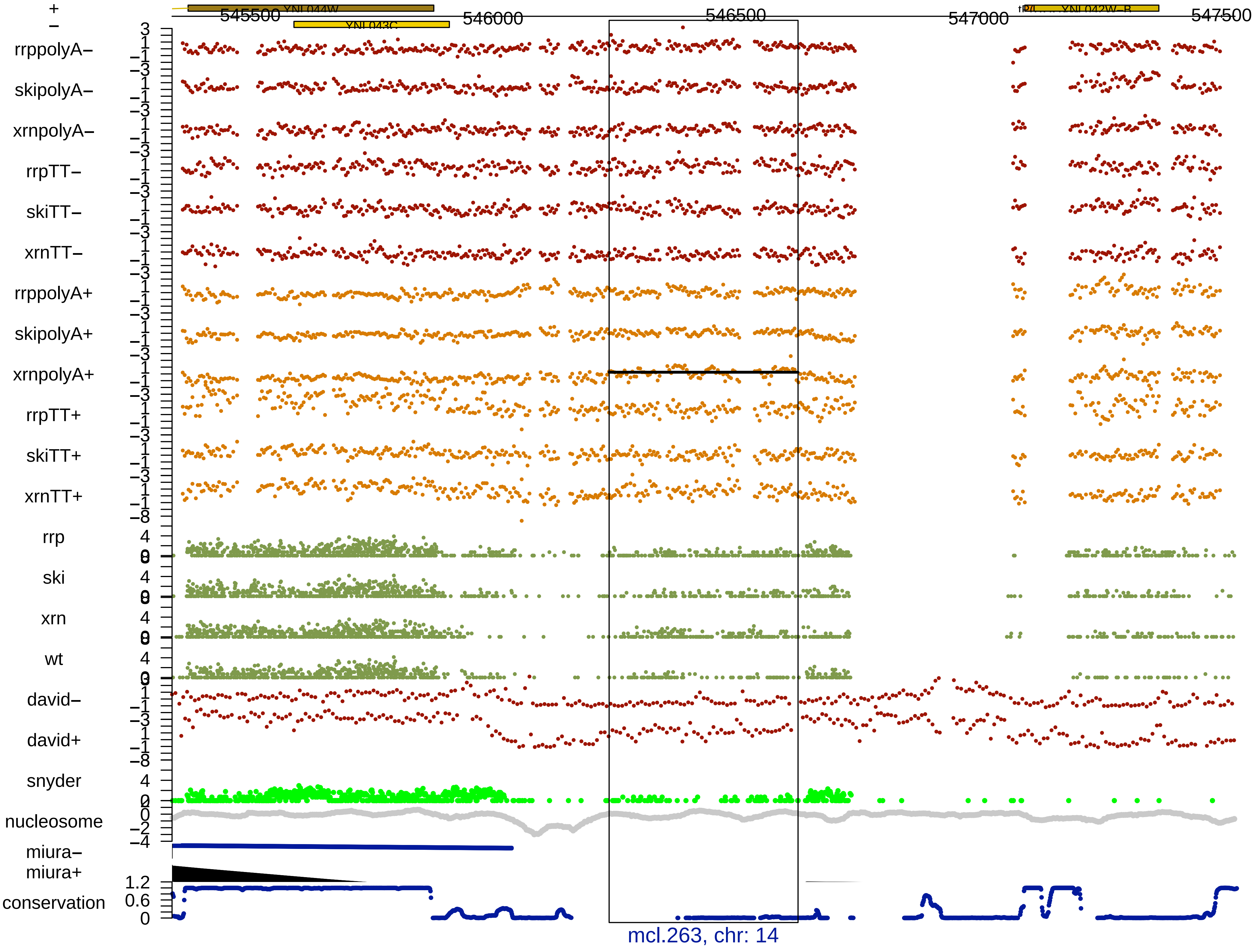

+
−
YNL044W
YNL043C
YNL042W-B
545500
546000
546500
547000
547500
rrppolyA−
skipolyA−
xrnpolyA−
rrpTT−
skiTT−
xrnTT−
rrppolyA+
skipolyA+
xrnpolyA+
rrpTT+
skiTT+
xrnTT+
rrp
ski
xrn
wt
david−
david+
snyder
nucleosome
miura−
miura+
conservation
mcl.263, chr: 14

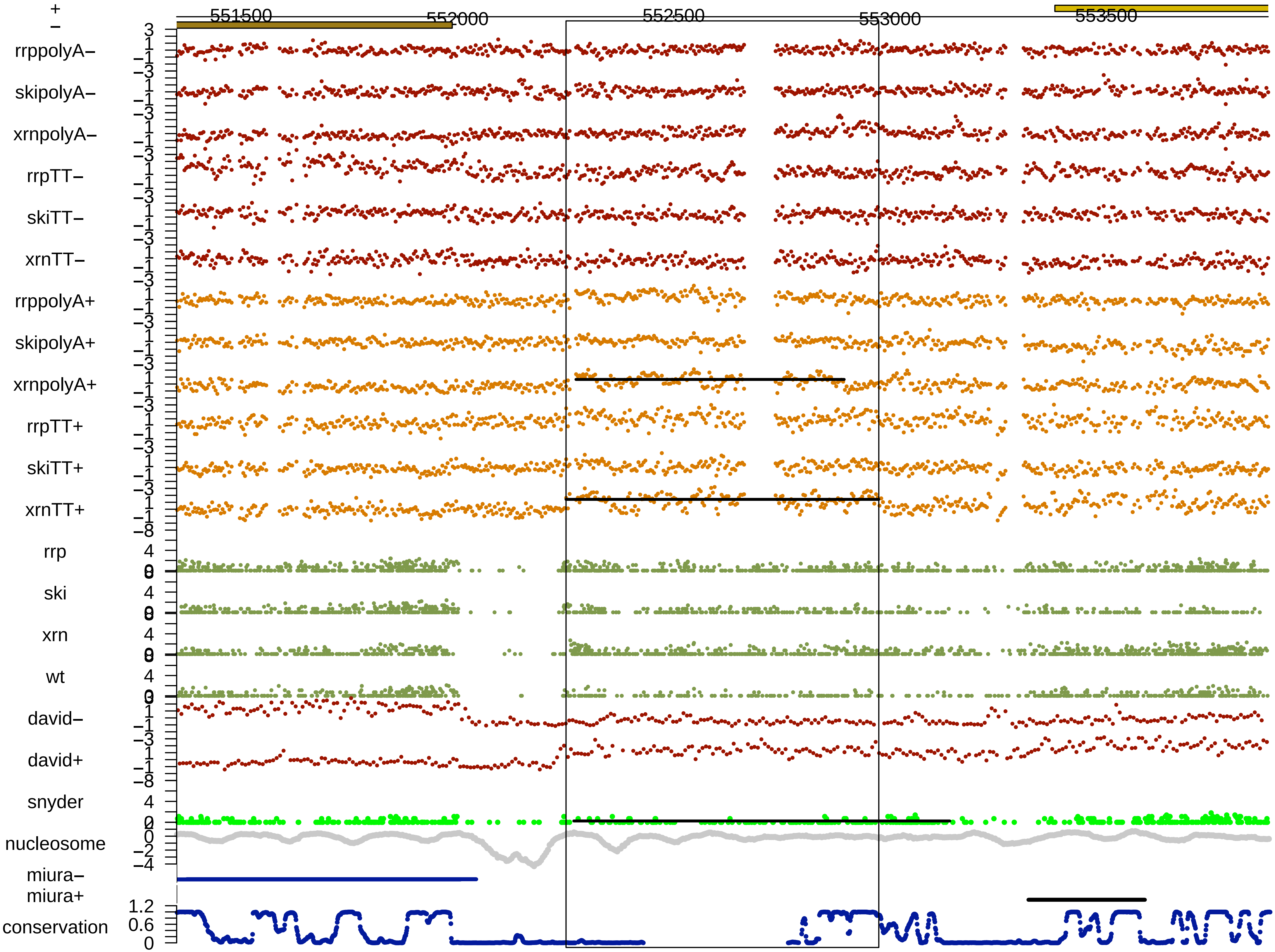

mcl.264, chr: 14

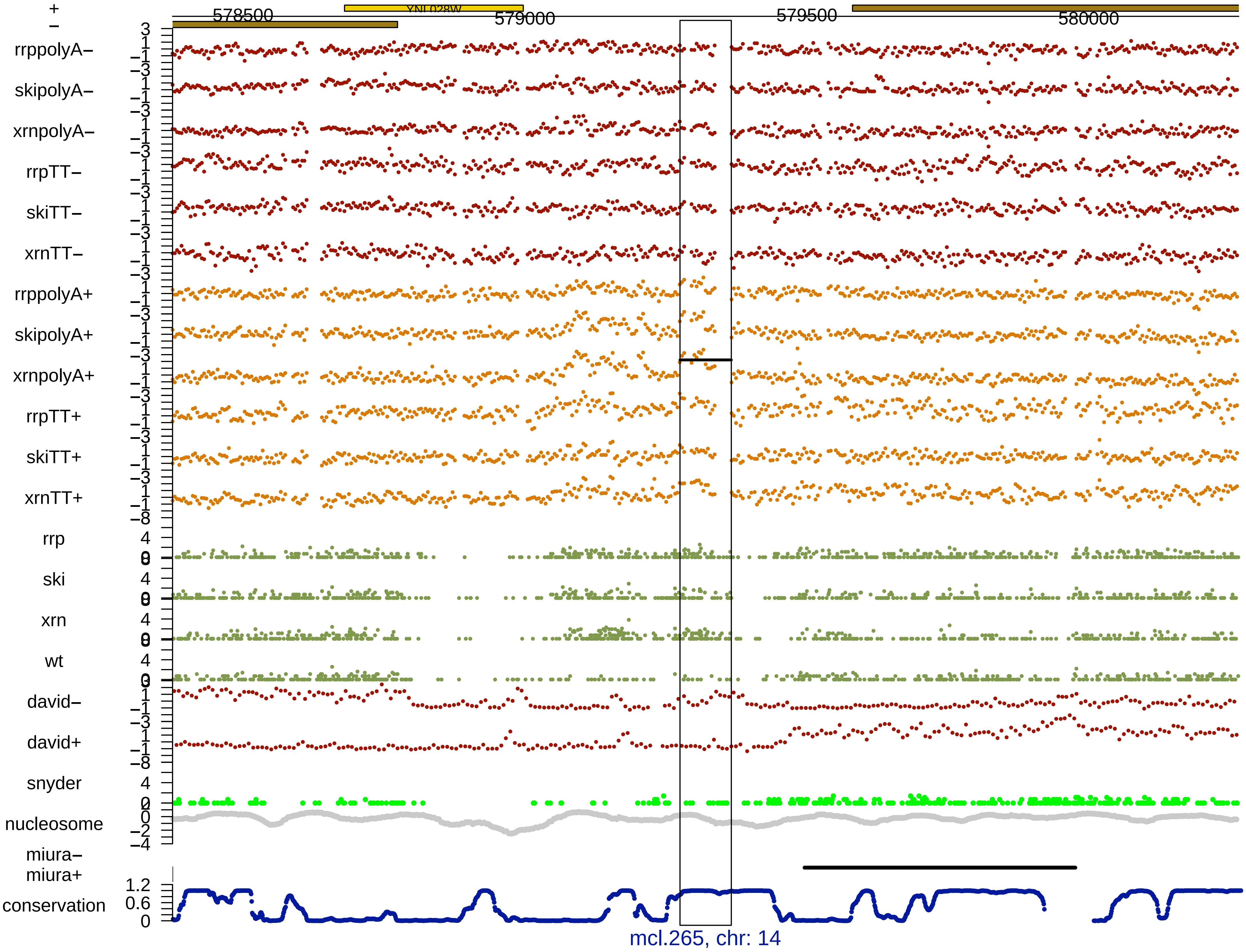

YNL028W
578500
579000
579500
580000
rrppolyA−
skipolyA−
xrnpolyA−
rrpTT−
skiTT−
xrnTT−
rrppolyA+
skipolyA+
xrnpolyA+
rrpTT+
skiTT+
xrnTT+
rrp
ski
xrn
wt
david−
david+
snyder
nucleosome
miura−
miura+
conservation
mcl.265, chr: 14

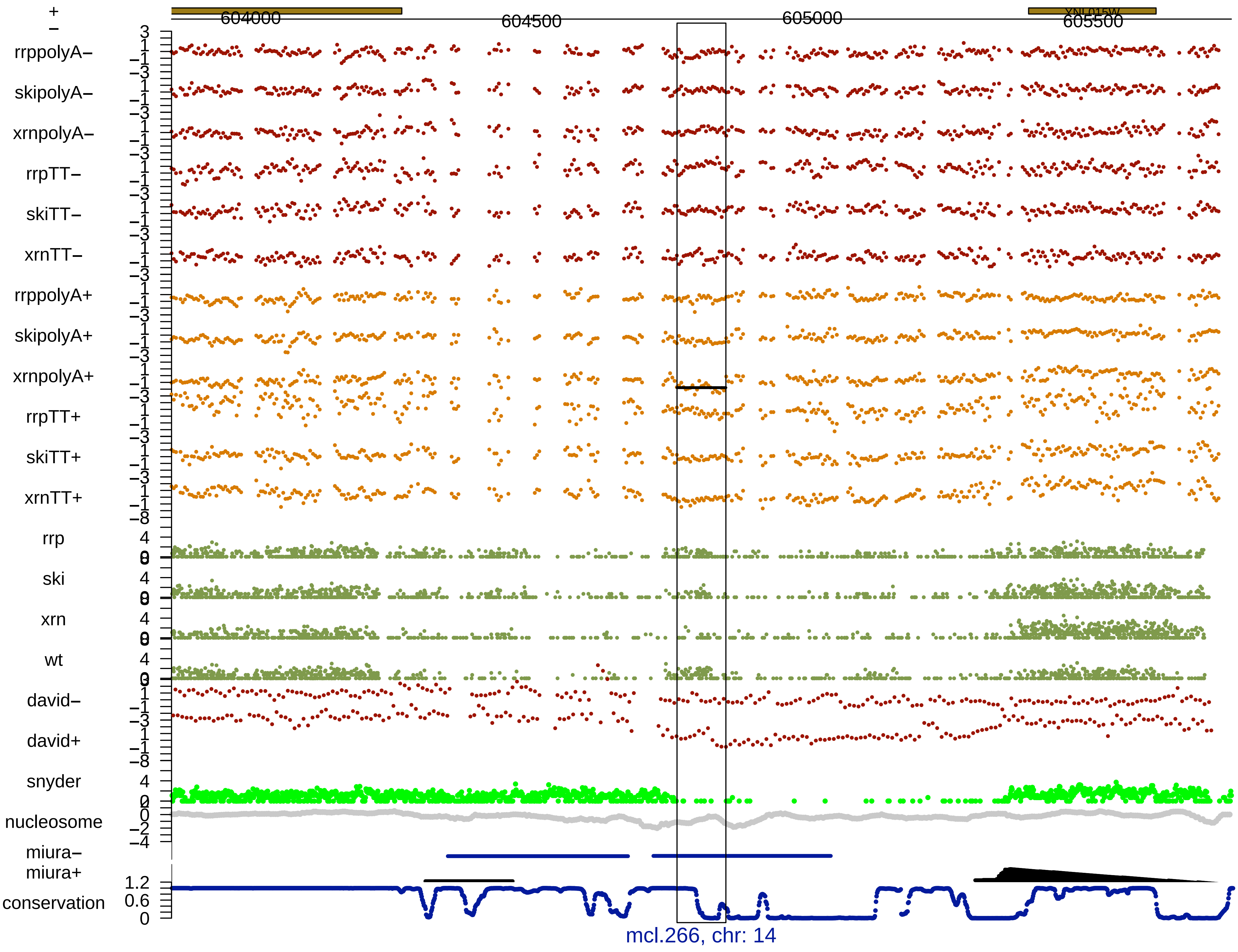
+
–
604000
604500
605000
605500
YNL015W
rrppolyA–
skipolyA–
xrnpolyA–
rrpTT–
skiTT–
xrnTT–
rrppolyA+
skipolyA+
xrnpolyA+
rrpTT+
skiTT+
xrnTT+
rrp
ski
xrn
wt
david–
david+
snyder
nucleosome
miura–
miura+
conservation
mcl.266, chr: 14

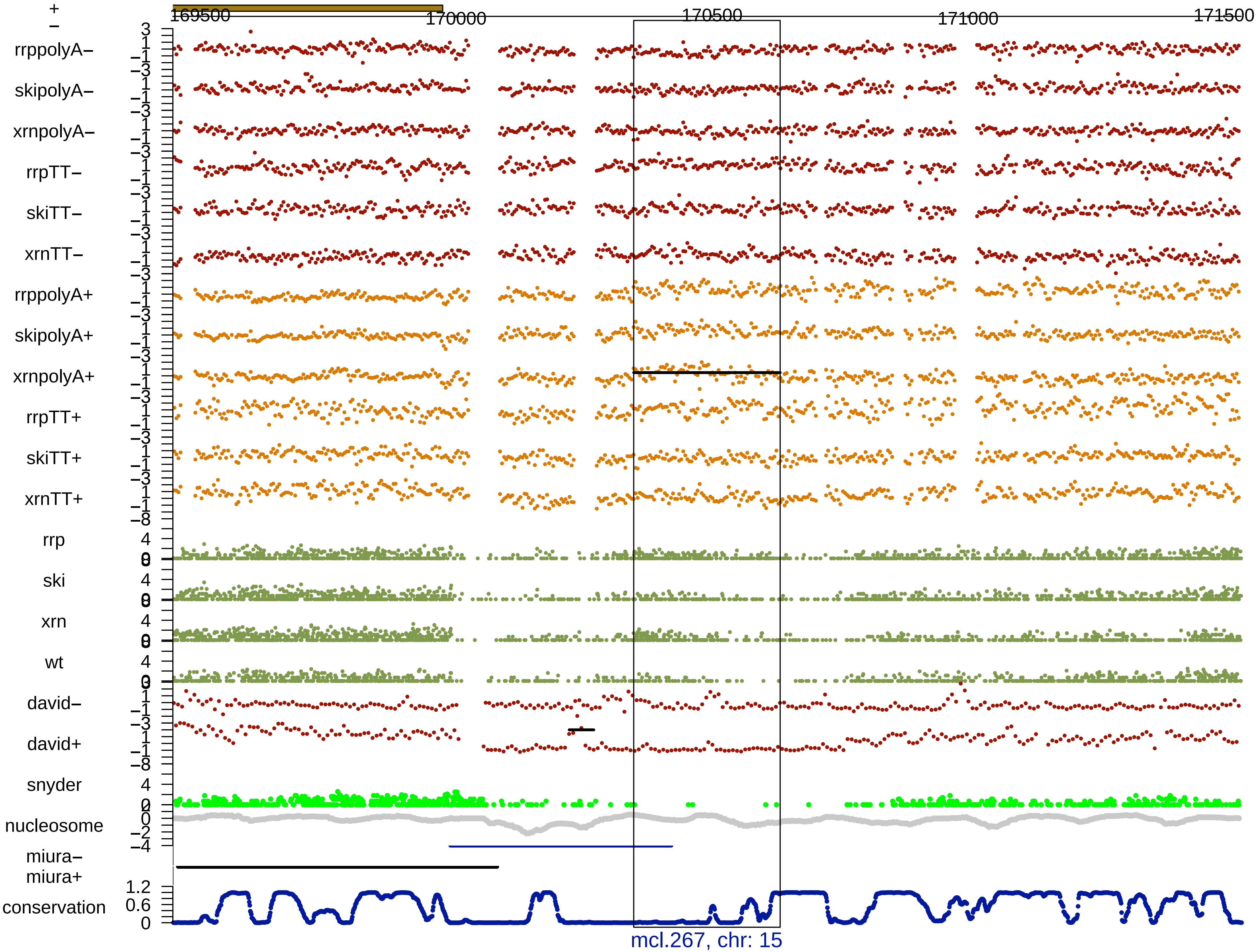

169500
170000
170500
171000
171500
rrppolyA−
skipolyA−
xrnpolyA−
rrpTT−
skiTT−
xrnTT−
rrppolyA+
skipolyA+
xrnpolyA+
rrpTT+
skiTT+
xrnTT+
rrp
ski
xrn
wt
david−
david+
snyder
nucleosome
miura−
miura+
conservation
mcl.267, chr: 15

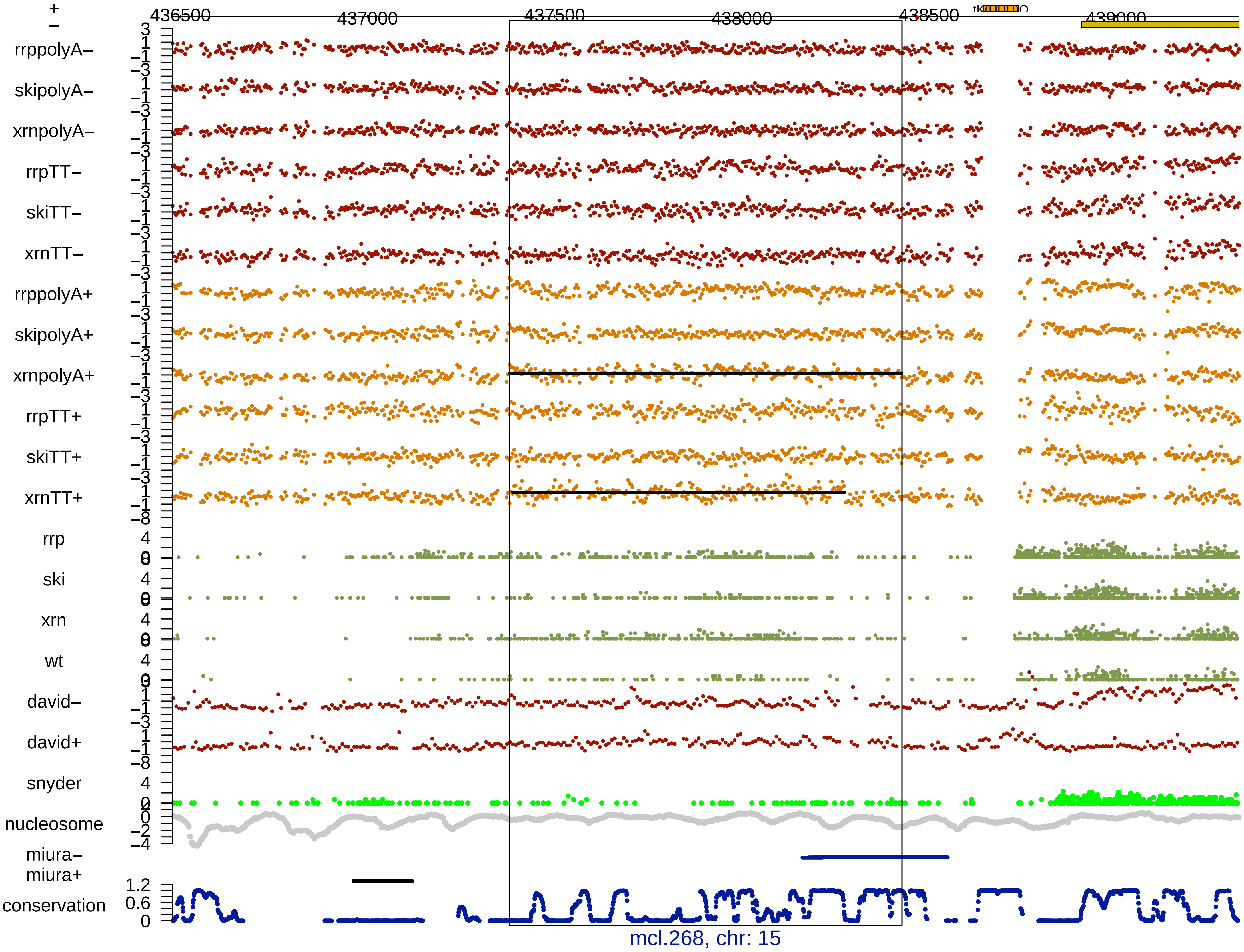

436500
437000
437500
438000
438500
439000
rrppolyA−
skipolyA−
xrnpolyA−
rrpTT−
skiTT−
xrnTT−
rrppolyA+
skipolyA+
xrnpolyA+
rrpTT+
skiTT+
xrnTT+
rrp
ski
xrn
wt
david−
david+
snyder
nucleosome
miura−
miura+
conservation
mcl.268, chr: 15

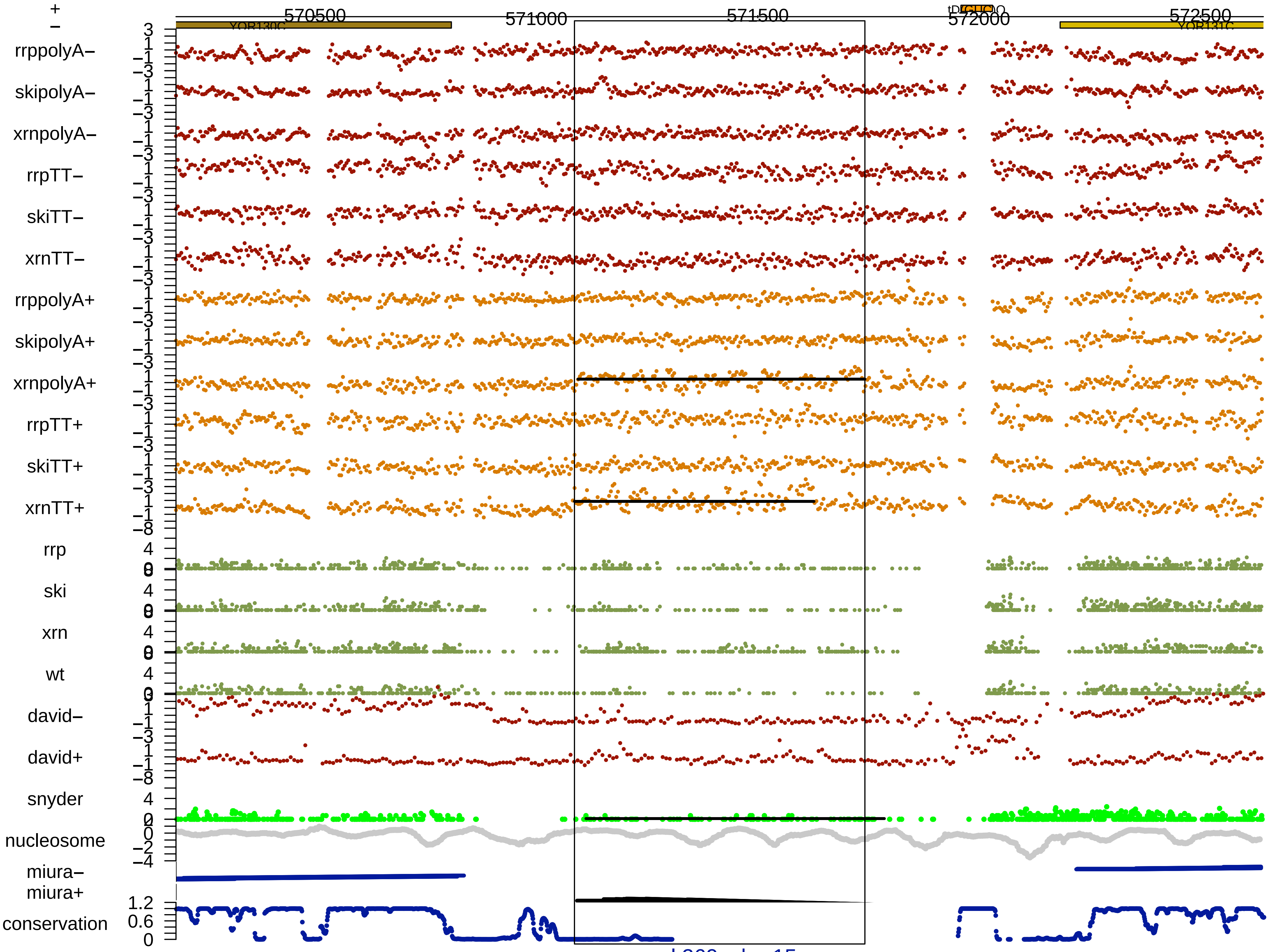

+
−
570500
571000
571500
572000
572500
YOR130C
YOR131C
rrppolyA−
skipolyA−
xrnpolyA−
rrpTT−
skiTT−
xrnTT−
rrppolyA+
skipolyA+
xrnpolyA+
rrpTT+
skiTT+
xrnTT+
rrp
ski
xrn
wt
david−
david+
snyder
nucleosome
miura−
miura+
conservation
mcl.269, chr: 15

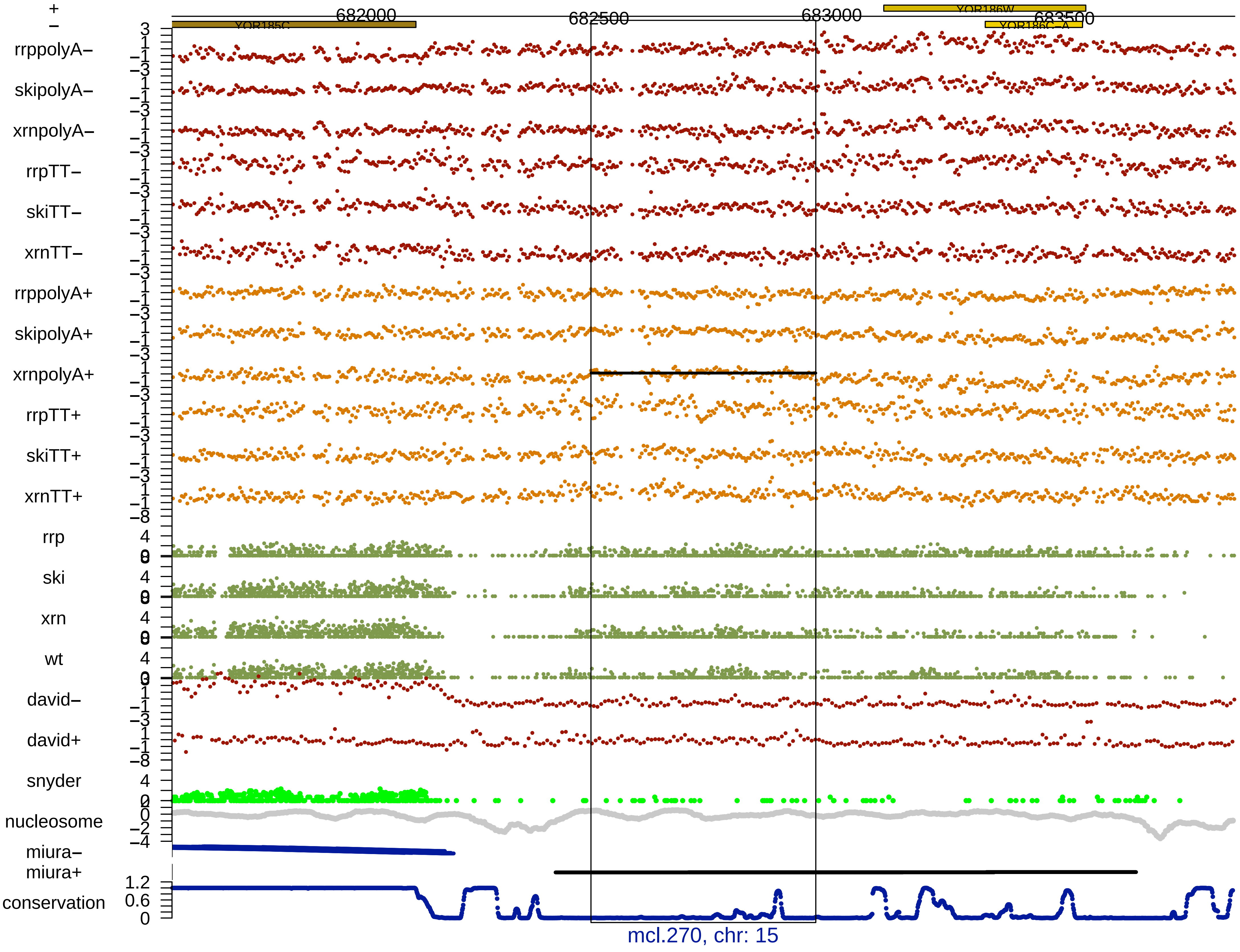

+
–
682000
682500
683000
683500
YOR185C
YOR186W
YOR186C-A
rrppolyA–
skipolyA–
xrnpolyA–
rrpTT–
skiTT–
xrnTT–
rrppolyA+
skipolyA+
xrnpolyA+
rrpTT+
skiTT+
xrnTT+
rrp
ski
xrn
wt
david–
david+
snyder
nucleosome
miura–
miura+
conservation
mcl.270, chr: 15

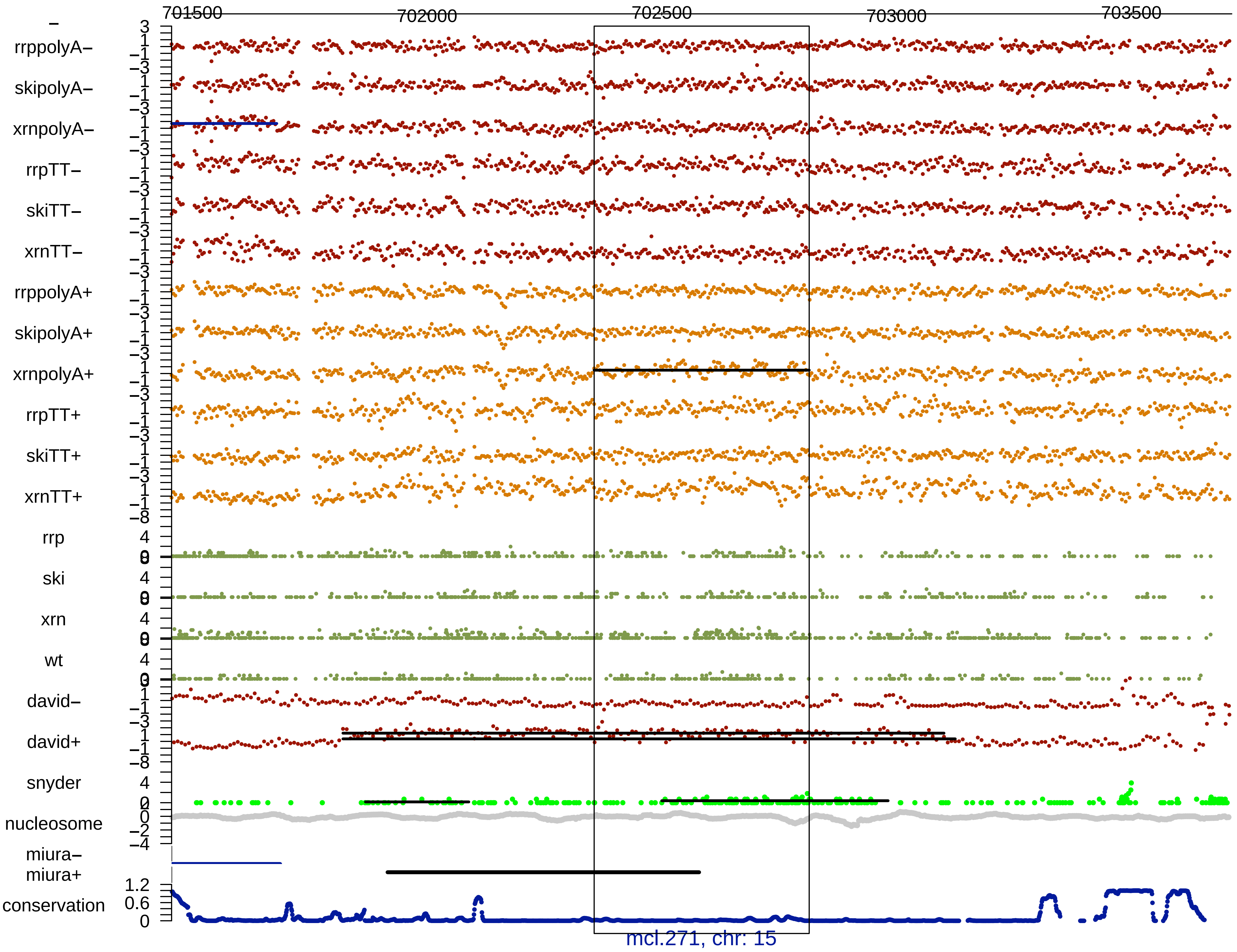

701500
702000
702500
703000
703500
rrppolyA−
skipolyA−
xrnpolyA−
rrpTT−
skiTT−
xrnTT−
rrppolyA+
skipolyA+
xrnpolyA+
rrpTT+
skiTT+
xrnTT+
rrp
ski
xrn
wt
david−
david+
snyder
nucleosome
miura−
miura+
conservation
mcl.271, chr: 15

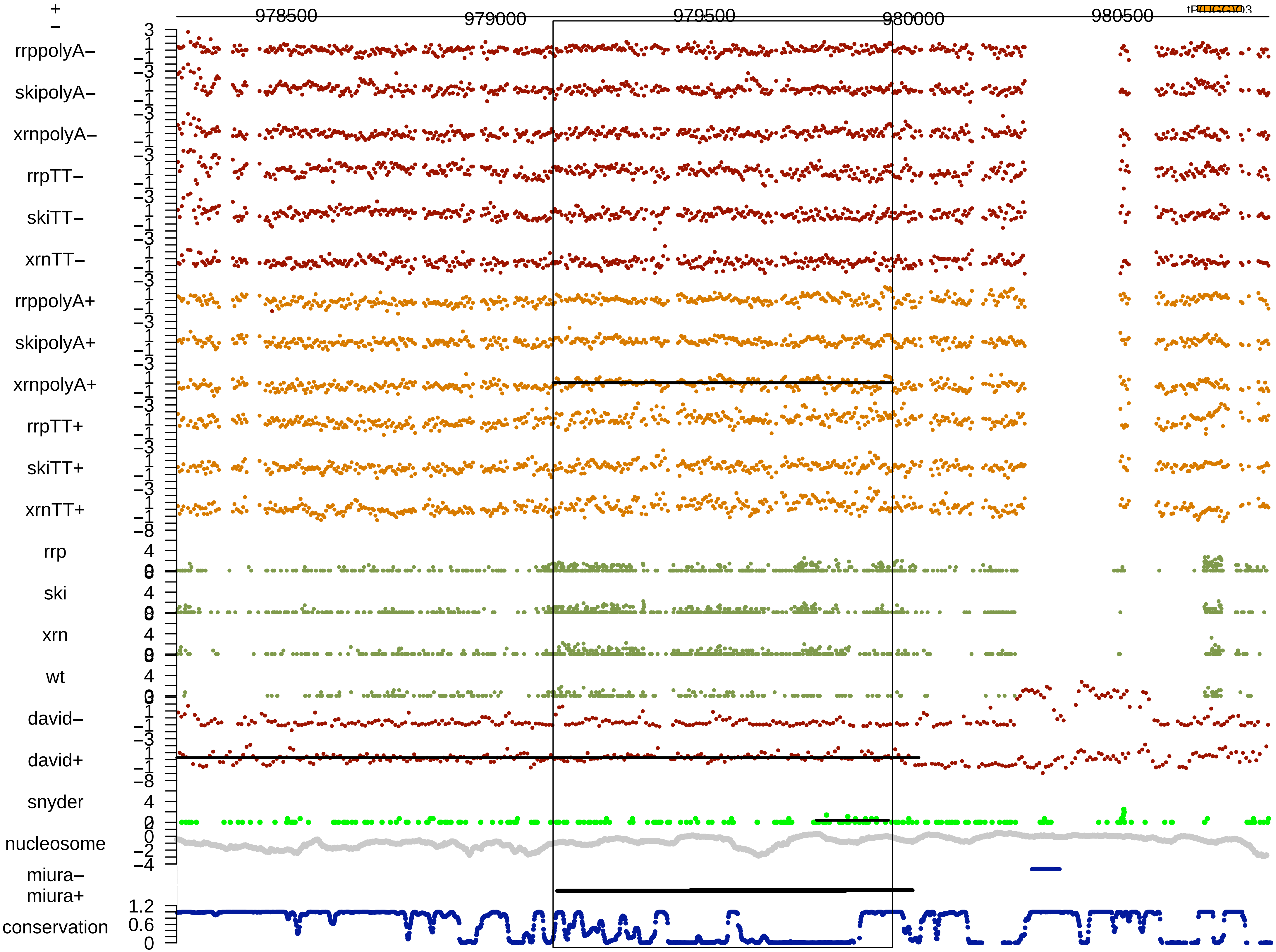

mcl.272, chr: 15

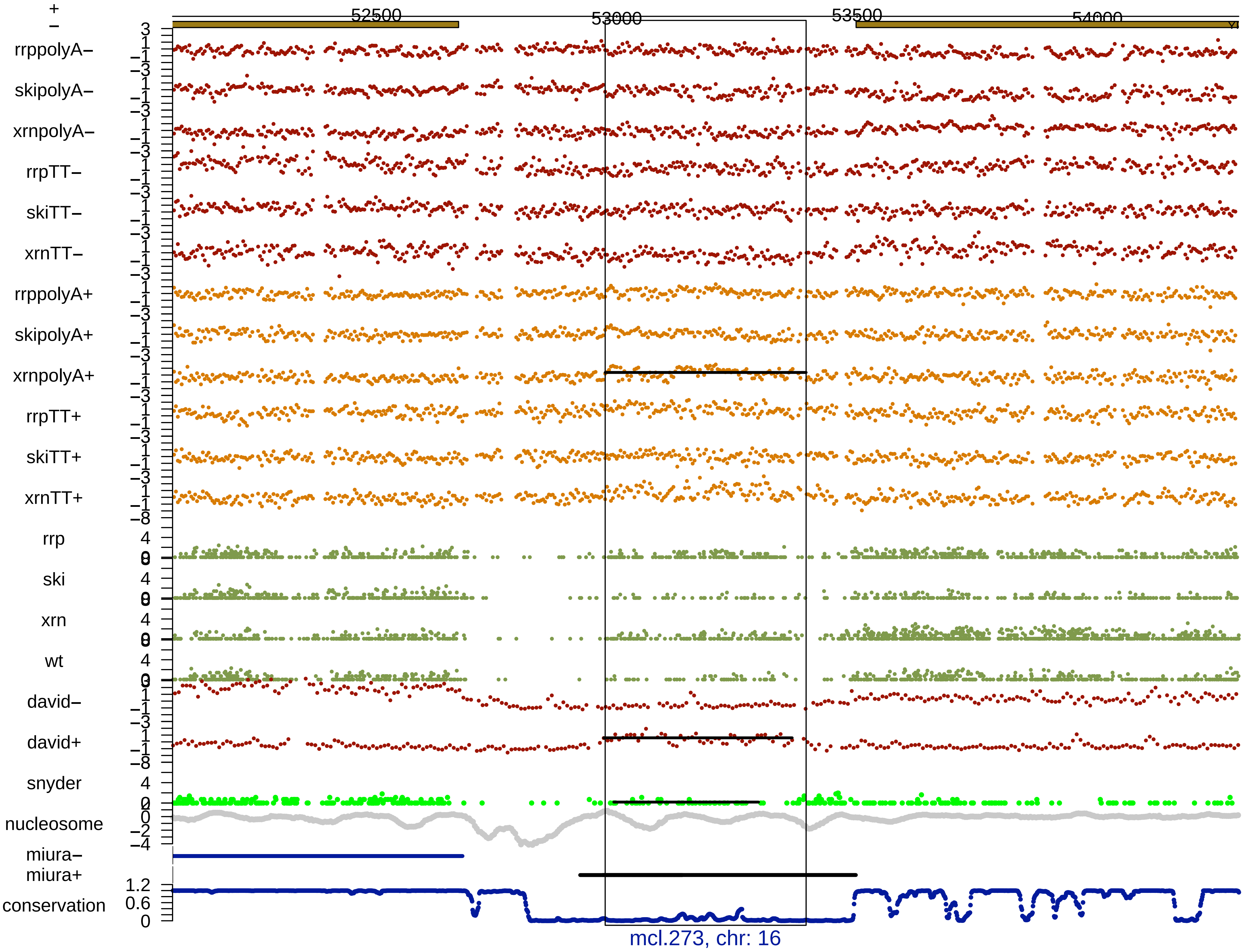

52500
53000
53500
54000
rrppolyA−
skipolyA−
xrnpolyA−
rrpTT−
skiTT−
xrnTT−
rrppolyA+
skipolyA+
xrnpolyA+
rrpTT+
skiTT+
xrnTT+
rrp
ski
xrn
wt
david−
david+
snyder
nucleosome
miura−
miura+
conservation
mcl.273, chr: 16

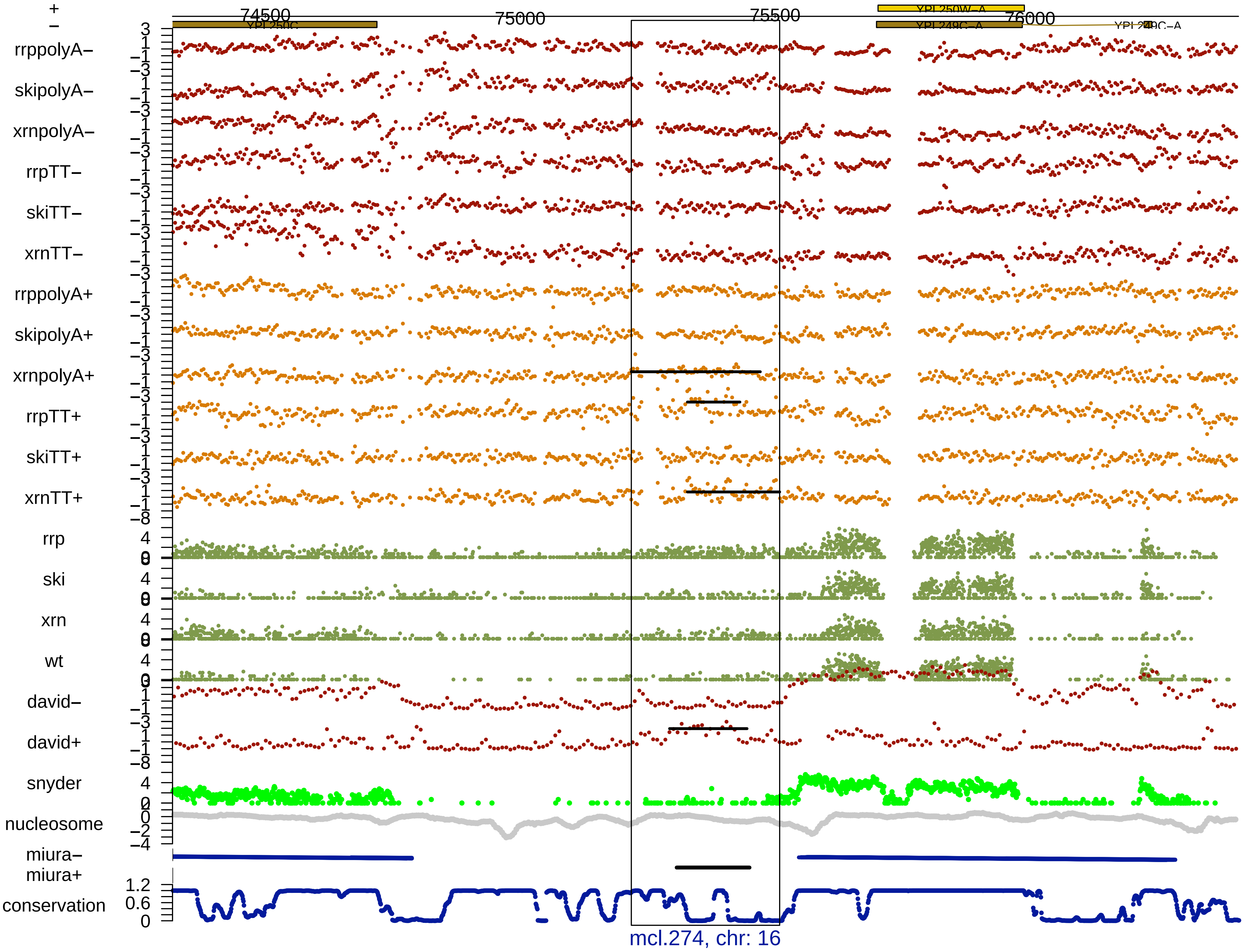
74500
75000
75500
76000
rrppolyA−
skipolyA−
xrnpolyA−
rrpTT−
skiTT−
xrnTT−
rrppolyA+
skipolyA+
xrnpolyA+
rrpTT+
skiTT+
xrnTT+
rrp
ski
xrn
wt
david−
david+
snyder
nucleosome
miura−
miura+
conservation
mcl.274, chr: 16

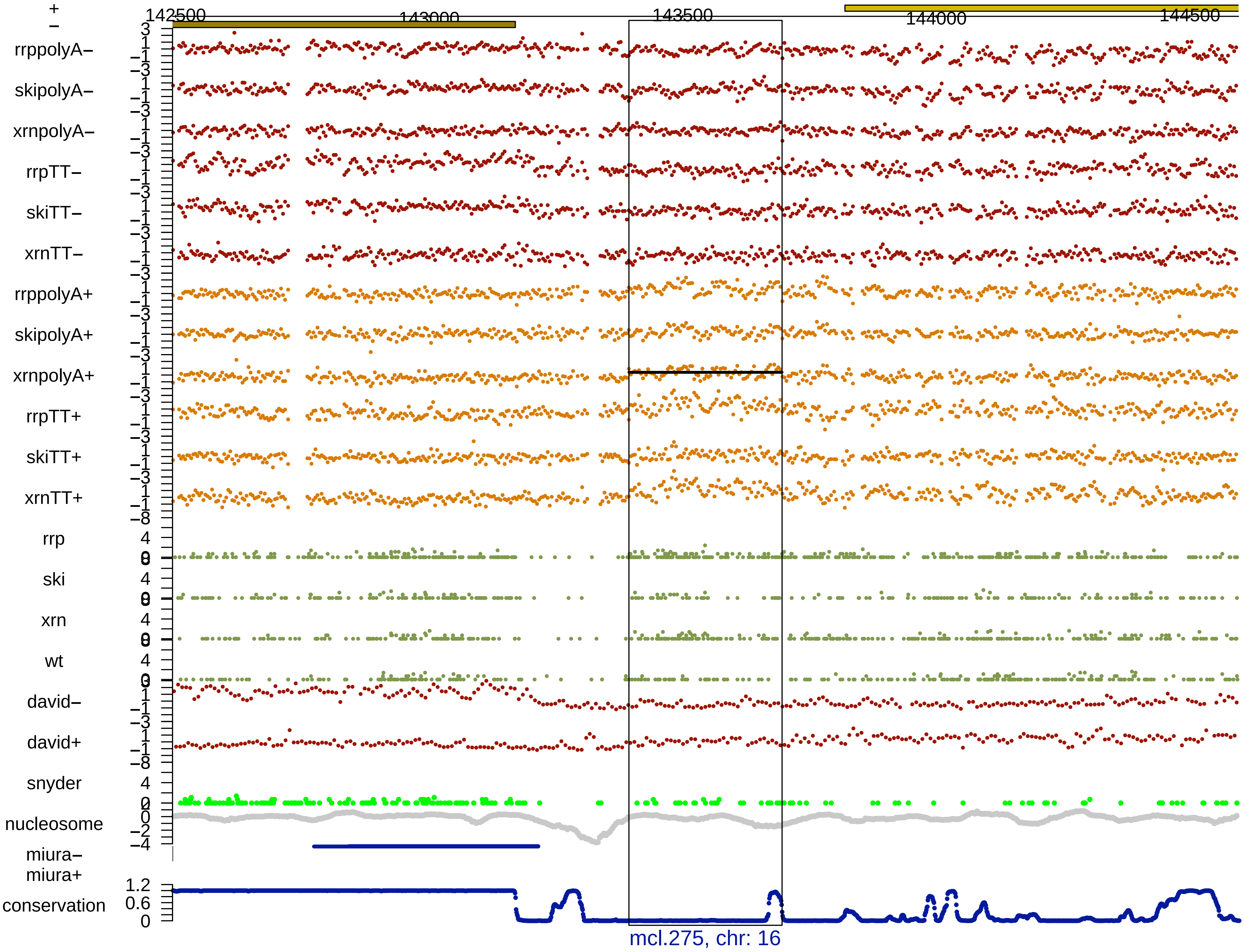

142500
143000
143500
144000
144500
rrppolyA−
skipolyA−
xrnpolyA−
rrpTT−
skiTT−
xrnTT−
rrppolyA+
skipolyA+
xrnpolyA+
rrpTT+
skiTT+
xrnTT+
rrp
ski
xrn
wt
david−
david+
snyder
nucleosome
miura−
miura+
conservation
mcl.275, chr: 16

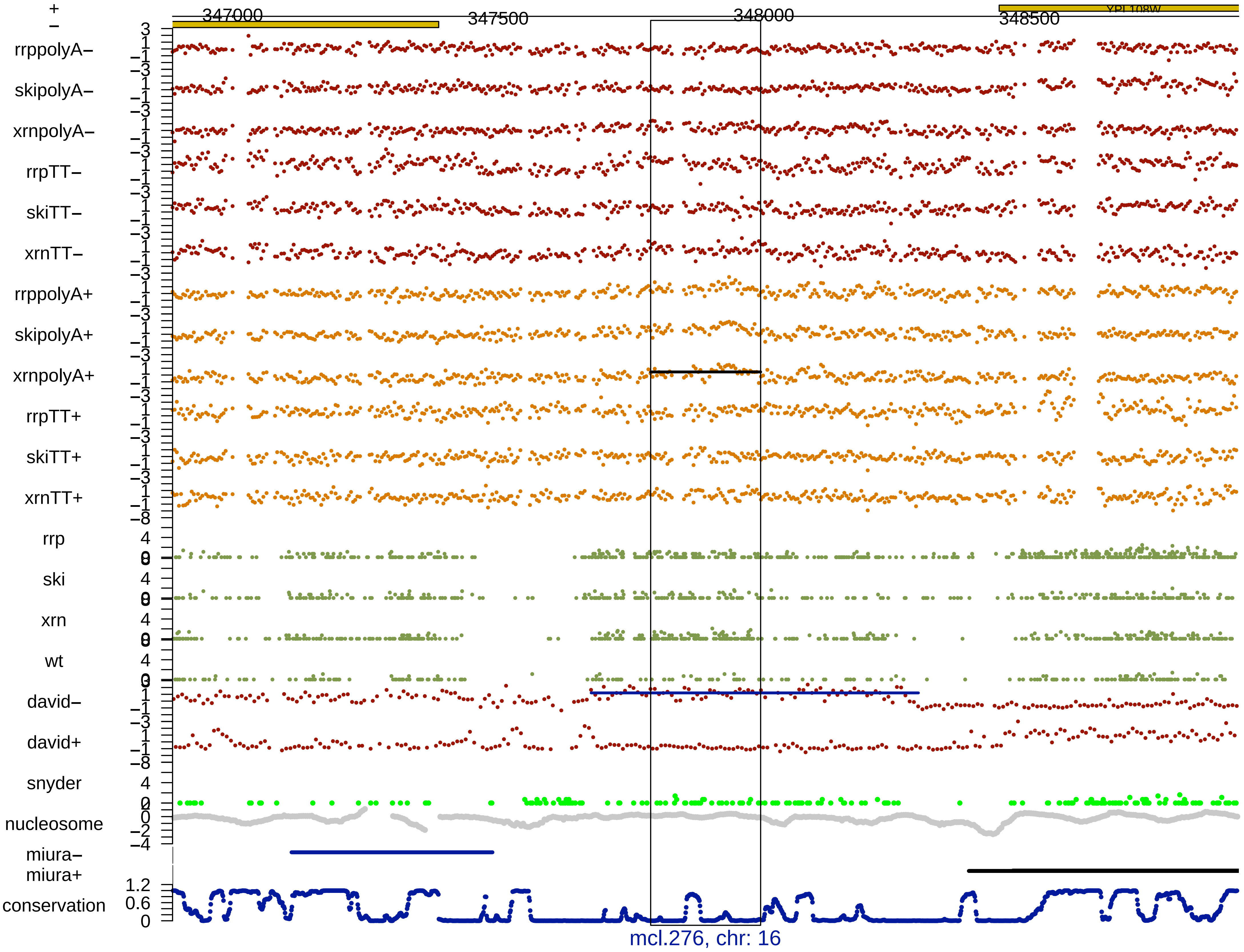

+
−
347000
347500
348000
348500
YPL108W
rrppolyA−
skipolyA−
xrnpolyA−
rrpTT−
skiTT−
xrnTT−
rrppolyA+
skipolyA+
xrnpolyA+
rrpTT+
skiTT+
xrnTT+
rrp
ski
xrn
wt
david−
david+
snyder
nucleosome
miura−
miura+
conservation
mcl.276, chr: 16

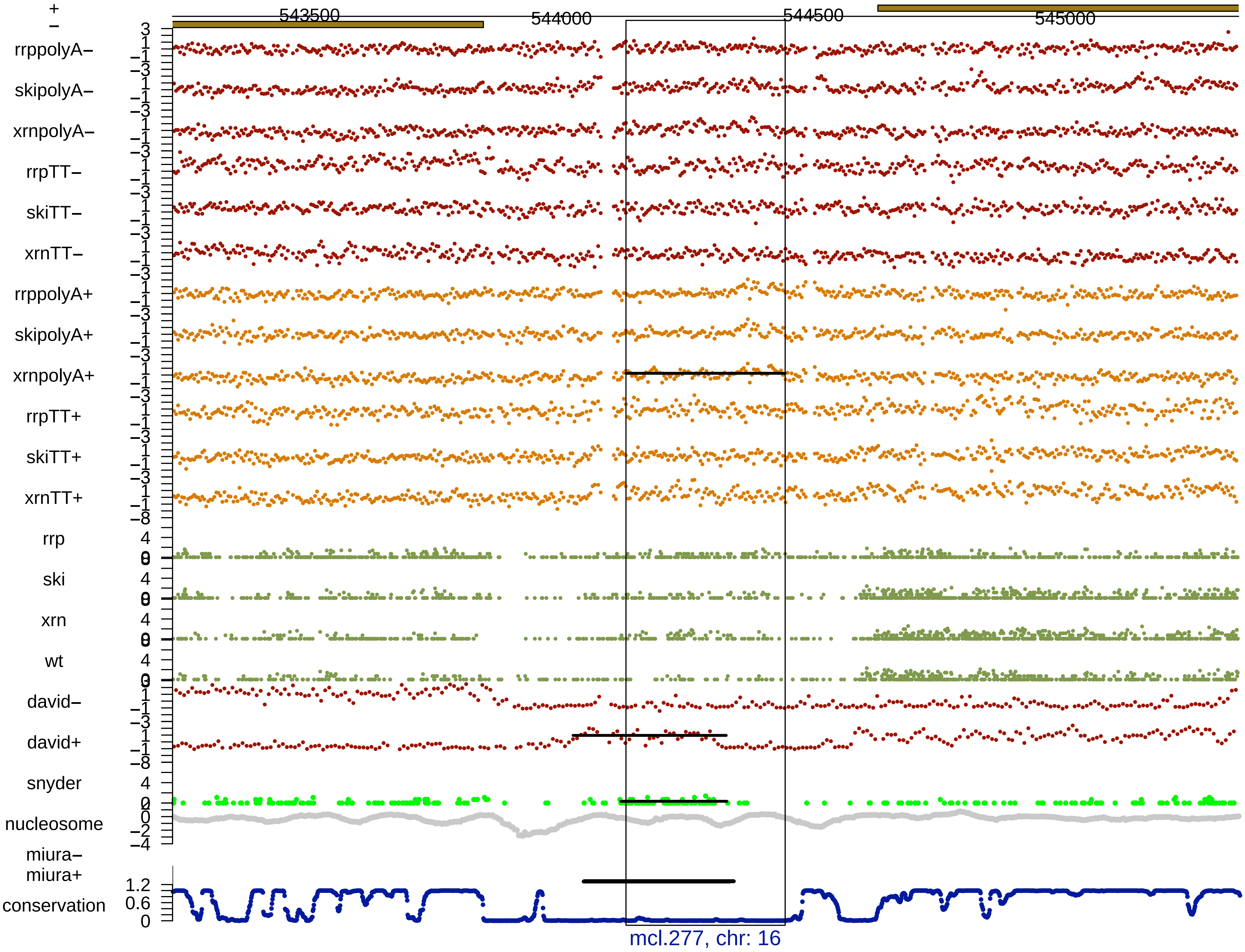

+
−
543500
544000
544500
545000
rrppolyA−
skipolyA−
xrnpolyA−
rrpTT−
skiTT−
xrnTT−
rrppolyA+
skipolyA+
xrnpolyA+
rrpTT+
skiTT+
xrnTT+
rrp
ski
xrn
wt
david−
david+
snyder
nucleosome
miura−
miura+
conservation
mcl.277, chr: 16

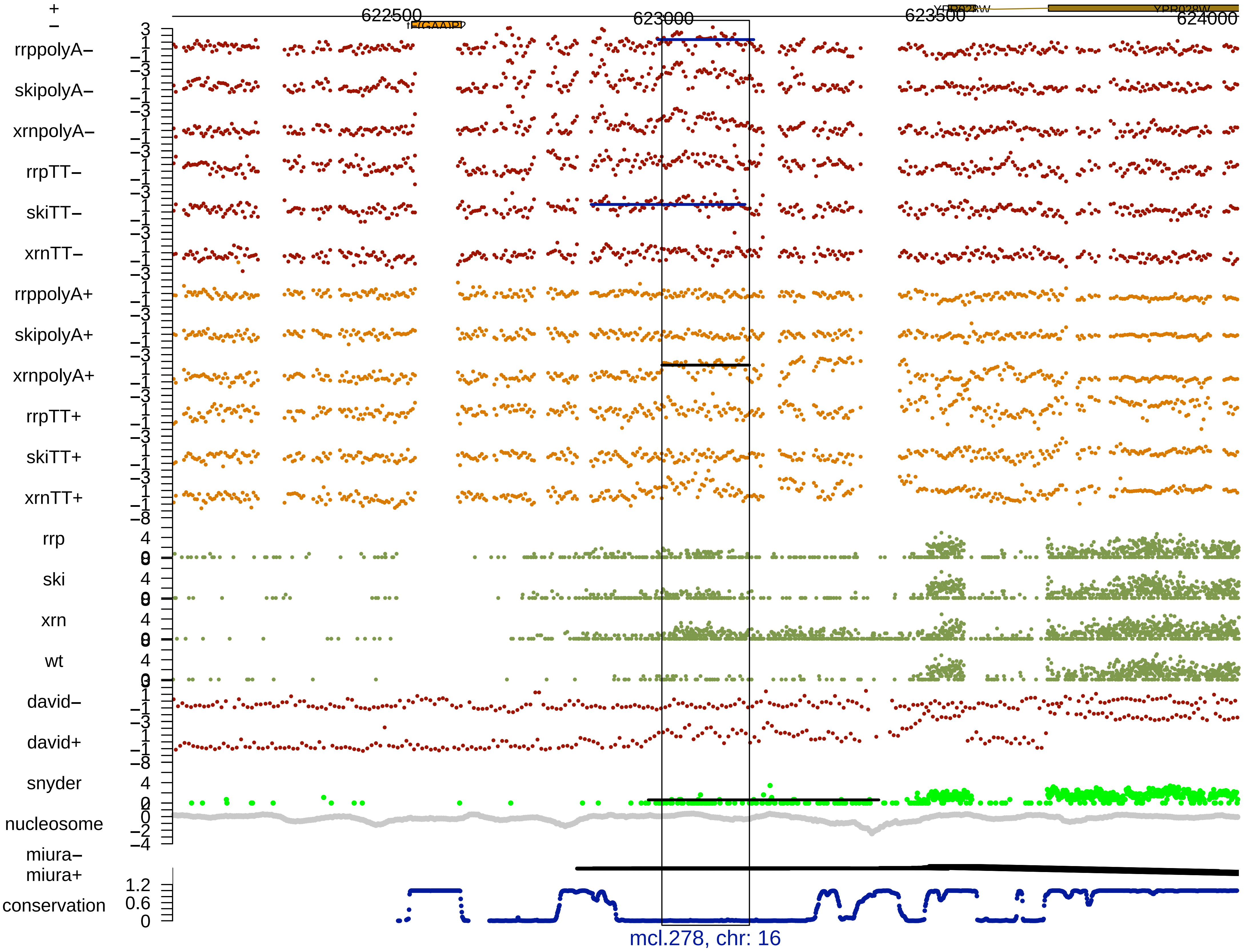

622500
623000
623500
624000
rrppolyA−
skipolyA−
xrnpolyA−
rrpTT−
skiTT−
xrnTT−
rrppolyA+
skipolyA+
xrnpolyA+
rrpTT+
skiTT+
xrnTT+
rrp
ski
xrn
wt
david−
david+
snyder
nucleosome
miura−
miura+
conservation
mcl.278, chr: 16

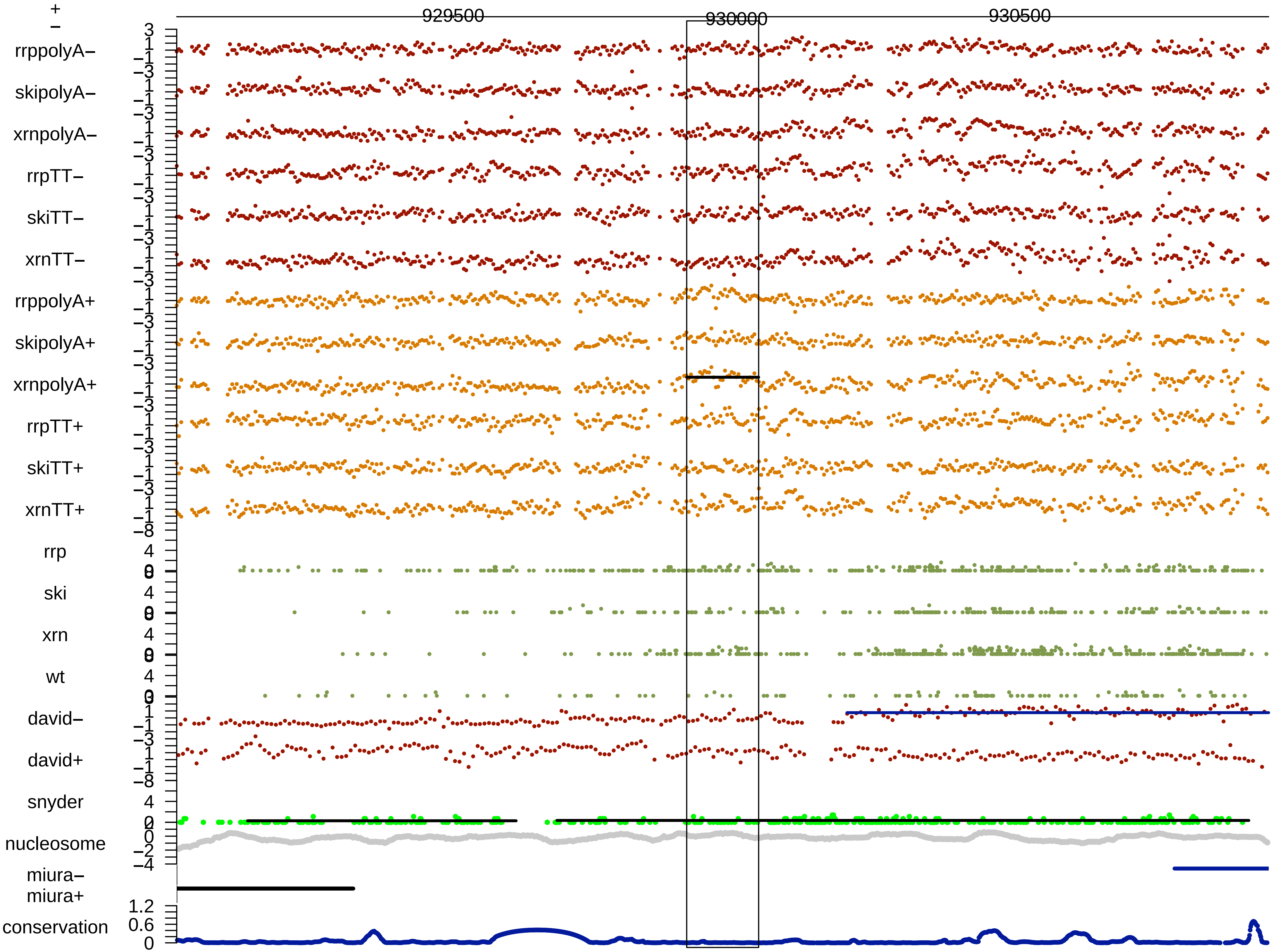

mcl.279, chr: 16

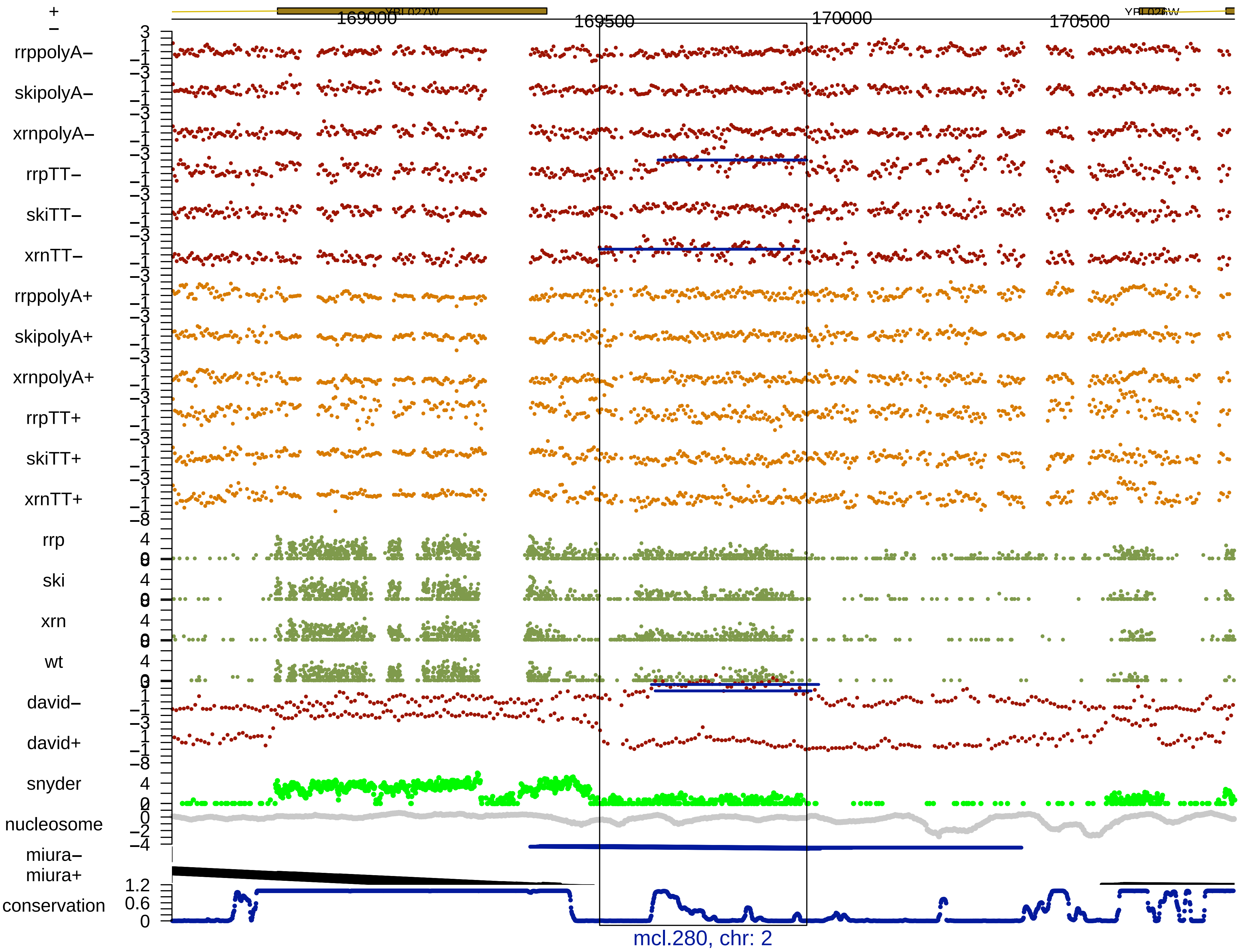

+
−
YBL027W
YBL026W
169000
169500
170000
170500
rrppolyA−
skipolyA−
xrnpolyA−
rrpTT−
skiTT−
xrnTT−
rrppolyA+
skipolyA+
xrnpolyA+
rrpTT+
skiTT+
xrnTT+
rrp
ski
xrn
wt
david−
david+
snyder
nucleosome
miura−
miura+
conservation
mcl.280, chr: 2

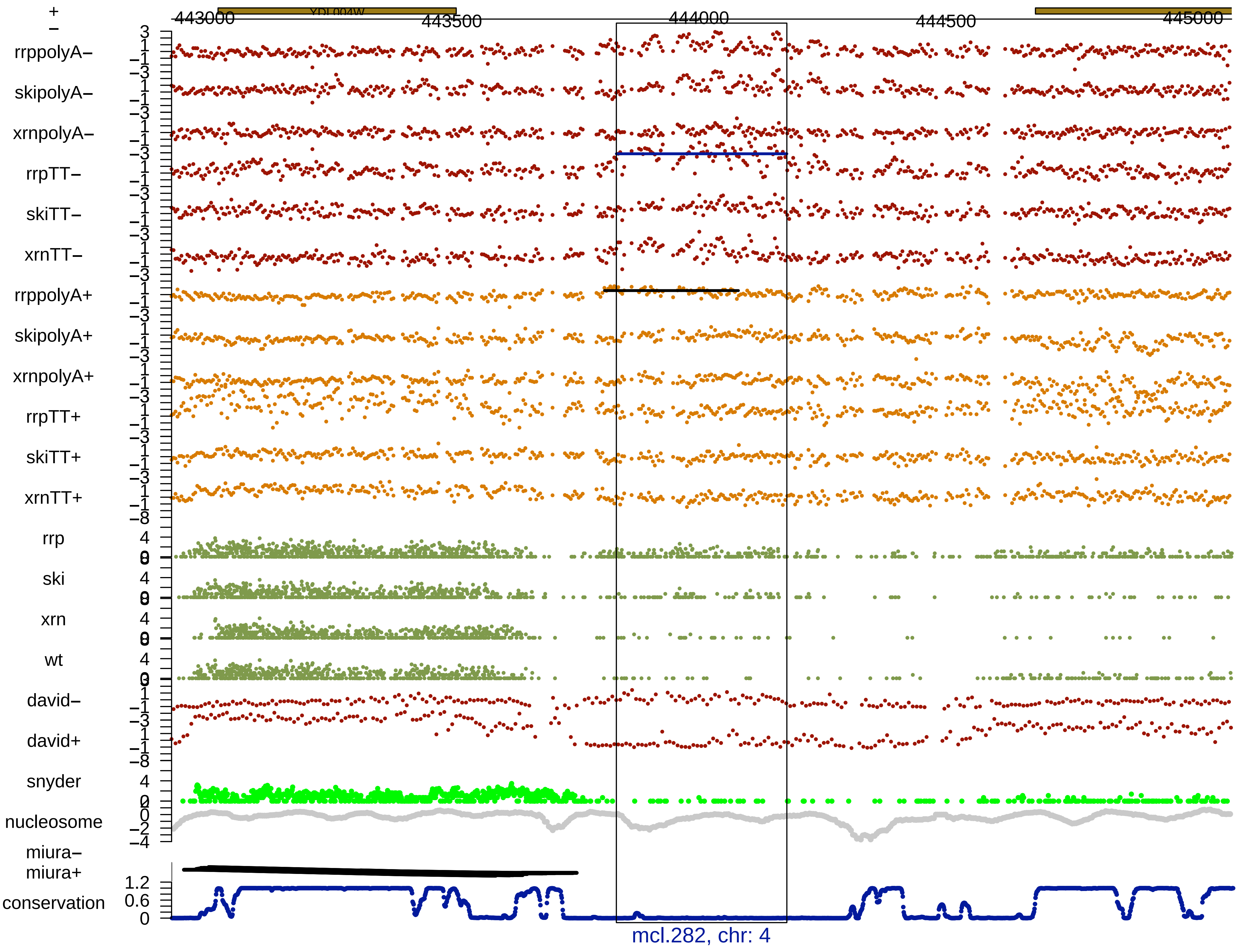

YDL004W
443000
443500
444000
444500
445000
rrppolyA−
skipolyA−
xrnpolyA−
rrpTT−
skiTT−
xrnTT−
rrppolyA+
skipolyA+
xrnpolyA+
rrpTT+
skiTT+
xrnTT+
rrp
ski
xrn
wt
david−
david+
snyder
nucleosome
miura−
miura+
conservation
mcl.282, chr: 4

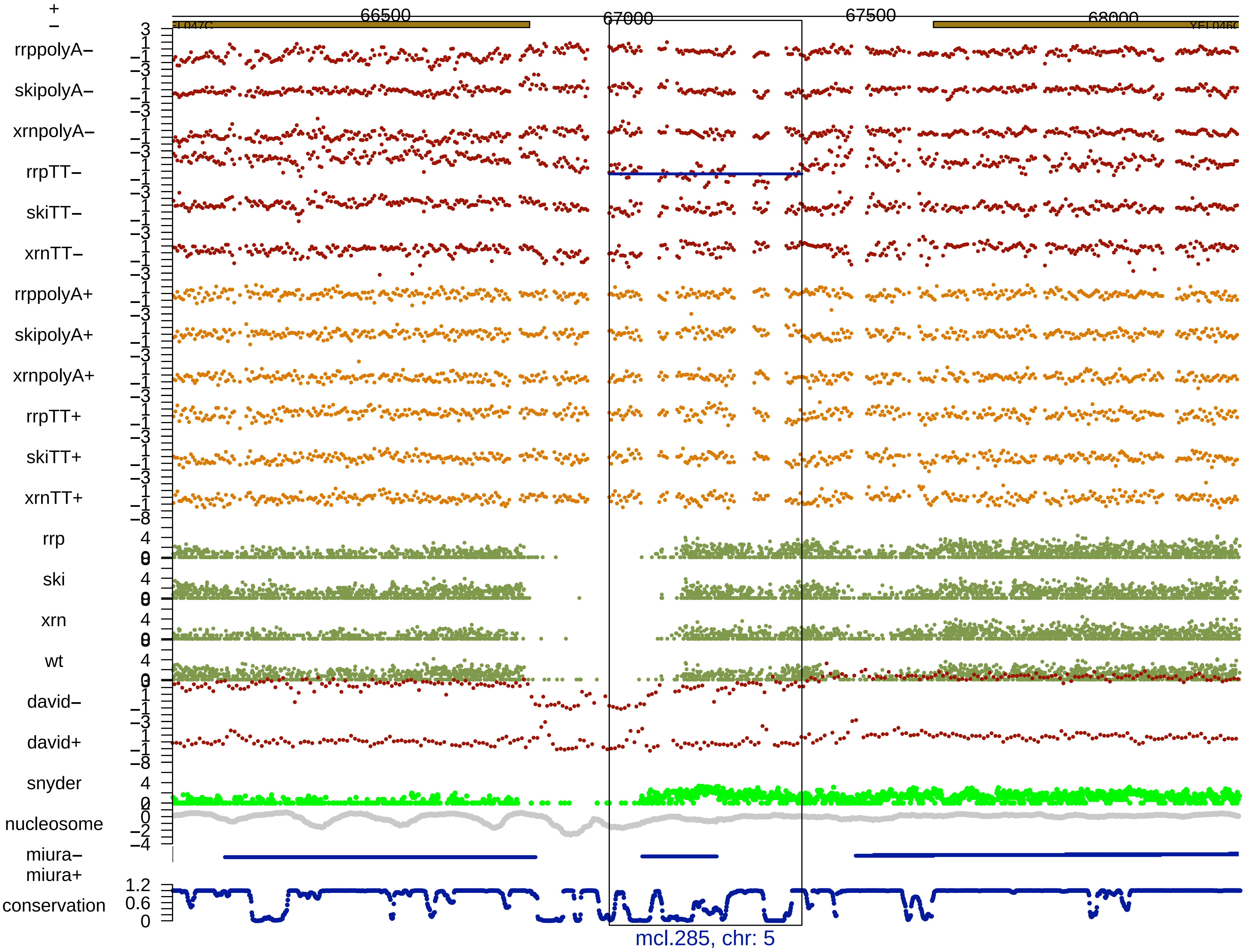
+
−
66500
67000
67500
68000
rrppolyA−
skipolyA−
xrnpolyA−
rrpTT−
skiTT−
xrnTT−
rrppolyA+
skipolyA+
xrnpolyA+
rrpTT+
skiTT+
xrnTT+
rrp
ski
xrn
wt
david−
david+
snyder
nucleosome
miura−
miura+
conservation
mcl.285, chr: 5

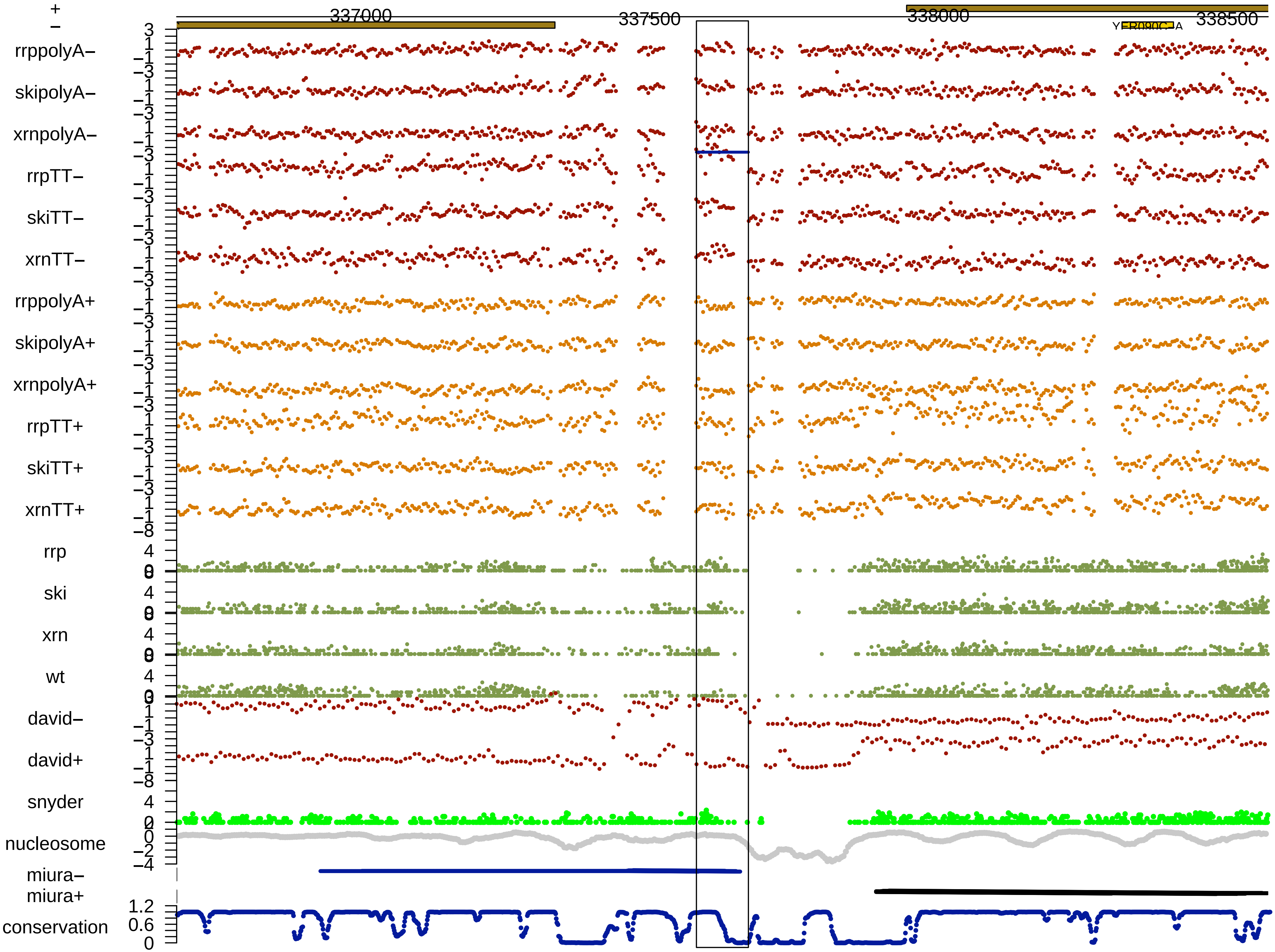

+
–
337000
337500
338000
338500
rrppolyA–
skipolyA–
xrnpolyA–
rrpTT–
skiTT–
xrnTT–
rrppolyA+
skipolyA+
xrnpolyA+
rrpTT+
skiTT+
xrnTT+
rrp
ski
xrn
wt
david–
david+
snyder
nucleosome
miura–
miura+
conservation
1.2
0.6
0

mcl.286, chr: 5

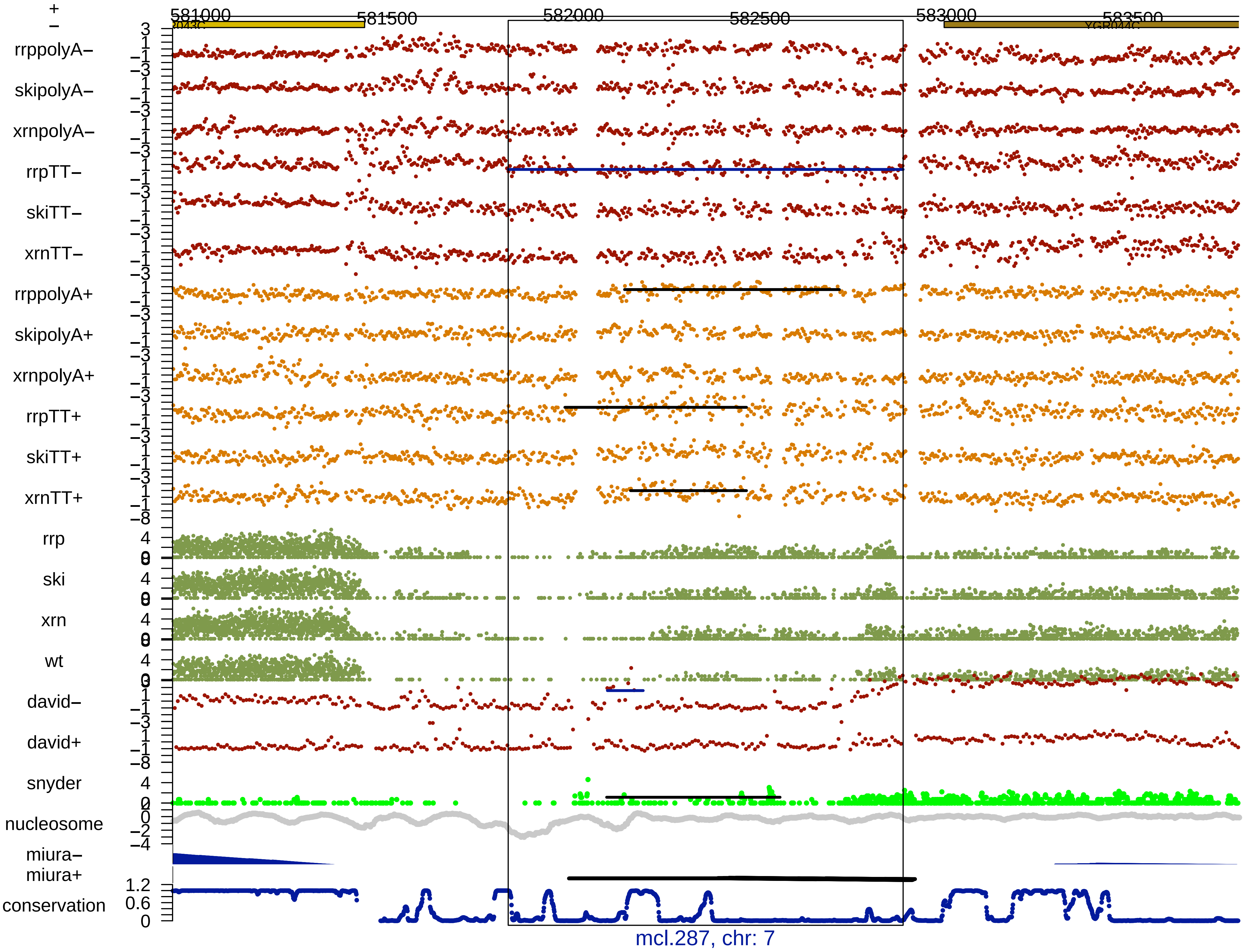
581000
581500
582000
582500
583000
583500
rrppolyA–
skipolyA–
xrnpolyA–
rrpTT–
skiTT–
xrnTT–
rrppolyA+
skipolyA+
xrnpolyA+
rrpTT+
skiTT+
xrnTT+
rrp
ski
xrn
wt
david–
david+
snyder
nucleosome
miura–
miura+
conservation
mcl.287, chr: 7

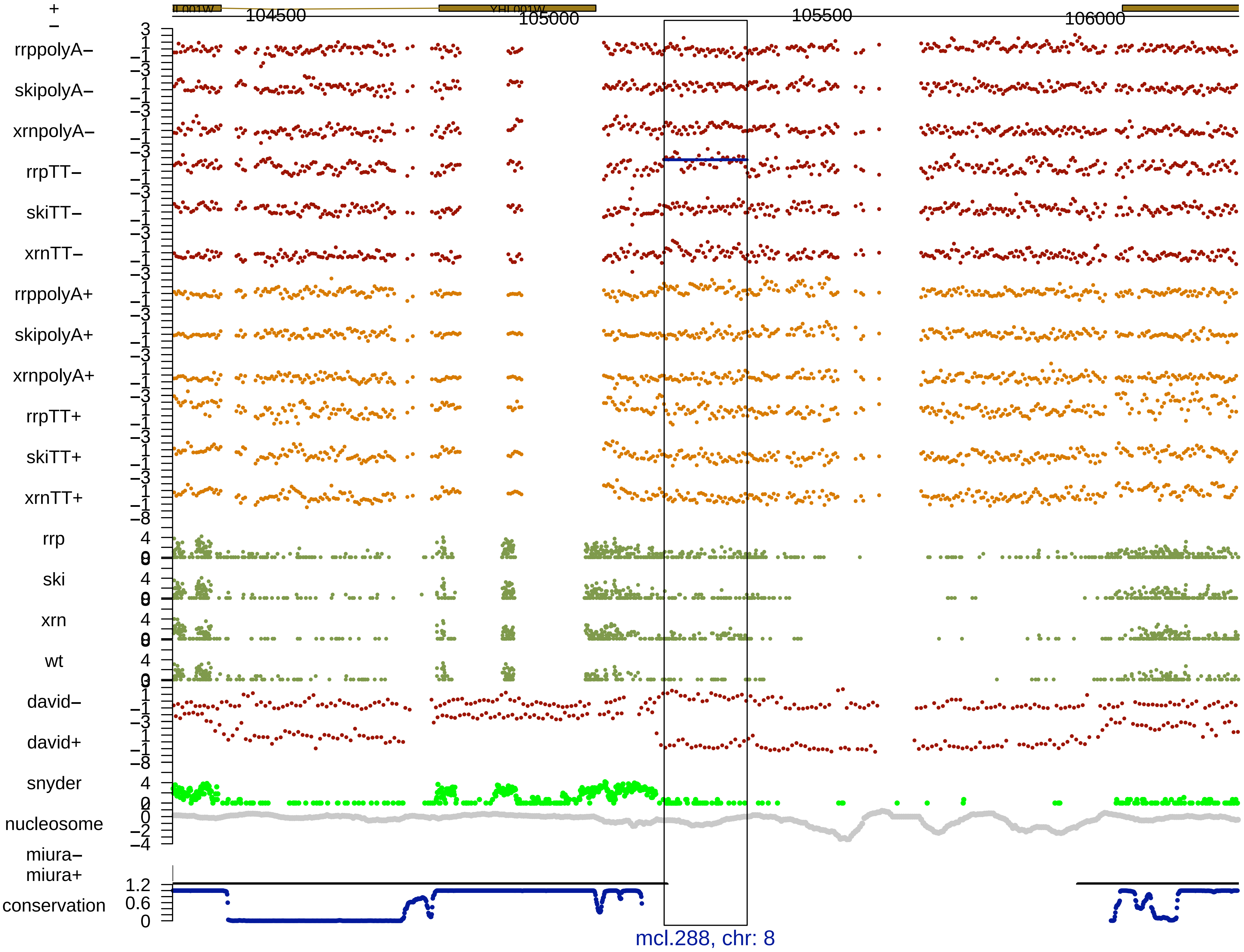

+
–
YHL001W
YHL001W
104500
105000
105500
106000
rrppolyA–
skipolyA–
xrnpolyA–
rrpTT–
skiTT–
xrnTT–
rrppolyA+
skipolyA+
xrnpolyA+
rrpTT+
skiTT+
xrnTT+
rrp
ski
xrn
wt
david–
david+
snyder
nucleosome
miura–
miura+
conservation
mcl.288, chr: 8

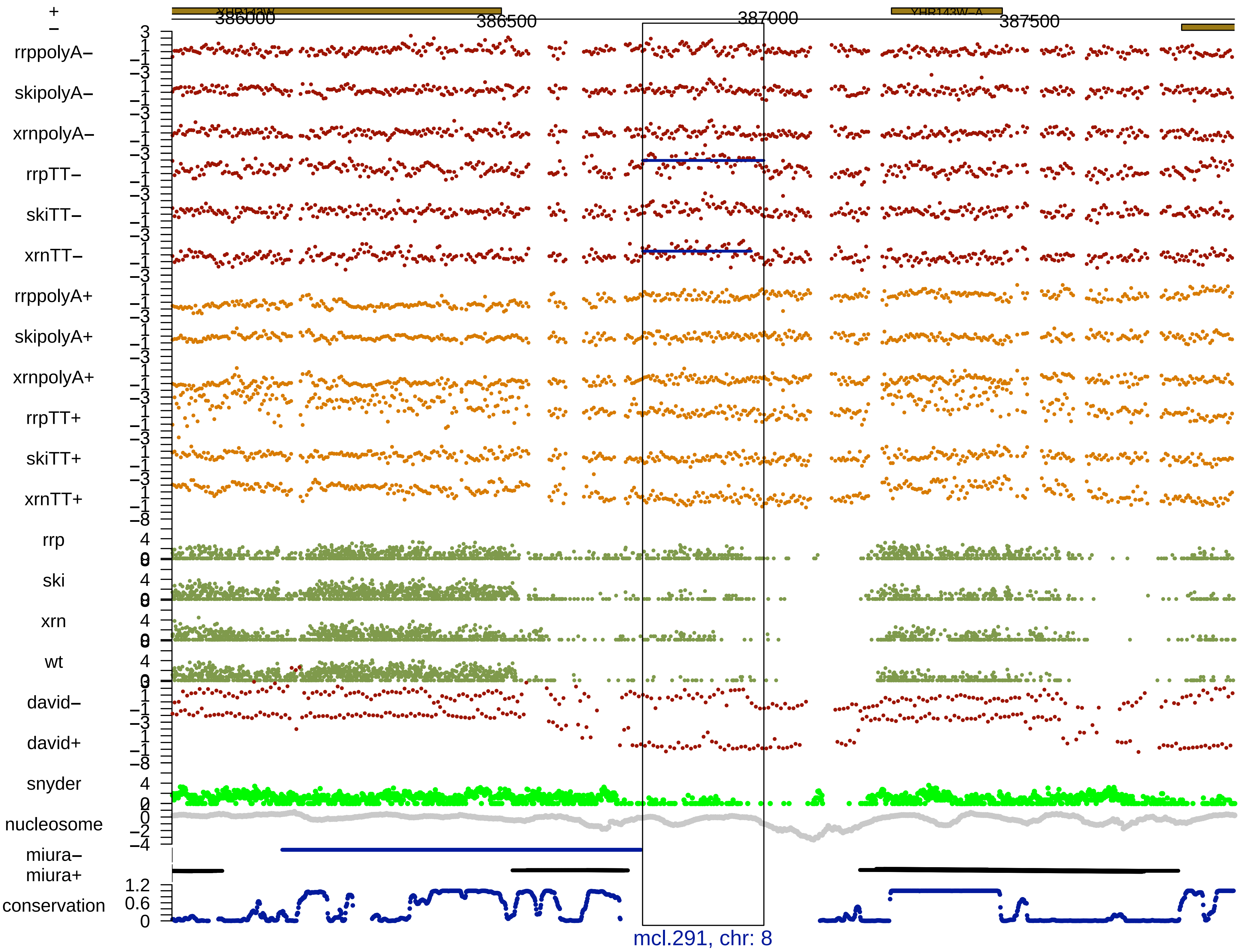

YHR142W
YHR143W-A
386000
386500
387000
387500
rrppolyA−
skipolyA−
xrnpolyA−
rrpTT−
skiTT−
xrnTT−
rrppolyA+
skipolyA+
xrnpolyA+
rrpTT+
skiTT+
xrnTT+
rrp
ski
xrn
wt
david−
david+
snyder
nucleosome
miura−
miura+
conservation
mcl.291, chr: 8

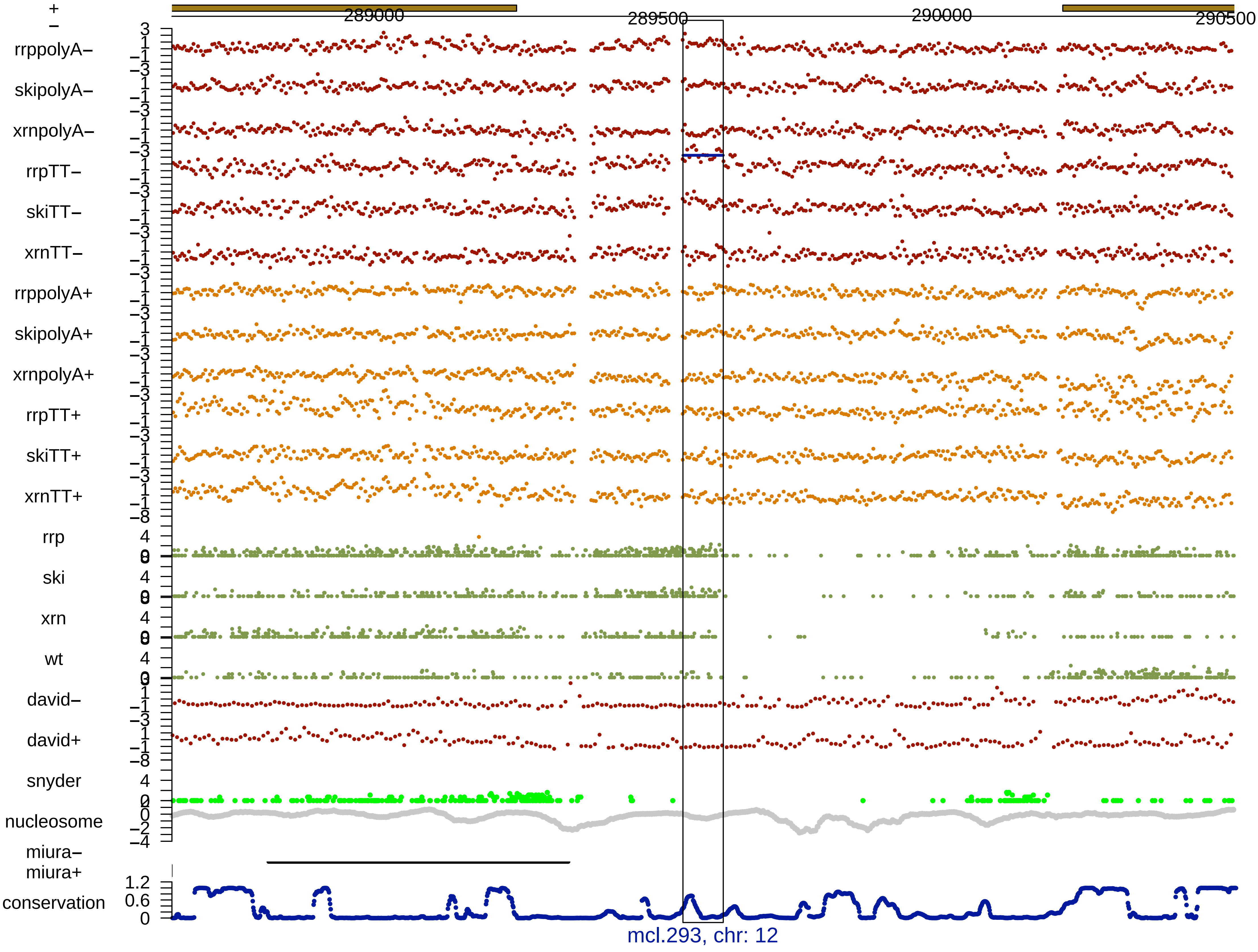

289000
289500
290000
290500
+
–
rrppolyA–
skipolyA–
xrnpolyA–
rrpTT–
skiTT–
xrnTT–
rrppolyA+
skipolyA+
xrnpolyA+
rrpTT+
skiTT+
xrnTT+
rrp
ski
xrn
wt
david–
david+
snyder
nucleosome
miura–
miura+
conservation
1.2
0.6
0
mcl.293, chr: 12

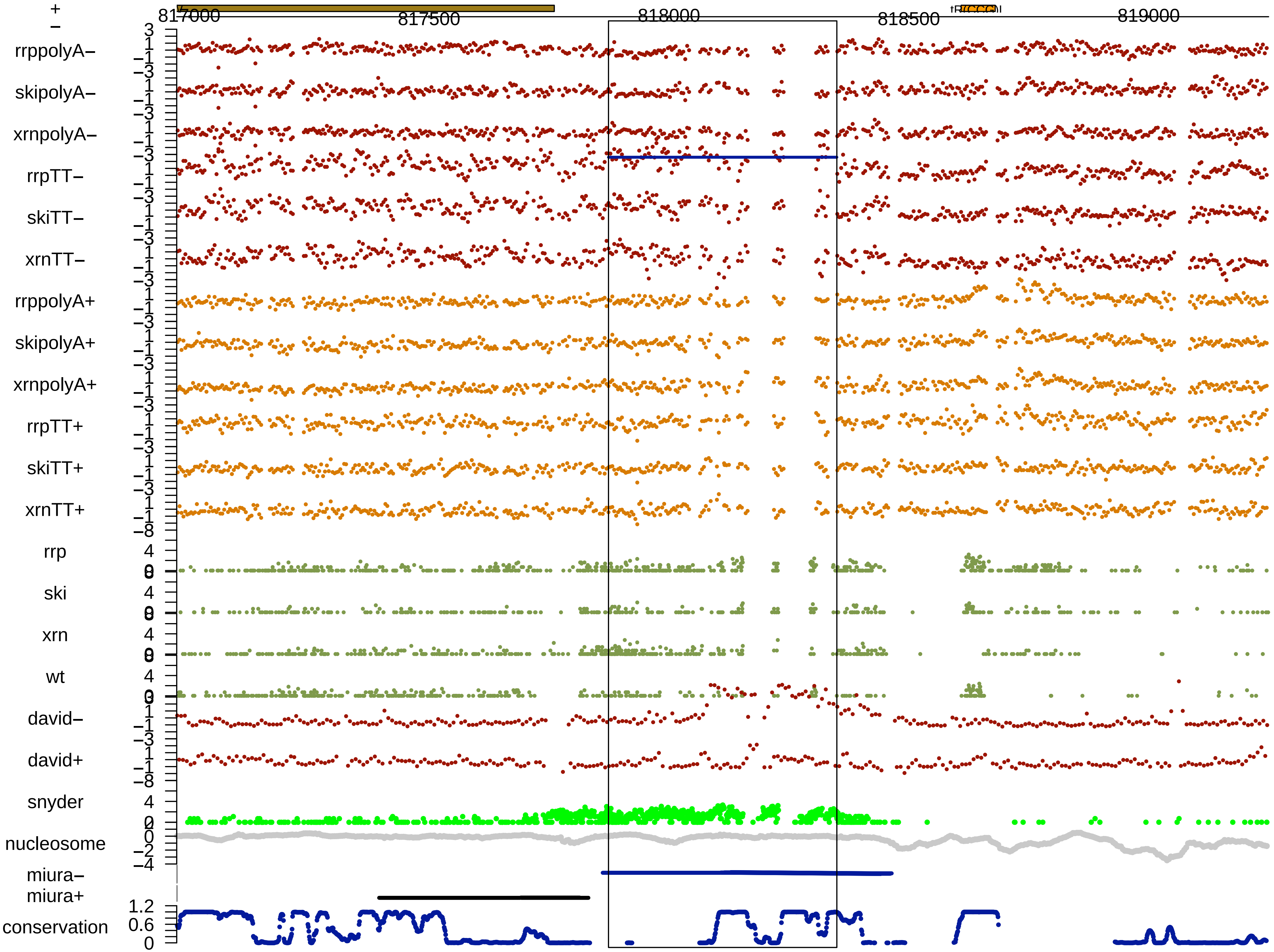

mcl.294, chr: 12

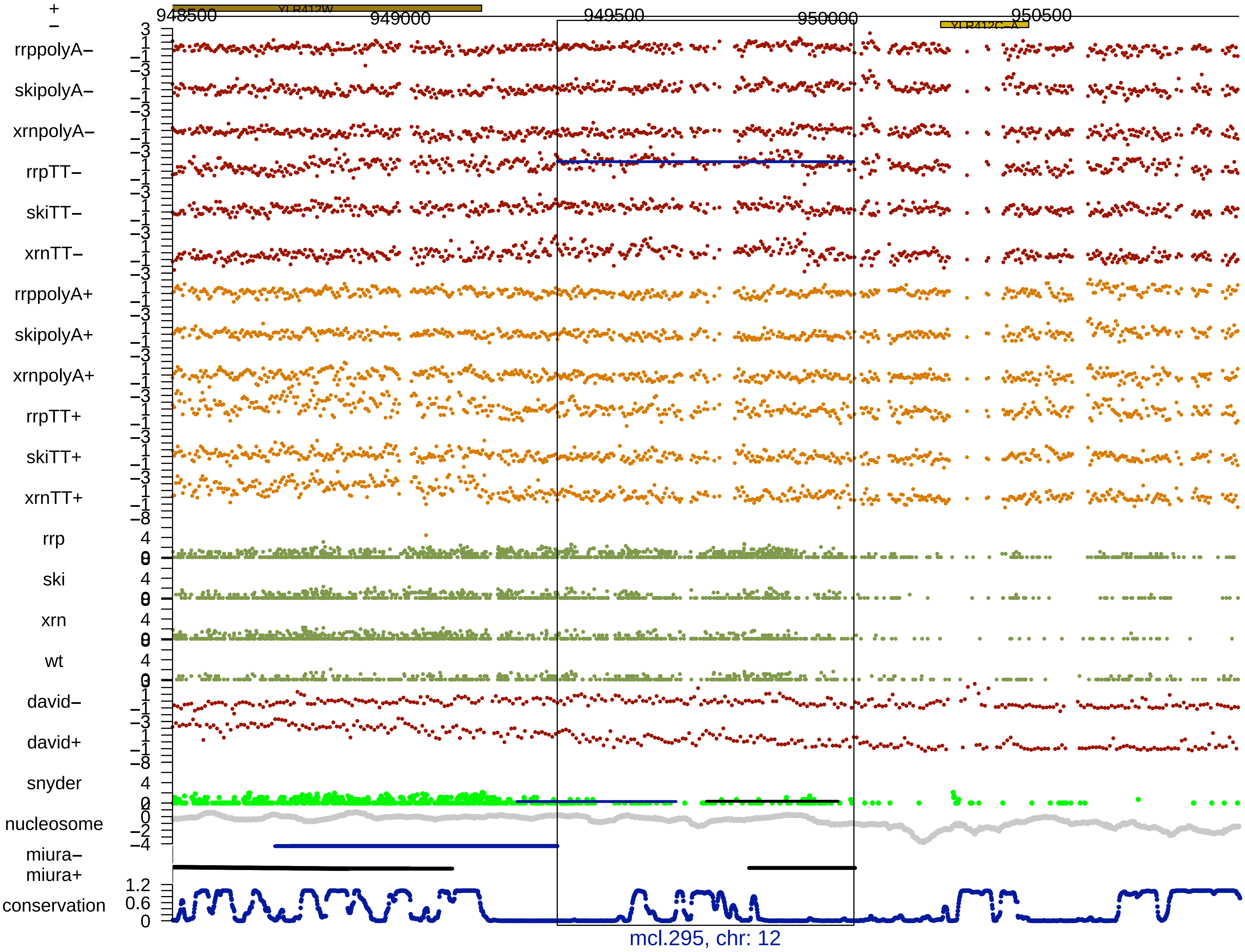
+
–
948500
949000
949500
950000
950500
YLR412W
YLR412C-A
rrppolyA–
skipolyA–
xrnpolyA–
rrpTT–
skiTT–
xrnTT–
rrppolyA+
skipolyA+
xrnpolyA+
rrpTT+
skiTT+
xrnTT+
rrp
ski
xrn
wt
david–
david+
snyder
nucleosome
miura–
miura+
conservation
mcl.295, chr: 12

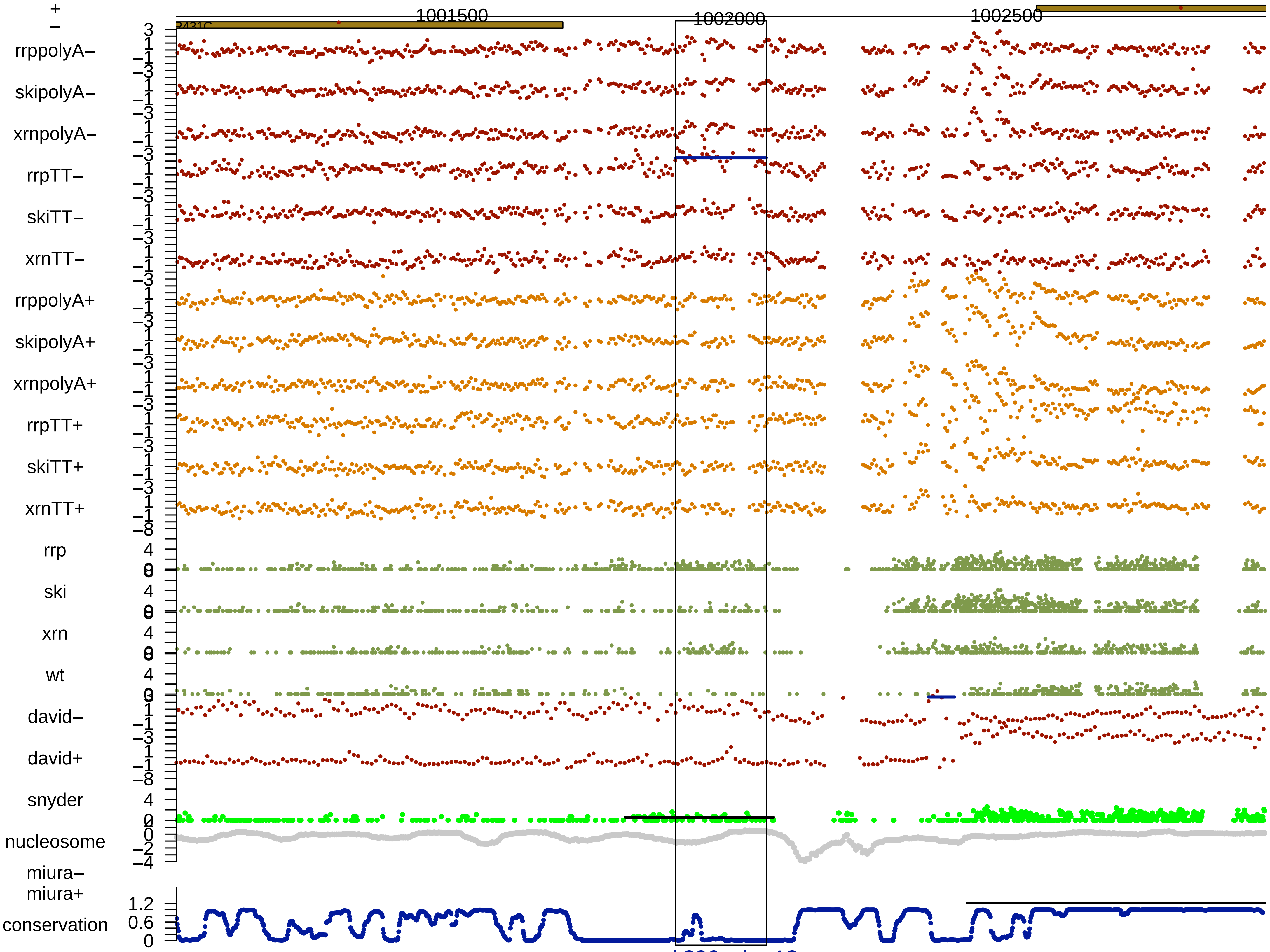

mcl.296, chr: 12

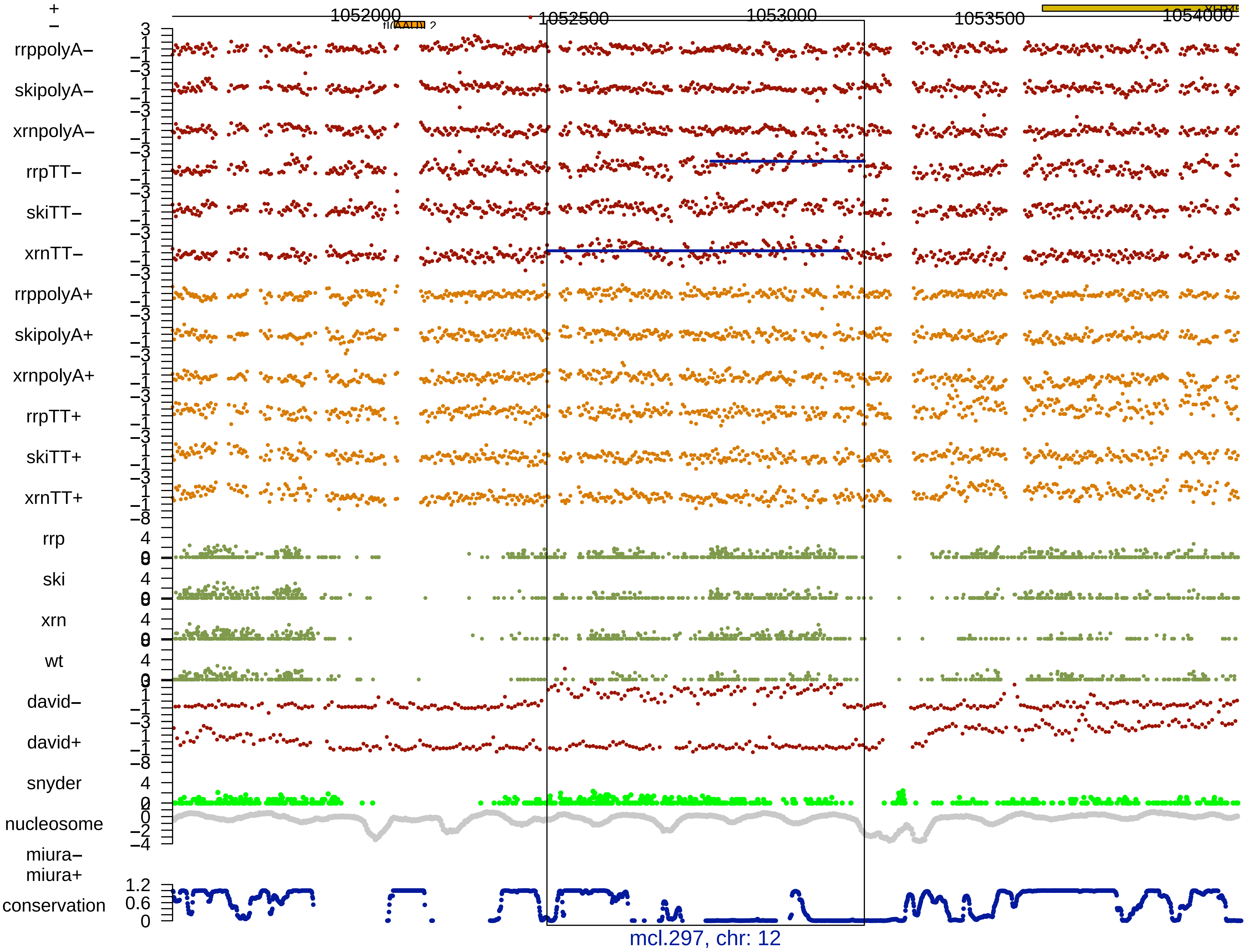

1052000
1052500
1053000
1053500
1054000
rrppolyA−
skipolyA−
xrnpolyA−
rrpTT−
skiTT−
xrnTT−
rrppolyA+
skipolyA+
xrnpolyA+
rrpTT+
skiTT+
xrnTT+
rrp
ski
xrn
wt
david−
david+
snyder
nucleosome
miura−
miura+
conservation
mcl.297, chr: 12

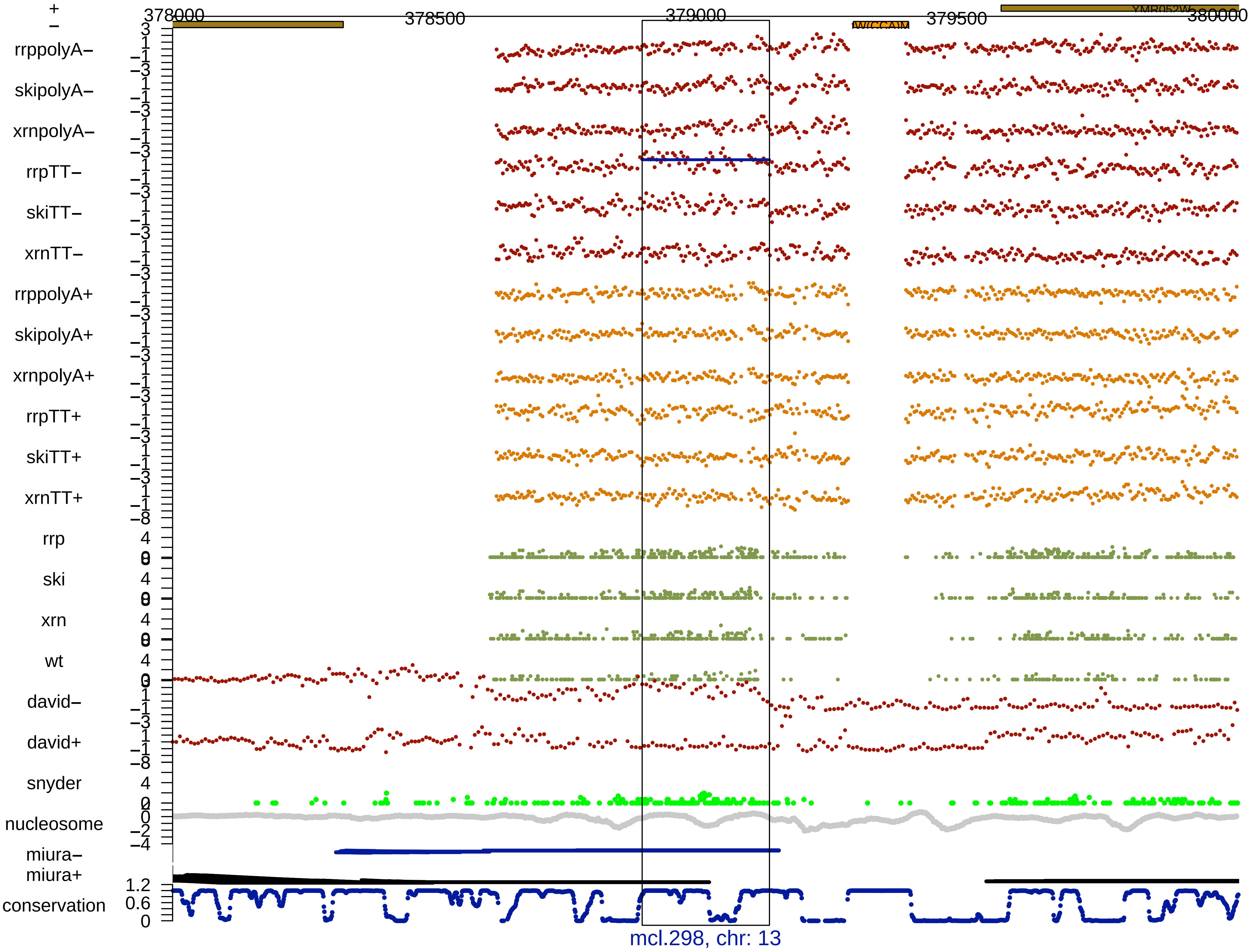
+
–
378000
378500
379000
379500
380000
YMR052W
rrppolyA–
skipolyA–
xrnpolyA–
rrpTT–
skiTT–
xrnTT–
rrppolyA+
skipolyA+
xrnpolyA+
rrpTT+
skiTT+
xrnTT+
rrp
ski
xrn
wt
david–
david+
snyder
nucleosome
miura–
miura+
conservation
mcl.298, chr: 13

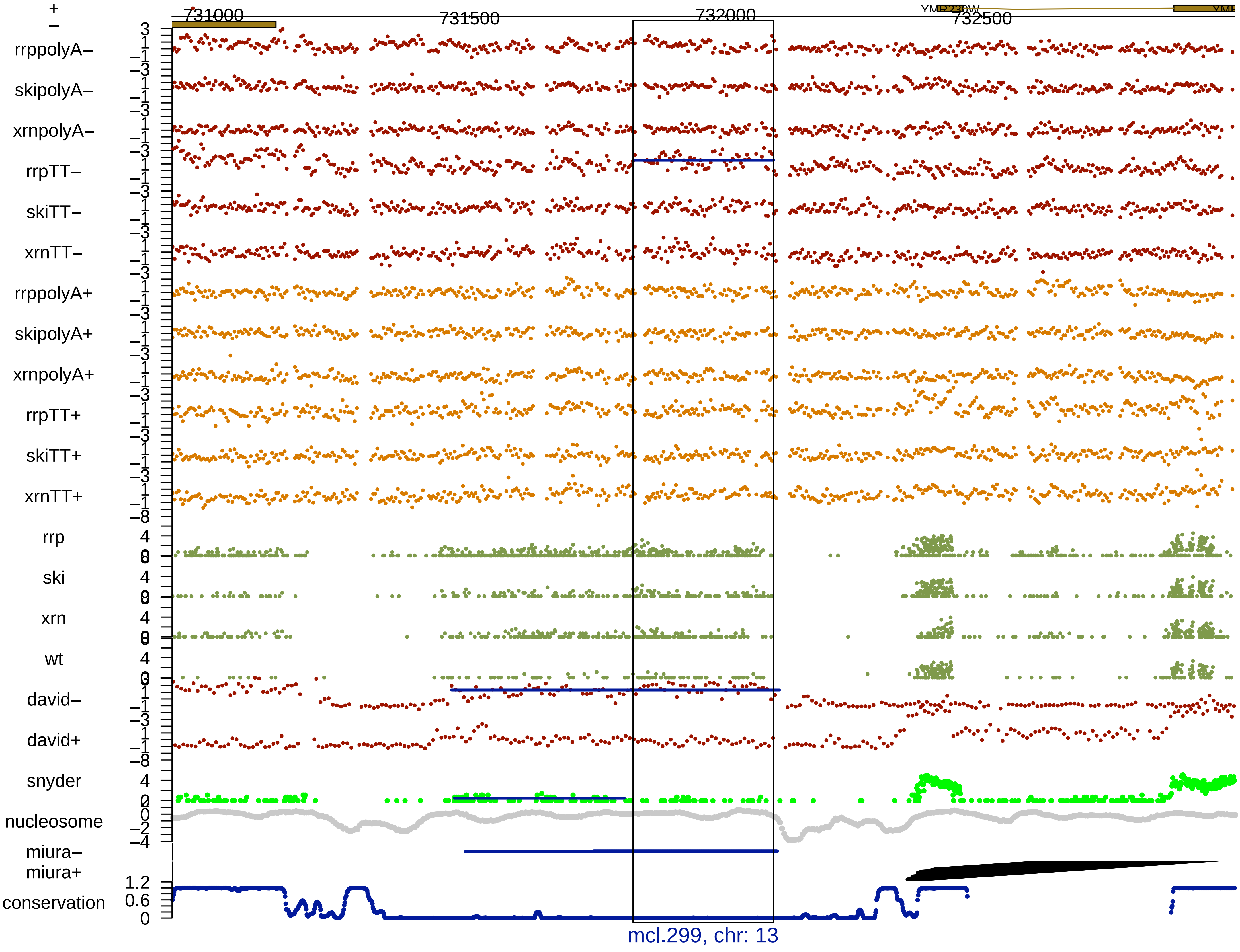

731000
731500
732000
732500
YMR230W
rrppolyA−
skipolyA−
xrnpolyA−
rrpTT−
skiTT−
xrnTT−
rrppolyA+
skipolyA+
xrnpolyA+
rrpTT+
skiTT+
xrnTT+
rrp
ski
xrn
wt
david−
david+
snyder
nucleosome
miura−
miura+
conservation
mcl.299, chr: 13

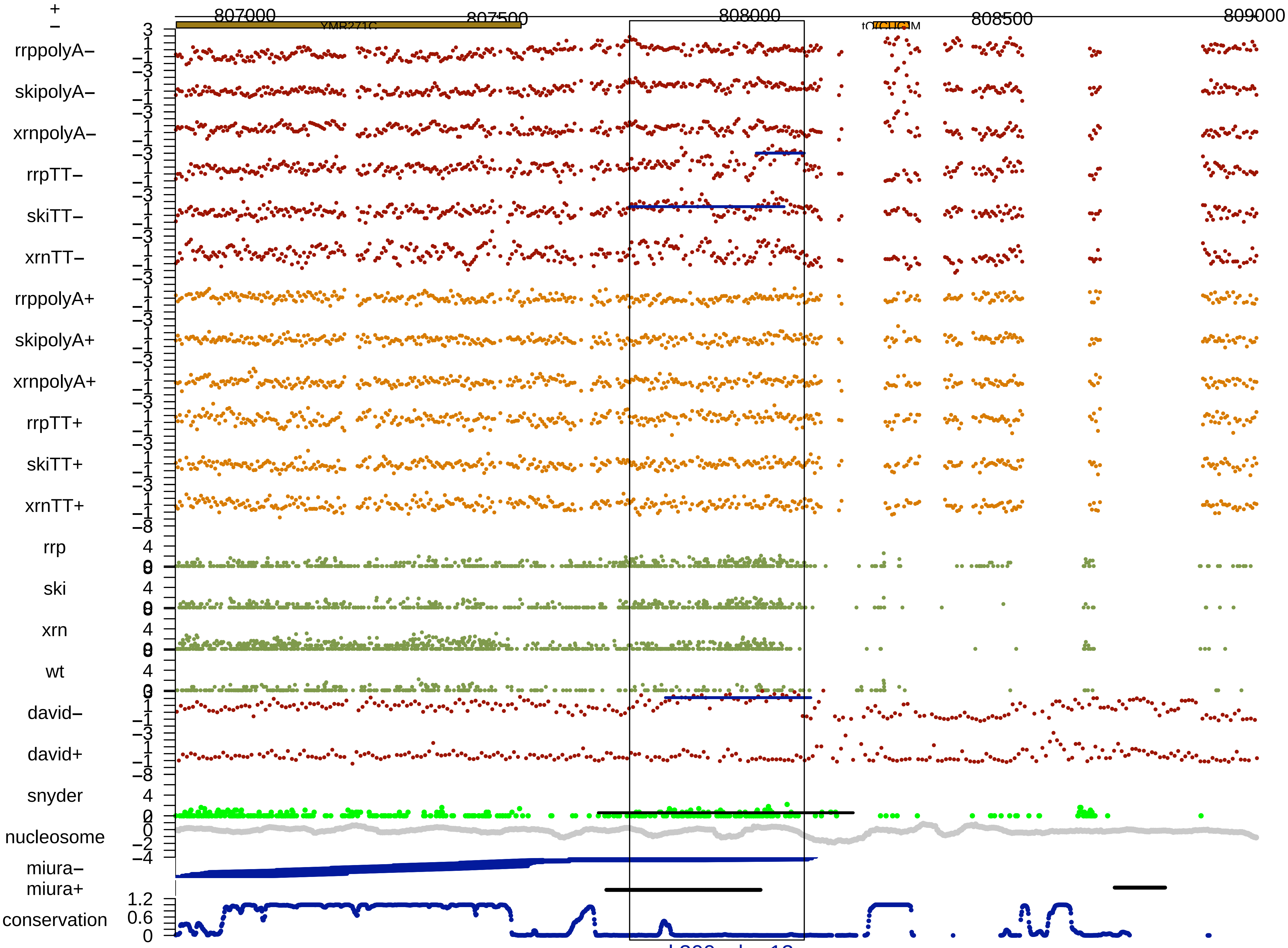

807000
807500
808000
808500
809000
YMR271C
rrppolyA−
skipolyA−
xrnpolyA−
rrpTT−
skiTT−
xrnTT−
rrppolyA+
skipolyA+
xrnpolyA+
rrpTT+
skiTT+
xrnTT+
rrp
ski
xrn
wt
david−
david+
snyder
nucleosome
miura−
miura+
conservation
mcl.300, chr: 13

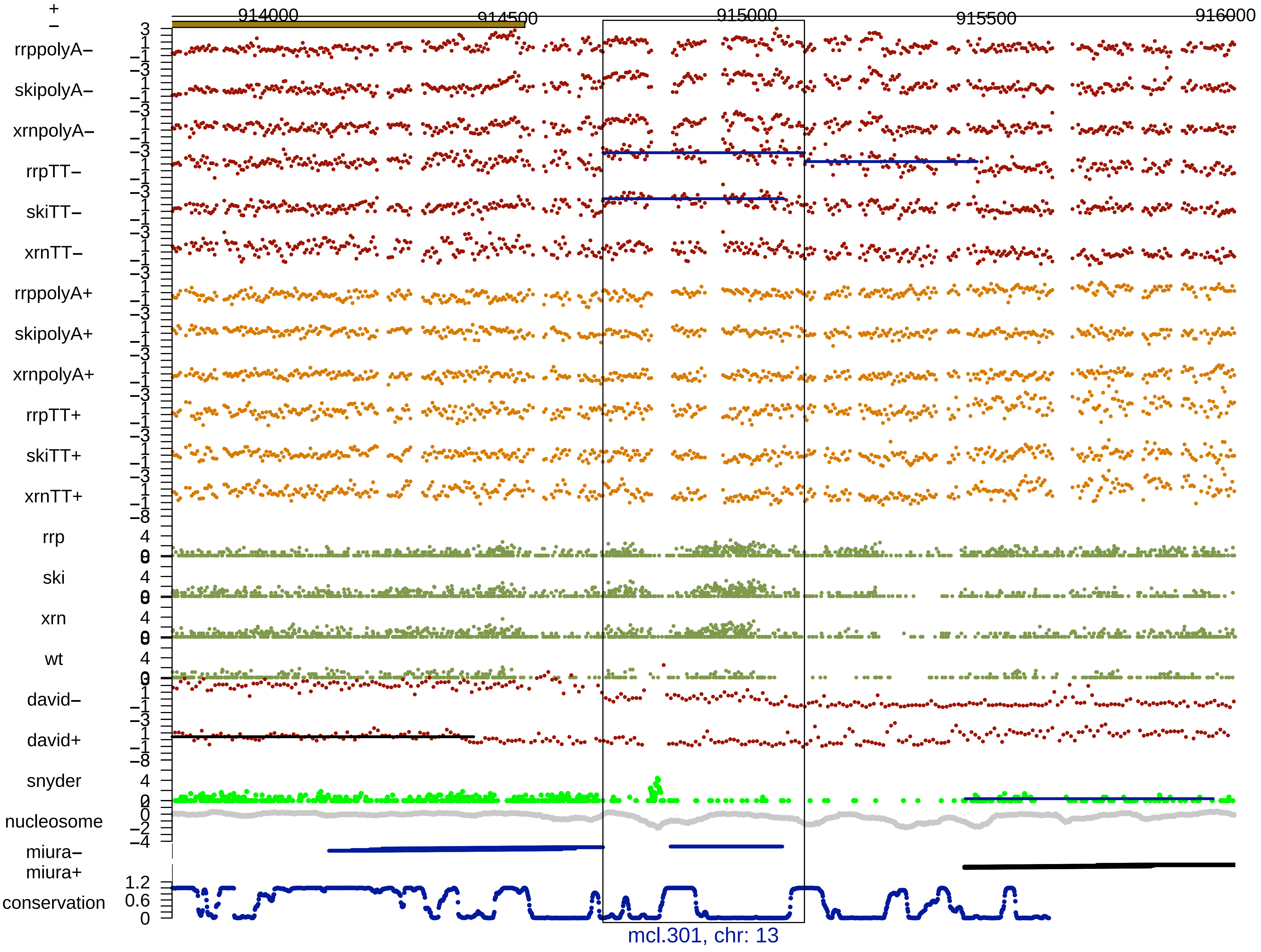

914000
914500
915000
915500
916000
rrppolyA−
skipolyA−
xrnpolyA−
rrpTT−
skiTT−
xrnTT−
rrppolyA+
skipolyA+
xrnpolyA+
rrpTT+
skiTT+
xrnTT+
rrp
ski
xrn
wt
david−
david+
snyder
nucleosome
miura−
miura+
conservation
mcl.301, chr: 13

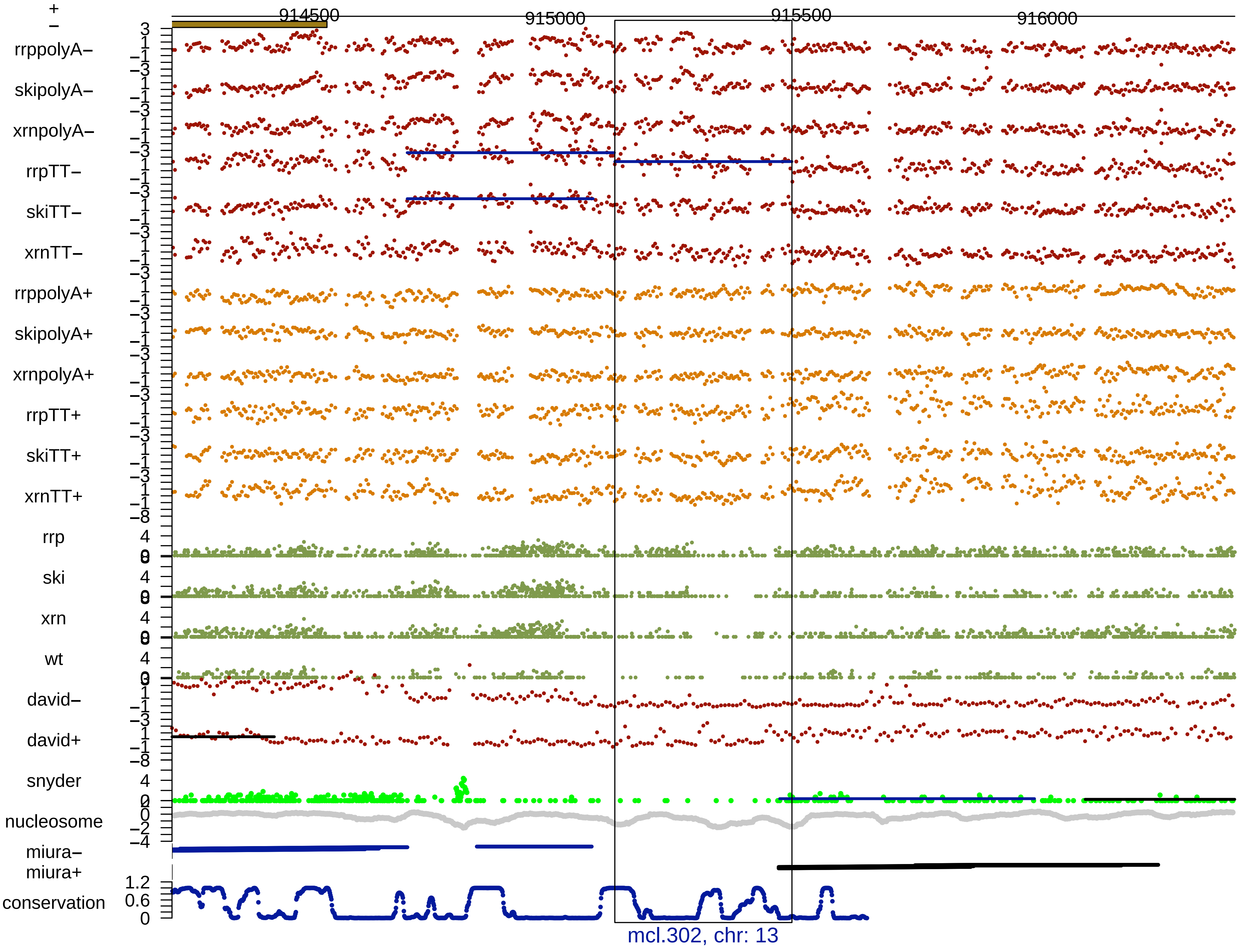

914500
915000
915500
916000
rrppolyA−
skipolyA−
xrnpolyA−
rrpTT−
skiTT−
xrnTT−
rrppolyA+
skipolyA+
xrnpolyA+
rrpTT+
skiTT+
xrnTT+
rrp
ski
xrn
wt
david−
david+
snyder
nucleosome
miura−
miura+
conservation
mcl.302, chr: 13

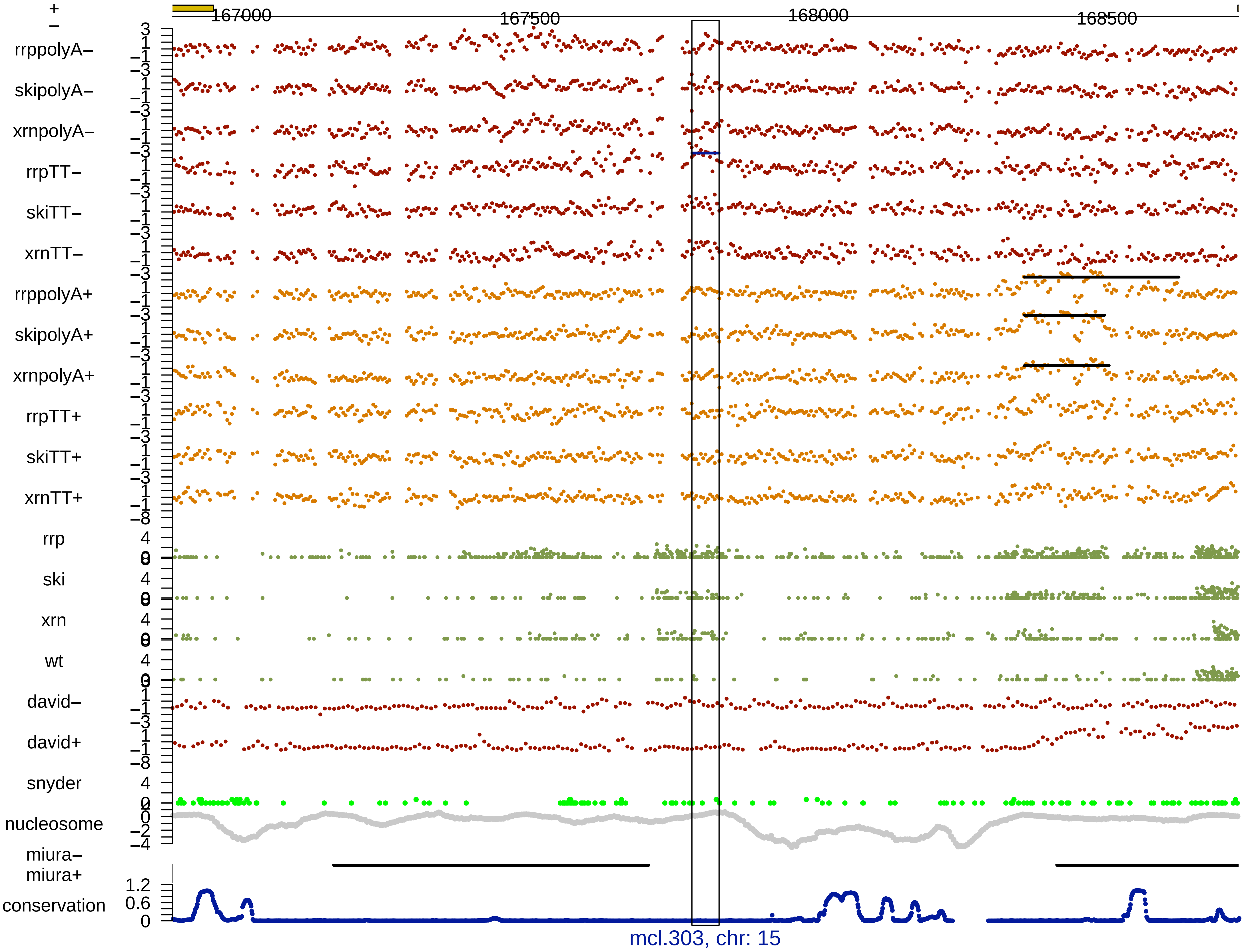
+
−
167000
167500
168000
168500
rrppolyA−
skipolyA−
xrnpolyA−
rrpTT−
skiTT−
xrnTT−
rrppolyA+
skipolyA+
xrnpolyA+
rrpTT+
skiTT+
xrnTT+
rrp
ski
xrn
wt
david−
david+
snyder
nucleosome
miura−
miura+
conservation
3
1
−1
−3
−8
4
0
2
−2
−4
1.2
0.6
mcl.303, chr: 15

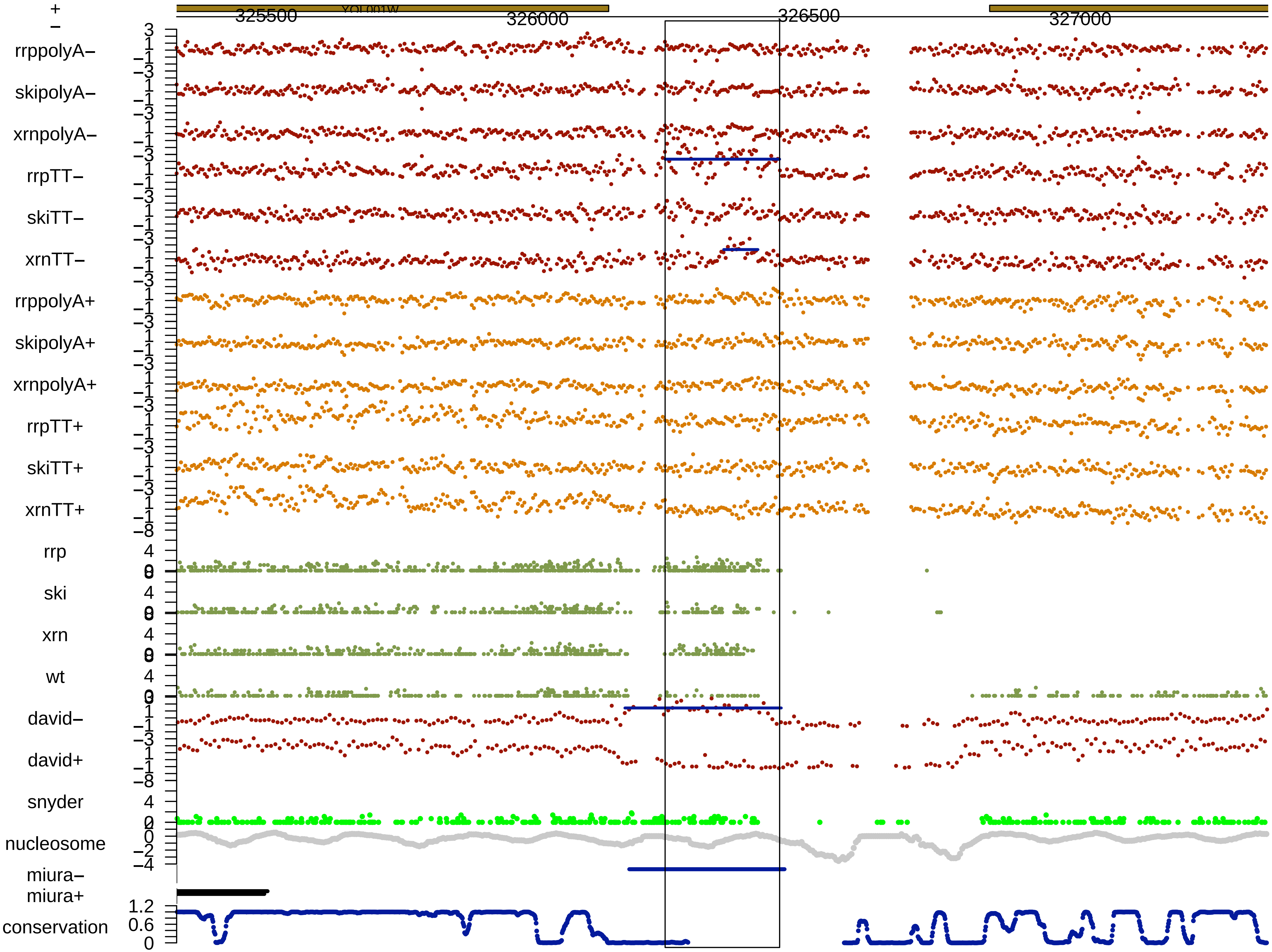

mcl.304, chr: 15

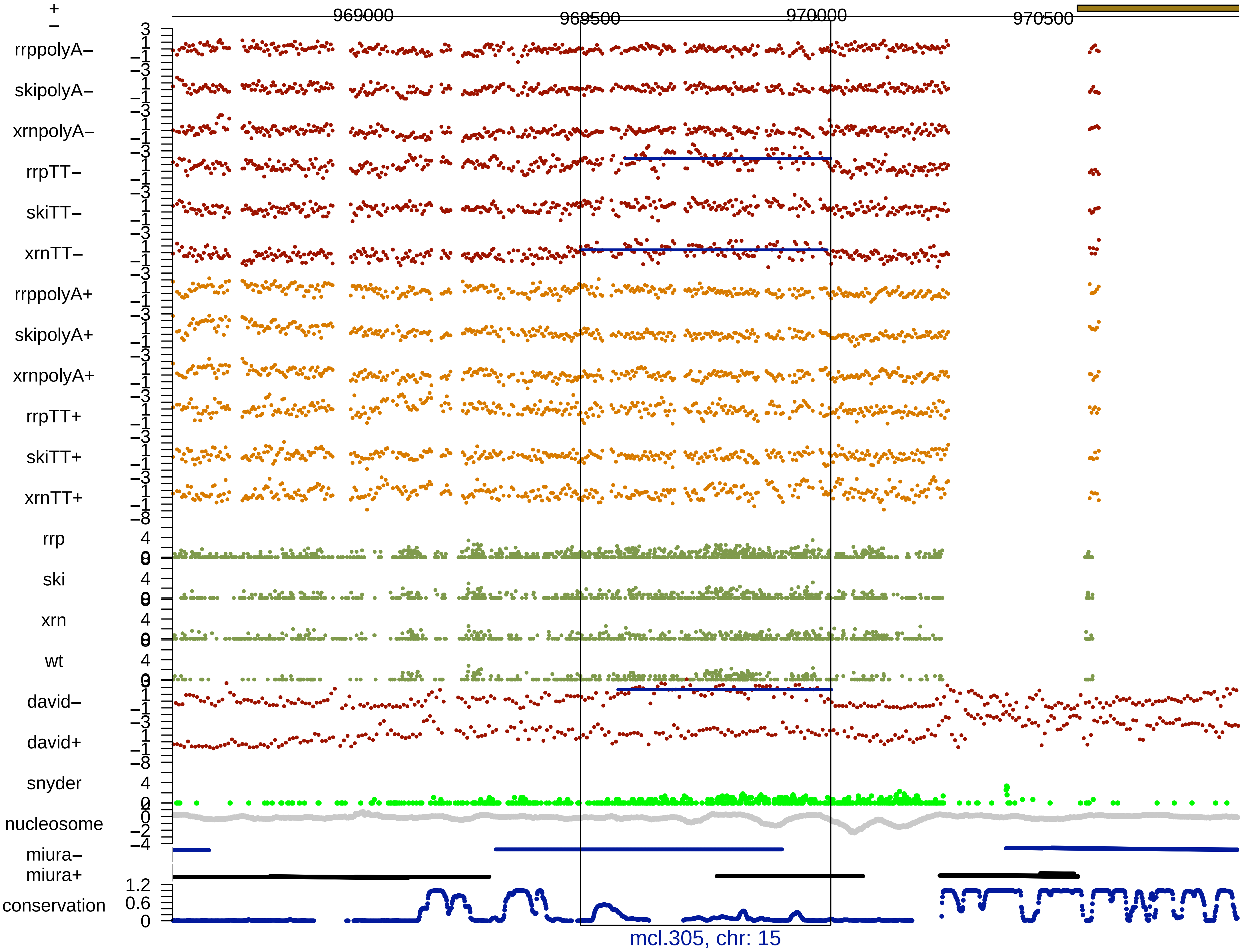
+
−
969000
969500
970000
970500
rrppolyA−
skipolyA−
xrnpolyA−
rrpTT−
skiTT−
xrnTT−
rrppolyA+
skipolyA+
xrnpolyA+
rrpTT+
skiTT+
xrnTT+
rrp
ski
xrn
wt
david−
david+
snyder
nucleosome
miura−
miura+
conservation
mcl.305, chr: 15

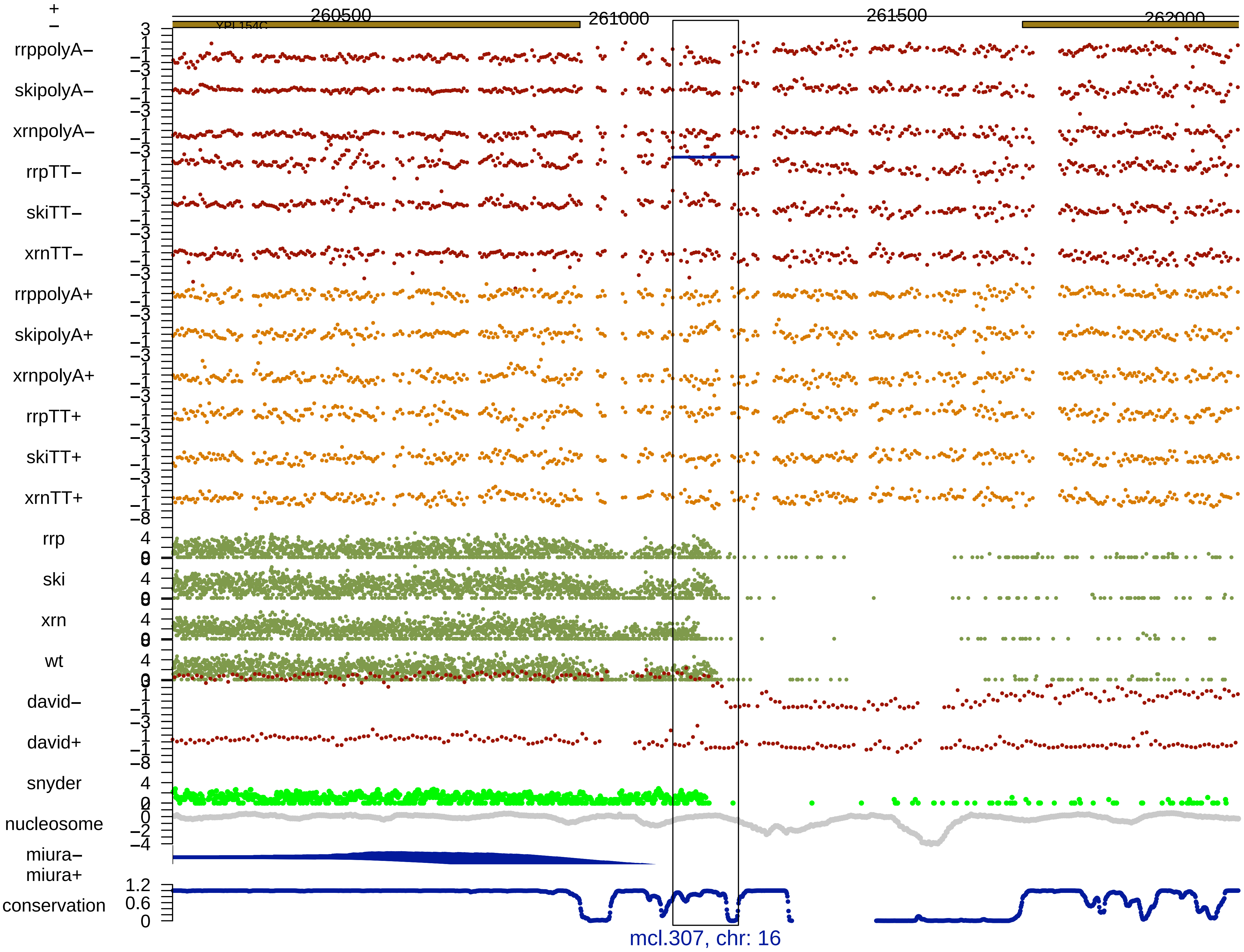

260500
261000
261500
262000
YPL154C
rrppolyA−
skipolyA−
xrnpolyA−
rrpTT−
skiTT−
xrnTT−
rrppolyA+
skipolyA+
xrnpolyA+
rrpTT+
skiTT+
xrnTT+
rrp
ski
xrn
wt
david−
david+
snyder
nucleosome
miura−
miura+
conservation
mcl.307, chr: 16

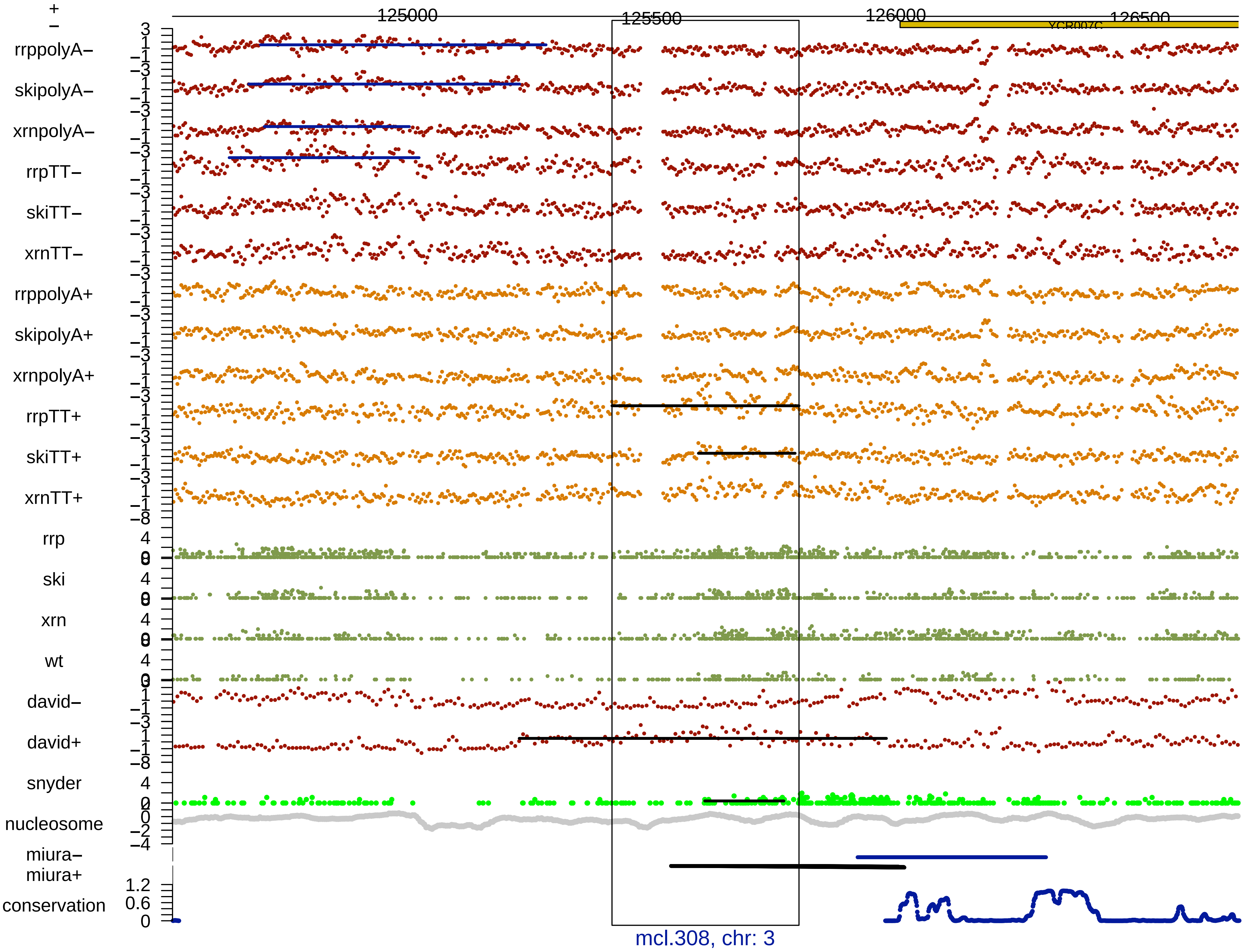

125000
125500
126000
126500
YCR007C
rrppolyA−
skipolyA−
xrnpolyA−
rrpTT−
skiTT−
xrnTT−
rrppolyA+
skipolyA+
xrnpolyA+
rrpTT+
skiTT+
xrnTT+
rrp
ski
xrn
wt
david−
david+
snyder
nucleosome
miura−
miura+
conservation
mcl.308, chr: 3

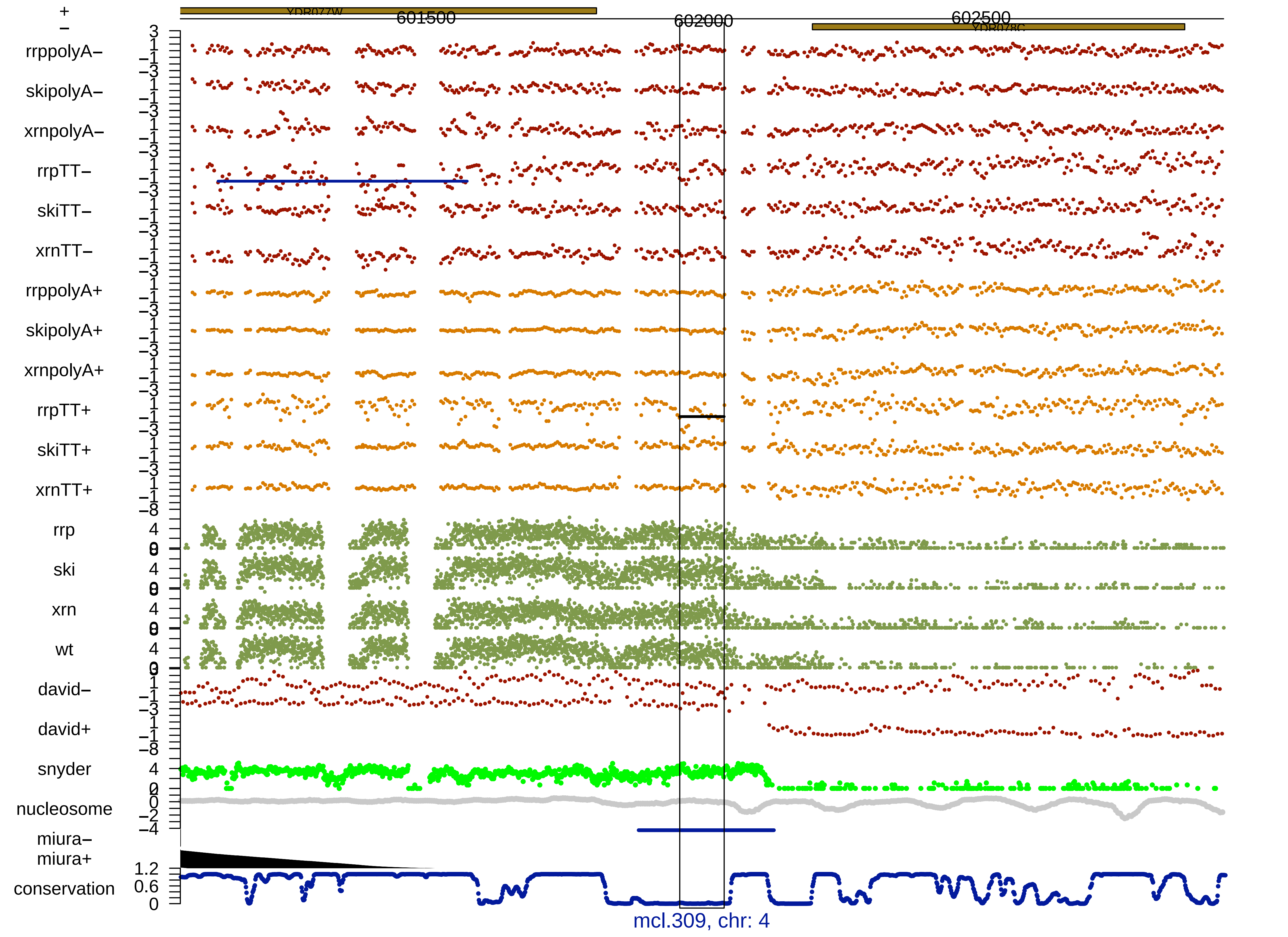

+
–
YDR077W
601500
602000
602500
YDR078C
rrppolyA–
skipolyA–
xrnpolyA–
rrpTT–
skiTT–
xrnTT–
rrppolyA+
skipolyA+
xrnpolyA+
rrpTT+
skiTT+
xrnTT+
rrp
ski
xrn
wt
david–
david+
snyder
nucleosome
miura–
miura+
conservation
mcl.309, chr: 4

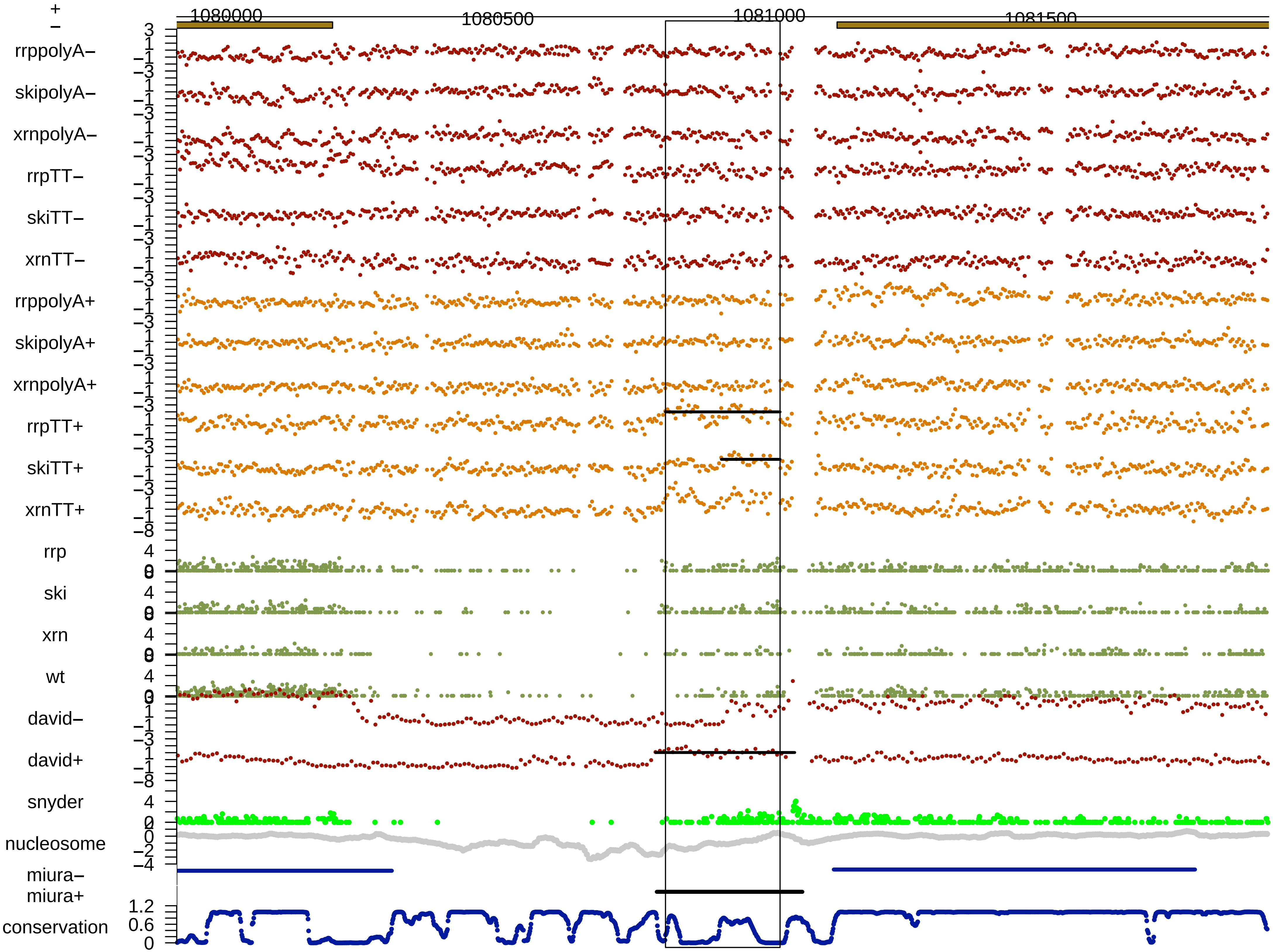
1080000
1080500
1081000
1081500
+
−
rrppolyA−
skipolyA−
xrnpolyA−
rrpTT−
skiTT−
xrnTT−
rrppolyA+
skipolyA+
xrnpolyA+
rrpTT+
skiTT+
xrnTT+
rrp
ski
xrn
wt
david−
david+
snyder
nucleosome
miura−
miura+
conservation
mcl.310, chr: 4

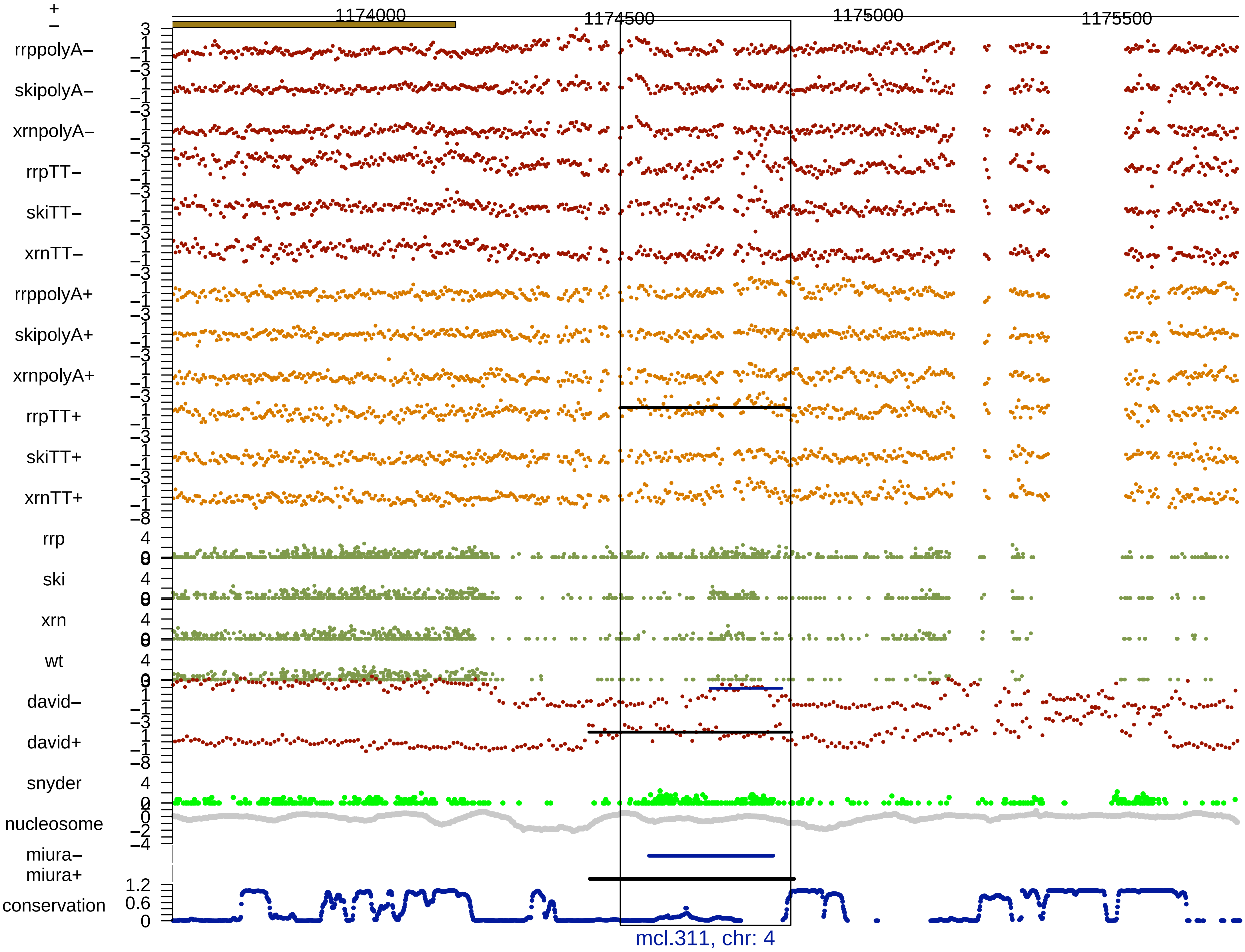

+
−
1174000
1174500
1175000
1175500
rrppolyA−
skipolyA−
xrnpolyA−
rrpTT−
skiTT−
xrnTT−
rrppolyA+
skipolyA+
xrnpolyA+
rrpTT+
skiTT+
xrnTT+
rrp
ski
xrn
wt
david−
david+
snyder
nucleosome
miura−
miura+
conservation
mcl.311, chr: 4

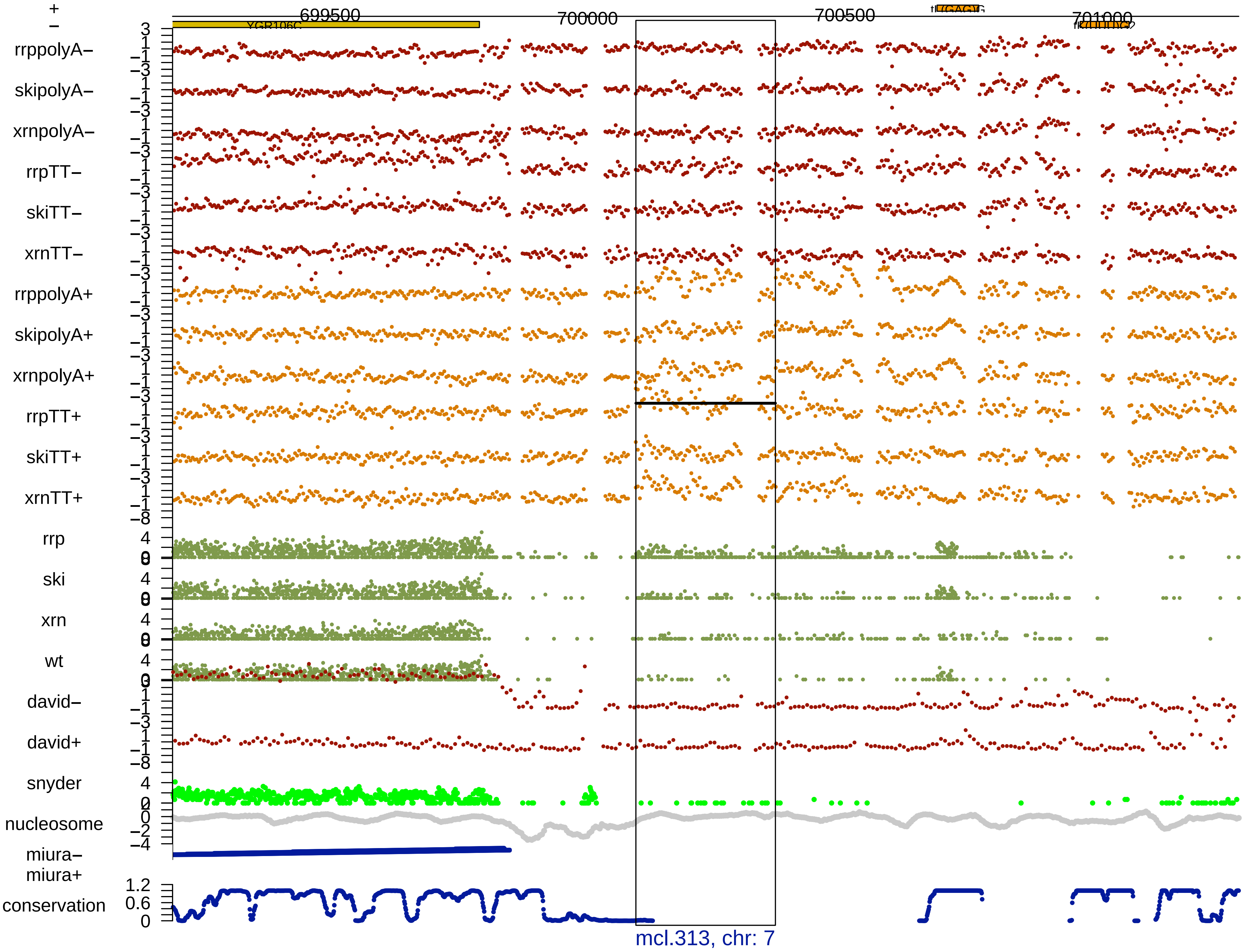

YGR106C
699500
700000
700500
701000
rrppolyA−
skipolyA−
xrnpolyA−
rrpTT−
skiTT−
xrnTT−
rrppolyA+
skipolyA+
xrnpolyA+
rrpTT+
skiTT+
xrnTT+
rrp
ski
xrn
wt
david−
david+
snyder
nucleosome
miura−
miura+
conservation
mcl.313, chr: 7

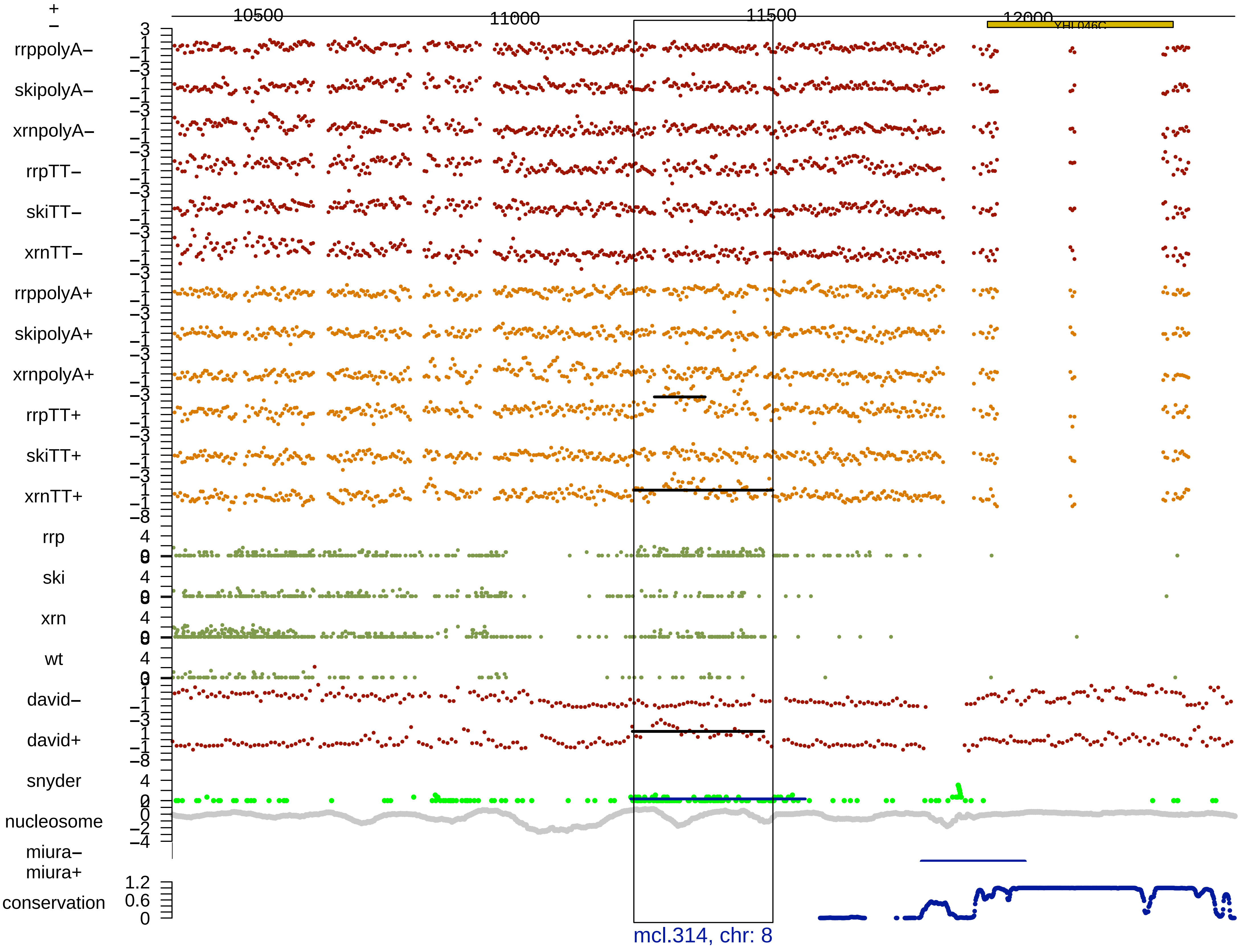

+
–
10500
11000
11500
12000
YHL046C
rrppolyA–
skipolyA–
xrnpolyA–
rrpTT–
skiTT–
xrnTT–
rrppolyA+
skipolyA+
xrnpolyA+
rrpTT+
skiTT+
xrnTT+
rrp
ski
xrn
wt
david–
david+
snyder
nucleosome
miura–
miura+
conservation
1.2
0.6
0
mcl.314, chr: 8

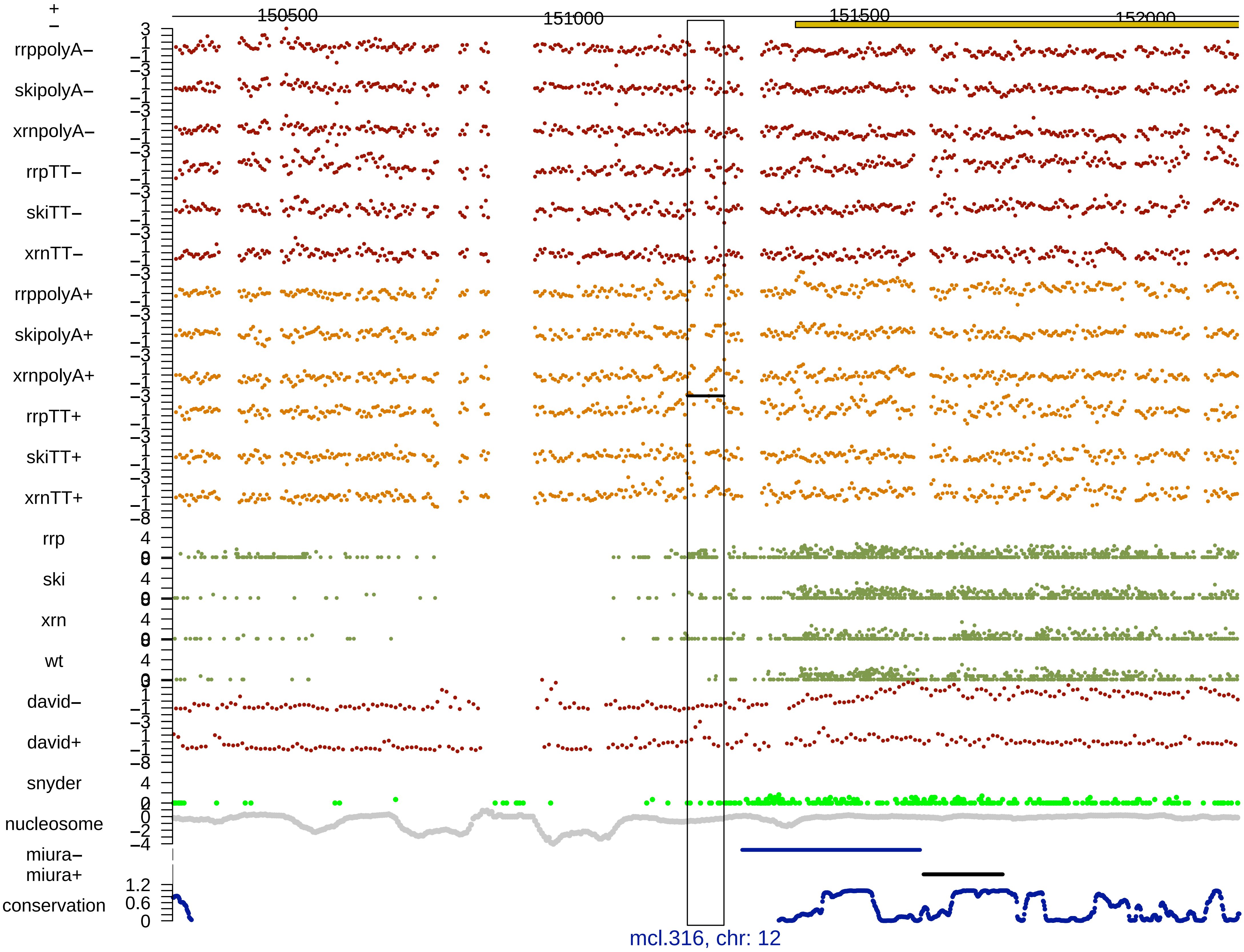

150500
151000
151500
152000
rrppolyA−
skipolyA−
xrnpolyA−
rrpTT−
skiTT−
xrnTT−
rrppolyA+
skipolyA+
xrnpolyA+
rrpTT+
skiTT+
xrnTT+
rrp
ski
xrn
wt
david−
david+
snyder
nucleosome
miura−
miura+
conservation
mcl.316, chr: 12

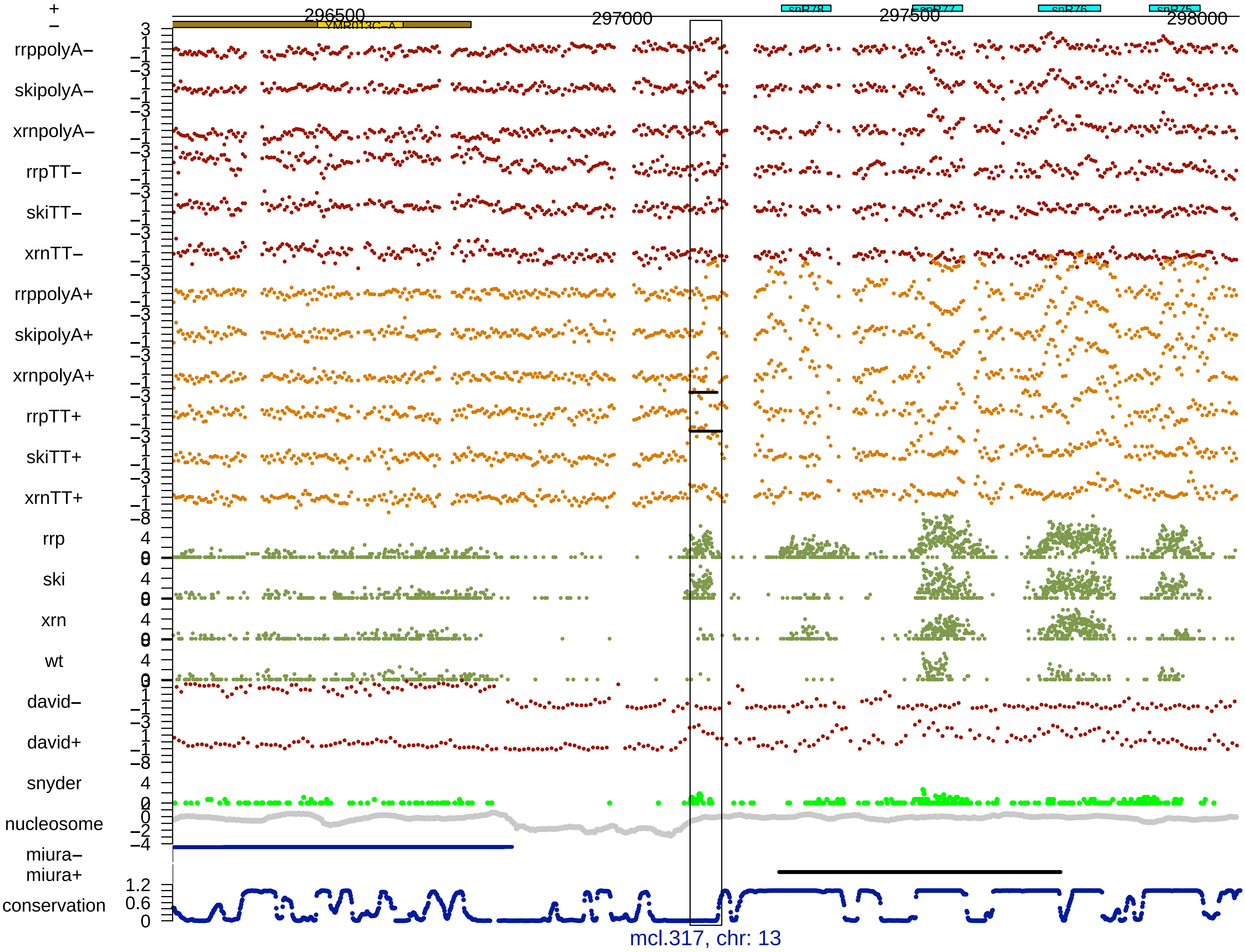

+
−
296500
297000
297500
298000
YMR013C-A
snR78
snR77
snR76
snR75
rrppolyA−
skipolyA−
xrnpolyA−
rrpTT−
skiTT−
xrnTT−
rrppolyA+
skipolyA+
xrnpolyA+
rrpTT+
skiTT+
xrnTT+
rrp
ski
xrn
wt
david−
david+
snyder
nucleosome
miura−
miura+
conservation
mcl.317, chr: 13

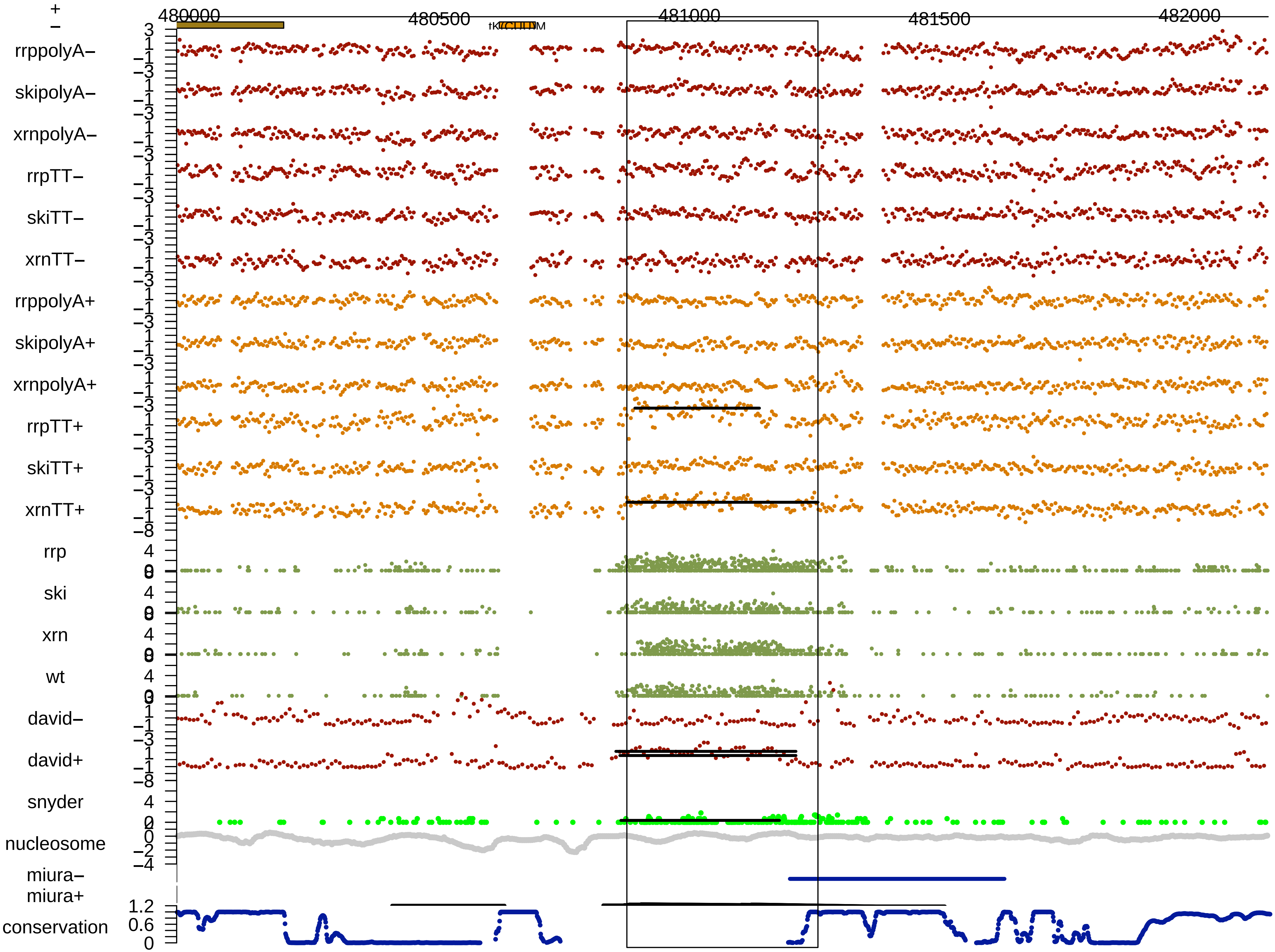

mcl.318, chr: 13

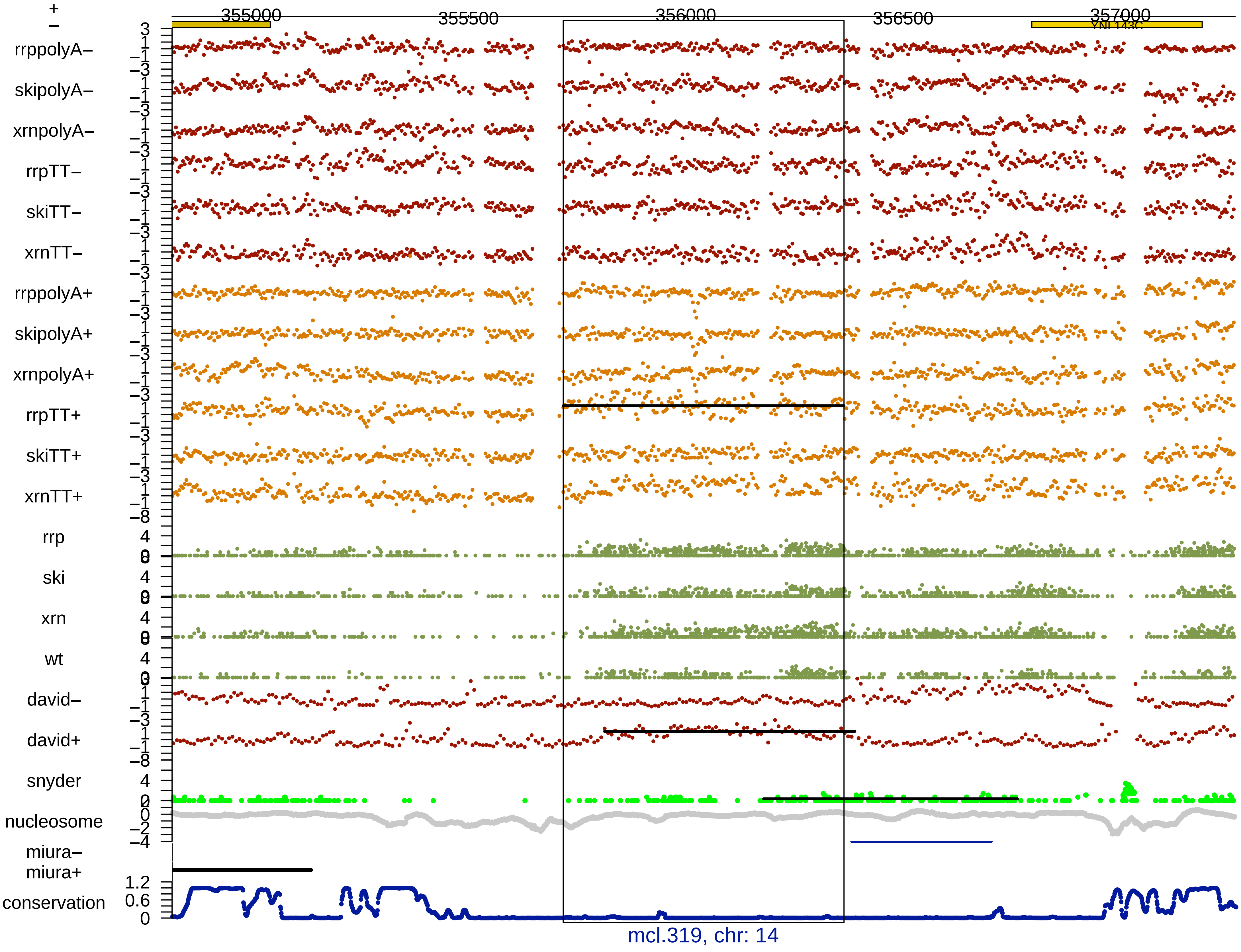
355000
355500
356000
356500
357000
YNL143C
+
–
rrppolyA–
skipolyA–
xrnpolyA–
rrpTT–
skiTT–
xrnTT–
rrppolyA+
skipolyA+
xrnpolyA+
rrpTT+
skiTT+
xrnTT+
rrp
ski
xrn
wt
david–
david+
snyder
nucleosome
miura–
miura+
conservation
mcl.319, chr: 14

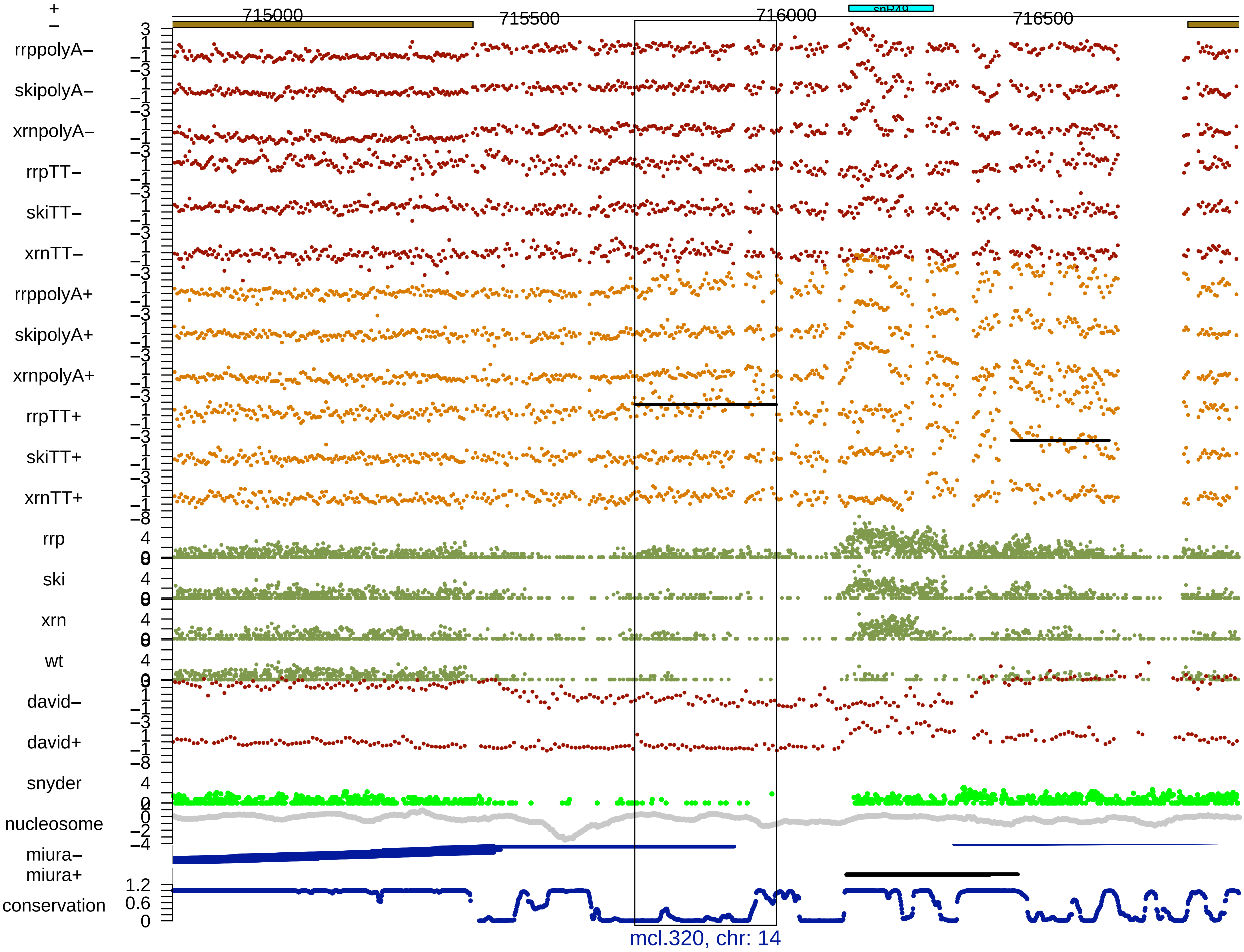

+
−
715000
715500
716000
716500
snR49
rrppolyA−
skipolyA−
xrnpolyA−
rrpTT−
skiTT−
xrnTT−
rrppolyA+
skipolyA+
xrnpolyA+
rrpTT+
skiTT+
xrnTT+
rrp
ski
xrn
wt
david−
david+
snyder
nucleosome
miura−
miura+
conservation
mcl.320, chr: 14

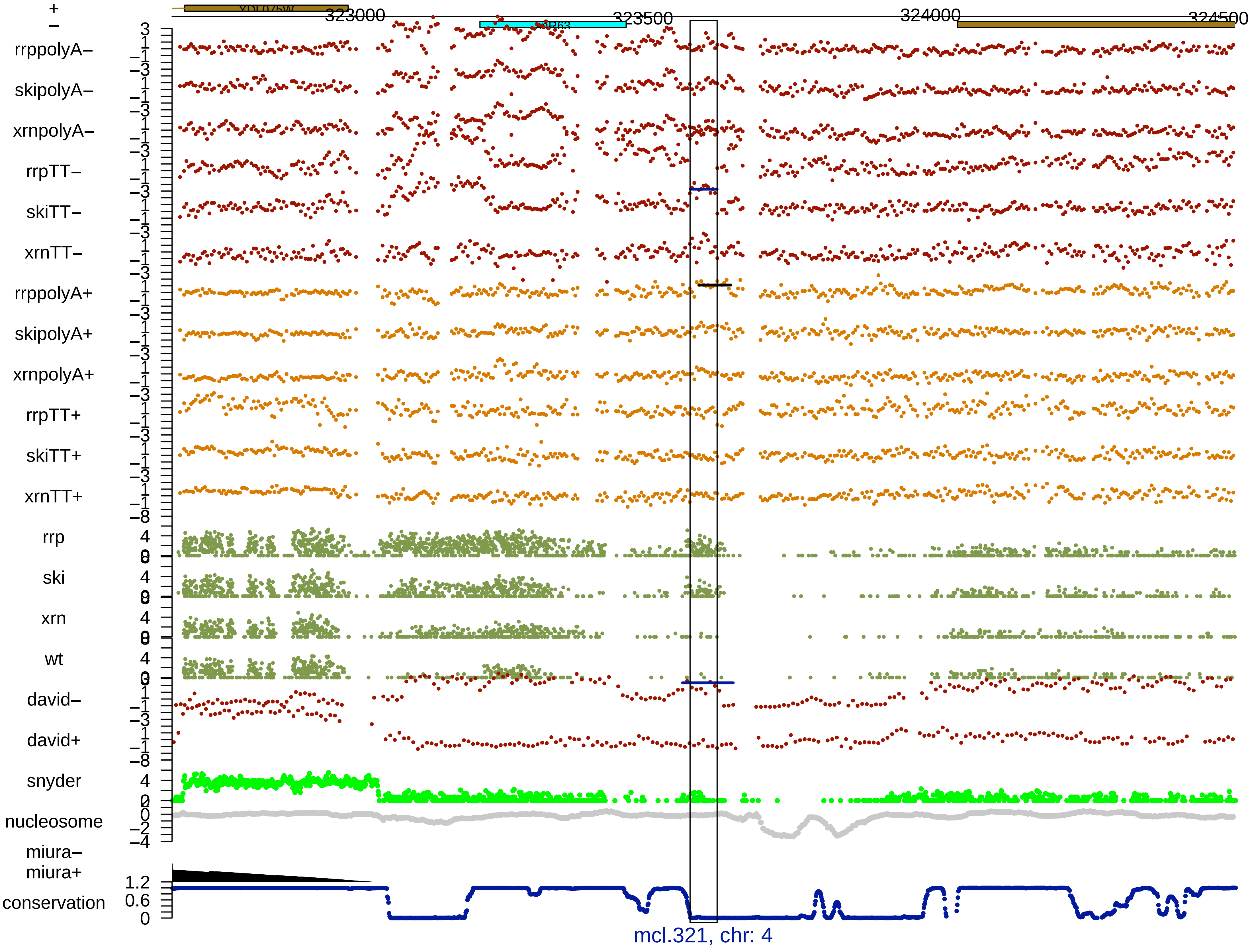

+
–
323000
323500
324000
324500
rrppolyA–
skipolyA–
xrnpolyA–
rrpTT–
skiTT–
xrnTT–
rrppolyA+
skipolyA+
xrnpolyA+
rrpTT+
skiTT+
xrnTT+
rrp
ski
xrn
wt
david–
david+
snyder
nucleosome
miura–
miura+
conservation
mcl.321, chr: 4

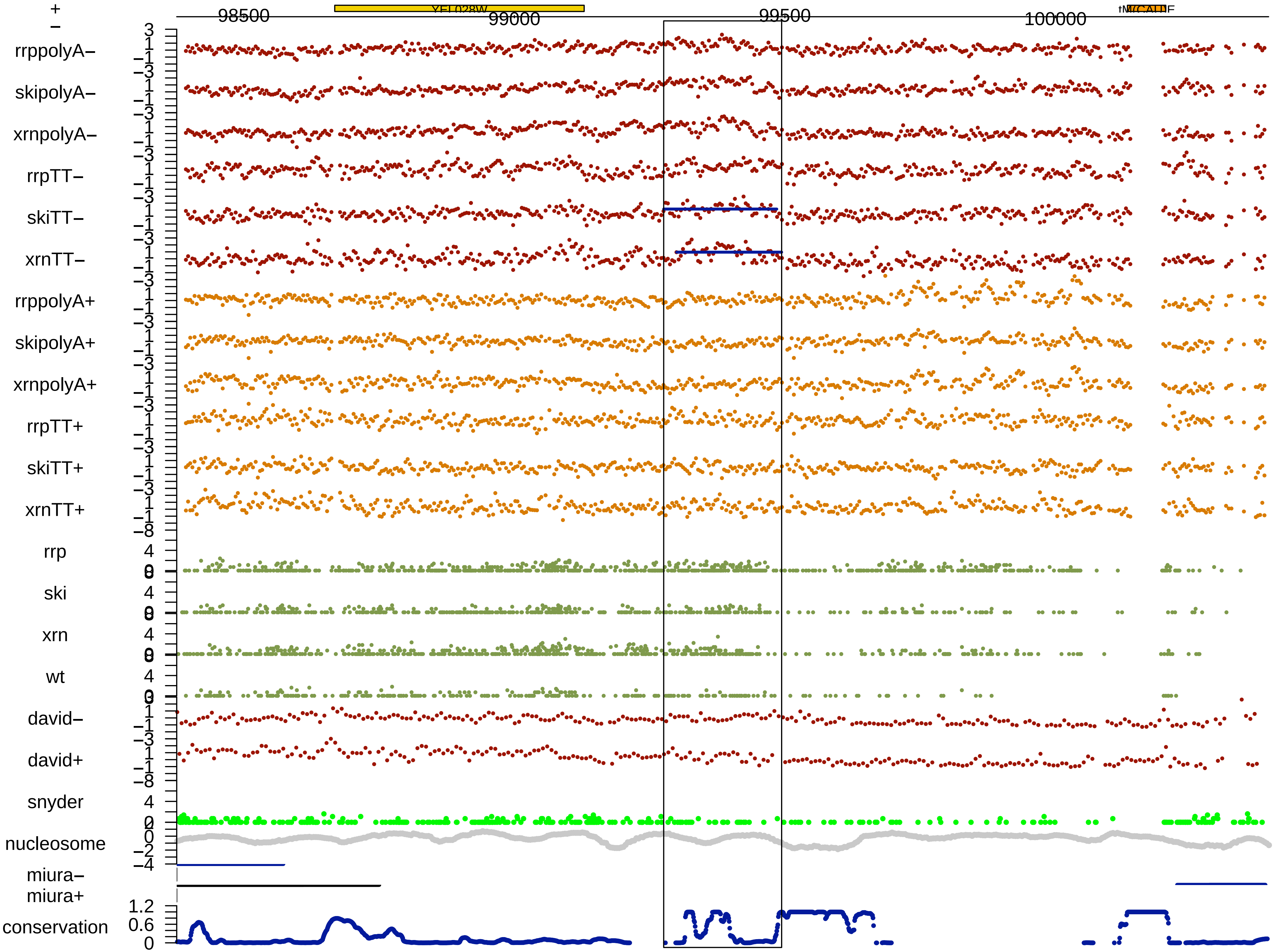
YEL028W
98500
99000
99500
100000
rrppolyA−
skipolyA−
xrnpolyA−
rrpTT−
skiTT−
xrnTT−
rrppolyA+
skipolyA+
xrnpolyA+
rrpTT+
skiTT+
xrnTT+
rrp
ski
xrn
wt
david−
david+
snyder
nucleosome
miura−
miura+
conservation

mcl.322, chr: 5

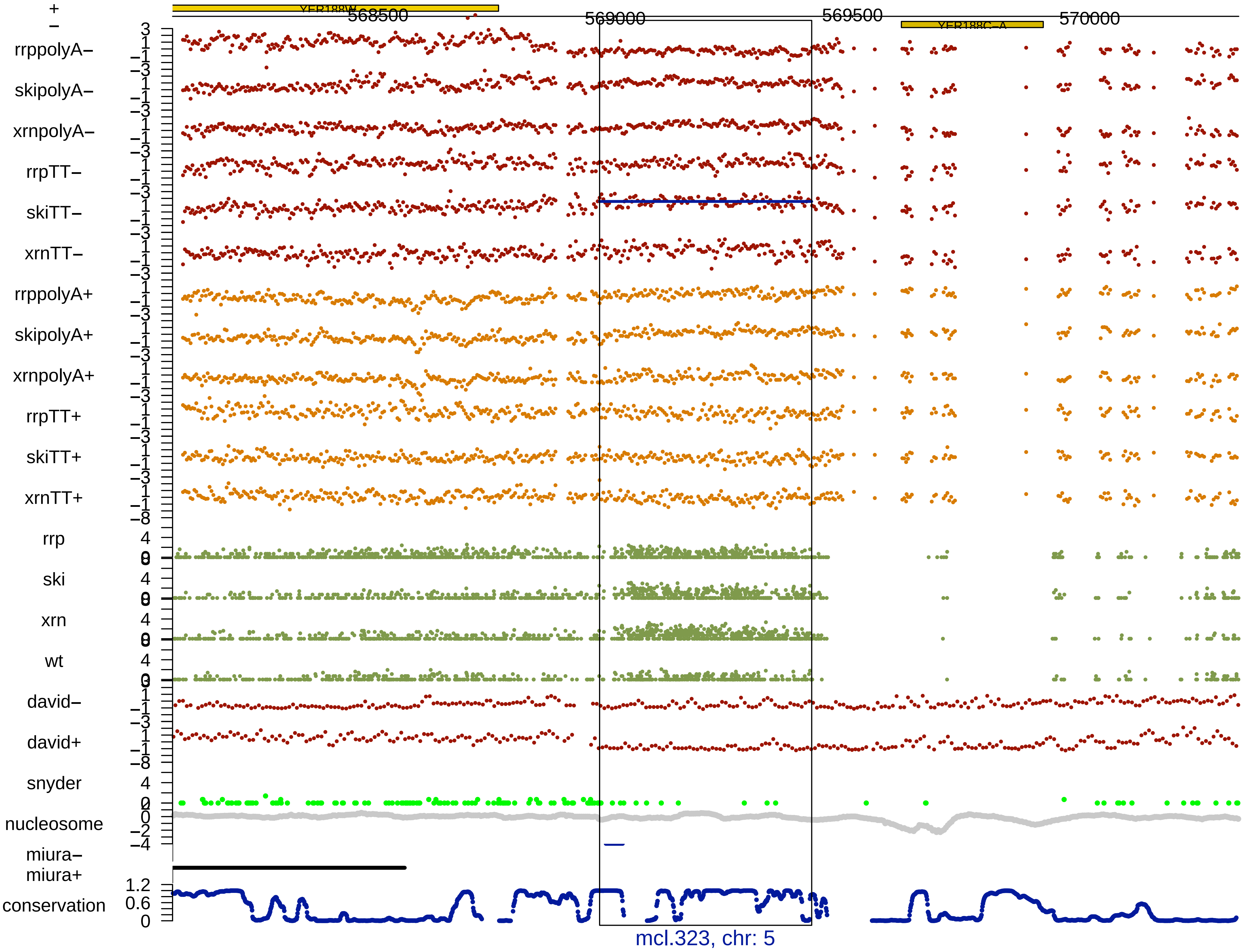

YER188W
568500
569000
569500
570000
YER188C-A
rrppolyA−
skipolyA−
xrnpolyA−
rrpTT−
skiTT−
xrnTT−
rrppolyA+
skipolyA+
xrnpolyA+
rrpTT+
skiTT+
xrnTT+
rrp
ski
xrn
wt
david−
david+
snyder
nucleosome
miura−
miura+
conservation
mcl.323, chr: 5

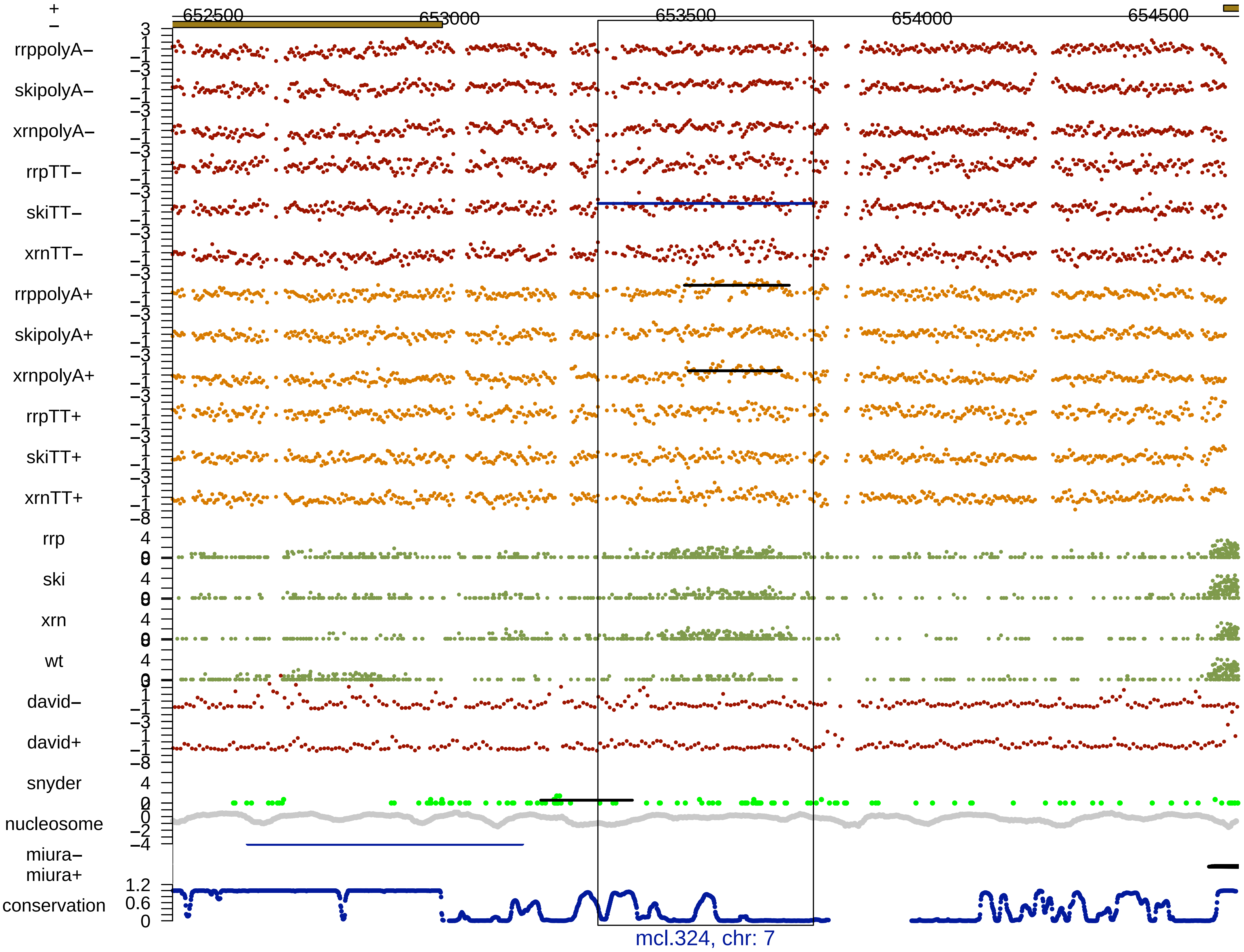

+
−
652500
653000
653500
654000
654500
rrppolyA−
skipolyA−
xrnpolyA−
rrpTT−
skiTT−
xrnTT−
rrppolyA+
skipolyA+
xrnpolyA+
rrpTT+
skiTT+
xrnTT+
rrp
ski
xrn
wt
david−
david+
snyder
nucleosome
miura−
miura+
conservation
mcl.324, chr: 7

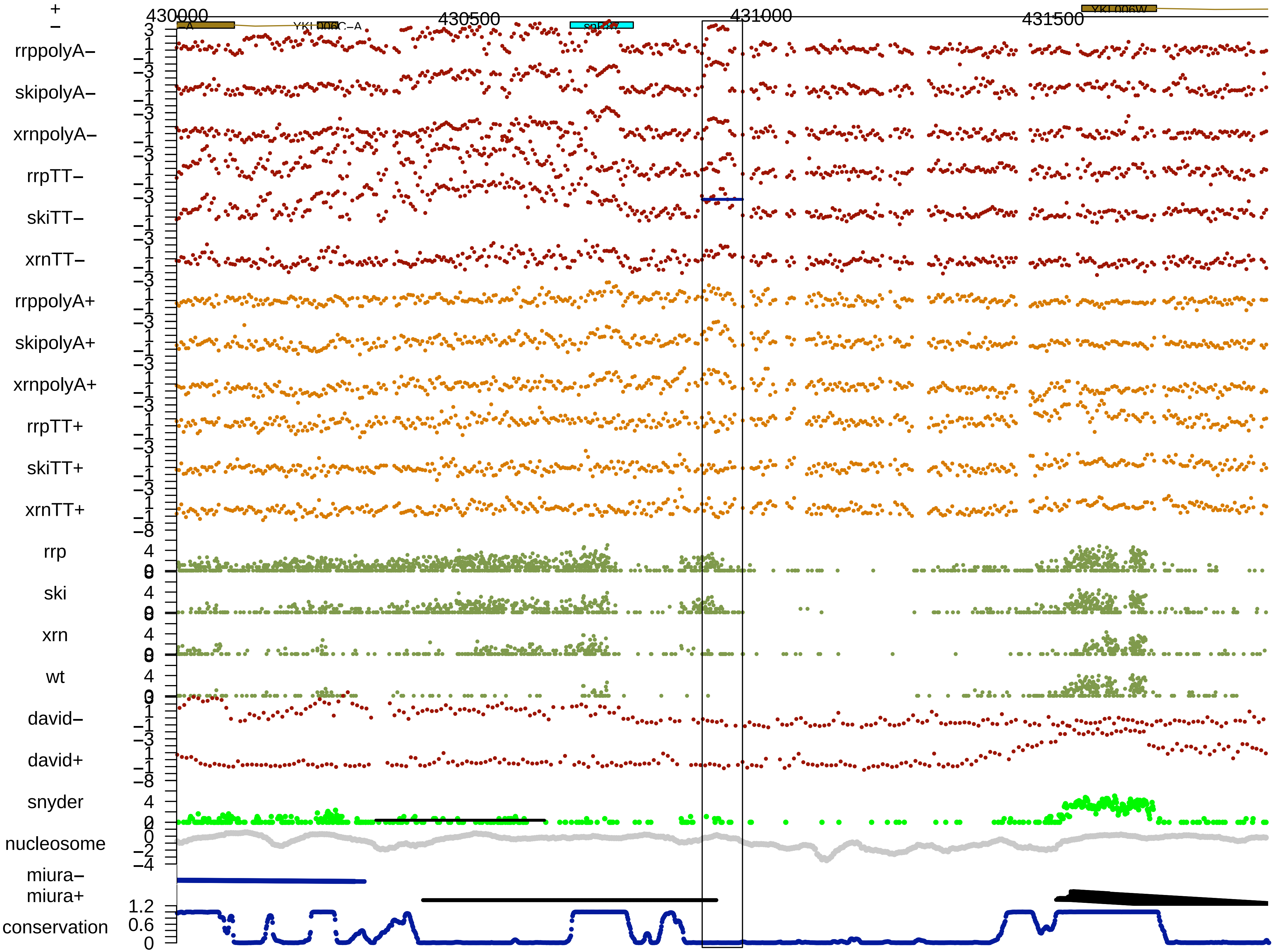

mcl.325, chr: 11

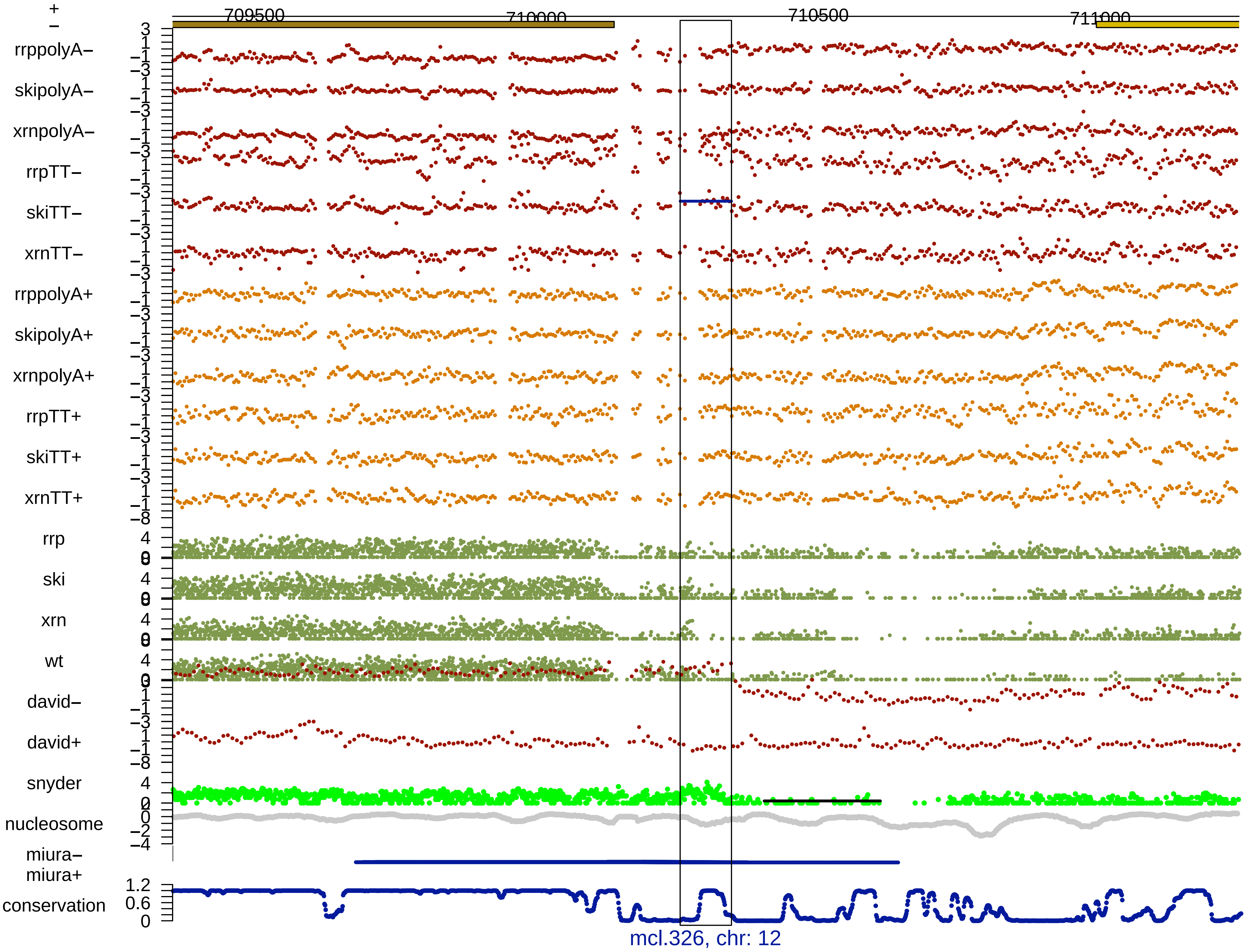

+
−
709500
710000
710500
711000
rrppolyA−
skipolyA−
xrnpolyA−
rrpTT−
skiTT−
xrnTT−
rrppolyA+
skipolyA+
xrnpolyA+
rrpTT+
skiTT+
xrnTT+
rrp
ski
xrn
wt
david−
david+
snyder
nucleosome
miura−
miura+
conservation
1.2
0.6
0
mcl.326, chr: 12

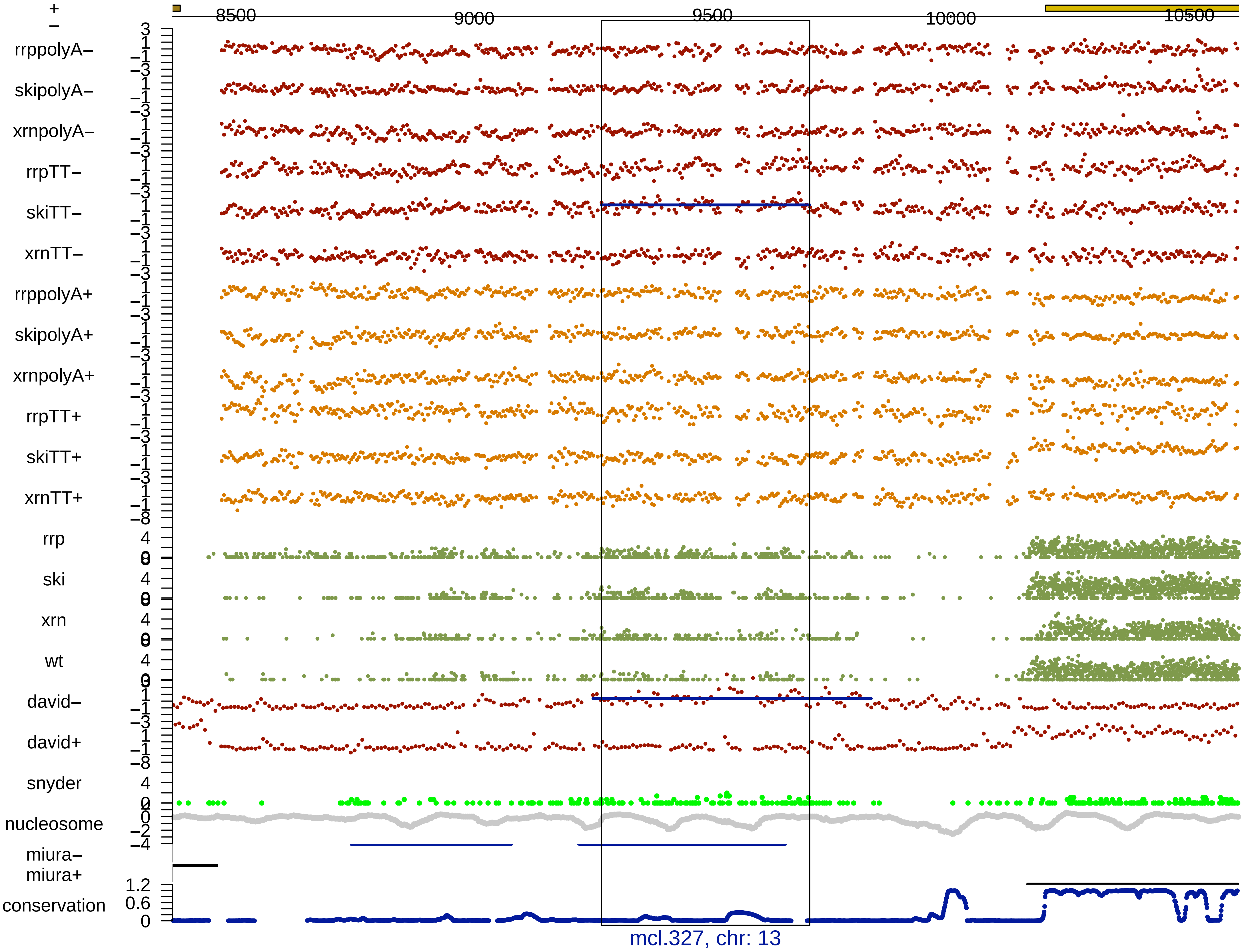

8500
9000
9500
10000
10500
rrppolyA−
skipolyA−
xrnpolyA−
rrpTT−
skiTT−
xrnTT−
rrppolyA+
skipolyA+
xrnpolyA+
rrpTT+
skiTT+
xrnTT+
rrp
ski
xrn
wt
david−
david+
snyder
nucleosome
miura−
miura+
conservation
mcl.327, chr: 13

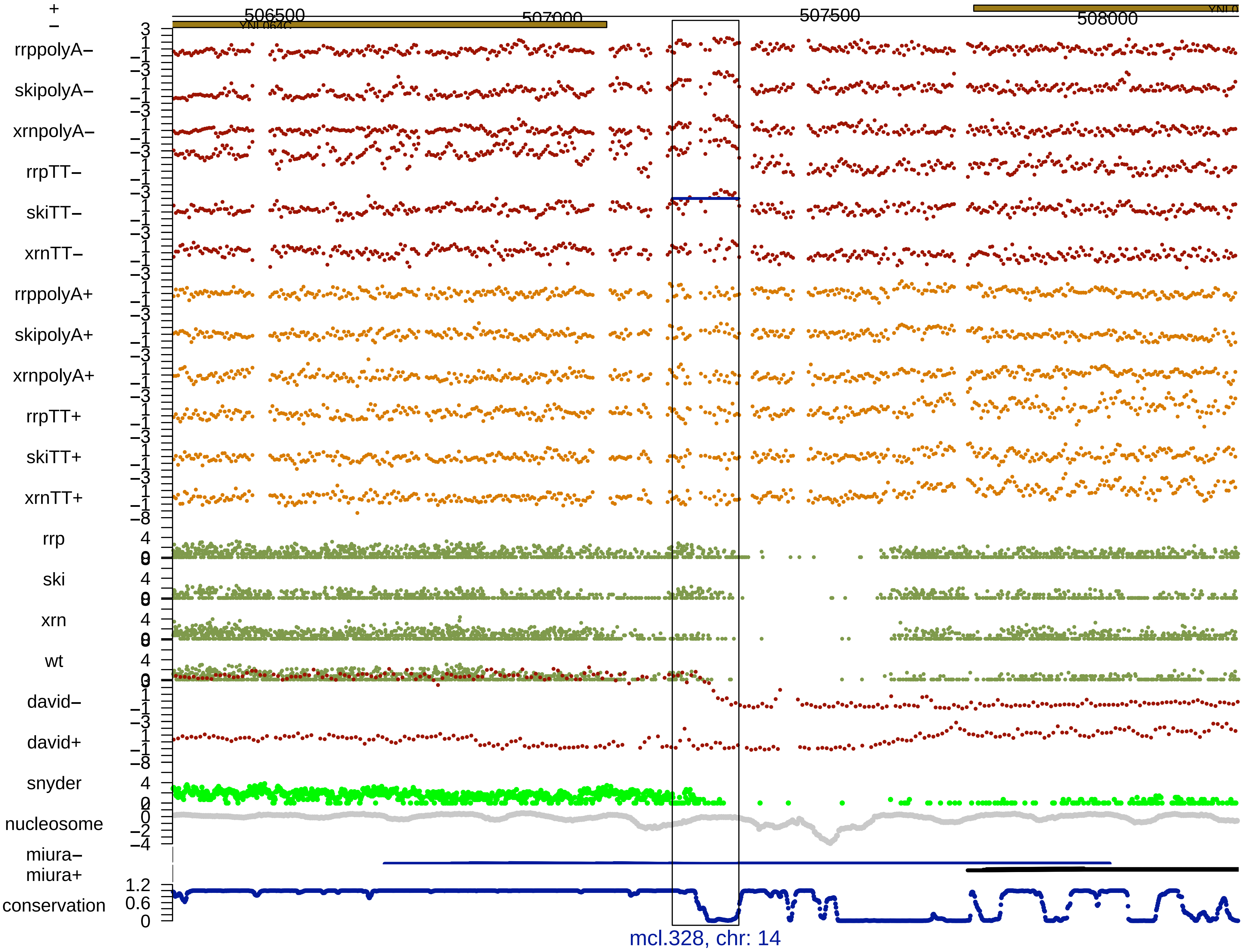

506500
507000
507500
508000
rrppolyA−
skipolyA−
xrnpolyA−
rrpTT−
skiTT−
xrnTT−
rrppolyA+
skipolyA+
xrnpolyA+
rrpTT+
skiTT+
xrnTT+
rrp
ski
xrn
wt
david−
david+
snyder
nucleosome
miura−
miura+
conservation
mcl.328, chr: 14

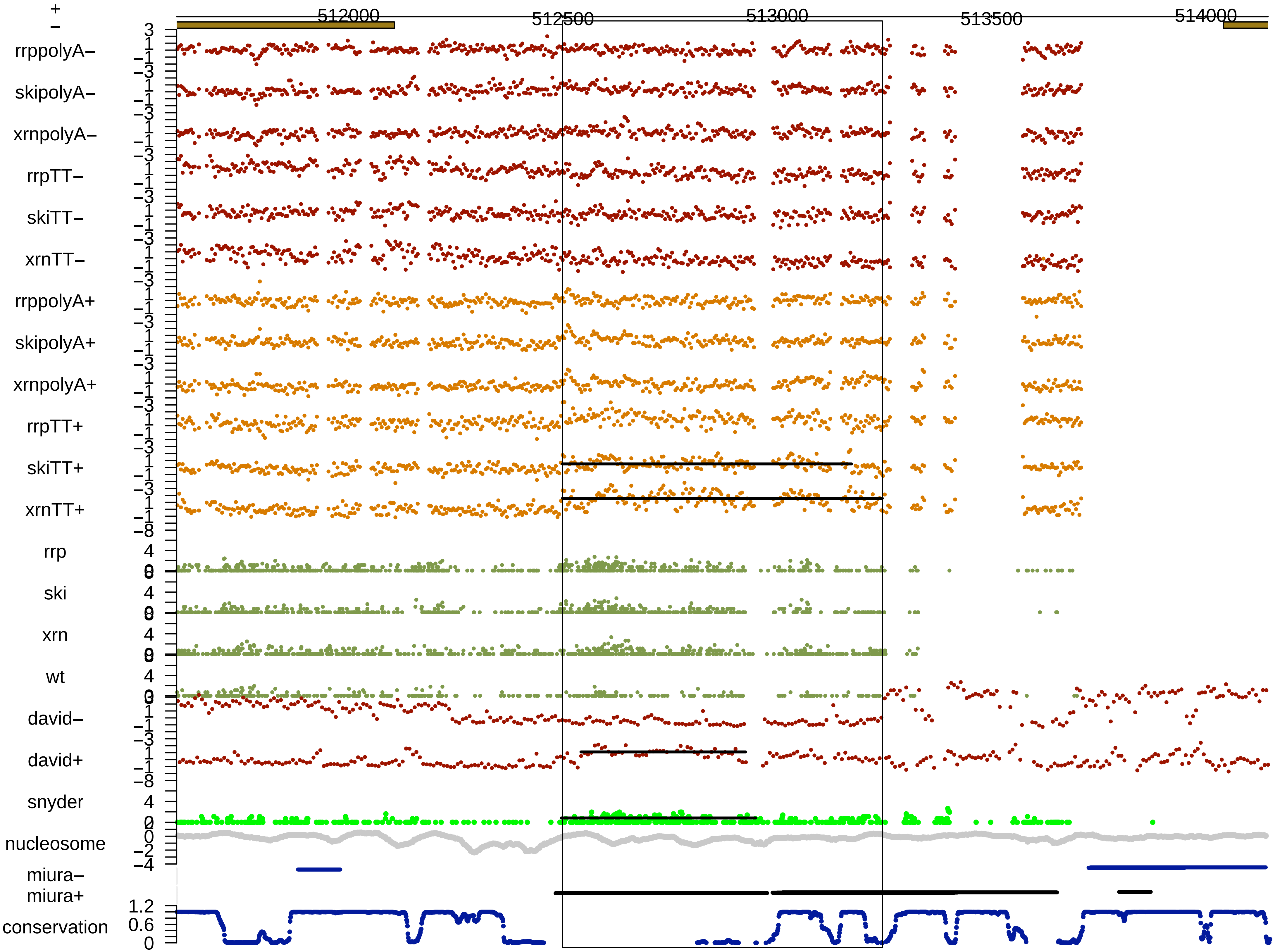
+
–
512000
512500
513000
513500
514000
rrppolyA–
skipolyA–
xrnpolyA–
rrpTT–
skiTT–
xrnTT–
rrppolyA+
skipolyA+
xrnpolyA+
rrpTT+
skiTT+
xrnTT+
rrp
ski
xrn
wt
david–
david+
snyder
nucleosome
miura–
miura+
conservation
3
1
–1
–3
–8
4
0
2
–2
–4
1.2
0.6
0

mcl.329, chr: 4

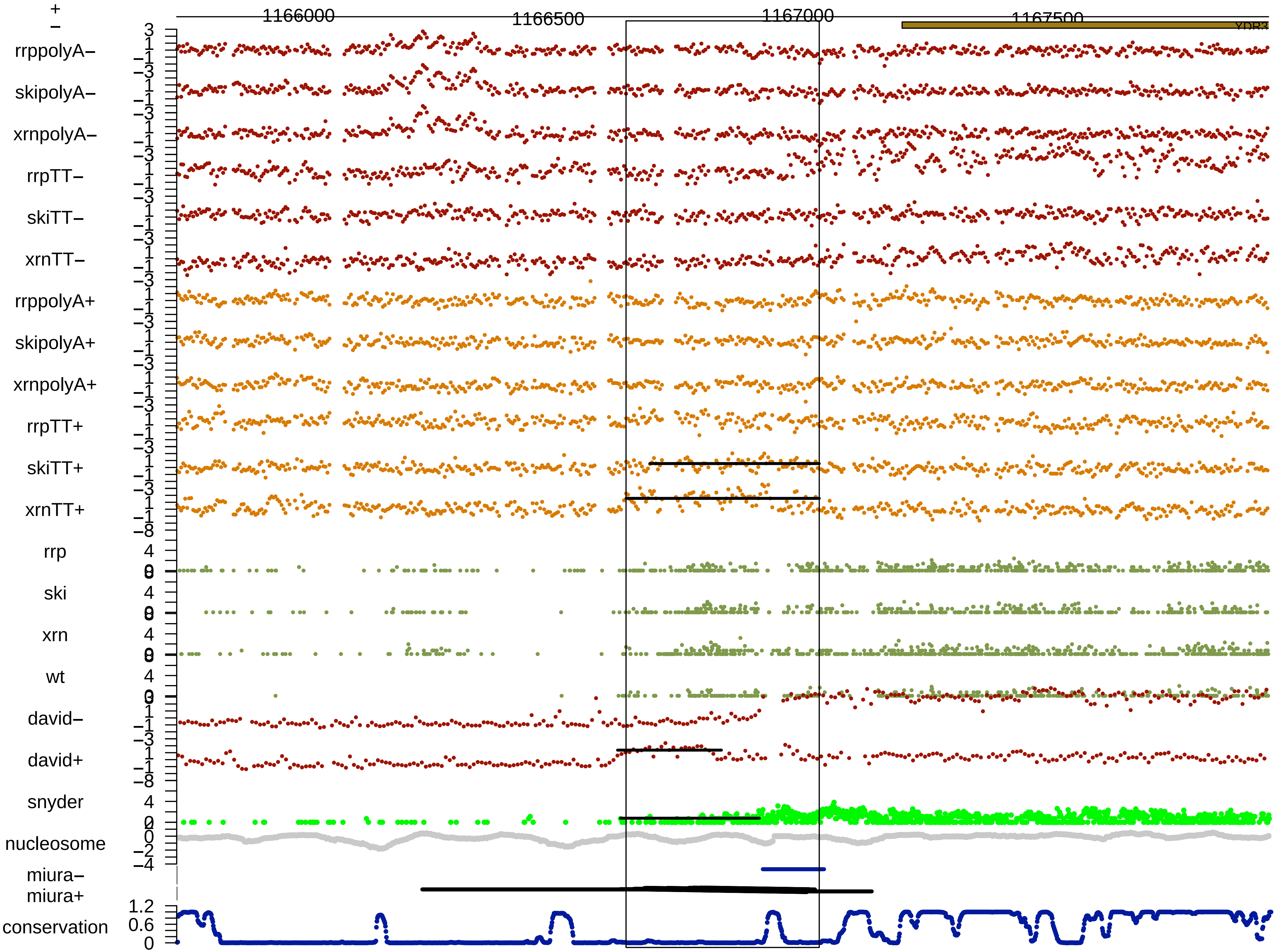
+
−
1166000
1166500
1167000
1167500
rrppolyA−
skipolyA−
xrnpolyA−
rrpTT−
skiTT−
xrnTT−
rrppolyA+
skipolyA+
xrnpolyA+
rrpTT+
skiTT+
xrnTT+
rrp
ski
xrn
wt
david−
david+
snyder
nucleosome
miura−
miura+
conservation

mcl.330, chr: 4

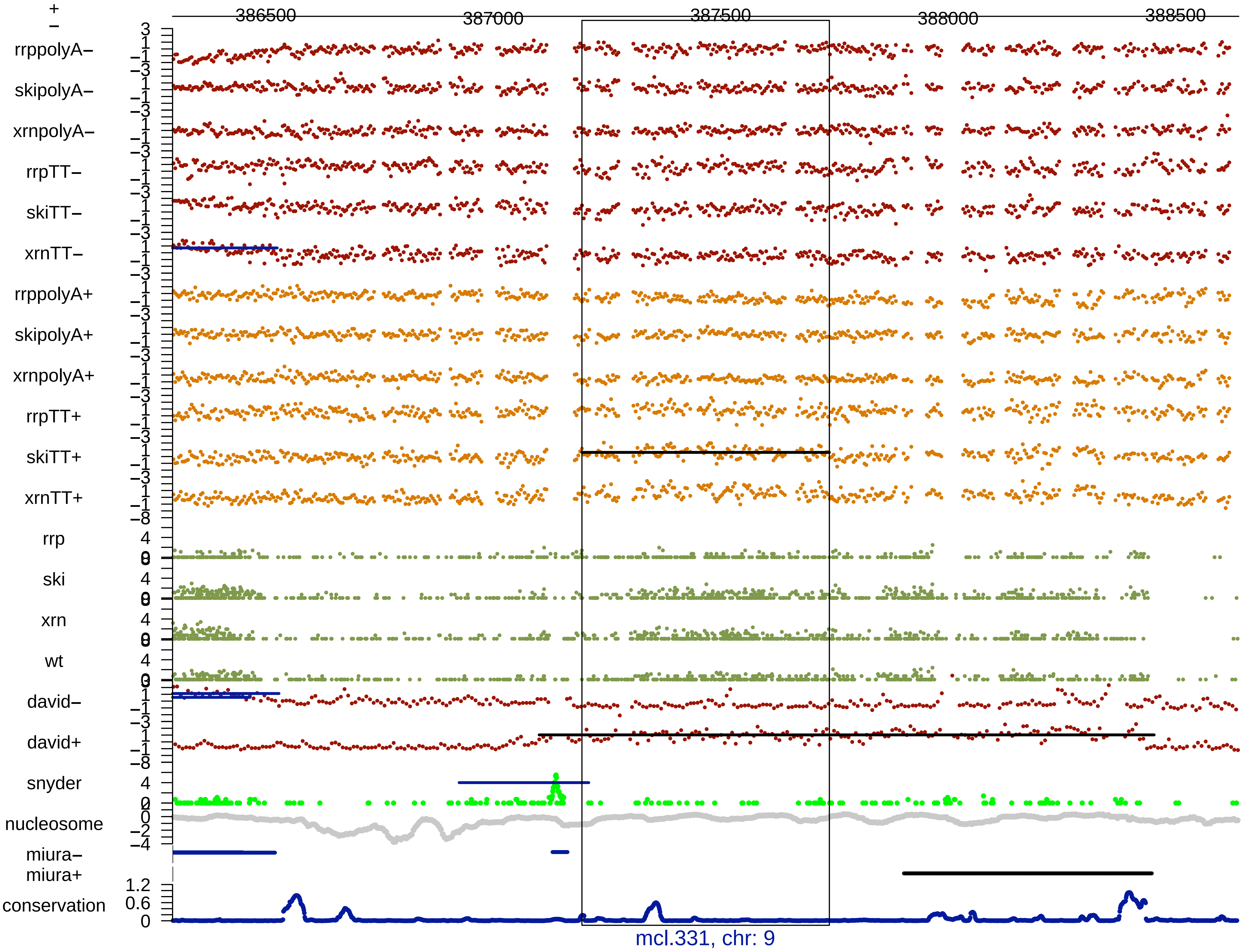
+
−
386500
387000
387500
388000
388500
rrppolyA−
skipolyA−
xrnpolyA−
rrpTT−
skiTT−
xrnTT−
rrppolyA+
skipolyA+
xrnpolyA+
rrpTT+
skiTT+
xrnTT+
rrp
ski
xrn
wt
david−
david+
snyder
nucleosome
miura−
miura+
conservation
mcl.331, chr: 9

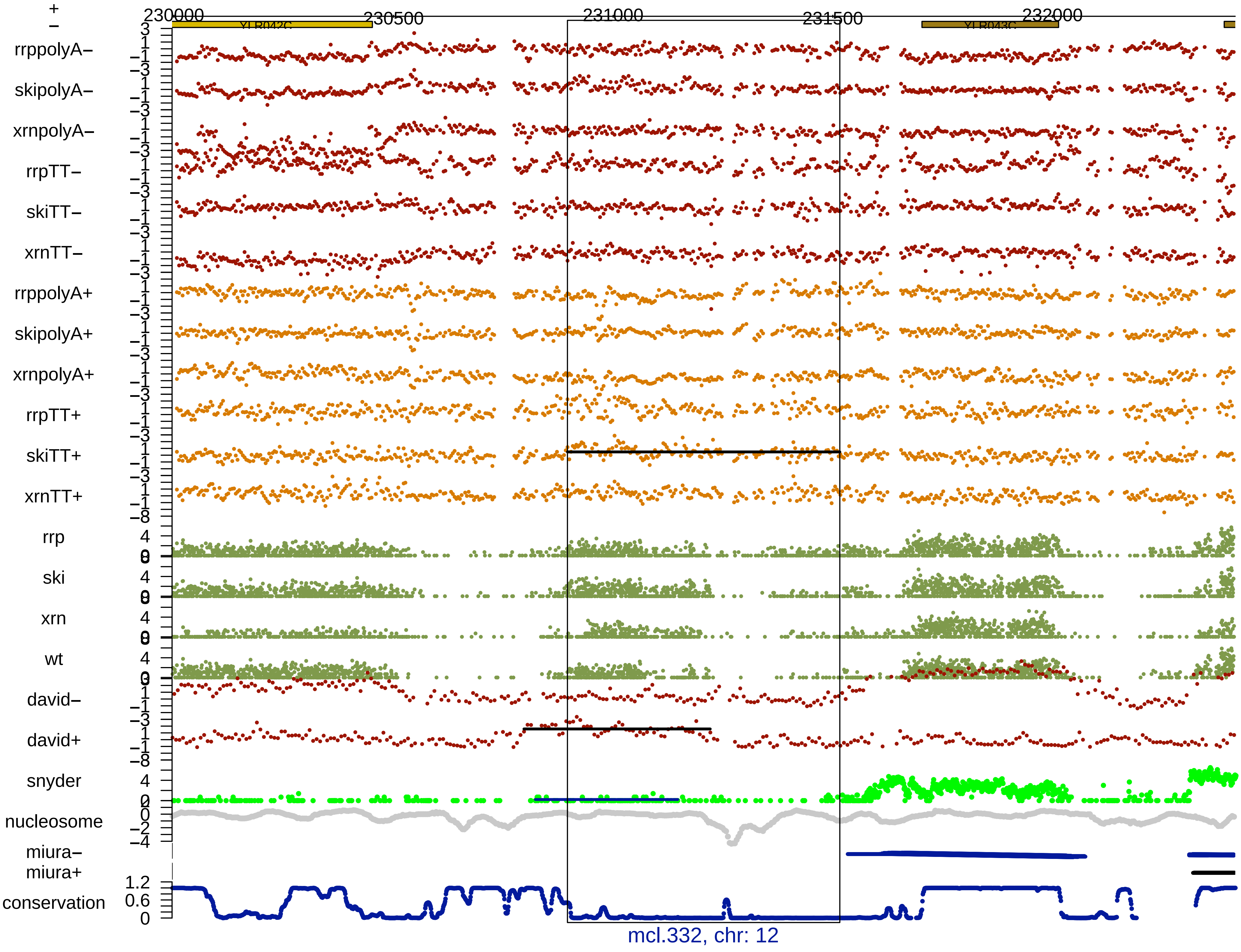
230000
230500
231000
231500
232000
rrppolyA−
skipolyA−
xrnpolyA−
rrpTT−
skiTT−
xrnTT−
rrppolyA+
skipolyA+
xrnpolyA+
rrpTT+
skiTT+
xrnTT+
rrp
ski
xrn
wt
david−
david+
snyder
nucleosome
miura−
miura+
conservation
mcl.332, chr: 12

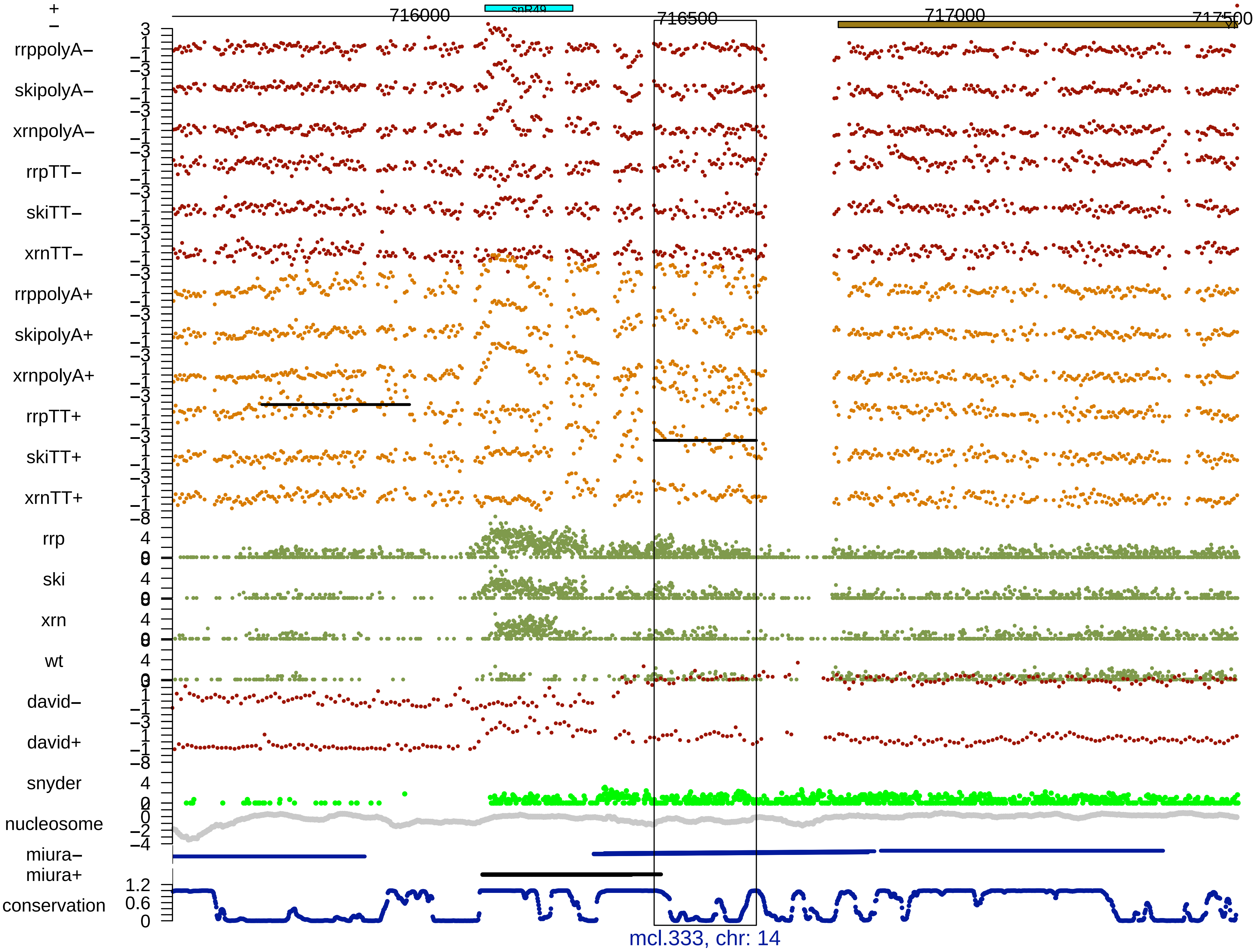

+
−
716000
snR49
716500
717000
717500
rrppolyA−
skipolyA−
xrnpolyA−
rrpTT−
skiTT−
xrnTT−
rrppolyA+
skipolyA+
xrnpolyA+
rrpTT+
skiTT+
xrnTT+
rrp
ski
xrn
wt
david−
david+
snyder
nucleosome
miura−
miura+
conservation
mcl.333, chr: 14

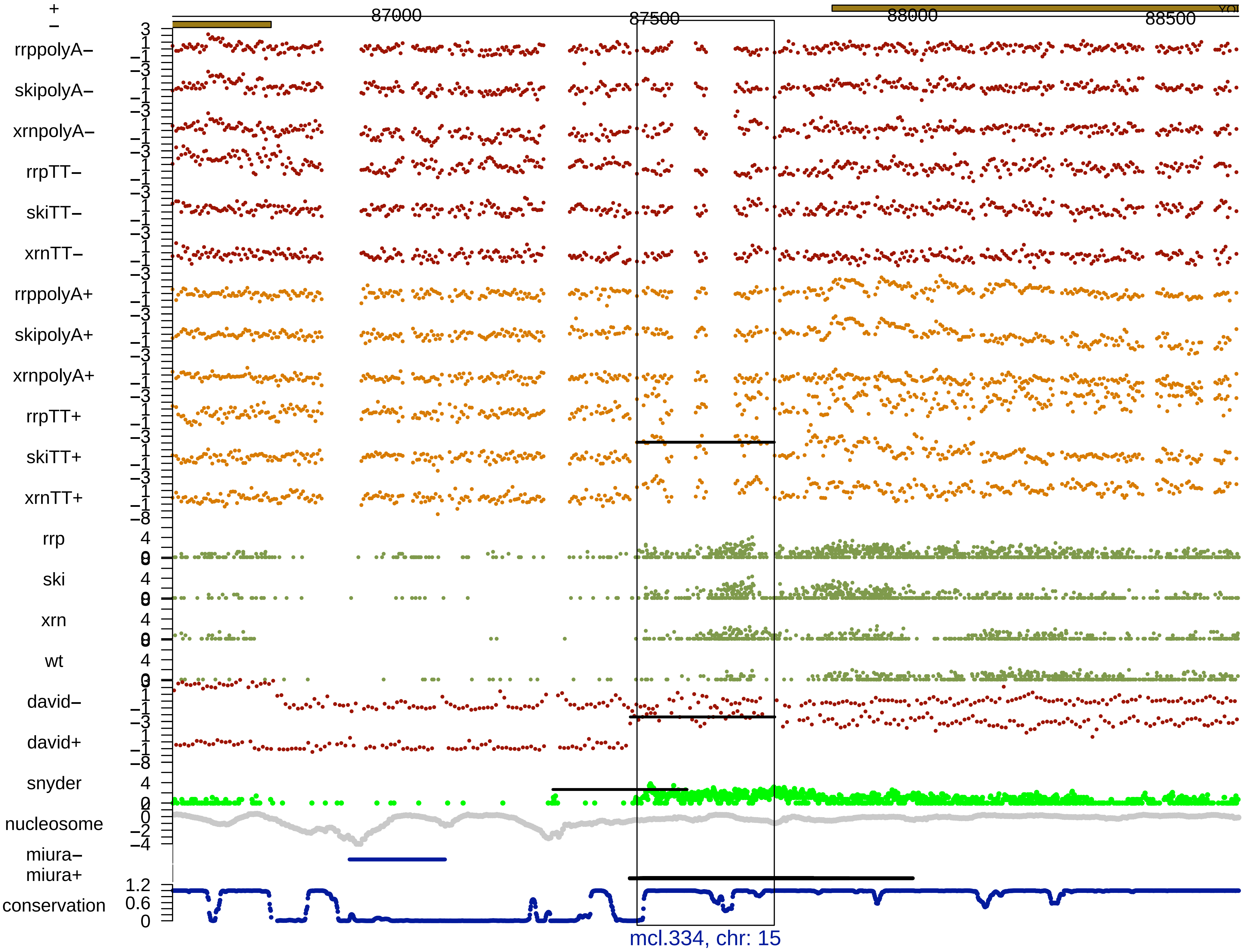
+
−
87000
87500
88000
88500
YOL
rrppolyA−
skipolyA−
xrnpolyA−
rrpTT−
skiTT−
xrnTT−
rrppolyA+
skipolyA+
xrnpolyA+
rrpTT+
skiTT+
xrnTT+
rrp
ski
xrn
wt
david−
david+
snyder
nucleosome
miura−
miura+
conservation
mcl.334, chr: 15

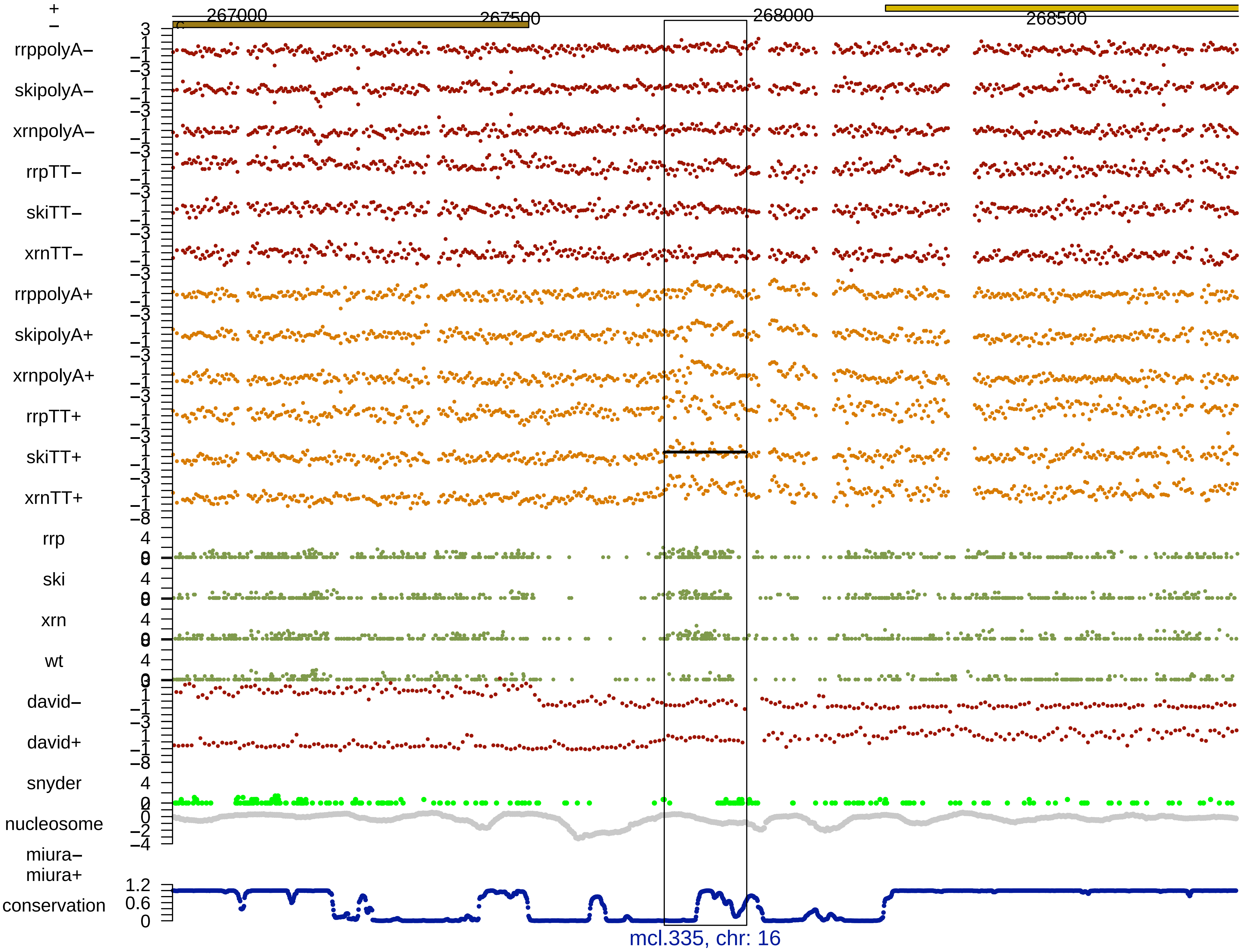

267000
267500
268000
268500
rrppolyA–
skipolyA–
xrnpolyA–
rrpTT–
skiTT–
xrnTT–
rrppolyA+
skipolyA+
xrnpolyA+
rrpTT+
skiTT+
xrnTT+
rrp
ski
xrn
wt
david–
david+
snyder
nucleosome
miura–
miura+
conservation
mcl.335, chr: 16

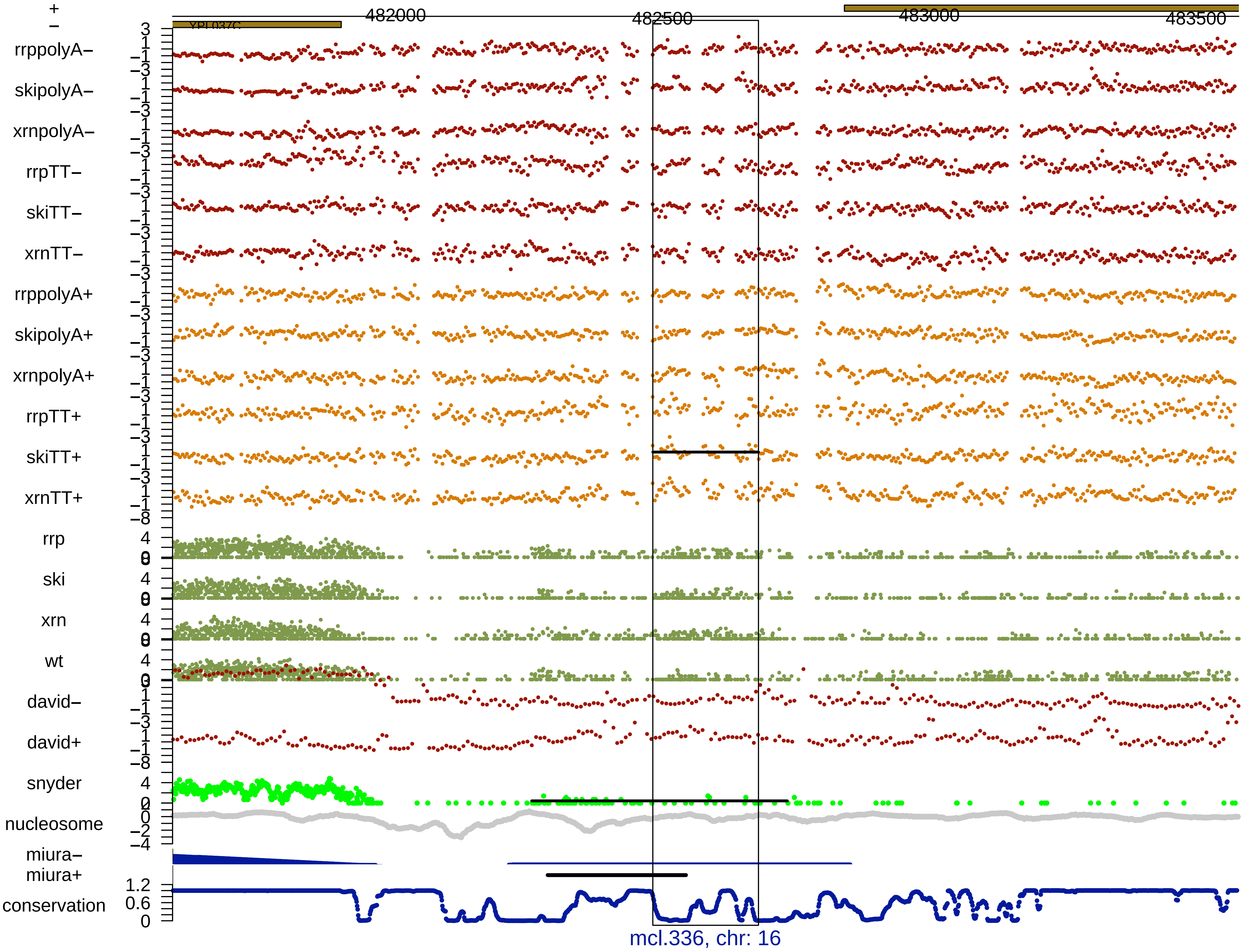

+
−
482000
482500
483000
483500
YPL037C
rrppolyA−
skipolyA−
xrnpolyA−
rrpTT−
skiTT−
xrnTT−
rrppolyA+
skipolyA+
xrnpolyA+
rrpTT+
skiTT+
xrnTT+
rrp
ski
xrn
wt
david−
david+
snyder
nucleosome
miura−
miura+
conservation
mcl.336, chr: 16

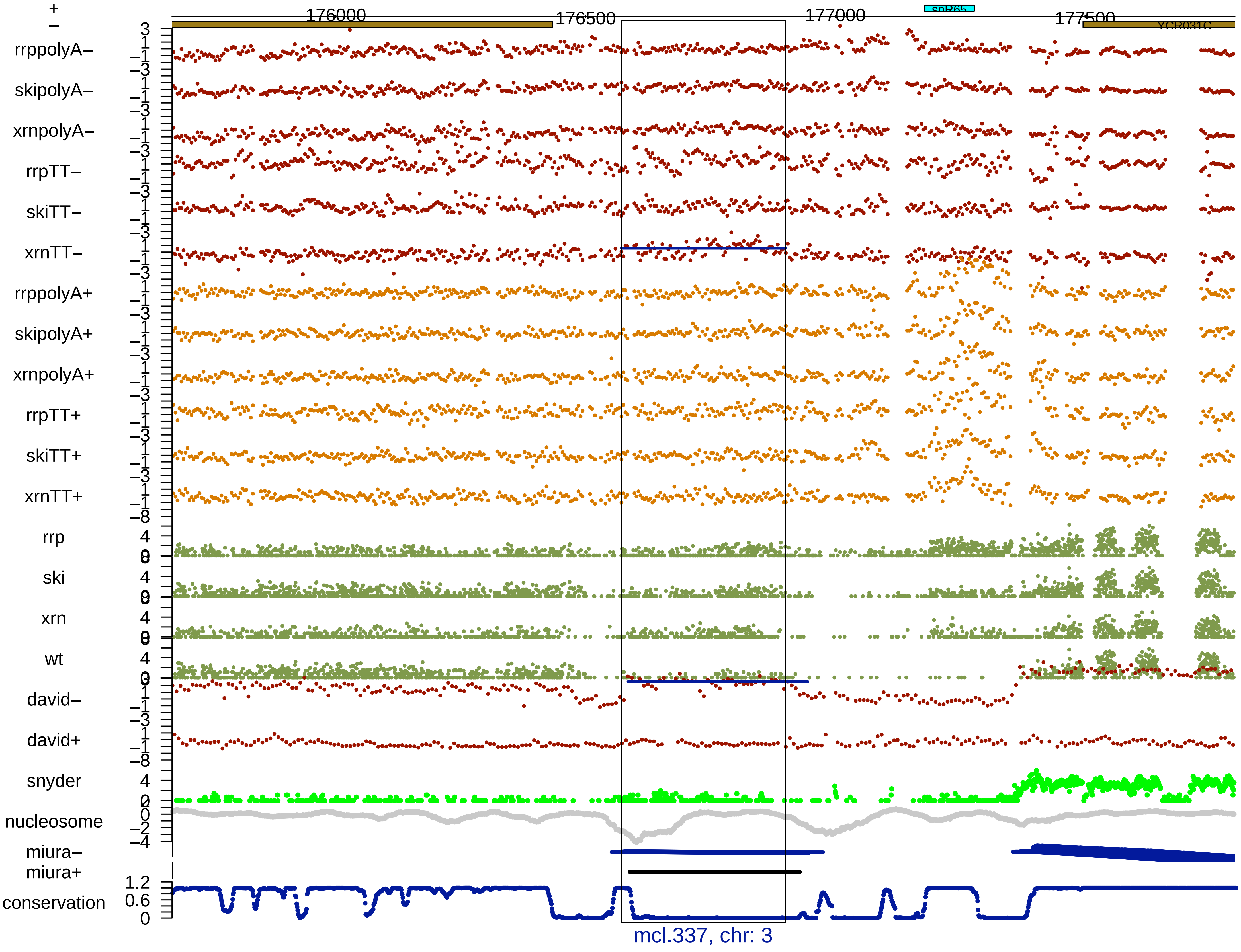

+
−
176000
176500
177000
177500
snR65
YCR031C
rrppolyA−
skipolyA−
xrnpolyA−
rrpTT−
skiTT−
xrnTT−
rrppolyA+
skipolyA+
xrnpolyA+
rrpTT+
skiTT+
xrnTT+
rrp
ski
xrn
wt
david−
david+
snyder
nucleosome
miura−
miura+
conservation
mcl.337, chr: 3

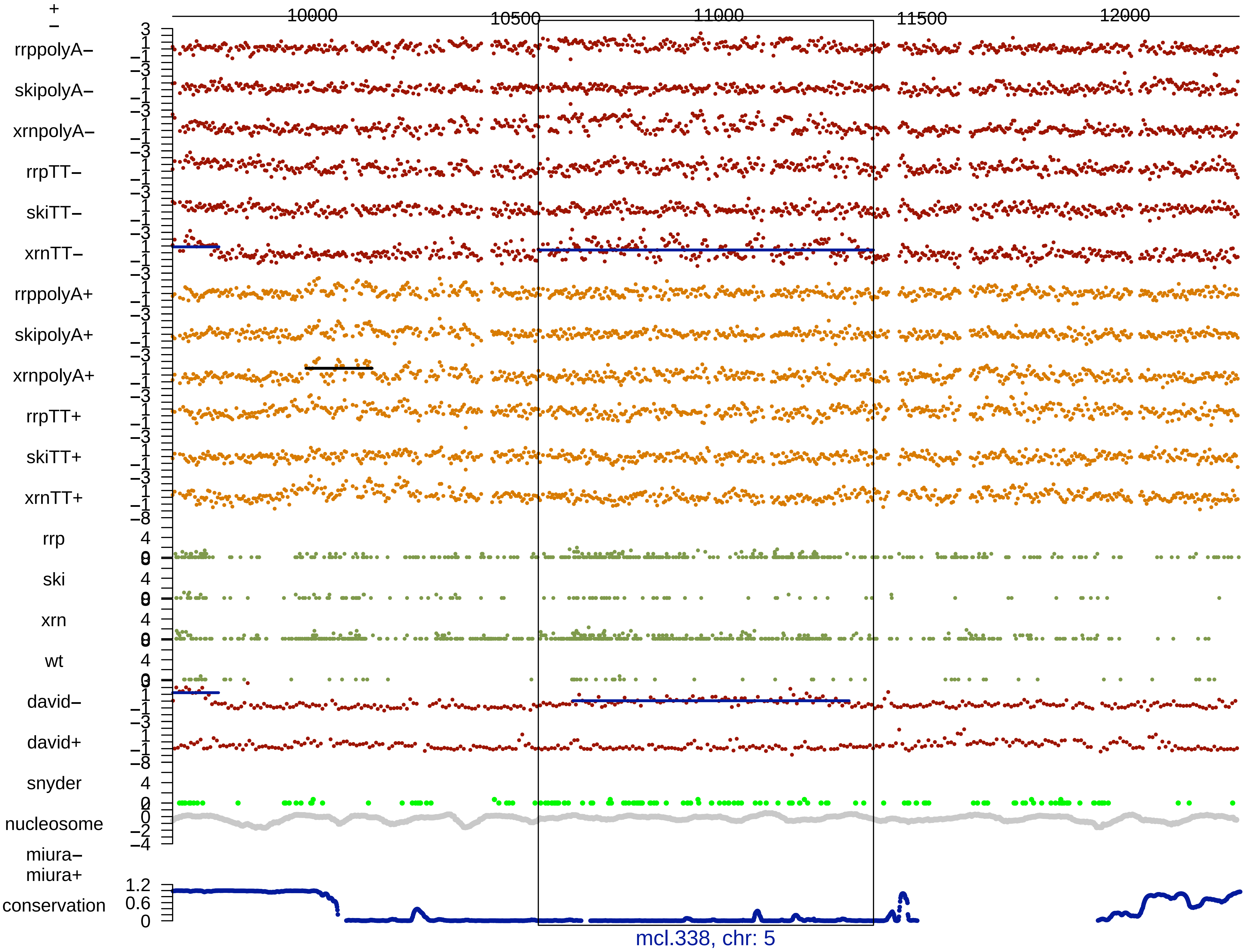

10000
10500
11000
11500
12000
rrppolyA−
skipolyA−
xrnpolyA−
rrpTT−
skiTT−
xrnTT−
rrppolyA+
skipolyA+
xrnpolyA+
rrpTT+
skiTT+
xrnTT+
rrp
ski
xrn
wt
david−
david+
snyder
nucleosome
miura−
miura+
conservation
mcl.338, chr: 5

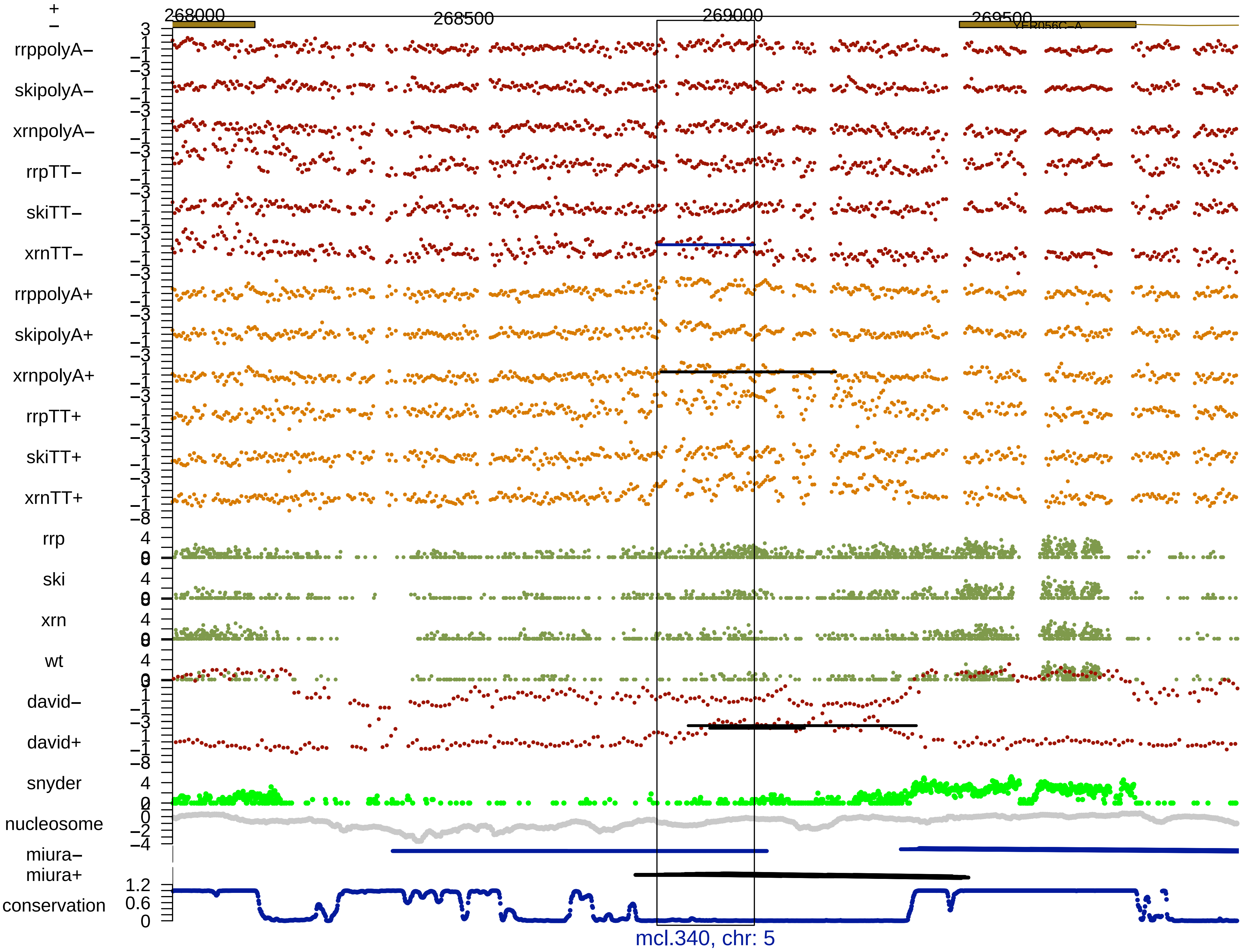

268000
268500
269000
269500
rrppolyA−
skipolyA−
xrnpolyA−
rrpTT−
skiTT−
xrnTT−
rrppolyA+
skipolyA+
xrnpolyA+
rrpTT+
skiTT+
xrnTT+
rrp
ski
xrn
wt
david−
david+
snyder
nucleosome
miura−
miura+
conservation
mcl.340, chr: 5

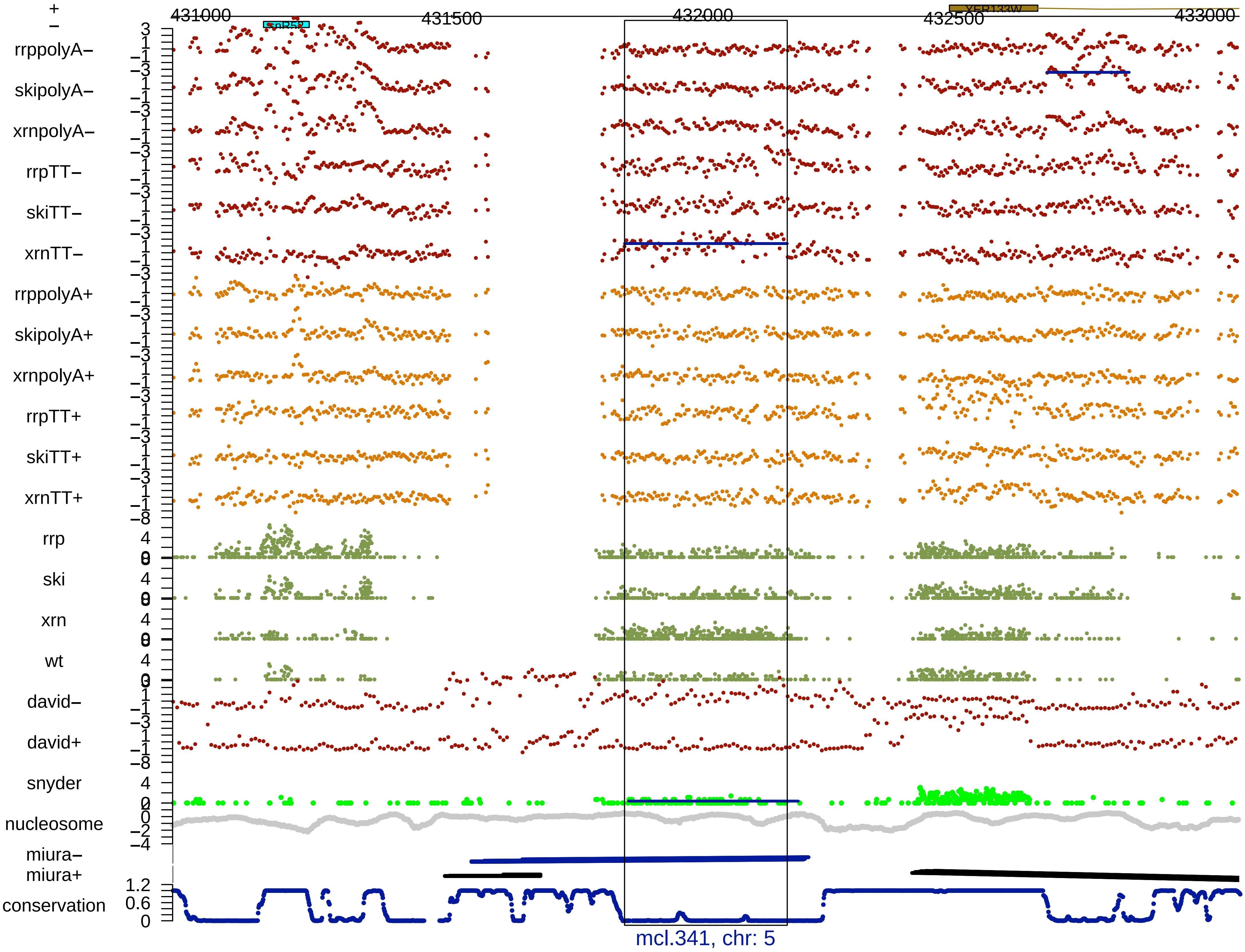

431000
431500
432000
432500
433000
YER133W
rrppolyA−
skipolyA−
xrnpolyA−
rrpTT−
skiTT−
xrnTT−
rrppolyA+
skipolyA+
xrnpolyA+
rrpTT+
skiTT+
xrnTT+
rrp
ski
xrn
wt
david−
david+
snyder
nucleosome
miura−
miura+
conservation
mcl.341, chr: 5

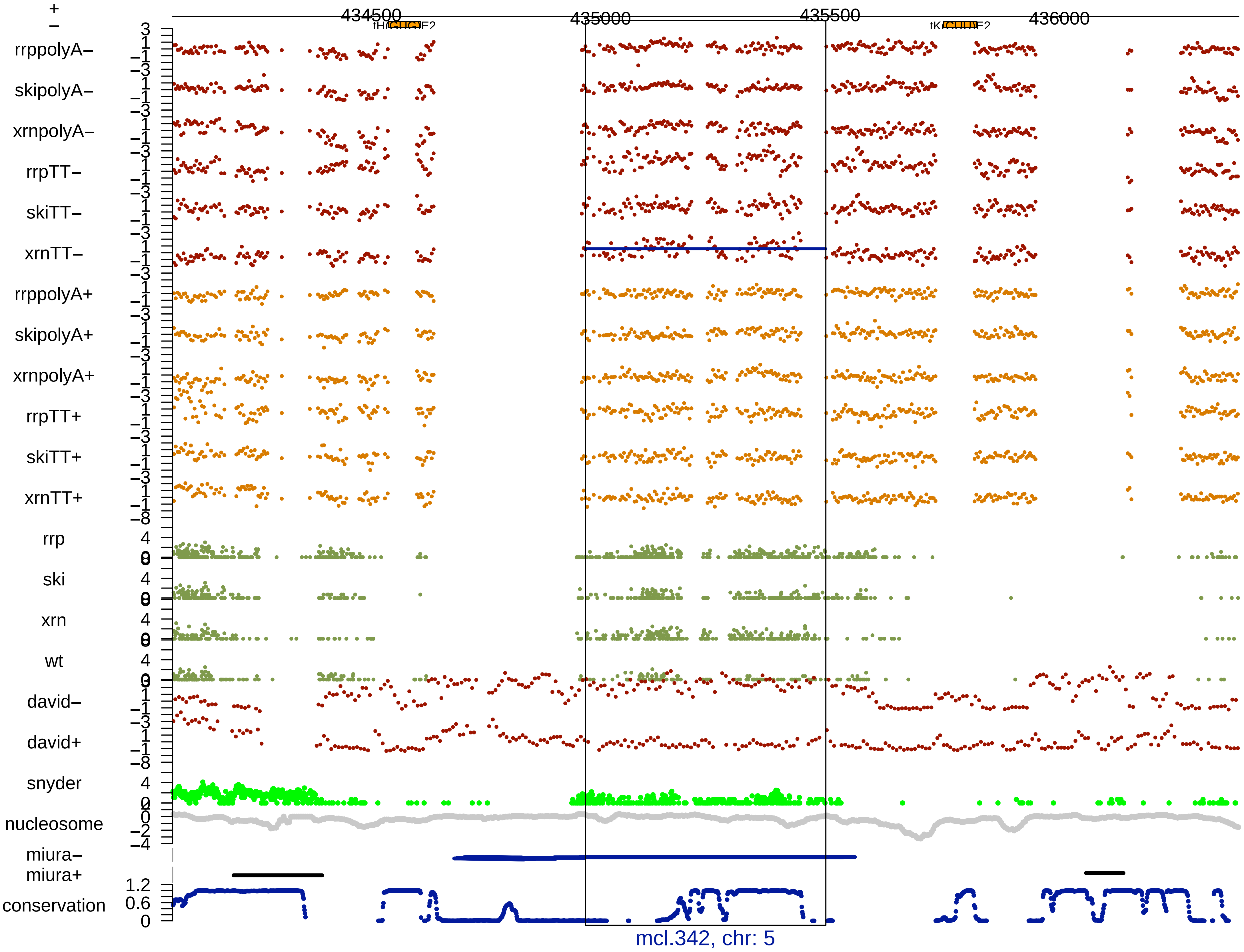
+
−
434500
435000
435500
436000
rrppolyA−
skipolyA−
xrnpolyA−
rrpTT−
skiTT−
xrnTT−
rrppolyA+
skipolyA+
xrnpolyA+
rrpTT+
skiTT+
xrnTT+
rrp
ski
xrn
wt
david−
david+
snyder
nucleosome
miura−
miura+
conservation
mcl.342, chr: 5

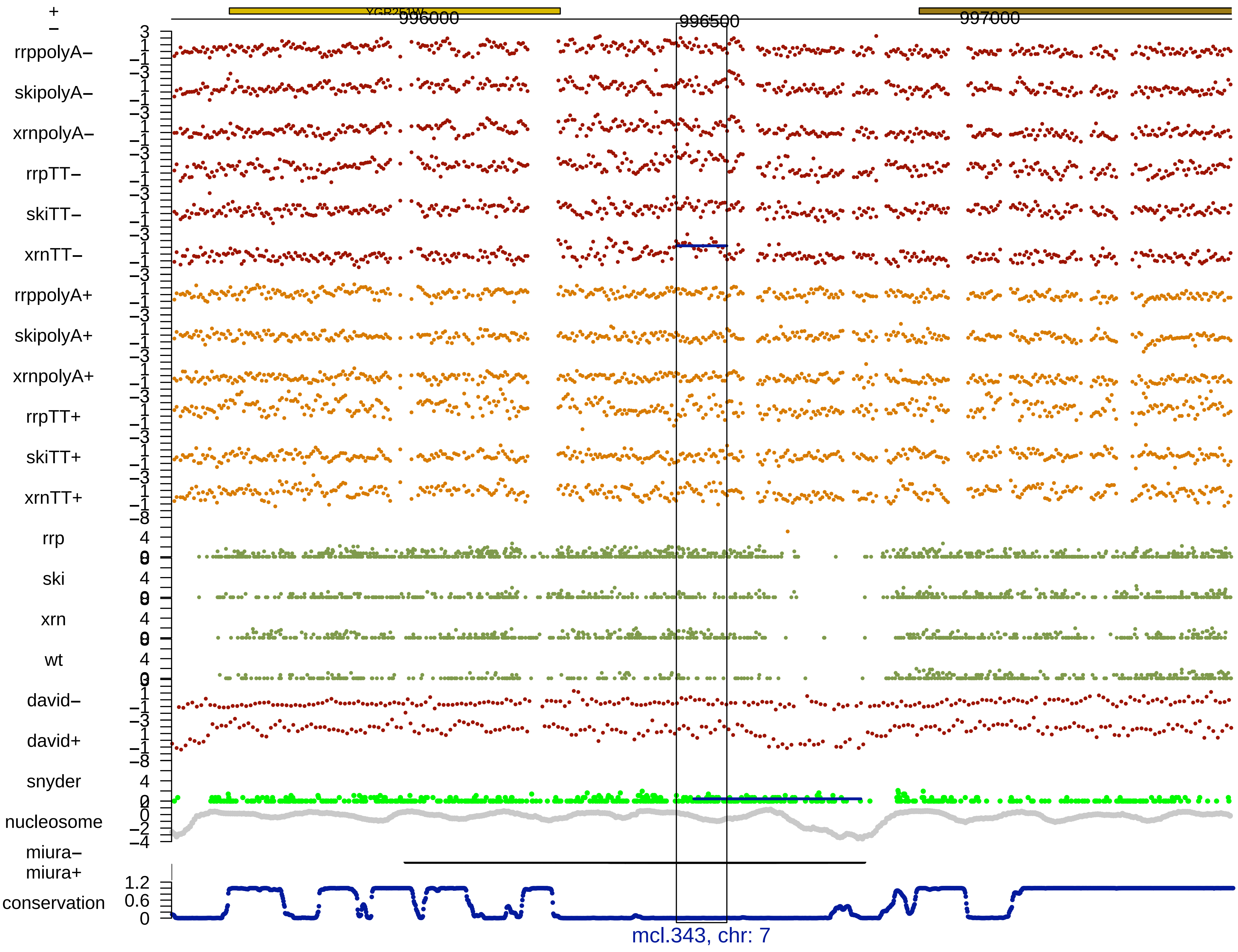

YGR251W
996000
996500
997000
+
−
rrppolyA−
skipolyA−
xrnpolyA−
rrpTT−
skiTT−
xrnTT−
rrppolyA+
skipolyA+
xrnpolyA+
rrpTT+
skiTT+
xrnTT+
rrp
ski
xrn
wt
david−
david+
snyder
nucleosome
miura−
miura+
conservation
mcl.343, chr: 7

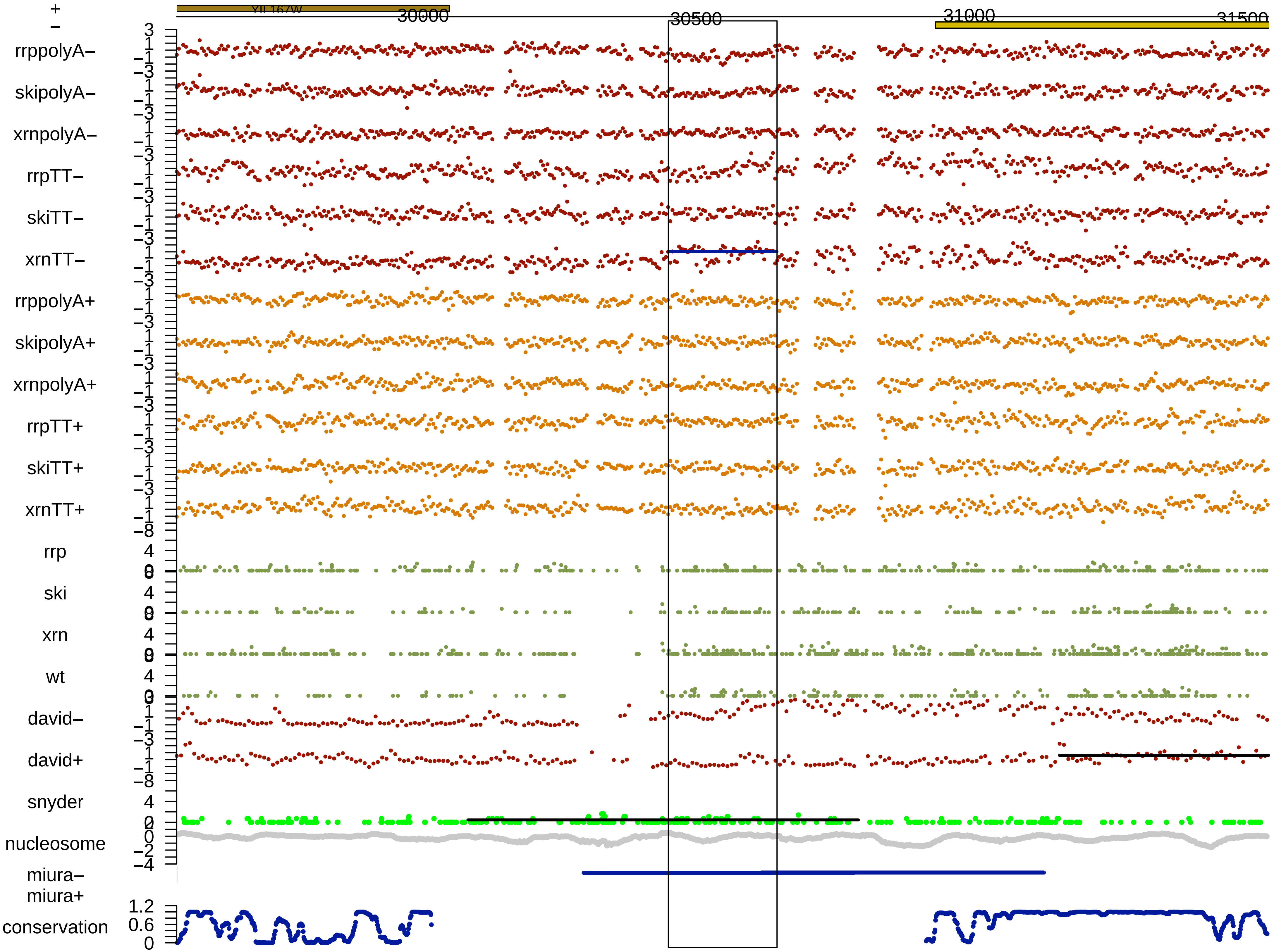
YIL167W
30000
30500
31000
31500
rrppolyA−
skipolyA−
xrnpolyA−
rrpTT−
skiTT−
xrnTT−
rrppolyA+
skipolyA+
xrnpolyA+
rrpTT+
skiTT+
xrnTT+
rrp
ski
xrn
wt
david−
david+
snyder
nucleosome
miura−
miura+
conservation

mcl.344, chr: 9

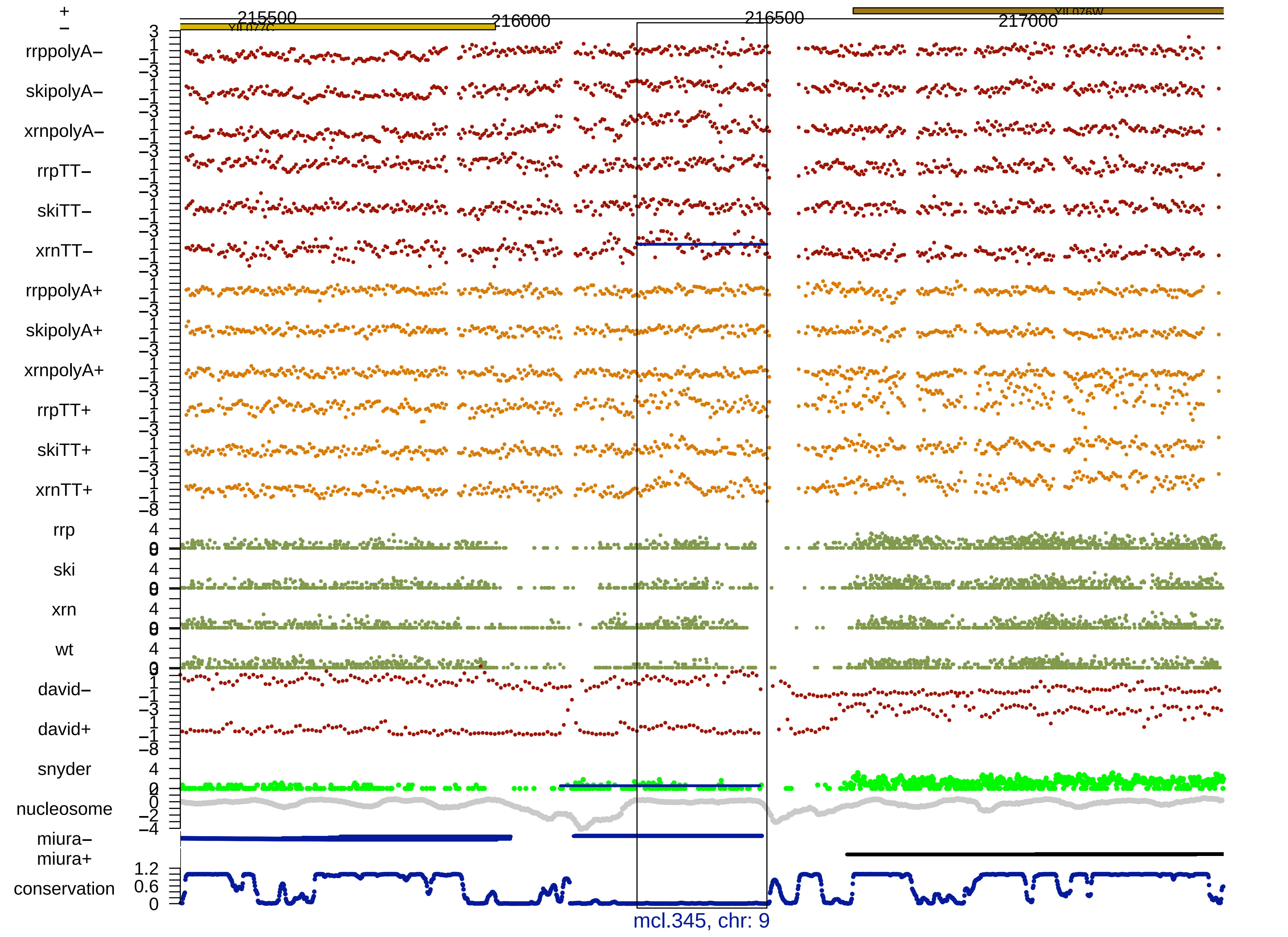

YIL077C
YIL076W
215500
216000
216500
217000
rrppolyA−
skipolyA−
xrnpolyA−
rrpTT−
skiTT−
xrnTT−
rrppolyA+
skipolyA+
xrnpolyA+
rrpTT+
skiTT+
xrnTT+
rrp
ski
xrn
wt
david−
david+
snyder
nucleosome
miura−
miura+
conservation
mcl.345, chr: 9

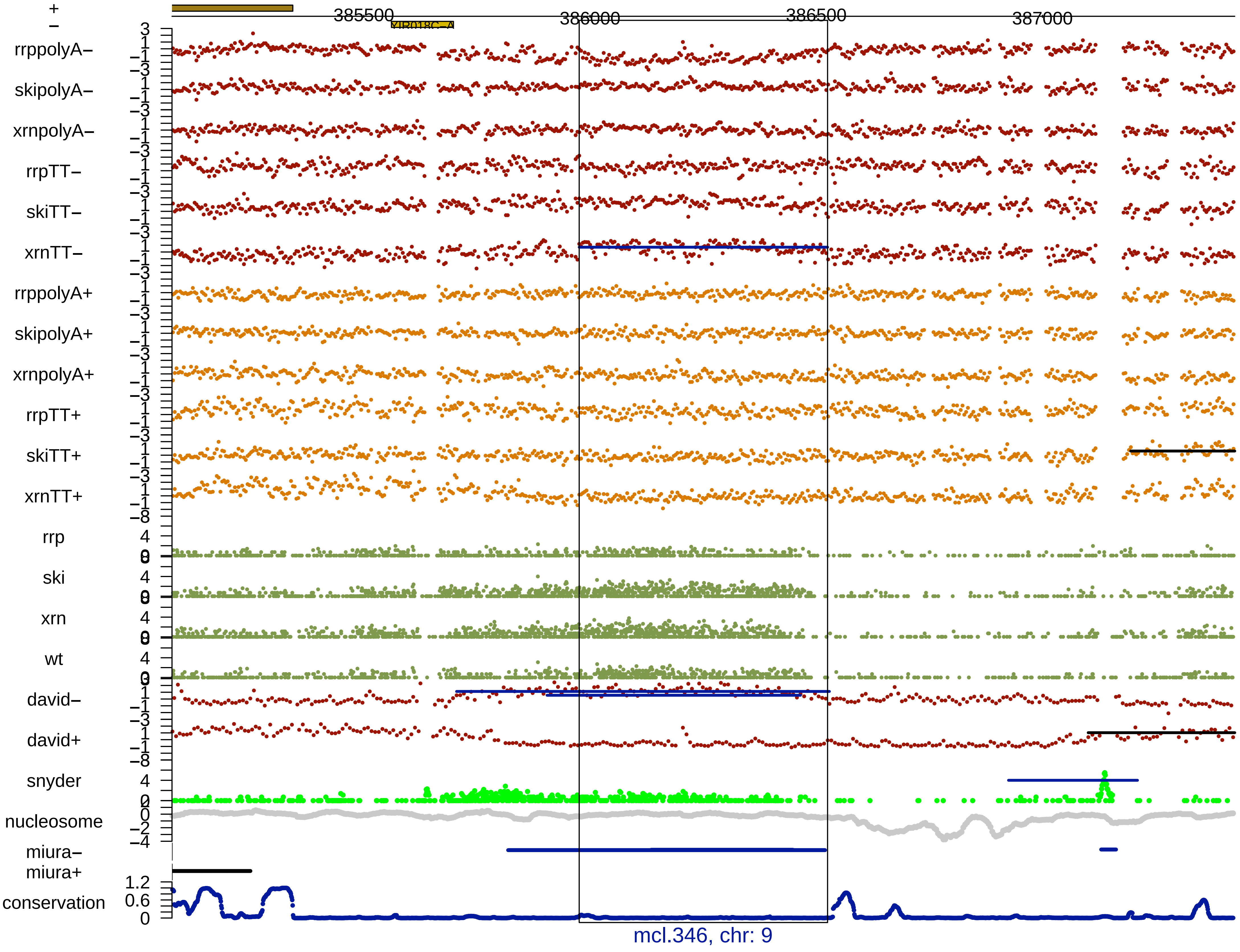

385500
386000
386500
387000
rrppolyA−
skipolyA−
xrnpolyA−
rrpTT−
skiTT−
xrnTT−
rrppolyA+
skipolyA+
xrnpolyA+
rrpTT+
skiTT+
xrnTT+
rrp
ski
xrn
wt
david−
david+
snyder
nucleosome
miura−
miura+
conservation
mcl.346, chr: 9

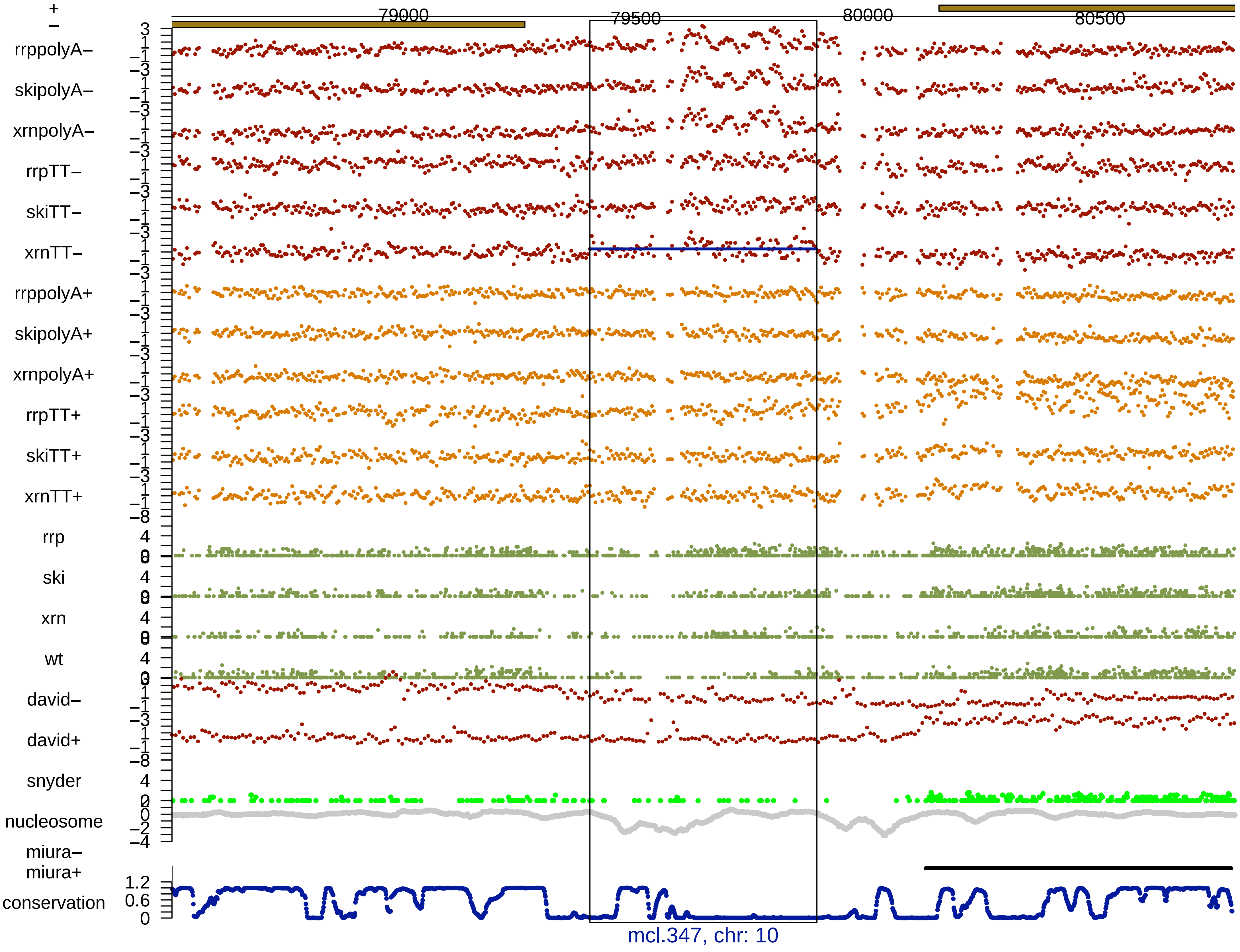

+
−
79000
79500
80000
80500
rrppolyA−
skipolyA−
xrnpolyA−
rrpTT−
skiTT−
xrnTT−
rrppolyA+
skipolyA+
xrnpolyA+
rrpTT+
skiTT+
xrnTT+
rrp
ski
xrn
wt
david−
david+
snyder
nucleosome
miura−
miura+
conservation
3
1
−1
−3
−8
4
0
2
−2
−4
1.2
0.6
mcl.347, chr: 10

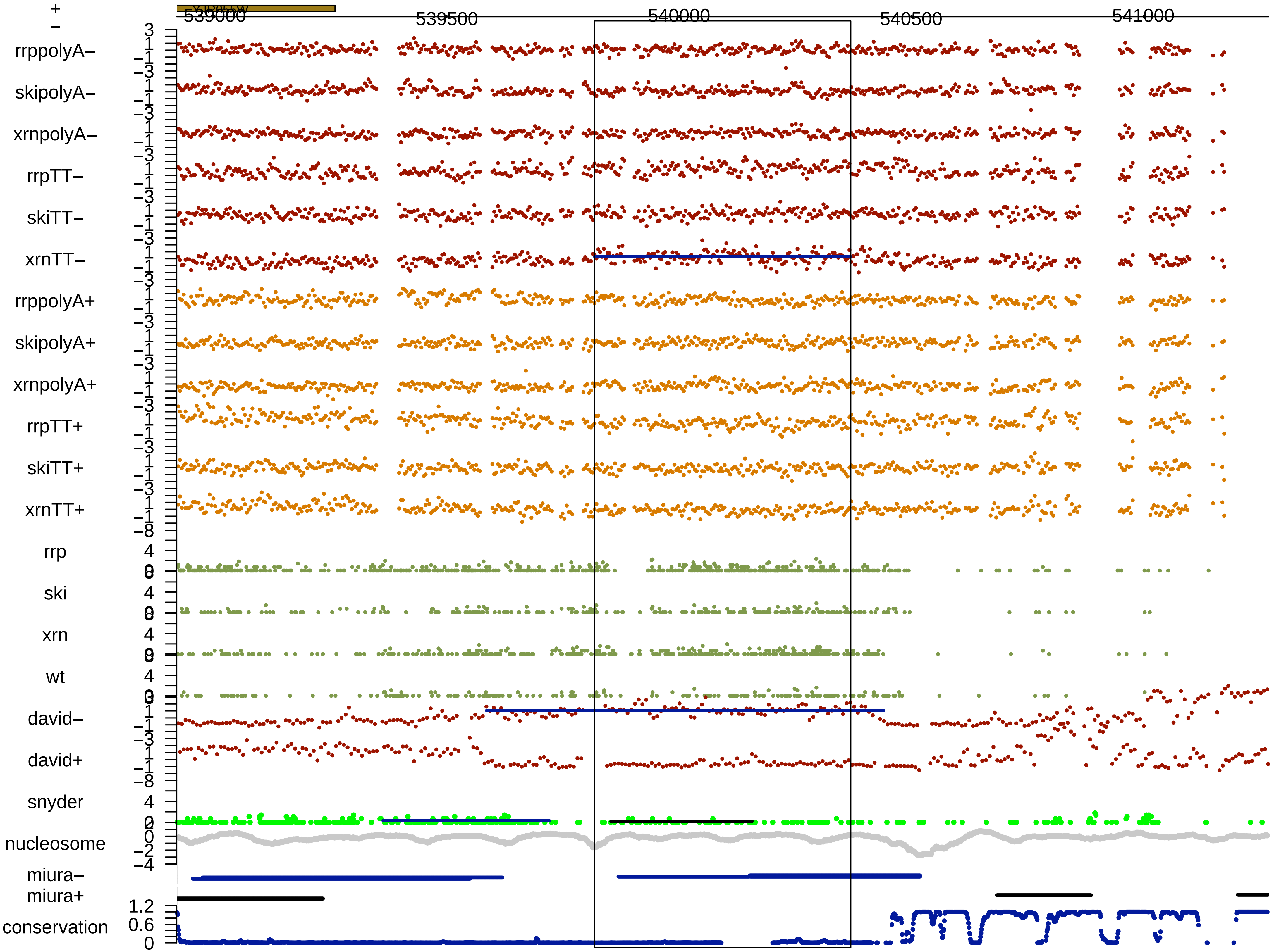

mcl.348, chr: 10

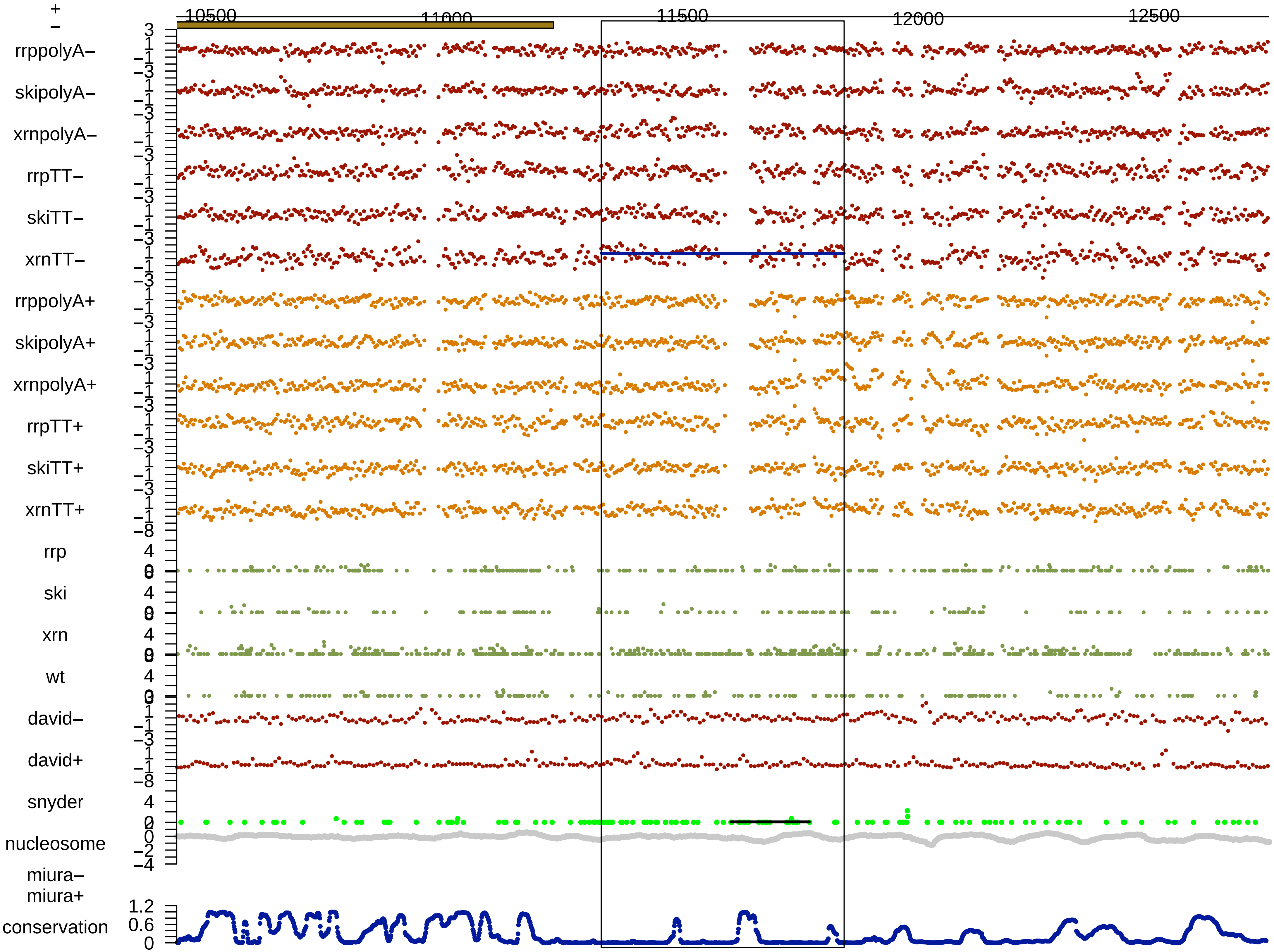

mcl.349, chr: 11

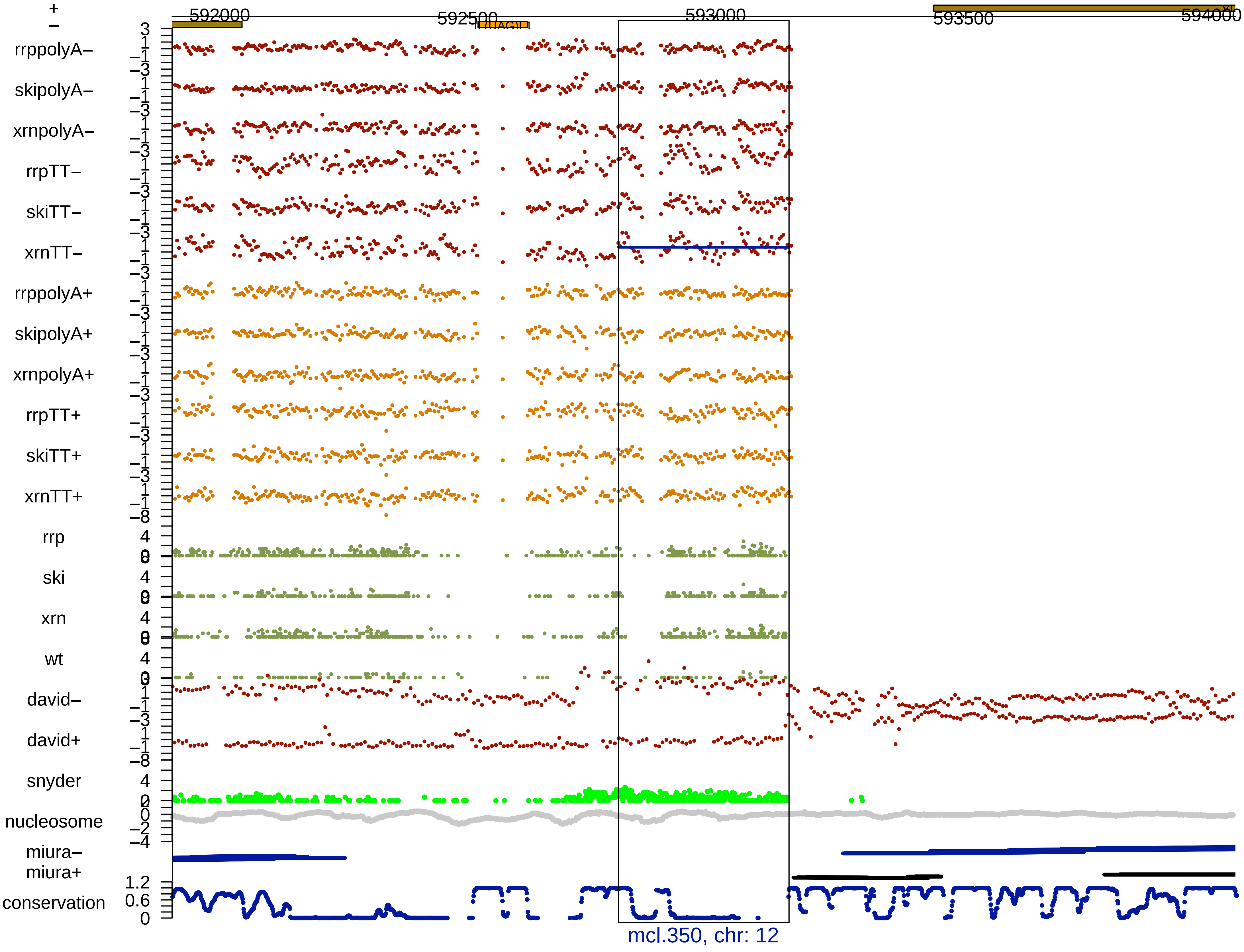
592000
592500
593000
593500
594000
rrppolyA–
skipolyA–
xrnpolyA–
rrpTT–
skiTT–
xrnTT–
rrppolyA+
skipolyA+
xrnpolyA+
rrpTT+
skiTT+
xrnTT+
rrp
ski
xrn
wt
david–
david+
snyder
nucleosome
miura–
miura+
conservation
mcl.350, chr: 12

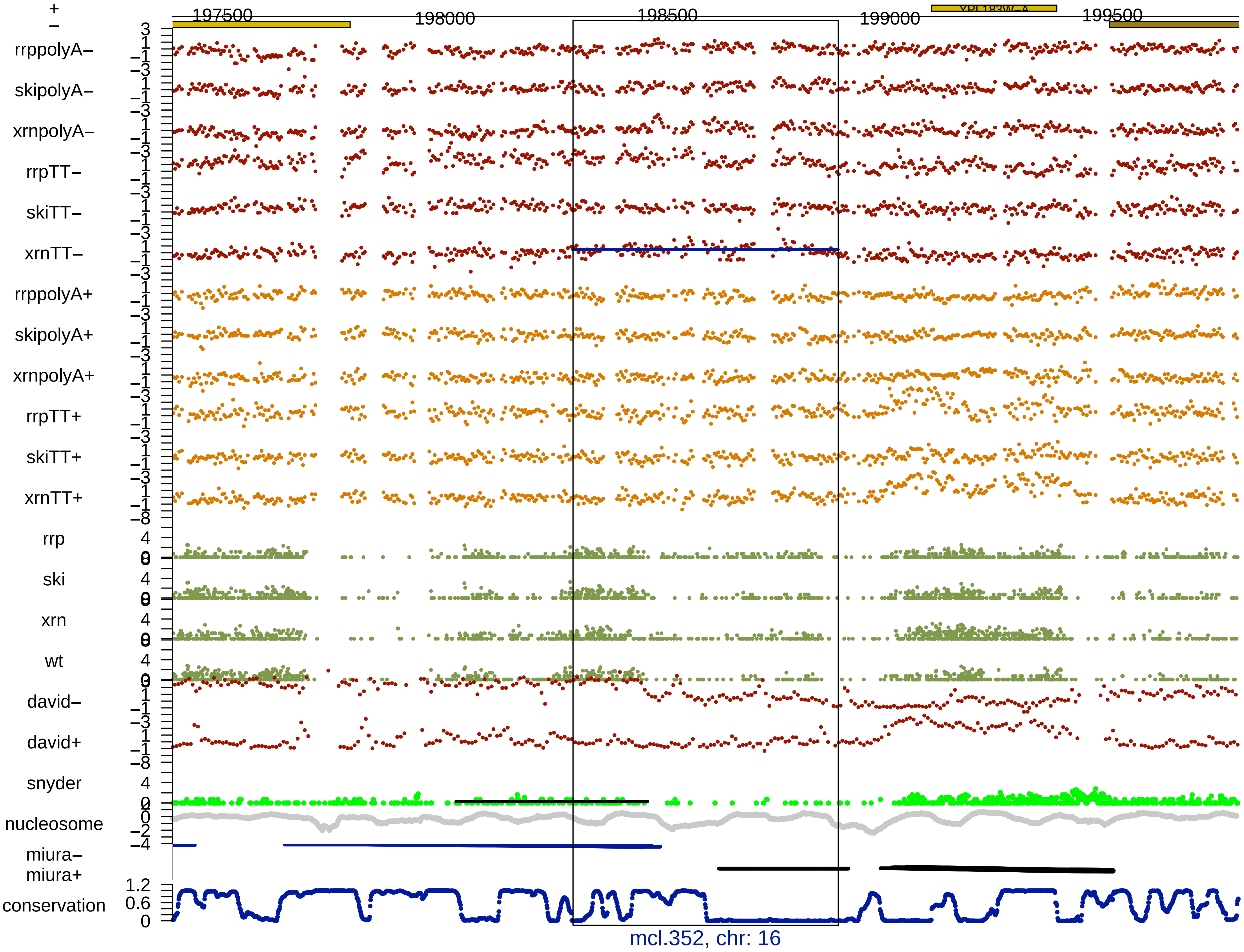

+
−
197500
198000
198500
199000
199500
rrppolyA−
skipolyA−
xrnpolyA−
rrpTT−
skiTT−
xrnTT−
rrppolyA+
skipolyA+
xrnpolyA+
rrpTT+
skiTT+
xrnTT+
rrp
ski
xrn
wt
david−
david+
snyder
nucleosome
miura−
miura+
conservation
mcl.352, chr: 16

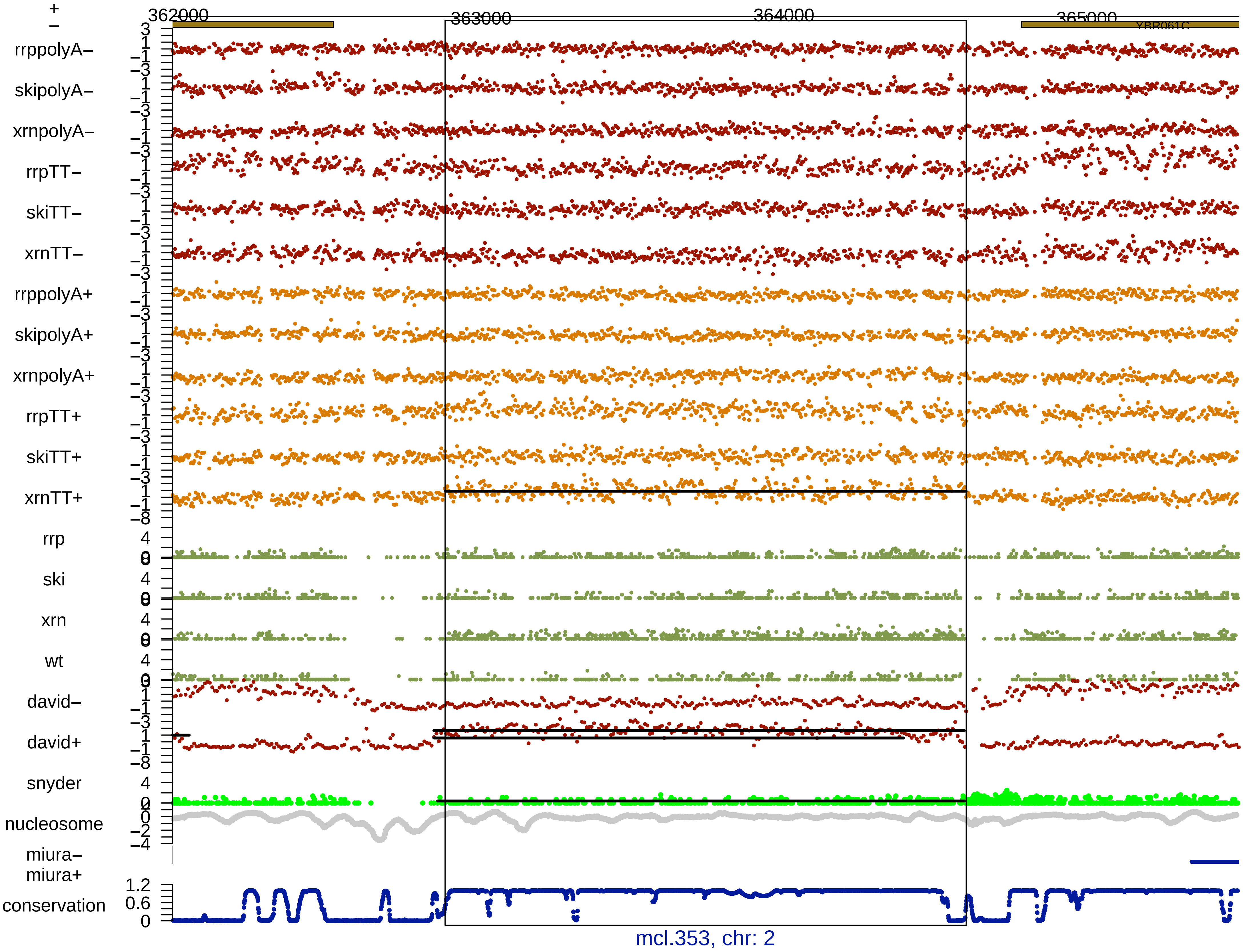

362000
363000
364000
365000
YBR061C
rrppolyA−
skipolyA−
xrnpolyA−
rrpTT−
skiTT−
xrnTT−
rrppolyA+
skipolyA+
xrnpolyA+
rrpTT+
skiTT+
xrnTT+
rrp
ski
xrn
wt
david−
david+
snyder
nucleosome
miura−
miura+
conservation
mcl.353, chr: 2

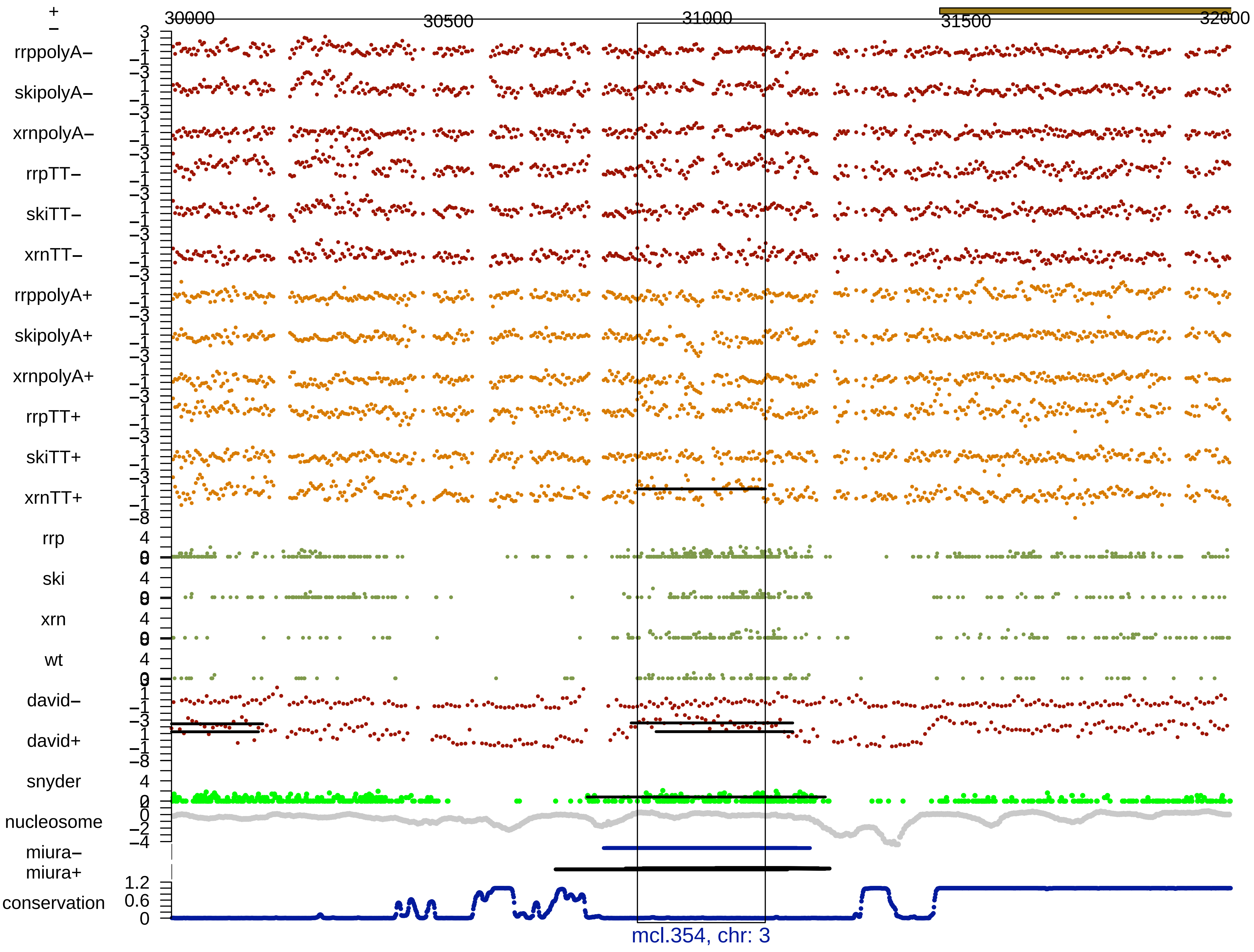

30000
30500
31000
31500
32000
rrppolyA−
skipolyA−
xrnpolyA−
rrpTT−
skiTT−
xrnTT−
rrppolyA+
skipolyA+
xrnpolyA+
rrpTT+
skiTT+
xrnTT+
rrp
ski
xrn
wt
david−
david+
snyder
nucleosome
miura−
miura+
conservation
mcl.354, chr: 3

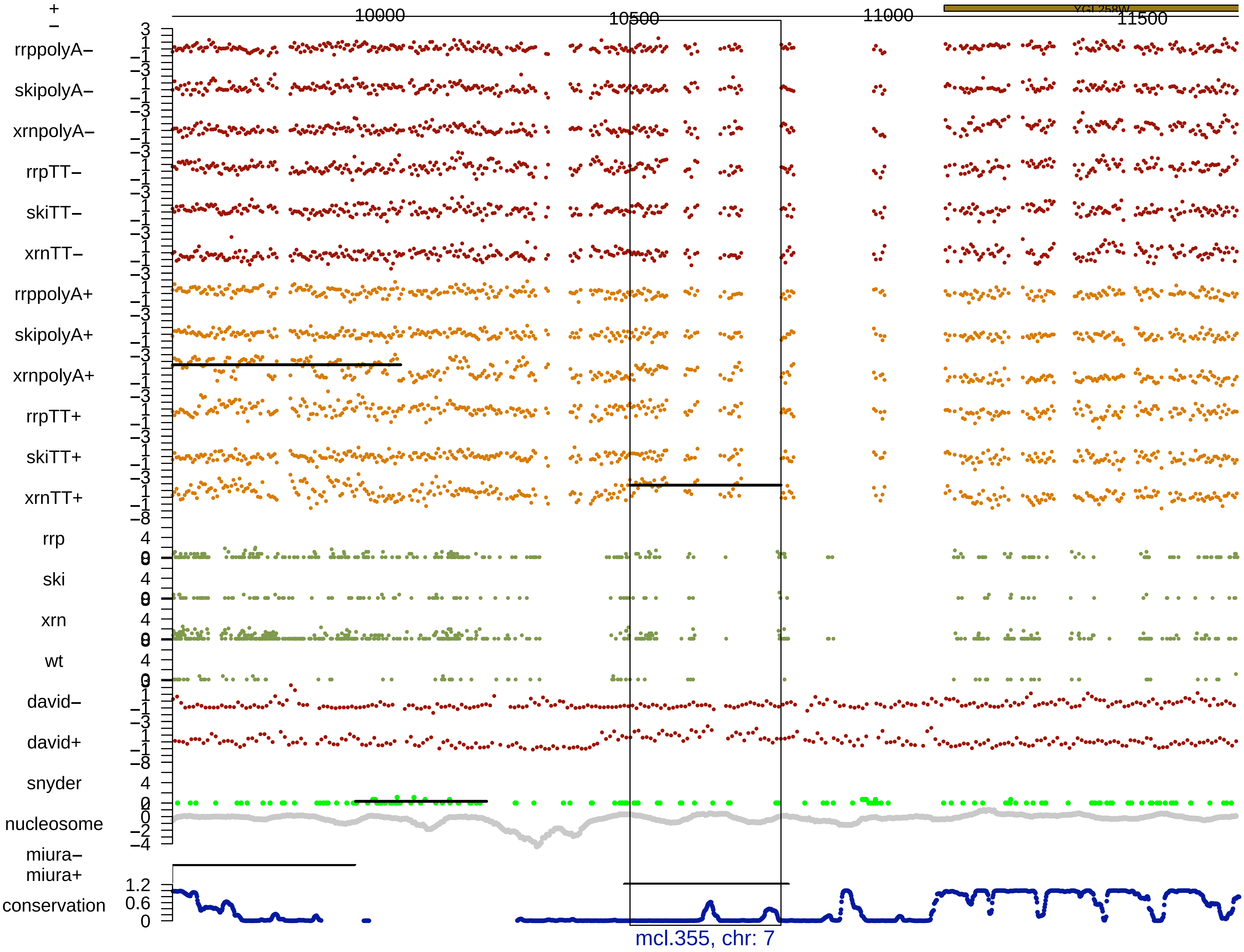

+
−
10000
10500
11000
11500
YGL258W
rrppolyA−
skipolyA−
xrnpolyA−
rrpTT−
skiTT−
xrnTT−
rrppolyA+
skipolyA+
xrnpolyA+
rrpTT+
skiTT+
xrnTT+
rrp
ski
xrn
wt
david−
david+
snyder
nucleosome
miura−
miura+
conservation
mcl.355, chr: 7

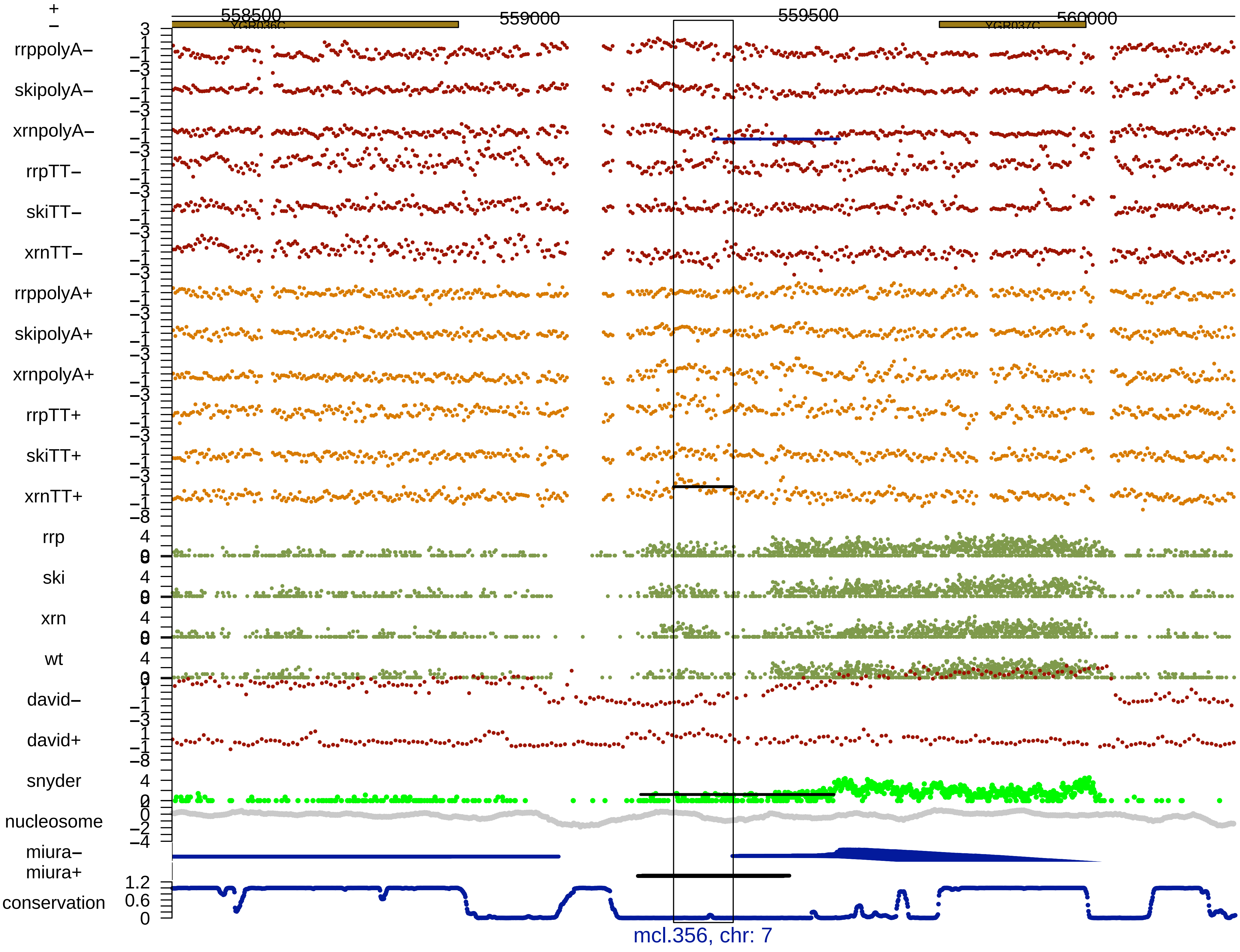
558500
559000
559500
560000
rrppolyA−
skipolyA−
xrnpolyA−
rrpTT−
skiTT−
xrnTT−
rrppolyA+
skipolyA+
xrnpolyA+
rrpTT+
skiTT+
xrnTT+
rrp
ski
xrn
wt
david−
david+
snyder
nucleosome
miura−
miura+
conservation
mcl.356, chr: 7

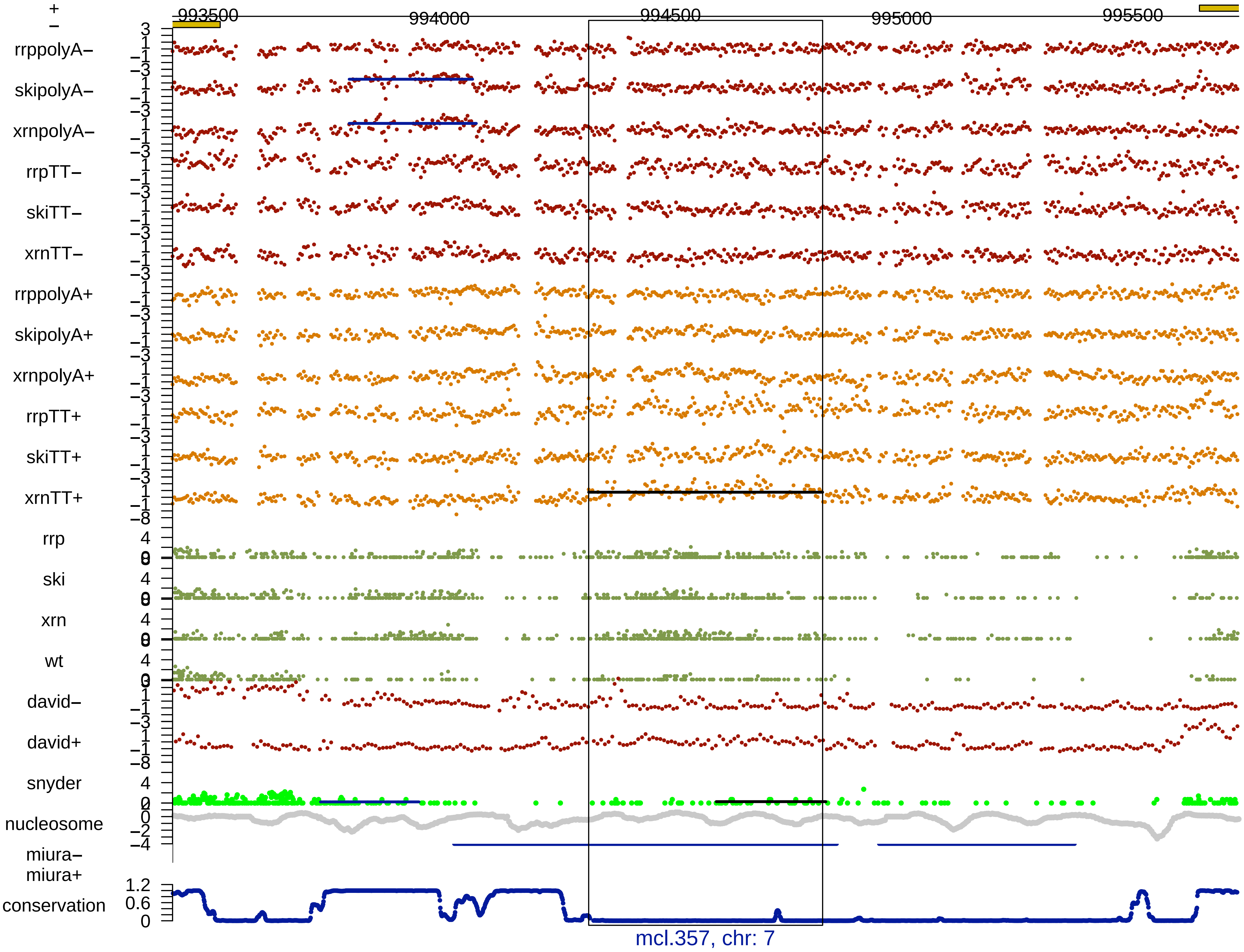

993500
994000
994500
995000
995500
rrppolyA−
skipolyA−
xrnpolyA−
rrpTT−
skiTT−
xrnTT−
rrppolyA+
skipolyA+
xrnpolyA+
rrpTT+
skiTT+
xrnTT+
rrp
ski
xrn
wt
david−
david+
snyder
nucleosome
miura−
miura+
conservation
mcl.357, chr: 7

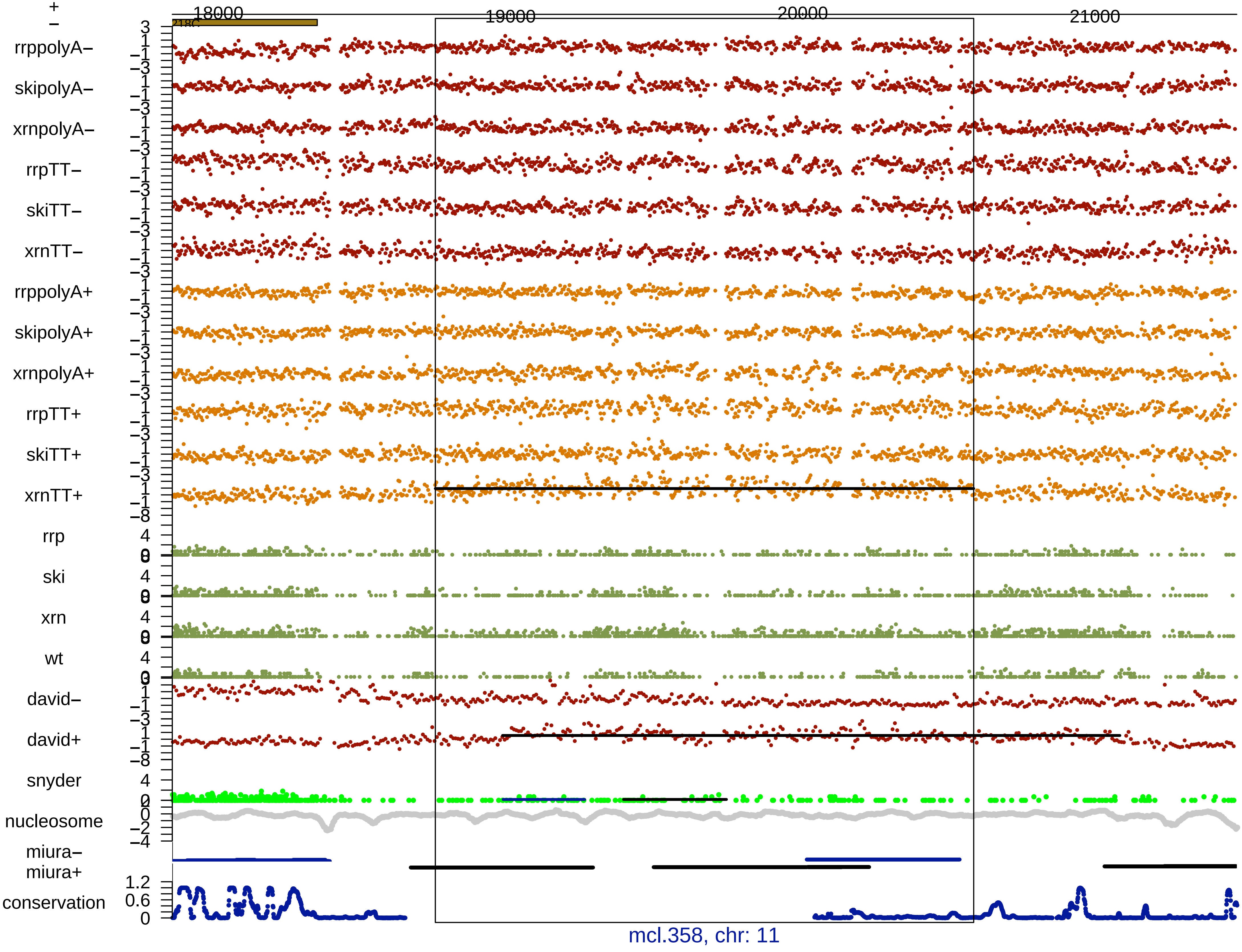

18000
19000
20000
21000
+
−
rrppolyA−
skipolyA−
xrnpolyA−
rrpTT−
skiTT−
xrnTT−
rrppolyA+
skipolyA+
xrnpolyA+
rrpTT+
skiTT+
xrnTT+
rrp
ski
xrn
wt
david−
david+
snyder
nucleosome
miura−
miura+
conservation
mcl.358, chr: 11

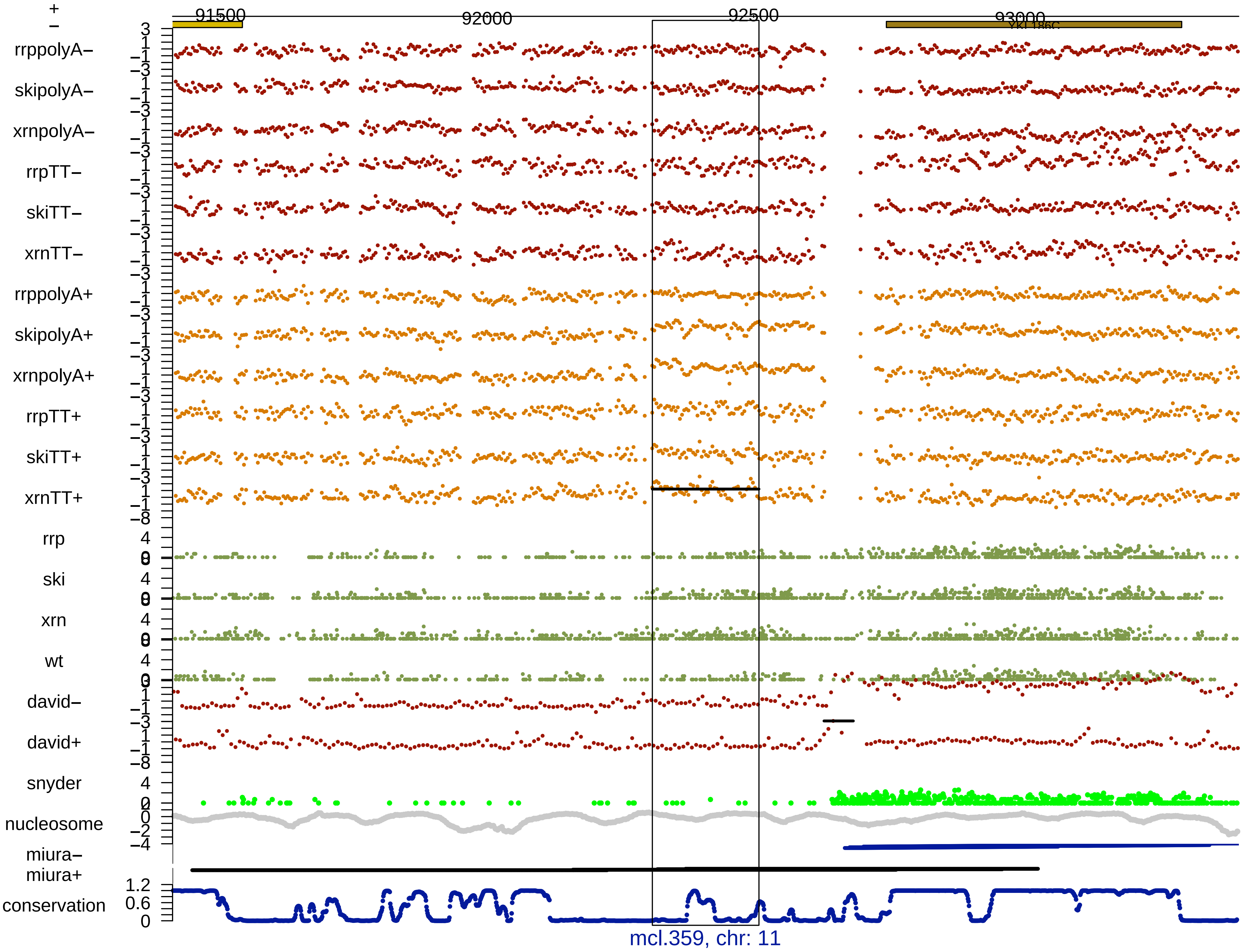

+
−
91500
92000
92500
93000
rrppolyA−
skipolyA−
xrnpolyA−
rrpTT−
skiTT−
xrnTT−
rrppolyA+
skipolyA+
xrnpolyA+
rrpTT+
skiTT+
xrnTT+
rrp
ski
xrn
wt
david−
david+
snyder
nucleosome
miura−
miura+
conservation
mcl.359, chr: 11

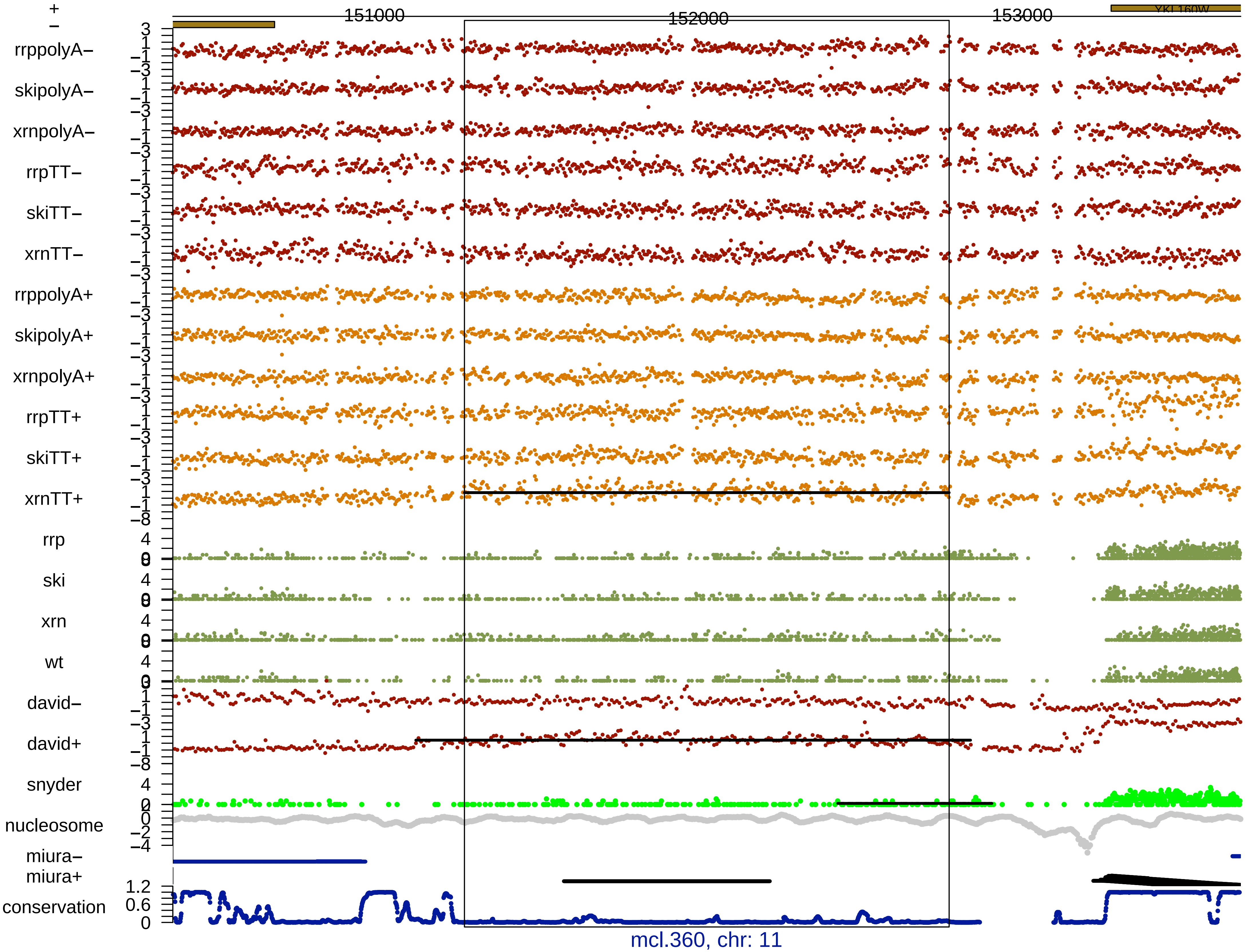

+
−
151000
152000
153000
YKL160W
rrppolyA−
skipolyA−
xrnpolyA−
rrpTT−
skiTT−
xrnTT−
rrppolyA+
skipolyA+
xrnpolyA+
rrpTT+
skiTT+
xrnTT+
rrp
ski
xrn
wt
david−
david+
snyder
nucleosome
miura−
miura+
conservation
mcl.360, chr: 11

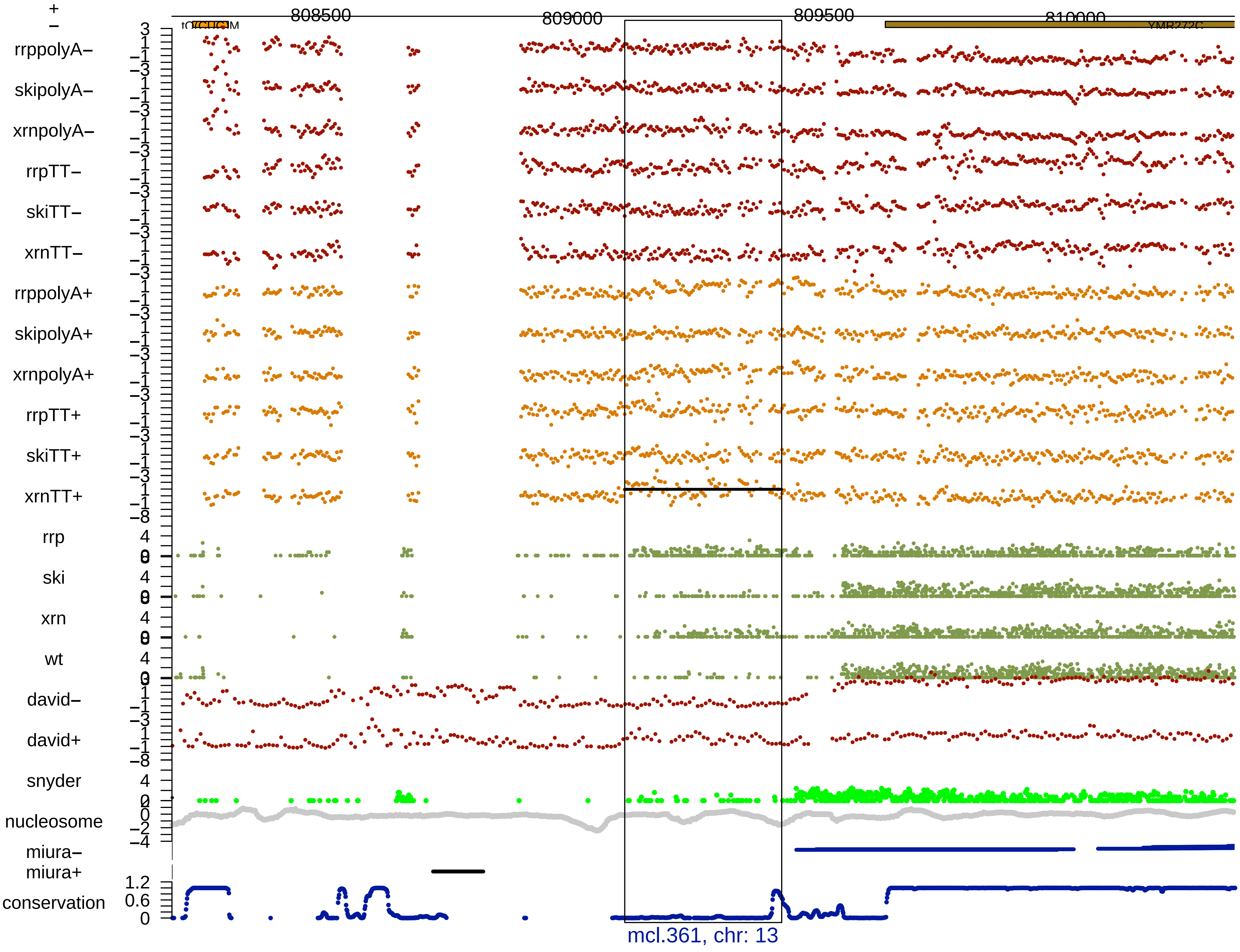

808500
809000
809500
810000
YMR272C
rrppolyA−
skipolyA−
xrnpolyA−
rrpTT−
skiTT−
xrnTT−
rrppolyA+
skipolyA+
xrnpolyA+
rrpTT+
skiTT+
xrnTT+
rrp
ski
xrn
wt
david−
david+
snyder
nucleosome
miura−
miura+
conservation
mcl.361, chr: 13

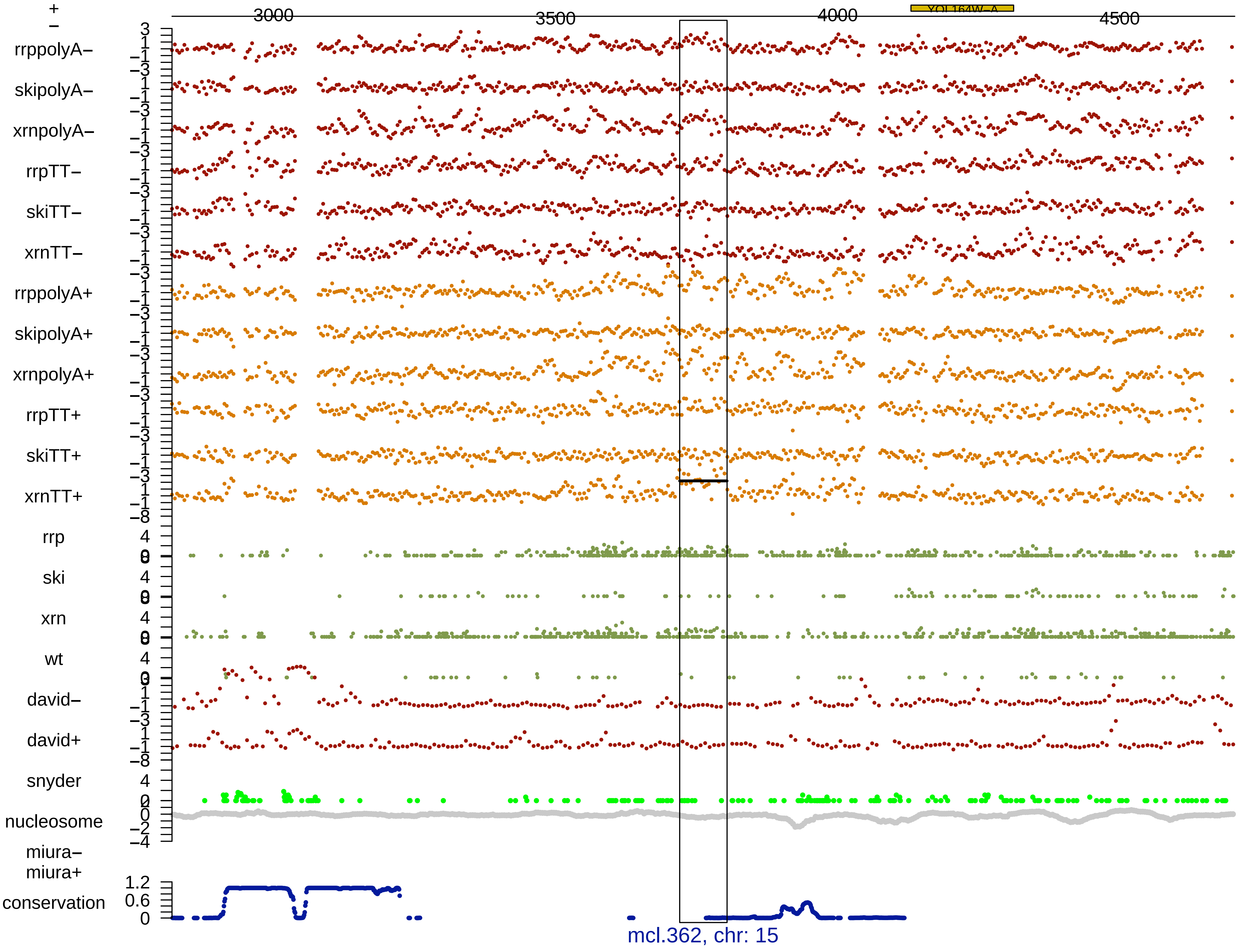

+
–
3000
3500
4000
4500
YOL164W-A
rrppolyA–
skipolyA–
xrnpolyA–
rrpTT–
skiTT–
xrnTT–
rrppolyA+
skipolyA+
xrnpolyA+
rrpTT+
skiTT+
xrnTT+
rrp
ski
xrn
wt
david–
david+
snyder
nucleosome
miura–
miura+
conservation
3
1
–1
–3
–8
4
0
2
–2
–4
1.2
0.6
mcl.362, chr: 15

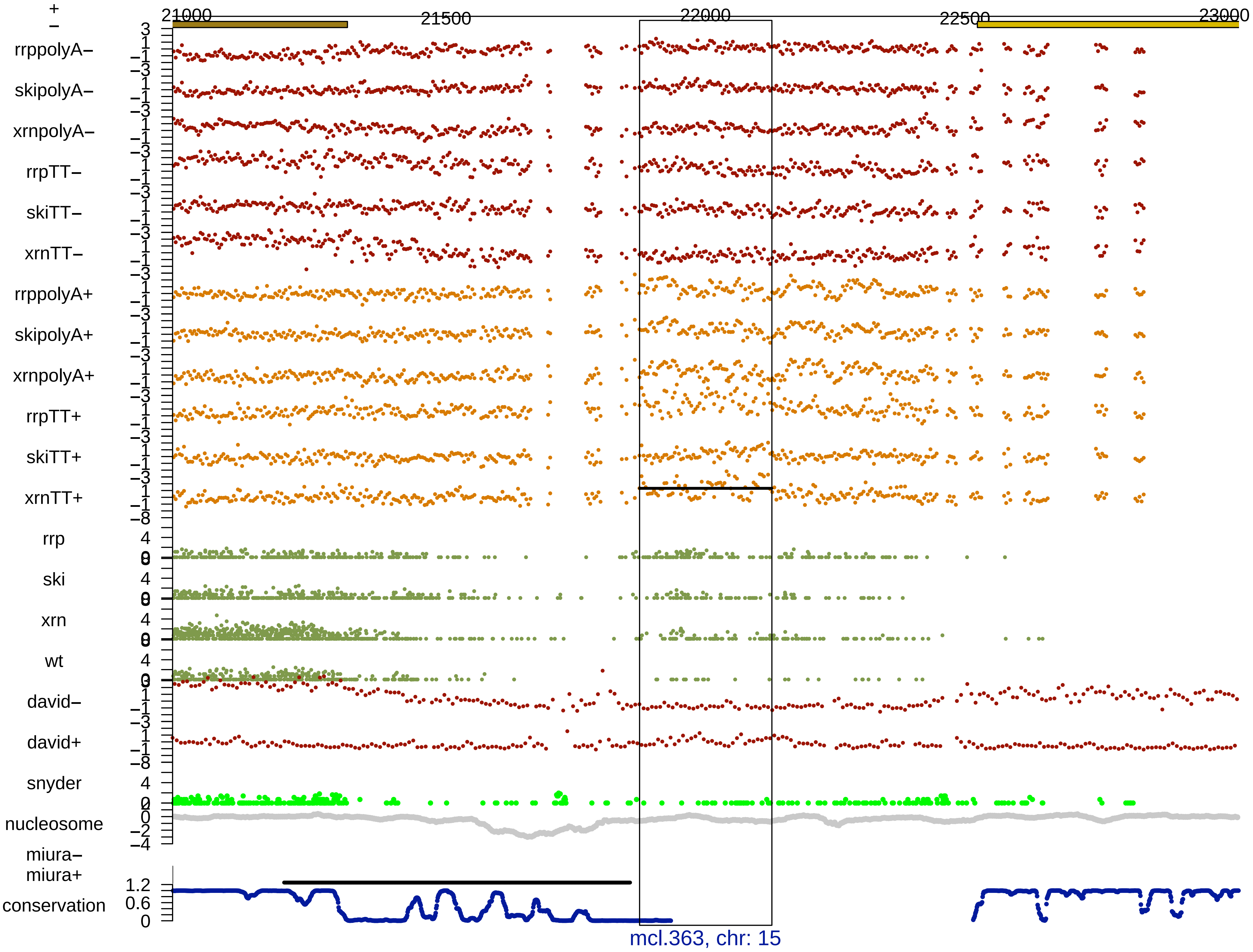

+
−
21000
21500
22000
22500
23000
rrppolyA−
skipolyA−
xrnpolyA−
rrpTT−
skiTT−
xrnTT−
rrppolyA+
skipolyA+
xrnpolyA+
rrpTT+
skiTT+
xrnTT+
rrp
ski
xrn
wt
david−
david+
snyder
nucleosome
miura−
miura+
conservation
mcl.363, chr: 15

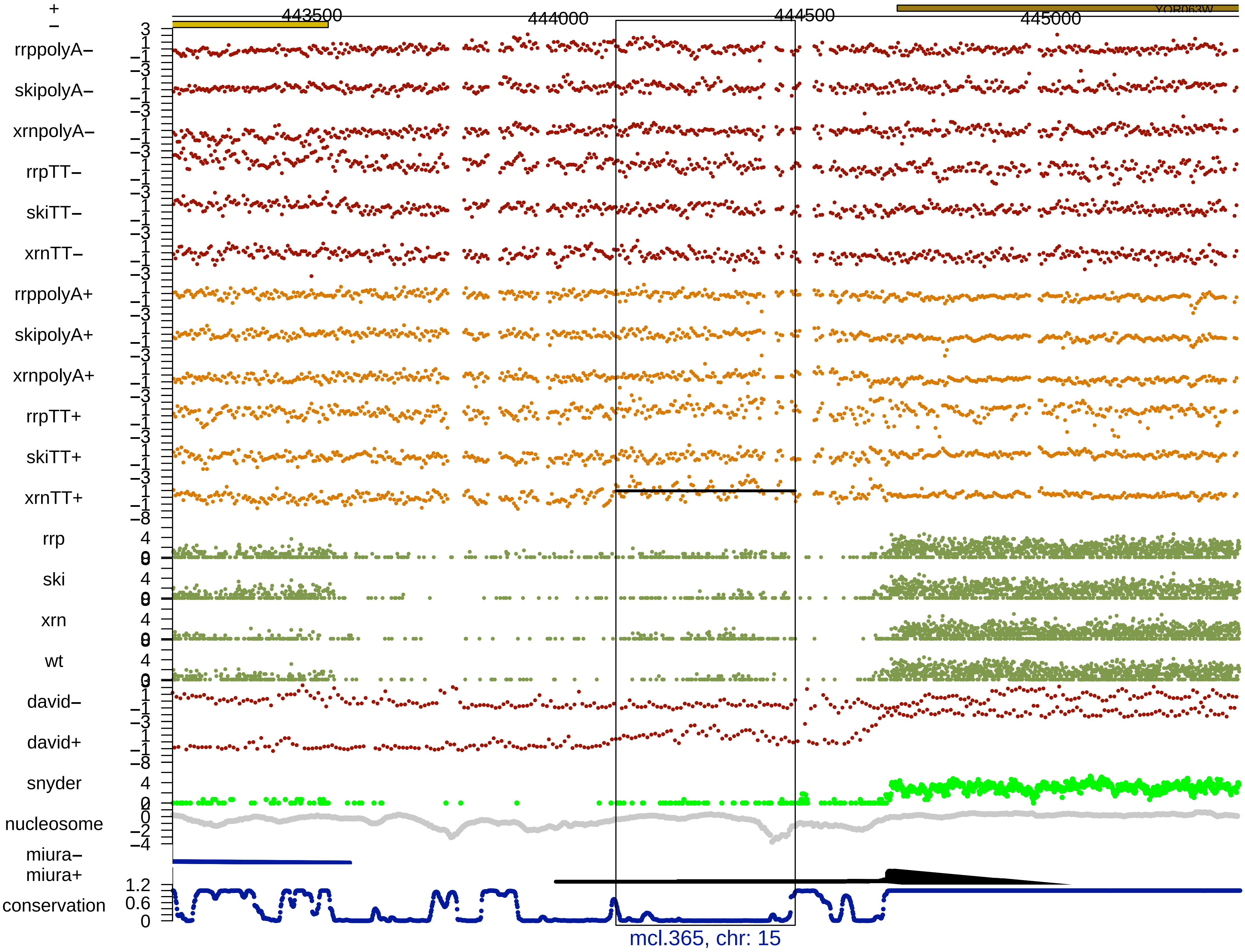
443500
444000
444500
445000
YOR063W
rrppolyA−
skipolyA−
xrnpolyA−
rrpTT−
skiTT−
xrnTT−
rrppolyA+
skipolyA+
xrnpolyA+
rrpTT+
skiTT+
xrnTT+
rrp
ski
xrn
wt
david−
david+
snyder
nucleosome
miura−
miura+
conservation
mcl.365, chr: 15

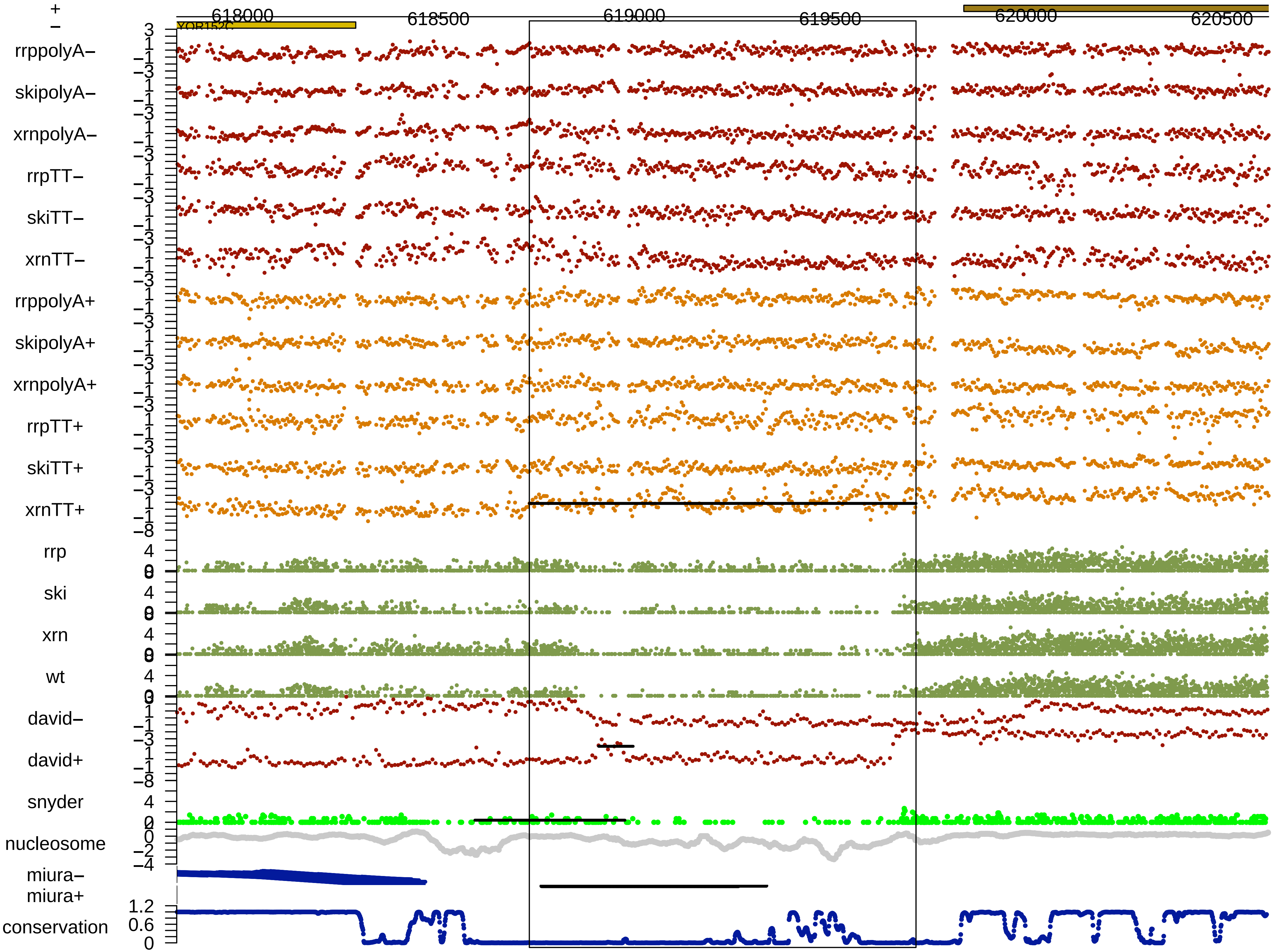

mcl.366, chr: 15
